# Sex differences in human skeletal muscle fiber types and the influence of age, physical activity, and muscle group: A systematic review and meta‐analysis

**DOI:** 10.14814/phy2.70616

**Published:** 2025-11-02

**Authors:** Jessica J. James, Maddison L. Mellow, Elizabeth P. Bueckers, Savannah B. Gutsch, David J. Wrucke, Andrew G. Pearson, Ashleigh E. Smith, Sandra K. Hunter

**Affiliations:** ^1^ Exercise and Rehabilitation Science Program, Department of Physical Therapy Marquette University Milwaukee Wisconsin USA; ^2^ Alliance for Research in Exercise, Nutrition and Activity (ARENA) Research Centre, Allied Health and Human Performance University of South Australia Adelaide South Australia Australia; ^3^ School of Kinesiology University of Michigan Ann Arbor Michigan USA

**Keywords:** aging, fiber types, physical activity, sex differences, skeletal muscle

## Abstract

To understand the sex differences in human skeletal muscle fibers, we determined whether sex differences in fiber cross‐sectional area (CSA), fiber type distribution, and proportional area remained after controlling for age, physical activity level, muscle group, and analysis technique. Meta‐analysis was performed on 6222 unique participants (Males, M = 3501; Females, F = 2721) (>18 years and free of disease) extracted from 156 studies. A random‐effects meta‐analysis was used to determine the main effect of sex, and subgroup analyses were performed to determine the influence of age, physical activity level, muscle group biopsied, and analysis technique. Males had greater type I CSA (M = 4936 ± 1250 μm^2^; F = 4151 ± 1074 μm^2^; *p* < 0.001), type II CSA (M = 5272 ± 1950 μm^2^; F = 3483 ± 1309 μm^2^; *p* < 0.001), type II distribution (M = 51.6 ± 14.6%; F = 48.3 ± 13.0%; *p* < 0.001), and type II proportional area (M = 55.0 ± 14.4%; F = 47.9 ± 13.1%; *p* < 0.001) than females. Conversely, females had greater type I distribution (F = 51.4 ± 12.1%; M = 48.3 ± 13.3%; *p* = 0.01) and type I proportional area (F = 51.8 ± 12.4%; M = 44.9 ± 13.2%; *p* < 0.001) than males. Sex differences were moderated by muscle group biopsied in type I and II proportional area and by age in type I and II fiber type distribution but remained in all other subgroup analyses. In healthy adults, males have larger type I and type II CSA, type II fiber type distribution, and type II proportional area than females, while females have greater type I fiber type distribution and type I proportional area than males. Sex differences generally remained regardless of age, physical activity level, muscle group, and analysis technique, indicating inherent biological sex differences in muscle fiber composition of whole skeletal muscle.

## Graphical abstract

Systematic review and meta‐analysis observing sex differences in fiber type size and composition. Shown are the number of studies identified through to the studies and number of males and females included in the analysis. The forest plot summarizes the sex differences in fiber type cross‐sectional area (CSA), fiber type distribution (% number) and proportional area (% of sample area). Males had larger CSA of type I and II fibers, and a greater distribution and proportional area of type II fibers than females. Females had a larger distribution and proportional area of type I fibers than males.

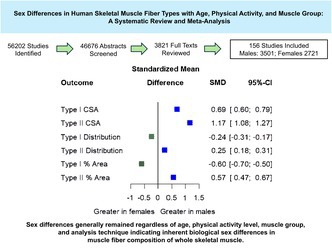

## 1 INTRODUCTION

Skeletal muscle fibers, characterized by differences in myosin heavy chain (MHC) isoforms (I, IIa, and IIx) and their functions, are the basic unit of skeletal muscle. Muscle fibers are highly plastic in size and composition and adapt to chronic perturbations including aging, exercise training and physical activity, and disuse and immobilization. Only in recent years has the influence of potential sex differences in skeletal muscle form and composition been evaluated as an important variable that influences the function of whole muscle (Hunter et al., 2023).

Skeletal muscles of males are larger, stronger, and more powerful than females across the lifespan, primarily due to sex differences in endogenous testosterone concentrations that emerge with male puberty in untrained (Chiu et al., 2020) and athlete populations (Nokoff et al., 2023; Senefeld et al., 2019). Adult males produce ~15‐fold higher testosterone concentrations after the onset of puberty than females, and this hormone has profound effects on skeletal muscle (Clark et al., 2019; Handelsman et al., 2018). For example, lower limb muscles in males are 40% more powerful than in young and older adult females, with even larger sex differences in power in the upper limb muscles (Alcazar et al., 2020; Hunter et al., 2023; Sundberg, Kuplic, et al., 2018; Wrucke et al., 2024). Muscle power is the product of the force (strength) and contraction velocity of the muscles and is therefore dependent upon the collective cross‐sectional area (CSA) of the fibers, the force they exert, and their shortening velocity. Thus, sex differences in power occur because males have larger fibers (CSA) and a greater type II proportional area (percentage of muscle area comprised of each fiber type) than females. This was first described by a review in 2016, which identified sex differences in proportional area by combining data from 14 different research studies (Hunter et al., 2016). Similar findings have since been described in recent reviews (Hunter et al., 2023; Nuzzo, 2023, 2024). It remains unknown; however, whether the sex differences in fiber type CSA, distribution, and proportional area persist with advanced aging, differences in physical activity levels, and across various muscles.

A recent meta‐analysis reported sex differences in proportional area, fiber type distribution (percentage number of fibers), and CSA (Nuzzo, 2024). However, the review (1) included chronic disease populations which may impact potential sex differences typically observed in healthy populations, and (2) did not complete the review according to PRISMA guidelines. To extend and determine the accuracy of the previous analyses on the sex differences in fiber type characteristics, we followed the PRISMA guidelines to erase potential biases. We also excluded known diseased populations that may have affected the sex differences in fiber type characteristics. Because fiber type properties adapt with training (Plotkin et al., 2021; Staron et al., 1994) and differ between different types of athletes (Tesch & Karlsson, 1985), we also examined the influence of physical activity levels on the fiber type size, distribution, and proportional area. Further, advanced age can have profound effects on fiber type characteristics within a muscle, including fiber death and atrophy (particularly type II fibers) (Grosicki et al., 2022; Horwath et al., 2025; Hunter et al., 2016; Lexell et al., 1988; Nilwik et al., 2013; Sundberg et al., 2025; Sundberg, Hunter, et al., 2018; Teigen et al., 2020). Therefore, we also examined the effect of aging on the sex differences in fiber type CSA, distribution, and proportional area. Finally, because the identification of different fiber types has varied across recent decades as techniques have evolved, we performed a subgroup analysis separating techniques that examine enzymatic properties of fibers (i.e., mATPase) and those that examine MHC isoform content (i.e., immunohistochemistry and homogenates).

The purpose of this study was to determine (1) the magnitude of sex differences in skeletal muscle CSA, fiber type distribution, and proportional area, and (2) whether there are sex differences after controlling for age, physical activity level, muscle group biopsied, and technique used to perform analysis. Understanding sex differences in muscle fiber types has implications for understanding sex differences in neuromuscular physiology, determining factors that may drive sex‐specific differences in disease pathology and sport performance, and developing sex‐specific exercise prescription and rehabilitation protocols.

## 2 METHODS

### 2.1 Protocol

The present study adhered to the Preferred Reporting Items for Systematic Reviews and Meta‐Analyses (PRISMA) guidelines and was prospectively registered with PROSPERO (registration ID: CRD42024627234). The 2020 PRISMA checklist can be found in Supplemental File S1.

### 2.2 Search strategy

An electronic literature search, which examined five electronic databases (PubMed, Web of Science, The Cochrane Library, SPORTDiscus, and CINAHL) from inception to February 1st, 2023, was executed to identify studies that performed muscle biopsies on both healthy male and female humans (see Supplemental File S2 for the full search strategy). Terms that related to the intervention (muscle biopsies, fiber type, and myosin heavy chain (see below)) and the comparator (males and females) were used as keywords in the search. The search was rerun to include studies published between February 2nd, 2023, and July 15th, 2024, and was limited to human studies published in English, with all other languages being excluded due to lack of translation capabilities. All search results were exported into a reference manager (EndNote, version 20.6; Clarivate, Philadelphia, PA).

Duplicate studies were removed in EndNote, and the remaining studies were exported into Covidence where additional duplicates were identified and removed. Further information on how articles were screened, selected, and extracted can be found in the following two sections. Next, four independent reviewers conducted the majority of the initial screening of titles and abstracts and evaluated the full‐text versions of potentially relevant studies (EPB, SBG, DJW, and AGP). Each study was screened by two separate reviewers (JJJ and DJW for titles and abstracts; JJJ and AGP for full‐text review). Screening conflicts were resolved via consensus of the two researchers. During screening, an additional search was completed by pearling the reference lists of included studies to find any relevant studies that may not have been identified by the initial search strategy.

### 2.3 Eligibility criteria, identification, and selection of studies

Three primary measures were used to characterize skeletal muscle fiber types: fiber CSA, fiber type distribution, and fiber proportional area. CSA is considered the size of an individual muscle fiber. Fiber type distribution is the percentage number of each fiber type within a sample (i.e., the number of type I and type II fibers, expressed as percentage of all the fibers in the sample) and is sometimes referred to as fiber type composition in studies. Proportional area is the total area percentage each fiber type occupies in a muscle cross section (i.e., accounts for the size and the distribution of the fibers to observe the percentage area each fiber occupies in an area of muscle).

The inclusion criteria for each study were created using a Population, Intervention, Comparator, and Outcome (PICO) framework: (1) the study population (P) was healthy adults ≥18 years of age, with no reported medical diagnosis; (2) the intervention (I) used to obtain the muscle sample was a muscle biopsy to determine CSA, fiber type distribution, and proportional area via one of the following techniques: (a) electrophoretic analysis of myosin heavy chain isoform expression from muscle homogenates, (b) histological analysis of muscle tissue sections analyzing for either myosin heavy chain isoforms (immunohistochemistry [IHC]), or (c) myofibrillar adenosine triphosphatase (mATPase); (3) the comparator (C) was differences between males and females in the same research study when observing the outcome; and (4) the outcome (O) was sex differences in type I and type II CSA, fiber type distribution, or proportional area.

Only published, peer‐reviewed studies were included. No restrictions on the number of participants studied were applied to ensure that all relevant literature was included. Studies with at least one group of males and one group of females which reported separate outcomes by sex were included. It was often unclear whether the participants were categorized by the authors as their biological sex or a specific gender. Throughout this manuscript, we use the terms males and females, in relation to both biological sex terms (male and female) and gender terms (men and women). We recognize that this may have resulted in some misrepresentations. Single‐sex studies and case studies were excluded. No animal studies, studies performed on children, systematic reviews, or meta‐analyses were included. Studies that included patients with any medical diagnosis were excluded unless healthy controls were included in the study, in which case only the controls were included in the analysis. Studies that included healthy individuals or healthy controls but were receiving elective surgery were also excluded. Studies that performed an intervention were excluded unless baseline data were presented, in which case only the baseline data were included in the analyses. Single‐fiber studies were excluded as type II fibers are more fragile and excluded when broken, potentially decreasing the type II characteristics (Grosicki et al., 2022; Trappe et al., 2003; Yu et al., 2007). Studies that performed fiber typing via MRI were excluded as this is not a validated technique to determine individual fiber types in both males and females (Baguet et al., 2011).

All studies had to provide appropriate variance of means to be included in the meta‐analyses. Four studies (Behan et al., 2002; Brooke & Kaiser, 1970; Mattiello‐Sverzut & Martins, 2023; Ringqvist, 1971; Sharanya et al., 2019) met all criteria, although they did not provide analyzable variance of means. After attempts to contact the authors, we were unsuccessful in obtaining data that could be included in the meta‐analyses; therefore, these studies were excluded from final analyses. A complete list of all studies excluded during the full text review, along with the reason for exclusion, can be found in Supplemental File S3.

### 2.4 Data extraction and analysis

Four authors extracted relevant data from the studies included (EPB, SBG, DJW, and AGP) and entered the data into a spreadsheet (Excel, version 16.89.1; Microsoft, Redmond, WA) created by the primary researcher (JJJ). A separate author cross‐checked all the data each author had extracted (JJJ). Discrepancies were resolved via consensus of the two researchers. A third screening of the extracted data was performed with the aim of identifying studies with duplicated results (i.e., the same participants biopsied). The initial screening was conducted by inspecting similar data, similar participant characteristics (including number of study participants and physical activity levels), and studies published by the same authors and determining if data were identical across studies. A subsequent screening was performed by reading the methods section of each study to see if the data was reported elsewhere. Once all studies with duplicate data were identified and it was deemed that the participants were likely the same across studies, the study was included, but the sample size was reduced proportionally based on the number of overlapping groups to reduce the weight of the study in the analyses. For example, if there were two studies that had duplicate data, the sample size was halved, but if there were three studies that had duplicate data, the sample size was reduced into thirds. In some cases, studies with the same data only included duplicate data of one or two of the main outcome variables (e.g., one study included CSA, fiber type distribution, and proportional area, and another study with the same participants only included CSA). In these cases, the number of participants was reduced for the specific duplicated outcome variable and not the whole study. In cases where reducing sample size resulted in a fraction, the sample size was rounded up to the nearest whole number. Studies with the same participants but multiple different muscles biopsied were included. An additional sensitivity analysis was performed which removed studies with duplicate data to ascertain whether findings differed when only included once. This analysis did not significantly alter effect sizes for any outcomes and therefore is not reported in this manuscript.

The following variables were extracted from each study: title, author names, year of publication, country of publication, aims of publication, method of recruitment, study design, physical activity level, number of participants, number of males and females, limitations of study, muscle biopsied, and technique used to perform analysis. Means and standard deviation (SD) of age, number of fibers analyzed, CSA, fiber type distribution, proportional area, and fiber diameter were also extracted. Studies that reported standard error (SE) as variance were converted to SD by the following equation: SE × square root of sample size. Studies that reported range as variance were converted to SE by the following: choosing the conversion factor for estimating the SD based on the sample size (Snedecor, 1946), calculating the total range (maximum–minimum), and then multiplying the range by the conversion factor. Studies that reported confidence intervals as variance were calculated as suggested by the Cochrane Handbook for Systematic Review of Interventions (Part 2 Section 7.7.3.2). Studies that reported interquartile range as variance were excluded from the analysis (*n* = 1). Studies that included means only for the muscle fiber type outcomes of interest (i.e., no SD or variance metric) were excluded from analyses (*n* = 4). If authors presented data in figures only, data were extracted using a graph digitizer (https://plotdigitizer.com/app). When studies included more than one group of males and females (e.g., sedentary group and not sedentary group, multiple age groups), each group of males and females were extracted as a separate study to perform appropriate sex difference comparisons. Each group was given an identifying name comprising the first authors' last name, year of publication, and descriptive information identifying the group if more than one group of males and females were reported in a study (e.g., young vs. older, experimental vs. control, sedentary vs. athlete, vastus lateralis vs. biceps brachii).

#### 2.4.1 Fiber type classifications and calculations

In the literature, slow twitch fibers can be classified as type I or MHC I (expressed by the MYH7 gene), and fast twitch fibers can be classified as type II or MHC II, type IIa or MHC IIa (expressed by the MYH2 gene), type IIx (classified as type IIb in some studies) or MHC IIx (expressed by the MYH1 gene), depending on the fiber type analysis technique. Previous research has found strong correlations between techniques that examine enzymatic properties of fibers (i.e., mATPase) and those that examine MHC isoform content (i.e., IHC and sodium dodecyl sulfate‐polyacrylamide gel electrophoresis (SDS‐PAGE)) (Chalmers & Row, 2011; Damer et al., 2022; Fry et al., 1994; Scott et al., 2001). Because of these strong correlations, and for ease of interpretation of results, we decided to combine all slow twitch fiber classifications for statistical analysis and are henceforth reported as type I. Additionally, all fast twitch fiber classifications were combined for statistical analysis and are henceforth reported as type II. Other rationale for combining IIa and IIx fibers into one group are that many studies identified in our search reported IIa/IIx hybrids in healthy young and older adults, making it difficult to separate IIa and IIx into two separate groups, even though many studies also reported IIx fibers in healthy adults. Further, because IIb fibers were reported as well–although we acknowledge IIb fibers are in trace amounts and are reported in older studies–we decided having one type II group would allow us to make general conclusions about type I versus type II fibers, without excluding many studies. The process for combining type IIa and IIx fibers to classify them as solely type II was performed as described subsequently. For studies that reported type IIa and IIx, the means were converted to type II as follows: for CSA, the means were summed and then averaged; for fiber type distribution and proportional area, the percentages were summed. The SD was converted to type II as follows: each SD was squared to get the variance; the variances were then summed to get the total variance; then, the square root of the total variance was calculated to estimate the combined SD. In cases where studies reported type IIa, IIb, and IIc, the same process was followed. Importantly, although the different analysis techniques are highly correlated, a subgroup analysis by technique was performed to determine if this influenced observed sex differences (described below).

If a study reported diameter, CSA was calculated as follows: π × radius^2^. In cases where studies reported CSA, fiber type distribution, or proportional area but did not report all variables and not all could be calculated, authors were contacted to obtain the relevant information. Authors were given 2 weeks to respond. If no response was received, the study was still included, and relevant data was included in analyses. If studies included data on two types of analysis techniques, the following was determined: mATPase was included over IHC for fiber type distribution data, and homogenate data was included over mATPase for proportional area data. The reasoning behind this decision was solely based on which method would include data from the highest number of studies when considering which technique had reported a greater number of variances reported (i.e., SD, SE, etc.).

#### 2.4.2 Subgroup analyses categories

Age was separated into three groups: young, midlife, and older. Age group was determined by the mean age of the participants and whether this was ≤35 years of age (young), between ~35 and 65 years of age (midlife), and ≥65 years of age (older). Physical activity level was separated into two groups: sedentary and not sedentary. Participants were assigned to the sedentary group when authors described them as sedentary, physically inactive, or only performed physical activity in accordance with an ordinary lifestyle. Participants were assigned to the not sedentary group if authors described them as mildly or recreationally active, athletes, or participated in intentional physical activity for a few hours and/or days per week. In addition to author descriptions, if authors reported V̇O_2_max, American College of Sports Medicine (ACSM) V̇O_2_max classifications were used to determine the group. Classifications of “Poor” and below were assigned to the sedentary group. Classifications of “Fair” and above were assigned to the not sedentary group. Studies that did not describe the physical activity levels of the participants or report V̇O_2_max were excluded from the subgroup analysis. Muscle group biopsied was separated into three groups: arm/shoulder, back/trunk, and leg. Analysis technique was separated into three groups: mATPase, IHC, and tissue homogenates. Homogenate data that included single muscle fibers were excluded due to the high fragility of type II fibers, which would potentially bias the data towards a type I fiber type distribution (Grosicki et al., 2022; Trappe et al., 2003; Yu et al., 2007). 14 groups reported two types of analyses. In cases where mATPase and IHC were reported for fiber type distribution, only mATPase was included. In cases where mATPase and tissue homogenates were reported for proportional area, only tissue homogenates were included, as half of the reported mATPase data did not report variance.

#### 2.4.3 Quality assessment

The Newcastle‐Ottawa Scale adapted for cross‐sectional studies was used to score studies on their risk of bias. This appraisal tool consists of seven questions: (1) representativeness of the sample; (2) sample size; (3) non‐respondents; (4) ascertainment of the screening/surveillance tool; (5) control of confounding factors; (6) assessment of the outcome; and (7) appropriate statistical tests. For question 1, studies received a score of 1 if they performed random sampling or had intentional non‐random sampling that was somewhat representative. Studies received a score of 0 if researchers performed a convenience sample or the sampling strategy was not described. For question 2, studies received a score of 1 if they performed a sample size calculation and the sample size was satisfactory. Studies received a score of 0 if the sample was not justified or not discussed. For question 3, studies received a score of 1 if greater than 95% of the participants in the sample were biopsied while studies with < 95% received a score of 0. For question 4, all studies received a score of 2 as only validated analysis techniques were included. For question 5, studies received a score of 1 if the authors performed a subgroup or multivariable analysis investigating sex and skeletal muscle composition. If no statistical analysis was performed controlling for sex, studies received a score of 0. For question 6, studies received a score of 2 if researchers were blinded to participant characteristics during analysis while studies that did not blind analysis or report their methods on personnel regarding analysis received a score of 0. For question 7, studies received a score of 1 if statistical tests regarding the skeletal muscle composition parameters of interest were reported and could be replicated. Studies that did not describe, reported an inadequate amount of information, or performed inappropriate statistical tests received a score of 0. Total scores were summed, and studies were categorized into four groups: very good (8–9 points), good (6–7 points), satisfactory (4–5 points), and unsatisfactory (0–3 points). Risk of bias appraisal was conducted independently by two investigators (EPB and JJJ), and disagreements were resolved by consensus. No studies were excluded based on the quality assessment. This scale can be found in Supplemental File S6.

### 2.5 Statistical analysis

Statistical analyses were conducted using R (version 4.3.1 “Beagle Scouts”). Due to expected heterogeneity between studies, random‐effects meta‐analyses (using “metacont” within the *meta* R package) (Balduzzi et al., 2019) were conducted to generate standardized mean differences (SMD; Hedge's *g*) and their 95% confidence intervals (CIs). All results are reported as weighted means and standard deviations, except in the results section titled ‘Study Characteristics’ where unweighted means are reported. All analyses used an inverse variance method, and test statistics and confidence intervals were adjusted using the Knapp and Hartung method. Effect sizes were interpreted as small (0.2), medium (0.5), or large (>0.8). A total of six main meta‐analyses were conducted to investigate sex differences (male vs. female) in type I CSA, type II CSA, type I fiber type distribution, type II fiber type distribution, type I proportional area, and type II proportional area. Additionally, four subgroup analyses were conducted for each main meta‐analysis to investigate whether sex differences were moderated by age (young, midlife, or older), physical activity level (sedentary, not sedentary), muscle group biopsied (arm/shoulder, back/trunk, leg), or analysis technique (mATPase, IHC, tissue homogenates). Forest plots were created for each main and subgroup analysis to display overall and individual study effect sizes, as well as overall effect sizes within subgroups using *forest* in the *metafor* package (Viechtbauer, 2010). These forest plots can be found in Supplemental File S5.

Heterogeneity was assessed using Cochran's *Q*‐test, and the magnitude of heterogeneity was estimated using the *I* ^2^ statistic (with 95% CIs; reflecting the proportion of between‐study variability due to true heterogeneity rather than chance). Heterogeneity according to the *I* ^2^ statistic was interpreted as: low heterogeneity (0%–29%); moderate heterogeneity (30%–49%); substantial heterogeneity (50%–74%); or considerable heterogeneity (>75%) (Jakobsen et al., 2014). Funnel plots were used to visualize publication bias and tested using Egger's regression test (Egger et al., 1997). In cases where Egger's test indicated significant asymmetry in funnel plots, Duval and Tweedie's trim‐and‐fill method (Duval & Tweedie, 2000) was used to determine the effect of publication bias on pooled effect sizes for each of the main meta‐analyses. Funnel plots can be found in Supplemental File S7.

## 3 RESULTS

**FIGURE 1:**
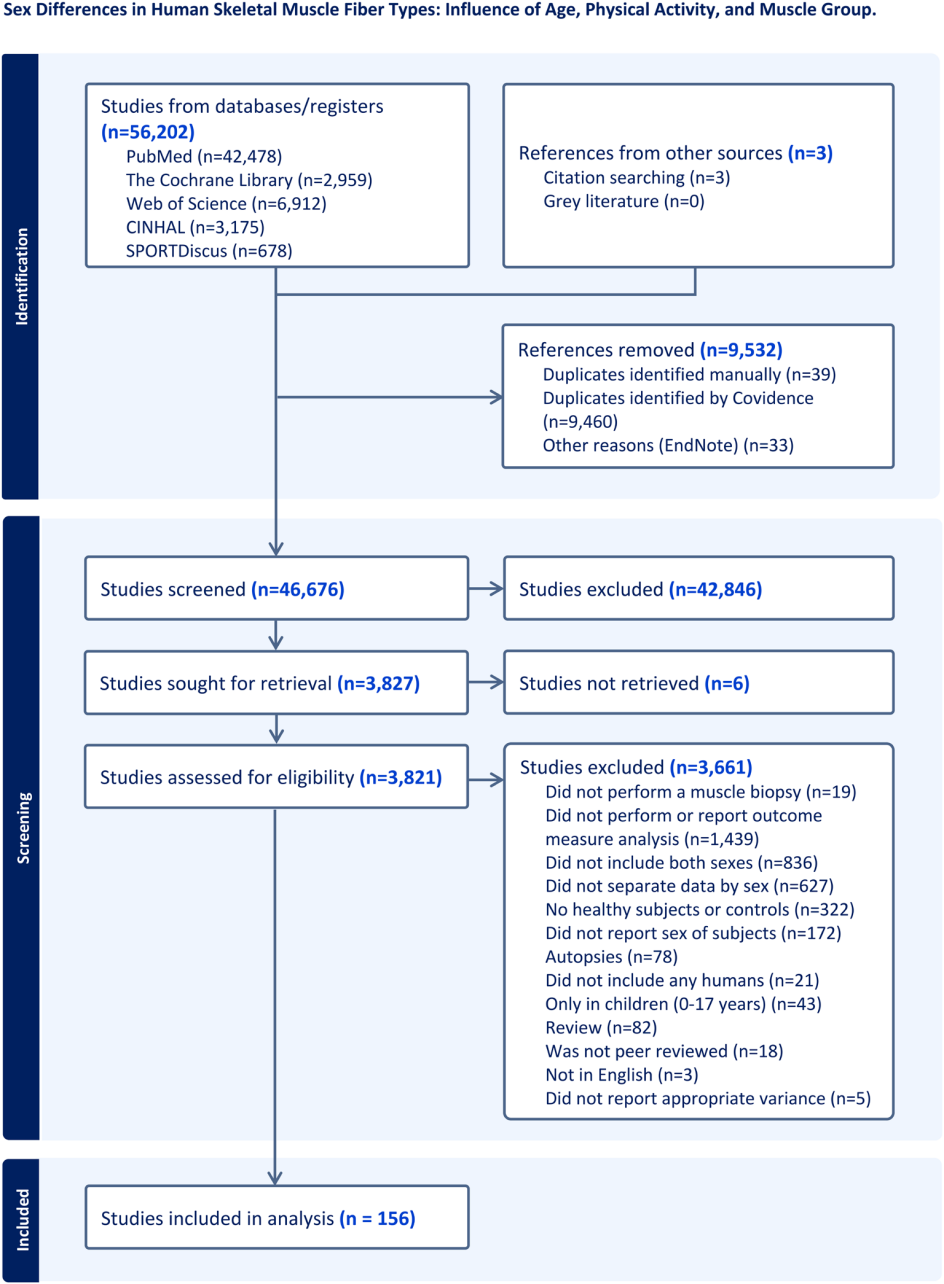
The PRISMA flow chart for the systematic review and meta‐analysis observing sex differences in fiber type composition. The diagram illustrates the number (*n*) of studies that were searched in the different phases of the systematic review. This diagram was created with permission from Covidence.

A PRISMA diagram displaying the synthesis of the analysis is presented in Figure 1.

### 3.1 Study characteristics

In total, 156 studies met the criteria for inclusion in the analysis (Abou Sawan et al., 2021; Ahmetov et al., 2009, 2011; Alway et al., 1992; Apple & Rogers, 1986a, 1986b; Ausin et al., 2017; Bailly et al., 2020; Bamman et al., 2003; Barnouin et al., 2017; Bell & Jacobs, 1989, 1990; Binet et al., 2023; Borges & Essen‐Gustavsson, 1989; Bouchard et al., 1986; Brose et al., 2003; Burke et al., 1977; Cairns et al., 2017; Calsbeek et al., 2002; Carter et al., 2001; Caswell et al., 2024; Churchward‐Venne et al., 2015; Coggan, Spina, King, Rogers, Brown, & Nemeth, 1992; Coggan, Spina, King, Rogers, Brown, Nemeth, & Holloszy, 1992; Costill et al., 1976; Dahlstrom et al., 1997; Dastmalchi et al., 2007; de Jong et al., 2023; den Hoed et al., 2009; Dial et al., 2021; Duscha et al., 2001; Edstrom & Nystrom, 1969; Engelbrecht et al., 2024; Engelhardt et al., 2022; Esbjornsson et al., 1993, 2012, 2021; Esbjornsson Liljedahl et al., 1996; Esbjornsson‐Liljedahl et al., 1999, 2002; Esbjornsson‐Liljedahl & Jansson, 1999; Essen‐Gustavsson & Borges, 1986; Fayet et al., 2001; Flueck et al., 2011; Froese & Houston, 1985; Fry et al., 1994; Galvan‐Alvarez et al., 2024; Galvan‐Alvarez, Gallego‐Selles, et al., 2023; Galvan‐Alvarez, Martin‐Rincon, et al., 2023; Gerard et al., 1987; Gerdle et al., 1997, 1998, 2000; Glenmark, 1994; Glenmark et al., 1992, 1994; Goedecke et al., 2000; Green et al., 2000, 2015; Guadalupe‐Grau et al., 2009, 2016; Guilherme, Semenova, Borisov, Kostryukova, et al., 2022; Guilherme, Semenova, Borisov, Larin, et al., 2022; Hakkinen et al., 2001, 2002; Hall, Lysenko, et al., 2021; Hall, Semenova, et al., 2021; He et al., 2001; Herda et al., 2019; Hoeg et al., 2009; Hoeg et al., 2013; Holmback et al., 2003; Horwath et al., 2021; Hostler et al., 2001; Jaworowski et al., 2002; Karlsen et al., 2019; Kim et al., 2005; Kosek et al., 2006; Kuipers et al., 1989; Kumagai et al., 2018; Larsson et al., 2006; Leenders, Verdijk, van der Hoeven, et al. 2013; Leenders, Verdijk, Van der Hoeven, Van Kranenburg, Nilwik, & Wodzig, 2013; Lexell et al., 1995; Liegnell et al., 2020; Lilja et al., 2023; Machek et al., 2020; Maher et al., 2009; Mannion, Dumas, et al., 1997; Mannion, Weber, et al., 1997; Marin et al., 1994; Martel et al., 2006; Maunder‐Sewry et al., 1980; Mazis et al., 2009; McDougall et al., 2023; McGuigan et al., 2001; McPhee et al., 2018; Messa et al., 2020; Miller et al., 1993, 2013; Moesgaard et al., 2022; Molsted et al., 2007; Montero et al., 2018; Moro et al., 2020; Norman et al., 2009, 2014; Nygaard et al., 1983; Oh et al., 2018; O'Hagan et al., 1995; Olmos et al., 2023; Owerkowicz et al., 2016; Paoli et al., 2013; Pollock et al., 2018; Porter et al., 2002; Ricoy et al., 1998; Ringqvist, 1974; Roberts et al., 2018; Roepstorff et al., 2006; Rolf et al., 1997; Ryushi et al., 1988; Sale et al., 1987; Schantz et al., 1983; Serrano et al., 2019; Shadiow et al., 2023; Sharman et al., 2001; Simoneau et al., 1985; Simoneau & Bouchard, 1989, 1995; Sjogaard, 1982; Sorensen et al., 2018; Stalberg et al., 1989; Staron et al., 1994, 2000; Steffensen et al., 2002; Sterczala et al., 2024; Sunnerhagen et al., 2000; Suominen et al., 1977; Suter et al., 1993; Takaragawa et al., 2021; Terzis et al., 2009; Thorstensson & Carlson, 1987; Toft et al., 2003; Torres et al., 2011; Trevino et al., 2019; Van Vossel et al., 2024; Varesco et al., 2022; Vescovo et al., 1996; Vikmoen et al., 2024; Walker et al., 2012; Wens et al., 2014; Whitman et al., 2005; Wiles et al., 1979; Williamson et al., 2001; Yasuda et al., 2005; Young, 1984; Yvert et al., 2020). The total number of participants from these studies was 7665, and 6222 were unique participants (males = 3501; females = 2721). The average study sample size was 44 participants (minimum: 4; maximum: 418). Individual study characteristics can be found in Supplemental File S4.

The publication year of the studies ranged from 1969 to 2024. Of the 156 studies, 113 were cross‐sectional observational designs (72.4%) and 43 incorporated the baseline data from interventions (27.6%). Most studies were conducted in Europe (*n* = 89; 57.1%), followed by North America (*n* = 57; 36.5%), then Asia (*n* = 4; 2.6%), Australia (*n* = 3; 1.9%), Africa (*n* = 2; 1.3%), and South America (*n* = 1; 0.6%).

Some studies included multiple groups of males and females, so they contributed multiple effects to the analysis, which comprised 220 participant groups. Of the 220 groups, 147 groups were categorized as young (66.8%) with an average age of ~25 years (males: 25.3 ± 4.2 years; females: 24.5 ± 4.0 years), 30 groups were categorized as midlife (13.6%) with an average age of ~51 years (males: 51.8 ± 9.2 years; females: 50.8 ± 8.3 years), and 43 groups were categorized as older (19.6%) with an average age of ~70 years (males: 70.3 ± 4.2 years; females: 69.5 ± 3.9 years). 57 groups were collected from intervention studies where only the baseline data was included (25.9%), while 163 groups were collected from observational or cross‐sectional design studies that included healthy individuals or had a healthy control group (74.1%). Of the 220 groups, 113 groups were categorized as not sedentary (51.4%), 34 groups were categorized as sedentary (15.4%), and 73 groups were unable to be determined (33.2%). Biopsies were extracted from the arm/shoulder in 12 groups (5.5%), the back/trunk in 8 groups (3.6%), and the leg in 199 groups (90.5%). One group extracted a biopsy from the temporal muscle (0.4%) and was therefore excluded from the muscle group biopsied subgroup analysis. 130 groups indicated the number of fibers that were analyzed. Of these, 33 groups (25%) did not analyze the recommended number of fibers (≥150) (Nederveen et al., 2020) (Table 1).

**TABLE 1:** Subject characteristics by subgroup analysis outcomes.

Category	Subcategory	*N*	% Analysis total
Age group	Young (≤35 years)	147	66.8
	Midlife (~35–65 years)	30	13.6
	Older (≥65 years)	43	19.6
Physical activity level	Not sedentary	113	51.4
	Sedentary	34	15.4
	Unable to be determined	73	33.2
Muscle group biopsied	Arm/Shoulder	12	5.5
	Back/Trunk	8	3.6
	Leg	199	90.5
	Head	1	0.4
Analysis technique	Myosin ATPase	142	64.5
	Immunohistochemistry	48	21.9
	Tissue homogenates	29	13.2
	Hematoxylin & eosin	1	0.4

*Note*: Data represent the number of participant groups out of the 220 total groups and the percentage they represent of the total analysis, across the four main subgroup analysis outcomes.

The arm/shoulder group included extraction of biopsies from the biceps brachii (*n* = 6; 2.7%), deltoid (*n* = 3; 1.4%), triceps brachii (*n* = 2; 0.9%), and extensor carpi radialis brevis (*n* = 1; 0.5%). The back/trunk group included the erector spinae (*n* = 3; 1.4%), lumbar multifidus (*n* = 2; 0.9%), lumbar longissimus (*n* = 1; 0.5%), latissimus dorsi (*n* = 1; 0.5%), and trapezius (*n* = 1; 0.5%). The leg group included the vastus lateralis (*n* = 180; 81.8% of the 220 groups), gastrocnemius (*n* = 14; 6.4%), tibialis anterior (*n* = 3; 1.4%), soleus (*n* = 1; 0.5%), and rectus femoris (*n* = 1; 0.5%). Regarding analysis techniques to identify the fibers, 142 groups performed mATPase (64.5%), 48 groups performed IHC (21.9%), and 29 groups performed muscle tissue homogenization (13.2%). Only one group performed hematoxylin and eosin (0.4%) and was therefore excluded from the analysis technique subgroup analysis (Table 1).

### 3.2 Meta‐analyses

#### 3.2.1 Primary meta‐analyses

**FIGURE 2:**
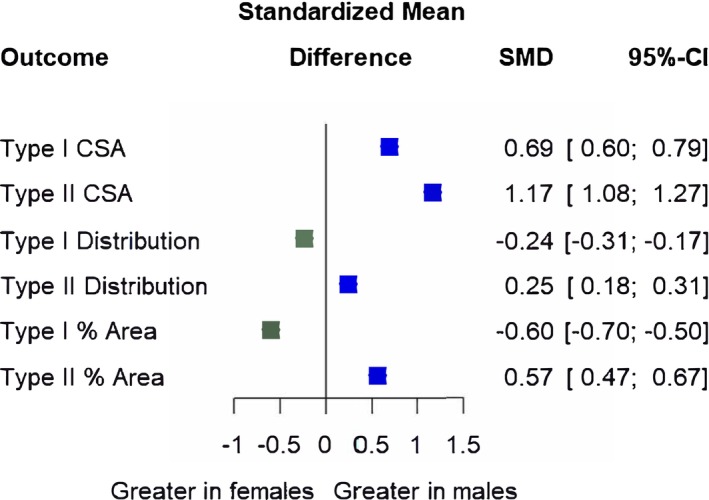
Sex differences in fiber type cross‐sectional area (CSA), fiber type distribution (% number) and proportional area (% of sample area). Forest plot displaying standardized mean differences (SMD) and 95% confidence intervals (CIs). Blue squares and positive values indicate outcomes greater in males, whereas green squares and negative values indicate outcomes greater in females. CSA, cross‐sectional area; Distribution, fiber type distribution; % Area, proportional area. Size of symbols in this figure are not representative of weight (which is determined by sample size and CIs) because displayed are multiple outcome variables.

Sex differences were observed across all outcomes. Males had greater type I CSA (4936 ± 1250 μm^2^) than females (4151 ± 1074 μm^2^; SMD = 0.69; 17.3% difference), greater type II CSA (5272 ± 1950 μm^2^) than females (3483 ± 1309 μm^2^; SMD = 1.17; 40.9% difference), greater type II fiber type distribution (51.6 ± 14.6%) than females (48.3 ± 13.0%; SMD = 0.25; 6.6% difference), and greater type II proportional area (55.0 ± 14.4%) than females (47.9 ± 13.1%; SMD = 0.57; 13.7% difference). Conversely, females had a greater type I fiber type distribution (51.4 ± 12.1%) than males (48.3 ± 13.3%; SMD = ‐0.24; 6.1% difference), and greater type I proportional area (51.8 ± 12.4%) than males (44.9 ± 13.2%; SMD = ‐0.60; 14.3% difference). SMDs and their 95% CIs are displayed in Figure 2, and full model outputs are displayed in Tables 2, 3, 4.

**TABLE 2:** Sex differences in fiber type cross‐sectional area as determined by random‐effects meta‐analyses.

Cross‐sectional area (mm^2^)		*N*		Type I								Type II							
		M	F	M	F	SM*D*	*p*	95% CI	*I* ^2^ (%)	Tau^2^	*Q*	M	F	SM*D*	*p*	95% CI	*I* ^2^ (%)	Ta*u* ^2^	*Q*
Total		2223	1772	4936 ± 1250	4151 ± 1073	0.69	<0.001	0.61, 0.79	30.9	0.08	203.94	5272 ± 1949	3483 ± 1309	1.17	<0.001	1.08, 1.27	30.4	0.099	202.71
Age	Young	1548	1247	4810 ± 1228	4092 ± 982	0.63	0.14	0.52, 0.73	23.5	0.04	118.98	5579 ± 1830	3797 ± 1307	1.07	<0.001	0.98, 1.15	0	0	87.11
	Midlife	298	242	5016 ± 1308	4222 ± 1156	0.72		0.41, 1.02	58.5	0.19	33.77	5068 ± 2812	3221 ± 1489	1.28		0.74, 1.83	80.7	0.77	72.72
	Older	533	378	5175 ± 1239	4258 ± 1099	0.84		0.65, 1.03	28.7	0.03	47.79	4671 ± 1441	2737 ± 1012	1.39		1.24, 1.55	0	0	32.28
Physical activity level	Sedentary	194	174	4672 ± 1202	3907 ± 944	0.75	0.68	0.42, 1.08	40.1	0.12	25.05	4581 ± 1695	3202 ± 1239	1.03	0.42	0.75, 1.31	21.6	0.06	19.13
	Not Sedentary	1393	1023	4954 ± 1262	4134 ± 1004	0.68		0.54, 0.82	38.2	0.09	114.96	5602 ± 2036	3747 ± 1263	1.15		1.03, 1.27	22.1	0.03	91.17
Muscle group biopsied	Arm/Shoulder	71	58	4667 ± 1160	3404 ± 982	1.02	0.08	0.45, 1.59	29	0.1	11.27	6897 ± 2300	3852 ± 1026	1.69	0.12	1.06, 2.32	28.6	1.18	11.2
	Back/Trunk	42	29	5876 ± 1207	4183 ± 853	1.47		−0.51, 3.46	51.2	0.26	4.1	5448 ± 2636	2708 ± 1050	1.3		0.41, 2.19	0	9	1.17
	Leg	2266	1780	4907 ± 1244	4166 ± 1035	0.66		0.57, 0.75	29.2	0.07	182.1	5259 ± 1876	3509 ± 1290	1.15		1.05, 1.24	31.2	0.1	187.48
Analysis technique	mATPase	1683	1320	4827 ± 1236	4017 ± 960	0.78	0.002	0.68, 0.89	27.4	0.07	141.96	5391 ± 2053	3545 ± 1295	1.17	0.96	1.06, 1.28	25.6	0.09	138.43
	IHC	696	547	5136 ± 1253	4446 ± 1184	0.5		0.34, 0.65	33.4	0.06	55.59	5119 ± 1495	3417 ± 1238	1.17		0.98, 1.35	45.2	0.12	67.47

*Note*: Data represent the main analyses outcomes and subgroup analyses outcomes. Data are weighted means ± standard deviation.

Abbreviations: CI, confidence interval; F, female; IHC, immunohistochemistry; M, male; mATPase, myofibrillar adenosine triphosphatase; *N*: number; SMD, standardized mean difference.

**TABLE 3:** Sex differences in fiber type distribution as determined by random‐effects meta‐analyses.

Fiber type distribution (%)		*N*		Type I								Type II							
		M	F	M	F	SMD	*p*	95% CI	*I* ^2^ (%)	Tau^2^	*Q*	M	F	SMD	*p*	95% CI	*I* ^2^ (%)	Tau^2^	*Q*
Total		2716	2031	48.3 ± 13.3	51.4 ± 12.1	−0.24	0.01	−0.31, −0.17	20.6	0.02	213.99	51.6 ± 14.6	48.3 ± 13.0	0.25	<0.001	0.18, 0.31	10.7	<0.001	190.46
Age	Young	2176	1620	48.1 ± 12.5	52.6 ± 11.3	−0.32	0.002	−0.41, −0.24	28	0.02	159.81	52.0 ± 13.3	47.3 ± 12.1	0.32	<0.001	0.24, 0.40	23.8	0.006	150.82
	Midlife	328	267	45.6 ± 14.9	44.9 ± 11.6	−0.02		−0.21, −0.17	6.5	0	20.32	54.3 ± 17.9	54.4 ± 13.7	0.08		−0.07, 0.22	0	0	11.66
	Older	443	321	51.6 ± 13.2	51.8 ± 12.8	−0.11		−0.25, 0.02	0	0.004	25.72	47.8 ± 14.9	47.7 ± 13.4	0.1		−0.03, 0.22	0	0	20.61
Physical activity level	Sedentary	276	248	46.0 ± 12.9	48.2 ± 11.0	−0.2	0.22	−0.38, −0.01	0	0.006	22.76	53.9 ± 14.3	51.5 ± 12.8	0.19	0.15	0.03, 0.34	0	0	16.21
	Not sedentary	1920	1317	49.7 ± 12.5	54.3 ± 11.5	−0.32		−0.40, −0.23	15.1	0.003	106.04	50.4 ± 13.1	45.7 ± 12.1	0.31		0.23, 0.39	13	<0.001	103.44
Muscle group biopsied	Arm/Shoulder	90	75	46.9 ± 11.2	47.4 ± 10.0	−0.07	0.29	−0.32, 0.18	0	0	4.31	51.8 ± 13.0	51.3 ± 11.8	0.05	0.11	−0.18, 0.27	0	0	3.52
	Back/Trunk	81	60	60.9 ± 9.8	63.7 ± 10.0	−0.27		−0.75, 0.21	19.6	0.016	7.47	37.9 ± 11.1	35.3 ± 10.4	0.21		−0.16, 0.58	0	0	4.53
	Leg	2768	2068	48.1 ± 13.1	51.4 ± 11.7	−0.25		−0.33, −0.18	26.3	0.029	206.29	52.0 ± 14.3	48.4 ± 12.6	0.27		0.20, 0.36	17.7	0.01	184.61
Analysis technique	mATPase	2107	1572	49.6 ± 12.6	53.0 ± 11.2	−0.22	0.28	−0.31, −0.12	29.5	0.04	177.2	50.0 ± 13.8	46.5 ± 12.3	0.24	0.45	0.16, 0.32	16	0.01	148.9
	IHC	831	628	45.1 ± 13.7	47.8 ± 12.4	−0.3		−0.41, −0.18	1.9	0.01	43.83	55.9 ± 14.9	52.6 ± 13.0	0.29		0.17, 0.41	7.9	0.01	46.7

*Note*: Data represent the main analyses outcomes and subgroup analyses outcomes. Data are weighted means ± standard deviation.

Abbreviations: CI, confidence interval; F, female; IHC, immunohistochemistry; M, male; mATPase, myofibrillar adenosine triphosphatase; *N*, number; SMD, standardized mean difference.

**TABLE 4:** Sex differences in proportional area as determined by random‐effects meta‐analyses.

Proportional area (%)		*N*		Type I								Type II							
		M	F	M	F	SMD	*p*	95% CI	*I* ^2^ (%)	Tau^2^	*Q*	M	F	SMD	*p*	95% CI	*I* ^2^ (%)	Tau^2^	*Q*
Total		1620	1206	44.9 ± 13.2	51.8 ± 12.4	−0.6	<0.001	−0.70, −0.50	26.1	0.03	148.94	55.0 ± 14.4	47.9 ± 13.1	0.57	<0.001	0.47, 0.67	23.7	0.03	142
Age	Young	1077	817	44.5 ± 13.0	51.6 ± 11.5	−0.63	0.2	−0.77, −0.49	36.1	0.09	123.55	55.5 ± 12.9	48.2 ± 11.8	0.59	0.54	0.46, 0.73	32.8	0.08	116.1
	Midlife	265	236	40.7 ± 12.3	48.5 ± 13.8	−0.66		−0.81, −0.50	0	<0.001	6.76	59.2 ± 15.4	51.3 ± 14.1	0.61		0.45, 0.77	0	0	6.84
	Older	283	186	50.3 ± 14.7	55.2 ± 14.2	−0.45		−0.65, −0.24	0	0	15.9	49.3 ± 15.0	44.1 ± 13.7	0.47		0.25, 0.70	9.5	<0.001	18.79
Physical activity level	Sedentary	298	289	40.7 ± 12.3	50.0 ± 12.4	−0.75	0.15	−0.96, −0.54	21.2	<0.001	22.85	59.2 ± 12.9	49.9 ± 13.4	0.67	0.38	0.46, 0.88	12.8	<0.001	19.5
	Not Sedentary	868	576	47.6 ± 13.3	54.5 ± 11.7	−0.58		−0.71, −0.45	19.6	<0.001	69.65	52.1 ± 13.4	45.2 ± 11.5	0.57		0.44, 0.69	13.2	<0.001	64.48
Muscle group biopsied	Arm/Shoulder	51	45	39.0 ± 9.7	43.6 ± 13.1	−0.36	0.001	−0.75, 0.04	0	0	2.65	60.5 ± 13.1	56.0 ± 13.9	0.32	<0.01	−0.02, 0.66	0	0	1.99
	Back/Trunk	71	54	58.8 ± 9.4	67.8 ± 7.3	−1.13		−1.55, −0.72	0	0	3.32	41.2 ± 9.7	31.9 ± 8.0	1.07		0.63, 1.50	0	0	3.74
	Leg	1503	1140	44.4 ± 13.4	51.1 ± 12.6	−0.58		−0.68, −0.48	27	0.03	134.3	55.5 ± 13.9	48.7 ± 12.7	0.55		0.45, 0.66	25	0.02	129.3
Analysis technique	mATPase	674	486	50.5 ± 13.2	56.2 ± 11.7	−0.57	0.81	−0.74, −0.40	31.9	0.1	76.3	49.5 ± 12.9	43.7 ± 11.6	0.53	0.66	0.37, 0.69	25.8	0.08	68.74
	IHC	383	298	40.9 ± 14.8	48.3 ± 14.6	−0.62		−0.78, −0.46	0	<0.001	15.8	58.6 ± 16.4	51.5 ± 14.7	0.57		0.42, 0.72	0	0	14.21
	Homog‐enates	568	455	41.0 ± 12.0	48.8 ± 11.5	−0.64		−0.81, −0.48	31.5	0.003	55.51	59.2 ± 12.7	50.8 ± 12.0	0.64		0.46, 0.81	34.3	0.01	57.87

*Note*: Data represent the main analyses outcomes and subgroup analyses outcomes. Data are weighted means ± standard deviation.

Abbreviations: CI, confidence interval; F, female; IHC, immunohistochemistry; M, male; mATPase, myofibrillar adenosine triphosphatase; *N*, number; SMD, standardized mean difference.

#### 3.2.2 Sex differences by age

**FIGURE 3:**
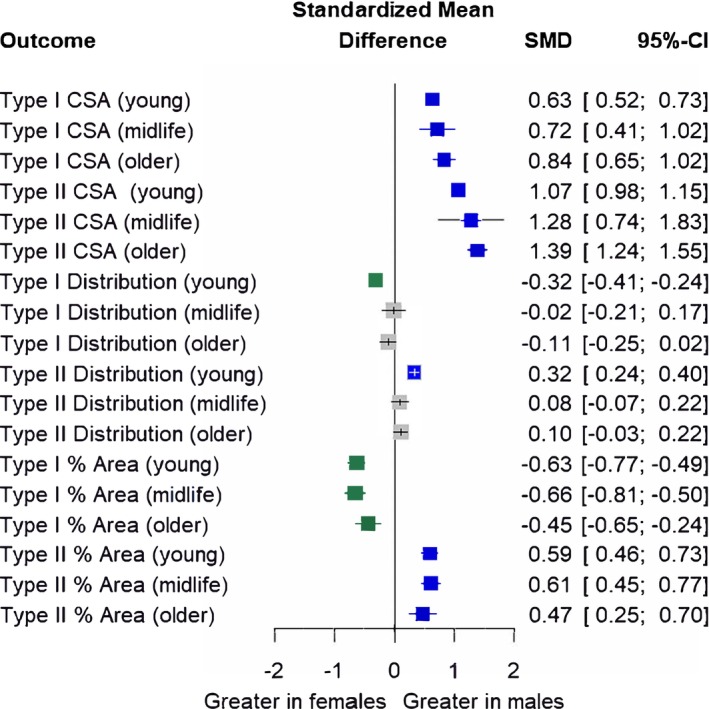
Subgroup analysis investigating sex differences by age group (young, midlife, and older). Forest plot displaying standardized mean differences (SMD) and 95% confidence intervals (CIs). Blue squares and positive values indicate outcomes greater in males, whereas green squares and negative values indicate outcomes greater in females. Gray boxes indicate outcomes where no sex differences were observed. CSA, cross‐sectional area; Distribution, fiber type distribution; % Area, proportional area. Size of symbols in this figure are not representative of weight (which is determined by sample size and CIs) because displayed are multiple outcome variables.

Subgroup analyses for age (Figure 3) showed sex differences remained for type I CSA (*p* = 0.14), type I proportional area (*p* = 0.20) and type II proportional area (*p* = 0.54). For type II CSA outcomes, subgroup analyses by age (*p* = 0.0008) revealed that the magnitude of the sex difference was the greatest in the older group (SMD = 1.39) and slightly decreased in the midlife (SMD = 1.28) and young groups (SMD = 1.07), although it remained significant. Conversely, for type I and type II fiber type distribution, sex differences were present in the young group (type I SMD = −0.32; type II SMD = 0.32) but were not present in the midlife (type I SMD = −0.02; type II SMD = 0.08) or older age groups (type I SMD = −0.11; type II SMD = 0.10).

#### 3.2.3 Sex differences by physical activity level

**FIGURE 4:**
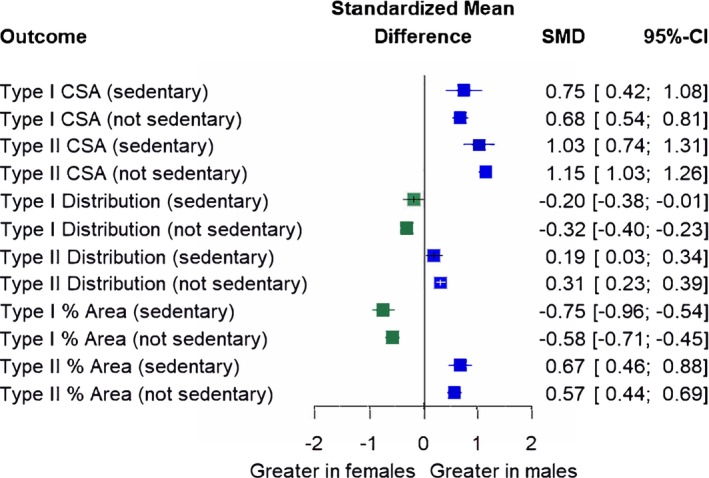
Subgroup analysis investigating sex differences by physical activity level (sedentary and not sedentary). Forest plot displaying standardized mean differences (SMD) and 95% confidence intervals (CIs). Blue squares and positive values indicate outcomes greater in males, whereas green squares and negative values indicate outcomes greater in females. CSA, cross‐sectional area; Distribution, fiber type distribution; % Area, proportional area. Size of symbols in this figure are not representative of weight (which is determined by sample size and CIs) because displayed are multiple outcome variables.

Across all outcomes, the sex differences in fiber type characteristics were not modified by physical activity level (Figure 4). Specifically, sex differences remained for both sedentary and not sedentary subgroups for type I CSA (*p* = 0.68), type II CSA (*p* = 0.42), type I fiber type distribution (*p* = 0.22), type II fiber type distribution (*p* = 0.15), type I proportional area (*p* = 0.15), and type II proportional area (*p* = 0.38).

#### 3.2.4 Sex differences by muscle group biopsied

**FIGURE 5:**
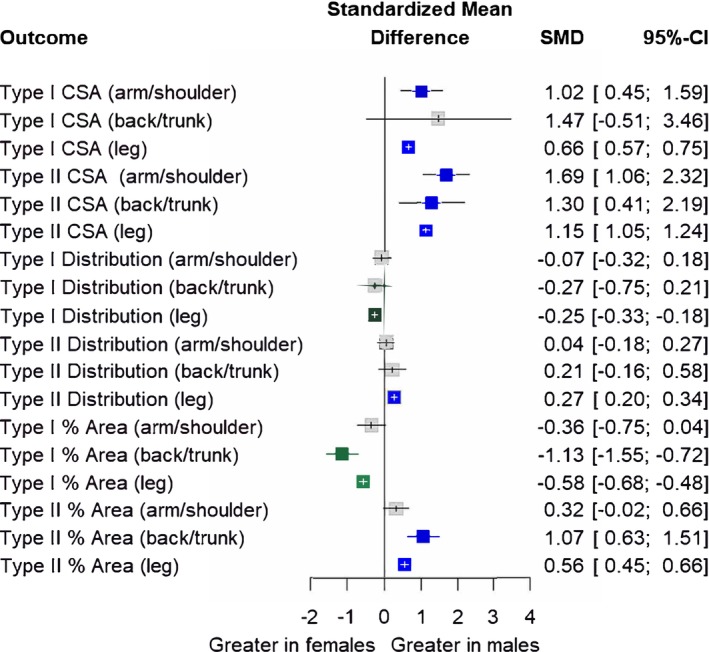
Subgroup analysis investigating sex differences by the muscle group biopsied (arm/shoulder, back/trunk, and leg). Forest plot displaying standardized mean differences (SMD) and 95% confidence intervals (CIs). Blue squares and positive values indicate outcomes greater in males, whereas green squares and negative values indicate outcomes greater in females. Gray boxes indicate outcomes where no sex differences were observed. CSA, cross‐sectional area; Distribution, fiber type distribution; % Area, proportional area. Size of symbols in this figure are not representative of weight (which is determined by sample size and CIs) because displayed are multiple outcome variables.

When analyzed by the muscle group biopsied as subgroups (Figure 5), sex differences remained for type I CSA (*p* = 0.08), type II CSA (*p* = 0.12), type I fiber type distribution (*p* = 0.29), and type II fiber type distribution (*p* = 0.11). For type I proportional area (*p* = 0.001) and type II proportional area (*p* = 0.002), subgroup analyses by muscle group biopsied revealed that the sex differences remained in the back/trunk (type I SMD = −1.13; type II SMD = 1.07) and leg (type I SMD = −0.58; type II SMD = 0.56) but no significant sex differences were observed in the arm/shoulder muscle group (type I SMD = −0.36; type II SMD = 0.32).

#### 3.2.5 Sex differences by analysis technique

**FIGURE 6:**
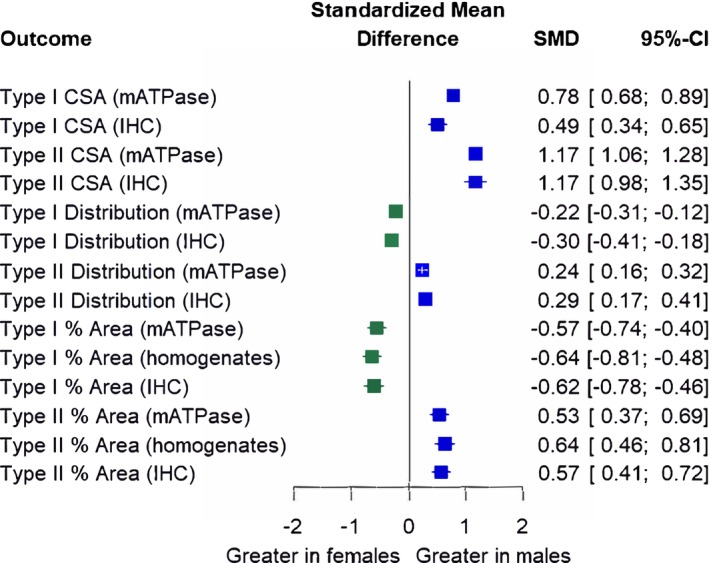
Subgroup analysis investigating sex differences by analysis technique (mATPase, IHC, and homogenates). Forest plot displaying standardized mean differences (SMD) and 95% confidence intervals (CIs). Blue squares and positive values indicate outcomes greater in males, whereas green squares and negative values indicate outcomes greater in females. CSA, cross‐sectional area; Distribution, fiber type distribution; % Area, proportional area; mATPase, myofibrillar adenosine triphosphatase; IHC: Immunohistochemistry; homogenates: muscle tissue homogenates. Size of symbols in this figure are not representative of weight (which is determined by sample size and CIs) because displayed are multiple outcome variables.

For type II CSA (*p* = 0.96), type I fiber type distribution (*p* = 0.28), type II fiber type distribution (*p* = 0.45), type I proportional area (*p* = 0.81), and type II proportional area (*p* = 0.66), sex differences did not differ significantly by analysis technique (Figure 6). Conversely, analysis technique significantly moderated sex differences in type I CSA (*p* = 0.002), whereby the magnitude of sex difference was greater when analyzed by mATPase (SMD = 0.78) compared to IHC (SMD = 0.49), although it remained significant.

### 3.3 Risk of bias and heterogeneity

Using the Newcastle Ottawa scale to assess risk of bias, of the 220 groups, 0 groups were categorized as very good, 24 groups were categorized as good (10.9%), 174 groups were categorized as satisfactory (79.1%), and 22 groups were categorized as unsatisfactory (10%). Of the 220 groups, 3% (*n* = 7) described participant recruitment and had a representative sample, 4% (*n* = 8) justified sample size, 93% (*n* = 204) biopsied ≥95% of all participants included in the study, 100% performed validated analysis techniques, 35% (*n* = 78) controlled for sex with subgroup or multivariate analysis, 21% (*n* = 47) had blinded researchers perform the analysis, and 91% (*n* = 201) described their statistics around their biopsy data. Risk of bias categories for each individual study are displayed in Supplemental File S4.

In the main meta‐analyses described previously, *I* ^2^ and *Q*‐test statistics detected: moderate and statistically significant heterogeneity for studies investigating type I CSA (*I* ^2^ = 30.9%, 95% CI: 14.6%, 44.0%; *Q* = 203.94, *p* < 0.001), and type II CSA (*I* ^2^ = 30.4%, 95% CI: 14.1%, 43.7%; *Q* = 202.71; *p* < 0.001); low and statistically significant heterogeneity for studies investigating type I fiber type distribution (*I* ^2^ = 20.6%; 95% CI: 3.1%, 34.8%; *Q* = 213.99, *p* = 0.013), type I proportional area (*I* ^2^ = 26.1%; 95% CI: 6.0%, 42.0%; *Q* = 148.94, *p* = 0.008), and type II proportional area (*I* ^2^ = 23.7%; 95% CI: 2.6%, 40.2%; *Q* = 142.80, *p* = 0.017); and low and non‐significant heterogeneity for type II fiber type distribution (*I* ^2^ = 10.7%; 95% CI: 0.0%, 27.0%; *Q* = 190.46, *p* = 0.135).

Funnel plots for each of the main meta‐analyses are displayed in the supplementary material. Statistical testing of funnel plot asymmetry using Egger's regression test suggested no publication bias for type I CSA (*t* = 0.79, *p* = 0.431), type I proportional area (*t* = −0.65, *p* = 0.516), or type II proportional area (*t* = 1.11; *p* = 0.269), while significant funnel plot asymmetry was detected for type II CSA (*t* = 2.23, *p* = 0.027), type I fiber type distribution (*t* = 2.93, *p* = 0.0039) and type II fiber type distribution (*t* = −2.77, *p* = 0.006). Trim‐and‐fill procedures suggested that original meta‐analysis models may have overestimated sex differences due to publication bias for type II CSA (*n* = 23 studies added; original SMD = 1.17; re‐estimated SMD = 1.03), and underestimated sex differences for type I fiber type distribution (*n* = 29 studies added; original SMD = −0.24; re‐estimated SMD = −0.32) and type II fiber type distribution (*n* = 27 studies added; original SMD = 0.25; re‐estimated SMD = 0.32).

## 4 DISCUSSION

This systematic review and meta‐analysis identified clear and robust sex differences in muscle fiber CSA, fiber type distribution, and proportional area in healthy populations across the lifespan regardless of physical activity levels, and in general remained across different ages, muscle groups biopsied, and techniques used for analysis. This systematic review included 156 studies comprising over 6200 unique participants and was conducted according to PRISMA guidelines. The major findings are fourfold. First, males have a larger type I CSA, type II CSA, type II fiber type distribution, and type II proportional area than females. Second, females have a greater type I fiber type distribution and type I proportional area compared with males. Third, sex differences in CSA, fiber type distribution, and proportional area remained regardless of physical activity level (sedentary vs. not sedentary). Fourth, age, muscle group biopsied, and analysis technique impact the strength of some identified sex differences. Collectively, these findings suggest there are inherent biological sex differences in skeletal muscle composition that remain regardless of physical activity levels and across most age groups, muscle groups, and analysis techniques. These findings may be useful in (1) understanding sex differences in neuromuscular physiology, (2) developing sex‐specific exercise prescription and rehabilitation protocols, and (3) determining factors that drive sex‐specific differences in disease pathology and sport performance.

### 4.1 Sex differences are present across skeletal muscle fiber type characteristics

Our findings demonstrate fundamental robust differences in the fiber characteristics of male and female skeletal muscle. The basic unit of skeletal muscle in humans, the muscle fiber, is comprised of myosin heavy chain (MHC) isoforms (I, IIa, IIx) and hybrids (I/IIa, IIa/IIx, I/IIa/IIx) that are differentiated by their contractile properties and metabolic profiles. These fiber types differ in size, capillary and mitochondrial content, and glycolytic and oxidative capacity (Schiaffino & Reggiani, 2011), consequently playing important roles in metabolism, the ability to perform activities of daily living, and sport performance. Although there is a continuum, when categorized, type I fibers have slower contractile kinetics, greater oxidative capacity, and are more fatigue resistant, while type IIa and type IIx produce more force and power as they have faster contractile kinetics (up to twice the maximal shortening velocity of type I fibers) (Krivickas et al., 2001; Sundberg, Hunter, et al., 2018; Trappe et al., 2003). The classification of fiber types has changed as analysis techniques have evolved in more recent years. Fiber types were traditionally determined based on their enzymatic properties as determined via mATPase. More recently, fiber types have been determined by their MHC isoform content as identified via IHC and SDS‐PAGE (Plotkin et al., 2021). For ease of interpretation of results, we chose a convenient classification which combined all slow twitch fiber classifications–reported as type I–and all fast twitch fiber classifications–reported as type II. Importantly, although previous research has found strong correlations between mATPase, IHC, and SDS‐PAGE (Chalmers & Row, 2011; Damer et al., 2022; Fry et al., 1994; Scott et al., 2001), a subgroup analysis was performed to determine if the analysis technique influenced observed sex differences.

Males and females were found to differ in type I CSA, distribution, and proportional area. Male type I CSA was 17.3% larger than that of females. Additionally, type I fiber type distribution of females was 6.1% (absolute: 3.1%) higher than in males. Lastly, female type I proportional area was 14.3% (absolute: 6.9%) higher than male type I proportional area. Because fiber types differ in their morphological, metabolic, and contractile properties, determining the proportional area of each fiber type, which can be used as a proxy to estimate the percent area each fiber occupies in a whole muscle, provides insight into the overall function of a muscle. For example, contractile speed, force, and power of skeletal muscle are dependent on the overall muscle fiber type proportional area. Specifically, larger type I proportional area in females would lead to females having lower muscle power production than males when matched for absolute amounts of muscle mass. Importantly, our observed sex difference in type I proportional area is consistent with slower contracting whole muscle of females compared with males when contracting at the same relative intensity (Hunter, 2024; Hunter et al., 2006, 2023; Wust et al., 2008). Finally, it is important to note that sex differences in type I CSA, fiber type distribution, and proportional area remained across all analysis techniques (mATPase, IHC, homogenates) as determined by subgroup analyses. Of note, the subgroup analysis revealed that the sex difference in type I CSA was smaller when analyzed by IHC versus mATPase (14.4% vs. 18.3%, respectively). Even though there are nuances between analysis techniques in type I CSA measures, significant sex differences in type I fiber type composition are present, regardless of analysis technique, emphasizing the presence of extremely robust biological sex differences.

Likewise, males and females were found to have robust differences in type II fiber type CSA, distribution, and proportional area. First, male type II fiber CSA was 40.9% larger than female type II fiber CSA, which is twice the relative magnitude of the sex difference we observed for type I fiber CSA. Second, male type II fiber type distribution was 6.6% (absolute: 3.3%) higher than female type II fiber type distribution. While many previous reviews have noted that there is not conclusive evidence of a sex difference in fiber type distribution (Hunter, 2016a; Hunter et al., 2023), a recent meta‐analysis identified a significant sex difference (Nuzzo, 2024). Our findings confirm the sex difference in fiber type distribution with the increased power of performing the systematic review according to PRISMA guidelines. Third, male type II proportional area was 13.7% (absolute: 7.1%) higher than female type II proportional area. These findings are consistent with previous reviews that also reported significant sex differences in proportional area (Hunter, 2016a; Hunter et al., 2023; Nuzzo, 2023; Nuzzo, 2024). Lastly, sex differences in type II CSA, fiber type distribution, and proportional area remained across all analysis techniques (mATPase, IHC, homogenates) as determined by subgroup analyses. Taken together, our findings suggest that there are biological sex differences in CSA, fiber type distribution, and proportional area. Our observations are the first to confirm that these observed sex differences in skeletal muscle composition are robust and present for all fiber type analysis techniques.

### 4.2 Sex differences in skeletal muscle fiber type characteristics generally remain with age

Males and females were found to have significant differences in type I and type II CSA and proportional area across all age groups (young, midlife, older). Males had significantly larger type II CSA at each age group, but the sex difference increased with age. It may be speculated that type II CSA decreases in females to a greater degree than in males throughout the lifespan (males: 17.7% decrease; females: 32.4% decrease) (Table 2). This has been observed previously in an experimental study performed on 220 adults, with it being noted that there may be potential sex‐based differences in mechanisms regarding atrophy with aging–greater muscle fiber in males and greater fiber atrophy in females (Roberts et al., 2018). Additionally, our subgroup analyses revealed that the sex differences in fiber type distribution were modified by age. Specifically, females had greater type I fiber type distribution than males in the young group but not in the midlife or older groups (relative sex difference: young: 8.9%, midlife: 1.6%, older: 0.4%; absolute sex difference: young: 4.5%, midlife: 0.7%, older: 0.2%). Similarly, males had greater type II fiber type distribution than females in the young group but not in the midlife or older groups (relative sex difference: young: 9.5%, midlife: 0.2%, older: 0.2%; absolute sex difference: young: 4.7%, midlife: 0.1%, older: 0.1%). These results are similar to Nuzzo (type I fiber type distribution: females > males in young/middle age, females = males in older age; type IIA/IIX fiber type distribution: males > females in young/middle age, males = females in older age) (Nuzzo, 2024). However, Nuzzo only reported two adult age groups (18–59 and >59) and did not specifically identify young vs. midlife groups, whereas we identified three adult age groups (18− ≤35, ~35–65, ≥65). We cannot conclude whether this absence of a sex difference in the midlife and older age groups is an effect of aging or due to low sample sizes in these two age groups, as the SMDs were small (−0.24 and 0.25). This remains an area of opportunity for further studies.

Importantly, while there were no sex differences observed for fiber type distribution in the midlife or older groups, sex differences in proportional area remained present in all age groups. This finding suggests that the sex differences in proportional area are driven by large sex differences in CSA, as proportional area is calculated by combining the variables CSA and fiber type distribution. Specifically, the sex differences in CSA at each age group are so substantial that the presence, or even lack of sex differences in fiber type distribution does not seem to impact proportional area. Proportional area likely has the most relevance to the contractile properties of the muscle, and consequently, physical function (Haizlip et al., 2015; Hunter, 2016b; Wust et al., 2008). Our findings suggest that biological sex differences in proportional area remain across the lifespan, and this is driven by the presence of sex differences in CSA in all age groups.

### 4.3 Sex differences in skeletal muscle fiber type characteristics remain regardless of physical activity level

Sex differences in CSA, fiber type distribution, and proportional area remained across all physical activity levels. Males had larger type I and type II CSA, type II fiber type distribution, and type II proportional area compared to females, while females had greater type I fiber type distribution and type I proportional area compared to males in each activity level group (sedentary, not sedentary). These findings suggest that biological sex differences in CSA, fiber type distribution, and proportional area are very robust. Despite the powerful effects of physical activity and training on fiber type characteristics (Plotkin et al., 2021; Staron et al., 1994), physical activity levels did not influence the sex differences in these fiber type characteristics.

We were not able to perform statistical analyses on the influence that physical activity levels have on CSA, fiber type distribution, and proportional area when combining males and females into one sedentary and one not sedentary group, as this was outside of the scope of our review. However, Tables 2, 3, 4 suggest that non‐sedentary participants had larger CSA of type I and type II fibers, greater type I fiber type distribution, and greater type I proportional area in both males and females. Further research needs to be performed to confirm these findings. Importantly, although these adaptations occurred, the sex difference percentage remained relatively constant, suggesting that physical activity may not diminish the identified biological sex differences. Our data are consistent with previous research that reports similar sex differences with short‐term training (e.g., 3 months) regarding relative adaptations in hypertrophy (Roberts et al., 2020) and cardiorespiratory function ($\dot{V}$ O_2_max) (Hunter et al., 2023), although some data suggests males may have greater cardiac adaptations with long‐term training (e.g., 1 year) (Howden et al., 2015). However, it remains unknown whether specific modes of physical activity (e.g., aerobic vs. endurance) or intensity and/or length of training (e.g., recreationally active vs. Olympic athlete, a few months vs. many years) can modify the sex difference percentage.

### 4.4 Sex differences in skeletal muscle fiber type characteristics generally remain regardless of the muscle group biopsied

Our subgroup analysis revealed that sex differences in CSA and fiber type distribution remained across all muscle groups biopsied (arm/shoulder, back/trunk, leg). However, our subgroup analysis revealed that females had a greater type I proportional area than males in the back/trunk and leg muscle groups, but not the arm/shoulder muscle group (relative sex difference: back/trunk: 17.5%, leg: 15.4%, arm/shoulder: 10.5%; absolute sex difference: back/trunk: 10.9%, leg: 7.4%, arm/shoulder: 4.3%). Similarly, males had a greater type II proportional area than females in the leg and back/trunk muscle groups, but not the arm/shoulder muscle group (relative sex difference: back/trunk: 29.8%, leg: 14.5%, arm/shoulder: 7.2%; absolute sex difference: back/trunk: 11.2%, leg: 7.5%, arm/shoulder: 4.2%). The sex differences observed in the back/trunk and leg muscle groups may be useful information for clinicians as these identified differences may play a role in specific injury risk. For example, bone fractures are more likely to occur in females, which have primarily been attributed to a higher prevalence of osteoporosis (Prevention CfDCa, 2021). However, it may be speculated that the greater type I proportional area in the back/trunk and leg muscle groups in females may put females at greater fall risk due to the slower contractile properties of type I fibers, potentially decreasing the ability to react quickly to perturbations – a main cause of falls. It is important to note that the sample sizes in the back/trunk and arm/shoulder muscle groups are small. Therefore, further research may be needed to confirm these findings and conclusions regarding muscle groups outside the lower body interpreted with caution.

### 4.5 Strengths and limitations

This study has several key strengths. First, this is the first review on this topic which conforms to PRISMA guidelines, which we performed in order to ensure rigor and replicability, enhance transparency, and promote confidence in the findings of a recent publication (Nuzzo, 2024), which reported preliminary sex differences in CSA, fiber type distribution, and proportional area. Further, by following PRISMA guidelines, we were able to assess the risk of bias of each article, as well as reduce researcher bias by having multiple authors screen, review, extract, and analyze data. Second, we did not include any participants with a medical diagnosis because of the potential impact on the sex differences typically observed in healthy populations. Third, we examined the influence of physical activity levels on skeletal muscle fiber type characteristics because of the different roles that sedentary behavior and active lifestyles could have had on findings. Fourth, we examined the effect of aging on the sex differences observed, as advanced age and fiber‐type specific atrophy could have impacted potential findings. Fifth, we examined the potential effect that different analysis techniques might have had on observed sex differences.

We also acknowledge several potential limitations of this review. First, we combined fiber types into two groups, type I and type II, and did not analyze based on IIa, IIx, and hybrid fibers. This limits conclusions that can be made about how factors such as age or physical activity impact the presence or size of these subgroupings of fiber types. Second, there is large variance in the not sedentary physical activity group because studies did not report physical activity levels using the same methods and questionnaires. Therefore, activity levels within the group were highly variable and may have ranged from moderately active to Olympic athletes. Third, while we are the first to investigate sex differences in the midlife age group, this midlife age group had the largest variance, which was likely due to the small number of studies, large age ranges of participants, and small numbers of participants. This emphasizes the need for more research to be performed on individuals 35–65 years of age. Further research including midlife ages should be performed to confirm these results. Fourth, we separated our muscle group biopsied by anatomical proximity due to small sample sizes of muscle groups in the upper body, limiting our ability to make specific conclusions about physiological differences. Future reviews separating muscles by function and action could answer physiologically relevant questions. Despite any potential limitations, our data provide novel evidence of significant biological sex differences in skeletal muscle fiber type characteristics.

## 5 CONCLUSION

This systematic review and meta‐analysis provide robust evidence that healthy adult males have larger CSA of type I and type II fibers, type II fiber type distribution, and type II proportional area than females, while females have greater type I fiber type distribution and type I proportional area when compared to males. Sex differences in CSA, fiber type distribution, and proportional area generally remained regardless of age, physical activity level, muscle group biopsied, and analysis technique. Our findings suggest there are inherent biological sex differences in skeletal muscle composition that appear to be unaltered by physical activity levels. These observations may be used to inform practitioners of potential causes of sex‐specific differences in aging and disease pathology and should be considered when developing sex‐specific exercise prescription and rehabilitation.

## AUTHOR CONTRIBUTIONS

J.J.J., M.L.M., A.E.S., and S.K.H. conceived and designed research. J.J.J., E.P.B., S.B.G., D.J.W., and A.G.P. reviewed and extracted data. M.L.M. conducted the analysis. J.J.J., M.L.M., A.E.S., and S.K.H. interpreted results of the analysis. J.J.J. and M.L.M. prepared figures and tables. J.J.J. and M.L.M. drafted the manuscript. J.J.J., M.L.M., E.P.B., S.B.G., D.J.W., A.G.P., A.E.S., and S.K.H. edited, revised, and approved the final version of the manuscript.

## FUNDING INFORMATION

This study was supported by the National Center for Advancing Translational Sciences, National Institutes of Health (NIH), through grant number 1TL1 TR001437 (to J.J.J.). Its contents are solely the responsibility of the authors and do not necessarily represent the official views of the NIH.

## CONFLICT OF INTEREST

No conflicts of interest, financial or otherwise, are declared by the authors.

## ETHICS STATEMENT

None.

## Supporting information


**Data S1.** PRISMA checklist.

**Data S2.** Search strategies.

**Data S3.** Table of full text review excluded studies and reasoning.

**Data S4.** Table of included studies and characteristics.

**Data S5.** Individual forest plots of each main and subgroup outcome.

**Data S6.** Risk of bias tool.

**Data S7.** Funnel plots of each main outcome.

## Data Availability

Additional data and files beyond what are provided in the supplemental material will be made available upon reasonable request.
