# Supplementary material for: Sex differences in human skeletal muscle fiber types and the influence of age, physical activity, and muscle group: A systematic review and meta‐analysis
Source: Physiol Rep. 2025 Nov 2;13(21):e70616. doi: 10.14814/phy2.70616 (PMC12580412; doi:10.14814/phy2.70616)
Supplement: Supplementary file 2 — Data S2. Search strategies. [file PHY2-13-e70616-s007.pdf]

PubMed

"Muscle, Skeletal"[Mesh] OR "Skeletal Muscle" OR "Muscle Fiber" OR "Muscle Fibre" OR "Muscle Fibers" OR "Muscle Fibres" OR "Muscle Fibers, Skeletal"[Mesh] OR "Skeletal Muscle Fiber" OR "Skeletal Muscle Fibre" OR "Skeletal Muscle Fibers" OR "Skeletal Muscle Fibres" OR "Skeletal Myocyte" OR "Skeletal Myocytes" OR Myofiber* OR Myofibre* OR "Muscle, Skeletal/physiology"[Mesh] OR "Muscle, Skeletal/metabolism"[Mesh] OR "Muscle, Skeletal/pathology" OR "Muscle, Skeletal/physiology*"

AND

"Biopsy"[Mesh] OR Biopsy OR Biopsies OR Biopsied OR "Muscle Biopsy" OR "Muscle Biopsies" OR "Muscle Sample" OR "Muscle Samples" OR "Myosin Heavy Chain" OR "Myosin Heavy Chains" OR "Myosin Heavy Chains/genetics" OR "Protein Isoform" OR "Protein Isoforms" OR "Muscle Homogenate" OR "Muscle Homogenates" OR "Muscle Fibers, Fast-Twitch"[Mesh] OR "Fast-Twitch Muscle Fiber" OR "Fast-Twitch Muscle Fibre" OR "Fast-Twitch Muscle Fibers" OR "Fast-Twitch Muscle Fibres" OR "Muscle Fibers, Slow-Twitch"[Mesh] OR "Slow-Twitch Muscle Fiber" OR "Slow-Twitch Muscle Fibre" OR "Slow-Twitch Muscle Fibers" OR "Slow-Twitch Muscle Fibres" OR "Type I Muscle Fiber" OR "Type I Muscle Fibre" OR "Type I Muscle Fibers" OR "Type I Muscle Fibres" OR "Type 1 Muscle Fiber" OR "Type 1 Muscle Fibre" OR "Type 1 Muscle Fibers" OR "Type 1 Muscle Fibres" OR "Type II Muscle Fiber" OR "Type II Muscle Fibre" OR "Type II Muscle Fibers" OR "Type II Muscle Fibres" OR "Type 2 Muscle Fiber" OR "Type 2 Muscle Fibre" OR "Type 2 Muscle Fibers" OR "Type 2 Muscle Fibres" OR "Type I and Type II" OR "Type 1 and Type 2" OR "Muscle Composition" OR "Muscle Fiber-Type Composition" OR "Muscle Fibre-Type Composition" OR "Muscle Fiber Area" OR "Muscle Fibre Area" OR "Fiber Area" OR "Fibre Area" OR "Proportion" OR "Proportional Area" OR "Percent Area" OR "Reference Values"[Mesh] OR "Reference Value" OR "Reference Values" OR "Reference Range" OR "Reference Ranges" OR "Normal Range" OR "Normal Ranges" OR "Normal Value" OR "Normal Values"

AND

"Male"[Mesh] OR Male* OR "Female"[Mesh] OR Female* OR Men OR Women OR "Sex Characteristics"[Mesh] OR "Sex Characteristic" OR "Sex Characteristics" OR "Sex Difference" OR "Sex Differences" OR "Sex Factors"[Mesh] OR "Sex Factor" OR "Sex Factors" OR "Sex Dimorphism" OR "Sex Dimorphisms" OR "Sexual Dimorphism" OR "Sexual Dimorphisms" OR "Sexual Dichromatism" OR "Gender Characteristic" OR "Gender Characteristics" OR "Gender Difference" OR "Gender Differences" OR "Gender Dimorphism" OR "Gender Dimorphisms"

Filters: Humans, English

The Cochrane Library

"Muscle Fiber" OR "Skeletal Muscle Fiber" OR "Skeletal Myocyte" OR "Myofiber" OR "Skeletal Muscle"

"Muscle, Skeletal" MESH TERM
"Muscle Fibers, Skeletal" MESH TERM

AND

"Biopsied" OR "Muscle Biopsy" OR "Myosin Heavy Chain" OR "Protein Isoform" OR "Muscle Homogenate" OR "Normal Range" OR "Normal Value" OR "Reference Range" OR "Muscle Sample" OR "Proportion" OR "Proportional Area" OR "Percent Area" OR "Fiber Area" OR "Muscle Fiber Area" OR "Fast-Twitch Muscle Fiber" OR "Slow-Twitch Muscle Fiber" OR "Muscle Composition" OR "Type I Muscle Fiber" OR "Type 1 Muscle Fiber" OR "Type II Muscle Fiber" OR "Type 2 Muscle Fiber" OR "Type I and Type II" OR "Type 1 and Type 2" OR "Muscle Fiber-Type Composition"

"Biopsy" MESH TERM
"Reference Values" MESH TERM
"Muscle Fibers, Fast-Twitch" MESH TERM
"Muscle Fibers, Slow-Twitch" MESH TERM

AND

"Sexual Dimorphism" OR "Gender Difference" OR "Sex Dimorphism" OR "Gender Characteristic" OR "Gender Dimorphism" OR "Sex Difference" OR "Sexual Dichromatism" OR Men OR Women

"Male" MESH TERM
"Female" MESH TERM
"Sex Characteristics" MESH TERM
"Sex Factors" MESH TERM

Web of Science

“muscle fib*” OR “Skeletal Muscle Fib*” OR “Skeletal Myocyte*” OR "Skeletal Muscle*" OR myofib*

AND

“Biops*” OR “Muscle Biops*” OR “Myosin Heavy Chain*” OR “Protein Isoform*” OR “Muscle Homogenate” OR “Reference Value” OR “Normal Range*” OR “Normal Value*” OR “Reference Range*” OR “muscle sample*” OR “Proportion” OR “Proportional Area” OR “percent area” OR ”fib* area” OR ”muscle fib* area” OR “Fast-Twitch Muscle Fib*” OR “Slow-Twitch Muscle Fib*” OR “Muscle Composition” OR “Type I Muscle Fib*”OR “Type II Muscle Fib*” OR “Type 1 Muscle Fib*” “Type 2 Muscle Fib*” OR “Type I and Type II” OR “Type 1 and Type 2” OR “Muscle Fib*-Type Composition”

AND

“Male*” OR “Female*” OR “Sex Characteristic*” OR “Sexual Dimorphism*” OR “Gender Difference*” OR “Sex Dimorphism*” OR “Gender Characteristic*” OR “Gender Dimorphism*” OR “Sex Difference*” OR “Sexual Dichromatism*” OR "Sex Factor*" OR “Men” OR “Women”

Filter by: English

CINHAL

“muscle fib*” OR “Skeletal Muscle Fib*” OR “Skeletal Myocyte*” OR myofib* OR (MH "Muscle, Skeletal")

AND

“Muscle Biops*” OR “Myosin Heavy Chain*” OR “Protein Isoform*” OR “Muscle Homogenate” OR “Normal Range*” OR “Normal Value*” OR “Reference Range*” OR “muscle sample*” OR “Proportion” OR “Proportional Area” OR “percent area” OR ”fib* area” OR ”muscle fib* area” OR “Fast-Twitch Muscle Fib*” OR “Slow-Twitch Muscle Fib*” OR “Muscle Composition” OR “Type I Muscle Fib*”OR “Type II Muscle Fib*” OR “Type 1 Muscle Fib*” “Type 2 Muscle Fib*” OR “Type I and Type II” OR “Type 1 and Type 2” OR “Muscle Fib*-Type Composition” OR (MH "Biopsy") OR (MH "Muscle Fibers") OR (MH "Reference Values")

AND

“Sex Characteristic*” OR “Sexual Dimorphism*” OR “Gender Difference*” OR “Sex Dimorphism*” OR “Gender Characteristic*” OR “Gender Dimorphism*” OR “Sex Difference*” OR “Sexual Dichromatism*” OR “Men” OR “Women” OR (MH "Male") OR (MH "Female") OR (MH "Sex Factors")

Filter: English

SPORTDiscus

“muscle fib*” OR “Skeletal Muscle Fib*” OR “Skeletal Myocyte*” OR myofib* OR DE "SKELETAL muscle" OR DE "SKELETAL muscle physiology" OR DE "MUSCLE metabolism"

AND

“Biops*” OR “Muscle Biops*” OR “Myosin Heavy Chain*” OR “Protein Isoform*” OR “Muscle Homogenate” OR “Reference Value” OR “Normal Range*” OR “Normal Value*” OR “Reference Range*” OR “muscle sample*” OR “Proportion” OR “Proportional Area” OR “percent area” OR ”fib* area” OR ”muscle fib* area” OR “Muscle Composition” OR “Type I Muscle Fib*”OR “Type II Muscle Fib*” OR “Type 1 Muscle Fib*” “Type 2 Muscle Fib*” OR “Type I and Type II” OR “Type 1 and Type 2” OR “Muscle Fib*-Type Composition” OR DE "FAST-twitch muscle fibers" OR DE "SLOW-twitch muscle fibers"

AND

“Male*” OR “Female*” OR “Sex Characteristic*” OR “Sexual Dimorphism*” OR “Gender Difference*” OR “Sex Dimorphism*” OR “Gender Characteristic*” OR “Gender Dimorphism*” OR “Sex Difference*” OR “Sexual Dichromatism*” OR "Sex Factor*" OR DE "MEN" OR DE "WOMEN"

Filter: English
