# Supplementary material for: Sex differences in human skeletal muscle fiber types and the influence of age, physical activity, and muscle group: A systematic review and meta‐analysis
Source: Physiol Rep. 2025 Nov 2;13(21):e70616. doi: 10.14814/phy2.70616 (PMC12580412; doi:10.14814/phy2.70616)
Supplement: Supplementary file 3 — Data S3. Table of full text review excluded studies and reasoning. [file PHY2-13-e70616-s003.pdf]

Full Text Review Excluded Studies and Reasoning Table

Title	Authors	Year	Exclusion Reason
Effects of resistance training on endurance capacity and muscle fiber composition in young top-level cyclists	Aagaard, P.; Andersen, J. L.; Bennekou, M.; Larsson, B.; Olesen, J. L.; Crameri, R.; Magnusson, S. P.; Kjaer, M.	2011	Does not include males and females
Mechanical muscle function, morphology, and fiber type in lifelong trained elderly	Aagaard, P.; Magnusson, P. S.; Larsson, B.; Kjaer, M.; Krustrup, P.	2007	Does not include males and females
Musculoskeletal adaptations to strength training in frail elderly: a matter of quantity or quality?	Aas, S. N.; Breit, M.; Karsrud, S.; Aase, O. J.; Rognlien, S. H.; Cumming, K. T.; Reggiani, C.; Seynnes, O.; Rossi, A. P.; Toniolo, L.; Raastad, T.	2020	Does not include male and female data
Effects of acute and chronic strength training on skeletal muscle autophagy in frail elderly men and women	Aas, S. N.; Tommerbakke, D.; Godager, S.; Nordseth, M.; Armani, A.; Sandri, M.; Benestad, H. B.; Raastad, T.	2020	Did not perform fiber type analysis
Mitochondrial reactive oxygen species generation in obese non-diabetic and type 2 diabetic participants	Abdul-Ghani, M. A.; Jani, R.; Chavez, A.; Molina-Carrion, M.; Tripathy, D.; DeFronzo, R. A.	2009	Did not perform fiber type analysis
Acute and chronic responses of skeletal-muscle to endurance and sprint exercise - a review	Abernethy, P. J.; Thayer, R.; Taylor, A. W.	1990	Review (ex: systematic review/meta-analysis)
Trained Integrated Postexercise Myofibrillar Protein Synthesis Rates Correlate with Hypertrophy in Young Males and Females	Abou Sawan, S.; Hodson, N.; Malowany, J. M.; West, D. W. D.; Tinline-Goodfellow, C.; Brook, M. S.; Smith, K.; Atherton, P. J.; Kumbhare, D.; Moore, D. R.	2022	Did not perform fiber type analysis
Incorporation of Dietary Amino Acids Into Myofibrillar and Sarcoplasmic Proteins in Free-Living Adults Is Influenced by Sex, Resistance Exercise, and Training Status	Abou Sawan, S.; Hodson, N.; Tinline-Goodfellow, C.; West, D. W. D.; Malowany, J. M.; Kumbhare, D.; Moore, D. R.	2021	Did not perform fiber type analysis
Proteomic signatures of in vivo muscle oxidative capacity in healthy adults	Adelnia, F.; Ubaida-Mohien, C.; Moaddel, R.; Shardell, M.; Lyashkov, A.; Fishbein, K. W.; Aon, M. A.; Spencer, R. G.; Ferrucci, L.	2020	Did not perform fiber type analysis
Skeletal muscle and cardiovascular adaptations to exercise conditioning in older coronary patients	Ades, P. A.; Waldmann, M. L.; Meyer, W. L.; Brown, K. A.; Poehlman, E. T.; Pendlebury, W. W.; Leslie, K. O.; Gray, P. R.; Lew, R. R.; LeWinter, M. M.	1996	Does not include male and female data
Effect of carbohydrate-protein supplementation on endurance training adaptations	Alghannam, A. F.; Templeman, I.; Thomas, J. E.; Jedrzejewski, D.; Griffiths, S.; Lemon, J.; Byers, T.; Reeves, S.; Gonzalez, J. T.; Thompson, D.; Bilzon, J.; Tsintzas, K.; Betts, J. A.	2020	Did not perform fiber type analysis
Density of nerve fibres and expression of substance P, NR2B-receptors and nerve growth factor in healthy human masseter muscle: An immunohistochemical study	Alhilou, A. M.; Shimada, A.; Svensson, C. I.; Ernberg, M.; Cairns, B. E.; Christidis, N.	2021	Did not perform fiber type analysis
Resveratrol Enhances Exercise-Induced Cellular and Functional Adaptations of Skeletal Muscle in Older Men and Women	Alway, S. E.; McCrory, J. L.; Kearcher, K.; Vickers, A.; Frear, B.; Gilleland, D. L.; Bonner, D. E.; Thomas, J. M.; Donley, D. A.; Lively, M. W.; Mohamed, J. S.	2017	Does not include male and female data

Muscle fibre type adaptation in the elderly human muscle	Andersen, J. L.	2003	Review (ex: systematic review/meta-analysis)
Resistance training and insulin action in humans: effects of de-training	Andersen, J. L.; Scljerling, P.; Andersen, L. L.; Dela, F.	2003	Does not include males and females
Increase in the degree of coexpression of myosin heavy chain isoforms in skeletal muscle fibers of the very old	Andersen, J. L.; Terzis, G.; Kryger, A.	1999	Does not include male and female data
Fatty acid composition of skeletal muscle reflects dietary fat composition in humans	Andersson, A.; Nalsen, C.; Tengblad, S.; Vessby, B.	2002	Did not perform fiber type analysis
Improving Strength, Power, Muscle Aerobic Capacity, and Glucose Tolerance through Short-term Progressive Strength Training Among Elderly People	Andersson, E. A.; Frank, P.; Ponten, M.; Ekblom, B.; Ekblom, M.; Moberg, M.; Sahlin, K.	2017	Does not include male and female data
Striated muscle fiber size, composition, and capillary density in diabetes in relation to neuropathy and muscle strength	Andreassen, C. S.; Jensen, J. M.; Jakobsen, J.; Ulhoj, B. P.; Andersen, H.	2014	Does not include male and female data
Aging and Muscle Function	Aoyagi, Y.; Shephard, R. J.	1992	Review (ex: systematic review/meta-analysis)
Influence of supplementation with branched-chain amino acids in combination with resistance exercise on p70S6 kinase phosphorylation in resting and exercising human skeletal muscle	Apro, W.; Blomstrand, E.	2010	Did not perform fiber type analysis
Fourteen days of bed rest induces a decline in satellite cell content and robust atrophy of skeletal muscle fibers in middle-aged adults	Arentson-Lantz, E. J.; English, K. L.; Paddon-Jones, D.; Fry, C. S.	2016	Does not include male and female data
Countering disuse atrophy in older adults with low-volume leucine supplementation	Arentson-Lantz, E. J.; Fiebig, K. N.; Anderson-Catania, K. J.; Deer, R. R.; Wacher, A.; Fry, C. S.; Lamon, S.; Paddon-Jones, D.	2020	Does not include male and female data
Skeletal Muscle Glycogen Content at Rest and During Endurance Exercise in Humans: A Meta-Analysis	Areta, J. L.; Hopkins, W. G.	2018	Review (ex: systematic review/meta-analysis)
Distinct patterns of skeletal muscle mitochondria fusion, fission and mitophagy upon duration of exercise training	Arribat, Y.; Broskey, N. T.; Greggio, C.; Boutant, L.; Alonso, S. C.; Kulkarni, S. S.; Lagarrigue, S.; Carnero, E. A.; Besson, C.; Canto, C.; Amati, F.	2019	Did not perform fiber type analysis
Characterization of adipocytes derived from fibro/adipogenic progenitors resident in human skeletal muscle	Arrighi, N.; Moratal, C.; Clement, N.; Giorgetti-Peraldi, S.; Peraldi, P.; Loubat, A.; Kurzenne, J. Y.; Dani, C.; Chopard, A.; Dechesne, C. A.	2015	Only in children (0-17 years)
New Insights on Bone Tissue and Structural Muscle-Bone Unit in Constitutional Thinness	Bailly, M.; Boscaro, A.; Thomas, T.; Feasson, L.; Costes, F.; Pereira, B.; Hager, J.; Estour, B.; Galusca, B.; Metz, L.; Courteix, D.; Thivel, D.; Verney, J.; Germain, N.	2022	Does not include male and female data

Reduced Respiratory Capacity in Muscle Mitochondria of Obese Subjects	Bakkman, L.; Fernstrom, M.; Loogna, P.; Rooyackers, O.; Brandt, L.; Lagerros, Y. T.	2010	Does not include males and females
Effects of aging on in vivo synthesis of skeletal muscle myosin heavy-chain and sarcoplasmic protein in humans	Balagopal, P.; Rooyackers, O. E.; Adey, D. B.; Ades, P. A.; Nair, K. S.	1997	Did not perform fiber type analysis
Age effect on transcript levels and synthesis rate of muscle MHC and response to resistance exercise	Balagopal, P.; Schimke, J. C.; Ades, P.; Adey, D.; Nair, K. S.	2001	Does not report sex of subjects
Cluster analysis tests the importance of myogenic gene expression during myofiber hypertrophy in humans	Bamman, M. M.; Petrella, J. K.; Kim, J. S.; Mayhew, D. L.; Cross, J. M.	2007	Did not perform fiber type analysis
Myogenic protein expression before and after resistance loading in 26-and 64-yr-old men and women	Bamman, M. M.; Ragan, R. C.; Kim, J. S.; Cross, J. M.; Hill, V. J.; Tuggle, S. C.; Allman, R. M.	2004	Did not perform fiber type analysis
Fibre type-specific satellite cell content in two models of muscle disease	Bankole, L. C.; Feasson, L.; Ponsot, E.; Kadi, F.	2013	Does not report sex of subjects
Aging, sex differences, and oxidative stress in human respiratory and limb muscles	Barreiro, E.; Coronell, C.; Lavina, B.; Ramirez-Sarmiento, A.; Orozco-Levi, M.; Gea, J.; Penam Project	2006	Did not perform fiber type analysis
Acute Exercise Remodels Promoter Methylation in Human Skeletal Muscle	Barres, R.; Yan, J.; Egan, B.; Treebak, J. T.; Rasmussen, M.; Fritz, T.; Caidahl, K.; Krook, A.; O'Gorman, D. J.; Zierath, J. R.	2012	Did not perform fiber type analysis
Anthropometric Characteristics Account for Time to Exhaustion in Cycling	Basset, F. A.; Billaut, F.; Joanisse, D. R.	2014	Does not include male and female data
Effects of short-term normobaric hypoxia on haematology, muscle phenotypes and physical performance in highly trained athletes	Basset, F. A.; Joanisse, D. R.; Boivin, F.; St-Onge, J.; Billaut, F.; Dore, J.; Chouinard, R.; Falgairette, G.; Richard, D.; Boulay, M. R.	2006	Does not include male and female data
Sexually differentiated, androgen-regulated, larynx-specific myosin heavy-chain isoforms in Xenopus tropicalis; comparison to Xenopus laevis	Baur, L. A.; Nasipak, B. T.; Kelley, D. B.	2008	Animal study
The fatty acid composition of skeletal muscle membrane phospholipid: Its relationship with the type of feeding and plasma glucose levels in young children	Baur, L. A.; O'Connor, J.; Pan, D. A.; Kriketos, A. D.; Storlien, L. H.	1998	Only in children (0-17 years)
Protein-Rich Food Ingestion Stimulates Mitochondrial Protein Synthesis in Sedentary Young Adults of Different BMIs	Beals, J. W.; Mackenzie, R. W. A.; van Vliet, S.; Skinner, S. K.; Pagni, B. A.; Niemiro, G. M.; Ulanov, A. V.; Li, Z.; Dilger, A. C.; Paluska, S. A.; De Lisio, M.; Burd, N. A.	2017	Did not perform fiber type analysis
Anabolic sensitivity of postprandial muscle protein synthesis to the ingestion of a protein-dense food is reduced in overweight and obese young adults	Beals, J. W.; Sukiennik, R. A.; Nallabelli, J.; Emmons, R. S.; van Vliet, S.; Young, J. R.; Ulanov, A. V.; Li, Z.; Paluska, S. A.; De Lisio, M.; Burd, N. A.	2016	Did not perform fiber type analysis
Contraction-induced muscle damage in humans following calcium channel blocker administration	Beaton, L. J.; Tarnopolsky, M. A.; Phillips, S. M.	2002	Does not include males and females
Human muscle satellite cells show age-related differential expression of S100B protein and RAGE	Beccafico, S.; Riuzzi, F.; Puglielli, C.; Mancinelli, R.; Fulle, S.; Sorci, G.; Donato, R.	2011	Does not include male and female data

Muscle characteristics in patients with chronic systemic inflammation	Beenakker, K. G. M.; Duijnisveld, B. J.; Van Der Linden, H. M. J.; Visser, C. P. J.; Westendorp, R. G. J.; Butler-Brown, G.; Nelissen, Rghh; Maier, A. B.	2012	No healthy subjects or controls
Effect of concurrent strength and endurance training on skeletal muscle properties and hormone concentrations in humans	Bell, G. J.; Syrotuik, D.; Martin, T. P.; Burnham, R.; Quinney, H. A.	2000	Does not include male and female data
Single-blind study of dystrophin staining in carriers of duchenne muscular-dystrophy	Bernier, F. P.; Greenberg, C. R.; Halliday, W. C.; Wrogemann, K.	1993	Did not perform fiber type analysis
Human skeletal muscle carnitine palmitoyltransferase I activity determined in isolated intact mitochondria	Berthon, P. M.; Howlett, R. A.; Heigenhauser, G. J. F.; Spriet, L. L.	1998	Did not perform fiber type analysis
A morphometric study on human muscle mitochondria in aging	Bertoni-Freddari, C.; Fattoretti, P.; Caselli, U.; Giorgetti, B.; Albanelli, S.; Torelli, F.; Felzani, G.; Vecchiet, J.	2002	Does not include males and females
Sex Differences in Endurance Running	Besson, T.; Macchi, R.; Rossi, J.; Morio, C. Y. M.; Kunimasa, Y.; Nicol, C.; Vercruyssen, F.; Millet, G. Y.	2022	Review (ex: systematic review/meta-analysis)
High expression of CPT1b in skeletal muscle in metabolically healthy older subjects	Betry, C.; Meugnier, E.; Pflieger, M.; Grenet, G.; Hercberg, S.; Galan, P.; Kesse-Guyot, E.; Vidal, H.; Laville, M.	2019	Did not perform fiber type analysis
Effects of 5 ppm hydrogen sulfide inhalation on biochemical properties of skeletal muscle in exercising men and women	Bhambhani, Y.; Burnham, R.; Snydmiller, G.; MacLean, I.; Martin, T.	1996	Did not perform fiber type analysis
Do skeletal muscle composition and gene expression as well as acute exercise-induced serum adaptations in older adults depend on fitness status?	Bizjak, D. A.; Zugel, M.; Schumann, U.; Tully, M. A.; Dallmeier, D.; Denkinger, M.; Steinacker, J. M.	2021	Does not include male and female data
Growth hormone treatment prevents the decrease in insulin-like growth factor I gene expression in patients undergoing abdominal surgery	Bjarnason, R.; Wickelgren, R.; Hermansson, M.; Hammarqvist, F.; Carlsson, B.; Carlsson, L. M. S.	1998	Did not perform fiber type analysis
Delayed myonuclear addition, myofiber hypertrophy, and increases in strength with high-frequency low-load blood flow restricted training to volitional failure	Bjornsen, T.; Wernbom, M.; Lovstad, A.; Paulsen, G.; D'Souza, R. F.; Cameron-Smith, D.; Flesche, A.; Hisdal, J.; Berntsen, S.; Raastad, T.	2019	Does not include male and female data
High-frequency blood flow-restricted resistance exercise results in acute and prolonged cellular stress more pronounced in type I than in type II fibers	Bjornsen, T.; Wernbom, M.; Paulsen, G.; Markworth, J. F.; Berntsen, S.; D'Souza, R. F.; Cameron-Smith, D.; Raastad, T.	2021	Does not include male and female data
Plasma FFA utilization and fatty acid-binding protein content are diminished in type 2 diabetic muscle	Blaak, E. E.; Wagenmakers, A. J. M.; Glatz, J. F. C.; Wolffenbuttel, B. H. R.; Kemerink, G. J.; Langenberg, C. J. M.; Heidendal, G. A. K.; Saris, W. H. M.	2000	Does not include males and females
The physiological impact of high-intensity interval training in octogenarians with comorbidities	Blackwell, J. E. M.; Gharahdaghi, N.; Brook, M. S.; Watanabe, S.; Boereboom, C. L.; Doleman, B.; Lund, J. N.; Wilkinson, D. J.; Smith, K.; Atherton, P. J.; Williams, J. P.; Phillips, B. E.	2021	Did not perform fiber type analysis

Effects of Histidine and beta-alanine Supplementation on Human Muscle Carnosine Storage	Blancquaert, L.; Everaert, I.; Missinne, M.; Baguet, A.; Stegen, S.; Volkaert, A.; Petrovic, M.; Vervaet, C.; Achten, E.; De Maeyer, M.; De Henauw, S.; Derave, W.	2017	Did not perform fiber type analysis
Primary human muscle satellite cell culture: Variations of cell yield, proliferation and differentiation rates according to age and sex of donors, site of muscle biopsy, and delay before processing	Bonavaud, S.; Thibert, P.; Gherardi, R. K.; Barlovatz-Meimon, G.	1997	Did not perform fiber type analysis
Progressive disorganization of the excitation-contraction coupling apparatus in aging human skeletal muscle as revealed by electron microscopy: A possible role in the decline of muscle performance	Boncompagni, S.; d'Amelio, L.; Fulle, S.; Fano, G.; Protasi, F.	2006	Did not perform fiber type analysis
Eccentric exercise markedly increases c-Jun NH2-terminal kinase activity in human skeletal muscle	Boppart, M. D.; Aronson, D.; Gibson, L.; Roubenoff, R.; Abad, L. W.; Bean, J.; Goodyear, L. J.; Fielding, R. A.	1999	Did not perform fiber type analysis
Increased mitophagy in the skeletal muscle of spinal and bulbar muscular atrophy patients	Borgia, D.; Malena, A.; Spinazzi, M.; Desbats, M. A.; Salviati, L.; Russell, A. P.; Miotto, G.; Tosatto, L.; Pegoraro, E.; Soraru, G.; Pennuto, M.; Vergani, L.	2017	Did not perform fiber type analysis
Low-intensity training increases peak arm VO2 by enhancing both convective and diffusive O-2 delivery	Boushel, R.; Ara, I.; Gnaiger, E.; Helge, J. W.; Gonzalez-Alonso, J.; Munck-Andersen, T.; Sondergaard, H.; Damsgaard, R.; van Hall, G.; Saltin, B.; Calbet, J. A. L.	2014	Does not include male and female data
Maintained peak leg and pulmonary VO2 despite substantial reduction in muscle mitochondrial capacity	Boushel, R.; Gnaiger, E.; Larsen, F. J.; Helge, J. W.; Gonzalez-Alonso, J.; Ara, I.; Munch-Andersen, T.; van Hall, G.; Sondergaard, H.; Saltin, B.; Calbet, J. A. L.	2015	Does not include male and female data
A High-Fat Diet Elicits Differential Responses in Genes Coordinating Oxidative Metabolism in Skeletal Muscle of Lean and Obese Individuals	Boyle, K. E.; Canham, J. P.; Consitt, L. A.; Zheng, D.; Koves, T. R.; Gavin, T. P.; Holbert, D.; Neufer, P. D.; Ilkayeva, O.; Muoio, D. M.; Houmard, J. A.	2011	Did not perform fiber type analysis
Plasma and Muscle Myostatin in Relation to Type 2 Diabetes	Brandt, C.; Nielsen, A. R.; Fischer, C. P.; Hansen, J.; Pedersen, B. K.; Plomgaard, P.	2012	Did not perform fiber type analysis
Effect of protein overfeeding on energy expenditure measured in a metabolic chamber	Bray, G. A.; Redman, L. M.; de Jonge, L.; Covington, J.; Rood, J.; Brock, C.; Mancuso, S.; Martin, C. K.; Smith, S. R.	2015	Did not perform fiber type analysis
Two Weeks of Reduced Activity Decreases Leg Lean Mass and Induces "Anabolic Resistance" of Myofibrillar Protein Synthesis in Healthy Elderly	Breen, L.; Stokes, K. A.; Churchward-Venne, T. A.; Moore, D. R.; Baker, S. K.; Smith, K.; Atherton, P. J.; Phillips, S. M.	2013	Did not perform fiber type analysis
Skeletal muscle mitochondrial and lipid droplet content assessed with standardized grid sizes for stereology	Broskey, N. T.; Daraspe, J.; Humbel, B. M.; Amati, F.	2013	Did not perform fiber type analysis
Aerobic conditioning alters the satellite cell and ribosome response to acute eccentric contractions in young men and women	Brown, A.; Thomas, A. C. Q.; Hatt, A. A.; McGlory, C.; Phillips, S. M.; Kumbhare, D.; Parise, G.; Joanisse, S.	2022	Does not include male and female data
Disassociation of muscle triglyceride content and insulin sensitivity after exercise training in patients with Type 2 diabetes	Bruce, C. R.; Kriketos, A. D.; Cooney, G. J.; Hawley, J. A.	2004	Does not include males and females

Effect of exercise training on skeletal muscle protein expression in relation to insulin sensitivity: Per-protocol analysis of a randomized controlled trial (GO-ACTIWE)	Bruhn, L.; Kjobsted, R.; Quist, J. S.; Gram, A. S.; Rosenkilde, M.; Faerch, K.; Wojtaszewski, J. F. P.; Stallknecht, B.; Blond, M. B.	2021	Did not perform fiber type analysis
Effects of aging on type II muscle fibers: A systematic review of the literature	Brunner, F.; Schmid, A.; Sheikhzadeh, A.; Nordin, M.; Yoon, J.; Frankel, V.	2007	Review (ex: systematic review/meta-analysis)
Changes in glucose and lipid metabolism following weight loss produced by a very low calorie diet in obese subjects	Bryson, J. M.; King, S. E.; Burns, C. M.; Baur, L. A.; Swaraj, S.; Caterson, I. D.	1996	Did not perform fiber type analysis
Effect of creatine supplementation and resistance-exercise training on muscle insulin-like growth factor in young adults	Burke, D. G.; Candow, D. G.; Chilibeck, P. D.; MacNeil, L. G.; Roy, B. D.; Tarnopolsky, M. A.; Ziegenfuss, T.	2008	Does not include male and female data
Muscle regeneration following repair of the rotator cuff	Butt, U.; Rashid, M. S.; Temperley, D.; Crank, S.; Birch, A.; Freemont, A. J.; Trail, I. A.	2016	No healthy subjects or controls
Morphometric studies on myonuclei in athletes	Cabric, M.; James, N. T.; Resic, A.	1998	Full text not available
Fiber typing of the erector spinae and multifidus muscles in healthy controls and back pain patients: a systematic literature review	Cagnie, B.; Dhooge, F.; Schumacher, C.; De Meulemeester, K.; Petrovic, M.; van Oosterwijck, J.; Danneels, L.	2015	Review (ex: systematic review/meta-analysis)
Age-related structural alterations in human skeletal muscle fibers and mitochondria are sex specific: relationship to single-fiber function	Callahan, D. M.; Bedrin, N. G.; Subramanian, M.; Berking, J.; Ades, P. A.; Toth, M. J.; Miller, M. S.	2014	Single Fiber Analysis
Muscle disuse alters skeletal muscle contractile function at the molecular and cellular levels in older adult humans in a sex-specific manner	Callahan, D. M.; Miller, M. S.; Sweeny, A. P.; Tourville, T. W.; Slauterbeck, J. R.; Savage, P. D.; Maugan, D. W.; Ades, P. A.; Beynnon, B. D.; Toth, M. J.	2014	Does not include male and female data
Chronic disuse and skeletal muscle structure in older adults: sex-specific differences and relationships to contractile function	Callahan, D. M.; Tourville, T. W.; Miller, M. S.; Hackett, S. B.; Sharma, H.; Cruickshank, N. C.; Slauterbeck, J. R.; Savage, P. D.; Ades, P. A.; Maughan, D. W.; Beynnon, B. D.; Toth, M. J.	2015	Does not include male and female data
Reduced rate of knee extensor torque development in older adults with knee osteoarthritis is associated with intrinsicmuscle contractile deficits	Callahan, D. M.; Tourville, T. W.; Slauterbeck, J. R.; Ades, P. A.; Stevens-Lapsley, J.; Beynnon, B. D.; Toth, M. J.	2015	Does not include male and female data
Ubiquitin Proteasome System Activity is Suppressed by Curcumin following Exercise-Induced Muscle Damage in Human Skeletal Muscle	Cardaci, T. D.; Machek, S. B.; Wilburn, D. T.; Hwang, P. S.; Willoughby, D. S.	2021	Did not perform fiber type analysis
Superior Intrinsic Mitochondria Respiration in Women Than in Men	Cardinale, D. A.; Larsen, F. J.; Schiffer, T. A.; Morales-Alamo, D.; Ekblom, B.; Calbet, J. A. L.; Holmberg, H. C.; Boushel, R.	2018	Did not perform fiber type analysis
Skeletal Muscle Ras-Related GTP Binding B mRNA and Protein Expression Is Increased after Essential Amino Acid Ingestion in Healthy Humans	Carlin, M. B.; Tanner, R. E.; Agergaard, J.; Jalili, T.; McClain, D. A.; Drummond, M. J.	2014	Did not perform fiber type analysis

Contractile protein concentrations in human single muscle fibers	Carroll, C. C.; Carrithers, J. A.; Trappe, T. A.	2004	Does not include male and female data
Resistance training frequency: strength and myosin heavy chain responses to two and three bouts per week	Carroll, T. J.; Abernethy, P. J.; Logan, P. A.; Barber, M.; McEniery, M. T.	1998	Does not include male and female data
Exercise and beta-alanine supplementation on carnosine-acrolein adduct in skeletal muscle	Carvalho, V. H.; Oliveira, A. H. S.; de Oliveira, L. F.; da Silva, R. P.; Di Mascio, P.; Gualano, B.; Artioli, G. G.; Medeiros, M. H. G.	2018	Does not report sex of subjects
Leucine supplementation chronically improves muscle protein synthesis in older adults consuming the RDA for protein	Casperson, S. L.; Sheffield-Moore, M.; Hewlings, S. J.; Paddon-Jones, D.	2012	Did not perform fiber type analysis
The Combined Oral Stable Isotope Assessment of Muscle (COSIAM) reveals D-3 creatine derived muscle mass as a standout cross-sectional biomarker of muscle physiology vitality in older age	Cegielski, J.; Brook, M. S.; Phillips, B. E.; Boereboom, C.; Gates, A.; Gladman, J. F. R.; Smith, K.; Wilkinson, D. J.; Atherton, P. J.	2022	Did not perform fiber type analysis
Muscle Protein Synthesis after Protein Administration in Critical Illness	Chapple, L. A. S.; Kouw, I. W. K.; Summers, M. J.; Weinel, L. M.; Gluck, S.; Raith, E.; Slobodian, P.; Soenen, S.; Deane, A. M.; van Loon, L. J. C.; Chapman, M. J.	2022	Did not perform fiber type analysis
Effect of endurance training on muscle microvascular filtration capacity and vascular bed morphometry in the elderly	Charles, M.; Charifi, N.; Verney, J.; Pichot, V.; Feasson, L.; Costes, F.; Denis, C.	2006	Does not include males and females
Comparison of muscle mechanical and histochemical properties between young and elderly subjects	Chelly, M. S.; Chamari, K.; Verney, J.; Denis, C.	2006	Does not include males and females
Fiber type and myosin heavy chain compositions of adult pretarsal orbicularis oculi muscle	Cheng, N. C.; Liao, S. L.; Wang, I. J.; Lin, I. C.; Tang, Y. B.	2007	Does not include male and female data
Type IIB human skeletal muscle fibers positively correlate with bone mineral density irrespective to age	Cheung, W. H.; Lee, W. S.; Qin, L.; Tang, N.; Hung, V. W. Y.; Leung, K. S.	2010	No healthy subjects or controls
Muscle capillarization, O-2 diffusion distance, and Vo(2) kinetics in old and young individuals	Chilibeck, P. D.; Paterson, D. H.; Cunningham, D. A.; Taylor, A. W.; Noble, E. G.	1997	Does not include male and female data
The effect of concurrent endurance and strength training on quantitative estimates of subsarcolemmal and intermyofibrillar mitochondria	Chilibeck, P. D.; Syrotuik, D. G.; Bell, G. J.	2002	Does not include male and female data
Eccentric contraction-induced injury to type I, IIa, and IIa/IIx muscle fibers of elderly adults	Choi, S. J.; Lim, J. Y.; Nibaldi, E. G.; Phillips, E. M.; Frontera, W. R.; Fielding, R. A.; Widrick, J. J.	2012	Does not include male and female data
Skeletal Muscle Mitochondria in Insulin Resistance: Differences in Intermyofibrillar Versus Subsarcolemmal Subpopulations and Relationship to Metabolic Flexibility	Chomentowski, P.; Coen, P. M.; Radikova, Z.; Goodpaster, B. H.; Toledo, F. G. S.	2011	Does not include male and female data
Moderate Exercise Attenuates the Loss of Skeletal Muscle Mass That Occurs With Intentional Caloric Restriction-Induced Weight Loss in Older, Overweight to Obese Adults	Chomentowski, P.; Dube, J. J.; Amati, F.; Stefanovic-Racic, M.; Zhu, S. J.; Toledo, F. G. S.; Goodpaster, B. H.	2009	Does not include male and female data

Safety and efficacy of resistance training in germ cell cancer patients undergoing chemotherapy: a randomized controlled trial	Christensen, J. F.; Jones, L. W.; Tolver, A.; Jorgensen, L. W.; Andersen, J. L.; Adamsen, L.; Hojman, P.; Nielsen, R. H.; Rorth, M.; Daugaard, G.	2014	Does not include male and female data
Diet-Induced Weight Loss and Exercise Alone and in Combination Enhance the Expression of Adiponectin Receptors in Adipose Tissue and Skeletal Muscle, but Only Diet-Induced Weight Loss Enhanced Circulating Adiponectin	Christiansen, T.; Paulsen, S. K.; Bruun, J. M.; Ploug, T.; Pedersen, S. B.; Richelsen, B.	2010	Did not perform fiber type analysis
Expression of 5-HT3 receptors and TTX resistant sodium channels (Na(V)1.8) on muscle nerve fibers in pain-free humans and patients with chronic myofascial temporomandibular disorders	Christidis, N.; Kang, I.; Cairns, B.; Kumar, U.; Dong, X. D.; Rosen, A.; Kopp, S.; Ernberg, M.	2014	Did not perform fiber type analysis
Defects of insulin action and skeletal muscle glucose metabolism in growth hormone-deficient adults persist after 24 months of recombinant human growth hormone therapy	Christopher, M.; Hew, F. L.; Oakley, M.; Rantzau, C.; Alford, F.	1998	Did not perform fiber type analysis
Mitochondrial adaptations to high intensity interval training in older females and males	Chrois, K. M.; Dohlmann, T. L.; Sogaard, D.; Hansen, C. V.; Dela, F.; Helge, J. W.; Larsen, S.	2020	Did not perform fiber type analysis
Resting skeletal muscle PNPLA2 (ATGL) and CPT1B are associated with peak fat oxidation rates in men and women but do not explain observed sex differences	Chrzanowski-Smith, O. J.; Edinburgh, R. M.; Smith, E.; Thomas, M. P.; Walhin, J. P.; Koumanov, F.; Williams, S.; Betts, J. A.; Gonzalez, J. T.	2021	Did not perform fiber type analysis
Intramyocellular Ceramides: Subcellular Concentrations and Fractional De Novo Synthesis in Postabsorptive Humans	Chung, J. O.; Koutsari, C.; Blachnio-Zabielska, A. U.; Hames, K. C.; Jensen, M. D.	2017	Did not perform fiber type analysis
Effects of meal ingestion on intramyocellular ceramide concentrations and fractional de novo synthesis in humans	Chung, J. O.; Koutsari, C.; Blachnio-Zablieska, A. U.; Hames, K. C.; Jensen, M. D.	2018	Did not perform fiber type analysis
Comparison of Two -Alanine Dosing Protocols on Muscle Carnosine Elevations	Church, D. D.; Hoffman, J. R.; Varanoske, A. N.; Wang, R.; Baker, K. M.; La Monica, M. B.; Beyer, K. S.; Dodd, S. J.; Oliveira, L. P.; Harris, R. C.; Fukuda, D. H.; Stout, J. R.	2017	Did not perform fiber type analysis
Human Sarcopenic Myoblasts Can Be Rescued by Pharmacological Reactivation of HIF-1 alpha	Cirillo, F.; Mangiavini, L.; La Rocca, P.; Piccoli, M.; Ghiroldi, A.; Rota, P.; Tarantino, A.; Canciani, B.; Coviello, S.; Messina, C.; Ciconte, G.; Pappone, C.; Peretti, G. M.; Anastasia, L.	2022	Did not perform fiber type analysis
mRNA Expression of Myosin Heavy Chain Isoforms in the Sphenomandibularis Portion of the Temporalis Muscle	Ciurana, N.; Artells, R.; Casado, A.; Potau, J. M.	2022	Autopsies
Effects of high- and low-velocity resistance training on the contractile properties of skeletal muscle fibers from young and older humans	Claflin, D. R.; Larkin, L. M.; Cederna, P. S.; Horowitz, J. F.; Alexander, N. B.; Cole, N. M.; Galecki, A. T.; Chen, S.; Nyquist, L. V.; Carlson, B. M.; Faulkner, J. A.; Ashton-Miller, J. A.	2011	Single Fiber Analysis
Skeletal muscle phosphatidylcholine fatty acids and insulin sensitivity in normal humans	Clore, J. N.; Li, J.; Gill, R.; Gupta, S.; Spencer, R.; Azzam, A.; Zuelzer, W.; Rizzo, W. B.; Blackard, W. G.	1998	Does not include male and female data

Skeletal Muscle Mitochondrial Energetics Are Associated With Maximal Aerobic Capacity and Walking Speed in Older Adults	Coen, P. M.; Jubrias, S. A.; Distefano, G.; Amati, F.; Mackey, D. C.; Glynn, N. W.; Manini, T. M.; Wohlgemuth, S. E.; Leeuwenburgh, C.; Cummings, S. R.; Newman, A. B.; Ferrucci, L.; Toledo, F. G. S.; Shankland, E.; Conley, K. E.; Goodpaster, B. H.	2013	Did not perform fiber type analysis
Aerobic Plus Resistance Exercise in Obese Older Adults Improves Muscle Protein Synthesis and Preserves Myocellular Quality Despite Weight Loss	Colleluori, G.; Aguirre, L.; Phadnis, U.; Fowler, K.; Armamento-Villarea, R.; Sun, Z.; Brunetti, L.; Park, J. H.; Kaipparettu, B. A.; Putluri, N.; Auetumrongsawat, V.; Yarasheski, K.; Qualls, C.; Villarea, D. T.	2019	Did not perform fiber type analysis
Oxidative capacity and ageing in human muscle	Conley, K. E.; Jubrias, S. A.; Esselman, P. C.	2000	Did not perform fiber type analysis
Exercise capacity in chronic heart failure patients is related to active gene transcription in skeletal muscle and not apoptosis	Conraads, V. M.; Hoymans, V. Y.; Vermeulen, T.; Beckers, P.; Possemiers, N.; de Maeseneer, M.; Vrints, C.; Martinet, W.	2009	Did not perform fiber type analysis
Plasma acylcarnitines during insulin stimulation in humans are reflective of age-related metabolic dysfunction	Consitt, L. A.; Koves, T. R.; Muoio, D. M.; Nakazawa, M.; Newton, C. A.; Houmard, J. A.	2016	Did not perform fiber type analysis
Age-related impairments in skeletal muscle PDH phosphorylation and plasma lactate are indicative of metabolic inflexibility and the effects of exercise training	Consitt, L. A.; Saxena, G.; Saneda, A.; Houmard, J. A.	2016	Did not perform fiber type analysis
Reverse fiber type disproportion: A distinct metabolic myopathy	Cooper, C. B.; Dolezal, B. A.; Riley, M.; Verity, M. A.; Shieh, P. B.	2016	No healthy subjects or controls
Concurrent Exercise on a Gravity-Independent Device during Simulated Microgravity	Cotter, J. A.; Yu, A.; Haddad, F.; Kreitenberg, A.; Baker, M. J.; Tesch, P. A.; Baldwin, K. M.; Caiozzo, V. J.; Adams, G. R.	2015	Did not perform fiber type analysis
A Single Bout of Ultra-Endurance Exercise Reveals Early Signs of Muscle Aging in Master Athletes	Coudy-Gandilhon, C.; Gueugneau, M.; Chambon, C.; Taillandier, D.; Combaret, L.; Polge, C.; Millet, G. Y.; Feasson, L.; Bechet, D.	2022	Does not include males and females
Cycling efficiency is related to the percentage of type-i muscle-fibers	Coyle, E. F.; Sidossis, L. S.; Horowitz, J. F.; Beltz, J. D.	1992	Does not include males and females
The Effect of Aging on Human Skeletal Muscle Mitochondrial and Intramyocellular Lipid Ultrastructure	Crane, J. D.; Devries, M. C.; Safdar, A.; Hamadeh, M. J.; Tarnopolsky, M. A.	2010	Did not perform fiber type analysis
Supplemental Oxygen Improves In Vivo Mitochondrial Oxidative Phosphorylation Flux in Sedentary Obese Adults With Type 2 Diabetes	Cree-Green, M.; Scalzo, R. L.; Harrall, K.; Newcomer, B. R.; Schauer, I. E.; Huebschmann, A. G.; McMillin, S.; Brown, M. S.; Orlicky, D.; Knaub, L.; Nadeau, K. J.; McClatchey, P. M.; Bauer, T. A.; Regensteiner, J. G.; Reusch, J. E. B.	2018	Does not include male and female data
Influence of muscle glycogen availability on ERK1/2 and Akt signaling after resistance exercise in human skeletal muscle	Creer, A.; Gallagher, P.; Slivka, D.; Jemiolo, B.; Fink, W.; Trappe, S.	2005	Does not include males and females

Effects of aging and gender on the spatial organization of nuclei in single human skeletal muscle cells	Cristea, A.; Qaisar, R.; Edlund, P. K.; Lindblad, J.; Bengtsson, E.; Larsson, L.	2010	Single Fiber Analysis
Muscular HSP70 content is higher in elderly compared to young, but is normalized after 12 weeks of strength training	Cumming, K. T.; Kvamme, N. H.; Schaad, L.; Ugelstad, I.; Raastad, T.	2021	Did not perform fiber type analysis
Histology of skeletal-muscle in adults with gh deficiency - comparison with normal muscle and response to gh treatment	Cuneo, R. C.; Salomon, F.; Wiles, C. M.; Round, J. M.; Jones, D.; Hesp, R.; Sonksen, P. H.	1992	Does not include male and female data
Omega-3 Supplementation Improves Isometric Strength But Not Muscle Anabolic and Catabolic Signaling in Response to Resistance Exercise in Healthy Older Adults	Dalle, S.; Van Roie, E.; Hiroux, C.; Vanmunster, M.; Coudyzer, W.; Suhr, F.; Bogaerts, S.; Van Thienen, R.; Koppo, K.	2021	Did not perform fiber type analysis
High intensity exercise downregulates FTO mRNA expression during the early stages of recovery in young males and females	Danaher, J.; Stathis, C. G.; Wilson, R. A.; Moreno-Asso, A.; Wellard, R. M.; Cooke, M. B.	2020	Did not perform fiber type analysis
Maintaining a clinical weight loss after intensive lifestyle intervention is the key to cardiometabolic health	Dandanell, S.; Skovborg, C.; Praest, C. B.; Kristensen, K. B.; Nielsen, M. G.; Lionett, S.; Jorgensen, S. D.; Vigelso, A.; Dela, F.; Helge, J. W.	2017	Did not perform fiber type analysis
Training at high exercise intensity promotes qualitative adaptations of mitochondrial function in human skeletal muscle	Daussin, F. N.; Zoll, J.; Ponsot, E.; Dufour, S. P.; Doutreleau, S.; Lonsdorfer, E.; Ventura-Clapier, R.; Mettauer, B.; Piquard, F.; Geny, B.; Richard, R.	2008	Does not include males and females
Acute cellular and molecular responses and chronic adaptations to low-load blood flow restriction and high-load resistance exercise in trained individuals	Davids, C. J.; Naess, T. C.; Moen, M.; Cumming, K. T.; Horwath, O.; Psilander, N.; Ekblom, B.; Coombes, J. S.; Peake, J.; Raastad, T.; Roberts, L. A.	2021	Does not include male and female data
The Effect of Fava Bean (Vicia faba L.) Protein Ingestion on Myofibrillar Protein Synthesis at Rest and after Resistance Exercise in Healthy, Young Men and Women: A Randomised Control Trial	Davies, R. W.; Kozior, M.; Lynch, A. E.; Bass, J. J.; Atherton, P. J.; Smith, K.; Jakeman, P. M.	2022	Did not perform fiber type analysis
Acute Effects of Cheddar Cheese Consumption on Circulating Amino Acids and Human Skeletal Muscle	de Hart, Nmmp; Mahmassani, Z. S.; Reidy, P. T.; Kelley, J. J.; McKenzie, A. I.; Petrocelli, J. J.; Bridge, M. J.; Baird, L. M.; Bastian, E. D.; Ward, L. S.; Howard, M. T.; Drummond, M. J.	2021	Did not perform fiber type analysis
Skeletal Muscle Disorders: A Noncardiac Source of Cardiac Troponin T	de Lavallaz, J. D.; Prepoudis, A.; Wendebourg, M. J.; Kesenheimer, E.; Kyburz, D.; Daikeler, T.; Haaf, P.; Wanschitz, J.; Loscher, W. N.; Schreiner, B.; Katan, M.; Jung, H. H.; Maurer, B.; Hammerer-Lercher, A.; Mayr, A.; Gualandro, D. M.; Acket, A.; Puelacher, C.; Boeddinghaus, J.; Nestelberger, T.; Lopez-Ayala, P.; Glarner, N.; Shrestha, S.; Manka, R.; Gawinecka, J.; Piscuoglio, S.; Gallon, J.; Wiedemann, S.; Sinnreich, M.; Mueller, C.	2022	No healthy subjects or controls
Glut-4 and insulin-receptor binding and kinase-activity in trained human muscle	Dela, F.; Handberg, A.; Mikines, K. J.; Vinten, J.; Galbo, H.	1993	Does not include males and females

Excitation calcium release uncoupling in aged single human skeletal muscle fibers	Delbono, O.; Orouke, K. S.; Ettinger, W. H.	1995	Does not include male and female data
Effects of functional electric stimulation cycle ergometry training on lower limb musculature in acute SCI individuals	Demchak, T. J.; Linderman, J. K.; Mysiw, W. J.; Jackson, R.; Suun, J.; Devor, S. T.	2005	Does not include male and female data
Habitual physical activity in daily life correlates positively with markers for mitochondrial capacity	den Hoed, M.; Hesselink, M. K. C.; van Kranenburg, G. P. J.; Westerterp, K. R.	2008	Did not perform fiber type analysis
Aging alters gene expression of growth and remodeling factors in human skeletal muscle both at rest and in response to acute resistance exercise	Dennis, R. A.; Przybyla, B.; Gurley, C.; Kortebein, P. M.; Simpson, P.; Sullivan, D. H.; Peterson, C. A.	2008	Does not include males and females
Muscle expression of genes associated with inflammation, growth, and remodeling is strongly correlated in older adults with resistance training outcomes	Dennis, R. A.; Zhu, H. Y.; Kortebein, P. M.; Bush, H. M.; Harvey, J. F.; Sullivan, D. H.; Peterson, C. A.	2009	Did not perform fiber type analysis
Sex differences in acute translational repressor 4E-BP1 activity and sprint performance in response to repeated-sprint exercise in team sport athletes	Dent, J. R.; Edge, J. A.; Hawke, E.; McMahon, C.; Mundel, T.	2015	Did not perform fiber type analysis
No evidence of oxidant stress during high-intensity rowing training	Dernbach, A. R.; Sherman, W. M.; Simonsen, J. C.; Flowers, K. M.; Lamb, D. R.	1993	Did not perform fiber type analysis
Menstrual cycle phase and sex influence muscle glycogen utilization and glucose turnover during moderate-intensity endurance exercise	Devries, M. C.; Hamadeh, M. J.; Phillips, S. M.; Tarnopolsky, M. A.	2006	Did not perform fiber type analysis
IMCL area density, but not IMCL utilization, is higher in women during moderate-intensity endurance exercise, compared with men	Devries, M. C.; Lowther, S. A.; Glover, A. W.; Hamadeh, M. J.; Tarnopolsky, M. A.	2007	Did not perform fiber type analysis
Skeletal Muscle Inflammation Following Repeated Bouts of Lengthening Contractions in Humans	Deyhle, M. R.; Gier, A. M.; Evans, K. C.; Eggett, D. L.; Nelson, W. B.; Parcell, A. C.; Hyldahl, R. D.	2016	Did not perform fiber type analysis
Is the erythrocyte membrane fatty acid composition a valid index of skeletal muscle membrane fatty acid composition?	Di Marino, L.; Maffettone, A.; Cipriano, P.; Sacco, M.; Di Palma, R.; Amato, B.; Quarto, G.; Riccardi, G.; Rivellese, A. A.	2000	No healthy subjects or controls
Muscle and serum myostatin expression in type 1 diabetes	Dial, A. G.; Monaco, C. M. F.; Grafham, G. K.; Romanova, N.; Simpson, J. A.; Tarnopolsky, M. A.; Perry, C. G. R.; Kalaitzoglou, E.; Hawke, T. J.	2020	Did not perform fiber type analysis
Protein Supplementation Augments Muscle Fiber Hypertrophy but Does Not Modulate Satellite Cell Content During Prolonged Resistance-Type Exercise Training in Frail Elderly	Dirks, M. L.; Tieland, M.; Verdijk, L. B.; Losen, M.; Nilwik, R.; Mensink, M.; de Groot, Lcpgm; van Loon, L. J. C.	2017	Does not include male and female data
Physical activity unveils the relationship between mitochondrial energetics, muscle quality, and physical function in older adults	Distefano, G.; Standley, R. A.; Zhang, X. L.; Carnero, E. A.; Yi, F.; Cornnell, H. H.; Coen, P. M.	2018	Did not perform fiber type analysis
Simultaneous measurements of free amino acid patterns of plasma, muscle and erythrocytes in healthy human subjects	Divino, J. C.; Bergstrom, J.; Stehle, P.; Furst, P.	1997	Did not perform fiber type analysis

Mitochondrial respiratory chain function and content are preserved in the skeletal muscle of active very old men and women	Dodds, R. M.; Davies, K.; Granic, A.; Hollingsworth, K. G.; Warren, C.; Gorman, G.; Turnbull, D. M.; Sayer, A. A.	2018	Did not perform fiber type analysis
Advancing our understanding of skeletal muscle across the lifecourse: Protocol for the MASS_Lifecourse study and characteristics of the first 80 participants	Dodds, R. M.; Hurst, C.; Hillman, S. J.; Davies, K.; Roberts, L.; Aspray, T. J.; Granic, A.; Sayer, A. A.	2022	Did not perform fiber type analysis
Oxidative proteome alterations during skeletal muscle ageing	dos Santos, S. L.; Baraibar, M. A.; Lundberg, S.; Eeg-Olofsson, O.; Larsson, L.; Friguet, B.	2015	No healthy subjects or controls
Skeletal muscle enzymes as predictors of 24-h energy metabolism in reduced-obese persons	Doucet, E.; Tremblay, A.; Simoneau, J. A.; Joanisse, D. R.	2003	Did not perform fiber type analysis
Adaptation of the diaphragm and the vastus lateralis in mild-to-moderate COPD	Doucet, M.; Debigare, R.; Joanisse, D. R.; Cote, C.; LeBlanc, P.; Gregoire, J.; Deslauriers, J.; Vaillancourt, R.; Maltais, F.	2004	Does not include male and female data
Resistance exercise increases AMPK activity and reduces 4E-BP1 phosphorylation and protein synthesis in human skeletal muscle	Dreyer, H. C.; Fujita, S.; Cadenas, J. G.; Chinkes, D. L.; Volpi, E.; Rasmussen, B. B.	2006	Did not perform fiber type analysis
Resistance exercise increases leg muscle protein synthesis and mTOR signalling independent of sex	Dreyer, H. C.; Fujita, S.; Glynn, E. L.; Drummond, M. J.; Volpi, E.; Rasmussen, B. B.	2010	Did not perform fiber type analysis
Essential Amino Acids Increase MicroRNA-499,-208b, and-23a and Downregulate Myostatin and Myocyte Enhancer Factor 2C mRNA Expression in Human Skeletal Muscle	Drummond, M. J.; Glynn, E. L.; Fry, C. S.; Dhanani, S.; Volpi, E.; Rasmussen, B. B.	2009	Did not perform fiber type analysis
An increase in essential amino acid availability upregulates amino acid transporter expression in human skeletal muscle	Drummond, M. J.; Glynn, E. L.; Fry, C. S.; Timmerman, K. L.; Volpi, E.; Rasmussen, B. B.	2010	Did not perform fiber type analysis
Exercise-induced alterations in intramyocellular lipids and insulin resistance: the athlete's paradox revisited	Dube, J. J.; Amati, F.; Stefanovic-Racic, M.; Toledo, F. G. S.; Sauers, S. E.; Goodpaster, B. H.	2008	Does not include male and female data
Muscle Characteristics and Substrate Energetics in Lifelong Endurance Athletes	Dube, J. J.; Broskey, N. T.; Despines, A. A.; Stefanovic-Racic, M.; Toledo, F. G. S.; Goodpaster, B. H.; Amati, F.	2016	Does not include male and female data
Skeletal muscle fibre type and capillary density in college-aged blacks and whites	Duey, W. J.; Bassett, D. R.; Torok, D. J.; Howley, E. T.; Bond, V.; Mancuso, P.; Trudell, R.	1997	Does not include males and females
Effect of acute alcohol ingestion on resistance exercise-induced mtorc1 signaling in human muscle	Duplanty, A. A.; Budnar, R. G.; Luk, H. Y.; Levitt, D. E.; Hill, D. W.; McFarlin, B. K.; Huggett, D. B.; Vingren, J. L.	2017	Did not perform fiber type analysis
Leg glucose and protein metabolism during an acute bout of resistance exercise in humans	Durham, W. J.; Miller, S. L.; Yeckel, C. W.; Chinkes, D. L.; Tipton, K. D.; Rasmussen, B. B.; Wolfe, R. R.	2004	Did not perform fiber type analysis
Regulation of muscle glycogen phosphorylase activity during intense aerobic cycling with elevated FFA	Dyck, D. J.; Peters, S. J.; Wendling, P. S.; Chesley, A.; Hultman, E.; Spriet, L. L.	1996	Does not include males and females
MRI characterization of skeletal muscle size and fatty infiltration in long--term trained and untrained individuals	Emanuelsson, E. B.; Berry, D. B.; Reitzner, S. M.; Arif, M.; Mardinoglu, A.; Gustafsson, T.; Ward, S. R.; Sundberg, C. J.; Chapman, M. A.	2022	Did not perform fiber type analysis

Sex-Specific Aspects of Skeletal Muscle Metabolism in the Clinical Context of Intensive Care Unit-Acquired Weakness	Engelhardt, L. J.; Grunow, J. J.; Wollersheim, T.; Carbon, N. M.; Balzer, F.; Spranger, J.; Weber-Carstens, S.	2022	No healthy subjects or controls
Resistance training performed at distinct angular velocities elicits velocity-specific alterations in muscle strength and mobility status in older adults	Englund, D. A.; Sharp, R. L.; Selsby, J. T.; Ganesan, S. S.; Franke, W. D.	2017	Did not perform fiber type analysis
Skeletal muscle morphology in power-lifters with and without anabolic steroids	Eriksson, A.; Kadi, F.; Malm, C.; Thornell, L. E.	2005	Does not report sex of subjects
Metabolic and morphological profile in skeletal muscle of healthy boys and girls	Esbjornsson, M.; Norman, B.; Dahlstrom, M.; Gierup, J.; Jansson, E.	2022	Only in children (0-17 years)
Uncoupling protein-3 gene expression: reduced skeletal muscle mRNA in obese humans during pronounced weight loss	Esterbauer, H.; Oberkofler, H.; Dallinger, G.; Breban, D.; Hell, E.; Krempler, F.; Patsch, W.	1999	Did not perform fiber type analysis
Effects of Fortetropin on the Rate of Muscle Protein Synthesis in Older Men and Women: A Randomized, Double-Blinded, Placebo-Controlled Study	Evans, W.; Shankaran, M.; Nyangau, E.; Field, T.; Mohammed, H.; Wolfe, R.; Schutzler, S.; Hellerstein, M.	2021	Did not perform fiber type analysis
Effect of training intensity on muscle lactate transporters and lactate threshold of cross-country skiers	Evertsen, F.; Medbo, J. I.; Bonen, A.	2001	Does not include male and female data
Effect of training on the activity of five muscle enzymes studied on elite cross-country skiers	Evertsen, F.; Medbo, J. I.; Jebens, E.; Gjovaag, T. F.	1999	Does not include male and female data
Hard training for 5 mo increases Na+-K+ pump concentration in skeletal muscle of cross-country skiers	Evertsen, F.; Medbo, J. I.; Jebens, E.; Nicolaysen, K.	1997	Did not perform fiber type analysis
Reduction of glutamine-synthetase messenger-rna in hypertrophied skeletal-muscle	Falduto, M. T.; Young, A. P.; Smyrniotis, G.; Hickson, R. C.	1992	Animal study
Gender difference in limb-girdle muscular dystrophy: a muscle fiber morphometric study in 101 patients	Fanin, M.; Nascimbeni, A. C.; Angelini, C.	2014	Did not perform fiber type analysis
Age and sex influence on oxidative damage and functional status in human skeletal muscle	Fano, G.; Mecocci, P.; Vecchiet, J.; Belia, S.; Fulle, S.; Polidori, M. C.; Felzani, G.; Senin, U.; Vecchiet, L.; Beal, M. F.	2001	Did not perform fiber type analysis
High Intensity Training May Reverse the Fiber Type Specific Decline in Myogenic Stem Cells in Multiple Sclerosis Patients	Farup, J.; Dalgas, U.; Keytsman, C.; Eijnde, B. O.; Wens, I.	2016	Does not report sex of subjects
Similar changes in muscle fiber phenotype with differentiated consequences for rate of force development: Endurance versus resistance training	Farup, J.; Sorensen, H.; Kjolhede, T.	2014	Does not report sex of subjects
Tissue-Specific Responses of Lipoprotein Lipase to Dietary Macronutrient Composition as a Predictor of Weight Gain Over 4 Years	Ferland, A.; Chateau-Degat, M. L.; Hernandez, T. L.; Eckel, R. H.	2012	Did not perform fiber type analysis
Flywheel Resistance Exercise to Maintain Muscle Oxidative Potential During Unloading	Fernandez-Gonzalo, R.; Irimia, J. M.; Cusso, R.; Gustafsson, T.; Linne, A.; Tesch, P. A.	2014	Did not perform fiber type analysis
HYPOPHOSPHATEMIA AND PHOSPHORUS DEPLETION IN RESPIRATORY AND PERIPHERAL MUSCLES OF PATIENTS WITH RESPIRATORY-FAILURE DUE TO COPD	Fiaccadori, E.; Coffrini, E.; Fracchia, C.; Rampulla, C.; Montagna, T.; Borghetti, A.	1994	Did not perform fiber type analysis

THE ROLE OF PROGRESSIVE RESISTANCE TRAINING AND NUTRITION IN THE PRESERVATION OF LEAN BODY-MASS IN THE ELDERLY	Fielding, R. A.	1995	Review (ex: systematic review/meta-analysis)
Genetic and epigenetic regulation of skeletal muscle ribosome biogenesis with exercise	Figueiredo, V. C.; Wen, Y.; Alkner, B.; Fernandez-Gonzalo, R.; Norrbom, J.; Vechetti, I. J.; Valentino, T.; Mobley, C. B.; Zentner, G. E.; Peterson, C. A.; McCarthy, J. J.; Murach, K. A.; von Walden, F.	2021	Did not perform fiber type analysis
Clinical significance and neuropathology of primary MADD in C34-T and G468-T mutations of the AMPD1 gene	Fischer, S.; Drenckhahn, C.; Wolf, C.; Eschrich, K.; Kellermann, S.; Froster, U. G.; Schober, R.	2005	No healthy subjects or controls
Sex and race contribute to variation in mitochondrial function and insulin sensitivity	Fisher, G.; Tay, J.; Warren, J. L.; Garvey, W. T.; Yarar-Fisher, C.; Gower, B. A.	2021	No cross-sectional area data
Mitochondrial Respiratory Capacity and Content Are Normal in Young Insulin-Resistant Obese Humans	Fisher-Wellman, K. H.; Weber, T. M.; Cathey, B. L.; Brophy, P. M.; Gilliam, L. A. A.; Kane, C. L.; Maples, J. M.; Gavin, T. P.; Houmard, J. A.; Neufer, P. D.	2014	Did not perform fiber type analysis
ATX II, a sodium channel toxin, sensitizes skeletal muscle to halothane, caffeine, and ryanodine	Fletcher, J. E.; Adnet, P. J.; Reyford, H.; Wieland, S. J.; Stewart, S. L.; Rosenberg, H.	1999	Did not perform fiber type analysis
Resting membrane potential of skeletal muscle calculated from plasma and muscle electrolyte and water contents	Forsberg, A. M.; Bergstrom, J.; Lindholm, B.; Hultman, E.	1997	Did not perform fiber type analysis
MUSCLE COMPOSITION IN RELATION TO AGE AND SEX	Forsberg, A. M.; Nilsson, E.; Werneman, J.; Bergstrom, J.; Hultman, E.	1991	Did not perform fiber type analysis
Sex-Based Differences in the Myogenic Response and Inflammatory Gene Expression Following Eccentric Contractions in Humans	Fortino, S. A.; Wageh, M.; Pontello, R.; McGlory, C.; Kumbhare, D.; Phillips, S. M.; Parise, G.	2022	No cross-sectional area data
Low-dose aspirin and COX inhibition in human skeletal muscle	Fountain, W. A.; Naruse, M.; Claiborne, A.; Stroh, A. M.; Gries, K. J.; Jones, A. M.; Minchev, K.; Lester, B. E.; Raue, U.; Trappe, S.; Trappe, T. A.	2020	Did not perform fiber type analysis
Sex differences in semitendinosus muscle fiber-type composition	Fournier, G.; Bernard, C.; Cievet-Bonfils, M.; Kenney, R.; Pingon, M.; Sappey-Marinier, E.; Chazaud, B.; Gondin, J.; Servien, E.	2022	No healthy subjects or controls
Strength training improves muscle aerobic capacity and glucose tolerance in elderly	Frank, P.; Andersson, E.; Ponten, M.; Ekblom, B.; Ekblom, M.; Sahlin, K.	2016	Does not include male and female data
Acute exercise reverses starvation-mediated insulin resistance in humans	Frank, P.; Katz, A.; Andersson, E.; Sahlin, K.	2013	Did not perform fiber type analysis
EFFECTS OF STEP EXERCISE ON MUSCLE DAMAGE AND MUSCLE CA(2+) CONTENT IN MEN AND WOMEN	Fredsted, A.; Clausen, T.; Overgaard, K.	2008	Did not perform fiber type analysis
Effect of training on muscle strength and motor function in the elderly	Frischknecht, R.	1998	Review (ex: systematic review/meta-analysis)

New Nordic Diet-Induced Weight Loss Is Accompanied by Changes in Metabolism and AMPK Signaling in Adipose Tissue	Fritzen, A. M.; Lundsgaard, A. M.; Jordy, A. B.; Poulsen, S. K.; Stender, S.; Pilegaard, H.; Astrup, A.; Larsen, T. M.; Wojtaszewski, J. F. P.; Richter, E. A.; Kiens, B.	2015	Did not perform fiber type analysis
Preserved Capacity for Adaptations in Strength and Muscle Regulatory Factors in Elderly in Response to Resistance Exercise Training and Deconditioning	Fritzen, A. M.; Thogersen, F. D.; Qadri, K. A. N.; Krag, T.; Sveen, M. L.; Vissing, J.; Jeppesen, T. D.	2020	No cross-sectional area data
Muscle fiber size and function in elderly humans: a longitudinal study	Frontera, W. R.; Reid, K. F.; Phillips, E. M.; Krivickas, L. S.; Hughes, V. A.; Roubenoff, R.; Fielding, R. A.	2008	Does not include male and female data
Muscle fiber characteristics of competitive power lifters	Fry, A. C.; Webber, J. M.; Weiss, L. W.; Harber, M. P.; Vaczi, M.; Pattison, N. A.	2003	Does not include males and females
Skeletal Muscle Autophagy and Protein Breakdown Following Resistance Exercise are Similar in Younger and Older Adults	Fry, C. S.; Drummond, M. J.; Glynn, E. L.; Dickinson, J. M.; Gundermann, D. M.; Timmerman, K. L.; Walker, D. K.; Volpi, E.; Rasmussen, B. B.	2013	Did not perform fiber type analysis
Fibre type-specific satellite cell response to aerobic training in sedentary adults	Fry, C. S.; Noehren, B.; Mula, J.; Ubele, M. F.; Westgate, P. M.; Kern, P. A.; Peterson, C. A.	2014	Does not include male and female data
Exercise, sex, menstrual cycle phase, and 17 beta-estradiol influence metabolism-related genes in human skeletal muscle	Fu, M. H. H.; Maher, A. C.; Hamadeh, M. J.; Ye, C. H.; Tarnopolsky, M. A.	2009	Did not perform fiber type analysist
Skeletal muscle signaling response to sprint exercise in men and women	Fuentes, T.; Guerra, B.; Ponce-Gonzalez, J. G.; Morales-Alamo, D.; Guadalupe-Grau, A.; Olmedillas, H.; Rodriguez-Garcia, L.; Feijoo, D.; De Pablos-Velasco, P.; Fernandez-Perez, L.; Santana, A.; Calbet, J. A. L.	2012	Did not perform fiber type analysis
Isoinertial and Isokinetic Sprints: Muscle Signalling	Fuentes, T.; Ponce-Gonzalez, J. G.; Morales-Alamo, D.; de Torres-Peralta, R.; Santana, A.; De Pablos-Velasco, P.; Olmedillas, H.; Guadalupe-Grau, A.; Rodriguez-Garcia, L.; Serrano-Sanchez, J. A.; Guerra, B.; Calbet, J. A. L.	2013	Does not include male and female data
Basal muscle intracellular amino acid kinetics in women and men	Fujita, S.; Rasmussen, B. B.; Bell, J. A.; Cadenas, J. G.; Volpi, E.	2007	Did not perform fiber type analysis
HUMAN MYASTHENIA-GRAVIS THYMIC MYOID CELLS - DENOVO IMMUNOHISTOCHEMICAL AND INTRACELLULAR ELECTROPHYSIOLOGICAL STUDIES	Furuya, A.; Kobayashi, T.; Kameda, N.; Tsukagoshi, H.	1991	No healthy subjects or controls
Human skeletal muscle fiber type specific protein content	Galpin, A. J.; Raue, U.; Jemiolo, B.; Trappe, T. A.; Harber, M. P.; Minchev, K.; Trappe, S.	2012	Case studies
Trade-Offs (and Constraints) in Organismal Biology	Garland, T.; Downs, C. J.; Ives, A. R.	2022	Review (ex: systematic review/meta-analysis)
Divergent skeletal muscle mitochondrial phenotype between male and female patients with chronic heart failure	Garnham, J. O.; Roberts, L. D.; Caspi, T.; Al-Owais, M. M.; Bullock, M.; Swoboda, P. P.; Koshy, A.; Gierula, J.; Paton, M. F.; Cubbon,	2020	Does not include male and female data

	R. M.; Kearney, M. T.; Bowen, T. S.; Witte, K. K.		
Direct evidence of fiber type-dependent GLUT-4 expression in human skeletal muscle	Gaster, M.; Poulsen, P.; Handberg, A.; Schroder, H. D.; Beck-Nielsen, H.	2000	Does not include male and female data
ELECTRICAL STIMULATION-INDUCED CHANGES IN SKELETAL-MUSCLE ENZYMES OF MEN AND WOMEN	Gauthier, J. M.; Theriault, R.; Theriault, G.; Gelinas, Y.; Simoneau, J. A.	1992	Did not perform fiber type analysis
Altered skeletal muscle (mitochondrial) properties in patients with mitochondrial DNA single deletion myopathy	Gehrig, S. M.; Mihaylova, V.; Frese, S.; Mueller, S. M.; Ligon-Auer, M.; Spengler, C. M.; Petersen, J. A.; Lundby, C.; Jung, H. H.	2016	Does not include male and female data
The human muscle proteome in aging	Gelfi, C.; Vigano, A.; Ripamonti, M.; Pontoglio, A.; Begum, S.; Pellegrino, M. A.; Grassi, B.; Bottinelli, R.; Wait, R.; Cerretelli, P.	2006	Does not report sex of subjects
Sera from young and older humans equally sustain proliferation and differentiation of human myoblasts	George, T.; Velloso, C. P.; Alsharidah, M.; Lazarus, N. R.; Harridge, S. D. R.	2010	Did not perform fiber type analysis
Peptide YY (PYY) Is Expressed in Human Skeletal Muscle Tissue and Expanding Human Muscle Progenitor Cells	Gheller, B. J.; Blum, J. E.; Merritt, E. K.; Cummings, B. P.; Thalacker-Mercer, A. E.	2019	Does not report sex of subjects
Rapidly elevated levels of PGC-1 alpha-b protein in human skeletal muscle after exercise: exploring regulatory factors in a randomized controlled trial	Gidlund, E. K.; Ydfors, M.; Appel, S.; Rundqvist, H.; Sundberg, C. J.; Norrbom, J.	2015	Did not perform fiber type analysis
Interrupting prolonged sitting with repeated chair stands or short walks reduces postprandial insulinemia in healthy adults	Gillen, J. B.; Estafanos, S.; Williamson, E.; Hodson, N.; Malowany, J. M.; Kumbhare, D.; Moore, D. R.	2021	Did not perform fiber type analysis D229
Three Minutes of All-Out Intermittent Exercise per Week Increases Skeletal Muscle Oxidative Capacity and Improves Cardiometabolic Health	Gillen, J. B.; Percival, M. E.; Skelly, L. E.; Martin, B. J.; Tan, R. B.; Tarnopolsky, M. A.; Gibala, M. J.	2014	Did not perform fiber type analysis
Effect of training with different intensities and volumes on muscle fibre enzyme activity and cross sectional area in the m. triceps brachii	Gjovaag, T. F.; Dahl, H. A.	2008	Does not include male and female data
RUNNING CAPACITY FROM ADOLESCENCE TO ADULTHOOD - RELATIONSHIP TO PHYSICAL CHARACTERISTICS	Glenmark, B.; Hedberg, G.; Jansson, E.	1993	Same subjects as another study
Excess Leucine Intake Enhances Muscle Anabolic Signaling but Not Net Protein Anabolism in Young Men and Women	Glynn, E. L.; Fry, C. S.; Drummond, M. J.; Timmerman, K. L.; Dhanani, S.; Volpi, E.; Rasmussen, B. B.	2010	Did not perform fiber type analysis
Addition of Carbohydrate or Alanine to an Essential Amino Acid Mixture Does Not Enhance Human Skeletal Muscle Protein Anabolism	Glynn, E. L.; Fry, C. S.; Timmerman, K. L.; Drummond, M. J.; Volpi, E.; Rasmussen, B. B.	2013	Did not perform fiber type analysis
Chronic dietary exposure to branched chain amino acids impairs glucose disposal in vegans but not in omnivores	Gojda, J.; Rossmeislova, L.; Strakova, R.; Tumova, J.; Elkalaf, M.; Jacek, M.; Tuma, P.; Potockova, J.; Krauzova, E.; Waldauf, P.; Trnka, J.; Stich, V.; Andel, M.	2017	Did not perform fiber type analysis
Skeletal muscle ex vivo mitochondrial respiration parallels decline in vivo oxidative capacity, cardiorespiratory fitness, and muscle strength: The Baltimore Longitudinal Study of Aging	Gonzalez-Freire, M.; Scalzo, P.; D'Agostino, J.; Moore, Z. A.; Diaz-Ruiz, A.; Fabbri, E.; Zane, A.; Chen, B.; Becker, K. G.; Lehrmann, E.; Zukley, L.; Chia, C. W.; Tanaka, T.; Coen, P. M.; Bernier, M.; de Cabo, R.; Ferrucci, L.	2018	Did not perform fiber type analysis

Skeletal muscle attenuation determined by computed tomography is associated with skeletal muscle lipid content	Goodpaster, B. H.; Kelley, D. E.; Thaete, F. L.; He, J.; Ross, R.	2000	Did not perform fiber type analysis
Intramuscular lipid content is increased in obesity and decreased by weight loss	Goodpaster, B. H.; Theriault, R.; Watkins, S. C.; Kelley, D. E.	2000	Does not include male and female data
Altered Skeletal Muscle Fatty Acid Handling in Subjects with Impaired Glucose Tolerance as Compared to Impaired Fasting Glucose	Goossens, G. H.; Moors, C. C. M.; Jocken, J. W. E.; van der Zijl, N. J.; Jans, A.; Konings, E.; Diamant, M.; Blaak, E. E.	2016	Did not perform fiber type analysis
Resistance exercise training influences skeletal muscle immune activation: a microarray analysis	Gordon, P. M.; Liu, D. M.; Sartor, M. A.; IglayReger, H. B.; Pistilli, E. E.; Gutmann, L.; Nader, G. A.; Hoffman, E. P.	2012	Did not perform fiber type analysis
Reference values for vastus lateralis fiber size and type in healthy subjects over 40 years old: a systematic review and metaanalysis	Gouzi, F.; Maury, J.; Molinari, N.; Pomies, P.; Mercier, J.; Prefaut, C.; Hayot, M.	2013	Review (ex: systematic review/meta-analysis)
Human and rat skeletal muscle adaptations to spinal cord injury	Gregory, C. M.; Vandenborne, K.; Castro, M. J.; Dudley, G. A.	2003	Does not report sex of subjects
Resistance exercise decreases skeletal muscle tumor necrosis factor alpha in frail elderly humans	Greiwe, J. S.; Cheng, B.; Rubin, D. C.; Yarasheski, K. E.; Semenkovich, C. F.	2001	Did not perform fiber type analysis
Cardiovascular and skeletal muscle health with lifelong exercise	Gries, K. J.; Raue, U.; Perkins, R. K.; Lavin, K. M.; Overstreet, B. S.; D'Acquisto, L. J.; Graham, B.; Finch, W. H.; Kaminsky, L. A.; Trappe, T. A.; Trappe, S.	2018	Did not perform fiber type analysis
Three DNA Polymorphisms Previously Identified as Markers for Handgrip Strength Are Associated With Strength in Weightlifters and Muscle Fiber Hypertrophy	Grishina, E. E.; Zmijewski, P.; Semenova, E. A.; Cieszczyk, P.; Huminska-Lisowska, K.; Michalowska-Sawczyn, M.; Maculewicz, E.; Crewther, B.; Orysiak, J.; Kostryukova, E. S.; Kulemin, N. A.; Borisov, O. V.; Khabibova, S. A.; Larin, A. K.; Pavlenko, A. V.; Lyubaeva, E. V.; Popov, D. V.; Lysenko, E. A.; Vepkhvadze, T. F.; Lednev, E. M.; Bondareva, E. A.; Erskine, R. M.; Generozov, E. V.; Ahmetov,, II	2019	Does not include male and female data
Single muscle fibre contractile function with ageing	Grosicki, G. J.; Zepeda, C. S.; Sundberg, C. W.		Review (ex: systematic review/meta-analysis)
Gender Dimorphism in Skeletal Muscle Leptin Receptors, Serum Leptin and Insulin Sensitivity	Guerra, B.; Fuentes, T.; Delgado-Guerra, S.; Guadalupe-Grau, A.; Olmedillas, H.; Santana, A.; Ponce-Gonzalez, J. G.; Dorado, C.; Calbet, J. A. L.	2008	Did not perform fiber type analysis
Are Genome-Wide Association Study Identified Single-Nucleotide Polymorphisms Associated With Sprint Athletic Status? A Replication Study With 3 Different Cohorts	Guilherme, Jplf; Semenova, E. A.; Zempo, H.; Martins, G. L.; Lancha, A. H.; Miyamoto-Mikami, E.; Kumagai, H.; Tobina, T.; Shiose, K.; Kakigi, R.; Tsuzuki, T.; Ichinoseki-Sekine, N.; Kobayashi, H.; Naito, H.; Borisov, O. V.; Kostryukova, E. S.; Kulemin, N. A.; Larin, A. K.; Generozov, E. V.; Fuku, N.; Ahmetov,, II	2021	Does not include male and female data

Muscle Fiber Type Composition, Fiber Diameter, Capillary Density in Temporalis and Masseter Muscles and Correlation with Bite Force	Guimaraes, T. B.; Ferreira, M. B.; Wakamatsu, A.; Oliveira, S. R.; Guimaraes, A. S.; Galdames, I. S.; Marie, S. N.	2013	Autopsies
EFFECT OF 7-10 DAYS OF CYCLE ERGOMETER EXERCISE ON SKELETAL-MUSCLE GLUT-4 PROTEIN-CONTENT	Gulve, E. A.; Spina, R. J.	1995	Did not perform fiber type analysis
Relationship between in vivo muscle force at different speeds of isokinetic movements and myosin isoform expression in men and women	Gur, H.; Gransberg, L.; vanDyke, D.; Knutsson, E.; Larsson, L.	2003	Only in children (0-17 years)
Pretranslational markers of contractile protein expression in human skeletal muscle: effect of limb unloading plus resistance exercise	Haddad, F.; Baldwin, K. M.; Tesch, P. A.	2005	Did not perform fiber type analysis
Daily heat treatment maintains mitochondrial function and attenuates atrophy in human skeletal muscle subjected to immobilization	Hafen, P. S.; Abbott, K.; Bowden, J.; Lopiano, R.; Hancock, C. R.; Hyldahl, R. D.	2019	Does not include male and female data
Sex-Based Differences in Skeletal Muscle Kinetics and Fiber-Type Composition	Haizlip, K. M.; Harrison, B. C.; Leinwand, L. A.	2015	Review (ex: systematic review/meta-analysis)
Native Whey Induces Similar Post Exercise Muscle Anabolic Responses as Regular Whey, Despite Greater Leucinemia, in Elderly Individuals	Hamarsland, H.; Aas, S. N.; Nordengen, A. L.; Holte, K.; Garthe, I.; Paulsen, G.; Cotter, M.; Borsheim, E.; Benestad, H. B.; Raastad, T.	2019	Did not perform fiber type analysis
Native Whey Induces Similar Adaptation to Strength Training as Milk, despite Higher Levels of Leucine, in Elderly Individuals	Hamarsland, H.; Johansen, M. K.; Seeberg, F.; Brochmann, M.; Garthe, I.; Benestad, H. B.; Raastad, T.	2019	Does not include male and female data
Native whey protein with high levels of leucine results in similar post-exercise muscular anabolic responses as regular whey protein: a randomized controlled trial	Hamarsland, H.; Nordengen, A. L.; Aas, S. N.; Holte, K.; Garthe, I.; Paulsen, G.; Cotter, M.; Borsheim, E.; Benestad, H. B.; Raastad, T.	2017	Did not perform fiber type analysis
Effects of eccentric cycling exercise on IGF-I splice variant expression in the muscles of young and elderly people	Hameed, M.; Toft, A. D.; Pedersen, B. K.; Harridge, S. D. R.; Goldspink, G.	2008	Does not include males and females
Muscle 'regenerative potential' determines physical recovery following total knee replacement	Hamilton, D. F.; McLeish, J. A.; Gaston, P.; Simpson, Ahrw	2013	Did not perform fiber type analysis
Benefits of higher resistance-training volume are related to ribosome biogenesis	Hammarstrom, D.; Ofsteng, S.; Koll, L.; Hanestadhaugen, M.; Hollan, I.; Apro, W.; Whist, J. E.; Blomstrand, E.; Ronnestad, B. R.; Ellefsen, S.	2020	Does not include male and female data
Reserve capacity for ATP consumption during isometric contraction in human skeletal muscle fibers	Han, Y. S.; Proctor, D. N.; Geiger, P. C.; Sieck, G. C.	2001	Does not include male and female data
Effects of alternating blood flow restricted training and heavy-load resistance training on myofiber morphology and mechanical muscle function	Hansen, S. K.; Ratzer, J.; Nielsen, J. L.; Suetta, C.; Karlsen, A.; Kvorning, T.; Frandsen, U.; Aagaard, P.	2020	Does not include male and female data
Exogenous carbohydrate spares muscle glycogen in men and women during 10 h of exercise	Harger-Domitrovich, S. G.; McClaughry, A. E.; Gaskill, S. E.; Ruby, B. C.	2007	Did not perform fiber type analysis

Sprint Training Increases Muscle Oxidative Metabolism During High-Intensity Exercise in Patients With Type 1 Diabetes	Harmer, A. R.; Chisholm, D. J.; McKenna, M. J.; Hunter, S. K.; Ruell, P. A.; Naylor, J. M.; Maxwell, L. J.; Flack, J. R.	2008	Did not perform fiber type analysis
Effects of type 1 diabetes, sprint training and sex on skeletal muscle sarcoplasmic reticulum Ca2+uptake and Ca2+-ATPase activity	Harmer, A. R.; Ruell, P. A.; Hunter, S. K.; McKenna, M. J.; Thom, J. M.; Chisholm, D. J.; Flack, J. R.	2014	Did not perform fiber type analysis
Gender-Specific Differences in Skeletal Muscle 11 beta-HSD1 Expression Across Healthy Aging	Hassan-Smith, Z. K.; Morgan, S. A.; Sherlock, M.; Hughes, B.; Taylor, A. E.; Lavery, G. G.; Tomlinson, J. W.; Stewart, P. M.	2015	Did not perform fiber type analysis
Resistance exercise acutely increases MHC and mixed muscle protein synthesis rates in 78-84 and 23-32 yr olds	Hasten, D. L.; Pak-Loduca, J.; Obert, K. A.; Yarasheski, K. E.	2000	Did not perform fiber type analysis
Desaturation of excess intramyocellular triacylglycerol in obesity: implications for glycemic control	Haugaard, S. B.; Madsbad, S.; Mu, H.; Vaag, A.	2010	Did not perform fiber type analysis
Intramyocellular triglyceride content in man, influence of sex, obesity and glycaemic control	Haugaard, S. B.; Mu, H. L.; Vaag, A.; Madsbad, S.	2009	Did not perform fiber type analysis
Desaturation of skeletal muscle structural and depot lipids in obese individuals during a very-low-calorie diet intervention	Haugaard, S. B.; Vaag, A.; Hoy, C. E.; Madsbad, S.	2007	Did not perform fiber type analysis
Sex and muscle structural lipids in obese subjects - an impact on insulin action?	Haugaard, S. B.; Vaag, A.; Hoy, C. E.; Madsbad, S.	2008	Did not perform fiber type analysis
A Critical Evaluation of the Biological Construct Skeletal Muscle Hypertrophy: Size Matters but So Does the Measurement	Haun, C. T.; Vann, C. G.; Roberts, B. M.; Vigotsky, A. D.; Schoenfeld, B. J.; Roberts, M. A.	2019	Review (ex: systematic review/meta-analysis)
Collagen, cross-linking, and advanced glycation end products in aging human skeletal muscle	Haus, J. M.; Carrithers, J. A.; Trappe, S. W.; Trappe, T. A.	2007	Does not include male and female data
Optimal velocity for maximal power production in non-isokinetic cycling is related to muscle fibre type composition	Hautier, C. A.; Linossier, M. T.; Belli, A.; Lacour, J. R.; Arsac, L. M.	1996	Does not include male and female data
Effects of weight loss and physical activity on muscle lipid content and droplet size	He, J.; Goodpaster, B. H.; Kelley, D. E.	2004	Does not include male and female data
Glucose tolerance and skeletal muscle gene expression in response to alternate day fasting	Heilbronn, L. K.; Civitarese, A. E.; Bogacka, I.; Smith, S. R.; Hulver, M.; Ravussin, E.	2005	Did not perform fiber type analysis
Interleukin-6 release is higher across arm than leg muscles during whole-body exercise	Helge, J. W.; Klein, D. K.; Andersen, T. M.; van Hall, G.; Calbet, J.; Boushel, R.; Saltin, B.	2011	Did not perform fiber type analysis
Increased fat oxidation and regulation of metabolic genes with ultraendurance exercise	Helge, J. W.; Rehrer, N. J.; Pilegaard, H.; Manning, P.; Lucas, S. J. E.; Gerrard, D. F.; Cotter, J. D.	2007	Does not include male and female data
Improved glucose tolerance after intensive life style intervention occurs without changes in muscle ceramide or triacylglycerol in morbidly obese subjects	Helge, J. W.; Stallknecht, B.; Drachmann, T.; Hellgren, L. I.; Jimenez-Jimenez, R.; Andersen, J. L.; Richelsen, B.; Bruun, J. M.	2011	No healthy subjects or controls

MUSCLE FIBER TYPE PROPORTION AND SIZE IS NOT ALTERED IN MCARDLE DISEASE	Henning, F.; Cunninghame, C. A.; Martin, M. A.; Rubio, J. C.; Arenas, J.; Lucia, A.; Hernandez-Lain, A.; Kohn, T. A.	2017	Review (ex: systematic review/meta-analysis)
Skeletal muscle mitochondrial DNA copy number and mitochondrial DNA deletion mutation frequency as predictors of physical performance in older men and women	Herbst, A.; Prior, S. J.; Lee, C. C.; Aiken, J. M.; McKenzie, D.; Hoang, A.; Liu, N. J.; Chen, X. W.; Xun, P. C.; Allison, D. B.; Wanagat, J.	2021	Did not perform fiber type analysis
SUCCESSIVE TIME COURSES OF STRENGTH DEVELOPMENT AND STEROID-HORMONE RESPONSES TO HEAVY-RESISTANCE TRAINING	Hickson, R. C.; Hidaka, K.; Foster, C.; Falduto, M. T.; Chatterton, R. T.	1994	Does not include male and female data
Short-term intense exercise training reduces stress markers and alters the transcriptional response to exercise in skeletal muscle	Hinkley, J. M.; Konopka, A. R.; Suer, M. K.; Harber, M. P.	2017	Did not perform fiber type analysis
Exercise training increases electron and substrate shuttling proteins in muscle of overweight men and women with the metabolic syndrome	Hittel, D. S.; Kraus, W. E.; Tanner, C. J.; Houmard, J. A.; Hoffman, E. P.	2005	Did not perform fiber type analysis
Behavioral, metabolic, and molecular correlates of lower insulin sensitivity in Mexican-Americans	Ho, R. C.; Davy, K. P.; Hickey, M. S.; Summers, S. A.; Melby, C. L.	2002	Did not perform fiber type analysis
Higher intramuscular triacylglycerol in women does not impair insulin sensitivity and proximal insulin signaling	Hoeg, L.; Roepstorff, C.; Thiele, M.; Richter, E. A.; Wojtaszewski, J. F. P.; Kiens, B.	2009	Same subjects as another study
Lipid-Induced Insulin Resistance Affects Women Less Than Men and Is Not Accompanied by Inflammation or Impaired Proximal Insulin Signaling	Hoeg, L. D.; Sjoberg, K. A.; Jeppesen, J.; Jensen, T. E.; Frosig, C.; Birk, J. B.; Bisiani, B.; Hiscock, N.; Pilegaard, H.; Wojtaszewski, J. F. P.; Richter, E. A.; Kiens, B.	2011	Did not perform fiber type analysis
Response of Mitochondrial Respiration in Adipose Tissue and Muscle to 8 Weeks of Endurance Exercise in Obese Subjects	Hoffmann, C.; Schneeweiss, P.; Randrianarisoa, E.; Schnauder, G.; Kappler, L.; Machann, J.; Schick, F.; Fritsche, A.; Heni, M.; Birkenfeld, A.; Niess, A. M.; Haring, H. U.; Weigert, C.; Moller, A.	2020	Did not perform fiber type analysis
Low-Volume Interval Training Improves Muscle Oxidative Capacity in Sedentary Adults	Hood, M. S.; Little, J. P.; Tarnopolsky, M. A.; Myslik, F.; Gibala, M. J.	2011	Did not perform fiber type analysis
The Muscle Protein Synthetic Response to Whey Protein Ingestion Is Greater in Middle-Aged Women Compared With Men	Horstman, A. M. H.; Kouw, I. W. K.; van Dijk, J. W.; Hamer, H. M.; Groen, B. B. L.; van Kranenburg, J.; Gorissen, S. H. M.; van Loon, L. J. C.	2019	Did not perform fiber type analysis
Changes in muscle strength, muscle fibre size and myofibrillar gene expression after immobilization and retraining in humans	Hortobagyi, T.; Dempsey, L.; Fraser, D.; Zheng, D.; Hamilton, G.; Lambert, J.; Dohm, L.	2000	Does not include male and female data
Postprandial leg uptake of triglyceride is greater in women than in men	Horton, T. J.; Commerford, S. R.; Pagliassotti, M. J.; Bessesen, D. H.	2002	Did not perform fiber type analysis
Variability in vastus lateralis fiber type distribution, fiber size, and myonuclear content along and between the legs	Horwath, O.; Envall, H.; Roja, J.; Emanuelsson, E. B.; Sanz, G.; Ekblom, B.; Apro, W.; Moberg, M.	2021	Does not include male and female data
TRAINING CESSATION DOES NOT ALTER GLUT-4 PROTEIN-LEVELS IN HUMAN SKELETAL-MUSCLE	Houmard, J. A.; Hortobagyi, T.; Neufer, P. D.; Johns, R. A.; Fraser, D. D.; Israel, R. G.; Dohm, G. L.	1993	Does not include males and females

Increased expression of microRNA-15a and microRNA-15b in skeletal muscle from adult offspring of women with diabetes in pregnancy	Houshmand-Oeregaard, A.; Schrolkamp, M.; Kelstrup, L.; Hansen, N. S.; Hjort, L.; Thuesen, A. C. B.; Broholm, C.; Mathiesen, E. R.; Clausen, T. D.; Vaag, A.; Damm, P.	2018	Did not perform fiber type analysis
Mitochondria! Bioenergetics and Fiber Type Assessments in Microbiopsy vs. Bergstrom Percutaneous Sampling of Human Skeletal Muscle	Hughes, M. C.; Ramos, S. V.; Turnbull, P. C.; Nejatbakhsh, A.; Baechler, B. L.; Tahmasebi, H.; Laham, R.; Gurd, B. J.; Quadrilatero, J.; Kane, D. A.; Perry, C. G. R.	2015	Not peer reviewed
Resistance exercise stimulates mixed muscle protein synthesis in lean and obese young adults	Hulston, C. J.; Woods, R. M.; Dewhurst-Trigg, R.; Parry, S. A.; Gagnon, S.; Baker, L.; James, L. J.; Markey, O.; Martin, N. R. W.; Ferguson, R. A.; van Hall, G.	2018	Did not perform fiber type analysis
The aging neuromuscular system and motor performance	Hunter, S. K.; Pereira, H. M.; Keenan, K. G.	2016	Review (ex: systematic review/meta-analysis)
Prolonged Adaptation to a Low or High Protein Diet Does Not Modulate Basal Muscle Protein Synthesis Rates - A Substudy	Hursel, R.; Martens, E. A. P.; Gonnissen, H. K. J.; Hamer, H. M.; Senden, J. M. G.; van Loon, L. J. C.; Westerterp-Plantenga, M. S.	2015	Did not perform fiber type analysis
Passive muscle heating attenuates the decline in vascular function caused by limb disuse	Hyldahl, R. D.; Hafen, P. S.; Nelson, W. B.; Ahmadi, M.; Pfeifer, B.; Mehling, J.; Gifford, J. R.	2021	Does not include male and female data
Moderately increased protein intake predominately from egg sources does not influence whole body, regional, or muscle composition responses to resistance training in older people	Iglay, H. B.; Apolzan, J. W.; Gerrard, D. E.; Eash, J. K.; Anderson, J. C.; Campbell, W. W.	2009	Does not include male and female data
Dexamethasone administration inhibits skeletal muscle expression of the androgen receptor and IGF-1-implications for steroid-induced myopathy	Inder, W. J.; Jang, C.; Obeyesekere, V. R.; Alford, F. P.	2010	Did not perform fiber type analysis
Skeletal Muscle 11 beta HSD1 Activity of Nondiabetic Subjects is Unaltered in Central Obesity-associated Insulin Resistance	Inder, W. J.; Obeyesekere, V. R.; Alford, F. P.; Jang, C.	2011	Did not perform fiber type analysis
Mitochondrial biogenesis and angiogenesis in skeletal muscle of the elderly	Iversen, N.; Krustrup, P.; Rasmussen, H. N.; Rasmussen, U. F.; Saltin, B.; Pilegaard, H.	2011	Does not include males and females
Vitamin D and skeletal muscle strength and endurance in COPD	Jackson, A. S.; Shrikrishna, D.; Kelly, J. L.; Hart, N.; Moxham, J.; Polkey, M. I.; Kemp, P.; Hopkinson, N. S.	2013	Does not include male and female data
Impact of blood flow-restricted bodyweight exercise on skeletal muscle adaptations	Jakobsgaard, J. E.; Christiansen, M.; Sieljacks, P.; Wang, J.; Groennebaek, T.; de Paoli, F.; Vissing, K.	2018	Does not include male and female data
Reducing NF-kappa B Signaling Nutritionally is Associated with Expedited Recovery of Skeletal Muscle Function After Damage	Jameson, T. S. O.; Pavis, G. F.; Dirks, M. L.; Lee, B. P.; Abdelrahman, D. R.; Murton, A. J.; Porter, C.; Alamdari, N.; Mikus, C. R.; Wall, B. T.; Stephens, F. B.	2021	Did not perform fiber type analysis
A novel method for evaluation of capillarity in human skeletal muscles from confocal 3D images	Janacek, J.; Cvetko, E.; Kubinova, L.; Travnik, L.; Erzen, I.	2011	Autopsies
Skeletal muscle 11 beta hydroxysteroid dehydrogenase type 1 activity is upregulated following elective abdominal surgery	Jang, C.; Obeyesekere, V. R.; Alford, F. P.; Inder, W. J.	2009	No healthy subjects or controls

Resting metabolic rate and skeletal muscle SERCA and Na+/K+ ATPase activities are not affected by fish oil supplementation in healthy older adults	Jannas-Vela, S.; Klingel, S. L.; Cervone, D. T.; Wickham, K. A.; Heigenhauser, G. J. F.; Mutch, D. M.; Holloway, G. P.; Spriet, L. L.	2020	Did not perform fiber type analysis
Effect of a 12-week endurance training program on force transfer and membrane integrity proteins in lean, obese, and type 2 diabetic subjects	Jannas-Vela, S.; Langer, H. T.; Marambio, H.; Baar, K.; Zbinden-Foncea, H.	2020	Did not perform fiber type analysis
Skeletal Muscle Satellite Cells in Sickle Cell Disease Patients and Their Responses to a Moderate-intensity Endurance Exercise Training Program	Januel, L.; Merlet, A. N.; He, Z. G.; Hourde, C.; Bartolucci, P.; Gellen, B.; Galacteros, F.; Messonnier, L. A.; Feasson, L.	2022	No healthy subjects or controls
ARE RESISTANCE TRAINING-MEDIATED DECREASES IN ULTRASOUND ECHO INTENSITY CAUSED BY CHANGES IN MUSCLE COMPOSITION, OR IS THERE AN ALTERNATIVE EXPLANATION?	Jenkins, N. D. M.	2016	Not peer reviewed
Macrophage Subpopulations and the Acute Inflammatory Response of Elderly Human Skeletal Muscle to Physiological Resistance Exercise	Jensen, S. M.; Bechshoft, C. J. L.; Heisterberg, M. F.; Schjerling, P.; Andersen, J. L.; Kjaer, M.; Mackey, A. L.	2020	Did not perform fiber type analysis
Sex- and fiber-type-related contractile properties in human single muscle fiber	Jeon, Y.; Choi, J.; Kim, H. J.; Lee, H.; Lim, J. Y.; Choi, S. J.	2019	Single Fiber Analysis
Satellite cell activity, without expansion, after nonhypertrophic stimuli	Joanisse, S.; McKay, B. R.; Nederveen, J. P.; Scribbans, T. D.; Gurd, B. J.; Gillen, J. B.; Gibala, M. J.; Tarnopolsky, M.; Parise, G.	2015	Does not include male and female data
Ectopic Lipid Accumulation and Reduced Glucose Tolerance in Elderly Adults Are Accompanied by Altered Skeletal Muscle Mitochondrial Activity	Johannsen, D. L.; Conley, K. E.; Bajpeyi, S.; Punyanitya, M.; Gallagher, D.; Zhang, Z. Y.; Covington, J.; Smith, S. R.; Ravussin, E.	2012	Does not include male and female data
Influence of muscle fibre type and pedal rate on the VO2-work rate slope during ramp exercise	Jones, A. M.; Campbell, I. T.; Pringle, J. S. M.	2004	No cross-sectional area data
Alteration in angiogenic and anti-angiogenic forms of vascular endothelial growth factor-A in skeletal muscle of patients with intermittent claudication following exercise training	Jones, W. S.; Duscha, B. D.; Robbins, J. L.; Duggan, N. N.; Regensteiner, J. G.; Kraus, W. E.; Hiatt, W. R.; Dokun, A. O.; Annex, B. H.	2012	Does not include male and female data
Myofibrillar distribution of succinate dehydrogenase activity and lipid stores differs in skeletal muscle tissue of paraplegic subjects	Jonkers, R. A. M.; Dirks, M. L.; Nabuurs, Cihc; De Feyter, H. M.; Praet, S. F. E.; Nicolay, K.; van Loon, L. J. C.; Prompers, J. J.	2012	Does not include male and female data
Large energetic adaptations of elderly muscle to resistance and endurance training	Jubrias, S. A.; Esselman, P. C.; Price, L. B.; Cress, M. E.; Conley, K. E.	2001	Does not include male and female data
Decline in isokinetic force with age: Muscle cross-sectional area and specific force	Jubrias, S. A.; Odderson, I. R.; Esselman, P. C.; Conley, K. E.	1997	Does not include male and female data
Differences in muscle contractile characteristics among bodybuilders, endurance trainers and control subjects	Jurimae, J.; Abernethy, P. J.; Quigley, B. M.; Blake, K.; McEniery, M. T.	1997	Does not include males and females
Comparing Even with Skewed Dietary Protein Distribution Shows No Difference in Muscle Protein Synthesis or Amino Acid Utilization in	Justesen, T. E. H.; Jespersen, S. E.; Thomsen, T. T.; Holm, L.; Van Hall, G.; Agergaard, J.	2022	Did not perform fiber type analysis

Healthy Older Individuals: A Randomized Controlled Trial			
The expression of androgen receptors in human neck and limb muscles: effects of training and self-administration of androgenic-anabolic steroids	Kadi, F.; Bonnerud, P.; Eriksson, A.; Thornell, L. E.	2000	Does not include males and females
Satellite cells and myonuclei in young and elderly women and men	Kadi, F.; Charifi, N.; Denis, C.; Lexell, J.	2004	Did not perform fiber type analysis
The number of satellite cells in slow and fast fibres from human vastus lateralis muscle	Kadi, F.; Charifi, N.; Henriksson, J.	2006	Does not report sex of subjects
Effects of anabolic steroids on the muscle cells of strength-trained athletes	Kadi, F.; Eriksson, A.; Holmner, S.; Thornell, L. E.	1999	Does not report sex of subjects
The effects of regular strength training on telomere length in human skeletal muscle	Kadi, F.; Ponsot, E.; Piehl-Aulin, K.; Mackey, A.; Kjaer, M.; Oskarsson, E.; Holm, L.	2008	Does not include males and females
Cereal and nonfat milk support muscle recovery following exercise	Kammer, L.; Ding, Z. P.; Wang, B.; Hara, D.; Liao, Y. H.; Ivy, J. L.	2009	Did not perform fiber type analysis
Relationship between plasma free fatty acid, intramyocellular triglycerides and long-chain acylcarnitines in resting humans	Kanaley, J. A.; Shadid, S.; Sheehan, M. T.; Guo, Z.; Jensen, M. D.	2009	Did not perform fiber type analysis
Hyperinsulinemia and skeletal muscle fatty acid trafficking	Kanaley, J. A.; Shadid, S.; Sheehan, M. T.; Guo, Z. K.; Jensen, M. D.	2013	Did not perform fiber type analysis
Smoke-induced neuromuscular junction degeneration precedes the fibre type shift and atrophy in chronic obstructive pulmonary disease	Kapchinsky, S.; Vuda, M.; Miguez, K.; Elkrief, D.; de Souza, A. R.; Baglole, C. J.; Aare, S.; MacMillan, N. J.; Baril, J.; Rozakis, P.; Sonjak, V.; Pion, C.; Aubertin-Leheudre, M.; Morais, J. A.; Jagoe, R. T.; Bourbeau, J.; Taivassalo, T.; Hepple, R. T.	2018	Does not include males and females
Age, Obesity, and Sex Effects on Insulin Sensitivity and Skeletal Muscle Mitochondrial Function	Karakelides, H.; Irving, B. A.; Short, K. R.; O'Brien, P.; Nair, K. S.	2010	Did not perform fiber type analysis
MATTERS OF FIBER SIZE AND MYONUCLEAR DOMAIN: DOES SIZE MATTER MORE THAN AGE?	Karlsen, A.; Couppe, C.; Andersen, J. L.; Mikkelsen, U. R.; Nielsen, R. H.; Magnusson, S. P.; Kjaer, M.; Mackey, A. L.	2015	Does not include males and females
REGULATION OF GLUCOSE-UTILIZATION IN HUMAN SKELETAL-MUSCLE DURING MODERATE DYNAMIC EXERCISE	Katz, A.; Sahlin, K.; Broberg, S.	1991	Does not include males and females
Sarcopenia	Keller, K.	2019	Review (ex: systematic review/meta-analysis)
INTERACTION BETWEEN GLUCOSE AND FREE FATTY-ACID METABOLISM IN HUMAN SKELETAL-MUSCLE	Kelley, D. E.; Mokan, M.; Simoneau, J. A.; Mandarino, L. J.	1993	Does not include males and females
A single bout of whole-leg, peristaltic pulse external pneumatic compression upregulates PGC-1 mRNA and endothelial nitric oxide sythase protein in human skeletal muscle tissue	Kephart, W. C.; Mobley, C. B.; Fox, C. D.; Pascoe, D. D.; Sefton, J. M.; Wilson, T. J.; Goodlett, M. D.; Kavazis, A. N.; Roberts, M. D.; Martin, J. S.	2015	Did not perform fiber type analysis

Gender-Related Differences in Muscle Injury, Oxidative Stress, and Apoptosis	Kerksick, C.; Taylor, L.; Harvey, A.; Willoughby, D.	2008	Did not perform fiber type analysis
Effect of weight loss on muscle fiber type, fiber size capillarity, and succinate dehydrogenase activity in humans	Kern, P. A.; Simsolo, R. B.; Fournier, M.	1999	Does not include male and female data
Myosin heavy chain isoform distribution in single fibres of bodybuilders	Kesidis, N.; Metaxas, T. I.; Vrabas, I. S.; Stefanidis, P.; Vamvakoudis, E.; Christoulas, K.; Mandroukas, A.; Balasas, D.; Mandroukas, K.	2008	Does not include males and females
Short-Term Effects of Dietary Fatty Acids on Muscle Lipid Composition and Serum Acylcarnitine Profile in Human Subjects	Kien, C. L.; Everingham, K. I.; Stevens, R. D.; Fukagawa, N. K.; Muoio, D. M.	2011	Did not perform fiber type analysis
Impact of resistance loading on myostatin expression and cell cycle regulation in young and older men and women	Kim, J. S.; Cross, J. M.; Bamman, M. M.	2005	Did not perform fiber type analysis
Load-mediated downregulation of myostatin mRNA is not sufficient to promote myofiber hypertrophy in humans: a cluster analysis	Kim, J. S.; Petrella, J. K.; Cross, J. M.; Bamman, M. M.	2007	Does not include male and female data
Aging affects different human muscles in various ways. An image analysis of the histomorphometric characteristics of fiber types in human masseter and vastus lateralis muscles from young adults and the very old	Kirkeby, S.; Garbarsch, C.	2000	Does not include male and female data
Histochemical studies of the masseter, the temporal and small zygomaticomandibular, and the temporomandibular masticatory muscles from aged male and female humans. Fiber types and myosin isoforms	Kirkeby, S.; Garbarsch, C.	2001	Autopsies
Regular exercise enhances insulin activation of IRS-1-associated PI3-kinase in human skeletal muscle	Kirwan, J. P.; Del Aguila, L. F.; Hernandez, J. M.; Williamson, D. L.; O'Gorman, D. J.; Lewis, R.; Krishnan, R. K.	2000	Did not perform fiber type analysis
Skeletal muscle abnormalities and exercise intolerance in older patients with heart failure and preserved ejection fraction	Kitzman, D. W.; Nicklas, B.; Kraus, W. E.; Lyles, M. F.; Eggebeen, J.; Morgan, T. M.; Haykowsky, M.	2014	Does not include male and female data
Automated segmentation of muscle fiber images using active contour models	Klemencic, A.; Kovacic, S.; Pernus, F.	1998	Case studies
McArdle disease does not affect skeletal muscle fibre type profiles in humans	Kohn, T. A.; Noakes, T. D.; Rae, D. E.; Rubio, J. C.; Santalla, A.; Nogales-Gadea, G.; Pinos, T.; Martin, M. A.; Arenas, J.; Lucia, A.	2014	No cross-sectional area data
EXERCISE REDUCES MUSCLE GLUCOSE-TRANSPORT PROTEIN (GLUT-4) MESSENGER-RNA IN TYPE-1 DIABETIC-PATIENTS	Koivisto, V. A.; Bourey, R. E.; Vuorinenmarkkola, H.; Koranyi, L.	1993	Does not include males and females
Stochastic mitochondrial DNA changes: bioenergy decline in type I skeletal muscle fibres correlates with a decline in the amount of amplifiable full-length mtDNA	Kopsidas, G.; Zhang, G. F.; Yarovaya, N.; Kovalenko, S.; Graves, S.; Richardson, M.; Linnane, A. W.	2002	No healthy subjects or controls
Fiber-type composition of the human jaw muscles - (Part 2) Role of hybrid fibers and factors responsible for inter-individual variation	Korfage, J. A. M.; Koolstra, J. H.; Langenbach, G. E. J.; van Eijden, Tmgj	2005	Review (ex: systematic review/meta-analysis)

One Week of Hospitalization Following Elective Hip Surgery Induces Substantial Muscle Atrophy in Older Patients	Kouw, I. W. K.; Groen, B. B. L.; Smeets, J. S. J.; Kramer, I. F.; van Kranenburg, J. M. X.; Nilwik, R.; Geurts, J. A. P.; ten Broeke, R. H. M.; Poeze, M.; van Loon, L. J. C.; Verdijk, L. B.	2019	No healthy subjects or controls
The effect of rowing ergometry and resistive exercise on skeletal muscle structure and function during bed rest	Krainski, F.; Hastings, J. L.; Heinicke, K.; Romain, N.; Pacini, E. L.; Snell, P. G.; Wyrick, P.; Palmer, M. D.; Haller, R. G.; Levine, B. D.	2014	Does not include male and female data
Effects of exercise on GLUT-4 and glycogenin gene expression in human skeletal muscle	Kraniou, Y.; Cameron-Smith, D.; Misso, M.; Collier, G.; Hargreaves, M.	2000	Did not perform fiber type analysis
HISTOMORPHOMETRIC AND HISTOPATHOLOGICAL STUDY OF THE HUMAN CRICOPHARYNGEUS MUSCLE - IN HEALTH AND IN MOTOR-NEURON DISEASE	Kristmundsdottir, F.; Mahon, M.; Froes, M. M. Q.; Cumming, W. J. K.	1990	Autopsies
Sex differences in single muscle fiber power in older adults	Krivickas, L. S.; Fielding, R. A.; Murray, A.; Callahan, D.; Johansson, A.; Dorer, D. J.; Frontera, W. R.	2006	Single Fiber Analysis
Age- and gender-related differences in maximum shortening velocity of skeletal muscle fibers	Krivickas, L. S.; Suh, D. W.; Wilkins, J.; Hughes, V. A.; Roubenoff, R.; Frontera, W. R.	2001	No cross-sectional area data
Resistance training in the oldest old: consequences for muscle strength, fiber types, fiber size, and MHC isoforms	Kryger, A. I.; Andersen, J. L.	2007	Does not include male and female data
Coenzyme Q10 does not improve peripheral insulin sensitivity in statin-treated men and women: the LIFESTAT study	Kuhlman, A. B.; Morville, T.; Dohlmann, T. L.; Hansen, M.; Kelly, B.; Helge, J. W.; Dela, F.	2019	Did not perform fiber type analysis
Determination of glutamine in muscle protein facilitates accurate assessment of proteolysis and de novo synthesis-derived endogenous glutamine production	Kuhn, K. S.; Schuhmann, K.; Stehle, P.; Darmaun, D.; Furst, P.	1999	Did not perform fiber type analysis
Metformin alters skeletal muscle transcriptome adaptations to resistance training in older adults	Kulkarni, A. S.; Peck, B. D.; Walton, R. G.; Kern, P. A.; Mar, J. C.; Windham, S. T.; Bamman, M. M.; Barzilai, N.; Peterson, C. A.	2020	Did not perform fiber type analysis
Mitochondrial regulators of fatty acid metabolism reflect metabolic dysfunction in type 2 diabetes mellitus	Kulkarni, S. S.; Salehzadeh, F.; Fritz, T.; Zierath, J. R.; Krook, A.; Osler, M. E.	2012	Did not perform fiber type analysis
The MOTS-c K14Q polymorphism in the mtDNA is associated with muscle fiber composition and muscular performance	Kumagai, H.; Natsume, T.; Kim, S. J.; Tobina, T.; Miyamoto-Mikami, E.; Shiose, K.; Ichinoseki-Sekine, N.; Kakigi, R.; Tsuzuki, T.; Miller, B.; Yen, K.; Murakami, H.; Miyachi, M.; Zempo, H.; Dobashi, S.; Machida, S.; Kobayashi, H.; Naito, H.; Cohen, P.; Fuku, N.	2022	Did not perform fiber type analysis
Cortisol concentrations in human skeletal muscle tissue after phonophoresis with 10% hydrocortisone gel	Kuntz, A. R.; Griffiths, C. M.; Rankin, J. M.; Armstrong, C. W.; McLoughlin, T. J.	2006	Did not perform fiber type analysis
Striated muscle-specific serine/threonine-protein kinase beta segregates with high versus low responsiveness to endurance exercise training	Kusic, D.; Connolly, J.; Kainulainen, H.; Semenova, E. A.; Borisov, O. V.; Larin, A. K.; Popov, D. V.; Generozov, E. V.; Ahmetov,, II; Britton, S. L.; Koch, L. G.; Burniston, J. G.	2020	Does not include male and female data

Effects of power training on muscle structure and neuromuscular performance	Kyrolainen, H.; Avela, J.; McBride, J. M.; Koskinen, S.; Andersen, J. L.; Sipila, S.; Takala, T. E. S.; Komi, P. V.	2005	Does not include males and females
Predictors of Whole-Body Insulin Sensitivity Across Ages and Adiposity in Adult Humans	Lalia, A. Z.; Dasari, S.; Johnson, M. L.; Robinson, M. M.; Konopka, A. R.; Distelmaier, K.; Port, J. D.; Glavin, M. T.; Esponda, R. R.; Nair, K. S.; Lanza, I. R.	2016	Did not perform fiber type analysis
Influence of omega-3 fatty acids on skeletal muscle protein metabolism and mitochondrial bioenergetics in older adults	Lalia, A. Z.; Dasari, S.; Robinson, M. M.; Abid, H.; Morse, D. M.; Klaus, K. A.; Lanza, I. R.	2017	Did not perform fiber type analysis
The effects of resistance training with or without peanut protein supplementation on skeletal muscle and strength adaptations in older individuals	Lamb, D. A.; Moore, J. H.; Smith, M. A.; Vann, C. G.; Osburn, S. C.; Ruple, B. A.; Fox, C. D.; Smith, K. S.; Altonji, O. M.; Power, Z. M.; Cerovsky, A. E.; Ross, C. O.; Cao, A. T.; Goodlett, M. D.; Huggins, K. W.; Fruge, A. D.; Young, K. C.; Roberts, M. D.	2020	Did not perform fiber type analysis
Aquatic Treadmill Training Reduces Blood Pressure Reactivity to Physical Stress	Lambert, B. S.; Greene, N. P.; Carradine, A. T.; Joubert, D. P.; Fluckey, J. D.; Riechman, S. E.; Crouse, S. F.	2014	Did not perform fiber type analysis
Anabolic responses to acute and chronic resistance exercise are enhanced when combined with aquatic treadmill exercise	Lambert, B. S.; Shimkus, K. L.; Fluckey, J. D.; Riechman, S. E.; Greene, N. P.; Cardin, J. M.; Crouse, S. F.	2015	Did not perform fiber type analysis
Effects of aging and resistance exercise on determinants of muscle strength	Lambert, C. P.; Evans, W. J.	2002	Review (ex: systematic review/meta-analysis)
Exercise but not diet-induced weight loss decreases skeletal muscle inflammatory gene expression in frail obese elderly persons	Lambert, C. P.; Wright, N. R.; Finck, B. N.; Villareal, D. T.	2008	No healthy subjects or controls
Effect of 23-day muscle disuse on sarcoplasmic reticulum Ca2+ properties and contractility in human type I and type II skeletal muscle fibers	Lamboley, C. R.; Wyckelsma, V. L.; Perry, B. D.; McKenna, M. J.; Lamb, G. D.	2016	Does not include male and female data
Endocrine responses and acute mTOR pathway phosphorylation to resistance exercise with leucine and whey	Lane, M. T.; Herda, T. J.; Fry, A. C.; Cooper, M. A.; Andre, M. J.; Gallagher, P. M.	2017	Does not include males and females
Actions of short-term fasting on human skeletal muscle myogenic and atrogenic gene expression	Larsen, A. E.; Tunstal, R. J.; Carey, K. A.; Nicholas, G.; Kambadur, R.; Crowe, T. C.; Cameron-Smith, D.	2006	Did not perform fiber type analysis
The Kenyan runners	Larsen, H. B.; Sheel, A. W.	2015	Review (ex: systematic review/meta-analysis)
Effect of dietary fat on serum and intramyocellular lipids and running performance	Larson-Meyer, D. E.; Borkhsenious, O. N.; Gullett, J. C.; Russell, R. R.; Devries, M. C.; Smith, S. R.; Ravussin, E.	2008	Did not perform fiber type analysis
The effect of a needle biopsy of the vastus lateralis on isokinetic muscular performance	Laskin, J.; Bell, G.; Burnham, R.	1998	Did not perform fiber type analysis
Effects of blood-flow restriction on biomarkers of myogenesis in response to resistance exercise	Layne, A. S.; Larkin-Kaiser, K.; MacNeil, R. G.; Dirain, M.; Sandesara, B.; Manini, T. M.; Buford, T. W.	2017	Did not perform fiber type analysis

Tetrahydropalmatine promotes myoblast differentiation through activation of p38MAPK and MyoD	Lee, S. J.; Yoo, M.; Go, G. Y.; Hwang, J.; Lee, H. G.; Kim, Y. K.; Seo, D. W.; Baek, N. I.; Ryu, J. H.; Kang, J. S.; Bae, G. U.	2014	Animal study
Age-associated decrease of type IIA/B human skeletal muscle fibers	Lee, W. S.; Cheung, W. H.; Qin, L.; Tang, N.; Leung, K. S.	2006	Does not include male and female data
MUSCLE PROTEINS DURING 60-DAY BEDREST IN WOMEN: IMPACT OF EXERCISE OR NUTRITION	Lemoine, J. K.; Haus, J. M.; Trappe, S. W.; Trappe, T. A.	2009	Does not include males and females
Differences in Muscle and Adipose Tissue Gene Expression and Cardio-Metabolic Risk Factors in the Members of Physical Activity Discordant Twin Pairs	Leskinen, T.; Rinnankoski-Tuikka, R.; Rintala, M.; Seppanen-Laakso, T.; Pollanen, E.; Alen, M.; Sipila, S.; Kaprio, J.; Kovanen, V.; Rahkila, P.; Oresic, M.; Kainulainen, H.; Kujala, U. M.	2010	Did not perform fiber type analysis
Reduced whole-body fat oxidation in women and in the elderly	Levadoux, E.; Morio, B.; Montaurier, C.; Puissant, V.; Boirie, Y.; Fellmann, N.; Picard, B.; Rousset, P.; Beaufrere, B.; Ritz, P.	2001	Does not include male and female data
Bioenergetic adaptation of individual human diaphragmatic myofibers to severe COPD	Levine, S.; Gregory, C.; Nguyen, T.; Shrager, J.; Kaiser, L.; Rubinstein, N.; Dudley, G.	2002	No healthy subjects or controls
Cellular adaptations in the diaphragm in chronic obstructive pulmonary disease	Levine, S.; Kaiser, L.; Leferovich, J.; Tikunov, B.	1997	Does not include male and female data
Effect of endurance and/or strength training on muscle fiber size, oxidative capacity, and capillarity in hemodialysis patients	Lewis, M. I.; Fournier, M.; Wang, H. Y.; Storer, T. W.; Casaburi, R.; Kopple, J. D.	2015	Does not include male and female data
Muscle capillarization: Morphological and morphometrical analyses of biopsy samples	Lexell, J.	1997	Review (ex: systematic review/meta-analysis)
Skeletal Muscle Angiopoietin-Like Protein 4 and Glucose Metabolism in Older Adults after Exercise and Weight Loss	Li, G. Y.; Zhang, H. F.; Ryan, A. S.	2020	Did not perform fiber type analysis
High doses of anti-inflammatory drugs compromise muscle strength and hypertrophic adaptations to resistance training in young adults	Lilja, M.; Mandic, M.; Apro, W.; Melin, M.; Olsson, K.; Rosenborg, S.; Gustafsson, T.; Lundberg, T. R.	2018	Did not perform fiber type analysis
Increased protein intake derived from leucine-enriched protein enhances the integrated myofibrillar protein synthetic response to short-term resistance training in untrained men and women: a 4-day randomized controlled trial	Lim, C.; Traylor, D. A.; McGlory, C.; Joanisse, S.; McKendry, J.; Grewal, T.; McLeod, J. C.; Prior, T.; Nunes, E. A.; Lees, M.; Phillips, S. M.	2022	Did not perform fiber type analysis
MUSCLE-FIBER CHARACTERISTICS, CAPILLARIES AND ENZYMES IN PATIENTS WITH FIBROMYALGIA AND CONTROLS	Lindh, M.; Johansson, G.; Hedberg, M.; Henning, G. B.; Grimby, G.	1995	Does not include males and females
The Impact of Endurance Training on Human Skeletal Muscle Memory, Global Isoform Expression and Novel Transcripts	Lindholm, M. E.; Giacomello, S.; Solnestam, B.; Fischer, H.; Huss, M.; Kjellqvist, S.; Sundberg, C. J.	2016	Did not perform fiber type analysis
The human skeletal muscle transcriptome: sex differences, alternative splicing, and tissue homogeneity assessed with RNA sequencing	Lindholm, M. E.; Huss, M.; Solnestam, B. W.; Kjellqvist, S.; Lundeberg, J.; Sundberg, C. J.	2014	Did not perform fiber type analysis

FIBER TYPE COMPOSITION OF THE HUMAN FEMALE TRAPEZIUS MUSCLE - ENZYME-HISTOCHEMICAL CHARACTERISTICS	Lindman, R.; Eriksson, A.; Thornell, L. E.	1991	Autopsies
Performance and fibre characteristics of human skeletal muscle during short sprint training and detraining on a cycle ergometer	Linossier, M. T.; Dormois, D.; Geyssant, A.; Denis, C.	1997	Does not include males and females
Skeletal muscle gene expression in response to resistance exercise: sex specific regulation	Liu, D.; Sartor, M. A.; Nader, G. A.; Gutmann, L.; Treutelaar, M. K.; Pistilli, E. E.; IglayReger, H. B.; Burant, C. F.; Hoffman, E. P.; Gordon, P. M.	2010	Did not perform fiber type analysis
Microarray Analysis Reveals Novel Features of the Muscle Aging Process in Men and Women	Liu, D. M.; Sartor, M. A.; Nader, G. A.; Pistilli, E. E.; Tanton, L.; Lilly, C.; Gutmann, L.; IglayReger, H. B.; Visich, P. S.; Hoffman, E. P.; Gordon, P. M.	2013	Did not perform fiber type analysis
Fiber content and myosin heavy chain composition of muscle spindles in aged human biceps brachii	Liu, J. X.; Eriksson, P. C.; Thornell, L. E.; Pedrosa-Domellof, F.	2005	Autopsies
Osteocalcin Induces Proliferation via Positive Activation of the PI3K/Akt, P38 MAPK Pathways and Promotes Differentiation Through Activation of the GPRC6A-ERK1/2 Pathway in C2C12 Myoblast Cells	Liu, S. F.; Gao, F.; Wen, L.; Ouyang, M.; Wang, Y.; Wang, Q.; Luo, L. P.; Jian, Z. J.	2017	Animal study
Prostaglandin E-2/cyclooxygenase pathway in human skeletal muscle: influence of muscle fiber type and age	Liu, S. Z.; Jemiolo, B.; Lavin, K. M.; Lester, B. E.; Trappe, S. W.; Trappe, T. A.	2016	Did not perform fiber type analysis
Higher proportion of fast-twitch (type II) muscle fibres in idiopathic inflammatory myopathies - evident in chronic but not in untreated newly diagnosed patients	Loell, I.; Helmers, S. B.; Dastmalchi, M.; Alexanderson, H.; Munters, L. A.; Nennesmo, I.; Lindroos, E.; Borg, K.; Lundberg, I. E.; Esbjornsson, M.	2011	Does not include male and female data
Skeletal muscle properties show collagen organization and immune cell content are associated with resistance exercise response heterogeneity in older persons	Long, D. E.; Peck, B. D.; Lavin, K. M.; Dungan, C. M.; Kosmac, K.; Tuggle, S. C.; Bamman, M. M.; Kern, P. A.; Peterson, C. A.	2022	Does not include male and female data
Development and characterization of polyspecific anti-mitochondrion antibodies for proteomics studies on in toto tissue homogenates	Loro, E.; Gianazza, E.; Cazzola, S.; Malena, A.; Wait, R.; Begum, S.; Brizio, C.; Dabbeni-Sala, F.; Vergani, L.	2009	No healthy subjects or controls
Time course of proteolytic, cytokine, and myostatin gene expression after acute exercise in human skeletal muscle	Louis, E.; Raue, U.; Yang, Y. F.; Jemiolo, B.; Trappe, S.	2007	No cross-sectional area data
Immunohistochemical evidence of nerve growth factor in Dupuytren's diseased palmar fascia	Lubahn, J. D.; Pollard, M.; Cooney, T.	2007	Did not perform fiber type analysis
Differential Autophagy Response in Men and Women After Muscle Damage	Luk, H. Y.; Appell, C.; Levitt, D. E.; Jiwan, N. C.; Vingren, J. L.	2021	Did not perform fiber type analysis
Resistance exercise-induced hormonal response promotes satellite cell proliferation in untrained men but not in women	Luk, H. Y.; Levitt, D. E.; Boyett, J. C.; Rojas, S.; Flader, S. M.; McFarlin, B. K.; Vingren, J. L.	2019	Did not perform fiber type analysis
Stereological estimates indicate that aging does not alter the capillary length density in the human posterior cricoarytenoid muscle	Lyon, M. J.; Steer, L. M.; Malmgren, L. T.	2007	Autopsies

Satellite cell pool expansion is affected by skeletal muscle characteristics	Macaluso, F.; Brooks, N. E.; Niesler, C. U.; Myburgh, K. H.	2013	Does not include males and females
Preferential Type II Muscle Fiber Damage From Plyometric Exercise	Macaluso, F.; Isaacs, A. W.; Myburgh, K. H.	2012	Does not include males and females
Skeletal muscle collagen content in humans after high-force eccentric contractions	Mackey, A. L.; Donnelly, A. E.; Turpeenniemi-Hujanen, T.; Roper, H. P.	2004	Did not perform fiber type analysis
Enhanced satellite cell proliferation with resistance training in elderly men and women	Mackey, A. L.; Esmarck, B.; Kadi, F.; Koskinen, S. O. A.; Kongsgaard, M.; Sylvestersen, A.; Hansen, J. J.; Larsen, G.; Kjaer, M.	2007	Does not include male and female data
ASSESSMENT OF SATELLITE CELL NUMBER AND ACTIVITY STATUS IN HUMAN SKELETAL MUSCLE BIOPSIES	Mackey, A. L.; Kjaer, M.; Charifi, N.; Henriksson, J.; Bojsen-Moller, J.; Holm, L.; Kadi, F.	2009	Does not report sex of subjects
The Order of Exercise during Concurrent Training for Rehabilitation Does Not Alter Acute Genetic Expression, Mitochondrial Enzyme Activity or Improvements in Muscle Function	MacNeil, L. G.; Glover, E.; Bergstra, T. G.; Safdar, A.; Tarnopolsky, M. A.	2014	Did not perform fiber type analysis
Women Have Higher Protein Content of beta-Oxidation Enzymes in Skeletal Muscle than Men	Maher, A. C.; Akhtar, M.; Vockley, J.; Tarnopolsky, M. A.	2010	Did not perform fiber type analysis
Sex Differences in Global mRNA Content of Human Skeletal Muscle	Maher, A. C.; Fu, M. H.; Isfort, R. J.; Varbanov, A. R.; Qu, X. A.; Tarnopolsky, M. A.	2009	Same subjects as another study
Low expression of long-chain acyl-CoA dehydrogenase in human skeletal muscle	Maher, A. C.; Mohsen, A. W.; Vockley, J.; Tarnopolsky, M. A.	2010	Did not perform fiber type analysis
Short-term metformin ingestion by healthy older adults improves myoblast function	Mahmassani, Z. S.; McKenzie, A. I.; Petrocelli, J. J.; de Hart, N. M.; Reidy, P. T.; Fix, D. K.; Ferrara, P. J.; Funai, K.; Drummond, M. J.	2021	Did not perform fiber type analysis
Angiotensin II receptor subtypes in the skeletal muscle vasculature of patients with severe congestive heart failure	Malendowicz, S. L.; Ennezat, P. V.; Testa, M.; Murray, L.; Sonnenblick, E. H.; Evans, T.; LeJemtel, T. H.	2000	Did not perform fiber type analysis
Fat content in individual muscle fibers of lean and obese subjects	Malenfant, P.; Joanisse, D. R.; Theriault, R.; Goodpaster, B. H.; Kelley, D. E.; Simoneau, J. A.	2001	Does not include male and female data
Elevated intramyocellular lipid concentration in obese subjects is not reduced after diet and exercise training	Malenfant, P.; Tremblay, A.; Doucet, E.; Imbeault, P.; Simoneau, J. A.; Joanisse, D. R.	2001	Does not include male and female data
Impaired Skeletal Muscle Oxygenation and Exercise Tolerance in Pulmonary Hypertension	Malenfant, S.; Potus, F.; Mainguy, V.; Leblanc, E.; Malenfant, M.; Ribeiro, F.; Saey, D.; Maltais, F.; Bonnet, S.; Provencher, S.	2015	Does not include male and female data
Dietary Protein Distribution Positively Influences 24-h Muscle Protein Synthesis in Healthy Adults	Mamerow, M. M.; Mettler, J. A.; English, K. L.; Casperson, S. L.; Arentson-Lantz, E.; Sheffield-Moore, M.; Layman, D. K.; Paddon-Jones, D.	2014	Did not perform fiber type analysis

Deltoid muscle fiber characteristics in adolescent and adult wrestlers	Mandroukas, A.; Metaxas, T.; Kesidis, N.; Christoulas, K.; Vamvakoudis, E.; Stefanidis, P.; Heller, J.; Ekblom, B.; Mandroukas, K.	2010	Does not include males and females
The influence of muscle fiber size and type distribution on electromyographic measures of back muscle fatigability	Mannion, A. F.; Dumas, G. A.; Stevenson, J. M.; Cooper, R. G.	1998	Does not include male and female data
CARNOSINE AND ANSERINE CONCENTRATIONS IN THE QUADRICEPS FEMORIS MUSCLE OF HEALTHY HUMANS	Mannion, A. F.; Jakeman, P. M.; Dunnett, M.; Harris, R. C.; Willan, P. L. T.	1992	Did not perform fiber type analysis
Impact of high-intensity interval training with or without l-citrulline on physical performance, skeletal muscle, and adipose tissue in obese older adults	Marcangeli, V.; Youssef, L.; Dulac, M.; Carvalho, L. P.; Hajj-Boutros, G.; Reynaud, O.; Guegan, B.; Buckinx, F.; Gaudreau, P.; Morais, J. A.; Mauriege, P.; Noirez, P.; Aubertin-Leheudre, M.; Gouspillou, G.	2022	Did not perform fiber type analysis
Muscle histopathology in myasthenia gravis with antibodies against MuSK and AChR	Martignago, S.; Fanin, M.; Albertini, E.; Pegoraro, E.; Angelini, C.	2009	No healthy subjects or controls
A single 60-min bout of peristaltic pulse external pneumatic compression transiently upregulates phosphorylated ribosomal protein s6	Martin, J. S.; Kephart, W. C.; Mobley, C. B.; Wilson, T. J.; Goodlett, M. D.; Roberts, M. D.	2017	Did not perform fiber type analysis
Lactate-induced lactylation in skeletal muscle is associated with insulin resistance in humans	Maschari, D.; Saxena, G.; Law, T. D.; Walsh, E.; Campbell, M. C.; Consitt, L. A.	2022	Did not perform fiber type analysis
Determining the Influence of Habitual Dietary Protein Intake on Physiological Muscle Parameters in Youth and Older Age	Mathewson, S. L.; Gordon, A. L.; Smith, K.; Atherton, P. J.; Greig, C. A.; Phillips, B. E.	2021	Did not perform fiber type analysis
HISTOCHEMICAL AND FUNCTIONAL PARAMETERS IN NORDIC COMBINATION ATHLETES	Matolin, S.; Vaverka, F.; Lunak, J.; Novak, J.; Horak, V.; Krejci, P.	1994	Does not report sex of subjects
Observations of branched-chain amino acid administration in humans	Matthews, D. E.	2005	Review (ex: systematic review/meta-analysis)
The effects of aging on biceps brachii muscle fibers - A morphometrical study from biopsies and autopsies	Mattiello-Sverzut, A. C.; Chimelli, L.; Moura, M. S. D.; Teixeira, S.; de Oliveira, J. A. M.	2003	No healthy subjects or controls
No Additional Benefits of Block-Over Evenly-Distributed High-Intensity Interval Training within a Polarized Microcycle	McGawley, K.; Juudas, E.; Kazior, Z.; Strom, K.; Blomstrand, E.; Hansson, O.; Holmberg, H. C.	2017	Does not include male and female data
Superior Aerobic Capacity and Indices of Skeletal Muscle Morphology in Chronically Trained Master Endurance Athletes Compared With Untrained Older Adults	McKendry, J.; Joanisse, S.; Baig, S.; Liu, B. Y.; Parise, G.; Greig, C. A.; Breen, L.	2020	Does not include males and females
Muscle strength after resistance training correlates to mediators of muscle mass and mitochondrial respiration in middle-aged adults	McKenna, C. F.; Salvador, A. F.; Keeble, A. R.; Khan, N. A.; De Lisio, M.; Konopka, A. R.; Paluska, S. A.; Burd, N. A.	2022	Did not perform fiber type analysis
Physiological and functional evaluation of healthy young and older men and women: design of the European MyoAge study	McPhee, J. S.; Hogrel, J. Y.; Maier, A. B.; Seppet, E.; Seynnes, O. R.; Sipila, S.; Bottinelli, R.; Barnouin, Y.; Bijlsma, A. Y.; Gapeyeva, H.; Maden-Wilkinson, T. M.; Meskers, C. G.; Paasuke, M.; Sillanpaa, E.;	2013	Did not perform fiber type analysis

	Stenroth, L.; Butler-Browne, G.; Narici, M. V.; Jones, D. A.		
Resistance Exercise Reverses Aging in Human Skeletal Muscle	Melov, S.; Tarnopolsky, M. A.; Beckman, K.; Felkey, K.; Hubbard, A.	2007	Did not perform fiber type analysis
Characteristics of skeletal muscle mitochondrial biogenesis induced by moderate-intensity exercise and weight loss in obesity	Menshikova, E. V.; Ritov, V. B.; Ferrell, R. E.; Azuma, K.; Goodpaster, B. H.; Kelley, D. E.	2007	Did not perform fiber type analysis
Effects of weight loss and physical activity on skeletal muscle mitochondrial function in obesity	Menshikova, E. V.; Ritov, V. B.; Toledo, F. G. S.; Ferrell, R. E.; Goodpaster, B. H.; Kelley, D. E.	2005	Did not perform fiber type analysis
Acute and chronic effects of resistance training on skeletal muscle markers of mitochondrial remodeling in older adults	Mesquita, P. H. C.; Lamb, D. A.; Parry, H. A.; Moore, J. H.; Smith, M. A.; Vann, C. G.; Osburn, S. C.; Fox, C. D.; Ruple, B. A.; Huggins, K. W.; Fruge, A. D.; Young, K. C.; Kavazis, A. N.; Roberts, M. D.	2020	Did not perform fiber type analysis
Metformin Increases Protein Phosphatase 2A Activity in Primary Human Skeletal Muscle Cells Derived from Lean Healthy Participants	Mestareehi, A.; Zhang, X. M.; Seyoum, B.; Msallaty, Z.; Mallisho, A.; Burghardt, K. J.; Kowluru, A.; Yi, Z. P.	2021	Did not perform fiber type analysis
Physiological and molecular responses to an acute bout of reduced-exertion high-intensity interval training (REHIT)	Metcalfe, R. S.; Koumanov, F.; Ruffino, J. S.; Stokes, K. A.; Holman, G. D.; Thompson, D.; Vollaard, N. B. J.	2015	Did not perform fiber type analysis
High-Frequency Neuromuscular Electrical Stimulation Increases Anabolic Signaling	Mettler, J. A.; Magee, D. M.; Doucet, B. M.	2018	Did not perform fiber type analysis
Skeletal muscle morphology and regulatory signalling in endurance-trained and sedentary individuals: The influence of ageing	Mikkelsen, U. R.; Agergaard, J.; Couppe, C.; Grosset, J. F.; Karlsen, A.; Magnusson, S. P.; Schjerling, P.; Kjaer, M.; Mackey, A. L.	2017	Does not include males and females
Molecular determinants of force production in human skeletal muscle fibers: effects of myosin isoform expression and cross-sectional area	Miller, M. S.; Bedrin, N. G.; Ades, P. A.; Palmer, B. M.; Toth, M. J.	2015	Does not include male and female data
Skeletal muscle myofilament adaptations to aging, disease, and disuse and their effects on whole muscle performance in older adult humans	Miller, M. S.; Callahan, D. M.; Toth, M. J.	2014	Review (ex: systematic review/meta-analysis)
Moderate-intensity resistance exercise alters skeletal muscle molecular and cellular structure and function in inactive older adults with knee osteoarthritis	Miller, M. S.; Callahan, D. M.; Tourville, T. W.; Slauterbeck, J. R.; Kaplan, A.; Fiske, B. R.; Savage, P. D.; Ades, P. A.; Beynnon, B. D.; Toth, M. J.	2017	No healthy subjects or controls
Chronic heart failure decreases cross-bridge kinetics in single skeletal muscle fibres from humans	Miller, M. S.; VanBuren, P.; LeWinter, M. M.; Braddock, J. M.; Ades, P. A.; Maughan, D. W.; Palmer, B. M.; Toth, M. J.	2010	Does not include male and female data
The dynamic equilibrium between ATP synthesis and ATP consumption is lower in isolated mitochondria from myotubes established from type 2 diabetic subjects compared to lean control	Minet, A. D.; Gaster, M.	2011	Did not perform fiber type analysis
Skeletal muscle studies in patients with HIV-related wasting syndrome	Miro, O.; Pedrol, E.; Cebrian, M.; Masanes, F.; Casademont, J.; Mallolas, J.; Grau, J. M.	1997	Does not report sex of subjects
Increased levels of apoptosis in gastrocnemius skeletal muscle in patients with peripheral arterial disease	Mitchell, R. G.; Duscha, B. D.; Robbins, J. L.; Redfern, S. I.; Chung, J.; Bensimhon, D. R.;	2007	Did not perform fiber type analysis

	Kraus, W. E.; Hiatt, W. R.; Regensteiner, J. G.; Annex, B. H.		
RECIPROCAL EXPRESSION OF DYSTROPHIN AND UTROPHIN IN MUSCLES OF DUCHENNE MUSCULAR-DYSTROPHY PATIENTS, FEMALE DMD-CARRIERS AND CONTROL SUBJECTS	Mizuno, Y.; Nonaka, I.; Hirai, S.; Ozawa, E.	1993	Does not report sex of subjects
Exercise Induces Different Molecular Responses in Trained and Untrained Human Muscle	Moberg, M.; Lindholm, M. E.; Reitzner, S. M.; Ekblom, B.; Sundberg, C. J.; Psilander, N.	2020	Did not perform fiber type analysis
Normal to enhanced intrinsic mitochondrial respiration in skeletal muscle of middle- to older-aged women and men with uncomplicated type 1 diabetes	Monaco, C. M. F.; Tarnopolsky, M. A.; Dial, A. G.; Nederveen, J. P.; Rebalka, I. A.; Nguyen, M.; Turner, L. V.; Perry, C. G. R.; Ljubicic, V.; Hawke, T. J.	2021	Did not perform fiber type analysis
Adverse changes in fibre type composition of the human masseter versus biceps brachii muscle during aging	Monemi, M.; Eriksson, P. O.; Eriksson, A.; Thornell, L. E.	1998	Autopsies
A mycoprotein-based high-protein vegan diet supports equivalent daily myofibrillar protein synthesis rates compared with an isonitrogenous omnivorous diet in older adults: a randomised controlled trial	Monteyne, A. J.; Dunlop, M. V.; Machin, D. J.; Coelho, M. O. C.; Pavis, G. F.; Porter, C.; Murton, A. J.; Abdelrahman, D. R.; Dirks, M. L.; Stephens, F. B.; Wall, B. T.	2021	Did not perform fiber type analysis
Hybrid fiber alterations in exercising seniors suggest contribution to fast-to-slow muscle fiber shift	Moreillon, M.; Alonso, S. C.; Broskey, N. T.; Greggio, C.; Besson, C.; Rousson, V.; Amati, F.	2019	Does not include male and female data
Influence of Gender, Obesity, and Muscle Lipase Activity on Intramyocellular Lipids in Sedentary Individuals	Moro, C.; Galgani, J. E.; Luu, L.; Pasarica, M.; Mairal, A.; Bajpeyi, S.; Schmitz, G.; Langin, D.; Liebisch, G.; Smith, S. R.	2009	Did not perform fiber type analysis
Muscle Protein Anabolic Resistance to Essential Amino Acids Does Not Occur in Healthy Older Adults Before or After Resistance Exercise Training	Moro, T.; Brightwell, C. R.; Deer, R. R.; Graber, T. G.; Galvan, E.; Fry, C. S.; Volpi, E.; Rasmussen, B. B.	2018	Does not include male and female data
Physical inactivity affects skeletal muscle insulin signaling in a birth weight-dependent manner	Mortensen, B.; Friedrichsen, M.; Andersen, N. R.; Alibegovic, A. C.; Hojbjerre, L.; Sonne, M. P.; Stallknecht, B.; Dela, F.; Wojtaszewski, J. F. P.; Vaag, A.	2014	Does not include males and females
Effect of birth weight and 12 weeks of exercise training on exercise-induced AMPK signaling in human skeletal muscle	Mortensen, B.; Hingst, J. R.; Frederiksen, N.; Hansen, R. W. W.; Christiansen, C. S.; Iversen, N.; Friedrichsen, M.; Birk, J. B.; Pilegaard, H.; Hellsten, Y.; Vaag, A.; Wojtaszewski, J. F. P.	2013	Does not include males and females
Different Molecular and Structural Adaptations with Eccentric and Conventional Strength Training in Elderly Men and Women	Mueller, M.; Breil, F. A.; Lurman, G.; Klossner, S.; Fluck, M.; Billeter, R.; Dapp, C.; Hoppeler, H.	2011	Does not include male and female data
Different response to eccentric and concentric training in older men and women	Mueller, M.; Breil, F. A.; Vogt, M.; Steiner, R.; Lippuner, K.; Popp, A.; Klossner, S.; Hoppeler, H.; Dapp, C.	2009	Does not include male and female data
Training Effects on Skeletal Muscle Calcium Handling in Human Chronic Heart Failure	Munkvik, M.; Rehn, T. A.; Slettalokkken, G.; Hasic, A.; Hallen, J.; Sjaastad, I.; Sejersted, O. M.; Lunde, P. K.	2010	Does not include males and females
Effects of endurance training status and sex differences on Na+,K+-pump mRNA expression,	Murphy, K. T.; Aughey, R. J.; Petersen, A. C.; Clark, S. A.; Goodman, C.; Hawley, J. A.;	2007	Did not perform fiber type analysis

content and maximal activity in human skeletal muscle	Cameron-Smith, D.; Snow, R. J.; McKenna, M. J.		
Prolonged submaximal exercise induces isoform-specific Na+-K+-ATPase mRNA and protein responses in human skeletal muscle	Murphy, K. T.; Petersen, A. C.; Goodman, C.; Gong, X.; Leppik, J. A.; Garnham, A. P.; Cameron-Smith, D.; Snow, R. J.; McKenna, M. J.	2006	Did not perform fiber type analysis
Intense exercise up-regulates Na+ ,K+-ATPase isoform mRNA, but not protein expression in human skeletal muscle	Murphy, K. T.; Snow, R. J.; Petersen, A. C.; Murphy, R. M.; Mollica, J.; Lee, J. S.; Garnham, A. P.; Aughey, R. J.; Leppik, J. A.; Medved, I.; Cameron-Smith, D.; McKenna, M. J.	2004	Did not perform fiber type analysis
Human skeletal muscle creatine transporter mRNA and protein expression in healthy, young males and females	Murphy, R. M.; Tunstall, R. J.; Mehan, K. A.; Cameron-Smith, D.; McKenna, M. J.; Spriet, L. L.; Hargreaves, M.; Snow, R. J.	2003	Does not include male and female data
The human endurance athlete: heterogeneity and adaptability of selected exercise and skeletal muscle characteristics	Myburgh, K. H.; Weston, A. R.	1998	Review (ex: systematic review/meta-analysis)
Morphometric analysis of the insertion of the upper head of the lateral pterygoid muscle	Naidoo, L. C. D.; Juniper, R. P.	1997	Autopsies
Extracellular vesicle-associated miRNAs are an adaptive response to gestational diabetes mellitus	Nair, S.; Guanzon, D.; Jayabalan, N.; Lai, A.; Scholz-Romero, K.; de Croft, P. K.; Ormazabal, V.; Palma, C.; Diaz, E.; McCarthy, E. A.; Shub, A.; Miranda, J.; Gratacos, E.; Crispi, F.; Duncombe, G.; Lappas, M.; McIntyre, H. D.; Rice, G.; Salomon, C.	2021	Does not include males and females
Masseter Muscle Properties Differ between the Left and Right Sides in Mandibular Class III Patients with Asymmetry	Nakashima, A.; Yamada, T.; Sugiyama, G.; Mizunoya, W.; Nakano, H.; Yasuda, K.; Takahashi, I.; Mori, Y.	2020	Does not include male and female data
Sarcopenia: characteristics, mechanisms and functional significance	Narici, M. V.; Maffulli, N.	2010	Review (ex: systematic review/meta-analysis)
Skeletal Muscle Fiber Size and Gene Expression in the Oldest-Old With Differing Degrees of Mobility	Naro, F.; Venturelli, M.; Monaco, L.; Toniolo, L.; Muti, E.; Milanese, C.; Zhao, J.; Richardson, R. S.; Schena, F.; Reggiani, C.	2019	Does not include male and female data
Influence of low-dose aspirin, resistance exercise, and sex on human skeletal muscle PGE(2)/COX pathway activity	Naruse, M.; Fountain, W. A.; Claiborne, A.; Chambers, T. L.; Jones, A. M.; Stroh, A. M.; Montenegro, C. F.; Lynch, C. E.; Minchev, K.; Trappe, S.; Trappe, T. A.	2021	Did not perform fiber type analysis
Serine/threonine protein kinase 25 (STK25): a novel negative regulator of lipid and glucose metabolism in rodent and human skeletal muscle	Nerstedt, A.; Cansby, E.; Andersson, C. X.; Laakso, M.; Stancakova, A.; Bluher, M.; Smith, U.; Mahlapuu, M.	2012	Did not perform fiber type analysis
Substrate-Specific Respiration of Isolated Skeletal Muscle Mitochondria after 1 h of Moderate Cycling in Sedentary Adults	Newsom, S. A.; Stierwalt, H. D.; Ehrlicher, S. E.; Robinson, M. M.	2021	Did not perform fiber type analysis
Developmental myosin heavy chains in the adult human diaphragm: coexpression patterns and effect of COPD	Nguyen, T.; Shrager, J.; Kaiser, L.; Mei, L. J.; Daood, M.; Watchko, J.; Rubinstein, N.; Levine, S.	2000	No healthy subjects or controls

Relationship of physical function to vastus lateralis capillary density and metabolic enzyme activity in elderly men and women	Nicklas, B. J.; Leng, I.; Delbono, O.; Kitzman, D. W.; Marsh, A. P.; Hundley, W. G.; Lyles, M. F.; O'Rourke, K. S.; Annex, B. H.; Kraus, W. E.	2008	Does not include male and female data
Resting MAPK expression in chronically trained endurance runners	Nicoll, J. X.; Fry, A. C.; Galpin, A. J.; Thomason, D. B.; Moore, C. A.	2017	Does not report sex of subjects
Sex-based differences in resting MAPK, androgen, and glucocorticoid receptor phosphorylation in human skeletal muscle	Nicoll, J. X.; Fry, A. C.; Mosier, E. M.	2019	Did not perform fiber type analysis
IGF-I measurement across blood, interstitial fluid, and muscle biocompartments following explosive, high-power exercise	Nindl, B. C.; Urso, M. L.; Pierce, J. R.; Scofield, D. E.; Barnes, B. R.; Kraemer, W. J.; Anderson, J. M.; Maresh, C. M.; Beasley, K. N.; Zambraski, E. J.	2012	Did not perform fiber type analysis
The effects of weight loss on insulin sensitivity, skeletal muscle composition and capillary density in obese non-diabetic subjects	Niskanen, L.; Uusitupa, M.; Sarlund, H.; Siitonen, O.; Paljarvi, L.; Laakso, M.	1996	Does not include male and female data
ACTN3 genotype and modulation of skeletal muscle response to exercise in human subjects	Norman, B.; Esbjornsson, M.; Rundqvist, H.; Osterlund, T.; Glenmark, B.; Jansson, E.	2014	Same subjects as another study
Functional and biochemical properties of chronically stimulated human skeletal muscle	Nuhr, M.; Crevenna, R.; Gohlsch, B.; Bittner, C.; Pleiner, J.; Wiesinger, G.; Fialka-Moser, V.; Quittan, M.; Pette, D.	2003	Does not include males and females
Evidence of an increased number of type IIb muscle fibers in insulin-resistant first-degree relatives of patients with NIDDM	Nyholm, B.; Qu, Z. Q.; Kaal, A.; Pedersen, S. B.; Gravholt, C. H.; Andersen, J. L.; Saltin, B.; Schmitz, O.	1997	Does not include male and female data
Skeletal muscle malonyl-CoA content at the onset of exercise at varying power outputs in humans	Odland, L. M.; Howlett, R. A.; Heigenhauser, G. J. F.; Hultman, E.; Spriet, L. L.	1998	Did not perform fiber type analysis
Maintenance of skeletal muscle function following reduced daily physical activity in healthy older adults: a pilot trial	Oikawa, S. Y.; Callahan, D. M.; McGlory, C.; Toth, M. J.; Phillips, S. M.	2019	Does not include males and females
Adipokine gene expression in peripheral blood of adult and juvenile dermatomyositis patients and their relation to clinical parameters and disease activity measures	Olazagasti, J. M.; Hein, M.; Crowson, C. S.; de Padilla, C. L.; Peterson, E.; Baechler, E. C.; Reed, A. M.	2015	Did not perform fiber type analysis
Enhanced adiponectin multimer ratio and skeletal muscle adiponectin receptor expression following exercise training and diet in older insulin-resistant adults	O'Leary, V. B.; Jorett, A. E.; Marchetti, C. M.; Gonzalez, F.; Phillips, S. A.; Ciaraldi, T. P.; Kirwan, J. P.	2007	Did not perform fiber type analysis
Effects of short-term resistance training on muscle strength and morphology in the elderly	O'Neill, D. E. T.; Thayer, R. E.; Taylor, A. W.; Dzialoszynski, T. M.; Noble, E. G.	2000	Does not include male and female data
Effect of endurance exercise on myosin heavy chain gene regulation in human skeletal muscle	O'Neill, D. S.; Zheng, D. H.; Anderson, W. K.; Dohm, G. L.; Houmard, J. A.	1999	Does not include males and females
Effects of running distance and training on Ca2+ content and damage in human muscle	Overgaard, K.; Fredsted, A.; Hyldal, A.; Ingemann-Hansen, T.; Gissel, H.; Clausen, T.	2004	Does not include male and female data
Amino acid ingestion improves muscle protein synthesis in the young and elderly	Paddon-Jones, D.; Sheffield-Moore, M.; Zhang, X. J.; Volpi, E.; Wolf, S. E.; Aarsland, A.; Wolfe, R. R.	2004	Did not perform fiber type analysis

Antioxidant pathways in human aged skeletal muscle: relationship with the distribution of type II fibers	Pansarasa, O.; Felzani, G.; Vecchiet, J.; Marzatico, F.	2002	Does not include male and female data
Effects of resistance training on cardiovascular responses to lower body negative pressure in the elderly	Panton, L. B.; Franke, W. D.; Bleil, D. A.; Baier, S. M.; King, D. S.	2001	Does not include male and female data
Resistance exercise training decreases oxidative damage to DNA and increases cytochrome oxidase activity in older adults	Parise, G.; Brose, A. N.; Tarnopolsky, M. A.	2005	Did not perform fiber type analysis
Age and sex differences in human skeletal muscle fibrosis markers and transforming growth factor-beta signaling	Parker, L.; Caldow, M. K.; Watts, R.; Levinger, P.; Cameron-Smith, D.; Levinger, I.	2017	No cross-sectional area data
Plasma 25-Hydroxyvitamin D Is Related to Protein Signaling Involved in Glucose Homeostasis in a Tissue-Specific Manner	Parker, L.; Levinger, I.; Mousa, A.; Howlett, K.; de Courten, B.	2016	Did not perform fiber type
The effects of aging on enzyme activities and metabolite concentrations in skeletal muscle from sedentary male and female subjects	Pastoris, O.; Boschi, F.; Verri, M.; Baiardi, P.; Felzani, G.; Vecchiet, J.; Dossena, M.; Catapano, M.	2000	No healthy subjects or controls
GH replacement titrated to serum IGF-1 does not reduce concentrations of myostatin in blood or skeletal muscle	Paul, R. G.; McMahon, C. D.; Elston, M. S.; Conaglen, J. V.	2019	Did not perform fiber type analysis
Vitamin C and E supplementation hampers cellular adaptation to endurance training in humans: a double-blind, randomised, controlled trial	Paulsen, G.; Cumming, K. T.; Holden, G.; Hallen, J.; Ronnestad, B. R.; Sveen, O.; Skaug, A.; Paur, I.; Bastani, N. E.; Ostgaard, H. N.; Buer, C.; Midttun, M.; Freuchen, F.; Wiig, H.; Ulseth, E. T.; Garthe, I.; Blomhoff, R.; Benestad, H. B.; Raastad, T.	2014	Does not include male and female data
Vitamin C and E supplementation alters protein signalling after a strength training session, but not muscle growth during 10 weeks of training	Paulsen, G.; Hamarsland, H.; Cumming, K. T.; Johansen, R. E.; Hulmi, J. J.; Borsheim, E.; Wiig, H.; Garthe, I.; Raastad, T.	2014	Does not include male and female data
Improved recovery from skeletal muscle damage is largely unexplained by myofibrillar protein synthesis or inflammatory and regenerative gene expression pathways	Pavis, G. F.; Jameson, T. S. O.; Dirks, M. L.; Lee, B. P.; Abdelrahman, D. R.; Murton, A. J.; Porter, C.; Alamdari, N.; Mikus, C. R.; Wall, B. T.; Stephens, F. B.	2021	Did not perform fiber type analysis
Dysregulation of muscle glycogen synthase in recovery from exercise in type 2 diabetes	Pedersen, A. J. T.; Hingst, J. R.; Friedrichsen, M.; Kristensen, J. M.; Hojlund, K.; Wojtaszewski, J. F. P.	2015	Did not perform fiber type analysis
Micro-RNAs in ALS muscle: Differences in gender, age at onset and disease duration	Pegoraro, V.; Merico, A.; Angelini, C.	2017	Did not perform fiber type analysis
Muscle insulin receptor concentrations in obese patients post bariatric surgery: relationship to hyperinsulinemia	Pender, C.; Goldfine, I. D.; Tanner, C. J.; Pories, W. J.; MacDonald, K. G.; Havel, P. J.; Houmard, J. A.; Youngren, J. F.	2004	Did not perform fiber type analysis
Angiotensin-Converting Enzyme 2 (SARS-CoV-2 receptor) expression in human skeletal muscle	Perez-Valera, M.; Martinez-Canton, M.; Gallego-Selles, A.; Galvan-Alvarez, V.; Gelabert-Rebato, M.; Morales-Alamo, D.; Santana, A.; Martin-Rodriguez, S.; Ponce-Gonzalez, J. G.; Larsen, S.; Losa-Reyna, J.; Perez-Suarez, I.; Dorado, C.; Curtelin, D.; Gonzalez-Henriquez, J. J.; Boushel, R.; Hallen, J.; Velasco, P. D.; Freixinet-Gilart, J.;	2021	Did not perform fiber type analysis

	Holmberg, H. C.; Helge, J. W.; Martin-Rincon, M.; Calbet, J. A. L.		
Altered Intramuscular Lipid Metabolism Relates to Diminished Insulin Action in Men, but Not Women, in Progression to Diabetes	Perreault, L.; Bergman, B. C.; Hunerdosse, D. M.; Eckel, R. H.	2010	Did not perform fiber type analysis
Fenofibrate administration does not affect muscle triglyceride concentration or insulin sensitivity in humans	Perreault, L.; Bergman, B. C.; Hunerdosse, D. M.; Howard, D. J.; Eckel, R. H.	2011	Did not perform fiber type analysis
Gender differences in lipoprotein lipase activity after acute exercise	Perreault, L.; Lavely, J. M.; Kittelson, J. M.; Horton, T. J.	2004	Did not perform fiber type analysis
Intracellular localization of diacylglycerols and sphingolipids influences insulin sensitivity and mitochondrial function in human skeletal muscle	Perreault, L.; Newsom, S. A.; Strauss, A.; Kerege, A.; Kahn, D. E.; Harrison, K. A.; Snell-Bergeon, J. K.; Nemkov, T.; D'Alessandro, A.; Jackman, M. R.; MacLean, P. S.; Bergman, B. C.	2018	Did not perform fiber type analysis
Exercise-induced protein kinase C isoform-specific activation in human skeletal muscle	Perrini, S.; Henriksson, J.; Zierath, J. R.; Widegren, U.	2004	Did not perform fiber type analysis
Elevated levels of IL-18 in plasma and skeletal muscle in chronic obstructive pulmonary disease	Petersen, A. M. W.; Penkowa, M.; Iversen, M.; Frydelund-Larsen, L.; Andersen, J. L.; Mortensen, J.; Lange, P.; Pedersen, B. K.	2007	Does not include male and female data
Efficacy of myonuclear addition may explain differential myofiber growth among resistance-trained young and older men and women	Petrella, J. K.; Kim, J. S.; Cross, J. M.; Kosek, D. J.; Bamman, M. M.	2006	Does not include male and female data
Potent myofiber hypertrophy during resistance training in humans is associated with satellite cell-mediated myonuclear addition: a cluster analysis	Petrella, J. K.; Kim, J. S.; Mayhew, D. L.; Cross, J. M.; Bamman, M. M.	2008	Does not report sex of subjects
Advanced Age Is Associated with Iron Dyshomeostasis and Mitochondrial DNA Damage in Human Skeletal Muscle	Picca, A.; Mankowski, R. T.; Kamenov, G.; Anton, S. D.; Manini, T. M.; Buford, T. W.; Saini, S. K.; Calvani, R.; Landi, F.; Bernabei, R.; Marzetti, E.; Leeuwenburgh, C.	2019	Did not perform fiber type analysis
Skeletal muscle adaptations to exercise are not influenced by metformin treatment in humans: secondary analyses of 2 randomized, clinical trials	Pilmark, N. S.; Oberholzer, L.; Halling, J. F.; Kristensen, J. M.; Bonding, C. P.; Elkjaer, I.; Lyngbaek, M.; Elster, G.; Siebenmann, C.; Holm, N. F. R.; Birk, J. B.; Larsen, E. L.; Lundby, A. K. M.; Wojtaszewski, J.; Pilegaard, H.; Poulsen, H. E.; Pedersen, B. K.; Hansen, K. B.; Karstoft, K.	2022	Does not include males and females
Skeletal muscle telomere length is not impaired in healthy physically active old women and men	Ponsot, E.; Lexiell, J.; Kadi, F.	2008	Did not perform fiber type analysis
Mitochondrial respiratory capacity and coupling control decline with age in human skeletal muscle	Porter, C.; Hurren, N. M.; Cotter, M. V.; Bhattarai, N.; Reidy, P. T.; Dillon, E. L.; Durham, W. J.; Tuvdendorj, D.; Sheffield-Moore, M.; Volpi, E.; Sidossis, L. S.; Rasmussen, B. B.; Borsheim, E.	2015	Did not perform fiber type analysis
Expression of Myosin Heavy Chain Isoforms in the Human Supraspinatus Muscle: Variations Related to Age and Sex	Potau, J. M.; Artells, R.; Munoz, C.; Diaz, T.; Bello-Hellegouarch, G.; Arias-Martorell, J.; Perez-Perez, A.; Monzo, M.	2012	Autopsies

Impaired Angiogenesis and Peripheral Muscle Microcirculation Loss Contribute to Exercise Intolerance in Pulmonary Arterial Hypertension	Potus, F.; Malenfant, S.; Graydon, C.; Mainguy, V.; Tremblay, E.; Breuils-Bonnet, S.; Ribeiro, F.; Porlier, A.; Maltais, F.; Bonnet, S.; Provencher, S.	2014	Does not include male and female data
Human neuromuscular structure and function in old age: A brief review	Power, G. A.; Dalton, B. H.; Rice, C. L.	2013	Review (ex: systematic review/meta-analysis)
Reduction in single muscle fiber rate of force development with aging is not attenuated in world class older masters athletes	Power, G. A.; Minozzo, F. C.; Spendiff, S.; Filion, M. E.; Konokhova, Y.; Purves-Smith, M. F.; Pion, C.; Aubertin-Leheudre, M.; Morais, J. A.; Herzog, W.; Hepple, R. T.; Taivassalo, T.; Rassier, D. E.	2016	Does not include males and females
Oxygen uptake kinetics during moderate, heavy and severe intensity 'submaximal' exercise in humans: the influence of muscle fibre type and capillarisation	Pringle, J. S. M.; Doust, J. H.; Carter, H.; Tolfrey, K.; Campbell, I. T.; Jones, A. M.; Sakkas, G. K.	2003	Does not include male and female data
Increased Skeletal Muscle Capillarization After Aerobic Exercise Training andWeight Loss Improves Insulin Sensitivity in Adults With IGT	Prior, S. J.; Blumenthal, J. B.; Katzel, L. I.; Goldberg, A. P.; Ryan, A. S.	2014	Does not include male and female data
Increased Skeletal Muscle Capillarization Independently Enhances Insulin Sensitivity in Older Adults After Exercise Training and Detraining	Prior, S. J.; Goldberg, A. P.; Ortmeyer, H. K.; Chin, E. R.; Chen, D. P.; Blumenthal, J. B.; Ryan, A. S.	2015	Does not include male and female data
Reduced Skeletal Muscle Capillarization and Glucose Intolerance	Prior, S. J.; McKenzie, M. J.; Joseph, L. J.; Ivey, F. M.; Macko, R. F.; Hafer-Macko, C. E.; Ryan, A. S.	2009	Does not include male and female data
Exercise training increases intramyocellular lipid and oxidative capacity in older adults	Pruchnic, R.; Katsiaras, A.; He, J.; Kelley, D. E.; Winters, C.; Goodpaster, B. H.	2004	Does not include male and female data
Effects of training, detraining, and retraining on strength, hypertrophy, and myonuclear number in human skeletal muscle	Psilander, N.; Eftestol, E.; Cumming, K. T.; Juvkam, I.; Ekblom, M. M.; Sunding, K.; Wernbom, M.; Holmberg, H. C.; Ekblom, B.; Bruusgaard, J. C.; Raastad, T.; Gundersen, K.	2019	Does not include male and female data
Exercise stress leads to an acute loss of mitochondrial proteins and disruption of redox control in skeletal muscle of older subjects: An underlying decrease in resilience with aging?	Pugh, J. N.; Stretton, C.; McDonagh, B.; Brownridge, P.; McArdle, A.; Jackson, M. J.; Close, G. L.	2021	Did not perform fiber type analysis
The profile and distribution of myosin heavy chain isoforms in middle-aged sedentary persons	Puhke, R.; Aunola, S.; Surakka, J.; Venojarvi, M.; Alev, K.; Seene, T.; Rusko, H.	2006	Does not include male and female data
Metabolic phenotype of skeletal muscle in early critical illness	Puthucheary, Z. A.; Astin, R.; McPhail, M. J. W.; Saeed, S.; Pasha, Y.; Bear, D. E.; Constantin, D.; Velloso, C.; Manning, S.; Calvert, L.; Singer, M.; Batterham, R. L.; Gomez-Romero, M.; Holmes, E.; Steiner, M. C.; Atherton, P. J.; Greenhaff, P.; Edwards, L. M.; Smith, K.; Harridge, S. D.; Hart, N.; Montgomery, H. E.	2018	Did not perform fiber type analysis

Effects of short-term submaximal training in humans on muscle metabolism in exercise	Putman, C. T.; Jones, N. L.; Hultman, E.; Hollidge-Horvat, M. G.; Bonen, A.; McConachie, D. R.; Heigenhauser, G. J. F.	1998	Does not include males and females
Effects of strength, endurance and combined training on myosin heavy chain content and fibre-type distribution in humans	Putman, C. T.; Xu, X.; Gillies, E.; MacLean, I. M.; Bell, G. J.	2004	Does not include male and female data
MUSCLE STRENGTH AND FIBER ADAPTATIONS TO A YEAR-LONG RESISTANCE TRAINING-PROGRAM IN ELDERLY MEN AND WOMEN	Pyka, G.; Lindenberger, E.; Charette, S.; Marcus, R.	1994	Does not include male and female data
Improved glycaemic control decreases inner mitochondrial membrane leak in type 2 diabetes	Rabol, R.; Hojberg, P. M. V.; Almdal, T.; Boushel, R.; Haugaard, S. B.; Madsbad, S.; Dela, F.	2009	Did not perform fiber type analysis
Insulin-Stimulated Muscle Glucose Uptake and Insulin Signaling in Lean and Obese Humans	Ramos, P. A.; Lytle, K. A.; Delivanis, D.; Nielsen, S.; LeBrasseur, N. K.; Jensen, M. D.	2021	Did not perform fiber type analysis
Aspirin as a COX inhibitor and anti-inflammatory drug in human skeletal muscle	Ratchford, S. M.; Lavin, K. M.; Perkins, R. K.; Jemiolo, B.; Trappe, S. W.; Trappe, T. A.	2017	Did not perform fiber type analysis
Transcriptome signature of resistance exercise adaptations: mixed muscle and fiber type specific profiles in young and old adults	Raue, U.; Trappe, T. A.; Estrem, S. T.; Qian, H. R.; Helvering, L. M.; Smith, R. C.; Trappe, S.	2012	Does not include male and female data
A data-driven methodology reveals novel myofiber clusters in older human muscles	Raz, Y.; van den Akker, E. B.; Roest, T.; Riaz, M.; van de Rest, O.; Suchiman, H. E. D.; Lakenberg, N.; Stassen, S. A.; van Putten, M.; Feskens, E. J. M.; Reinders, M. J. T.; Goeman, J.; Beekman, M.; Raz, V.; Slagboom, P. E.	2020	No cross-sectional area data
Endurance performance in masters athletes	Reaburn, P.; Dascombe, B.	2008	Review (ex: systematic review/meta-analysis)
Myostatin, insulin-like growth factor-1, and leukemia inhibitory factor mRNAs are upregulated in chronic human disuse muscle atrophy	Reardon, K. A.; Davis, J.; Kapsa, R. M. I.; Choong, P.; Byrne, E.	2001	Does not include male and female data
Muscle diffusion tensor imaging in glycogen storage disease V (McArdle disease)	Rehmann, R.; Schlaffke, L.; Froeling, M.; Kley, R. A.; Kuhnle, E.; De Marees, M.; Forsting, J.; Rohm, M.; Tegenthoff, M.; Schmidt-Wilcke, T.; Vorgerd, M.	2019	Did not perform fiber type analysis
Muscle power failure in mobility-limited older adults: preserved single fiber function despite lower whole muscle size, quality and rate of neuromuscular activation	Reid, K. F.; Doros, G.; Clark, D. J.; Patten, C.; Carabello, R. J.; Cloutier, G. J.; Phillips, E. M.; Krivickas, L. S.; Frontera, W. R.; Fielding, R. A.	2012	Single Fiber Analysis
Protein Composition of Endurance Trained Human Skeletal Muscle	Reidy, P. T.; Hinkley, J. M.; Trappe, T. A.; Trappe, S. W.; Harber, M. P.	2014	Does not report sex of subjects
Soy-dairy protein blend and whey protein ingestion after resistance exercise increases amino acid transport and transporter expression in human skeletal muscle	Reidy, P. T.; Walker, D. K.; Dickinson, J. M.; Gundermann, D. M.; Drummond, M. J.; Timmerman, K. L.; Cope, M. B.; Mukherjea, R.; Jennings, K.; Volpi, E.; Rasmussen, B. B.	2014	Does not report sex of subjects
Protein Blend Ingestion Following Resistance Exercise Promotes Human Muscle Protein Synthesis	Reidy, P. T.; Walker, D. K.; Dickinson, J. M.; Gundermann, D. M.; Drummond, M. J.; Timmerman, K. L.; Fry, C. S.; Borack, M. S.;	2013	Did not perform fiber type analysis

	Cope, M. B.; Mukherjea, R.; Jennings, K.; Volpi, E.; Rasmussen, B. B.		
Expression of striated activator of rho-signaling in human skeletal muscle following acute exercise and long-term training	Reitzner, S. M.; Norrbom, J.; Sundberg, C. J.; Gidlund, E. K.	2018	Did not perform fiber type analysis
Nicotinamide riboside supplementation alters body composition and skeletal muscle acetylcarnitine concentrations in healthy obese humans	Remie, C. M. E.; Roumans, K. H. M.; Moonen, M. P. B.; Connell, N. J.; Havekes, B.; Mevenkamp, J.; Lindeboom, L.; de Wit, V. H. W.; van de Weijer, T.; Aarts, Sabm; Lutgens, E.; Schomakers, B. V.; Elfrink, H. L.; Zapata-Perez, R.; Houtkooper, R. H.; Auwerx, J.; Hoeks, J.; Schrauwen-Hinderling, V. B.; Phielix, E.; Schrauwen, P.	2020	Did not perform fiber type analysis
ANGIOTENSIN-CONVERTING ENZYME IN HUMAN SKELETAL-MUSCLE - A SIMPLE IN-VITRO ASSAY OF ACTIVITY IN NEEDLE-BIOPSY SPECIMENS	Reneland, R.; Lithell, H.	1994	Did not perform fiber type analysis
Automated image-analysis method for the quantification of fiber morphometry and fiber type population in human skeletal muscle	Reyes-Fernandez, P. C.; Periou, B.; Decrouy, X.; Relaix, F.; Authier, F. J.	2019	Does not include male and female data
Mitochondrial electron transport chain function is enhanced in inspiratory muscles of patients with chronic obstructive pulmonary disease	Ribera, F.; N'Guessan, B.; Zoll, J.; Fortin, D.; Serrurier, B.; Mettauer, B.; Bigard, X.; Ventura-Clapier, R.; Lampert, E.	2003	No healthy subjects or controls
Hypobaric live high-train low does not improve aerobic performance more than live low-train low in cross-country skiers	Robach, P.; Hansen, J.; Pichon, A.; Lundby, A. K. M.; Dandanell, S.; Falch, G. S.; Hammarstrom, D.; Pesta, D. H.; Siebenmann, C.; Keiser, S.; Kerivel, P.; Whist, J. E.; Ronnestad, B. R.; Lundby, C.	2018	Did not perform fiber type analysis
Relationship between leg muscle capillary density and peak hyperemic blood flow with endurance capacity in peripheral artery disease	Robbins, J. L.; Jones, W. S.; Duscha, B. D.; Allen, J. D.; Kraus, W. E.; Regensteiner, J. G.; Hiatt, W. R.; Annex, B. H.	2011	Did not perform fiber type analysis
Myogenic mRNA markers in young and old human skeletal muscle prior to and following sequential exercise bouts	Roberts, M. D.; Dalbo, V. J.; Sunderland, K.; Poole, C.; Hassell, S. E.; Kerksick, C. M.	2011	Does not include males and females
Long-term synthesis rates of skeletal muscle DNA and protein are higher during aerobic training in older humans than in sedentary young subjects but are not altered by protein supplementation	Robinson, M. M.; Turner, S. M.; Hellerstein, M. K.; Hamilton, K. L.; Miller, B. F.	2011	Did not perform fiber type analysis
Gender differences in substrate utilization during submaximal exercise in endurance-trained subjects	Roepstorff, C.; Steffensen, C. H.; Madsen, M.; Stallknecht, B.; Kanstrup, I. L.; Richter, E. A.; Kiens, B.	2002	Did not perform fiber type analysis
Effect of age on in vivo rates of mitochondrial protein synthesis in human skeletal muscle	Rooyackers, O. E.; Adey, D. B.; Ades, P. A.; Nair, K. S.	1996	Did not perform fiber type analysis
Measurement of synthesis rates of specific muscle proteins using needle biopsy samples	Rooyackers, O. E.; Balagopal, P.; Nair, K. S.	1997	Does not report sex of subjects
Effect of eccentric action velocity on expression of genes related to myostatin signaling pathway in human skeletal muscle	Roschel, H.; Ugrinowistch, C.; Santos, A. R.; Barbosa, W. P.; Miyabara, E. H.; Tricoli, V.; Aoki, M. S.	2018	Does not include males and females
Influence of age, sex, and strength training on human muscle gene expression determined by microarray	Roth, S. M.; Ferrell, R. E.; Peters, D. G.; Metter, E. J.; Hurley, B. F.; Rogers, M. A.	2002	Did not perform fiber type analysis

Myostatin gene expression is reduced in humans with heavy resistance strength training: A brief communication	Roth, S. M.; Martel, G. F.; Ferrell, R. E.; Metter, E. J.; Hurley, B. F.; Rogers, M. A.	2003	Did not perform fiber type analysis
Skeletal muscle satellite cell populations in healthy young and older men and women	Roth, S. M.; Martel, G. F.; Ivey, F. M.; Lemmer, J. T.; Metter, E. J.; Hurley, B. F.; Rogers, M. A.	2000	Did not perform fiber type analysis
Skeletal muscle satellite cell characteristics in young and older men and women after heavy resistance strength training	Roth, S. M.; Martel, G. F.; Ivey, F. M.; Lemmer, J. T.; Tracy, B. L.; Metter, E. J.; Hurley, B. F.; Rogers, M. A.	2001	Does not include male and female data
Muscle biopsy and muscle fiber hypercontraction: a brief review	Roth, S. M.; Martel, G. F.; Rogers, M. A.	2000	Review (ex: systematic review/meta-analysis)
Factors Influencing Substrate Oxidation During Submaximal Cycling: A Modelling Analysis	Rothschild, J. A.; Kilding, A. E.; Stewart, T.; Plews, D. J.	2022	Review (ex: systematic review/meta-analysis)
Multi-omic integrated networks connect DNA methylation and miRNA with skeletal muscle plasticity to chronic exercise in Type 2 diabetic obesity	Rowlands, D. S.; Page, R. A.; Sukala, W. R.; Giri, M.; Ghimbovschi, S. D.; Hayat, I.; Cheema, B. S.; Lys, I.; Leikis, M.; Sheard, P. W.; Wakefield, S. J.; Breier, B.; Hathout, Y.; Brown, K.; Marathi, R.; Orkunoglu-Suer, F. E.; Devaney, J. M.; Leiken, B.; Many, G.; Krebs, J.; Hopkins, W. G.; Hoffman, E. P.	2014	Did not perform fiber type analysis
GENDER DIFFERENCES IN SUBSTRATE UTILIZATION DURING EXERCISE	Ruby, B. C.; Robergs, R. A.	1994	Review (ex: systematic review/meta-analysis)
Histologic Differences in Human Rotator Cuff Muscle Based on Tear Characteristics	Ruderman, L.; Leinroth, A.; Rueckert, H.; Tabarestani, T.; Baker, R.; Levin, J.; Cook, C. E.; Klifto, C. S.; Hilton, M. J.; Anakwenze, O.	2022	Did not perform fiber type analysis
Influence of nutrient ingestion on amino acid transporters and protein synthesis in human skeletal muscle after sprint exercise	Rundqvist, H. C.; Esbjornsson, M.; Rooyackers, O.; Osterlund, T.; Moberg, M.; Apro, W.; Blomstrand, E.; Jansson, E.	2017	Did not perform fiber type analysis
Acute sprint exercise transcriptome in human skeletal muscle	Rundqvist, H. C.; Montelius, A.; Osterlund, T.; Norman, B.; Esbjornsson, M.; Jansson, E.	2019	Does not include male and female data
Regulation of skeletal muscle sucrose, non-fermenting 1/AMP-activated protein kinase-related kinase (SNARK) by metabolic stress and diabetes	Rune, A.; Osler, M. E.; Fritz, T.; Zierath, J. R.	2009	Did not perform fiber type analysis
Evidence against a sexual dimorphism in glucose and fatty acid metabolism in skeletal muscle cultures from age-matched men and post-menopausal women	Rune, A.; Salehzadeh, F.; Szekeres, F.; Kuhn, I.; Osler, M. E.; Al-Khalili, L.	2009	Review (ex: systematic review/meta-analysis)
Brown adipocyte progenitor population is modified in obese and diabetic skeletal muscle	Russell, A. P.; Crisan, M.; Leger, B.; Corselli, M.; McAinch, A. J.; O'Brien, P. E.; Cameron-Smith, D.; Peault, B.; Casteilla, L.; Giacobino, J. P.	2012	Did not perform fiber type analysis
CHANGES IN SKELETAL-MUSCLE AND BODY-COMPOSITION AFTER DISCONTINUATION OF	Rutherford, O. M.; Jones, D. A.; Round, J. M.; Buchanan, C. R.; Preece, M. A.	1991	Only in children (0-17 years)

GROWTH-HORMONE TREATMENT IN GROWTH-HORMONE DEFICIENT YOUNG-ADULTS			
Aberrant Mitochondrial Homeostasis in the Skeletal Muscle of Sedentary Older Adults	Safdar, A.; Hamadeh, M. J.; Kaczor, J. J.; Raha, S.; Debeer, J.; Tarnopolsky, M. A.	2010	Did not perform fiber type analysis
Mitochondrial DNA damage in calf skeletal muscle and walking performance in people with peripheral artery disease	Saini, S. K.; McDermott, M. M.; Picca, A.; Li, L. Y.; Wohlgemuth, S. E.; Kosmac, K.; Peterson, C. A.; Tian, L.; Ferrucci, L.; Guralnik, J. M.; Sufit, R. L.; Leeuwenburgh, C.	2020	Did not perform fiber type analysis
Atypical fast SERCA1a protein expression in slow myofibers and differential S-nitrosylation prevented by exercise during long term bed rest	Salanova, M.; Schiffl, G.; Blottner, D.	2009	Does not include males and females
Early resistance training-mediated stimulation of daily muscle protein synthetic responses to higher habitual protein intake in middle-aged adults	Salvador, A. F.; McKenna, C. F.; Paulussen, K. J. M.; Keeble, A. R.; Askow, A. T.; Fang, H. Y.; Li, Z.; Ulanov, A. V.; Paluska, S. A.; Moore, D. R.; Burd, N. A.	2021	Did not perform fiber type analysis
Sex differences in skeletal muscle Phosphatase and tensin homolog deleted on chromosome 10 (PTEN) levels: A cross-sectional study	Samaan, M. C.; Anand, S. S.; Sharma, A. M.; Samjoo, I. A.; Tarnopolsky, M. A.	2015	Did not perform fiber type analysis
Overfeeding Reduces Insulin Sensitivity and Increases Oxidative Stress, without Altering Markers of Mitochondrial Content and Function in Humans	Samocha-Bonet, D.; Campbell, L. V.; Mori, T. A.; Croft, K. D.; Greenfield, J. R.; Turner, N.; Heilbronn, L. K.	2012	Did not perform fiber type analysis
Activating brown adipose tissue through exercise (ACTIBATE) in young adults: Rationale, design and methodology	Sanchez-Delgado, G.; Martinez-Tellez, B.; Olza, J.; Aguilera, C. M.; Labayen, I.; Ortega, F. B.; Chillon, P.; Fernandez-Reguera, C.; Alcantara, J. M. A.; Martinez-Avila, W. D.; Munoz-Hernandez, V.; Acosta, F. M.; Prados-Ruiza, J.; Amaro-Gallete, F. J.; Hidalgo-Garcia, L.; Rodriguez, L.; Ruiz, Y. A. K.; Ramirez-Navarro, A.; Muros-de Fuentes, M. A.; Garcia-Rivero, Y.; Sanchez-Sanchez, R.; Jimenez, J. D. B.; de Teresa, C.; Navarrete, S.; Lozano, R.; Brea-Gomez, E.; Rubio-Lopez, J.; Ruiz, M. R.; Cano-Nieto, A.; Llamas-Elvira, J. M.; Rios, J. A. J.; Gil, A.; Ruiz, J. R.	2015	Did not perform fiber type analysis
Quantitative analysis of proteins of metabolism by reverse phase protein microarrays identifies potential biomarkers of rare neuromuscular diseases	Santacatterina, F.; Chamorro, M.; de Arenas, C. N.; Navarro, C.; Martin, M. A.; Cuezva, J. M.; Sanchez-Arago, M.	2015	Did not perform fiber type analysis
Skeletal muscle changes in patients with chronic heart failure before and after treatment with enalapril	Schaufelberger, M.; Andersson, G.; Eriksson, B. O.; Grimby, G.; Held, P.; Swedberg, K.	1996	Does not include male and female data
MSTN mRNA after Varying Exercise Modalities in Humans	Schiffer, T.; Geisler, S.; Sperlich, B.; Struder, H. K.	2011	Does not report sex of subjects
Myosin heavy chain isoform expression and in vivo isometric performance: A regression model	Schilling, B. K.; Fry, A. C.; Chiu, L. Z. F.; Weiss, L. W.	2005	Does not include males and females

HYPERTROPHIC EFFECTS OF CONCENTRIC VS. ECCENTRIC MUSCLE ACTIONS: A SYSTEMATIC REVIEW AND META-ANALYSIS	Schoenfeld, B. J.; Ogborn, D. I.; Vigotsky, A. D.; Franchi, M. V.; Krieger, J. W.	2017	Review (ex: systematic review/meta-analysis)
Insulin and Fiber Type in the Offspring of T2DM Subjects with Resistance Training and Detraining	Schofield, K. L.; Rehrer, N. J.; Perry, T. L.; Ross, A.; Andersen, J. L.; Osborne, H.	2012	Does not include male and female data
Fiber type dependent upregulation of human skeletal muscle UCP2 and UCP3 mRNA expression by high-fat diet	Schrauwen, P.; Hoppeler, H.; Billeter, R.; Bakker, A. H. F.; Pendergast, D. R.	2001	Does not include male and female data
Effects of resistance training on the rate of muscle protein synthesis in frail elderly people	Schulte, J. N.; Yarasheski, K. E.	2001	Did not perform fiber type analysis
Fibre-Specific Responses to Endurance and Low Volume High Intensity Interval Training: Striking Similarities in Acute and Chronic Adaptation	Scribbans, T. D.; Edgett, B. A.; Vorobej, K.; Mitchell, A. S.; Joanisse, S. D.; Matusiak, J. B. L.; Parise, G.; Quadrilatero, J.; Gurd, B. J.	2014	Does not include male and female data
Induction of human skeletal muscle lipoprotein lipase gene expression by short-term exercise is transient	Seip, R. L.; Mair, K.; Cole, T. G.; Semenkovich, C. F.	1997	Did not perform fiber type analysis
Glucose transporter expression in skeletal muscle of endurance-trained individuals	Seki, Y.; Berggren, J. R.; Houmard, J. A.; Charron, M. J.	2006	Did not perform fiber type analysis
PHYSIOLOGICAL, METABOLIC, AND MUSCLE-FIBER TYPE CHARACTERISTICS OF MUSCULUS UVULAE IN SLEEP-APNEA HYPOPNEA SYNDROME AND IN SNORERS	Series, F.; Cote, C.; Simoneau, J. A.; Gelinas, Y.; Stpierre, S.; Leclerc, J.; Ferland, R.; Marc, I.	1995	Does not include male and female data
Characteristics of the genioglossus and musculus uvulae in sleep apnea hypopnea syndrome and in snorers	Series, F.; Simoneau, J. A.; StPierre, S.; Marc, I.	1996	Does not include male and female data
Repeated Sprints Alter Signaling Related to Mitochondrial Biogenesis in Humans	Serpiello, F. R.; McKenna, M. J.; Bishop, D. J.; Aughey, R. J.; Caldow, M. K.; Cameron-Smith, D.; Stepto, N. K.	2012	Did not perform fiber type analysis
Lack of Increase in Muscle Mitochondrial Protein Synthesis During the Course of Aerobic Exercise and Its Recovery in the Fasting State Irrespective of Obesity	Serrano, N.; Tran, L.; Hoffman, N.; Roust, L.; De Filippis, E. A.; Carroll, C. C.; Patel, S. H.; Kras, K. A.; Buras, M.; Katsanos, C. S.	2021	Did not perform fiber type analysis
Effects of Peanut Protein Supplementation on Resistance Training Adaptations in Younger Adults	Sexton, C. L.; Smith, M. A.; Smith, K. S.; Osburn, S. C.; Godwin, J. S.; Ruple, B. A.; Hendricks, A. M.; Mobley, C. B.; Goodlett, M. D.; Fruge, A. D.; Young, K. C.; Roberts, M. D.	2021	Does not include male and female data
Fructose overfeeding in first-degree relatives of type 2 diabetic patients impacts energy metabolism and mitochondrial functions in skeletal muscle	Seyssel, K.; Meugnier, E.; Le, K. A.; Durand, C.; Disse, E.; Blond, E.; Pays, L.; Nataf, S.; Brozek, J.; Vidal, H.; Tappy, L.; Laville, M.	2016	Does not include males and females
MORPHOMETRIC STUDIES OF NORMAL MUSCLE MITOCHONDRIA	Shah, A.; Sahgal, V.	1991	Does not report sex of subjects
Unique expression of cytoskeletal proteins in human soft palate muscles	Shah, F.; Berggren, D.; Holmlund, T.; Jaghagen, E. L.; Stal, P.	2016	Autopsies

Mitochondrial DNA deletion and sarcopenia	Shah, V. O.; Scariano, J.; Waters, D.; Qualls, C.; Morgan, M.; Pickett, G.; Gasparovic, C.; Dokladny, K.; Moseley, P.; Raj, D. S. C.	2009	Did not perform fiber type analysis
Lumbar multifidus muscle degenerates in individuals with chronic degenerative lumbar spine pathology	Shahidi, B.; Hubbard, J. C.; Gibbons, M. C.; Ruoss, S.; Zlomislic, V.; Allen, R. T.; Garfin, S. R.; Ward, S. R.	2017	No healthy subjects or controls
Sex differences in COPD-related quadriceps muscle dysfunction and fibre abnormalities	Sharanya, A.; Ciano, M.; Withana, S.; Kemp, P. R.; Polkey, M. I.; Sathyapala, S. A.	2019	Data reported in median and IQR
Combining short-term metformin treatment and one bout of exercise does not increase insulin action in insulin-resistant individuals	Sharoff, C. G.; Hagobian, T. A.; Malin, S. K.; Chipkin, S. R.; Yu, H. Y.; Hirshman, M. F.; Goodyear, L. J.; Braun, B.	2010	Did not perform fiber type analysis
Enhancement of muscle mitochondrial function by growth hormone	Short, K. R.; Moller, N.; Bigelow, M. L.; Coenen-Schimke, J.; Nair, K. S.	2008	Did not perform fiber type analysis
Effect of short-term prednisone use on blood flow, muscle protein metabolism, and function	Short, K. R.; Nygren, J.; Bigelow, M. L.; Nair, K. S.	2004	Did not perform fiber type analysis
Changes in myosin heavy chain mRNA and protein expression in human skeletal muscle with age and endurance exercise training	Short, K. R.; Vittone, J. L.; Bigelow, M. L.; Proctor, D. N.; Coenen-Schimke, J. M.; Rys, P.; Nair, K. S.	2005	Intervention with no baseline data
Extracellular vesicular miRNA expression is not a proxy for skeletal muscle miRNA expression in males and females following acute, moderate intensity exercise	Silver, J. L.; Alexander, S. E.; Dillon, H. T.; Lamon, S.; Wadley, G. D.	2020	Did not perform fiber type analysis
Impaired Muscle Mitochondrial Function in Familial Partial Lipodystrophy	Simha, V.; Lanza, I. R.; Dasari, S.; Klaus, K. A.; Le Brasseur, N.; Vuckovic, I.; Laurenti, M. C.; Cobelli, C.; Port, J. D.; Nair, K. S.	2022	Did not perform fiber type analysis
GENETIC DETERMINISM OF FIBER-TYPE PROPORTION IN HUMAN SKELETAL-MUSCLE	Simoneau, J. A.; Bouchard, C.	1995	Review (ex: systematic review/meta-analysis)
Altered glycolytic and oxidative capacities of skeletal muscle contribute to insulin resistance in NIDDM	Simoneau, J. A.; Kelley, D. E.	1997	Did not perform fiber type analysis
Markers of capacity to utilize fatty acids in human skeletal muscle: relation to insulin resistance and obesity and effects of weight loss	Simoneau, J. A.; Veerkamp, J. H.; Turcotte, L. P.; Kelley, D. E.	1999	Did not perform fiber type analysis
Noninvasive Estimation of Myosin Heavy Chain Composition in Human Skeletal Muscle	Simunic, B.; Degens, H.; Rittweger, J.; Narici, M.; Mekjavic, I. B.; Pisot, R.	2011	Does not include male and female data
Insulin-like growth factor I in skeletal muscle after weight-lifting exercise in frail elders	Singh, M. A. F.; Ding, W. J.; Manfredi, T. J.; Solares, G. S.; O'Neill, E. F.; Clements, K. M.; Ryan, N. D.; Kehayias, J. J.; Fielding, R. A.; Evans, W. J.	1999	Does not include male and female data
Effect of increased and maintained frequency of speed endurance training on performance and muscle adaptations in runners	Skovgaard, C.; Almquist, N. W.; Bangsbo, J.	2017	Does not include male and female data
Effect of tapering after a period of high-volume sprint interval training on running performance and muscular adaptations in moderately trained runners	Skovgaard, C.; Almquist, N. W.; Kvorning, T.; Christensen, P. M.; Bangsbo, J.	2018	Does not include male and female data

Effect of speed endurance training and reduced training volume on running economy and single muscle fiber adaptations in trained runners	Skovgaard, C.; Christiansen, D.; Christensen, P. M.; Almquist, N. W.; Thomassen, M.; Bangsbo, J.	2018	Does not include male and female data
Age-RelatedAnabolic Resistance of Myofibrillar Protein Synthesis Is Exacerbated in Obese Inactive Individuals	Smeuninx, B.; McKendry, J.; Wilson, D.; Martin, U.; Breen, L.	2017	Does not include male and female data
Modulation of autophagy signaling with resistance exercise and protein ingestion following short-term energy deficit	Smiles, W. J.; Areta, J. L.; Coffey, V. G.; Phillips, S. M.; Moore, D. R.; Stellingwerff, T.; Burke, L. M.; Hawley, J. A.; Camera, D. M.	2015	Did not perform fiber type analysis
Timing of the initial muscle biopsy does not affect the measured muscle protein fractional synthesis rate during basal, postabsorptive conditions	Smith, G. I.; Villareal, D. T.; Lambert, C. P.; Reeds, D. N.; Mohammed, B. S.; Mittendorfer, B.	2010	Does not include males and females
Sex-specific alterations in mRNA level of key lipid metabolism enzymes in skeletal muscle of overweight and obese subjects following endurance exercise	Smith, I. J.; Huffman, K. M.; Durheim, M. T.; Duscha, B. D.; Kraus, W. E.	2009	Did not perform fiber type analysis
Three weeks of interrupting sitting lowers fasting glucose and glycemic variability, but not glucose tolerance, in free-living women and men with obesity	Smith, J. A. B.; Savikj, M.; Sethi, P.; Platt, S.; Gabriel, B. M.; Hawley, J. A.; Dunstan, D.; Krook, A.; Zierath, J. R.; Naslund, E.	2021	Did not perform fiber type analysis
Muscle mass and strength gains following 6 months of resistance type exercise training are only partly preserved within one year with autonomous exercise continuation in older adults	Snijders, T.; Leenders, M.; de Groot, Lcpgm; van Loon, L. J. C.; Verdijk, L. B.	2019	Does not report sex of subjects
The impact of sarcopenia and exercise training on skeletal muscle satellite cells	Snijders, T.; Verdijk, L. B.; van Loon, L. J. C.	2009	Review (ex: systematic review/meta-analysis)
Key Components of Human Myofibre Denervation and Neuromuscular Junction Stability are Modulated by Age and Exercise	Soendenbroe, C.; Bechshoft, C. J. L.; Heisterberg, M. F.; Jensen, S. M.; Bomme, E.; Schjerling, P.; Karlsen, A.; Kjaer, M.; Andersen, J. L.; Mackey, A. L.	2020	Does not include male and female data
Preserved stem cell content and innervation profile of elderly human skeletal muscle with lifelong recreational exercise	Soendenbroe, C.; Dahl, C. L.; Meulengracht, C.; Tamas, M.; Svensson, R. B.; Schjerling, P.; Kjaer, M.; Andersen, J. L.; Mackey, A. L.	2022	Does not include males and females
Muscle adaptation to short-term fasting in healthy lean humans	Soeters, M. R.; Sauerwein, H. P.; Dubbelhuis, P. F.; Groener, J. E.; Ackermans, M. T.; Fliers, E.; Aerts, J. M.; Serlie, M. J.	2008	Does not include males and females
High-intensity interval training improves insulin sensitivity in older individuals	Sogaard, D.; Lund, M. T.; Scheuer, C. M.; Dehlbaek, M. S.; Dideriksen, S. G.; Abildskov, C. V.; Christensen, K. K.; Dohlmann, T. L.; Larsen, S.; Vigelso, A. H.; Dela, F.; Helge, J. W.	2018	Did not perform fiber type analysis
Progressive Hyperglycemia across the Glucose Tolerance Continuum in Older Obese Adults Is Related to Skeletal Muscle Capillarization and Nitric Oxide Bioavailability	Solomon, T. P. J.; Haus, J. M.; Li, Y. J.; Kirwan, J. P.	2011	Does not include male and female data

Lipoprotein lipase activity does not predict very low-density lipoprotein-triglyceride fatty acid oxidation during exercise	Sondergaard, E.; Andersen, I. R.; Sorensen, L. P.; Gormsen, L. C.; Nielsen, S.	2017	Did not perform fiber type analysis
EFFECTS OF SHORT-TERM FREE-WEIGHT AND SEMIBLOCK PERIODIZATION RESISTANCE TRAINING ON METABOLIC SYNDROME	South, M. A.; Layne, A. S.; Stuart, C. A.; Triplett, N. T.; Ramsey, M.; Howell, M. E.; Sands, W. A.; Mizuguchi, S.; Hornsby, W. G.; Kavanaugh, A. A.; Stone, M. H.	2016	Does not include male and female data
Altered muscle oxidative phenotype impairs exercise tolerance but does not Improve after exercise training in multiple sclerosis	Spaas, J.; Goulding, R. P.; Keytsman, C.; Fonteyn, L.; van Horssen, J.; Jaspers, R. T.; Eijnde, B. O.; Wust, R. C. I.	2022	Does not include male and female data
Common Genetic Variation in the Human FNDC5 Locus, Encoding the Novel Muscle-Derived 'Browning' Factor Irisin, Determines Insulin Sensitivity	Staiger, H.; Bohm, A.; Scheler, M.; Berti, L.; Machann, J.; Schick, F.; Machicao, F.; Fritsche, A.; Stefan, N.; Weigert, C.; Krook, A.; Haring, H. U.; de Angelis, M. H.	2013	Did not perform fiber type analysis
Human skeletal muscle fiber types: Delineation, development, and distribution	Staron, R. S.	1997	Review (ex: systematic review/meta-analysis)
ASSESSMENT OF SKELETAL-MUSCLE DAMAGE IN SUCCESSIVE BIOPSIES FROM STRENGTH-TRAINED AND UNTRAINED MEN AND WOMEN	Staron, R. S.; Hikida, R. S.; Murray, T. F.; Nelson, M. M.; Johnson, P.; Hagerman, F.	1992	Did not perform fiber type analysis
MYOSIN POLYMORPHISM AND DIFFERENTIAL EXPRESSION IN ADULT HUMAN SKELETAL-MUSCLE	Staron, R. S.; Johnson, P.	1993	Review (ex: systematic review/meta-analysis)
Effect of short-term training on mitochondrial ATP production rate in human skeletal muscle	Starritt, E. C.; Angus, D.; Hargreaves, M.	1999	Did not perform fiber type analysis
Ribosome biogenesis may augment resistance training-induced myofiber hypertrophy and is required for myotube growth in vitro	Stec, M. J.; Kelly, N. A.; Many, G. M.; Windham, S. T.; Tuggle, S. C.; Bamman, M. M.	2016	Does not include male and female data
Plasma adiponectin concentration is associated with skeletal muscle insulin receptor tyrosine phosphorylation, and low plasma concentration precedes a decrease in whole-body insulin sensitivity in humans	Stefan, N.; Vozarova, B.; Funahashi, T.; Matsuzawa, Y.; Weyer, C.; Lindsay, R. S.; Youngren, J. F.; Havel, P. J.; Pratley, R. E.; Bogardus, C.; Tataranni, P. A.	2002	Did not perform fiber type analysis
Insulin-like growth factor-I biocompartmentalization across blood, interstitial fluid and muscle, before and after 3 months of chronic resistance exercise	Sterczala, A. J.; Pierce, J. R.; Barnes, B. R.; Urso, M. L.; Matheny, R. W.; Scofield, D. E.; Flanagan, S. D.; Maresh, C. M.; Zambraski, E. J.; Kraemer, W. J.; Nindl, B. C.	2022	Did not perform fiber type analysis
Electrophysiological and histological changes of paraspinal muscles in adolescent idiopathic scoliosis	Stetkarova, I.; Zamecnik, J.; Bocek, V.; Vasko, P.; Brabec, K.; Krbec, M.	2016	No healthy subjects or controls
Skeletal Muscle Fibre Characteristics of the Lumbar Multifidus Muscle in Patients Undergoing Microdiscectomy for Unilateral Lumbar Disc Herniation	Stevens, S.; Agten, A.; Snijders, T.; Plazier, M.; Bamps, S.; Assieker, T.; Betz, M. W.; Timmermans, A.; van Loon, L. J. C.; Vandenabeele, F.	2022	Does not include male and female data
Skeletal Muscle ACSL Isoforms Relate to Measures of Fat Metabolism in Humans	Stierwalt, H. D.; Ehrlicher, S. E.; Robinson, M. M.; Newsom, S. A.	2021	Did not perform fiber type analysis

The role of donor age and gender in the success of human muscle precursor cell transplantation	Stolting, M. N. L.; Hefermehl, L. J.; Tremp, M.; Azzabi, F.; Sulser, T.; Eberli, D.	2017	No cross-sectional area data
Genetic and nongenetic determinants of skeletal muscle glucose transporter 4 messenger ribonucleic acid levels and insulin action in twins	Storgaard, H.; Poulsen, P.; Ling, C.; Groop, L.; Vaag, A. A.	2006	Did not perform fiber type analysis
Age-related reduction in single muscle fiber calcium sensitivity is associated with decreased muscle power in men and women	Straight, C. R.; Ades, P. A.; Toth, M. J.; Miller, M. S.	2018	Does not include male and female data
Improvements in skeletal muscle fiber size with resistance training are age-dependent in older adults: a systematic review and meta-analysis	Straight, C. R.; Fedewa, M. V.; Toth, M. J.; Miller, M. S.	2020	Review (ex: systematic review/meta-analysis)
Quadriceps Lipid Content Has Sex-Specific Associations With Whole-Muscle, Cellular, and Molecular Contractile Function in Older Adults	Straight, C. R.; Voigt, T. B.; Jala, A. V.; Chase, J. D.; Ringham, O. R.; Ades, P. A.; Toth, M. J.; Miller, M. S.	2019	No cross-sectional area data
Neither Hematocrit Normalization nor Exercise Training Restores Oxygen Consumption to Normal Levels in Hemodialysis Patients	Stray-Gundersen, J.; Howden, E. J.; Parsons, D. B.; Thompson, J. R.	2016	Does not include male and female data
Effects of Sample Size on Differential Gene Expression, Rank Order and Prediction Accuracy of a Gene Signature	Stretch, C.; Khan, S.; Asgarian, N.; Eisner, R.; Vaisipour, S.; Damaraju, S.; Graham, K.; Bathe, O. F.; Steed, H.; Greiner, R.; Baracos, V. E.	2013	Did not perform fiber type analysis
Bone marrow derived cells in adult skeletal muscle tissue in humans	Stromberg, A.; Jansson, M.; Fischer, H.; Rullman, E.; Hagglund, H.; Gustafsson, T.	2013	Does not include males and females
PRE-TRAINING MUSCLE CHARACTERISTICS OF SUBJECTS WHO ARE OBESE DETERMINE HOW WELL EXERCISE TRAINING WILL IMPROVE THEIR INSULIN RESPONSIVENESS	Stuart, C. A.; Lee, M. L.; South, M. A.; Howell, M. E. A.; Cartwright, B. M.; Ramsey, M. W.; Stone, M. H.	2017	No healthy subjects or controls
Slow-Twitch Fiber Proportion in Skeletal Muscle Correlates With Insulin Responsiveness	Stuart, C. A.; McCurry, M. P.; Marino, A.; South, M. A.; Howell, M. E. A.; Layne, A. S.; Ramsey, M. W.; Stone, M. H.	2013	Does not include male and female data
Influence of diagnostic categories, age, and gender on antioxidative defense and lipid peroxidation in skeletal muscle of patients with neuromuscular diseases	Stuerenburg, H. J.; Stangneth, B.; Kohlschutter, A.; Finckh, B.	2003	Did not perform fiber type analysis
Gender differences in muscle inflammation after eccentric exercise	Stupka, N.; Lowther, S.; Chorneyko, K.; Bourgeois, J. M.; Hogben, C.; Tarnopolsky, M. A.	2000	Did not perform fiber type analysis
Cellular adaptation to repeated eccentric exercise-induced muscle damage	Stupka, N.; Tarnopolsky, M. A.; Yardley, N. J.; Phillips, S. M.	2001	Did not perform fiber type analysis
Resistance training induces qualitative changes in muscle morphology, muscle architecture, and muscle function in elderly postoperative patients	Suetta, C.; Andersen, J. L.; Dalgas, U.; Berget, J.; Koskinen, S.; Aagaard, P.; Magnusson, S. P.; Kjaer, M.	2008	No healthy subjects or controls
Coordinated increase in skeletal muscle fiber area and expression of IGF-I with resistance exercise in elderly post-operative patients	Suetta, C.; Clemmensen, C.; Andersen, J. L.; Magnusson, S. P.; Schjerling, P.; Kjaer, M.	2010	Does not include male and female data

Skeletal muscle IGF-1 is lower at rest and after resistance exercise in humans with obesity	Sullivan, B. P.; Weiss, J. A.; Nie, Y. H.; Garner, R. T.; Drohan, C. J.; Kuang, S. H.; Stout, J.; Gavin, T. P.	2020	Does not include male and female data
The anabolic response to resistance exercise and a protein-rich meal is not diminished by age	Symons, T. B.; Sheffield-Moore, M.; Mamerow, M. M.; Wolfe, R. R.; Paddon-Jones, D.	2011	Did not perform fiber type analysis
A Moderate Serving of High-Quality Protein Maximally Stimulates Skeletal Muscle Protein Synthesis in Young and Elderly Subjects	Symons, T. B.; Sheffield-Moore, M.; Wolfe, R. R.; Paddon-Jones, D.	2009	Did not perform fiber type analysis
Carnosine, taurine and enzyme activities of human skeletal muscle fibres from elderly subjects with osteoarthritis and young moderately active subjects	Tallon, M. J.; Harris, R. C.; Maffulli, N.; Tarnopolsky, M. A.	2007	No cross-sectional area data
Molecular mechanisms involved in TGF-beta 1-induced Muscle-derived stem cells differentiation to smooth muscle cells	Tang, X.; Su, X. H.; Zhong, Z. H.; Wen, C. L.; Zhang, T. S.; Zhu, Y. L.	2019	Did not perform fiber type analysis
Type-2 muscle fiber atrophy is associated with sarcopenia in elderly men with hip fracture	Tanganelli, F.; Meinke, P.; Hofmeister, F.; Jarmusch, S.; Baber, L.; Mehaffey, S.; Hintze, S.; Ferrari, U.; Neuerburg, C.; Kammerlander, C.; Schoser, B.; Drey, M.	2021	No healthy subjects or controls
Influence of endurance exercise training and sex on intramyocellular lipid and mitochondrial ultrastructure, substrate use, and mitochondrial enzyme activity	Tarnopolsky, M. A.; Rennie, C. D.; Robertshaw, H. A.; Fedak-Tarnopolsky, S. N.; Devries, M. C.; Hamadeh, M. J.	2007	Does not include male and female data
Gender differences in carbohydrate loading are related to energy intake	Tarnopolsky, M. A.; Zawada, C.; Richmond, L. B.; Carter, S.; Shearer, J.; Graham, T.; Phillips, S. M.	2001	Did not perform fiber type analysis
Beneficial effects of dark chocolate on exercise capacity in sedentary subjects: underlying mechanisms. A double blind, randomized, placebo controlled trial	Taub, P. R.; Ramirez-Sanchez, I.; Patel, M.; Higginbotham, E.; Moreno-Ulloa, A.; Roman-Pintos, L. M.; Phillips, P.; Perkins, G.; Ceballos, G.; Villarreal, F.	2016	Did not perform fiber type analysis
The effects of endurance training on muscle fibre types and enzyme activities	Taylor, A. W.; Bachman, L.	1999	Review (ex: systematic review/meta-analysis)
Inadequate protein intake affects skeletal muscle transcript profiles in older humans	Thalacker-Mercer, A. E.; Fleet, J. C.; Craig, B. A.; Carnell, N. S.; Campbell, W. W.	2007	Did not perform fiber type analysis
Short-term aerobic conditioning prior to resistance training augments muscle hypertrophy and satellite cell content in healthy young men and women	Thomas, A. C. Q.; Brown, A.; Hatt, A. A.; Manta, K.; Costa-Parke, A.; Kamal, M.; Joanisse, S.; McGlory, C.; Phillips, S. M.; Kumbhare, D.; Parise, G.	2022	Does not include male and female data
A single bout of eccentric exercise increases HSP27 and HSC/HSP70 in human skeletal muscle	Thompson, H. S.; Scordilis, S. P.; Clarkson, P. M.; Lohrer, W. A.	2001	Did not perform fiber type analysis
EFFECTS OF AGE AND TRAINING ON SKELETAL-MUSCLE PHYSIOLOGY AND PERFORMANCE	Thompson, L. V.	1994	Review (ex: systematic review/meta-analysis)
Satellite cells and training in the elderly	Thornell, L. E.; Lindstrom, M.; Renault, V.; Mouly, V.; Butler-Browne, G. S.	2003	Review (ex: systematic

			review/meta-analysis)
Chronic congestive heart failure elicits adaptations of endurance exercise in diaphragmatic muscle	Tikunov, B.; Levine, S.; Mancini, D.	1997	Autopsies
Expression profiling following local muscle inactivity in humans provides new perspective on diabetes-related genes	Timmons, J. A.; Norrbom, J.; Scheele, C.; Thonberg, H.; Wahlestedt, C.; Tesch, P.	2006	Did not perform fiber type analysis
Indices of extracellular matrix turnover in human masseter muscles as markers of craniofacial form - a preliminary study	Tippett, H. L.; Dodgson, L. K.; Hunt, N. P.; Lewis, M. P.	2008	Did not perform fiber type analysis
Sex and fiber type independently influence AMPK, TBC1D1, and TBC1D4 at rest and during recovery from high-intensity exercise in humans	Tobias, I. S.; Lazauskas, K. K.; Siu, J.; Costa, P. B.; Coburn, J. W.; Galpin, A. J.	2020	No cross-sectional area data
Insulin kinetics, insulin action, and muscle morphology in lean or slightly overweight persons with impaired glucose tolerance	Toft, I.; Bonaa, K. H.; Lindal, S.; Jenssen, T.	1998	Does not include male and female data
Impact of prolonged overfeeding on skeletal muscle mitochondria in healthy individuals	Toledo, F. G. S.; Johannsen, D. L.; Covington, J. D.; Bajpeyi, S.; Goodpaster, B.; Conley, K. E.; Ravussin, E.	2018	Did not perform fiber type analysis
Mitochondrial capacity in skeletal muscle is not stimulated by weight loss despite increases in insulin action and decreases in intramyocellular lipid content	Toledo, F. G. S.; Menshikova, E. V.; Azuma, K.; Radikovi, Z.; Kelley, C. A.; Ritov, V. B.; Kelley, D. E.	2008	Does not include male and female data
Changes induced by physical activity and weight loss in the morphology of intermyofibrillar mitochondria in obese men and women	Toledo, F. G. S.; Watkins, S.; Kelley, D. E.	2006	Did not perform fiber type analysis
Myosin heavy chain isoforms in human laryngeal muscles: An expression study based on gel electrophoresis	Toniolo, L.; Macchi, V.; Porzionato, A.; Paoli, A.; Marchese-Ragona, R.; De Caro, R.; Reggiani, C.	2008	No cross-sectional area data
Age-related differences in skeletal muscle protein synthesis: relation to markers of immune activation	Toth, M. J.; Matthews, D. E.; Tracy, R. P.; Previs, M. J.	2005	Does not include male and female data
Utility of Neuromuscular Electrical Stimulation to Preserve Quadriceps Muscle Fiber Size and Contractility After Anterior Cruciate Ligament Injuries and Reconstruction: A Randomized, Sham-Controlled, Blinded Trial	Toth, M. J.; Tourville, T. W.; Voigt, T. B.; Choquette, R. H.; Anair, B. M.; Falcone, M. J.; Failla, M. J.; Stevens-Lapslaey, J. E.; Endres, N. K.; Slauterbeck, J. R.; Beynnon, B. D.	2020	No healthy subjects or controls
Diathermy treatment increases heat shock protein expression in female, but not male skeletal muscle	Touchberry, C.; Le, T.; Richmond, S.; Prewitt, M.; Beck, D.; Carr, D.; Vardiman, P.; Gallagher, P.	2008	Did not perform fiber type analysis
Atypical Skeletal Muscle Profiles in Human Immunodeficiency Virus-Infected Asymptomatic Middle-Aged Adults	Tran, T.; Guardigni, V.; Pencina, K. M.; Amato, A. A.; Floyd, M.; Brawley, B.; Mozeleski, B.; McKinnon, J.; Woodbury, E.; Heckel, E.; Li, Z. Y.; Storer, T.; Sax, P. E.; Montano, M.	2018	Does not include male and female data
Reduced Levels of NAD in Skeletal Muscle and Increased Physiologic Frailty Are Associated With Viral Coinfection in Asymptomatic Middle-Aged Adults	Tran, T.; Pencina, K. M.; Schultz, M. B.; Li, Z. Y.; Ghattas, C.; Lau, J.; Sinclair, D. A.; Montano, M.	2022	Did not perform fiber type analysis

Human soleus single muscle fiber function with exercise or nutrition countermeasures during 60 days of bed rest	Trappe, S.; Creer, A.; Minchev, K.; Slivka, D.; Louis, E.; Luden, N.; Trappe, T.	2008	Does not include males and females
Single muscle fibre contractile properties in young and old men and women	Trappe, S.; Gallagher, P.; Harber, M.; Carrithers, J.; Fluckey, J.; Trappe, T.	2003	Single Fiber Analysis
Resistance training improves single muscle fiber contractile function in older women	Trappe, S.; Godard, M.; Gallagher, P.; Carroll, C.; Rowden, G.; Porter, D.	2001	Does not include males and females
Single muscle fiber adaptations with marathon training	Trappe, S.; Harber, M.; Creer, A.; Gallagher, P.; Slivka, D.; Minchev, K.; Whitsett, D.	2006	Does not include male and female data
Calf muscle strength in humans	Trappe, S. W.; Trappe, T. A.; Lee, G. A.; Costill, D. L.	2001	Does not include male and female data
Influence of aging and long-term unloading on the structure and function of human skeletal muscle	Trappe, T.	2009	Review (ex: systematic review/meta-analysis)
Influence of acetaminophen and ibuprofen on skeletal muscle adaptations to resistance exercise in older adults	Trappe, T. A.; Carroll, C. C.; Dickinson, J. M.; LeMoine, J. K.; Haus, J. M.; Sullivan, B. E.; Lee, J. D.; Jemiolo, B.; Weinheimer, E. M.; Hollon, C. J.	2011	Does not include male and female data
Time course and fibre type-dependent nature of calcium-handling protein responses to sprint interval exercise in human skeletal muscle	Tripp, T. R.; Frankish, B. P.; Lun, V.; Wiley, J. P.; Shearer, J.; Murphy, R. M.; MacInnis, M. J.	2022	Does not include male and female data
Exercise training increases lipid metabolism gene expression in human skeletal muscle	Tunstall, R. J.; Mehan, K. A.; Wadley, G. D.; Collier, G. R.; Bonen, A.; Hargreaves, M.; Cameron-Smith, D.	2002	Did not perform fiber type analysis
Comparison of bolus injection and constant infusion methods for measuring muscle protein fractional synthesis rate in humans	Tuvdendorj, D.; Chinkes, D. L.; Bahadorani, J.; Zhang, X. J.; Sheffield-Moore, M.; Killewich, L. A.; Wolfe, R. R.	2014	Did not perform fiber type analysis
Na+-K+-ATPase alpha 2-gene and skeletal muscle characteristics in response to long-term overfeeding	Ukkola, O.; Joanisse, D. R.; Tremblay, A.; Bouchard, C.	2003	Does not include males and females
Changes in ubiquitin proteasome pathway gene expression in skeletal muscle with exercise and statins	Urso, M. L.; Clarkson, P. M.; Hittel, D.; Hoffman, E. P.; Thompson, P. D.	2005	Does not include males and females
Exercise training effects on skeletal muscle plasticity and IGF-1 receptors in frail elders	Urso, M. L.; Singh, M. A. F.; Ding, W. J.; Evans, W. J.; Cosmas, A. C.; Manfredi, T. G.	2005	Did not perform fiber type analysis
The effect of testosterone on gastrocnemius muscle fibres in growing and adult male and female rats: A histochemical, morphometric and ultrastructural study	Ustunel, I.; Akkoyunlu, G.; Demir, R.	2003	Animal study
GLUCOSE FATTY-ACID CYCLE OPERATES IN HUMANS AT THE LEVELS OF BOTH WHOLE-BODY AND SKELETAL-MUSCLE DURING LOW AND HIGH PHYSIOLOGICAL PLASMA-INSULIN CONCENTRATIONS	Vaag, A. A.; Handberg, A.; Skott, P.; Richter, E. A.; Becknielsen, H.	1994	Did not perform fiber type analysis
Muscle Quality is More Impaired in Sarcopenic Patients With Chronic Obstructive Pulmonary Disease	van de Bool, C.; Gosker, H. R.; van den Borst, B.; Op den Kamp, C. M.; Slot, I. G. M.; Schols, Amwj	2016	Does not include male and female data

Altered skeletal muscle fatty acid handling is associated with the degree of insulin resistance in overweight and obese humans	van der Kolk, B. W.; Goossens, G. H.; Jocken, J. W.; Blaak, E. E.	2016	Did not perform fiber type analysis
DNA methylation alterations in muscle of critically ill patients	Van Dyck, L.; Guiza, F.; Derese, I.; Pauwels, L.; Casaer, M. P.; Hermans, G.; Wouters, P. J.; Van den Berghe, G.; Vanhorebeek, I.	2022	Did not perform fiber type analysis
Maximizing postexercise muscle glycogen synthesis: carbohydrate supplementation and the application of amino acid or protein hydrolysate mixtures	van Loon, L. J. C.; Saris, W. H. M.; Kruijshoop, M.; Wagenmakers, A. J. M.	2000	Does not include males and females
The mechanistic bases of the power-time relationship: muscle metabolic responses and relationships to muscle fibre type	Vanhatalo, A.; Black, M. I.; DiMenna, F. J.; Blackwell, J. R.; Schmidt, J. F.; Thompson, C.; Wylie, L. J.; Mohr, M.; Bangsbo, J.; Krustrup, P.; Jones, A. M.	2016	Does not include male and female data
Satellite cells in human skeletal muscle; from birth to old age	Verdijk, L. B.; Snijders, T.; Drost, M.; Delhaas, T.; Kadi, F.; van Loon, L. J. C.	2014	Does not include males and females
PRETRANSLATIONAL AND POSTTRANSLATIONAL UP-REGULATION OF MUSCLE-SPECIFIC GLYCOGEN-SYNTHASE IN ATHLETES	Vestergaard, H.; Andersen, P. H.; Lund, S.; Schmitz, O.; Junker, S.; Pedersen, O.	1994	Does not include males and females
GH signaling in skeletal muscle and adipose tissue in healthy human subjects: impact of gender and age	Vestergaard, P. F.; Vendelbo, M. H.; Pedersen, S. B.; Juul, A.; Ringgard, S.; Moller, N.; Jessen, N.; Jorgensen, J. O. L.	2014	Did not perform fiber type analysis
Effects of high-intensity interval training with hyperbaric oxygen	Villela, M. A.; Dunworth, S. A.; Kraft, B. D.; Harlan, N. P.; Natoli, M. J.; Suliman, H. B.; Moon, R. E.	2022	Did not perform fiber type analysis
Alpha-actinin-3 deficiency does not significantly alter oxidative enzyme activity in fast human muscle fibres	Vincent, B.; Windelinckx, A.; Van Proeyen, K.; Masschelein, E.; Nielens, H.; Ramaekers, M.; Van Leemputte, M.; Hespel, P.; Thomis, M.	2012	Does not include males and females
Effect of resistance exercise on muscle steroidogenesis	Vingren, J. L.; Kraemer, W. J.; Hatfield, D. L.; Anderson, J. M.; Volek, J. S.; Ratamess, N. A.; Thomas, G. A.; Ho, J. Y.; Fragala, M. S.; Maresh, C. M.	2008	Did not perform fiber type analysis
Effect of sex differences on human MEF2 regulation during endurance exercise	Vissing, K.; McGee, S. L.; Roepstorff, C.; Schjerling, P.; Hargreaves, M.; Kiens, B.	2008	Did not perform fiber type analysis
Exogenous amino acids stimulate net muscle protein synthesis in the elderly	Volpi, E.; Ferrando, A. A.; Yeckel, C. W.; Tipton, K. D.; Wolfe, R. R.	1998	Does not include males and females
Essential amino acids are primarily responsible for the amino acid stimulation of muscle protein anabolism in healthy elderly adults	Volpi, E.; Kobayashi, H.; Sheffield-Moore, M.; Mittendorfer, B.; Wolfe, R. R.	2003	Did not perform fiber type analysis
The response of muscle protein anabolism to combined hyperaminoacidemia and glucose-induced hyperinsulinemia is impaired in the elderly	Volpi, E.; Mittendorfer, B.; Rasmussen, B. B.; Wolfe, R. R.	2000	Did not perform fiber type analysis
Oral amino acids stimulate muscle protein anabolism in the elderly despite higher first-pass splanchnic extraction	Volpi, E.; Mittendorfer, B.; Wolf, S. E.; Wolfe, R. R.	1999	Did not perform fiber type analysis

EFFECT OF ACUTE TRANSCRANIAL MAGNETIC STIMULATION ON INTRACELLULAR SIGNALLING IN HUMAN SKELETAL MUSCLE	von Walden, F.; Gidlund, E. K.; Liu, C.; Ramstrand, N.; Norrbom, J.; von Wachenfelt, N.; Kjellgren, H.; Sundberg, C. J.; Ponten, E.; Alkner, B.	2020	Did not perform fiber type analysis
Histologic Evaluation of Nonvisual Afferent Sensory Upper Eyelid Proprioception	Vrcek, I.; Blumer, R.; Blandford, A.; Somogyi, M.; Durairaj, V.; Blaydon, S.; Shore, J.; Amato, M.; Nakra, T.	2020	Did not perform fiber type analysis
Changes in insulin action and GLUT-4 with 6 days of inactivity in endurance runners	Vukovich, M. D.; Arciero, P. J.; Kohrt, W. M.; Racette, S. B.; Hansen, P. A.; Holloszy, J. O.	1996	Did not perform fiber type analysis
Differential effects of exercise on insulin-signaling gene expression in human skeletal muscle	Wadley, G. D.; Tunstall, R. J.; Sanigorski, A.; Collier, G. R.; Hargreaves, M.; Cameron-Smith, D.	2001	Did not perform fiber type analysis
Metformin blunts muscle hypertrophy in response to progressive resistance exercise training in older adults: A randomized, double-blind, placebo-controlled, multicenter trial: The MASTERS trial	Walton, R. G.; Dungan, C. M.; Long, D. E.; Tuggle, S. C.; Kosmac, K.; Peck, B. D.; Bush, H. M.; Tezanos, A. G. V.; McGwin, G.; Windham, S. T.; Ovalle, F.; Bamman, M. M.; Kern, P. A.; Peterson, C. A.	2019	Does not include male and female data
Impact of maximal strength training on work efficiency and muscle fiber type in the elderly: Implications for physical function and fall prevention	Wang, E.; Nyberg, S. K.; Hoff, J.; Zhao, J.; Leivseth, G.; Torhaug, T.; Husby, O. S.; Helgerud, J.; Richardson, R. S.	2017	Does not include males and females
Skeletal muscle mRNA for IGF-IEa, IGF-II, and IGF-I receptor is decreased in sedentary chronic hemodialysis patients	Wang, H.; Casaburi, R.; Taylor, W. E.; Aboellail, H.; Storer, T. W.; Kopple, J. D.	2005	Did not perform fiber type analysis
Subtyping obesity with microarrays: implications for the diagnosis and treatment of obesity	Wang, S.; Sparks, L. M.; Xie, H.; Greenway, F. L.; de Jonge, L.; Smith, S. R.	2009	Did not perform fiber type analysis
Relationship of Physical Function to Single Muscle Fiber Contractility in Older Adults: Effects of Resistance Training With and Without Caloric Restriction	Wang, Z. M.; Leng, X. Y.; Messi, M. L.; Choi, S. J.; Marsh, A. P.; Nicklas, B.; Delbono, O.	2019	Does not include male and female data
Increased Smad Signaling and Reduced MRF Expression in Skeletal Muscle from Obese Subjects	Watts, R.; McAinch, A. J.; Dixon, J. B.; O'Brien, P. E.; Cameron-Smith, D.	2013	Does not report sex of subjects
Concentric resistance training increases muscle strength without affecting microcirculation	Weber, M. A.; Hildebrandt, W.; Schroder, L.; Kinscherf, R.; Krix, M.; Bachert, P.; Delorme, S.; Essig, M.; Kauczor, H. U.; Krakowski-Roosen, H.	2010	Did not perform fiber type analysis
Pathologic skeletal muscle perfusion in patients with myositis: Detection with quantitative contrast-enhanced US - Initial results	Weber, M. A.; Krix, M.; Jappe, U.; Huttner, H. B.; Hartmann, M.; Meyding-Lamade, U.; Essig, M.; Fiehn, C.; Kauczor, H. U.; Delorme, S.	2006	Did not perform fiber type analysis
Ingestion of Free Amino Acids Compared with an Equivalent Amount of Intact Protein Results in More Rapid Amino Acid Absorption and Greater Postprandial Plasma Amino Acid Availability Without Affecting Muscle Protein Synthesis Rates in Young Adults in a Double-Blind Randomized Trial	Weijzen, M. E. G.; van Gassel, R. J. J.; Kouw, I. W. K.; Trommelen, J.; Gorissen, S. H. M.; van Kranenburg, J.; Goessens, J. P. B.; van de Poll, M. C. G.; Verdijk, L. B.; van Loon, L. J. C.	2022	Did not perform fiber type analysis

Polyadenylated RNA, actin mRNA, and myosin heavy chain mRNA in young and old human skeletal muscle	Welle, S.; Bhatt, K.; Thornton, C.	1996	No cross-sectional area data
Stimulation of myofibrillar synthesis by exercise is mediated by more efficient translation of mRNA	Welle, S.; Bhatt, K.; Thornton, C. A.	1999	Does not include male and female data
Insulin-like growth factor-I, actin, and myosin heavy chain messenger RNAs in skeletal muscle after an injection of growth hormone in subjects over 60 years old	Welle, S.; Thornton, C.	1997	Did not perform fiber type analysis
MYOFIBRILLAR PROTEIN-SYNTHESIS IN YOUNG AND OLD HUMAN-SUBJECTS AFTER 3 MONTHS OF RESISTANCE TRAINING	Welle, S.; Thornton, C.; Statt, M.	1995	Did not perform fiber type analysis
High Intensity Aerobic and Resistance Exercise Can Improve Glucose Tolerance in Persons With Multiple Sclerosis A Randomized Controlled Trial	Wens, I.; Dalgas, U.; Vandenabeele, F.; Verboven, K.; Hansen, D.; Deckx, N.; Cools, N.; Eijnde, B. O.	2017	No healthy subjects or controls
Acute low-load resistance exercise with and without blood flow restriction increased protein signalling and number of satellite cells in human skeletal muscle	Wernbom, M.; Apro, W.; Paulsen, G.; Nilsen, T. S.; Blomstrand, E.; Raastad, T.	2013	Did not perform fiber type analysis
Sex-based comparisons of myofibrillar protein synthesis after resistance exercise in the fed state	West, D. W. D.; Burd, N. A.; Churchward-Venne, T. A.; Camera, D. M.; Mitchell, C. J.; Baker, S. K.; Hawley, J. A.; Coffey, V. G.; Phillips, S. M.	2012	Did not perform fiber type analysis
A 7-day high-fat, high-calorie diet induces fibre-specific increases in intramuscular triglyceride and perilipin protein expression in human skeletal muscle	Whytock, K. L.; Parry, S. A.; Turner, M. C.; Woods, R. M.; James, L. J.; Ferguson, R. A.; Stahlman, M.; Boren, J.; Strauss, J. A.; Cocks, M.; Wagenmakers, A. J. M.; Hulston, C. J.; Shepherd, S. O.	2020	Does not include male and female data
Metabolic and functional changes in transgender individuals following cross-sex hormone treatment: Design and methods of the GEnder Dysphoria Treatment in Sweden (GETS) study	Wiik, A.; Andersson, D. P.; Brismar, T. B.; Chanpen, S.; Dhejne, C.; Ekstrom, T. J.; Flanagan, J. N.; Holmberg, M.; Kere, J.; Lilja, M.; Lindholm, M. E.; Lundberg, T. R.; Maret, E.; Melin, M.; Olsson, S. M.; Rullman, E.; Wahlen, K.; Arver, S.; Gustafsson, T.	2018	Not peer reviewed (ex: thesis, conference poster).
Expression of both oestrogen receptor alpha and beta in human skeletal muscle tissue	Wiik, A.; Ekman, M.; Johansson, O.; Jansson, E.; Esbjornsson, M.	2009	Did not perform fiber type analysis
Oestrogen receptor beta is expressed in adult human skeletal muscle both at the mRNA and protein level	Wiik, A.; Glenmark, B.; Ekman, M.; Esbjornsson-Liljedahl, M.; Johansson, O.; Bodin, K.; Enmark, E.; Jansson, E.	2003	Did not perform fiber type analysis
Effects of prolonged fasting on AMPK signaling, gene expression, and mitochondrial respiratory chain content in skeletal muscle from lean and obese individuals	Wijngaarden, M. A.; van der Zon, G. C.; van Dijk, K. W.; Pijl, H.; Guigas, B.	2013	Did not perform fiber type analysis
Metabolic capacity of the diaphragm in patients with COPD	Wijnhoven, J. H.; Janssen, A. J. M.; van Kuppevelt, T. H.; Rodenburg, R. J. T.; Dekhuijzen, P. N. R.	2006	Does not include males and females
Reduced exercise tolerance in CHF may be related to factors other than impaired skeletal muscle oxidative capacity	Williams, A. D.; Selig, S.; Hare, D. L.; Hayes, A.; Krum, H.; Patterson, J.; Geerling, R. H.; Toia, D.; Carey, M. F.	2004	Does not include male and female data

Estradiol in females may negate skeletal muscle myostatin mRNA expression and serum myostatin propeptide levels after eccentric muscle contractions	Willoughby, D. S.; Wilborn, C. D.	2006	Did not perform fiber type analysis
THE EFFECTS OF ENDURANCE, STRENGTH, AND POWER TRAINING ON MUSCLE FIBER TYPE SHIFTING	Wilson, J. M.; Loenneke, J. P.; Jo, E.; Wilson, G. J.; Zourdos, M. C.; Kim, J. S.	2012	Review (ex: systematic review/meta-analysis)
The characterization of decellularized human skeletal muscle as a blueprint for mimetic scaffolds	Wilson, K.; Terlouw, A.; Roberts, K.; Wolchok, J. C.	2016	Autopsies
CAPILLARIES WITHIN HUMAN SKELETAL-MUSCLE FIBERS	Wolf, R.; Goebel, H. H.; Gutmann, L.; Schochet, S.	1991	No healthy subjects or controls
NO EFFECT OF CARBOHYDRATE FEEDING ON GLYCOGEN-SYNTHASE IN HUMAN MUSCLE DURING EXERCISE	Yan, Z.; Spencer, M. K.; Katz, A.	1993	Did not perform fiber type analysis
SUBUNIT COMPONENTS IN SALT-SOLUBLE AND INSOLUBLE FRACTIONS OF CARP MYOFIBRILS DURING FROZEN STORAGE	Yoshikawa, K.; Inoue, N.; Kawai, Y.; Shinano, H.	1995	Animal study
Potential effects of long-term abuse of anabolic androgen steroids on human skeletal muscle	Yu, J. G.; Isaksson, A.; Rova, A.; Tegner, Y.; Eriksson, A.; Malm, C.	2020	Does not report sex of subjects
Metabolic and mitogenic signal transduction in human skeletal muscle after intense cycling exercise	Yu, M.; Stepto, N. K.; Chibalin, A. V.; Fryer, L. G. D.; Carling, D.; Krook, A.; Hawley, J. A.; Zierath, J. R.	2003	Does not report sex of subjects
Do blood cells mimic gene expression profile alterations known to occur in muscular adaptation to endurance training?	Zeibig, J.; Karlic, H.; Lohninger, A.; Dumsgaard, R.; Smekal, G.	2005	Does not include males and females
The I allele of the angiotensin-converting enzyme gene is associated with an increased percentage of slow-twitch type I fibers in human skeletal muscle	Zhang, B.; Tanaka, H.; Shono, N.; Miura, S.; Kiyonaga, A.; Shindo, M.; Saku, K.	2003	Does not include male and female data
DOC2B promotes insulin sensitivity in mice via a novel KLC1-dependent mechanism in skeletal muscle	Zhang, J.; Oh, E.; Merz, K. E.; Aslamy, A.; Veluthakal, R.; Salunkhe, V. A.; Ahn, M.; Tunduguru, R.; Thurmond, D. C.	2019	Did not perform fiber type analysis
Effects of an amylopectin and chromium complex on the anabolic response to a suboptimal dose of whey protein	Ziegenfuss, T. N.; Lopez, H. L.; Kedia, A.; Habowski, S. M.; Sandrock, J. E.; Raub, B.; Kerksick, C. M.; Ferrando, A. A.	2017	Did not perform fiber type analysis
Human skeletal muscle mitochondria in aging: Lack of detectable morphological and enzymic defects	Zucchini, C.; Pugnaloni, A.; Pallotti, F.; Solmi, R.; Crimi, M.; Castaldini, C.; Biagini, G.; Lenaz, G.	1995	Did not perform fiber type analysis
The ELSA trial: single versus combinatory effects of non-prohibited beta-2 agonists on skeletal muscle metabolism, cardiopulmonary function and endurance performance-study protocol for a randomized 4-way balanced cross-over trial	Zugel, M.; Bizjak, D. A.; Nussbaumer, D.; Winkert, K.; Takabayashi, K.; Kirsten, J.; Washington, M.; Treff, G.; Dreyhaupt, J.; Steeb, L.; Diel, P.; Parr, M. K.; Steinacker, J. M.; Persch, H.	2021	Not peer reviewed (ex: thesis, conference poster)
An image analysis study of vastus lateralis muscle fibers in malignant hyperthermia susceptible patients	Zukowski, F.; De Craemer, D.; Van den Branden, C.; De Cauwer, H.; Heytens, L.; Martin, J. J.	1998	No healthy subjects or controls
Effect of short-term exercise training on intramyocellular lipid content	Bajpeyi, S.; Reed, MA.; Molskness, S.; Newton, C.; Tanner, CJ.; McCartney, JS.; Houmard, JA.	2012	Does not include male and female data

Apoptosis in skeletal myocytes of patients with chronic heart failure is associated with exercise intolerance	Adams, Volker; Jiang, Hong; Yu, Jiangtao; MÃ¶bius-Winkler, Sven; Fiehn, Eduard; Linke, Axel; Weigl, Claudia; Schuler, Gerhard; Hambrecht, Rainer; Adams, V.; Jiang, H.; Yu, J.; MÃ¶bius-Winkler, S.; Fiehn, E.; Linke, A.; Weigl, C.; Schuler, G.; Hambrecht, R.	1999	Does not report sex of subjects
Anti-hydroxy-3-methylglutaryl-coenzyme A reductase (anti-HMGCR) antibody in necrotizing myopathy: treatment outcomes, cancer risk, and role of autoantibody level	Aggarwal, R.; Moghadam-Kia, S.; Lacomis, D.; Malik, A.; Qi, Z.; Koontz, D.; Burlingame, R. W.; Oddis, C. V.; Burlingame, R. W.; Oddis, C. V.	2020	Did not perform fiber type analysis
Biopsy samples from the erector spinae of persons with nonspecific chronic low back pain display a decrease in glycolytic muscle fibers	Agten, Anouk; Stevens, Sjoerd; Verbrugghe, Jonas; Timmermans, Annick; Vandenabeele, Frank	2020	Does not include male and female data
Human muscle fiber type-specific insulin signaling: impact of obesity and type 2 diabetes	Albers, Peter H.; Pedersen, Andreas J. T.; Birk, Jesper B.; Kristensen, Dorte E.; Vind, Birgitte F.; Baba, Otto; NÃ¸hr, Jane; HÃ¸jlund, Kurt; Wojtaszewski, JÃ¸rgen F. P.	2015	Does not include male and female data
Influence of Post-Exercise Carbohydrate-Protein Ingestion on Muscle Glycogen Metabolism in Recovery and Subsequent Running Exercise	Alghannam, Abdullah F.; Jedrzejewski, Dawid; Bilzon, James; Thompson, Dylan; Tsintzas, Kostas; Betts, James A.	2016	Did not perform fiber type analysis
Moderate Intensity Exercise Training Improves Skeletal Muscle Performance inÂ Symptomatic and Asymptomatic StatinÂ Users	Allard, Neeltje A. E.; Janssen, Lando; Aussieker, Thorben; Stoffels, Anouk A. F.; Rodenburg, Richard J.; Assendelft, Willem J. J.; Thompson, Paul D.; Snijders, Tim; Hopman, Maria T. E.; Timmers, Silvie	2021	Does not include male and female data
Statins Affect Skeletal Muscle Performance: Evidence for Disturbances in Energy Metabolism	Allard, Neeltje A. E.; Schirris, Tom J. J.; Verheggen, Rebecca J.; Russel, Frans G. M.; Rodenburg, Richard J.; Smeitink, Jan A. M.; Thompson, Paul D.; Hopman, Maria T. E.; Timmers, Silvie	2018	Did not perform fiber type analysis
Skeletal muscle triglycerides, diacylglycerols, and ceramides in insulin resistance: another paradox in endurance-trained athletes?	Amati, F.; DubÃ©, J. J.; Alvarez-Carnero, E.; Edreira, M. M.; Chomentowski, P.; Coen, P. M.; Switzer, G. E.; Bickel, P. E.; Stefanovic-Racic, M.; Toledo, F. G.; Goodpaster, B. H.; Amati, Francesca; DubÃ©, John J.; Alvarez-Carnero, Elvis; Edreira, Martin M.; Chomentowski, Peter; Coen, Paul M.; Switzer, Galen E.; Bickel, Perry E.; Stefanovic-Racic, Maja	2011	Does not include male and female data
Deregulation of microRNAs in blood and skeletal muscles of myotonic dystrophy type 1 patients	Ambrose, Kathlin K.; Ishak, Taufik; Lay-Hoong, Lian; Khean-Jin, Goh; Kum-Thong, Wong; Ahmad-Annuar, Azlina; Meow-Keong, Thong; Lian, Lay-Hoong; Goh, Khean-Jin; Wong, Kum-Thong; Thong, Meow-Keong	2017	Did not perform fiber type analysis
Effect of summer intermission on skeletal muscle of adolescent soccer players	Amigo, N.; Cadefau, J. A.; Ferrer, I.; Tarrados, N.; Cusso, R.	1998	Only in children (0-17 years)
Expression of neurotrophic factors in diabetic muscle--relation to neuropathy and muscle strength	Andreassen, C. S.; Jakobsen, J.; Flyvbjerg, A.; Andersen, H.	2009	Did not perform fiber type analysis

Resistance training variable manipulations are less relevant than intrinsic biology in affecting muscle fiber hypertrophy	Angleri, Vitor; Damas, Felipe; Phillips, Stuart M.; Selistreâ€deâ€Araujo, Heloisa Sobreiro; Cornachione, Anabelle Silva; Stotzer, UlianaÂ Sbeguen; Santanielo, Natalia; Soligon, Samuel Domingos; Costa, LuizÂ Augusto Riani; LixandrÃ£o, Manoel EmÃlio; ConceiÃ§Ã£o, Miguel Soares; Vechin, Felipe Cassaro; Ugrinowitsch, Carlos; Libardi, Cleiton Augusto	2022	Does not include males and females
Sporadic inclusion body myositis: morphology, regeneration, and cytoskeletal structure of muscle fibres	Arnardottir, S.; Borg, K.; Ansved, T.	2004	Does not include male and female data
Small vessel abnormalities in alternating hemiplegia of childhood: pathophysiologic implications	Auvin, S.; Joriot-Chekaf, S.; Cuvellier, J. C.; Pandit, F.; Cuisset, J. M.; Ruchoux, M. M.; VallÃ©e, L.	2006	Only in children (0-17 years)
Direct effects of TNF-Î± on local fuel metabolism and cytokine levels in the placebo-controlled, bilaterally infused human leg: increased insulin sensitivity, increased net protein breakdown, and increased IL-6 release	Bach, Ermina; Nielsen, Roni R.; Vendelbo, Mikkel H.; MÃ¸ller, Andreas B.; Jessen, Niels; Buhl, Mads; K-HafstrÃ¸m, Thomas; Holm, Lars; Pedersen, Steen B.; Pilegaard, Henriette; BiensÃ¸, Rasmus S.; JÃ¸rgensen, Jens O. L.; MÃ¸ller, Niels	2013	Does not include males and females
Change in the contractile behavior of muscle fibers in subjects with primary muscle dysfunction	Back, Claudio GregÃ³rio Nuernberg; Benedini-Elias, Priscila C. O.; Mattiello, Stela M.; Sobreira, Claudia; Martinez, Edson Z.; Mattiello-Sverzut, Ana Claudia	2013	No healthy subjects or controls
Lipid in skeletal muscle myotubes is associated to the donors' insulin sensitivity and physical activity phenotypes	Bajpeyi, Sudip; Myrland, Cassandra K.; Covington, Jeffrey D.; Obanda, Diana; Cefalu, William T.; Smith, Steven R.; Rustan, Arild C.; Ravussin, Eric	2014	Does not include males and females
ANT1 is reduced in sporadic inclusion body myositis	Barca, E.; Aguennouz, M.; Mazzeo, A.; Messina, S.; Toscano, A.; Vita, G. L.; Portaro, S.; Parisi, D.; Rodolico, C.; Barca, E.; Aguennouz, M.; Mazzeo, A.; Messina, S.; Toscano, A.; Vita, G. L.; Portaro, S.; Parisi, D.; Rodolico, C.	2013	Did not perform fiber type analysis
Oxidative stress and respiratory muscle dysfunction in severe chronic obstructive pulmonary disease	Barreiro, E.; de la Puente, B.; Minguella, J.; Corominas, J. M.; Serrano, S.; Hussain, S. N. A.; Gea, J.	2005	Does not include males and females
The effect of acute (60 minute) insulin stimulation upon human skeletal muscle glycogen synthase and protein phosphatase-1 in non-insulin-dependent diabetic patients and control subjects	Barriocanal, L. A.; Borthwick, A. C.; Stewart, M.; Wells, A.; Hurel, S. J.; Yeaman, S. J.; Taylor, R.	1995	Did not perform fiber type analysis
Histological abnormalities in muscle from patients with certain types of fibrositis	Bartels, E. M.; Danneskiold-SamsÃ¸e, B.	1986	Did not perform fiber type analysis
A well-tolerated core needle muscle biopsy process suitable for children and adults	Barthelemy, Florian; Woods, Jeremy D.; Nievesâ€Rodriguez, Shirley; Douine, Emilie D.; Wang, Richard; Wanagat, Jonathan; Miceli, M. Carrie; Nelson, Stanley F.; Nieves-Rodriguez, Shirley	2020	Did not perform fiber type analysis

Sex differences in volume overload in skinned fibers	Bening, C.; Hamouda, K.; Leyh, R.	2016	No healthy subjects or controls
Pathologic Effects of External-Beam Irradiation on Human Vocal Folds	Berg, Eric E.; Kolachala, Vasantha; Branski, Ryan C.; Muller, Susan; Johns, Michael M.	2011	No healthy subjects or controls
The Duration of Thigh Tourniquet Use Associated With Anterior Cruciate Ligament Reconstruction Does Not Produce Cellular-Level Contractile Dysfunction of the Quadriceps Muscle at 3 Weeks After Surgery	Beynnon, Bruce D.; Pius, Alexa K.; Tourville, Timothy W.; Endres, Nathan K.; Failla, Mathew J.; Choquette, Rebecca H.; DeSarno, Mike; Toth, Michael J.	2022	No healthy subjects or controls
Effects of Histidine and Î²-alanine Supplementation on Human Muscle Carnosine Storage	Blancquaert, Laura; Everaert, Inge; Missinne, Maarten; Baguet, Audrey; Stegen, Sanne; Volkaert, Anneke; Petrovic, Mirko; Vervaet, Chris; Achten, Eric; De Maeyer, Mieke; De Henauw, Stefaan; Derave, W. I. M.	2017	Did not perform fiber type analysis
Increased expression of GDF-15 may mediate ICU-acquired weakness by down-regulating muscle microRNAs	Bloch, S. A. A.; Lee, J. Y.; Syburra, T.; Rosendahl, U.; Griffiths, M. J. D.; Kemp, P. R.; Polkey, M. I.	2015	Does not include males and females
IRS-1 serine phosphorylation and insulin resistance in skeletal muscle from pancreas transplant recipients	Bouzakri, K.; Karlsson, H. K. R.; Vestergaard, H.; Madsbad, S.; Christiansen, E.; Zierath, J. R.; Bouzakri, Karim; Karlsson, HÃ¥kan K. R.; Vestergaard, Henrik; Madsbad, Sten; Christiansen, Erik; Zierath, Juleen R.	2006	Did not perform fiber type analysis
Metabolic demands and replenishment of muscle glycogen after a rugby league match simulation protocol	Bradley, Warren J.; Hannon, Marcus P.; Benford, Victoria; Morehen, James C.; Twist, Craig; Shepherd, Sam; Cocks, Matthew; Impey, Samuel G.; Cooper, Robert G.; Morton, James P.; Close, Graeme L.	2017	Does not include males and females
Muscle glycogen utilisation during Rugby match play: Effects of pre-game carbohydrate	Bradley, Warren J.; Morehen, James C.; Haigh, Julian; Clarke, Jon; Donovan, Timothy F.; Twist, Craig; Cotton, Caroline; Shepherd, Sam; Cocks, Matthew; Sharma, Asheesh; Impey, Samuel G.; Cooper, Robert G.; Maclaren, Don P. M.; Morton, James P.; Close, Graeme L.	2016	Does not include males and females
Effect of creatine and weight training on muscle creatine and performance in vegetarians	Burke, D. G.; Chilibeck, P. D.; Parise, G.; Candow, D. G.; Mahoney, D.; Tarnopolsky, M.	2003	Does not include male and female data
Apoptosis is present in skeletal muscle of cachectic gastro-intestinal cancer patients	Busquets, S.; Deans, C.; Figueras, M.; Moore-Carrasco, R.; LÃ³pez-Soriano, F. J.; Fearon, K. C.; ArgilÃ©s, J. M.	2007	Did not perform fiber type analysis
Inflammatory markers in skeletal muscle of older adults	Caldow, M. K.; Cameron-Smith, D.; Levinger, P.; McKenna, M. J.; Levinger, I.; Caldow, Marissa K.; Cameron-Smith, David; Levinger, Pazit; McKenna, Michael J.; Levinger, Itamar	2013	Did not perform fiber type analysis
Deregulation of microRNA-503 contributes to diabetes mellitus-induced impairment of	Caporali, A.; Meloni, M.; VÃ¶llenkle, C.; Bonci, D.; Sala-Newby, G. B.; Addis, R.; Spinetti, G.; Losa, S.; Masson, R.; Baker, A.	2011	No healthy subjects or controls

endothelial function and reparative angiogenesis after limb ischemia	H.; Agami, R.; le Sage, C.; Condorelli, G.; Madeddu, P.; Martelli, F.; Emanueli, C.; Caporali, Andrea; Meloni, Marco; VÃ¶llenkle, Christine; Bonci, Desiree		
Survival in critical illness is associated with early activation of mitochondrial biogenesis	CarrÃ©, J. E.; Orban, J. C.; Re, L.; Felsmann, K.; Iffert, W.; Bauer, M.; Suliman, H. B.; Piantadosi, C. A.; Mayhew, T. M.; Breen, P.; Stotz, M.; Singer, M.; CarrÃ©, Jane E.; Orban, Jean-Christophe; Re, Lorenza; Felsmann, Karen; Iffert, Wiebke; Bauer, Michael; Suliman, Hagir B.; Piantadosi, Claude A.	2010	Did not perform fiber type analysis
Skeletal muscle characteristics of people with multiple sclerosis	Carroll, C. C.; Gallagher, P. M.; Seidle, M. E.; Trappe, S. W.	2005	Does not report sex of subjects
The effect of zidovudine on skeletal muscle mtDNA in HIV-1 infected patients with mild or no muscle dysfunction	Casademont, J.; Barrientos, A.; Grau, J. M.; Pedrol, E.; Estivill, X.; Urbano-Marquez, A.; Nunes, V.; Casademont, J.; Barrientos, A.; Grau, J. M.; Pedrol, E.; Estivill, X.; Urbano-MÃ¡rquez, A.; Nunes, V.	1996	No healthy subjects or controls
Cytokine signature of inflammation mediated by autoreactive Th-cells, in calf muscle of claudicating patients with Fontaine stage II peripheral artery disease	Casale, George P.; Thompson, Jonathan R.; Carpenter, Lauren C.; Kim, Julian; Lackner, Timothy J.; Mietus, Constance J.; Ha, Duy M.; Myers, Sara A.; Brunette, Katyarina E.; Li, Shuai; Shields, Christina; Willcockson, Gregory; Pipinos, Iraklis I.	2021	Did not perform fiber type analysis
Unique Transcriptome Signature Distinguishes Patients With Heart Failure With Myopathy	Caspi, Talia; Straw, Sam; Cheng, Chew; Garnham, Jack O.; Scragg, Jason L.; Smith, Jessica; Koshy, Aaron O.; Levelt, Eylem; Sukumar, Piruthivi; Gierula, John; Beech, David J.; Kearney, Mark T.; Cubbon, Richard M.; Wheatcroft, Stephen B.; Witte, Klaus K.; Roberts, Lee D.; Bowen, T. Scott	2020	Did not perform fiber type analysis
Common errors in textbook descriptions of muscle fiber size in nontrained humans	Chalmers, Gordon R.; Row, Brandi S.	2011	Review (ex: systematic review/meta-analysis)
Permeabilised skeletal muscle reveals mitochondrial deficiency in malignant hyperthermia-susceptible individuals	Chang, Leon; Daly, Catherine; Miller, Dorota M.; Allen, Paul D.; Boyle, John P.; Hopkins, Philip M.; Shaw, Marie-Anne	2019	Did not perform fiber type analysis
Skeletal muscle myosin heavy chain synthesis in type 1 diabetes	Charlton, Michael R.; Balagopal, P.; Nair, K. Sreekumaran; Charlton, M. R.; Nair, K. S.	1997	Does not include male and female data
Early onset of inflammation and later involvement of TGFbeta in Duchenne muscular dystrophy	Chen, Y.; Nagaraju, K.; Bakay, M.; McIntyre, O.; Rawat, R.; Shi, R.; Hoffman, E. P.	2005	Only in children (0-17 years)
The effect of aerobic exercise training on the distribution of succinate dehydrogenase activity throughout muscle fibres	Chilibeck, P. D.; Bell, G. J.; Socha, T.; Martin, T.	1998	Does not include male and female data
Intramyocellular Lipid and Impaired Myofiber Contraction in Normal Weight and Obese Older Adults	Choi, Seung J.; Files, D. Clark; Tan, Zhang; Zhong-Min, Wang; Messi, Maria L.; Gregory, Heather; Stone, John; Lyles, Mary F.; Dhar, Sanjay; Marsh, Anthony P.;	2016	Does not include male and female data

	Nicklas, Barbara J.; Delbono, Osvaldo; Zhang, Tan; Wang, Zhong-Min		
Acute free fatty acid elevation eliminates endurance training effect on insulin sensitivity	Chow, L. S.; Seaquist, E. R.; Eberly, L. E.; Mashek, M. T.; Schimke, J. M.; Nair, K. S.; Mashek, D. G.; Chow, Lisa S.; Seaquist, Elizabeth R.; Eberly, Lynn E.; Mashek, Mara T.; Schimke, Jill M.; Nair, K. Sreekumaran; Mashek, Doug G.	2012	Did not perform fiber type analysis
Comparison of Two Î²-Alanine Dosing Protocols on Muscle Carnosine Elevations	Church, David D.; Hoffman, Jay R.; Varanoske, Alyssa N.; Wang, Ran; Baker, Kayla M.; La Monica, Michael B.; Beyer, Kyle S.; Dodd, Sarah J.; Oliveira, Leonardo P.; Harris, Roger C.; Fukuda, David H.; Stout, Jeffrey R.	2017	Did not perform fiber type analysis
Low anaerobic threshold and increased skeletal muscle lactate production in subjects with Huntington's disease	Ciammola, A.; Sassone, J.; Sciacco, M.; Mencacci, N. E.; Ripolone, M.; Bizzi, C.; Colciago, C.; Moggio, M.; Parati, G.; Silani, V.; Malfatto, G.; Ciammola, Andrea; Sassone, Jenny; Sciacco, Monica; Mencacci, NiccolÃ² E.; Ripolone, Michela; Bizzi, Caterina; Colciago, Clarissa; Moggio, Maurizio; Parati, Gianfranco	2011	Did not perform fiber type analysis
Influence of ageing and essential amino acids on quantitative patterns of troponin T alternative splicing in human skeletal muscle	Coble, Joel; Schilder, Rudolf J.; Berg, Arthur; Drummond, Micah J.; Rasmussen, Blake B.; Kimball, Scot R.	2015	Does not include males and females
Athletes with exercise-associated fatigue have abnormally short muscle DNA telomeres	Collins, M.; Renault, V.; Grobler, L. A.; St. Clair Gibson, A.; Lambert, M. I.; Derman, E. W.; Butler-Browne, G. S.; Noakes, T. D.; Mouly, V.	2003	Does not include male and female data
Muscle Fiber Hypertrophy and Myonuclei Addition: A Systematic Review and Meta-analysis	ConceiÃ‡ÃƒO, Miguel S.; Vechin, Felipe C.; LixandrÃƒO, Manoel; Damas, Felipe; Tricoli, Valmor; Roschel, Hamilton; Ugrinowitsch, Carlos; Libardi, Cleiton A.; Camera, Donny	2018	Review (ex: systematic review/meta-analysis)
Higher mitochondrial respiration and uncoupling with reduced electron transport chain content in vivo in muscle of sedentary versus active subjects	Conley, K. E.; Amara, C. E.; Bajpeyi, S.; Costford, S. R.; Murray, K.; Jubrias, S. A.; Arakaki, L.; Marcinek, D. J.; Smith, S. R.; Conley, Kevin E.; Amara, Catherine E.; Bajpeyi, Sudip; Costford, Sheila R.; Murray, Kori; Jubrias, Sharon A.; Arakaki, Lori; Marcinek, David J.; Smith, Steven R.	2013	Does not include males and females
Impairments in site-specific AS160 phosphorylation and effects of exercise training	Consitt, Leslie A.; Van Meter, Jessica; Newton, Christopher A.; Collier, David N.; Dar, Moahad S.; Wojtaszewski, JÃ¸rgen F. P.; Treebak, Jonas T.; Tanner, Charles J.; Houmard, Joseph A.	2013	Did not perform fiber type analysis
Rapid development of systemic insulin resistance with overeating is not accompanied by robust changes in skeletal muscle glucose and lipid metabolism	Cornford, Andrea S.; Hinko, Alexander; Nelson, Rachael K.; Barkan, Ariel L.; Horowitz, Jeffrey F.	2013	Did not perform fiber type analysis
Effect of serial cell passaging in the retention of fiber type and mitochondrial content in primary human myotubes	Covington, Jeffrey D.; Myland, Cassandra K.; Rustan, Arild C.; Ravussin, Eric; Smith, Steven R.; Bajpeyi, Sudip	2015	Does not include males and females

The effect of aging on human skeletal muscle mitochondrial and intramyocellular lipid ultrastructure	Crane, J. D.; Devries, M. C.; Safdar, A.; Hamadeh, M. J.; Tarnopolsky, M. A.; Crane, Justin D.; Devries, Michaela C.; Safdar, Adeel; Hamadeh, Mazen J.; Tarnopolsky, Mark A.	2010	Did not perform fiber type analysis
Glycogen levels in wildland firefighters during wildfire suppression	Cuddy, J. S.; Slivka, D. R.; Tucker, T. J.; Hailes, W. S.; Ruby, B. C.; Cuddy, John S.; Slivka, Dustin R.; Tucker, Tyler J.; Hailes, Walter S.; Ruby, Brent C.	2011	Did not perform fiber type analysis
Muscle characteristics, energy intake and expenditure in the dancer	Dahlstrom, M.	1997	Does not include males and females
Myosin Heavy Chain Composition of the Human Genioglossus Muscle	Daugherty, Megan; Luo, Qingwei; Sokoloff, Alan J.	2012	Autopsies
Heterotopic ossification in complex orthopaedic combat wounds: quantification and characterization of osteogenic precursor cell activity in traumatized muscle	Davis, T. A.; O'Brien, F. P.; Anam, K.; Grijalva, S.; Potter, B. K.; Elster, E. A.; Davis, Thomas A.; O'Brien, Frederick P.; Anam, Khairul; Grijalva, Steven; Potter, Benjamin K.; Elster, Eric A.	2011	Does not include males and females
Short-Term Adaptations in Skeletal Muscle Mitochondrial Oxidative Capacity and Metabolic Pathways to Breaking up Sedentary Behaviors in Overweight or Obese Adults	De Jong, Nathan P.; Rudolph, Michael C.; Jackman, Matthew R.; Sharp, Rachel R.; Jones, Ken; Houck, Julie; Pan, Zhaoxing; Reusch, Jane E. B.; MacLean, Paul S.; Bessesen, Daniel H.; Bergouignan, Audrey	2022	Did not perform fiber type analysis
Exercise-induced phosphorylation of the novel Akt substrates AS160 and filamin A in human skeletal muscle	Deshmukh, A.; Coffey, V. G.; Zhong, Z.; Chibalin, A. V.; Hawley, J. A.; Zierath, J. R.	2006	Does not include males and females
Mammalian target of rapamycin complex 1 activation is required for the stimulation of human skeletal muscle protein synthesis by essential amino acids	Dickinson, J. M.; Fry, C. S.; Drummond, M. J.; Gundermann, D. M.; Walker, D. K.; Glynn, E. L.; Timmerman, K. L.; Dhanani, S.; Volpi, E.; Rasmussen, B. B.; Dickinson, Jared M.; Fry, Christopher S.; Drummond, Micah J.; Gundermann, David M.; Walker, Dillon K.; Glynn, Erin L.; Timmerman, Kyle L.; Dhanani, Shaheen; Volpi, Elena; Rasmussen, Blake B.	2011	Did not perform fiber type analysis
Statin Treatment Decreases Mitochondrial Respiration But Muscle Coenzyme Q10 Levels Are Unaltered: The LIFESTAT Study	Dohlmann, Tine LovsÃ¸; Morville, Thomas; Kuhlman, Anja Birk; ChrÃ¸is, Karoline Maise; Helge, JÃ¸rn Wulff; Dela, Flemming; Larsen, Steen	2018	Did not perform fiber type analysis
Increased skeletal muscle-specific microRNA in the blood of patients with COPD	Donaldson, Anna; Natanek, Samantha A.; Lewis, Amy; Man, William D. C.; Hopkinson, Nicholas S.; Polkey, Michael I.; Kemp, Paul R.	2013	Does not include male and female data
Gastric cancer does not affect the expression of atrophy-related genes in human skeletal muscle	D'Orlando, Cristina; Marzetti, Emanuele; FranÃ§ois, Stephanie; Lorenzi, Maria; Conti, Valentina; di Stasio, Enrico; Rosa, Fausto; Brunelli, Silvia; Doglietto, Giovan Battista; Pacelli, Fabio; Bossola, Maurizio	2014	Did not perform fiber type analysis
Chronic Adaptations to Eccentric Training: A Systematic Review	Douglas, Jamie; Pearson, Simon; Ross, Angus; McGuigan, Mike	2017	Review (ex: systematic

			review/meta-analysis)
Amino acids are necessary for the insulin-induced activation of mTOR/S6K1 signaling and protein synthesis in healthy and insulin resistant human skeletal muscle	Drummond, M. J.; Bell, J. A.; Fujita, S.; Dreyer, H. C.; Glynn, E. L.; Volpi, E.; Rasmussen, B. B.	2008	Did not perform fiber type analysis
Regulation by insulin of gene expression in human skeletal muscle and adipose tissue. Evidence for specific defects in type 2 diabetes	Ducluzeau, Pierre-Henri; Perretti, Noel; Laville, Martine; Andreelli, Fabrizio; Vega, Nathalie; Riou, Jean-Paul; Vidal, Hubert; Ducluzeau, P. H.; Perretti, N.; Laville, M.; Andreelli, F.; Vega, N.; Riou, J. P.; Vidal, H.	2001	Did not perform fiber type analysis
Electromyography and muscle biopsy in chronic isolated Myalgia: A prospective study	Echanizâ€Laguna, Andoni; Chanson, Jeanâ€Baptiste	2016	Did not perform fiber type analysis
Evidence for low muscle capillary supply as a pathogenic factor in chronic compartment syndrome	Edmundsson, D.; Toolanen, G.; Thornell, L.; StÃ¥l, P.	2010	Does not include male and female data
Evidence for low muscle capillary supply as a pathogenic factor in chronic compartment syndrome Edmundsson et al. Capillary supply of lower leg muscle in CECS	Edmundsson, D.; Toolanen, G.; Thornell, Lâ€E; StÃ¥l, P.	2010	Does not include male and female data
Physical performance and muscular characteristics in different stages of COPD	Eliason, G.; Abdel-Halim, S.; Arvidsson, B.; Kadi, F.; Piehl-Aulin, K.	2009	Does not include male and female data
Alterations in the muscle-to-capillary interface in patients with different degrees of chronic obstructive pulmonary disease	Eliason, G.; Abdel-Halim, S. M.; Piehl-Aulin, K.; Kadi, F.; Eliason, Gabriella; Abdel-Halim, Samy M.; Piehl-Aulin, Karin; Kadi, Fawzi	2010	Does not include male and female data
Reduction in plasma leucine after sprint exercise is greater in males than in females	EsbjÃ¶rnsson, M.; Rooyackers, O.; Norman, B.; Rundqvist, H. C.; Nowak, J.; BÃ¼low, J.; Simonsen, L.; Jansson, E.	2012	Did not perform fiber type analysis
Gene expression of carnosine-related enzymes and transporters in skeletal muscle	Everaert, Inge; De Naeyer, HÃ©lÃ¨ne; Taes, Youri; Derave, Wim	2013	Did not perform fiber type analysis
Glycogen synthesis in muscle fibers during active recovery from intense exercise	Fairchild, T. J.; Armstrong, A. A.; Rao, A.; Liu, H.; Lawrence, S.; Fournier, P. A.	2003	Does not include males and females
Expression of insulin/IGF-I hybrid receptors is increased in skeletal muscle of patients with chronic primary hyperinsulinemia	Federici, Massimo; Lauro, Davide; D.Adamo, Monica; Giovannone, Barbara; Porzio, Ottavia; Mellozzi, Monica; Tamburrano, Guido; Sbraccia, Paolo; Sesti, Giorgio; Federici, M.; Lauro, D.; D'Adamo, M.; Giovannone, B.; Porzio, O.; Mellozzi, M.; Tamburrano, G.; Sbraccia, P.; Sesti, G.	1998	Did not perform fiber type analysis
Human Rotator Cuff Tears Have an Endogenous, Inducible Stem Cell Source Capable of Improving Muscle Quality and Function After Rotator Cuff Repair	Feeley, Brian T.; Liu, Mengyao; Ma, C. Benjamin; Agha, Obiajulu; Aung, Mya; Lee, Carlin; Liu, Xuhui	2020	Did not perform fiber type analysis
Hyperthermia, but not muscle water deficit, increases glycogen use during intense exercise	FernÃ¡ndez-ElÃas, V. E.; Hamouti, N.; Ortega, J. F.; Mora-RodrÃguez, R.	2015	Does not report sex of subjects
The Î²3-adrenergic receptor agonist mirabegron improves glucose homeostasis in obese humans	Finlin, Brian S.; Memetimin, Hasiyet; Beibei, Zhu; Confides, Amy L.; Vekaria, Hemendra J.; El Khouli, Riham H.; Johnson, Zachary R.;	2020	Does not include male and female data

	Westgate, Philip M.; Jianzhong, Chen; Morris, Andrew J.; Sullivan, Patrick G.; Dupont-Versteegden, Esther E.; Kern, Philip A.; Zhu, Beibei; Chen, Jianzhong		
Cell stress molecules in the skeletal muscle of GNE myopathy	Fischer, Charlotte; Kleinschnitz, Konstanze; Wrede, Arne; Muth, Ingrid; Kruse, Niels; Nishino, Ichizo; Schmidt, Jens	2013	Did not perform fiber type analysis
Males and females exhibit similar muscle glycogen recovery with varied recovery food sources	Flynn, Shannon; Rosales, Alejandro; Hailes, Walter; Ruby, Brent	2020	Did not perform fiber type analysis
The adaptations to strength training: morphological and neurological contributions to increased strength	Folland, J. P.; Williams, A. G.	2007	Review (ex: systematic review/meta-analysis)
Morphological changes of the multifidus muscle in patients with symptomatic lumbar disc herniation	Franke, J.; Hesse, T.; Tournier, C.; Schuberth, W.; Mawrin, C.; Lehuec, J. C.; Grasshoff, H.	2009	No healthy subjects or controls
Gender differences in muscular adaptation to strength training	Friedmann-Bette, B.	2008	Not peer reviewed (ex: thesis, conference poster)
Micro-RNA expression in muscle and fiber morphometry in myotonic dystrophy type 1	Fritegotto, Chiara; Ferrati, Chiara; Pegoraro, Valentina; Angelini, Corrado	2017	Does not include males and females
Muscle fiber and performance adaptations to resistance exercise with MyoVive, colostrum or casein and whey supplementation	Fry, A. C.; Schilling, B. K.; Chiu, L. Z. F.; Weiss, L. W.; Kreider, R. B.; Rasmussen, C. J.	2003	Does not include male and female data
Isokinetic strength testing in research and practice	Gaines, J. M.; Talbot, L. A.	1999	Review (ex: systematic review/meta-analysis)
GLUT4 is reduced in slow muscle fibers of type 2 diabetic patients: is insulin resistance in type 2 diabetes a slow, type 1 fiber disease?	Gaster, Michael; Staehr, Peter; Beck-Nielsen, Henning; Schroder, Henrick D.; Handberg, Aase; Gaster, M.; Staehr, P.; Beck-Nielsen, H.; SchrÃ¸der, H. D.; Handberg, A.	2001	Does not include males and females
Repeated high-intensity exercise modulates Ca2+ sensitivity of human skeletal muscle fibers	Gejl, K. D.; Hvid, L. G.; Willis, S. J.; Andersson, E.; Holmberg, H. â€C; Jensen, R.; Frandsen, U.; Hansen, J.; Plomgaard, P.; Ã˜rtenblad, N.	2016	Does not include males and females
Fibrosis, low vascularity, and fewer slow fibers after rotator-cuff injury	Gigliotti, Deanna; Xu, Mark C.; Davidson, Michael J.; Macdonald, Peter B.; Leiter, Jeff R. S.; Anderson, Judy E.	2017	Does not include male and female data
Autophagy markers LC3 and p62 accumulate in immune-mediated necrotizing myopathy	Girolamo, Francesco; Lia, Anna; Annese, Tiziana; Giannini, Margherita; Amati, Angela; D'Abbicco, Dario; Tampoia, Marilina; Virgintino, Daniela; Ribatti, Domenico; Serlenga, Luigi; Iannone, Florenzo; Trojano, Maria	2019	Does not include male and female data

10-20-30 training increases performance and lowers blood pressure and VEGF in runners	Gliemann, Lasse; Gunnarsson, Thomas P.; Hellsten, Ylva; Bangsbo, Jens	2015	Does not include male and female data
Mitochondrial coupling and capacity of oxidative phosphorylation in skeletal muscle of Inuit and Caucasians in the arctic winter	Gnaiger, E.; Boushel, R.; SÃ¸ndergaard, H.; Munchâ€Andersen, T.; Damsgaard, R.; Hagen, C.; DÃezâ€SÃ¡nchez, C.; Ara, I.; Wrightâ€Paradis, C.; Schrauwen, P.; Hesselink, M.; Calbet, J. A. L.; Christiansen, M.; Helge, J. W.; Saltin, B.	2015	Does not include male and female data
Muscle dysfunction in elderly individuals with hip fracture	Gonzalez-Crespo, M. R.; Arenas, J.; Gomez-Reino, J. J.; Campos, Y.; Borstein, B.; Martin, M. A.; Cabello, A.; Garcia-Rayo, R.; Ricoy, J. R.	1999	Did not perform fiber type analysis
Glucose ingestion causes GLUT4 translocation in human skeletal muscle	Goodyear, Laurie J.; Hirshman, Michael F.; Napoli, Raffaele; Calles, Jorge; Markuns, Jeffrey F.; Ljungqvist, Olle; Horton, Edward S.; Goodyear, L. J.; Hirshman, M. F.; Napoli, R.; Calles, J.; Markuns, J. F.; Ljungqvist, O.; Horton, E. S.	1996	Did not perform fiber type analysis
Metabolic response of muscle to alanine, glutamine, and valine supplementation during severe illness	Gore, D. C.; Wolfe, R. R.; Gore, Dennis C.; Wolfe, Robert R.	2003	Does not include males and females
Muscle fiber type IIX atrophy is involved in the loss of fat-free mass in chronic obstructive pulmonary disease	Gosker, H. R.; Engelen, M. P. K.; van Mameren, H.; van Dijk, P. J.; van der Vusse, G. J.; Wouters, E. F. M.; Schols, A. M. W.	2002	Does not include male and female data
Insulin resistance in morbid obesity: reversal with intramyocellular fat depletion	Greco, Aldo V.; Mingrone, Gertrude; Giancaterini, Annalisa; Manco, Melania; Morroni, Manrico; Cinti, Saverio; Granzotto, Marnie; Vettor, Roberto; Camastra, Stefania; Ferrannini, Ele	2002	Did not perform fiber type analysis
Skeletal muscle pathology in endurance athletes with acquired training intolerance	Grobler, L. A.; Collins, M.; Lambert, M. I.; Sinclair-Smith, C.; Derman, W.; Gibson, A. S.; Noakes, T. D.	2004	Does not include male and female data
Immunolocalization of interleukin-1 receptors in the sarcolemma and nuclei of skeletal muscle in patients with idiopathic inflammatory myopathies	Grundtman, C.; Salomonsson, S.; Dorph, C.; Bruton, J.; Andersson, U.; Lundberg, I. E.	2007	Did not perform fiber type analysis
Regulation and function of FTO mRNA expression in human skeletal muscle and subcutaneous adipose tissue	Grunnet, L. G.; Nilsson, E.; Ling, C.; Hansen, T.; Pedersen, O.; Groop, L.; Vaag, A.; Poulsen, P.; Grunnet, Louise G.; Nilsson, Emma; Ling, Charlotte; Hansen, Torben; Pedersen, Oluf; Groop, Leif; Vaag, Allan; Poulsen, Pernille	2009	Did not perform fiber type analysis
Morphometric and histochemical study of the human vocal muscle	Guida, H. L.; Zorzetto, N. L.	2000	Autopsies
Autophagy in locomotor muscles of patients with chronic obstructive pulmonary disease	Guo, Yeting; Gosker, Harry R.; Schols, Annemie M. W. J.; Kapchinsky, Sophia; Bourbeau, Jean; Sandri, Marco; Jagoe, R. Thomas; DebigarÃ©, Richard; Maltais, FranÃ§ois; Taivassalo, Tanja; Hussain, Sabah N. A.	2013	Did not perform fiber type analysis

Elevated tumor necrosis factor-alpha in skeletal muscle after stroke	Hafer-Macko, C. E.; Yu, S.; Ryan, A. S.; Ivey, F. M.; Macko, R. F.; Hafer-Macko, Charlene E.; Yu, Shuzhen; Ryan, Alice S.; Ivey, Frederick M.; Macko, Richard F.	2005	Did not perform fiber type analysis
Muscle CSA, force production, an activation of leg extensors during isometric and dynamic actions in middle-aged and elderly men and women	Hakkinen, K.; Alen, M.; Kallinen, M.; Izquierdo, M.; Jokelainen, K.; Lassila, H.; Malkia, E.; Kraemer, W. J.; Newton, R. U.	1998	Did not perform muscle biopsy
No Difference between Spray Dried Milk and Native Whey Supplementation with Strength Training	Hamarsland, HÃ¥Vard; Handegard, Vilde; KÃ…Shagen, Mauritz; Benestad, Haakon B.; Raastad, Truls	2019	Does not include male and female data
No effect of 25-hydroxyvitamin D supplementation on the skeletal muscle transcriptome in vitamin D deficient frail older adults	Hangelbroek, Roland W. J.; Vaes, Anouk M. M.; Boekschoten, Mark V.; Verdijk, Lex B.; Hooiveld, Guido J. E. J.; van Loon, Luc J. C.; de Groot, Lisette C. P. G. M.; Kersten, Sander	2019	Did not perform fiber type analysis
Exercise training normalizes skeletal muscle vascular endothelial growth factor levels in patients with essential hypertension	Hansen, A. H.; Nielsen, J. J.; Saltin, B.; Hellsten, Y.	2010	Does not include male and female data
Altered signaling for mitochondrial and myofibrillar biogenesis in skeletal muscles of patients with multiple sclerosis	Hansen, Dominique; Wens, Inez; Vandenabeele, Frank; Verboven, Kenneth; Eijnde, Bert O.	2015	Does not include male and female data
Relationship between efficiency and pedal rate in cycling: significance of internal power and muscle fiber type composition	Hansen, E. A.; SjÃ¸gaard, G.	2007	Does not include males and females
Respiratory muscle fiber morphometry. Correlation with pulmonary function and nutrition	Hards, J. M.; Reid, W. D.; Pardy, R. L.; ParÃ©, P. D.	1990	No healthy subjects or controls
Gender-Specific Differences in Skeletal Muscle 11Î²-HSD1 Expression Across Healthy Aging	Hassan-Smith, Zaki K.; Morgan, Stuart A.; Sherlock, Mark; Hughes, Beverly; Taylor, Angela E.; Lavery, Gareth G.; Tomlinson, Jeremy W.; Stewart, Paul M.	2015	Did not perform fiber type analysis
Impaired fat oxidation after a single high-fat meal in insulin-sensitive nondiabetic individuals with a family history of type 2 diabetes	Heilbronn, L. K.; Gregersen, S.; Shirkhedkar, D.; Hu, D.; Campbell, L. V.; Heilbronn, Leonie K.; Gregersen, SÃ¸ren; Shirkhedkar, Deepali; Hu, Dachun; Campbell, Lesley V.	2007	Did not perform fiber type analysis
Effects of regular resistance training on muscle histopathology and morphometry in elderly patients with chronic kidney disease	Heiwe, S.; Clyne, N.; TollbÃ¤ck, A.; Borg, K.	2005	Does not include male and female data
Growth hormone administration and exercise effects on muscle fiber and diameter in moderately frail older people	Hennessey, J. V.; Chromiak, J. A.; Della Ventura, S.; Reinert, S. E.; Puhl, J.; Kiel, D. P.; Rosen, C. J.; Vandenburgh, H.; MacLean, D. B.	2001	Does not include male and female data
An exploratory study of contractile force production in muscle fibers from patients with inflammatory myopathies	Henning, Franclo; Kohn, Tertius Abraham	2020	Does not include males and females
Acquired defects of glycogen synthase activity in cultured human skeletal muscle cells: influence of high glucose and insulin levels	Henry, Robert R.; Ciaraldi, Theodore P.; Mudaliar, Sunder; Abrams, Leslie; Nikoulina, Svetlana E.; Henry, R. R.; Ciaraldi, T. P.; Mudaliar, S.; Abrams, L.; Nikoulina, S. E.	1996	Does not include males and females

Subjects with early-onset type 2 diabetes show defective activation of the skeletal muscle PGC-1{alpha}/Mitofusin-2 regulatory pathway in response to physical activity	HernÃ¡ndez-Alvarez, M. I.; Thabit, H.; Burns, N.; Shah, S.; Brema, I.; Hatunic, M.; Finucane, F.; Liesa, M.; Chiellini, C.; Naon, D.; Zorzano, A.; Nolan, J. J.; HernÃ¡ndez-Alvarez, MarÃa Isabel; Thabit, Hood; Burns, Nicole; Shah, Syed; Brema, Imad; Hatunic, Mensud; Finucane, Francis; Liesa, Marc	2010	Does not include males and females
Muscle Oxygen Supply and Use in Type 1 Diabetes, From Ambient Air to the Mitochondrial Respiratory Chain: Is There a Limiting Step?	Heyman, Elsa; Daussin, FrÃ©dÃ©ric; Wieczorek, Valerie; Caiazzo, Robert; Matran, RÃ©gis; Berthon, PhanÃ©lie; Aucouturier, Julien; Berthoin, Serge; Descatoire, AurÃ©lien; Leclair, Erwan; Marais, GaÃ«lle; Combes, Adrien; Fontaine, Pierre; Tagougui, SÃ©mah	2020	Did not perform fiber type analysis
Noninvasive Monitoring of Training Induced Muscle Adaptation with (31)P-MRS: Fibre Type Shifts Correlate with Metabolic Changes	Hoff, Eike; Brechtel, Lars; Strube, Patrick; Konstanczak, Paul; Stoltenburg-Didinger, Gisela; Perka, Carsten; Putzier, Michael	2013	Does not include males and females
Targeted upregulation of pyruvate dehydrogenase kinase (PDK)-4 in slow-twitch skeletal muscle underlies the stable modification of the regulatory characteristics of PDK induced by high-fat feeding	Holness, Mark J.; Kraus, Alexandra; Harris, Robert A.; Sugden, Mary C.; Holness, M. J.; Kraus, A.; Harris, R. A.; Sugden, M. C.	2000	Animal study
The Muscle Protein Synthetic Response to Whey Protein Ingestion Is Greater in Middle-Aged Women Compared With Men	Horstman, A. M. H.; Kouw, I. W. K.; van Dijk, J. W.; Hamer, H. M.; Groen, B. B. L.; van Kranenburg, J.; Gorissen, S. H. M.; van Loon, L. J. C.; Horstman, Astrid M. H.; Kouw, Imre W. K.; van Dijk, Jan-Willem; Hamer, Henrike M.; Groen, Bart B. L.; van Kranenburg, Janneau; Gorissen, Stefan H. M.; van Loon, Luc J. C.	2018	Did not perform fiber type analysis
Insulin-regulated mitochondrial gene expression is associated with glucose flux in human skeletal muscle	Huang, X.; Eriksson, K. F.; Vaag, A.; Lehtovirta, M.; Hansson, M.; Laurila, E.; Kanninen, T.; Olesen, B. T.; Kurucz, I.; Koranyi, L.; Groop, L.	1999	Did not perform fiber type analysis
Impaired cathepsin L gene expression in skeletal muscle is associated with type 2 diabetes	Huang, Xudong; Vaag, Allan; Carlsson, Emma; Hansson, Mona; AhrÃ©n, Bo; Groop, Leif; AhrÃ©n, Bo	2003	Did not perform fiber type analysis
Transcriptional deficits in oxidative phosphorylation with statin myopathy	Hubal, M. J.; Reich, K. A.; De Biase, A.; Bilbie, C.; Clarkson, P. M.; Hoffman, E. P.; Thompson, P. D.; Hubal, Monica J.; Reich, Kimberly A.; De Biase, Andrea; Bilbie, Cherie; Clarkson, Priscilla M.; Hoffman, Eric P.; Thompson, Paul D.	2011	Did not perform fiber type analysis
Sex Dimorphism in Muscle Damage--induced Inflammation	Hui-Ying, L. U. K.; Levitt, Danielle E.; Appell, Casey; Vingren, Jakob L.	2021	Did not perform fiber type analysis
Proteomics analysis of human skeletal muscle reveals novel abnormalities in obesity and type 2 diabetes	Hwang, H.; Bowen, B. P.; Lefort, N.; Flynn, C. R.; De Filippis, E. A.; Roberts, C.; Smoke, C. C.; Meyer, C.; HÃ¸jlund, K.; Yi, Z.; Mandarino, L. J.; Hwang, Hyonson; Bowen, Benjamin P.; Lefort, Natalie; Flynn, Charles R.; De Filippis, Elena A.; Roberts, Christine;	2010	Did not perform fiber type analysis

	Smoke, Christopher C.; Meyer, Christian; HÃ¸jlund, Kurt		
Combined training enhances skeletal muscle mitochondrial oxidative capacity independent of age	Irving, Brian A.; Lanza, Ian R.; Henderson, Gregory C.; Rao, Rajesh R.; Spiegelman, Bruce M.; Nair, K. Sreekumaran	2015	Did not perform fiber type analysis
Lipid-induced insulin resistance in human muscle is associated with changes in diacylglycerol, protein kinase C, and IkappaB-alpha	Itani, Samar I.; Ruderman, Neil B.; Schmieder, Frank; Boden, Guenther	2002	Does not include males and females
Involvement of protein kinase C in human skeletal muscle insulin resistance and obesity	Itani, Samar I.; Zhou, Qian; Pories, Walter J.; MacDonald, Kenneth G.; Dohm, G. Lynis; Itani, S. I.; Zhou, Q.; Pories, W. J.; MacDonald, K. G.; Dohm, G. L.	2000	Does not include males and females
Decreased insulin responsiveness of glucose uptake in cultured human skeletal muscle cells from insulin-resistant nondiabetic relatives of type 2 diabetic families	Jackson, Sandra; Bagstaff, Stephanie M.; Lynn, Stephen; Yeaman, Stephen J.; Turnbull, Douglass M.; Walker, Mark; Jackson, S.; Bagstaff, S. M.; Lynn, S.; Yeaman, S. J.; Turnbull, D. M.; Walker, M.	2000	Did not perform fiber type analysis
Composition and adaptation of human myotendinous junction and neighboring muscle fibers to heavy resistance training	Jakobsen, J. R.; Mackey, A. L.; Knudsen, A. B.; Koch, M.; KjÃ¦r, M.; Krogsgaard, M. R.	2017	Did not perform fiber type analysis
The effect of strength training, recreational soccer and running exercise on stretch-shortening cycle muscle performance during countermovement jumping	Jakobsen, M. D.; Sundstrup, E.; Randers, M. B.; KjÃ¦r, M.; Andersen, L. L.; Krustrup, P.; Aagaard, P.	2012	Does not include males and females
Effect of limited ischemia time on the amount and function of mitochondria within human skeletal muscle cells	Jawhar, A.; Ponelies, N.; Schild, L.	2016	No healthy subjects or controls
Mitochondrial Function in an In Vitro Model of Skeletal Muscle of Patients With Protracted Critical Illness and Intensive Care Unit-Acquired Weakness	JiroutkovÃ¡, KateÅ™ina; KrajÄovÃ¡, AdÃ©la; ÅŽiak, Jakub; Fric, Michal; Gojda, Jan; DÅžupa, ValÃ©r; Kalous, Martin; TÅ¯movÃ¡, Jana; Trnka, Jan; DuÅ¡ka, FrantiÅ¡ek; Å½iak, Jakub; DÅ¾upa, ValÃ©r	2017	No healthy subjects or controls
Neuromuscular Electrical Stimulation Preserves Leg Lean Mass in Geriatric Patients	Karlsen, Anders; Cullum, Christopher Kjaer; Norheim, Kristoffer Larsen; Scheel, Frederik Ulrik; Zinglersen, Amanda Hempel; Vahlgren, Julie; Schjerling, Peter; Kjaer, Michael; Mackey, Abigail L.	2020	No cross-sectional area data
Differences in associations between HSD11B1 gene expression and metabolic parameters in subjects with and without impaired glucose homeostasis	Karlsson, C.; JernÃ¥s, M.; Olsson, B.; Lystig, T.; Gummesson, A.; Storlien, L.; Groop, L.; Carlsson, B.	2010	Did not perform fiber type analysis
Depressed Protein Synthesis and Anabolic Signaling Potentiate ACL Tearâ€“Resultant Quadriceps Atrophy	Keeble, Alexander R.; Brightwell, Camille R.; Latham, Christine M.; Thomas, Nicholas T.; Mobley, C. Brooks; Murach, Kevin A.; Johnson, Darren L.; Noehren, Brian; Fry, Christopher S.	2023	Does not include male and female data
Quantification and characterization of grouped type I myofibers in human aging	Kelly, Neil A.; Hammond, Kelley G.; Stec, Michael J.; Bickel, C. Scott; Windham, Samuel T.; Tuggle, S. Craig; Bamman, Marcas M.	2018	Does not include male and female data

Regulation of uncoupling protein-2 and uncoupling protein-3 mRNA expression during lipid infusion in human skeletal muscle and subcutaneous adipose tissue	Khalfallah, Yadh; Fages, Sophie; Laville, Martine; Langin, Dominique; Vidal, Hubert; Khalfallah, Y.; Fages, S.; Laville, M.; Langin, D.; Vidal, H.	2000	Does not include males and females
Expression of specific matrix metalloproteinases in inflammatory myopathies	Kieseier, B. C.; Schneider, C.; Clements, J. M.; Gearing, A. J.; Gold, R.; Toyka, K. V.; Hartung, H. P.	2001	No healthy subjects or controls
Classical PKC is not associated with defective insulin signaling in patients with impaired glucose tolerance	Kim do, M.; Jang, H. J.; Han, S. J.; Ha, E. S.; Kim, Y. K.; Park, J. W.; Song, K. E.; Jung, S. H.; Ahn, S. M.; Choi, S. E.; Kim, H. J.; Kim, D. J.; Lee, H. C.; Lee, K. W.; Kim, Do Min; Jang, Hyun Ju; Han, Seung Jin; Ha, Eun Suk; Kim, Yun Kyung; Park, Jee Won	2009	Did not perform fiber type analysis
Expression and functional significance of nicotinamide N-methyl transferase in skeletal muscles of patients with chronic obstructive pulmonary disease	Kim, H. C.; Mofarrahi, M.; Vassilakopoulos, T.; Maltais, F.; Sigala, I.; Debigare, R.; Bellenis, I.; Hussain, S. N.	2010	Does not include males and females
Human enterovirus in the gastrocnemius of patients with peripheral arterial disease	Kim, Julian K. S.; Zhu, Zhen; Casale, George; Koutakis, Panagiotis; McComb, Rodney D.; Swanson, Stanley; Thompson, Jonathan; Miserlis, Dimitrios; Johanning, Jason M.; Haynatzki, Gleb; Pipinos, Iraklis I.	2013	Did not perform fiber type analysis
Elevation of serum insulin concentration during euglycemic hyperinsulinemic clamp studies leads to similar activation of insulin receptor kinase in skeletal muscle of subjects with and without NIDDM	Klein, Harald H.; Vestergaard, Henrik; Kotzke, Gerd; Pedersen, Oluf; Klein, H. H.; Vestergaard, H.; Kotzke, G.; Pedersen, O.	1995	Did not perform fiber type analysis
Exercise pattern influences skeletal muscle hybrid fibers of runners and nonrunners	Kohn, T. A.; Essen-Gustavsson, B.; Myburgh, K. H.	2007	Does not report sex of subjects
Transcriptional levels of growth factors in skeletal muscle of maintenance hemodialysis patients...Proceedings of the 4th International Congress on Uremia Research and Toxicity, September 14-17, 2006, Izmir, Turkey	Kopple, J. D.; Wang, H.; Fournier, M.; Storer, T.; Zhang, S. M.; Song, H. Y.; Lewis, M.	2006	Did not perform fiber type analysis
Effects of exercise and insulin on insulin signaling proteins in human skeletal muscle	Koval, J. A.; Maezono, K.; Patti, M. E.; Pendergrass, M.; DeFronzo, R. A.; Mandarino, L. J.	1999	Did not perform fiber type analysis
Plasma Amino Acids Stimulate Uncoupled Respiration of Muscle Subsarcolemmal Mitochondria in Lean but Not Obese Humans	Kras, Katon A.; Hoffman, Nyssa; Roust, Lori R.; Patel, Shivam H.; Carroll, Chad C.; Katsanos, Christos S.	2017	Did not perform fiber type analysis
Insulin-stimulated Akt kinase activity is reduced in skeletal muscle from NIDDM subjects	Krook, Anna; Roth, Richard A.; Jiang, Xin Jian; Zierath, Juleen R.; Wallberg-Henriksson, Harriet; Krook, A.; Roth, R. A.; Jiang, X. J.; Zierath, J. R.; Wallberg-Henriksson, H.	1998	Does not include males and females
Insulin signaling and action in cultured skeletal muscle cells from lean healthy humans with high and low insulin sensitivity	KrÃ¼tzfeldt, J.; Kausch, C.; Volk, A.; Klein, H. H.; Rett, K.; HÃ¤ring, H. U.; Stumvoll, M.	2000	Did not perform fiber type analysis
Decreased expression of heat shock protein 72 in skeletal muscle of patients with type 2 diabetes correlates with insulin resistance	Kurucz, Istvan; Morva, Agota; Vaag, Allan; Eriksson, Karl-Fredrik; Huang, Xudong; Groop, Leif; Koranyi, Laszlo	2002	Did not perform fiber type analysis

Neither Peristaltic Pulse Dynamic Compressions nor Heat Therapy Accelerate Glycogen Resynthesis after Intermittent Running	Kyoungrae, K. I. M.; Kargl, Christopher K.; Bohyun, R. O.; Bohyun, Song; Stein, Kimberly; Gavin, Timothy P.; Roseguini, Bruno T.	2021	Did not perform fiber type analysis
Muscle diseases in elders: a 10-year retrospective study	Laguno, Montserrat; Miro, Oscar; Perea, Milagrosa; Picon, Marta; Urbano-Marquez, Alvaro; Grau, Josep M.; MirÃ³, Oscar; PicÃ³n, Marta; Urbano-MÃ¡rquez, Alvaro	2002	Does not report sex of subjects
Expression of genes related to muscle plasticity after strength and power training regimens	Lamas, L.; Aoki, M. S.; Ugrinowitsch, C.; Campos, G. E. R.; Regazzini, M.; Moriscot, A. S.; Tricoli, V.	2010	Does not include males and females
Enterovirus related metabolic myopathy: a postviral fatigue syndrome	Lane, R. J. M.; Soteriou, B. A.; Zhang, H.; Archard, L. C.; Lane, R. J. M.; Soteriou, B. A.; Zhang, H.; Archard, L. C.	2003	Did not perform fiber type analysis
Measurement of human skeletal muscle oxidative capacity by 31P-MR spectroscopy: a cross-validation with in vitro measurements	Lanza, I. R.; Bhagra, S.; Nair, K. S.; Port, J. D.; Lanza, Ian R.; Bhagra, Sumit; Nair, K. Sreekumaran; Port, John D.	2011	Did not perform fiber type analysis
The effect of high-intensity training on mitochondrial fat oxidation in skeletal muscle and subcutaneous adipose tissue	Larsen, S.; Danielsen, J. H.; SÃ¸ndergÃ¥rd, S. D.; SÃ¸gaard, D.; Vigelsoe, A.; Dybboe, R.; Skaaby, S.; Dela, F.; Helge, J. W.	2015	Did not perform fiber type analysis
Skeletal muscle morphology and aerobic capacity in patients with obstructive sleep apnoea syndrome	Larsson, B. W.; Kadi, F.; Ulfberg, J.; Aulin, K. P.	2008	Does not include male and female data
Determining the role of sarcomeric proteins in facioscapulohumeral muscular dystrophy: a study protocol	Lassche, Saskia; Ottenheijm, Coen Ac; Voermans, Nicol C.; Westeneng, Henk-Jan; Janssen, Barbara H.; van der Maarel, SilvÃ¨re M.; Hopman, Maria T.; Padberg, George W.; Stienen, Ger Jm; van Engelen, Baziel Gm; Ottenheijm, Coen A. C.; Stienen, Ger J. M.; van Engelen, Baziel G. M.	2013	Not peer reviewed (ex: thesis, conference poster)
Reduced specific force in patients with mild and severe facioscapulohumeral muscular dystrophy	Lassche, Saskia; Voermans, Nicol C.; Schreuder, Tim; Heerschap, Arend; KÃ¼sters, Benno; Ottenheijm, Coen A. C.; Hopman, Maria T. E.; Engelen, Baziel G. M.; van Engelen, Baziel Gm	2021	Does not include male and female data
Preserved single muscle fiber specific force in facioscapulohumeral muscular dystrophy	Lassche, Saskia; Voermans, Nicol C.; van der Pijl, Robbert; van den Berg, Marloes; Heerschap, Arend; van Hees, Hieronymus; Kusters, Benno; van der Maarel, SilvÃ¨re M.; Ottenheijm, Coen A. C.; van Engelen, Baziel G. M.	2020	Does not include male and female data
Changes in muscle fiber cross-sectional area and concentrations of Na, K-ATPase in deltoid muscle in patients with impingement syndrome of the shoulder	Leivseth, G.; Reikeras, O.	1994	Does not include male and female data
Quantification of lectin fluorescence in GNE myopathy muscle biopsies	Leoyklang, Petcharat; Class, Bradley; Noguchi, Satoru; Gahl, William A.; Carrillo, Nuria; Nishino, Ichizo; Huizing, Marjan; Malicdan, May Christine	2018	Did not perform fiber type analysis
Human aging, muscle mass, and fiber type composition	Lexell, J.	1995	Review (ex: systematic

			review/meta-analysis)
Strength training and muscle hypertrophy in older men and women	Lexell, J.	2000	Review (ex: systematic review/meta-analysis)
Passive force and viscoelastic properties of single fibers in human aging muscles	Lim, Jae-Young; Choi, Seung Jun; Widrick, Jeffrey J.; Phillips, Edward M.; Frontera, Walter R.	2019	Does not include male and female data
Response of growth and myogenic factors in humans skeletal muscle to strength training	Liu, Y.; Heinichen, M.; Wirth, K.; Schmidtbleicher, D.; Steinacker, J. M.	2008	Does not include males and females
Tenascin-C and alpha-smooth muscle actin positive cells are increased in the large airways in patients with COPD	LÃ¶fdahl, Magnus; Kaarteenaho, Riitta; Lappi-Blanco, Elisa; Tornling, GÃ¶ran; SkÃ¶ld, Magnus C.	2011	Did not perform fiber type analysis
Metformin to Augment Strength Training Effective Response in Seniors (MASTERS): study protocol for a randomized controlled trial	Long, Doug E.; Peck, Bailey D.; Martz, Jenny L.; Tuggle, S. Craig; Bush, Heather M.; McGwin, Gerald; Kern, Philip A.; Bamman, Marcas M.; Peterson, Charlotte A.	2017	Not peer reviewed (ex: thesis, conference poster)
Myoplasmic Ca2+ concentration during exertional rhabdomyolysis	LÃ³pez, J. R.; Rojas, B.; Gonzalez, M. A.; Terzic, A.	1995	Does not include males and females
Determinants of VO2 kinetics at high power outputs during a ramp exercise protocol	LucÃa, A.; Rivero, J. L.; PÃ©rez, M.; Serrano, A. L.; Calbet, J. A. L.; Santalla, A.; Chicharro, J. L.	2002	Does not include males and females
Skeletal muscle plasticity with marathon training in novice runners	Luden, N.; Hayes, E.; Minchev, K.; Louis, E.; Raue, U.; Conley, T.; Trappe, S.	2012	Does not include male and female data
Lower muscle regenerative potential in full-thickness supraspinatus tears compared to partial-thickness tears	Lundgreen, Kirsten; Lian, Ã˜ystein Bjerkestrand; Engebretsen, Lars; Scott, Alex	2013	No healthy subjects or controls
beta-Amyloid is a substrate of autophagy in sporadic inclusion body myositis	LÃ¼nemann, J. D.; Schmidt, J.; Schmid, D.; Barthel, K.; Wrede, A.; Dalakas, M. C.; MÃ¼nz, C.	2007	No healthy subjects or controls
Impaired expression of insulin-like growth factor-1 system in skeletal muscle of amyotrophic lateral sclerosis patients	Lunetta, C.; Serafini, M.; Prelle, A.; Magni, P.; Dozio, E.; Ruscica, M.; Sassone, J.; Colciago, C.; Moggio, M.; Corbo, M.; Silani, V.; Lunetta, Christian; Serafini, Massimo; Prelle, Alessandro; Magni, Paolo; Dozio, Elena; Ruscica, Massimiliano; Sassone, Jenny; Colciago, Clarissa; Moggio, Maurizio	2012	Does not include male and female data
Absence of developmental and unconventional myosin heavy chain in human suprahyoid muscles	Luo, Qingwei; Douglas, Megan; Burkholder, Thomas; Sokoloff, Alan J.	2014	Autopsies
Muscle connective tissue content of endurance-trained and inactive individuals	Mackey, A. L.; Donnelly, A. E.; Roper, H. P.	2005	Does not include male and female data
Reduced Physical Activity Alters the Leucine-Stimulated Translatome in Aged Skeletal Muscle	Mahmassani, Ziad S.; McKenzie, Alec I.; Petrocelli, Jonathan J.; Hart, Naomi M. de; Fix, Dennis K.; Kelly, Joshua J.; Baird, Lisa	2021	Does not include male and female data

	M.; Howard, Michael T.; Drummond, Micah J.; De Hart, Naomi M.		
Influence of peak VO2 and muscle fiber type on the efficiency of moderate exercise	Mallory, L. A.; Scheuermann, B. W.; Hoelting, B. D.; Weiss, M.; McAllister, R. M.; Barstow, T. J.	2002	Does not include male and female data
Human satellite cells have regenerative capacity and are genetically manipulable	Marg, Andreas; Escobar, Helena; Gloy, Sina; Kufeld, Markus; Zacher, Joseph; Spuler, Andreas; Birchmeier, Carmen; IzsvÃ¡k, Zsuzsanna; Spuler, Simone	2014	Did not perform fiber type analysis
Clinical value of a [18F]-FDG PET-CT muscle-to-muscle SUV ratio for the diagnosis of active dermatomyositis	Martis, Nihal; Viau, Philippe; Zenone, Thierry; Andry, Fanny; Grados, AurÃ©lie; Ebbo, Mikael; Castela, Emeline; Brihaye, Benoit; Denis, Eric; Liguori, StÃ©phane; Audemard, Alexandra; Schoindre, Yoland; Morin, Anne-Sophie; Terrier, Benjamin; Marcq, Laurent; Mounier, Nicolas; Lidove, Olivier; Chaborel, Jean-Philippe; Quinsat, Denis	2019	Did not perform fiber type analysis
Reduced HDAC2 in skeletal muscle of COPD patients	Masako, To; Swallow, Elisabeth B.; Akashi, Kenich; Haruki, Kosuke; Natanek, S. Amanda; Polkey, Michael I.; Kazuhiro, Ito; Barnes, Peter J.; To, Masako; Ito, Kazuhiro	2017	Did not perform fiber type analysis
Constitutive STAT3 phosphorylation contributes to skeletal muscle insulin resistance in type 2 diabetes	Mashili, F.; Chibalin, A. V.; Krook, A.; Zierath, J. R.; Mashili, Fredirick; Chibalin, Alexander V.; Krook, Anna; Zierath, Juleen R.	2013	Did not perform fiber type analysis
Changes of muscular fibre types in erector spinae and multifidus muscles in the unstable lumbar spine	Matejka, J.; Zuchova, M.; Koudela, K.; Pavelka, T.	2006	Does not include male and female data
Muscular adaptation to concentric and eccentric exercise at equal power levels	Mayhew, T. P.; Rothstein, J. M.; Finucane, S. D.; Lamb, R. L.	1995	Does not include male and female data
Increased pyruvate dehydrogenase kinase expression in cultured myotubes from obese and diabetic individuals	McAinch, A.; Cornall, L.; Watts, R.; Hryciw, D.; O'Brien, P.; Cameron-Smith, D.	2015	No healthy subjects or controls
Carbohydrate effect: hormone and oxidative changes	McAnulty, S.; McAnulty, L.; Nieman, D.; Morrow, J.; Dumke, C.; Utter, A.	2007	Does not include males and females
Statistical analysis of fiber area in human skeletal muscle	McGuigan, M. R. M.; Kraemer, W. J.; Deschenes, M. R.; Gordon, S. E.; Kitaura, T.; Scheett, T. P.; Sharman, M. J.; Staron, R. S.	2002	Does not include males and females
Superior Aerobic Capacity and Indices of Skeletal Muscle Morphology in Chronically Trained Master Endurance Athletes Compared With Untrained Older Adults	McKendry, James; Joanisse, Sophie; Baig, Shanat; Liu, Boyang; Parise, Gianni; Greig, Carolyn A.; Breen, Leigh	2019	Does not include males and females
Effect of local cold application during exercise on gene expression related to mitochondrial homeostasis	Meister, Ben; Collins, Chris; McGlynn, Mark; Slivka, Dustin	2021	Did not perform fiber type analysis
Elevated expression of prostaglandin e2 synthetic pathway in skeletal muscle of prior polio patients	Melin, Eva; Lindroos, Eva; Lundberg, Ingrid E.; Borg, Kristian; Korotkova, Marina	2014	Did not perform fiber type analysis

Effects of exercise on mitochondrial content and function in aging human skeletal muscle	Menshikova, E. V.; Ritov, V. B.; Fairfull, L.; Ferrell, R. E.; Kelley, D. E.; Goodpaster, B. H.; Menshikova, Elizabeth V.; Ritov, Vladimir B.; Fairfull, Liane; Ferrell, Robert E.; Kelley, David E.; Goodpaster, Bret H.	2006	Did not perform fiber type analysis
Decreased exercise-induced expression of nuclear factor-ÎºB-regulated genes in muscle of patients with COPD	Mercken, E. M.; Hageman, G. J.; Langen, R. C.; Wouters, E. F.; Schols, A. M.; Mercken, Evi M.; Hageman, Geja J.; Langen, Ramon C.; Wouters, Emiel F.; Schols, Annemie M.	2011	Does not include males and females
Skeletal muscle abnormalities and genetic factors related to vertical talus	Merrill, L. J.; Gurnett, C. A.; Connolly, A. M.; Pestronk, A.; Dobbs, M. B.; Merrill, Laura J.; Gurnett, Christina A.; Connolly, Anne M.; Pestronk, Alan; Dobbs, Matthew B.	2011	Only in children (0-17 years)
Increased expression of atrogenes and TWEAK family members after severe burn injury in nonburned human skeletal muscle	Merritt, Edward K.; Thalacker-Mercer, Anna; Cross, James M.; Windham, Samuel T.; Thomas, Steven J.; Bamman, Marcas M.	2013	Did not perform fiber type analysis
Resistance Training Enhances Skeletal Muscle Innervation Without Modifying the Number of Satellite Cells or their Myofiber Association in Obese Older Adults	Messi, MarÃa Laura; Tao, Li; Zhong-Min, Wang; Marsh, Anthony P.; Nicklas, Barbara; Delbono, Osvaldo; Li, Tao; Wang, Zhong-Min	2016	Does not include male and female data
Histological support for the difference between malignant hyperthermia susceptible (MHS), equivocal (MHE) and negative (MHN) muscle biopsies	Mezin, P.; Payen, J. F.; Bosson, J. L.; Brambilla, E.; Stieglitz, P.	1997	No healthy subjects or controls
Rosiglitazone improves downstream insulin receptor signaling in type 2 diabetic patients	Miyazaki, Yoshinori; He, Helen; Mandarino, Lawrence J.; DeFronzo, Ralph A.	2003	Did not perform fiber type analysis
Using laser capture microdissection to study fiber specific signaling in locomotor muscle in COPD: A pilot study	Mohan, Divya; Lewis, Amy; Patel, Mehul S.; Curtis, Katrina J.; Lee, Jen Y.; Hopkinson, Nicholas S.; Wilkinson, Ian B.; Kemp, Paul R.; Polkey, Michael I.	2017	Does not include males and females
Association between statin-associated myopathy and skeletal muscle damage	Mohaupt, M. G.; Karas, R. H.; Babiychuk, E. B.; Sanchez-Freire, V.; Monastyrskaya, K.; Iyer, L.; Hoppeler, H.; Breil, F.; Draeger, A.; Mohaupt, Markus G.; Karas, Richard H.; Babiychuk, Eduard B.; Sanchez-Freire, VerÃ³nica; Monastyrskaya, Katia; Iyer, Lakshmanan; Hoppeler, Hans; Breil, Fabio; Draeger, Annette	2009	Did not perform fiber type analysis
Exercise performance and skeletal muscles in patients with advanced Chagas disease	Montes de Oca, M.; Torres, S. H.; Loyo, J. G.; Vazquez, F.; HernÃ¡ndez, N.; Anchustegui, B.; PuigbÃ³, J. J.; Montes de Oca, MarÃa; Torres, Sonia H.; Loyo, JosÃ© G.; Vazquez, Francia; HernÃ¡ndez, Noelina; Anchustegui, BegoÃ±a; PuigbÃ³, Juan J.	2004	Does not include male and female data
BAIBA Does Not Regulate UCP-3 Expression in Human Skeletal Muscle as a Response to Aerobic Exercise	Morales, Flor E.; Forsse, Jeffrey S.; Andre, Thomas L.; McKinley-Barnard, Sarah K.; Hwang, Paul S.; Anthony, Ian G.; Tinsley, Grant M.; Spillane, Mike; Grandjean, Peter W.; Ramirez, Alejandro; Willoughby, Darryn S.	2017	Does not include males and females

Combined epigallocatechin-3-gallate and resveratrol supplementation for 12 wk increases mitochondrial capacity and fat oxidation, but not insulin sensitivity, in obese humans: a randomized controlled trial	Most, Jasper; Timmers, Silvie; Warnke, Ines; Jocken, Johan W. E.; van Boekschoten, Mark; de Groot, Philip; Bendik, Igor; Schrauwen, Patrick; Goossens, Gijs H.; Blaak, Ellen E.	2016	Did not perform fiber type analysis
Effects of endurance training on skeletal muscle mitochondrial function in Huntington disease patients	Mueller, Sandro Manuel; Gehrig, Saskia Maria; Petersen, Jens A.; Frese, Sebastian; Mihaylova, Violeta; Ligon-Auer, Maria; Khmara, Natalia; Nuoffer, Jean-Marc; Schaller, AndrÃ©; Lundby, Carsten; Toigo, Marco; Jung, Hans H.	2017	Does not include males and females
Satellite cell content in Huntington's disease patients in response to endurance training	Mueller, Sandro Manuel; Mihaylova, Violeta; Frese, Sebastian; Petersen, Jens A.; Ligon-Auer, Maria; Aguayo, David; FlÃ¼ck, Martin; Jung, Hans H.; Toigo, Marco	2019	Does not report sex of subjects
Exercise training-induced improvement in skeletal muscle PGC-1Î±-mediated fat metabolism is independent of dietary glycemic index	Mulya, Anny; Haus, Jacob M.; Solomon, Thomas P. J.; Kelly, Karen R.; Malin, Steven K.; Rocco, Michael; Barkoukis, Hope; Kirwan, John P.	2017	Did not perform fiber type analysis
AMP-activated protein kinase (AMPK) is activated in muscle of subjects with type 2 diabetes during exercise	Musi, Nicolas; Fujii, Nobuharu; Hirshman, Michael F.; Ekberg, Ingvar; Froberg, Sven; Ljungqvist, Olle; Thorell, Anders; Goodyear, Laurie J.; Musi, N.; Fujii, N.; Hirshman, M. F.; Ekberg, I.; FrÃ¶berg, S.; Ljungqvist, O.; Thorell, A.; Goodyear, L. J.	2001	Does not include males and females
ATP economy of force maintenance in human tibialis anterior muscle	Nakagawa, Y.; Ratkevicius, A.; Mizuno, M.; Quistorff, B.	2005	Does not include male and female data
Myotendinous alterations and effects of resistive loading in old age	Narici, M. V.; Maganaris, C. N.; Reeves, N. D.	2005	Review (ex: systematic review/meta-analysis)
Heterogeneity of quadriceps muscle phenotype in chronic obstructive pulmonary disease (Copd); implications for stratified medicine?	Natanek, Samantha A.; Gosker, Harry R.; Slot, Ilse G. M.; Marsh, Gemma S.; Hopkinson, Nicholas S.; Man, William D. C.; Tal-Singer, Ruth; Moxham, John; Kemp, Paul R.; Schols, Annemie M. W. J.; Polkey, Michael I.	2013	Does not include male and female data
Incidence of adverse events associated with percutaneous muscular biopsy among healthy and diseased subjects	Neves, Jr M.; Barreto, G.; Boobis, L.; Harris, R.; Roschel, H.; Tricoli, V.; Ugrinowitsch, C.; NegrÃ£o, C.; Gualano, B.	2012	Review (ex: systematic review/meta-analysis)
Relationship between muscle fiber composition and functional capacity of back muscles in healthy subjects and patients with back pain	Ng, J. K.; Richardson, C. A.; Kippers, V.; Parnianpour, M.	1998	Review (ex: systematic review/meta-analysis)
Carbohydrate-electrolyte ingestion during intermittent high-intensity running	Nicholas, C. W.; Tsintzas, K.; Boobis, L.; Williams, C.	1999	Does not include males and females
Alterations in quadriceps muscle cellular and molecular properties in adults with moderate knee osteoarthritis	Noehren, B.; Kosmac, K.; Walton, R. G.; Murach, K. A.; Lyles, M. F.; Loeser, R. F.; Peterson, C. A.; Messier, S. P.	2018	Does not include male and female data

Adaptations to Speed Endurance Training in Highly Trained Soccer Players	Nyberg, Michael; Fiorenza, Matteo; Lund, Anders; Christensen, Magnus; Rkmer, T. U. E.; Piil, Peter; Hostrup, Morten; Christensen, Peter M.; Holbek, Simon; Ravnholt, Thomas; Gunnarsson, Thomas P.; Bangsbo, Jens	2016	Does not include males and females
Altered fiber distribution and fiber-specific glycolytic and oxidative enzyme activity in skeletal muscle of patients with type 2 diabetes	Oberbach, A.; Bossenz, Y.; Lehmann, S.; Niebauer, J.; Adams, V.; Paschke, R.; SchÃ¶n, M. R.; BlÃ¼her, M.; Punkt, K.; Oberbach, Andreas; Bossenz, Yvonne; Lehmann, Stefanie; Niebauer, Josef; Adams, Volker; Paschke, Ralf; SchÃ¶n, Michael R.; BlÃ¼her, Matthias; Punkt, Karla	2006	Does not include male and female data
Diclofenac sodium (Voltaren) reduced exercise-induced injury in human skeletal muscle	O'Grady, M.; Hackney, A. C.; Schneider, K.; Bossen, E.; Steinberg, K.; Douglas, J. M., Jr.; Murray, W. J.; Watkins, W. D.	2000	Does not include males and females
Low-load resistance muscular training with moderate restriction of blood flow after anterior cruciate ligament reconstruction	Ohta, H.; Kurosawa, H.; Ikeda, H.; Iwase, Y.; Satou, N.; Nakamura, S.	2003	Does not include male and female data
Inflammatory cytokine response to exercise in alpha-1-antitrypsin deficient COPD patients 'on' or 'off' augmentation therapy	Olfert, I. Mark; Malek, Moh H.; Eagan, Tomas Ml; Wagner, Harrieth; Wagner, Peter D.; Eagan, Tomas M. L.	2014	Did not perform fiber type analysis
Structure of the Human Uvula	Olofsson, K.; Mattsson, C.; HammarstrÃ¶m, M.; HellstrÃ¶m, S.	1999	Did not perform fiber type analysis
Hepatocyte growth factor (HGF) and the satellite cell response following muscle lengthening contractions in humans	O'Reilly, C.; McKay, B.; Phillips, S.; Tarnopolsky, M.; Parise, G.; O'Reilly, Ciara; McKay, Bryon; Phillips, Stuart; Tarnopolsky, Mark; Parise, Gianni	2008	Does not include males and females
Effect of local heat application during exercise on gene expression related to mitochondrial homeostasis	O'Reilly, Nattie; Collins, Christopher; McGlynn, Mark L.; Slivka, Dustin	2021	Did not perform fiber type analysis
Diaphragm dysfunction in chronic obstructive pulmonary disease	Ottenheijm, C. A. C.; Heunks, L. M. A.; Sieck, G. C.; Zhan, W.; Jansen, S. M.; Degens, H.; de Boo, T.; Dekhuijzen, P. N. R.; Ottenheijm, Coen A. C.; Heunks, Leo M. A.; Sieck, Gary C.; Zhan, Wen-Zhi; Jansen, Suzanne M.; Degens, Hans; de Boo, Theo; Dekhuijzen, P. N. Richard	2005	Does not include male and female data
Insulin-induced hexokinase II expression is reduced in obesity and NIDDM	Pendergrass, Merri; Koval, Janice; Vogt, Christoph; Yki-Jarvinen, Hannele; Iozzo, Patricia; Pipek, Ruben; Ardehali, Hossein; Printz, Richard; Granner, Daryl; DeFronzo, Ralph A.; Mandarino, Lawrence J.; Pendergrass, M.; Koval, J.; Vogt, C.; Yki-Jarvinen, H.; Iozzo, P.; Pipek, R.; Ardehali, H.; Printz, R.; Granner, D.	1998	Did not perform fiber type analysis
Inflexibility in intramuscular triglyceride fractional synthesis distinguishes prediabetes from obesity in humans	Perreault, L.; Bergman, B. C.; Hunerdosse, D. M.; Playdon, M. C.; Eckel, R. H.; Perreault, Leigh; Bergman, Bryan C.; Hunerdosse, Devon M.; Playdon, Mary C.; Eckel, Robert H.	2010	Does not include male and female data

Biomarkers of Ectopic Fat Deposition: The Next Frontier in Serum Lipidomics	Perreault, Leigh; Starling, Anne P.; Glueck, Deborah; Brozinick, Joseph T.; Sanders, Phil; Siddall, Parker; Kuo, Ming Shang; Dabelea, Dana; Bergman, Bryan C.	2016	Did not perform fiber type analysis
High-intensity aerobic interval training increases fat and carbohydrate metabolic capacities in human skeletal muscle	Perry, C. G. R.; Heigenhauser, G. J. F.; Bonen, A.; Spriet, L. L.	2008	Did not perform fiber type analysis
Long-term effect of glycyl-glutamine after elective surgery on free amino acids in muscle	Petersson, B.; Waller, S. O.; Vinnars, E.; Wernerman, J.; Petersson, B.; Waller, S. O.; Vinnars, E.; Wernerman, J.	1994	No healthy subjects or controls
High oxidative capacity due to chronic exercise training attenuates lipid-induced insulin resistance	Phielix, E.; Meex, R.; Ouwens, D. M.; Sparks, L.; Hoeks, J.; Schaart, G.; Moonen-Kornips, E.; Hesselink, M. K.; Schrauwen, P.; Phielix, Esther; Meex, Ruth; Ouwens, D. Margriet; Sparks, Lauren; Hoeks, Joris; Schaart, Gert; Moonen-Kornips, Esther; Hesselink, Matthijs K. C.; Schrauwen, Patrick	2012	Does not include males and females
Mitochondrial dysfunction and lipid accumulation in the human diaphragm during mechanical ventilation	Picard, M.; Jung, B.; Liang, F.; Azuelos, I.; Hussain, S.; Goldberg, P.; Godin, R.; Danialou, G.; Chaturvedi, R.; Rygiel, K.; Matecki, S.; Jaber, S.; Rosiers, C. D.; Karpati, G.; Ferri, L.; Burelle, Y.; Turnbull, D. M.; Taivassalo, T.; Petrof, B. J.; Picard, Martin	2012	Did not perform fiber type analysis
Machine learning algorithms reveal unique gene expression profiles in muscle biopsies from patients with different types of myositis	Pinal-Fernandez, Iago; Casal-Dominguez, Maria; Derfoul, Assia; Pak, Katherine; Miller, Frederick W.; Milisenda, Jose CÃ©sar; Grau-Junyent, Josep Maria; Selva-O'Callaghan, Albert; Carrion-Ribas, Carme; Paik, Julie J.; Albayda, Jemima; Christopher-Stine, Lisa; Lloyd, Thomas E.; Corse, Andrea M.; Mammen, Andrew L.	2020	Does not report sex of subjects
Identification of distinctive interferon gene signatures in different types of myositis	Pinal-Fernandez, Iago M. D. PhD; Casal-Dominguez, Maria M. D. PhD; Derfoul, Assia PhD; Pak, Katherine M. D.; Plotz, Paul M. D.; Miller, Frederick W. M. D. PhD; Milisenda, Jose C. M. D.; Grau-Junyent, Josep M. M. D. PhD; Selva-O'Callaghan, Albert M. D. PhD; Paik, Julie M. D.; Albayda, Jemima M. D.; Christopher-Stine, Lisa M. D. M. P. H.; Lloyd, Thomas E. M. D. PhD; Corse, Andrea M. M. D.; Mammen, Andrew L. M. D. PhD; Pinal-Fernandez, Iago; Casal-Dominguez, Maria; Derfoul, Assia; Pak, Katherine; Plotz, Paul	2019	Did not perform fiber type analysis
Influence of resistance exercise intensity and metabolic stress on anabolic signaling and expression of myogenic genes in skeletal muscle	Popov, Daniil V.; Lysenko, Evgeny A.; Bachinin, Anton V.; Miller, Tatiana F.; Kurochkina, Nadezda S.; Kravchenko, Irina V.; Furalyov, Vladimir A.; Vinogradova, Olga L.	2015	Does not include males and females
Impact of genetic versus environmental factors on the control of muscle glycogen synthase activation in twins	Poulsen, Pernille; Wojtaszewski, JÃ¸rgen F. P.; Petersen, Inge; Christensen, Kaare;	2005	Did not perform fiber type analysis

	Richter, Erik A.; Beck-Nielsen, Henning; Vaag, Allan; Wojtaszewski, JÃ¸rgen F. P.		
Skeletal muscle insulin resistance in normoglycemic subjects with a strong family history of type 2 diabetes is associated with decreased insulin-stimulated insulin receptor substrate-1 tyrosine phosphorylation	Pratipanawatr, Wilailak; Pratipanawatr, Thongchai; Cusi, Kenneth; Berria, Rachele; Adams, John M.; Jenkinson, Christopher P.; Maezono, Katsumi; DeFronzo, Ralph A.; Mandarino, Lawrence J.; Pratipanawatr, W.; Pratipanawatr, T.; Cusi, K.; Berria, R.; Adams, J. M.; Jenkinson, C. P.; Maezono, K.; DeFronzo, R. A.; Mandarino, L. J.	2001	Did not perform fiber type analysis
Satellite cell response to concurrent resistance exercise and high-intensity interval training in sedentary, overweight/obese, middle-aged individuals	Pugh, Jamie K.; Faulkner, Steve H.; Turner, Mark C.; Nimmo, Myra A.	2018	Does not include male and female data
Kinome Profiling Reveals Abnormal Activity of Kinases in Skeletal Muscle From Adults With Obesity and Insulin Resistance	Qi, Yue; Zhang, Xiangmin; Seyoum, Berhane; Msallaty, Zaher; Mallisho, Abdullah; Caruso, Michael; Damacharla, Divyasri; Ma, Danjun; Al-Janabi, Wissam; Tagett, Rebecca; Alharbi, Majed; Calme, Griffin; Mestareehi, Aktham; Draghici, Sorin; Abou-Samra, Abdul; Kowluru, Anjaneyulu; Yi, Zhengping	2019	Did not perform fiber type analysis
Activity profile and physiological response to football training for untrained males and females, elderly and youngsters: influence of the number of players	Randers, M. B.; Nybo, L.; Petersen, J.; Nielsen, J. J.; Christiansen, L.; Bendiksen, M.; Brito, J.; Bangsbo, J.; Krustrup, P.	2010	Does not include male and female data
A signalling role for muscle glycogen in the regulation of pace during prolonged exercise	Rauch, H. G. L.; St Clair Gibson, A.; Lambert, E. V.; Noakes, T. D.	2005	Does not include males and females
Regional Myosin heavy chain distribution in selected paraspinal muscles	Regev, G. J.; Kim, C. W.; Thacker, B. E.; Tomiya, A.; Garfin, S. R.; Ward, S. R.; Lieber, R. L.; Regev, Gilad J.; Kim, Choll W.; Thacker, Bryan E.; Tomiya, Akihito; Garfin, Steven R.; Ward, Samuel R.; Lieber, Richard L.	2010	No healthy subjects or controls
Neuromuscular Electrical Stimulation Combined with Protein Ingestion Preserves Thigh Muscle Mass But Not Muscle Function in Healthy Older Adults During 5 Days of Bed Rest	Reidy, Paul T.; McKenzie, Alec I.; Brunker, Preston; Nelson, Daniel S.; Barrows, Katherine M.; Supiano, Mark; LaStayo, Paul C.; Drummond, Micah J.; Reidy, Paul; McKenzie, Alec; Nelson, Daniel; Barrows, Katherine; LaStayo, Paul; Drummond, Micah	2017	Does not include male and female data
Intrinsic properties of the adult human mylohyoid muscle: neural organization, fiber-type distribution, and myosin heavy chain expression	Ren, Min; Mu, Liancai	2005	Autopsies
Skeletal muscle ultrastructure and function in statin-tolerant individuals	Rengo, Jason L.; Callahan, Damien M.; Savage, Patrick D.; Ades, Philip A.; Toth, Michael J.	2016	Does not include male and female data
Single-fiber electromyography in hyperCKemia: the value of fiber density	Restivo, D. A.; Pavone, V.; Nicotra, A.; Restivo, D. A.; Pavone, V.; Nicotra, A.	2012	Did not perform muscle biopsy

Expression of NF-kappaB and IkappaB proteins in skeletal muscle of gastric cancer patients	Rhoads, M. G.; Kandarian, S. C.; Pacelli, F.; Doglietto, G. B.; Bossola, M.	2010	No healthy subjects or controls
Impact of rs361072 in the phosphoinositide 3-kinase p110beta gene on whole-body glucose metabolism and subunit protein expression in skeletal muscle	Ribel-Madsen, R.; Poulsen, P.; Holmkvist, J.; Mortensen, B.; Grarup, N.; Friedrichsen, M.; JÃ¸rgensen, T.; Lauritzen, T.; Wojtaszewski, J. F.; Pedersen, O.; Hansen, T.; Vaag, A.; Ribel-Madsen, Rasmus; Poulsen, Pernille; Holmkvist, Johan; Mortensen, Brynjulf; Grarup, Niels; Friedrichsen, Martin; JÃ¸rgensen, Torben; Lauritzen, Torsten	2010	Did not perform fiber type analysis
Familial resemblance for muscle phenotypes in the HERITAGE Family Study	Rico-Sanz, J.; Rankinen, T.; Joanisse, D. R.; Leon, A. S.; Skinner, J. S.; Wilmore, J. H.; Rao, D. C.; Bouchard, C.	2003	Does not include male and female data
Deficiency of subsarcolemmal mitochondria in obesity and type 2 diabetes	Ritov, Vladimir B.; Menshikova, Elizabeth V.; He, Jing; Ferrell, Robert E.; Goodpaster, Bret H.; E. Kelley, David; Kelley, David E.	2005	Did not perform fiber type analysis
The role of haemoglobin mass on VO2max following normobaric 'live high-train low' in endurance-trained athletes	Robach, P.; Siebenmann, C.; Jacobs, R. A.; Rasmussen, P.; Nordsborg, N.; Pesta, D.; Gnaiger, E.; DÃaz, V.; Christ, A.; Fiedler, J.; Crivelli, N.; Secher, N. H.; Pichon, A.; Maggiorini, M.; Lundby, C.	2012	Did not perform fiber type analysis
Inorganic Nitrate Mimics Exercise-Stimulated Muscular Fiber-Type Switching and Myokine and Î³-Aminobutyric Acid Release	Roberts, Lee D.; Ashmore, Tom; McNally, Ben D.; Murfitt, Steven A.; Fernandez, Bernadette O.; Feelisch, Martin; Lindsay, Ross; Siervo, Mario; Williams, Elizabeth A.; Murray, Andrew J.; Griffin, Julian L.	2017	Does not include male and female data
Exercise Induction of Key Transcriptional Regulators of Metabolic Adaptation in Muscle Is Preserved in Type 2 Diabetes	Sabaratnam, Rugivan; Pedersen, Andreas J.; Eskildsen, Tilde V.; Kristensen, Jonas M.; Wojtaszewski, JÃ¸rgen F. P.; HÃ¸jlund, Kurt	2019	Does not include males and females
Negative myoglobin staining in hemiplegic muscle of acute stroke patients predicts functional recovery	Sato, Y.; Iwamoto, J.; Kanoko, T.; Satoh, K.	2005	Full text not available
Angiotensin-converting enzyme gene expression in skeletal muscle in patients with chronic heart failure	Schaufelberger, M.; Drexler, H.; Schieffer, E.; Swedberg, K.	1998	Does not include male and female data
Skeletal muscle fiber composition and capillarization in patients with chronic heart failure: relation to exercise capacity and central hemodynamics	Schaufelberger, M.; Eriksson, B. O.; Grimby, G.; Held, P.; Swedberg, K.; Schaufelberger, M.; Eriksson, B. O.; Grimby, G.; Held, P.; Swedberg, K.	1995	Does not include male and female data
Load and failure behavior of human muscle samples in the context of proximal femur replacement	Schleifenbaum, Stefan; Schmidt, Michael; MÃ¶bius, Robert; WolfskÃ¤mpf, Thomas; SchrÃ¶der, Christian; Grunert, Ronny; Hammer, Niels; Prietzel, Torsten	2016	No healthy subjects or controls
The E3 ubiquitin ligase TRIM62 and inflammation-induced skeletal muscle atrophy	Schmidt, Franziska; Kny, Melanie; Zhu, Xiaoxi; Wollersheim, Tobias; Persicke, Kathleen; Langhans, Claudia; Lodka, Doerte; Kleber, Christian; Weber-Carstens, Steffen; Fielitz, Jens	2014	Did not perform fiber type analysis

The effect of a 3-month low-intensity endurance training program on fat oxidation and acetyl-CoA carboxylase-2 expression	Schrauwen, Patrick; van Aggel-Leijssen, Dorien P. C.; Hul, Gabby; Wagenmakers, Anton J. M.; Vidal, Hubert; Saris, Wim H. M.; van Baak, Marleen A.	2002	Does not include males and females
Muscle biopsy substantiates long-term MRI alterations one year after a single dose of botulinum toxin injected into the lateral gastrocnemius muscle of healthy volunteers	Schroeder, A. S.; Ertl-Wagner, B.; Britsch, S.; SchrÃ¶der, J. M.; Nikolin, S.; Weis, J.; MÃ¼ller-Felber, W.; Koerte, I.; Stehr, M.; Berweck, S.; Borggraefe, I.; Heinen, F.	2009	Does not include males and females
Effect of resistance exercise intensity on the expression of PGC-1Î± isoforms and the anabolic and catabolic signaling mediators, IGF-1 and myostatin, in human skeletal muscle	Schwarz, Neil A.; McKinley-Barnard, Sarah K.; Spillane, Mike B.; Andre, Thomas L.; Gann, Joshua J.; Willoughby, Darryn S.	2016	Does not include males and females
Bone Morphogenetic Proteins and myostatin pathways: key mediator of human sarcopenia	Scimeca, Manuel; Piccirilli, Eleonora; Mastrangeli, Francesca; Rao, Cecilia; Feola, Maurizio; Orlandi, Augusto; Gasbarra, Elena; Bonanno, Elena; Tarantino, Umberto	2017	Does not include male and female data
Human masseter muscle fiber type properties, skeletal malocclusions, and muscle growth factor expression	Sciote, J. J.; Horton, M. J.; Rowlerson, A. M.; Ferri, J.; Close, J. M.; Raoul, G.; Sciote, James Joseph; Horton, Michael J.; Rowlerson, Anthea M.; Ferri, Joel; Close, John M.; Raoul, Gwenael	2012	Does not include male and female data
Unloading shortening velocity and myosin heavy chain variations in human laryngeal muscle fibers	Sciote, J. J.; Morris, T. J.; Horton, M. J.; Brandon, C. A.; Rosen, C.	2002	No healthy subjects or controls
Acceleration of cutaneous healing by electrical stimulation: Degenerate electrical waveform down-regulates inflammation, up-regulates angiogenesis and advances remodeling in temporal punch biopsies in a human volunteer study	Sebastian, Anil; Syed, Farhatullah; Perry, Donna; Balamurugan, Vinayagapriya; Colthurst, James; Chaudhry, Iskander H.; Bayat, Ardeshir	2011	Did not perform muscle biopsy
Desmin and dystrophin abnormalities in upper airwayÂ muscles of snorers and patients with sleep apnea	Shah, Farhan; Franklin, Karl A.; Holmlund, ThorbjÃ¶rn; Levring JÃ¤ghagen, Eva; Berggren, Diana; Forsgren, Sture; StÃ¥l, Per	2019	Does not include males and females
Diagnostic outcome of muscle biopsy	Shaibani, Aziz; Jabari, Duaa; Jabbour, Moussa; Arif, Chia; Lee, Minjae; Rahbar, Mohammad Hossein	2015	No healthy subjects or controls
Effect of supraspinatus tendon injury on supraspinatus and infraspinatus muscle passive tension and associated biochemistry	Silldorff, Morgan D.; Choo, Alexander D.; Choi, Anthony J.; Lin, Evie; Carr, J. Austin; Lieber, Richard L.; Lane, John G.; Ward, Samuel R.	2014	No healthy subjects or controls
Histochemical and physiological correlates of training- and detraining-induced changes in the recovery from a fatigue test	Sinacore, D. R.; Coyle, E. F.; Hagberg, J. M.; Holloszy, J. O.	1993	Not peer reviewed (ex: thesis, conference poster)
Blood volume expansion does not explain the increase in peak oxygen uptake induced by 10Â weeks of endurance training	Skattebo, Ã˜yvind; Bjerring, Anders Wold; Auensen, Marius; Sarvari, Sebastian Imre; Cumming, Kristoffer Toldnes; Capelli, Carlo; HallÃ©n, Jostein	2020	Does not include male and female data
CALPAIN activity is increased in skeletal muscle from gastric cancer patients with no or minimal weight loss	Smith, I. J.; Aversa, Z.; Hasselgren, P. O.; Pacelli, F.; Rosa, F.; Doglietto, G. B.; Bossola, M.	2011	Did not perform fiber type analysis

Myosin heavy-chain composition of the human hyoglossus muscle	Sokoloff, A. J.; Daugherty, M.; Li, H.; Sokoloff, Alan J.; Daugherty, Megan; Li, Haiyan	2010	Autopsies
Vascular Defects and Spinal Cord Hypoxia in Spinal Muscular Atrophy	Somers, Eilidh; Lees, Robert D.; Hoban, Katie; Sleigh, James N.; Zhou, Haiyan; Muntoni, Francesco; Talbot, Kevin; Gillingwater, Thomas H.; Parson, Simon H.	2016	Only in children (0-17 years)
c-Jun NH2-terminal kinase activity in subcutaneous adipose tissue but not nuclear factor-kappaB activity in peripheral blood mononuclear cells is an independent determinant of insulin resistance in healthy individuals	Sourris, K. C.; Lyons, J. G.; de Courten, M. P.; Dougherty, S. L.; Henstridge, D. C.; Cooper, M. E.; Hage, M.; Dart, A.; Kingwell, B. A.; Forbes, J. M.; de Courten, B.; Sourris, Karly C.; Lyons, Jasmine G.; de Courten, Maximilian P. J.; Dougherty, Sonia L.; Henstridge, Darren C.; Cooper, Mark E.; Hage, Michelle; Dart, Anthony; Kingwell, Bronwyn A.	2009	Did not perform fiber type analysis
Differences in Mitochondrial Coupling Reveal a Novel Signature of Mitohormesis in Muscle of Healthy Individuals	Sparks, Lauren M.; Redman, Leanne M.; Conley, Kevin E.; Harper, Mary-Ellen; Hodges, Andrew; Eroshkin, Alexey; Costford, Sheila R.; Gabriel, Meghan E.; Yi, Fanchao; Shook, Cherie; Cornnell, Heather H.; Ravussin, Eric; Smith, Steven R.	2016	Does not include male and female data
Effects of 12 Months of Caloric Restriction on Muscle Mitochondrial Function in Healthy Individuals	Sparks, Lauren Marie; Redman, Leanne M.; Conley, Kevin E.; Harper, Mary-Ellen; Yi, Fanchao; Hodges, Andrew; Eroshkin, Alexey; Costford, Sheila R.; Gabriel, Meghan E.; Shook, Cherie; Cornnell, Heather H.; Ravussin, Eric; Smith, Steven R.; Sparks, Lauren M.	2016	Did not perform fiber type analysis
The possible role of locally produced cytokines in the pathogenesis of peritrochanteric fractures in the elderly	Spiliopoulou, I.; Korovessis, P.; Stamatakis, M.; Paliogianni, F.; Dimitracopoulos, G.; Spiliopoulou, I.; Korovessis, P.; Stamatakis, M.; Paliogianni, F.; Dimitracopoulos, G.	2001	Did not perform fiber type analysis
Myopathy causing camptocormia in idiopathic Parkinson's disease: a multidisciplinary approach	Spuler, S.; Krug, H.; Klein, C.; Medialdea, I. C.; Jakob, W.; Ebersbach, G.; Gruber, D.; Hoffmann, K. T.; Trottenberg, T.; Kupsch, A.; Spuler, Simone; Krug, Henriette; Klein, Christine; Medialdea, Isabel Chaure; Jakob, Wibke; Ebersbach, Georg; Gruber, Doreen; Hoffmann, Karl-Titus; Trottenberg, Thomas; Kupsch, Andreas	2010	No healthy subjects or controls
Effect of acute exercise on AMPK signaling in skeletal muscle of subjects with type 2 diabetes: a time-course and dose-response study	Sriwijitkamol, A.; Coletta, D. K.; Wajcberg, E.; Balbontin, G. B.; Reyna, S. M.; Barrientes, J.; Eagan, P. A.; Jenkinson, C. P.; Cersosimo, E.; Defronzo, R. A.; Sakamoto, K.; Musi, N.; Sriwijitkamol, Apiradee; Coletta, Dawn K.; Wajcberg, Estela; Balbontin, Gabriela B.; Reyna, Sara M.; Barrientes, John; Eagan, Phyllis A.; Jenkinson, Christopher P.	2007	Did not perform fiber type analysis

Skeletal Muscle Energetics and Mitochondrial Function Are Impaired Following 10 Days of Bed Rest in Older Adults	Standley, Robert A.; Distefano, Giovanna; Trevino, Michelle B.; Chen, Emily; Narain, Niven R.; Greenwood, Bennett; Kondakci, Gramoz; Tolstikov, Vladimir V.; Kiebish, Michael A.; Yu, Gongxin; Qi, Feng; Kelly, Daniel P.; Vega, Rick B.; Coen, Paul M.; Goodpaster, Bret H.	2020	Does not include male and female data
Fiber-type changes found in muscles of patients with low back pain: gender differences were identified in fiber-type distribution and size	Stephenson, M.	2001	Full text not available
Global gene expression in skeletal muscle from well-trained strength and endurance athletes	Stepto, N. K.; Coffey, V. G.; Carey, A. L.; Ponnampalam, A. P.; Canny, B. J.; Powell, D.; Hawley, J. A.	2009	Does not include males and females
Overexpression of GLUT5 in diabetic muscle is reversed by pioglitazone	Stuart, C. A.; Howell, M. E. A.; Yin, D.	2007	No cross-sectional area data
Insulin Responsiveness in Metabolic Syndrome after Eight Weeks of Cycle Training	Stuart, Charles A.; South, Mark A.; Lee, Michelle L.; McCurry, Melanie P.; Howell, Mary E. A.; Ramsey, Michael W.; Stone, Michael H.	2013	Does not include male and female data
Major histocompatibility complex class I expression can be used as a diagnostic tool to differentiate idiopathic inflammatory myopathies from dystrophies	Sundaram, C.; Uppin, M. S.; Meena, A. K.; Sundaram, C.; Uppin, Megha S.; Meena, A. K.	2008	Does not include male and female data
Endurance training improves fitness and strength in patients with Becker muscular dystrophy	Sveen, M. L.; Jeppesen, T. D.; Hauerslev, S.; KÃ¸ber, L.; Krag, T. O.; Vissing, J.	2008	Does not include males and females
Increased capillaries in mitochondrial myopathy: implications for the regulation of oxygen delivery	Taivassalo, T.; Ayyad, K.; Haller, R. G.; Taivassalo, Tanja; Ayyad, Karen; Haller, Ronald G.	2012	Does not include male and female data
Autophagic-lysosomal pathway is the main proteolytic system modified in the skeletal muscle of esophageal cancer patients	Tardif, Nicolas; Klaude, Maria; Lundell, Lars; Thorell, Anders; Rooyackers, Olav	2013	Did not perform fiber type analysis
Suction-modified BergstrÃ¶m muscle biopsy technique: Experience with 13,500 procedures	Tarnopolsky, M. A.; Pearce, E.; Smith, K.; Lach, B.	2011	Did not perform fiber type analysis
Anatomy and fiber type composition of human interarytenoid muscle	Tellis, C. M.; Thekdi, A.; Rosen, C.; Sciote, J. J.	2004	No healthy subjects or controls
A decade of aerobic endurance training: histological evidence for fibre type transformation	Thayer, R.; Collins, J.; Noble, E. G.; Taylor, A. W.	2000	Does not include males and females
Regenerative defect in vastus lateralis muscle of patients with chronic obstructive pulmonary disease	ThÃ©riault, Marie-Eve; ParÃ©, Marie-Eve; Lemire, Bruno B.; Maltais, FranÃ§ois; DebigarÃ©, Richard; ParÃ©, Marie-Ãˆve	2014	Does not include males and females
Skeletal muscle TLR4 and TACE are associated with body fat percentage in older adults	Timmerman, Kyle L.; Connors, Ian D.; Deal, Michael A.; Mott, Rachael E.	2016	Did not perform fiber type analysis
Marathon runners: how do they age?	Trappe, S.	2007	Review (ex: systematic review/meta-analysis)

Local anesthetic effects on gene transcription in human skeletal muscle biopsies	Trappe, Todd A.; Standley, Robert A.; Liu, Sophia Z.; Jemiolo, Bozena; Trappe, Scott W.; Harber, Matthew P.	2013	Does not include males and females
Respirometric Profiling of Muscle Mitochondria and Blood Cells Are Associated With Differences in Gait Speed Among Community-Dwelling Older Adults	Tyrrell, Daniel J.; Bharadwaj, Manish S.; Van Horn, Cynthia G.; Kritchevsky, Stephen B.; Nicklas, Barbara J.; Molina, Anthony J. A.	2015	Did not perform fiber type analysis
Variable inflammation and intramuscular STAT3 phosphorylation and myeloperoxidase levels after downhill running	van de Vyver, M.; Myburgh, K. H.	2014	Does not include males and females
Levosimendan enhances force generation of diaphragm muscle from patients with chronic obstructive pulmonary disease	van Hees, H. W.; Dekhuijzen, P. N.; Heunks, L. M.	2009	Does not include male and female data
Expression of protein kinase C isoforms and interleukin-1beta in myofibrillar myopathy	Vattemi, G.; Tonin, P.; Mora, M.; Filosto, M.; Morandi, L.; Savio, C.; Dal Pra, I.; Rizzuto, N.; Tomelleri, G.	2004	No healthy subjects or controls
The Role of Muscle Glycogen Content and Localization in High-Intensity Exercise Performance: A Placebo-Controlled Trial	Vigh-Larsen, Jeppe F.; Ã˜Rtenblad, Niels; Nielsen, Joachim; Emil Andersen, O. L. E.; Overgaard, Kristian; Mohr, Magni	2022	Does not include males and females
Differentiated mTOR but not AMPK signaling after strength vs endurance exercise in training-accustomed individuals	Vissing, K.; McGee, S. L.; Farup, J.; KjÃ¸lhede, T.; Vendelbo, M. H.; Jessen, N.	2013	Does not include males and females
Aquaporin 4 expression in human skeletal muscle fiber types	Vizzaccaro, Elisa; Terracciano, Chiara; Rastelli, Emanuele; Massa, Roberto	2018	No cross-sectional area data
Effect of pulmonary rehabilitation on peripheral muscle fiber remodeling in patients with COPD in GOLD stages II to IV	Vogiatzis, I.; Terzis, G.; Stratakos, G.; Cherouveim, E.; Athanasopoulos, D.; Spetsioti, S.; Nasis, I.; Manta, P.; Roussos, C.; Zakynthinos, S.; Vogiatzis, Ioannis; Terzis, Gerasimos; Stratakos, Grigoris; Cherouveim, Evgenia; Athanasopoulos, Dimitris; Spetsioti, Stauroula; Nasis, Ioannis; Manta, Panagiota; Roussos, Charis; Zakynthinos, Spyros	2011	Does not include males and females
Histomorphologic examination of skeletal muscle preparations does not differentiate between malignant hyperthermia-susceptible and -normal patients	von Breunig, Franziska; Wappler, Frank; Hagel, Christian; von Richthofen, Verena; Fiege, Marko; Weisshorn, Ralf; Stavrou, Dimitrios; Schulte am Esch, Jochen	2004	Does not include male and female data
Altered autophagy gene expression and persistent atrophy suggest impaired remodeling in chronic hemiplegic human skeletal muscle	von Walden, F.; Jakobsson, F.; EdstrÃ¶m, L.; Nader, G. A.	2012	Does not include male and female data
Activation of the Pro-Oxidant PKCÎ²II-p66Shc Signaling Pathway Contributes to Pericyte Dysfunction in Skeletal Muscles of Patients With Diabetes With Critical Limb Ischemia	Vono, Rosa; Fuoco, Claudia; Testa, Stefano; PirrÃ², Stefano; Maselli, Davide; McCollough, David Ferland; Sangalli, Elena; Pintus, Gianfranco; Giordo, Roberta; Finzi, Giovanna; Sessa, Fausto; Cardani, Rosanna; Gotti, Ambra; Losa, Sergio; Cesareni, Gianni; Rizzi, Roberto; Bearzi, Claudia; Cannata, Stefano; Spinetti, Gaia; Gargioli, Cesare	2016	Does not include male and female data

Acute Hypoglycemia in Healthy Humans Impairs Insulin-Stimulated Glucose Uptake and Glycogen Synthase in Skeletal Muscle: A Randomized Clinical Study	Voss, Thomas S.; Vendelbo, Mikkel H.; Kampmann, Ulla; Hingst, Janne R.; Wojtaszewski, JÃ¸rgen F. P.; Svart, Mads V.; MÃ¸ller, Niels; Jessen, Niels	2017	Did not perform fiber type analysis
Troglitazone downregulates delta-6 desaturase gene expression in human skeletal muscle cell cultures	Wahl, Hans GÃ¼nther; Kausch, Christiana; Machicao, Fausto; Rett, Kristian; Stumvoll, Michael; HÃ¤ring, Hans-Ulrich	2002	Did not perform fiber type analysis
The expression of vascular endothelial growth factor in skeletal muscle of patients with sleep disorders	WÃ¥hlin-Larsson, B.; Ulfberg, J.; Aulin, K. P.; Kadi, F.	2009	Does not report sex of subjects
Type I interferon-inducible gene expression in blood is present and reflects disease activity in dermatomyositis and polymyositis	Walsh, R. J.; Kong, S. W.; Yao, Y.; Jallal, B.; Kiener, P. A.; Pinkus, J. L.; Beggs, A. H.; Amato, A. A.; Greenberg, S. A.	2007	Did not perform fiber type analysis
Urinary excretion of an intravenous 26Mg dose as an indicator of marginal magnesium deficiency in adults	WÃ¤lti, M. K.; Walczyk, T.; Zimmermann, M. B.; Fortunato, G.; Weber, M.; Spinas, G. A.; Hurrell, R. F.	2006	Did not perform fiber type analysis
Epinephrine infusion does not enhance net muscle glycogenolysis during prolonged aerobic exercise	Wendling, P. S.; Peters, S. J.; Heigenhauser, G. J. F.; Spriet, L. L.	1996	Did not perform fiber type analysis
Walking performance is positively correlated to calf muscle fiber size in peripheral artery disease subjects, but fibers show aberrant mitophagy: an observational study	White, Sarah H.; McDermott, Mary M.; Sufit, Robert L.; Kosmac, Kate; Bugg, Alex W.; Gonzalez-Freire, Marta; Ferrucci, Luigi; Tian, Lu; Zhao, Lihui; Gao, Ying; Kibbe, Melina R.; Criqui, Michael H.; Leeuwenburgh, Christiaan; Peterson, Charlotte A.	2016	Does not include male and female data
Histochemical and morphological characteristics of the vastus lateralis muscle in patients with chronic obstructive pulmonary disease	Whittom, F.; Jobin, J.; Simard, P.; Leblanc, P.; Simard, C.; Bernard, S.; Belleau, R.; Maltais, F.	1998	Does not include male and female data
Limited diagnostic value of enzyme analysis in patients with mitochondrial tRNA mutations	Wibrand, F.; Jeppesen, T. D.; Frederiksen, A. L.; Olsen, D. B.; Duno, M.; Schwartz, M.; Vissing, J.	2010	Did not perform fiber type analysis
Muscular heat shock protein response and muscle damage after semiâ€professional football match	Wiig, HÃ¥vard; Cumming, Kristoffer T.; Handegaard, Vilde; Stabell, Jostein; Spencer, Matthew; Raastad, Truls	2022	Does not include males and females
Increased cardiorespiratory fitness and skeletal muscle size following single-leg knee extension exercise training	Wolff, Christopher A.; Konopka, Adam R.; Suer, Miranda K.; Trappe, Todd A.; Kaminsky, Leonard A.; Harber, Matthew P.	2019	Does not include male and female data
Dynamics of myosin degradation in intensive care unit-acquired weakness during severe critical illness	Wollersheim, Tobias; Woehlecke, Janine; Krebs, Martin; Hamati, Jida; Lodka, Doerte; Luther-Schroeder, Anja; Langhans, Claudia; Haas, Kurt; Radtke, Theresa; Kleber, Christian; Spies, Claudia; Labeit, Siegfried; Schuelke, Markus; Spuler, Simone; Spranger, Joachim; Weber-Carstens, Steffen; Fielitz, Jens	2014	Does not include male and female data
Skeletal muscle capillarization and oxidative metabolism in healthy smokers	WÃ¼st, R. C. I.; Jaspers, R. T.; van der Laarse, W. J.; Degens, H.	2008	Does not include male and female data
Secreted Frizzled-Related Protein 2 and Inflammation-Induced Skeletal Muscle Atrophy	Xiaoxi, Zhu; Kny, Melanie; Schmidt, Franziska; Hahn, Alexander; Wollersheim,	2017	No healthy subjects or controls

	Tobias; Kleber, Christian; Weber-Carstens, Steffen; Fielitz, Jens; Zhu, Xiaoxi		
Exercise, aging, and muscle protein metabolism	Yarasheski, Kevin E.	2003	Review (ex: systematic review/meta-analysis)
Global assessment of regulation of phosphorylation of insulin receptor substrate-1 by insulin in vivo in human muscle	Yi, Z.; Langlais, P.; De Filippis, E. A.; Luo, M.; Flynn, C. R.; Schroeder, S.; Weintraub, S. T.; Mapes, R.; Mandarino, L. J.; Yi, Zhengping; Langlais, Paul; De Filippis, Elena A.; Luo, Moulun; Flynn, Charles R.; Schroeder, Stefanie; Weintraub, Susan T.; Mapes, Rebekka; Mandarino, Lawrence J.	2007	Did not perform fiber type analysis
Alterations of signaling pathways in muscle tissues of patients with amyotrophic lateral sclerosis	Yin, F.; Ye, F.; Tan, L.; Liu, K.; Xuan, Z.; Zhang, J.; Wang, W.; Zhang, Y.; Jiang, X.; Zhang, D. Y.	2012	Did not perform fiber type analysis
Immunohistochemical quantification of heparan sulfate proteoglycan and collagen IV in skeletal muscle capillary basement membranes of patients with diabetic nephropathy	Yokoyama, Hiroki; HÃ¸yer, Poul E.; Hansen, Pernille M.; Van den Born, Jacob; Jensen, Tonny; Berden, Jo H. M.; Deckert, Torsten; Garbarsch, Charly; Yokoyama, H.; HÃ¸yer, P. E.; Hansen, P. M.; van den Born, J.; Jensen, T.; Berden, J. H.; Deckert, T.; Garbarsch, C.	1997	Did not perform fiber type analysis
Skeletal muscle content of membrane glycoprotein PC-1 in obesity. Relationship to muscle glucose transport	Youngren, J. F.; Maddux, B. A.; Sasson, S.; Sbraccia, P.; Tapscott, E. B.; Swanson, M. S.; Dohm, G. L.; Goldfine, I. D.; Youngren, J. F.; Maddux, B. A.; Sasson, S.; Sbraccia, P.; Tapscott, E. B.; Swanson, M. S.; Dohm, G. L.; Goldfine, I. D.	1996	Did not perform fiber type analysis
Contribution of Nonesterified Fatty Acids to Mitogen-Activated Protein Kinase Activation in Human Skeletal Muscle During Endurance Exercise	Zbinden-Foncea, Hermann; van Loon, Luc J. C.; Raymackers, Jean-Marc; Francaux, Marc; Deldicque, Louise	2013	Did not perform fiber type analysis
ACTN3 R577X genotypes associate withÂ Class II and deepbite malocclusions	Zebrick, Brian; Teeramongkolgul, Teesit; Nicot, Romain; Horton, Michael J.; Raoul, Gwenael; Ferri, Joel; Vieira, Alexandre R.; Sciote, James J.	2014	Does not include male and female data
Human slow troponin T (TNNT1) pre-mRNA alternative splicing is an indicator of skeletal muscle response to resistance exercise in older adults	Zhang, Tan; Choi, Seung Jun; Wang, Zhong-Min; Birbrair, Alexander; Messi, MarÃa L.; Jin, Jian-Ping; Marsh, Anthony P.; Nicklas, Barbara; Delbono, Osvaldo	2014	Does not include male and female data
Histological characterization and biochemical analysis of paraspinal muscles in neuromuscularly healthy subjects	Zimmermann, Claudia; Kalepu, Rajakiran; Ponfick, Matthias; Reichel, Heiko; Cakir, Balkan; Zierz, Stephan; Gdynia, Hans-JÃ¼rgen; Kassubek, Jan; Ludolph, Albert C.; Rosenbohm, Angela	2015	No healthy subjects or controls
Inflammatory response during slow- and fast-twitch muscle regeneration	Zimowska, Malgorzata; Kasprzycka, Paulina; Bocian, Katarzyna; Delaney, Kamila; Jung, Piotr; Kuchcinska, Kinga; Kaczmarska, Karolina; Gladysz, Daria; Streminska, Wladyslawa; Ciemerych, Maria Anna	2017	Animal study

Limb immobilization induces a coordinate down-regulation of mitochondrial and other metabolic pathways in men and women	Abadi, A.; Glover, E. I.; Isfort, R. J.; Raha, S.; Safdar, A.; Yasuda, N.; Kaczor, J. J.; Melov, S.; Hubbard, A.; Qu, X.; Phillips, S. M.; Tarnopolsky, M.	2009	Did not perform fiber type analysis
Single-leg cycle training is superior to double-leg cycling in improving the oxidative potential and metabolic profile of trained skeletal muscle	Abbiss, C. R.; Karagounis, L. G.; Laursen, P. B.; Peiffer, J. J.; Martin, D. T.; Hawley, J. A.; Fatehee, N. N.; Martin, J. C.	2011	Does not report sex of subjects
Skeletal muscle fibrosis is associated with decreased muscle inflammation and weakness in patients with chronic kidney disease	Abramowitz, M. K.; Paredes, W.; Zhang, K.; Brightwell, C. R.; Newsom, J. N.; Kwon, H. J.; Custodio, M.; Buttar, R. S.; Farooq, H.; Zaidi, B.; Pai, R.; Pessin, J. E.; Hawkins, M.; Fry, C. S.	2018	Did not perform fiber type analysis
Skeletal muscle myosin heavy chain composition and resistance training	Adams, G. R.; Hather, B. M.; Baldwin, K. M.; Dudley, G. A.	1993	Does not include males and females
Nuclear factor-kappa B activation in skeletal muscle of patients with chronic heart failure: correlation with the expression of inducible nitric oxide synthase	Adams, V.; SpÃ¤te, U.; KrÃ¤nkel, N.; Schulze, P. C.; Linke, A.; Schuler, G.; Hambrecht, R.	2003	Does not include males and females
Increased inducible nitric oxide synthase in skeletal muscle biopsies from patients with chronic heart failure	Adams, V.; Yu, J.; MÃ¶bius-Winkler, S.; Linke, A.; Weigl, C.; Hilbrich, L.; Schuler, G.; Hambrecht, R.	1997	Does not include males and females
Intramuscular fat and inflammation differ in older adults: the impact of frailty and inactivity	Addison, O.; Drummond, M. J.; LaStayo, P. C.; Dibble, L. E.; Wende, A. R.; McClain, D. A.; Marcus, R. L.	2014	Does not report sex of subjects Mira Atkinson
Increased Intramuscular Adipose Tissue Is Related to Increased Capillarization in Older Adults	Addison, O.; Ryan, A. S.; Blumenthal, J.; Prior, S. J.	2020	Did not perform fiber type analysis
Reduced synthesis of muscle proteins in chronic renal failure	Adey, D.; Kumar, R.; McCarthy, J. T.; Nair, K. S.	2000	Did not perform fiber type analysis
Immune status and apoptosis activation during brain death	Adrie, C.; Monchi, M.; Fulgencio, J. P.; Cottias, P.; Haouache, H.; Alvarez-Gonzalvez, A.; Guerrini, P.; Cavaillon, J. M.; Adib-Conquy, M.	2010	Did not perform fiber type analysis
Effects of inactivity on human muscle glutathione synthesis by a double-tracer and single-biopsy approach	Agostini, F.; Dalla Libera, L.; Rittweger, J.; Mazzucco, S.; Jurdana, M.; Mekjavic, I. B.; Pisot, R.; Gorza, L.; Narici, M.; Biolo, G.	2010	Does not include males and females
Post-exercise cold water immersion does not alter high intensity interval training-induced exercise performance and Hsp72 responses, but enhances mitochondrial markers	Aguiar, P. F.; MagalhÃ£es, S. M.; Fonseca, I. A.; da Costa Santos, V. B.; de Matos, M. A.; Peixoto, M. F.; Nakamura, F. Y.; Crandall, C.; AraÃºjo, H. N.; Silveira, L. R.; Rocha-Vieira, E.; de Castro MagalhÃ£es, F.; Amorim, F. T.	2016	Does not include males and females
Effect of HIF1A gene polymorphism on human muscle performance	Ahmetov,, II; Hakimullina, A. M.; Lyubaeva, E. V.; Vinogradova, O. L.; Rogozkin, V. A.	2008	Did not perform fiber type analysis
Taurine in normal and diseased human skeletal muscle	Airaksinen, E. M.; PaljÃ¤rvi, L.; Partanen, J.; Collan, Y.; Laakso, R.; PentikÃ¤inen, T.	1990	Does not include male and female data

Office based muscle biopsy using Vacora vacuum assisted biopsy system	Akarolo-Anthony, S. N.; Ogundiran, T. O.; Nkwodimmah, C.; Famooto, A.; Famooto, A. S.; Adediji, J.; Rotimi, C. N.; Balogun, W.; Adeleye, J.; Adebamowo, C. A.	2012	Did not perform fiber type analysis
Intact glucose transport in morphologically altered denervated skeletal muscle from quadriplegic patients	Aksnes, A. K.; Hjeltnes, N.; WahlstrÃ¶m, E. O.; Katz, A.; Zierath, J. R.; Wallberg-Henriksson, H.	1996	Does not include males and females
The Common miRNA Signatures Associated with Mitochondrial Dysfunction in Different Muscular Dystrophies	Aksu-Menges, E.; Akkaya-Ulum, Y. Z.; Dayangac-Erden, D.; Balci-Peynircioglu, B.; Yuzbasioglu, A.; Topaloglu, H.; Talim, B.; Balci-Hayta, B.	2020	No healthy subjects or controls
Human Body Composition and Immunity: Visceral Adipose Tissue Produces IL-15 and Muscle Strength Inversely Correlates with NK Cell Function in Elderly Humans	Al-Attar, A.; Presnell, S. R.; Clasey, J. L.; Long, D. E.; Walton, R. G.; Sexton, M.; Starr, M. E.; Kern, P. A.; Peterson, C. A.; Lutz, C. T.	2018	Does not include male and female data
Proteasome inhibition in skeletal muscle cells unmasks metabolic derangements in type 2 diabetes	Al-Khalili, L.; de Castro Barbosa, T.; Ostling, J.; Massart, J.; Cuesta, P. G.; Osler, M. E.; Katayama, M.; NystrÃ¶m, A. C.; Oscarsson, J.; Zierath, J. R.	2014	Does not include males and females
Mitochondrial abnormalities and low grade inflammation are present in the skeletal muscle of a minority of patients with amyotrophic lateral sclerosis; an observational myopathology study	Al-Sarraj, S.; King, A.; Cleveland, M.; Pradat, P. F.; Corse, A.; Rothstein, J. D.; Leigh, P. N.; Abila, B.; Bates, S.; Wurthner, J.; Meininger, V.	2014	Did not perform fiber type analysis
Integrated method for quantitative morphometry and oxygen transport modeling in striated muscle	Al-Shammari, A. A.; Kissane, R. W. P.; Holbek, S.; Mackey, A. L.; Andersen, T. R.; Gaffney, E. A.; Kjaer, M.; Egginton, S.	2019	Does not include males and females
Histochemical and ultrastructural characteristics of leg muscle fibres in patients with repairative abdominal aortic aneurysm (AAA)	Albani, M.; Kiskinis, D.; Natsis, K.; Megalopoulos, A.; Gigis, P.; Guiba-Tziampiri, O.	2000	Does not include males and females
Restrictions in ATP diffusion within sarcomeres can provoke ATP-depleted zones impairing exercise capacity in chronic obstructive pulmonary disease	Alekseev, A. E.; Guzun, R.; Reyes, S.; Pison, C.; Schlattner, U.; Selivanov, V. A.; Cascante, M.	2016	Does not include males and females
Improved exercise performance and increased aerobic capacity after endurance training of patients with stable polymyositis and dermatomyositis	Alemo Munters, L.; Dastmalchi, M.; Katz, A.; EsbjÃ¶rnsson, M.; Loell, I.; Hanna, B.; LidÃ©n, M.; Westerblad, H.; Lundberg, I. E.; Alexanderson, H.	2013	Did not perform fiber type analysis
Changes in neuromuscular performance and muscle fiber characteristics of elite power athletes self-administering androgenic and anabolic steroids	AlÃ©n, M.; HÃ¤kkinen, K.; Komi, P. V.	1984	Does not include males and females
Single cell analysis reveals the involvement of the long non-coding RNA Pvt1 in the modulation of muscle atrophy and mitochondrial network	Alessio, E.; Buson, L.; Chemello, F.; Peggion, C.; Grespi, F.; Martini, P.; Massimino, M. L.; Pacchioni, B.; Millino, C.; Romualdi, C.; Bertoli, A.; Scorrano, L.; Lanfranchi, G.; Cagnin, S.	2019	Animal study
Impact of Muscle Glycogen Availability on the Capacity for Repeated Exercise in Man	Alghannam, A. F.; Jedrzejewski, D.; Tweddle, M. G.; Gribble, H.; Bilzon, J.; Thompson, D.; Tsintzas, K.; Betts, J. A.	2016	Does not include males and females

Peripheral muscle endurance and the oxidative profile of the quadriceps in patients with COPD	Allaire, J.; Maltais, F.; Doyon, J. F.; NoÃ«l, M.; LeBlanc, P.; Carrier, G.; Simard, C.; Jobin, J.	2004	Does not include male and female data
Lipofuscin accumulation in the vastus lateralis muscle in patients with chronic obstructive pulmonary disease	Allaire, J.; Maltais, F.; LeBlanc, P.; Simard, P. M.; Whittom, F.; Doyon, J. F.; Simard, C.; Jobin, J.	2002	Does not include male and female data
Postexercise cold water immersion modulates skeletal muscle PGC-1Î± mRNA expression in immersed and nonimmersed limbs: evidence of systemic regulation	Allan, R.; Sharples, A. P.; Close, G. L.; Drust, B.; Shepherd, S. O.; Dutton, J.; Morton, J. P.; Gregson, W.	2017	Does not include males and females
Dynamic changes in the contractile apparatus during exercise	Allen, D. G.	2013	Review (ex: systematic review/meta-analysis)
Effects of Including Sprints in LIT Sessions during a 14-d Camp on Muscle Biology and Performance Measures in Elite Cyclists	Almquist, N. W.; Wilhelmsen, M.; Ellefsen, S.; Sandbakk, Ã˜; RÃ¸nnestad, B. R.	2021	Does not include males and females
Recruitment of single muscle fibers during submaximal cycling exercise	Altenburg, T. M.; Degens, H.; van Mechelen, W.; Sargeant, A. J.; de Haan, A.	2007	Does not include male and female data
Contrasts in muscle and myofibers of elite male and female bodybuilders	Alway, S. E.; Grumbt, W. H.; Gonyea, W. J.; Stray-Gundersen, J.	1989	Subjects used steroids
Mild mitochondrial uncoupling impacts cellular aging in human muscles in vivo	Amara, C. E.; Shankland, E. G.; Jubrias, S. A.; Marcinek, D. J.; Kushmerick, M. J.; Conley, K. E.	2007	Did not perform muscle biopsy
Gluteus medius muscle atrophy is related to contralateral and ipsilateral hip joint osteoarthritis	Amaro, A.; Amado, F.; Duarte, J. A.; Appell, H. J.	2007	No healthy subjects or controls
Calcium dysregulation, functional calpainopathy, and endoplasmic reticulum stress in sporadic inclusion body myositis	Amici, D. R.; Pinal-Fernandez, I.; MÃ¡zala, D. A.; Lloyd, T. E.; Corse, A. M.; Christopher-Stine, L.; Mammen, A. L.; Chin, E. R.	2017	Did not perform fiber type analysis
Rapid switch-off of the human myosin heavy chain IIX gene after heavy load muscle contractions is sustained for at least four days	Andersen, J. L.; Gruschy-Knudsen, T.	2018	Does not include males and females
Myosin heavy chain isoforms in single fibres from m. vastus lateralis of soccer players: effects of strength-training	Andersen, J. L.; Klitgaard, H.; Bangsbo, J.; Saltin, B.	1994	Does not include males and females
Mismatch between myosin heavy chain mRNA and protein distribution in human skeletal muscle fibers	Andersen, J. L.; Schiaffino, S.	1997	Does not include males and females
Changes in the human muscle force-velocity relationship in response to resistance training and subsequent detraining	Andersen, L. L.; Andersen, J. L.; Magnusson, S. P.; Suetta, C.; Madsen, J. L.; Christensen, L. R.; Aagaard, P.	2005	Does not include males and females
The effect of resistance training combined with timed ingestion of protein on muscle fiber size and muscle strength	Andersen, L. L.; Tufekovic, G.; Zebis, M. K.; Crameri, R. M.; Verlaan, G.; Kjaer, M.; Suetta, C.; Magnusson, P.; Aagaard, P.	2005	Does not include males and females
Capillary supply of the quadriceps femoris muscle of man: adaptive response to exercise	Andersen, P.; Henriksson, J.	1977	Does not include males and females
Expression of the major insulin regulatable glucose transporter (GLUT4) in skeletal muscle	Andersen, P. H.; Lund, S.; Vestergaard, H.; Junker, S.; Kahn, B. B.; Pedersen, O.	1993	Does not include male and female data

of noninsulin-dependent diabetic patients and healthy subjects before and after insulin infusion			
Type 2 diabetes is associated with altered NF-Î°B DNA binding activity, JNK phosphorylation, and AMPK phosphorylation in skeletal muscle after LPS	Andreasen, A. S.; Kelly, M.; Berg, R. M.; MÃ¸ller, K.; Pedersen, B. K.	2011	Does not include males and females
Decreased insulin-stimulated 3-0-methylglucose transport in in vitro incubated muscle strips from type II diabetic subjects	AndrÃ©asson, K.; Galuska, D.; ThÃ¶rne, A.; Sonnenfeld, T.; Wallberg-Henriksson, H.	1991	Did not perform fiber type analysis
Increased levels of the Akt-specific phosphatase PH domain leucine-rich repeat protein phosphatase (PHLPP)-1 in obese participants are associated with insulin resistance	Andreozzi, F.; Procopio, C.; Greco, A.; Mannino, G. C.; Miele, C.; Raciti, G. A.; Iadicicco, C.; Beguinot, F.; Pontiroli, A. E.; Hribal, M. L.; Folli, F.; Sesti, G.	2011	Did not perform fiber type analysis
Pro-inflammatory S100A11 is elevated in inflammatory myopathies and reflects disease activity and extramuscular manifestations in myositis	AndrÃ©s Cerezo, L.; HulejovÃ¡, H.; Å umovÃ¡, B.; KropÃ¡Ä�kovÃ¡, T.; KryÅ¡tÅ¯fkovÃ¡, O.; Klein, M.; Mann, H. F.; ZÃ¡meÄ�nÃk, J.; Pecha, O.; Pavelka, K.; VencovskÃ½, J.; Å enolt, L.	2019	Did not perform fiber type analysis
Mitochondrial function is impaired in the skeletal muscle of pre-frail elderly	Andreux, P. A.; van Diemen, M. P. J.; Heezen, M. R.; Auwerx, J.; Rinsch, C.; Groeneveld, G. J.; Singh, A.	2018	Did not perform fiber type analysis
Intermittent claudication and muscle fiber fine structure: morphometric data on mitochondrial volumes	Angquist, K. A.; SjÃ¶strÃ¶m, M.	1980	No healthy subjects or controls
Reference ranges for muscle carnitine concentration in children	Angsten, G.; Cederblad, G.; Meurling, S.	2003	Only in children (0-17 years)
Impaired muscle function with aging. A background factor in the incidence of fractures of the proximal end of the femur	Aniansson, A.; Zetterberg, C.; Hedberg, M.; Henriksson, K. G.	1984	No healthy subjects or controls
Increased frequency of activated satellite cells in overacting inferior oblique muscles from humans	Antunes-Foschini, R. M.; Ramalho, F. S.; Ramalho, L. N.; Bicas, H. E.	2006	Autopsies
Activated satellite cells in medial rectus muscles of patients with strabismus	Antunes-Foschini, R. S.; Miyashita, D.; Bicas, H. E.; McLoon, L. K.	2008	Autopsies
Myofiber HLA-DR expression is a distinctive biomarker for antisynthetase-associated myopathy	Aouizerate, J.; De Antonio, M.; Bassez, G.; Gherardi, R. K.; Berenbaum, F.; Guillevin, L.; Berezne, A.; Valeyre, D.; Maisonobe, T.; Dubourg, O.; Cosnes, A.; Benveniste, O.; Authier, F. J.	2014	No healthy subjects or controls
Satellite cell activation in human skeletal muscle after training: evidence for muscle fiber neoformation	Appell, H. J.; Forsberg, S.; Hollmann, W.	1988	Does not include males and females
Profile of creatine kinase isoenzymes in skeletal muscles of marathon runners	Apple, F. S.; Rogers, M. A.; Sherman, W. M.; Costill, D. L.; Hagerman, F. C.; Ivy, J. L.	1984	Does not include males and females
CK and LD isozymes in human single muscle fibers in trained athletes	Apple, F. S.; Tesch, P. A.	1989	Does not include males and females
Inflammatory response in facioscapulohumeral muscular dystrophy (FSHD): immunocytochemical and genetic analyses	Arahata, K.; Ishihara, T.; Fukunaga, H.; Orimo, S.; Lee, J. H.; Goto, K.; Nonaka, I.	1995	Does not include male and female data

Postviral fatigue syndrome: persistence of enterovirus RNA in muscle and elevated creatine kinase	Archard, L. C.; Bowles, N. E.; Behan, P. O.; Bell, E. J.; Doyle, D.	1988	Does not report sex of subjects
Immunohistological intensity measurements as a tool to assess sarcolemma-associated protein expression	Arechavala-Gomeza, V.; Kinali, M.; Feng, L.; Brown, S. C.; Sewry, C.; Morgan, J. E.; Muntoni, F.	2010	Only in children (0-17 years)
Abdominal subcutaneous adipose tissue insulin resistance and lipolysis in patients with non-alcoholic steatohepatitis	Armstrong, M. J.; Hazlehurst, J. M.; Hull, D.; Guo, K.; Borrows, S.; Yu, J.; Gough, S. C.; Newsome, P. N.; Tomlinson, J. W.	2014	Did not perform muscle biopsy
Inclusion body myositis--sensory dysfunction revealed with quantitative determination of somatosensory thresholds	Arnardottir, S.; Svanborg, E.; Borg, K.	2003	No healthy subjects or controls
Exercise stimulates c-Jun NH2 kinase activity and c-Jun transcriptional activity in human skeletal muscle	Aronson, D.; Boppart, M. D.; Dufresne, S. D.; Fielding, R. A.; Goodyear, L. J.	1998	Did not perform fiber type analysis
Exercise stimulates the mitogen-activated protein kinase pathway in human skeletal muscle	Aronson, D.; Violan, M. A.; Dufresne, S. D.; Zangen, D.; Fielding, R. A.; Goodyear, L. J.	1997	Did not perform fiber type analysis
Extracellular-regulated protein kinase cascades are activated in response to injury in human skeletal muscle	Aronson, D.; Wojtaszewski, J. F.; Thorell, A.; Nygren, J.; Zangen, D.; Richter, E. A.; Ljungqvist, O.; Fielding, R. A.; Goodyear, L. J.	1998	Did not perform fiber type analysis
The FibromiR miR-214-3p Is Upregulated in Duchenne Muscular Dystrophy and Promotes Differentiation of Human Fibro-Adipogenic Muscle Progenitors	Arrighi, N.; Moratal, C.; Savary, G.; Fassy, J.; Nottet, N.; Pons, N.; ClÃ©ment, N.; Fellah, S.; Larrue, R.; Magnone, V.; Lebrigand, K.; Pottier, N.; Dechesne, C.; Vassaux, G.; Dani, C.; Peraldi, P.; Mari, B.	2021	Did not perform fiber type analysis
Prevention of quadriceps wasting after immobilization: an evaluation of the effect of electrical stimulation	Arvidsson, I.; Arvidsson, H.; Eriksson, E.; Jansson, E.	1986	No healthy subjects or controls
Posterior cricoarytenoid bellies: relationship between their function and histology	Asanau, A.; Timoshenko, A. P.; Prades, J. M.; Galusca, B.; Martin, C.; FÃ©asson, L.	2011	No healthy subjects or controls
Distinct subtypes of type I fibers of human skeletal muscle	Askanas, V.; Engel, W. K.	1975	No healthy subjects or controls
Skeletal muscle phenotype is associated with exercise tolerance in patients with peripheral arterial disease	Askew, C. D.; Green, S.; Walker, P. J.; Kerr, G. K.; Green, A. A.; Williams, A. D.; Febbraio, M. A.	2005	Does not include male and female data
Creatine Monohydrate Supplementation, but not Creatyl-L-Leucine, Increased Muscle Creatine Content in Healthy Young Adults: A Double-Blind Randomized Controlled Trial	Askow, A. T.; Paulussen, K. J. M.; McKenna, C. F.; Salvador, A. F.; Scaroni, S. E.; Hamann, J. S.; Ulanov, A. V.; Li, Z.; Paluska, S. A.; Beaudry, K. M.; De Lisio, M.; Burd, N. A.	2022	Did not perform fiber type analysis
Muscle glycogen accumulation after a marathon: roles of fiber type and pro- and macroglycogen	Asp, S.; Daugaard, J. R.; Rohde, T.; Adamo, K.; Graham, T.	1999	Does not include males and females
Impaired muscle glycogen resynthesis after a marathon is not caused by decreased muscle GLUT-4 content	Asp, S.; Rohde, T.; Richter, E. A.	1997	Does not include males and females
Effects of viral and Mycoplasma infections on the ultrastructure of human skeletal muscle	AstrÃ¶m, E.; Friman, G.; PilstrÃ¶m, L.	1975	Does not include males and females

Interspersed normoxia during live high, train low interventions reverses an early reduction in muscle Na+, K +ATPase activity in well-trained athletes	Aughey, R. J.; Clark, S. A.; Gore, C. J.; Townsend, N. E.; Hahn, A. G.; Kinsman, T. A.; Goodman, C.; Chow, C. M.; Martin, D. T.; Hawley, J. A.; McKenna, M. J.	2006	Does not include males and females
Chronic intermittent hypoxia and incremental cycling exercise independently depress muscle in vitro maximal Na+-K+-ATPase activity in well-trained athletes	Aughey, R. J.; Gore, C. J.; Hahn, A. G.; Garnham, A. P.; Clark, S. A.; Petersen, A. C.; Roberts, A. D.; McKenna, M. J.	2005	Does not include males and females
Muscle Na+-K+-ATPase activity and isoform adaptations to intense interval exercise and training in well-trained athletes	Aughey, R. J.; Murphy, K. T.; Clark, S. A.; Garnham, A. P.; Snow, R. J.; Cameron-Smith, D.; Hawley, J. A.; McKenna, M. J.	2007	Does not include males and females
The relation between cycling time to exhaustion and anaerobic threshold	Aunola, S.; Alanen, E.; Marniemi, J.; Rusko, H.	1990	Does not include males and females
Interleukin-1 expression in normal motor endplates and muscle fibers showing neurogenic changes	Authier, F. J.; Chazaud, B.; Mhiri, C.; Eliezer-Vanerot, M. C.; Poron, F.; Barlovatz-Meimon, G.; Gherardi, R. K.	1997	Does not include males and females
Changes in myostatin signaling in non-weight-losing cancer patients	Aversa, Z.; Bonetto, A.; Penna, F.; Costelli, P.; Di Rienzo, G.; Lacitignola, A.; Baccino, F. M.; Ziparo, V.; Mercantini, P.; Rossi Fanelli, F.; Muscaritoli, M.	2012	No healthy subjects or controls
Lipids activate skeletal muscle mitochondrial fission and quality control networks to induce insulin resistance in humans	Axelrod, C. L.; Fealy, C. E.; Erickson, M. L.; Davuluri, G.; Fujioka, H.; Dantas, W. S.; Huang, E.; Pergola, K.; Mey, J. T.; King, W. T.; Mulya, A.; Hsia, D.; Burguera, B.; Tandler, B.; Hoppel, C. L.; Kirwan, J. P.	2021	Did not perform fiber type analysis
Exercise training remodels human skeletal muscle mitochondrial fission and fusion machinery towards a pro-elongation phenotype	Axelrod, C. L.; Fealy, C. E.; Mulya, A.; Kirwan, J. P.	2019	No healthy subjects or controls
Altered skeletal muscle lipase expression and activity contribute to insulin resistance in humans	Badin, P. M.; Louche, K.; Mairal, A.; Liebisch, G.; Schmitz, G.; Rustan, A. C.; Smith, S. R.; Langin, D.; Moro, C.	2011	Did not perform fiber type analysis
Calpain 3 deficiency is associated with myonuclear apoptosis and profound perturbation of the IkappaB alpha/NF-kappaB pathway in limb-girdle muscular dystrophy type 2A	Baghdiguian, S.; Martin, M.; Richard, I.; Pons, F.; Astier, C.; Bourg, N.; Hay, R. T.; Chemaly, R.; Halaby, G.; Loiselet, J.; Anderson, L. V.; Lopez de Munain, A.; Fardeau, M.; Mangeat, P.; Beckmann, J. S.; Lefranc, G.	1999	Did not perform fiber type analysis
A comparison of vertebral muscle fiber characteristics between human and monkey tissue	Bagnall, K. M.; Ford, D. M.; McFadden, K. D.; Greenhill, B. J.; Raso, V. J.	1983	No healthy subjects or controls
Effects of sprint training combined with vegetarian or mixed diet on muscle carnosine content and buffering capacity	Baguet, A.; Everaert, I.; De Naeyer, H.; Reyngoudt, H.; Stegen, S.; Beeckman, S.; Achten, E.; Vanhee, L.; Volkaert, A.; Petrovic, M.; Taes, Y.; Derave, W.	2011	Did not perform fiber type analysis
Anomalies in perifascicular muscle fibers as an differential-diagnostic criterion. II. Perifascicular hypertrophies in primary myopathies	BÃ¤hr, M.; Peiffer, J.	1987	No healthy subjects or controls
MAFbx, MuRF1, and the stress-activated protein kinases are upregulated in muscle cells during total knee arthroplasty	Bailey, A. N.; Hocker, A. D.; Vermillion, B. R.; Smolkowski, K.; Shah, S. N.; Jewett, B. A.; Dreyer, H. C.	2012	No healthy subjects or controls

Multiplex immunoassay analysis of cytokines in idiopathic inflammatory myopathy	Baird, G. S.; Montine, T. J.	2008	Did not perform fiber type analysis
Insulin receptor function and glycogen synthase activity in skeletal muscle biopsies from patients with insulin-dependent diabetes mellitus: effects of physical training	Bak, J. F.; Jacobsen, U. K.; JÃ¸rgensen, F. S.; Pedersen, O.	1989	Did not perform fiber type analysis
Effects of growth hormone on fuel utilization and muscle glycogen synthase activity in normal humans	Bak, J. F.; MÃ¸ller, N.; Schmitz, O.	1991	Does not include males and females
Exercise-enhanced activation of glycogen synthase in human skeletal muscle	Bak, J. F.; Pedersen, O.	1990	Did not perform fiber type analysis
Activity of insulin receptor kinase and glycogen synthase in skeletal muscle from patients with chronic renal failure	Bak, J. F.; Schmitz, O.; SÃ¸rensen, S. S.; FrÃ¸kjaer, J.; Kjaer, T.; Pedersen, O.	1989	No healthy subjects or controls
Effects of high intensity canoeing training on fibre area and fibre type in the latissimus dorsi muscle	Baker, S. J.; Hardy, L.	1989	Does not include males and females
Quantitative and qualitative adaptation of human skeletal muscle mitochondria to hypoxic compared with normoxic training at the same relative work rate	Bakkman, L.; Sahlin, K.; Holmberg, H. C.; Tonkonogi, M.	2007	Did not perform fiber type analysis
Skeletal muscle myosin heavy-chain synthesis rate in healthy humans	Balagopal, P.; Ljungqvist, O.; Nair, K. S.	1997	Does not include male and female data
Isolation of myosin heavy chain from small skeletal muscle samples by preparative continuous elution gel electrophoresis: application to measurement of synthesis rate in human and animal tissue	Balagopal, P.; Nair, K. S.; Stirewalt, W. S.	1994	Does not include male and female data
Inter-tissue expression patterns of the key metabolic biomarker PGC-1Î± in severely obese individuals: Implication in obesity-induced disease	Balampanis, K.; Chasapi, A.; Kourea, E.; Tanoglidi, A.; Hatziagelaki, E.; Lambadiari, V.; Dimitriadis, G.; Lambrou, G. I.; Kalfarentzos, F.; Melachrinou, M.; Sotiropoulou-Bonikou, G.	2019	Did not perform fiber type analysis
Effects of weight loss and leptin on skeletal muscle in human subjects	Baldwin, K. M.; Joanisse, D. R.; Haddad, F.; Goldsmith, R. L.; Gallagher, D.; Pavlovich, K. H.; Shamoon, E. L.; Leibel, R. L.; Rosenbaum, M.	2011	Does not include male and female data
Evaluation of a New Skeletal Troponin I Assay in Patients with Idiopathic Inflammatory Myopathies	Bamberg, K.; MehtÃ¤lÃ¤, L.; Arola, O.; Laitinen, S.; Nordling, P.; Strandberg, M.; Strandberg, N.; Paltta, J.; Mali, M.; Espinosa-Ortega, F.; PirilÃ¤, L.; Lundberg, I. E.; Savukoski, T.; Pettersson, K.	2020	Did not perform fiber type analysis
Skeletal muscle regeneration in facioscapulohumeral muscular dystrophy is correlated with pathological severity	Banerji, C. R. S.; Henderson, D.; Tawil, R. N.; Zammit, P. S.	2020	No healthy subjects or controls
DUX4 expressing immortalized FSHD lymphoblastoid cells express genes elevated in FSHD muscle biopsies, correlating with the early stages of inflammation	Banerji, C. R. S.; Panamarova, M.; Zammit, P. S.	2020	Did not perform fiber type analysis

Enhanced pyruvate dehydrogenase activity does not affect muscle O2 uptake at onset of intense exercise in humans	Bangsbo, J.; Gibala, M. J.; Krustrup, P.; GonzÃ¡lez-Alonso, J.; Saltin, B.	2002	Does not include males and females
Effect of muscle acidity on muscle metabolism and fatigue during intense exercise in man	Bangsbo, J.; Madsen, K.; Kiens, B.; Richter, E. A.	1996	Does not include males and females
Accumulated O2 deficit during intense exercise and muscle characteristics of elite athletes	Bangsbo, J.; Michalsik, L.; Petersen, A.	1993	Does not include males and females
Ingestion of lean meat elevates muscle inositol hexakisphosphate kinase 1 protein content independent of a distinct post-prandial circulating proteome in young adults with obesity	Barclay, R. D.; Beals, J. W.; Drnevich, J.; Imai, B. S.; Yau, P. M.; Ulanov, A. V.; Tillin, N. A.; Villegas-Montes, M.; Paluska, S. A.; Watt, P. W.; De Lisio, M.; Burd, N. A.; Mackenzie, R. W.	2020	Did not perform fiber type analysis
The ultrastructure of normal and denervated human facial muscle	Bardosi, A.; Goebel, H.; Stennert, E.	1987	Did not perform fiber type analysis
Walking performance, oxygen uptake kinetics and resting muscle pyruvate dehydrogenase complex activity in peripheral arterial disease	Barker, G. A.; Green, S.; Green, A. A.; Walker, P. J.	2004	Did not perform fiber type analysis
Immune response and mitochondrial metabolism are commonly deregulated in DMD and aging skeletal muscle	Baron, D.; Magot, A.; Ramstein, G.; Steenman, M.; Fayet, G.; Chevalier, C.; Jourdon, P.; Houlgatte, R.; Savagner, F.; Pereon, Y.	2011	Did not perform fiber type analysis
Inflammatory cells and apoptosis in respiratory and limb muscles of patients with COPD	Barreiro, E.; Ferrer, D.; Sanchez, F.; Minguella, J.; Marin-Corral, J.; Martinez-Llorens, J.; Lloreta, J.; Gea, J.	2011	Does not include males and females
The phosphodiesterase-4 inhibitor roflumilast reverts proteolysis in skeletal muscle cells of patients with COPD cachexia	Barreiro, E.; Puig-Vilanova, E.; Salazar-Degracia, A.; Pascual-Guardia, S.; Casadevall, C.; Gea, J.	2018	Does not report sex of subjects
Endoplasmic reticulum stress and unfolded protein response profile in quadriceps of sarcopenic patients with respiratory diseases	Barreiro, E.; Salazar-Degracia, A.; Sancho-MuÃ±oz, A.; Gea, J.	2019	No cross-sectional area data
Differences in micro-RNA expression profile between vastus lateralis samples and myotubes in COPD cachexia	Barreiro, E.; Sancho-MuÃ±oz, A.; Puig-Vilanova, E.; Salazar-Degracia, A.; Pascual-Guardia, S.; Casadevall, C.; Gea, J.	2019	Did not perform fiber type analysis
Cytokine profile in quadriceps muscles of patients with severe COPD	Barreiro, E.; Schols, A. M.; Polkey, M. I.; Galdiz, J. B.; Gosker, H. R.; Swallow, E. B.; Coronell, C.; Gea, J.	2008	Does not include males and females
Influence of muscle fiber type and pedal frequency on oxygen uptake kinetics of heavy exercise	Barstow, T. J.; Jones, A. M.; Nguyen, P. H.; Casaburi, R.	1996	Does not include males and females
Influence of muscle fibre type and fitness on the oxygen uptake/power output slope during incremental exercise in humans	Barstow, T. J.; Jones, A. M.; Nguyen, P. H.; Casaburi, R.	2000	Does not include males and females
CCR2A and CCR2B, the two isoforms of the monocyte chemoattractant protein-1 receptor are up-regulated and expressed by different cell subsets in idiopathic inflammatory myopathies	Bartoli, C.; Civatte, M.; Pellissier, J. F.; Figarella-Branger, D.	2001	No healthy subjects or controls
Human temporomandibular joint and myofascial pain biochemical profiles: a case-control study	Basi, D. L.; Velly, A. M.; Schiffman, E. L.; Lenton, P. A.; Besspiata, D. A.; Rankin, A. M.; Hughes, P. J.; Swift, J. Q.; Kehl, L. J.	2012	Did not perform fiber type analysis

Idiopathic inflammatory myopathy human derived cells retain their ability to increase mitochondrial function	Basualto-AlarcÃ³n, C.; Urra, F. A.; BozÃ¡n, M. F.; JaÃ±a, F.; Trangulao, A.; Bevilacqua, J. A.; CÃ¡rdenas, J. C.	2020	No healthy subjects or controls
Skeletal muscle dysfunction in idiopathic pulmonary arterial hypertension	Batt, J.; Ahmed, S. S.; Correa, J.; Bain, A.; Granton, J.	2014	Did not perform fiber type analysis
Quadriceps atrophy in the anterior cruciate insufficient knee	Baugher, W. H.; Warren, R. F.; Marshall, J. L.; Joseph, A.	1984	Does not include males and females
Arteriolization of capillaries and FGF-2 upregulation in skeletal muscles of patients with chronic peripheral arterial disease	Baum, O.; Djonov, V.; Ganster, M.; Widmer, M.; Baumgartner, I.	2005	Did not perform fiber type analysis
Angiogenesis-related ultrastructural changes to capillaries in human skeletal muscle in response to endurance exercise	Baum, O.; GÃ¼beli, J.; Frese, S.; Torchetti, E.; Malik, C.; Odriozola, A.; Graber, F.; Hoppeler, H.; Tschanz, S. A.	2015	Does not include males and females
Capillary ultrastructure and mitochondrial volume density in skeletal muscle in relation to reduced exercise capacity of patients with intermittent claudication	Baum, O.; Torchetti, E.; Malik, C.; Hoier, B.; Walker, M.; Walker, P. J.; Odriozola, A.; Graber, F.; Tschanz, S. A.; Bangsbo, J.; Hoppeler, H.; Askew, C. D.; Hellsten, Y.	2016	Does not include male and female data
Oxidative capacities of cardiac and skeletal muscles of heart transplant recipients: mitochondrial effects of cyclosporin-A and its vehicle Cremophor-EL	N' Guessan BB; Sanchez, H.; Zoll, J.; Ribera, F.; Dufour, S.; Lampert, E.; Kindo, M.; Geny, B.; Ventura-Clapier, R.; Mettauer, B.	2014	Does not include males and females
Variability in estimating eccentric contraction-induced muscle damage and inflammation in humans	Beaton, L. J.; Tarnopolsky, M. A.; Phillips, S. M.	2002	Does not include male and female data
Comparison of clinical performance, histology and single-fiber contractility in free neurovascular muscle flaps	Becker, M. H.; Wermter, T. B.; Brenner, B.; Walter, G. F.; Berger, A.	2000	Does not include male and female data
Optical probing of gastrocnemius in patients with peripheral artery disease characterizes myopathic biochemical alterations and correlates with stage of disease	Becker, R. A.; Cluff, K.; Duraisamy, N.; Mehraein, H.; Farhoud, H.; Collins, T.; Casale, G. P.; Pipinos,, II; Subbiah, J.	2017	Does not include males and females
Skeletal muscle adaptation in response to supervised exercise training for intermittent claudication	Beckitt, T. A.; Day, J.; Morgan, M.; Lamont, P. M.	2012	No healthy subjects or controls
Use of capillary Western immunoassay (Wes) for quantification of dystrophin levels in skeletal muscle of healthy controls and individuals with Becker and Duchenne muscular dystrophy	Beekman, C.; Janson, A. A.; Baghat, A.; van Deutekom, J. C.; Datson, N. A.	2018	Does not report sex of subjects
Maximum rate of oxygen consumption related to succinate dehydrogenase activity in skeletal muscle fibres of chronic heart failure patients and controls	Bekedam, M. A.; van Beek-Harmsen, B. J.; Boonstra, A.; van Mechelen, W.; Visser, F. C.; van der Laarse, W. J.	2003	Does not include male and female data
Myoglobin concentration in skeletal muscle fibers of chronic heart failure patients	Bekedam, M. A.; van Beek-Harmsen, B. J.; van Mechelen, W.; Boonstra, A.; van der Laarse, W. J.	2009	Does not include male and female data
Skeletal Muscle Function, Structure, and Metabolism in Patients With Heart Failure With Reduced Ejection Fraction and Heart Failure With Preserved Ejection Fraction	Bekfani, T.; Bekhite Elsaied, M.; Derlien, S.; Nisser, J.; Westermann, M.; Nietzsche, S.; Hamadanchi, A.; FrÃ¶b, E.; Westphal, J.; Haase, D.; Kretzschmar, T.; Schlattmann, P.; Smolenski, U. C.; Lichtenauer, M.; Wernly,	2020	Does not include male and female data

	B.; Jirak, P.; Lehmann, G.; MÃ¶bius-Winkler, S.; Schulze, P. C.		
Determinants of oxygen uptake kinetics in older humans following single-limb endurance exercise training	Bell, C.; Paterson, D. H.; Kowalchuk, J. M.; Moy, A. P.; Thorp, D. B.; Noble, E. G.; Taylor, A. W.; Cunningham, D. A.	2001	Does not include males and females
Effect of high velocity resistance training on peak torque, cross sectional area and myofibrillar ATPase activity	Bell, G. J.; Petersen, S. R.; MacLean, I.; Reid, D. C.; Quinney, H. A.	1992	Does not include males and females
Dysregulation of muscle fatty acid metabolism in type 2 diabetes is independent of malonyl-CoA	Bell, J. A.; Volpi, E.; Fujita, S.; Cadenas, J. G.; Rasmussen, B. B.	2006	Did not perform fiber type analysis
Skeletal muscle protein anabolic response to increased energy and insulin is preserved in poorly controlled type 2 diabetes	Bell, J. A.; Volpi, E.; Fujita, S.; Cadenas, J. G.; Sheffield-Moore, M.; Rasmussen, B. B.	2006	Did not perform fiber type analysis
Muscle fiber types and morphometric analysis of skeletal msucle in six-year-old children	Bell, R. D.; MacDougall, J. D.; Billeter, R.; Howald, H.	1980	Only in children (0-17 years)
Oral creatine supplementation decreases plasma markers of adenine nucleotide degradation during a 1-h cycle test	Bellinger, B. M.; Bold, A.; Wilson, G. R.; Noakes, T. D.; Myburgh, K. H.	2000	Does not include males and females
Effects of long-duration bed rest on structural compartments of m. soleus in man	Belozerova, I.; Shenkman, B.; Mazin, M.; Leblanc, A.	2001	Does not include males and females
Metabolically assessed muscle fibre recruitment in brief isometric contractions at different intensities	Beltman, J. G.; de Haan, A.; Haan, H.; Gerrits, H. L.; van Mechelen, W.; Sargeant, A. J.	2004	Does not include male and female data
Changes in PCr/Cr ratio in single characterized muscle fibre fragments after only a few maximal voluntary contractions in humans	Beltman, J. G.; Sargeant, A. J.; Haan, H.; van Mechelen, W.; de Haan, A.	2004	Does not include male and female data
Voluntary activation level and muscle fiber recruitment of human quadriceps during lengthening contractions	Beltman, J. G.; Sargeant, A. J.; van Mechelen, W.; de Haan, A.	2004	No cross-sectional area data
Deficiency of Na+/K(+)-ATPase and sarcoplasmic reticulum Ca(2+)-ATPase in skeletal muscle and cultured muscle cells of myotonic dystrophy patients	Benders, A. A.; Timmermans, J. A.; Oosterhof, A.; Ter Laak, H. J.; van Kuppevelt, T. H.; Wevers, R. A.; Veerkamp, J. H.	1993	Does not report sex of subjects
Short Leukocyte Telomere Length Precedes Clinical Expression of Atherosclerosis: The Blood-and-Muscle Model	Benetos, A.; Toupance, S.; Gautier, S.; Labat, C.; Kimura, M.; Rossi, P. M.; Settembre, N.; Hubert, J.; Frimat, L.; Bertrand, B.; Boufi, M.; Flecher, X.; Sadoul, N.; Eschwege, P.; Kessler, M.; Tzanetakou, I. P.; Doulamis, I. P.; Konstantopoulos, P.; Tzani, A.; Korou, M.; Gkogkos, A.; Perreas, K.; Menenakos, E.; Samanidis, G.; Vasiloglou-Gkanis, M.; Kark, J. D.; Malikov, S.; Verhulst, S.; Aviv, A.	2018	Did not perform fiber type analysis
Nuclear speckles are involved in nuclear aggregation of PABPN1 and in the pathophysiology of oculopharyngeal muscular dystrophy	Bengoechea, R.; Tapia, O.; Casafont, I.; Berciano, J.; Lafarga, M.; Berciano, M. T.	2012	Does not report sex of subjects
Muscle biopsy in primary fibromyalgia. Light-microscopical and histochemical findings	Bengtsson, A.; Henriksson, K. G.; Larsson, J.	1986	Does not report sex of subjects

Maximal strength training increases muscle force generating capacity and the anaerobic ATP synthesis flux without altering the cost of contraction in elderly	Berg, O. K.; Kwon, O. S.; Hureau, T. J.; Clifton, H. L.; Thurston, T.; Le Fur, Y.; Jeong, E. K.; Amann, M.; Richardson, R. S.; Trinity, J. D.; Wang, E.; Layec, G.	2018	Does not include male and female data
Localisation and composition of skeletal muscle diacylglycerol predicts insulin resistance in humans	Bergman, B. C.; Hunerdosse, D. M.; Kerege, A.; Playdon, M. C.; Perreault, L.	2012	No cross-sectional area data
Increasing dietary fat elicits similar changes in fat oxidation and markers of muscle oxidative capacity in lean and obese humans	Bergouignan, A.; Gozansky, W. S.; Barry, D. W.; Leitner, W.; MacLean, P. S.; Hill, J. O.; Draznin, B.; Melanson, E. L.	2012	Did not perform fiber type analysis
Energy cost and fatigue during intermittent electrical stimulation of human skeletal muscle	BergstrÃ¶m, M.; Hultman, E.	1988	Did not perform fiber type analysis
Functional and Structural Adaptations of Skeletal Muscle in Long-Term Juvenile Dermatomyositis: A Controlled Cross-Sectional Study	Berntsen, K. S.; Raastad, T.; Marstein, H.; Kirkhus, E.; Merckoll, E.; Cumming, K. T.; FlatÃ¸, B.; Sjaastad, I.; Sanner, H.	2020	Does not include male and female data
Increased collagen content in insulin-resistant skeletal muscle	Berria, R.; Wang, L.; Richardson, D. K.; Finlayson, J.; Belfort, R.; Pratipanawatr, T.; De Filippis, E. A.; Kashyap, S.; Mandarino, L. J.	2006	Did not perform fiber type analysis
Four days of simulated shift work reduces insulin sensitivity in humans	Bescos, R.; Boden, M. J.; Jackson, M. L.; Trewin, A. J.; Marin, E. C.; Levinger, I.; Garnham, A.; Hiam, D. S.; Falcao-Tebas, F.; Conte, F.; Owens, J. A.; Kennaway, D. J.; McConell, G. K.	2018	Did not perform fiber type analysis
Muscle activity and muscle agrin regulate the organization of cytoskeletal proteins and attached acetylcholine receptor (AchR) aggregates in skeletal muscle fibers	Bezakova, G.; LÃ¸mo, T.	2001	Animal study
Effects of 10-ppm hydrogen sulfide inhalation in exercising men and women. Cardiovascular, metabolic, and biochemical responses	Bhambhani, Y.; Burnham, R.; Snydmiller, G.; MacLean, I.	1997	Did not perform fiber type analysis
Skeletal muscle mitochondrial DNA injury in patients with unilateral peripheral arterial disease	Bhat, H. K.; Hiatt, W. R.; Hoppel, C. L.; Brass, E. P.	1999	Does not include males and females
Time course of molecular responses of human skeletal muscle to acute bouts of resistance exercise	Bickel, C. S.; Slade, J.; Mahoney, E.; Haddad, F.; Dudley, G. A.; Adams, G. R.	2005	Did not perform fiber type analysis
Acute molecular responses of skeletal muscle to resistance exercise in able-bodied and spinal cord-injured subjects	Bickel, C. S.; Slade, J. M.; Haddad, F.; Adams, G. R.; Dudley, G. A.	2003	Did not perform fiber type analysis
Morphometry of skeletal muscle capillaries: the relationship between capillary ultrastructure and ageing in humans	Bigler, M.; Koutsantonis, D.; Odriozola, A.; Halm, S.; Tschanz, S. A.; Zakrzewicz, A.; Weichert, A.; Baum, O.	2016	Did not perform fiber type analysis
Myosin types in human skeletal muscle fibers	Billeter, R.; Weber, H.; Lutz, H.; Howald, H.; Eppenberger, H. M.; Jenny, E.	1980	Does not include males and females
Physiologic hyperinsulinemia stimulates protein synthesis and enhances transport of selected amino acids in human skeletal muscle	Biolo, G.; Declan Fleming, R. Y.; Wolfe, R. R.	1995	Does not include males and females

Inhibition of muscle glutamine formation in hypercatabolic patients	Biolo, G.; Fleming, R. Y.; Maggi, S. P.; Nguyen, T. T.; Herndon, D. N.; Wolfe, R. R.	2000	Did not perform fiber type analysis
Inverse regulation of protein turnover and amino acid transport in skeletal muscle of hypercatabolic patients	Biolo, G.; Fleming, R. Y.; Maggi, S. P.; Nguyen, T. T.; Herndon, D. N.; Wolfe, R. R.	2002	Did not perform fiber type analysis
Transmembrane transport and intracellular kinetics of amino acids in human skeletal muscle	Biolo, G.; Fleming, R. Y.; Maggi, S. P.; Wolfe, R. R.	1995	Does not include males and females
Insulin action on muscle protein kinetics and amino acid transport during recovery after resistance exercise	Biolo, G.; Williams, B. D.; Fleming, R. Y.; Wolfe, R. R.	1999	Does not include males and females
Effects of Training Status and Exercise Mode on Global Gene Expression in Skeletal Muscle	Bizjak, D. A.; ZÃ¼gel, M.; Treff, G.; Winkert, K.; Jerg, A.; Hudemann, J.; Mooren, F. C.; KrÃ¼ger, K.; NieÃŸ, A.; Steinacker, J. M.	2021	Does not include males and females
Determination of succinic oxidase activity in human skeletal muscle	BjÃ¶rntorp, P.; FahlÃ©n, M.; Holm, J.; ScherstÃ©n, T.; Szostak, V.	1970	Did not perform fiber type analysis
Respiration and phosphorylation of mitochondria isolated from the skeletal muscle of diabetic and normal subjects	BjÃ¶rntorp, P.; ScherstÃ©n, T.; Fagerberg, S. E.	1967	Did not perform fiber type analysis
Group III/IV muscle afferents limit the intramuscular metabolic perturbation during whole body exercise in humans	Blain, G. M.; Mangum, T. S.; Sidhu, S. K.; Weavil, J. C.; Hureau, T. J.; Jessop, J. E.; Bledsoe, A. D.; Richardson, R. S.; Amann, M.	2016	Does not include males and females
Effects of limb immobilization on cytochrome c oxidase activity and GLUT4 and GLUT5 protein expression in human skeletal muscle	Blakemore, S. J.; Rickhuss, P. K.; Watt, P. W.; Rennie, M. J.; Hundal, H. S.	1996	Does not include males and females
COPD is deleterious for pericytes: implications during training-induced angiogenesis in skeletal muscle	Blervaque, L.; PomiÃ¨s, P.; Rossi, E.; Catteau, M.; BlandiniÃ¨res, A.; Passerieux, E.; BlaquiÃ¨re, M.; Ayoub, B.; Molinari, N.; Mercier, J.; Perez-Martin, A.; Marchi, N.; Smadja, D. M.; Hayot, M.; Gouzi, F.	2020	Did not perform fiber type analysis
Rapid determination of myosin heavy chain expression in rat, mouse, and human skeletal muscle using multicolor immunofluorescence analysis	Bloemberg, D.; Quadrilatero, J.	2012	Does not include males and females
How to calculate human muscle fibre areas in biopsy samples--methodological considerations	Blomstrand, E.; Celsing, F.; FridÃ©n, J.; Ekblom, B.	1984	Does not include males and females
Exercise training induces similar elevations in the activity of oxoglutarate dehydrogenase and peak oxygen uptake in the human quadriceps muscle	Blomstrand, E.; Krustrup, P.; SÃ¸ndergaard, H.; RÃ¥degran, G.; Calbet, J. A.; Saltin, B.	2011	Does not include males and females
Maximum rate of oxygen uptake by human skeletal muscle in relation to maximal activities of enzymes in the Krebs cycle	Blomstrand, E.; RÃ¥degran, G.; Saltin, B.	1997	Did not perform fiber type analysis
Polymyositis with cytochrome oxidase negative muscle fibres. Early quadriceps weakness and poor response to immunosuppressive therapy	Blume, G.; Pestronk, A.; Frank, B.; Johns, D. R.	1997	Did not perform fiber type analysis
Neuromuscular junctions are stable in patients with cancer cachexia	Boehm, I.; Miller, J.; Wishart, T. M.; Wigmore, S. J.; Skipworth, R. J.; Jones, R. A.; Gillingwater, T. H.	2020	No healthy subjects or controls

Skeletal Muscle Regeneration in Advanced Diabetic Peripheral Neuropathy	Bohnert, K. L.; Hastings, M. K.; Sinacore, D. R.; Johnson, J. E.; Klein, S. E.; McCormick, J. J.; Gontarz, P.; Meyer, G. A.	2020	No healthy subjects or controls
Skeletal Muscle Gene Expression in Long-Term Endurance and Resistance Trained Elderly	Bolotta, A.; Filardo, G.; Abruzzo, P. M.; Astolfi, A.; De Sanctis, P.; Di Martino, A.; Hofer, C.; Indio, V.; Kern, H.; LÃ¶fler, S.; Marcacci, M.; Zampieri, S.; Marini, M.; Zucchini, C.	2020	Does not include males and females
Gene expression and fiber type variations in repeated vastus lateralis biopsies	Boman, N.; BurÃ©n, J.; Antti, H.; Svensson, M. B.	2015	Does not include males and females
Gene expression variability in human skeletal muscle transcriptome responses to acute resistance exercise	Bonafiglia, J. T.; Menzies, K. J.; Gurd, B. J.	2019	Did not perform fiber type analysis
Preparation of isolated human muscle fibers: a technical report	Bonavaud, S.; Agbulut, O.; D'Honneur, G.; Nizard, R.; Mouly, V.; Butler-Browne, G.	2002	No healthy subjects or controls
Effect of training with eccentric muscle contractions on skeletal muscle metabolites	Bonde-Petersen, F.; Henriksson, J.; Knuttgen, H. G.	1973	Does not include males and females
Short-term training increases human muscle MCT1 and femoral venous lactate in relation to muscle lactate	Bonen, A.; McCullagh, K. J.; Putman, C. T.; Hultman, E.; Jones, N. L.; Heigenhauser, G. J.	1998	Does not include males and females
Duchenne dystrophy: focal alterations in the distribution of concanavalin A binding sites at the muscle cell surface	Bonilla, E.; Schotland, D. L.; Wakayama, Y.	1978	Did not perform fiber type analysis
Muscle fibre type composition, motoneuron firing properties, axonal conduction velocity and refractory period for foot extensor motor units in dystrophia myotonica	Borg, J.; EdstrÃ¶m, L.; Butler-Browne, G. S.; Thornell, L. E.	1987	No healthy subjects or controls
Prior poliomyelitis-reduced capillary supply and metabolic enzyme content in hypertrophic slow-twitch (type I) muscle fibres	Borg, K.; Henriksson, J.	1991	Does not include male and female data
Neurogenic involvement in distal myopathy (Welander). Histochemical and morphological observations on muscle and nerve biopsies	Borg, K.; Solders, G.; Borg, J.; EdstrÃ¶m, L.; Kristensson, K.	1989	Does not include male and female data
Intake of branched-chain amino acids influences the levels of MAFbx mRNA and MuRF-1 total protein in resting and exercising human muscle	Borgenvik, M.; AprÃ³, W.; Blomstrand, E.	2012	Did not perform fiber type analysis
Enzyme activities in type I and II muscle fibres of human skeletal muscle in relation to age and torque development	Borges, O.; EssÃ©n-Gustavsson, B.	1989	Same subjects as another study
GLUT-4 expression is not consistently higher in type-1 than in type-2 fibres of rat and human vastus lateralis muscles; an immunohistochemical study	Borghouts, L. B.; Schaart, G.; Hesselink, M. K.; Keizer, H. A.	2000	Does not include males and females
The effects of aging, physical training, and a single bout of exercise on mitochondrial protein expression in human skeletal muscle	Bori, Z.; Zhao, Z.; Koltai, E.; Fatouros, I. G.; Jamurtas, A. Z.; Douroudos,, II; Terzis, G.; Chatzinikolaou, A.; Sovatzidis, A.; Draganidis, D.; Boldogh, I.; Radak, Z.	2012	Does not include males and females

Purinergic receptors expressed in human skeletal muscle fibres	BornÃ¸, A.; Ploug, T.; Bune, L. T.; Rosenmeier, J. B.; Thaning, P.	2012	Does not include male and female data
Effect of an amino acid, protein, and carbohydrate mixture on net muscle protein balance after resistance exercise	Borsheim, E.; Aarsland, A.; Wolfe, R. R.	2004	Did not perform fiber type analysis
Effect of carbohydrate intake on net muscle protein synthesis during recovery from resistance exercise	BÃ¸rsheim, E.; Cree, M. G.; Tipton, K. D.; Elliott, T. A.; Aarsland, A.; Wolfe, R. R.	2004	Did not perform fiber type analysis
Essential amino acids and muscle protein recovery from resistance exercise	BÃ¸rsheim, E.; Tipton, K. D.; Wolf, S. E.; Wolfe, R. R.	2002	Did not perform fiber type analysis
Increased amyloid Î²-peptide uptake in skeletal muscle is induced by hyposialylation and may account for apoptosis in GNE myopathy	Bosch-MoratÃ³, M.; Iriondo, C.; Guivernau, B.; Valls-Comamala, V.; Vidal, N.; OlivÃ©, M.; Querfurth, H.; MuÃ±oz, F. J.	2016	Does not include males and females
LMNA mutations, skeletal muscle lipid metabolism, and insulin resistance	Boschmann, M.; Engeli, S.; Moro, C.; Luedtke, A.; Adams, F.; Gorzelniak, K.; Rahn, G.; MÃ¤hler, A.; Dobberstein, K.; KrÃ¼ger, A.; Schmidt, S.; Spuler, S.; Luft, F. C.; Smith, S. R.; Schmidt, H. H.; Jordan, J.	2010	Does not include males and females
Metabolic and hemodynamic responses to exercise in subcutaneous adipose tissue and skeletal muscle	Boschmann, M.; Rosenbaum, M.; Leibel, R. L.; Segal, K. R.	2002	Did not perform muscle biopsy
The effect of fatigue on store and re-use of elastic energy in slow and fast types of human skeletal muscle	Bosco, C.; Tihanyi, J.; Latteri, F.; Fekete, G.; Apor, P.; Rusko, H.	1986	Does not report sex of subjects
Skeletal muscle apoptosis is not increased in gastric cancer patients with mild-moderate weight loss	Bossola, M.; Mirabella, M.; Ricci, E.; Costelli, P.; Pacelli, F.; Tortorelli, A. P.; Muscaritoli, M.; Rossi Fanelli, F.; Baccino, F. M.; Tonali, P. A.; Doglietto, G. B.	2006	No healthy subjects or controls
Regulation of muscle cathepsin B proteolytic activity in protein-depleted patients with chronic diseases	Bosutti, A.; Toigo, G.; Ciocchi, B.; Situlin, R.; Guarnieri, G.; Biolo, G.	2002	Does not include males and females
Gene expression analysis in myotonic dystrophy: indications for a common molecular pathogenic pathway in DM1 and DM2	Botta, A.; Vallo, L.; Rinaldi, F.; Bonifazi, E.; Amati, F.; Biancolella, M.; Gambardella, S.; Mancinelli, E.; Angelini, C.; Meola, G.; Novelli, G.	2007	Did not perform fiber type analysis
Force-velocity properties of human skeletal muscle fibres: myosin heavy chain isoform and temperature dependence	Bottinelli, R.; Canepari, M.; Pellegrino, M. A.; Reggiani, C.	1996	Does not include males and females
A mutant tropomyosin that causes hypertrophic cardiomyopathy is expressed in vivo and associated with an increased calcium sensitivity	Bottinelli, R.; Coviello, D. A.; Redwood, C. S.; Pellegrino, M. A.; Maron, B. J.; Spirito, P.; Watkins, H.; Reggiani, C.	1998	Does not include males and females
Expression and regulation by insulin of low-density lipoprotein receptor-related protein mRNA in human skeletal muscle	Boucher, P.; Ducluzeau, P. H.; Davelu, P.; Andreelli, F.; Vallier, P.; Riou, J. P.; Laville, M.; Vidal, H.	2002	Did not perform fiber type analysis
Enhanced glucose metabolism is preserved in cultured primary myotubes from obese donors in response to exercise training	Bourlier, V.; Saint-Laurent, C.; Louche, K.; Badin, P. M.; Thalamas, C.; de Glisezinski, I.; Langin, D.; Sengenes, C.; Moro, C.	2013	Does not include males and females
Maintained peak leg and pulmonary VO2 despite substantial reduction in muscle mitochondrial capacity	Boushel, R.; Gnaiger, E.; Larsen, F. J.; Helge, J. W.; GonzÃ¡lez-Alonso, J.; Ara, I.; Munch-	2015	Does not include male and female data

	Andersen, T.; van Hall, G.; SÃ¸ndergaard, H.; Saltin, B.; Calbet, J. A.		
Patients with type 2 diabetes have normal mitochondrial function in skeletal muscle	Boushel, R.; Gnaiger, E.; Schjerling, P.; Skovbro, M.; KraunsÃ¸e, R.; Dela, F.	2007	Does not include males and females
Skeletal muscle stem cell characteristics and myonuclei content in patients with rheumatoid arthritis: a cross-sectional study	Boutrup, R. J.; Farup, J.; Vissing, K.; Kjaer, M.; Mikkelsen, U. R.	2018	Does not include male and female data
Malonyl CoenzymeA decarboxylase regulates lipid and glucose metabolism in human skeletal muscle	Bouzakri, K.; Austin, R.; Rune, A.; Lassman, M. E.; Garcia-Roves, P. M.; Berger, J. P.; Krook, A.; Chibalin, A. V.; Zhang, B. B.; Zierath, J. R.	2008	Does not include male and female data
Potassium, sodium and magnesium contents in skeletal muscle of renal stone-formers: a study in an area of low potassium intake	Bovornpadungkitti, S.; Sriboonlue, P.; Tavichakorntrakool, R.; Prasongwatana, V.; Suwantrai, S.; Predanon, C.; Tosukhowong, P.; Suntarapa, S.	2000	Did not perform fiber type analysis
Dermatomyositis, polymyositis, and Coxsackie-B-virus infection	Bowles, N. E.; Dubowitz, V.; Sewry, C. A.; Archard, L. C.	1987	Only in children (0-17 years)
Hyperreactive (hyaline, opaque, dark) muscle fibers in Duchenne dystrophy. A biopsy study of 16 dystrophy and 205 other neuronmuscular disease cases and controls	Boxler, K.; Jerusalem, F.	1978	Does not report sex of subjects
Masseter muscle adaptation following surgical correction of vertical maxillary excess	Boyd, S. B.; Gonyea, W. J.; Legan, H. L.; Bell, W. H.	1989	Does not include male and female data
Acute endurance exercise increases plasma membrane fatty acid transport proteins in rat and human skeletal muscle	Bradley, N. S.; Snook, L. A.; Jain, S. S.; Heigenhauser, G. J.; Bonen, A.; Spriet, L. L.	2012	Did not perform fiber type analysis
Skeletal muscle neuronal nitric oxide synthase micro protein is reduced in people with impaired glucose homeostasis and is not normalized by exercise training	Bradley, S. J.; Kingwell, B. A.; Canny, B. J.; McConell, G. K.	2007	Did not perform fiber type analysis
Platelet bioenergetics correlate with muscle energetics and are altered in older adults	Braganza, A.; Corey, C. G.; Santanasto, A. J.; Distefano, G.; Coen, P. M.; Glynn, N. W.; Nouraie, S. M.; Goodpaster, B. H.; Newman, A. B.; Shiva, S.	2019	Did not perform fiber type analysis
Muscle fiber type composition and effects of vocal fold immobilization on the two compartments of the human posterior cricoarytenoid: a case study of four patients	Brandon, C. A.; Rosen, C.; Georgelis, G.; Horton, M. J.; Mooney, M. P.; Sciote, J. J.	2003	No cross-sectional area data
Decreased NADH dehydrogenase and ubiquinol-cytochrome c oxidoreductase in peripheral arterial disease	Brass, E. P.; Hiatt, W. R.; Gardner, A. W.; Hoppel, C. L.	2001	Did not perform fiber type analysis
Association between mitochondrial dysfunction and severity and outcome of septic shock	Brealey, D.; Brand, M.; Hargreaves, I.; Heales, S.; Land, J.; Smolenski, R.; Davies, N. A.; Cooper, C. E.; Singer, M.	2002	Does not report sex of subjects
Skeletal muscle abnormalities in pulmonary arterial hypertension	Breda, A. P.; Pereira de Albuquerque, A. L.; Jardim, C.; Morinaga, L. K.; Suesada, M. M.; Fernandes, C. J.; Dias, B.; LourenÃ§o, R. B.; Salge, J. M.; Souza, R.	2014	Does not include male and female data

The influence of carbohydrate-protein co-ingestion following endurance exercise on myofibrillar and mitochondrial protein synthesis	Breen, L.; Philp, A.; Witard, O. C.; Jackman, S. R.; Selby, A.; Smith, K.; Baar, K.; Tipton, K. D.	2011	Does not include males and females
Individual Response Variation in the Effects of Weight Loss and Exercise on Insulin Sensitivity and Cardiometabolic Risk in Older Adults	Brennan, A. M.; Standley, R. A.; Yi, F.; Carnero, E. A.; Sparks, L. M.; Goodpaster, B. H.	2020	Does not include male and female data
Skeletal muscle vitamin D in patients with end stage osteoarthritis of the knee	Brennan-Speranza, T. C.; Mor, D.; Mason, R. S.; Bartlett, J. R.; Duque, G.; Levinger, I.; Levinger, P.	2017	Did not perform fiber type analysis
Changes in skeletal muscle histology and metabolism in patients undergoing exercise deconditioning: effect of propionyl-L-carnitine	Brevetti, G.; Fanin, M.; De Amicis, V.; Carrozzo, R.; Di Lello, F.; Martone, V. D.; Angelini, C.	1997	Does not include males and females
Muscle fibrosis and maladaptation occur progressively in CKD and are rescued by dialysis	Brightwell, C. R.; Kulkarni, A. S.; Paredes, W.; Zhang, K.; Perkins, J. B.; Gatlin, K. J.; Custodio, M.; Farooq, H.; Zaidi, B.; Pai, R.; Buttar, R. S.; Tang, Y.; Melamed, M. L.; Hostetter, T. H.; Pessin, J. E.; Hawkins, M.; Fry, C. S.; Abramowitz, M. K.	2021	Does not include male and female data
The time course of the adaptations of human muscle proteome to bed rest and the underlying mechanisms	Brocca, L.; Cannavino, J.; Coletto, L.; Biolo, G.; Sandri, M.; Bottinelli, R.; Pellegrino, M. A.	2012	Does not include males and females
Isoenergetic dietary protein restriction decreases myosin heavy chain IIx fraction and myosin heavy chain production in humans	Brodsky, I. G.; Suzara, D.; Hornberger, T. A.; Goldspink, P.; Yarasheski, K. E.; Smith, S.; Kukowski, J.; Esser, K.; Bedno, S.	2004	Intervention with no baseline data
Deficient leukemia inhibitory factor signaling in muscle precursor cells from patients with type 2 diabetes	Broholm, C.; Brandt, C.; Schultz, N. S.; Nielsen, A. R.; Pedersen, B. K.; Scheele, C.	2012	Did not perform fiber type analysis
The human posterior cricoarytenoid (PCA) muscle and diaphragm. A histochemical comparison as a basis for reinnervation attempts	BrÃ¸ndbo, K.; Dahl, H. A.; Teig, E.; Gujord, K. M.	1986	Does not include males and females
The histographic analysis of human muscle biopsies with regard to fiber types. 1. Adult male and female	Brooke, M. H.; Engel, W. K.	1969	No healthy subjects or controls
The stiffness response of type IIa fibres after eccentric exercise-induced muscle damage is dependent on ACTN3 r577X polymorphism	Broos, S.; Malisoux, L.; Theisen, D.; Van Thienen, R.; Francaux, M.; Thomis, M. A.; Deldicque, L.	2019	Does not include males and females
Nutritional assessment and muscle energy metabolism in severe chronic congestive heart failure--effects of long-term dietary supplementation	Broqvist, M.; Arnqvist, H.; DahlstrÃ¶m, U.; Larsson, J.; Nylander, E.; Permert, J.	1994	No healthy subjects or controls
Muscle energy metabolism in severe chronic congestive heart failure--effect of treatment with enalapril	Broqvist, M.; DahlstrÃ¶m, U.; Karlsson, E.; Larsson, J.	1992	No healthy subjects or controls
Skeletal muscle mitochondria in the elderly: effects of physical fitness and exercise training	Broskey, N. T.; Greggio, C.; Boss, A.; Boutant, M.; Dwyer, A.; Schlueter, L.; Hans, D.; Gremion, G.; Kreis, R.; Boesch, C.; Canto, C.; Amati, F.	2014	Did not perform fiber type analysis
p38 MAPK activation upregulates proinflammatory pathways in skeletal muscle cells from insulin-resistant type 2 diabetic patients	Brown, A. E.; Palsgaard, J.; Borup, R.; Avery, P.; Gunn, D. A.; De Meyts, P.; Yeaman, S. J.; Walker, M.	2015	Did not perform fiber type analysis

A quantitative study of the histochemical and morphometric characteristics of the human cricopharyngeus muscle	Brownlow, H.; Whitmore, I.; Willan, P. L.	1989	Autopsies
Muscle oxidative capacity is a better predictor of insulin sensitivity than lipid status	Bruce, C. R.; Anderson, M. J.; Carey, A. L.; Newman, D. G.; Bonen, A.; Kriketos, A. D.; Cooney, G. J.; Hawley, J. A.	2003	Did not perform fiber type analysis
Endurance training in obese humans improves glucose tolerance and mitochondrial fatty acid oxidation and alters muscle lipid content	Bruce, C. R.; Thrush, A. B.; Mertz, V. A.; Bezaire, V.; Chabowski, A.; Heigenhauser, G. J.; Dyck, D. J.	2006	Did not perform fiber type analysis
Histochemical staining. Its use for detection of glyceraldehyde-3-phosphate dehydrogenase in skeletal muscle	Brumback, R. A.; Susag, M. E.; Gerst, J. W.	1981	Does not report sex of subjects
Diet and exercise reduce low-grade inflammation and macrophage infiltration in adipose tissue but not in skeletal muscle in severely obese subjects	Bruun, J. M.; Helge, J. W.; Richelsen, B.; Stallknecht, B.	2006	Did not perform fiber type analysis
Interleukin-18 in plasma and adipose tissue: effects of obesity, insulin resistance, and weight loss	Bruun, J. M.; Stallknecht, B.; Helge, J. W.; Richelsen, B.	2007	Did not perform fiber type analysis
Loss of oxidative defense and potential blockade of satellite cell maturation in the skeletal muscle of patients with cancer but not in the healthy elderly	BrzeszczyÅ„ska, J.; Johns, N.; Schilb, A.; Degen, S.; Degen, M.; Langen, R.; Schols, A.; Glass, D. J.; Roubenoff, R.; Greig, C. A.; Jacobi, C.; Fearon, KCh; Ross, J. A.	2016	Did not perform fiber type analysis
Alterations in the in vitro and in vivo regulation of muscle regeneration in healthy ageing and the influence of sarcopenia	BrzeszczyÅ„ska, J.; Meyer, A.; McGregor, R.; Schilb, A.; Degen, S.; Tadini, V.; Johns, N.; Langen, R.; Schols, A.; Glass, D. J.; Roubenoff, R.; Ross, J. A.; Fearon, K. C. H.; Greig, C. A.; Jacobi, C.	2018	Did not perform fiber type analysis
Direct effects of locally administered lipopolysaccharide on glucose, lipid, and protein metabolism in the placebo-controlled, bilaterally infused human leg	Buhl, M.; Bosnjak, E.; Vendelbo, M. H.; Gjedsted, J.; Nielsen, R. R.; K. HafstrÃ¸m T; Vestergaard, E. T.; Jessen, N.; TÃ¸nnesen, E.; MÃ¸ller, A. B.; Pedersen, S. B.; Pilegaard, H.; BiensÃ¸, R. S.; JÃ¸rgensen, J. O.; MÃ¸ller, N.	2013	Does not include males and females
Skeletal muscle DNA methylation modifications and psychopharmacologic treatment in bipolar disorder	Burghardt, K. J.; Howlett, B. H.; Sanders, E.; Dass, S. E.; Msallaty, Z.; Mallisho, A.; Seyoum, B.; Yi, Z.	2019	Did not perform fiber type analysis
Similar metabolic adaptations during exercise after low volume sprint interval and traditional endurance training in humans	Burgomaster, K. A.; Howarth, K. R.; Phillips, S. M.; Rakobowchuk, M.; Macdonald, M. J.; McGee, S. L.; Gibala, M. J.	2008	Did not perform fiber type analysis
Changes in skeletal muscle morphology and biochemistry after cardiac transplantation	BussiÃ¨res, L. M.; Pflugfelder, P. W.; Taylor, A. W.; Noble, E. G.; Kostuk, W. J.	1997	Does not include males and females
Redox balance following magnetic stimulation training in the quadriceps of patients with severe COPD	Bustamante, V.; Casanova, J.; LÃ³pez de SantamarÃa, E.; Mas, S.; SellarÃ©s, J.; Gea, J.; GÃ¡ldiz, J. B.; Barreiro, E.	2008	Does not include males and females
Muscle regeneration following repair of the rotator cuff	Butt, U.; Rashid, M. S.; Temperley, D.; Crank, S.; Birch, A.; Freemont, A. J.; Trail, I. A.	2016	No healthy subjects or controls
Muscle fiber types in thoracic erector spinae muscles. Fiber types in idiopathic and other forms of scoliosis	Bylund, P.; Jansson, E.; Dahlberg, E.; Eriksson, E.	1987	Only in children (0-17 years)

Fine structural changes in electrostimulated human skeletal muscle. Evidence for predominant effects on fast muscle fibres	Cabric, M.; Appell, H. J.; Resic, A.	1988	Does not include males and females
Chaperone-mediated autophagy components are upregulated in sporadic inclusion-body myositis muscle fibres	Cacciottolo, M.; Nogalska, A.; D'Agostino, C.; Engel, W. K.; Askanas, V.	2013	Did not perform fiber type analysis
Artificial gravity as a countermeasure to microgravity: a pilot study examining the effects on knee extensor and plantar flexor muscle groups	Caiozzo, V. J.; Haddad, F.; Lee, S.; Baker, M.; Paloski, W.; Baldwin, K. M.	2009	Does not include males and females
The membrane-associated 40 KD fatty acid binding protein (Berk's protein), a putative fatty acid transporter is present in human skeletal muscle	Calles-Escandon, J.; Sweet, L.; Ljungqvist, O.; Hirshman, M. F.	1996	Did not perform fiber type analysis
Muscle and adipose tissue morphology, insulin sensitivity and beta-cell function in diabetic and nondiabetic obese patients: effects of bariatric surgery	Camastra, S.; Vitali, A.; Anselmino, M.; Gastaldelli, A.; Bellini, R.; Berta, R.; Severi, I.; Baldi, S.; Astiarraga, B.; Barbatelli, G.; Cinti, S.; Ferrannini, E.	2017	Did not perform fiber type analysis
No excess of mitochondrial DNA deletions within muscle in progressive multiple sclerosis	Campbell, G. R.; Reeve, A. K.; Ziabreva, I.; Reynolds, R.; Turnbull, D. M.; Mahad, D. J.	2013	Does not include male and female data
Low intensity exercise in humans accelerates mitochondrial ATP production and pulmonary oxygen kinetics during subsequent more intense exercise	Campbell-O'Sullivan, S. P.; Constantin-Teodosiu, D.; Peirce, N.; Greenhaff, P. L.	2002	Does not include males and females
Acute myopathy after liver transplantation	Campellone, J. V.; Lacomis, D.; Kramer, D. J.; Van Cott, A. C.; Giuliani, M. J.	1998	Did not perform fiber type analysis
Possible role for nitric oxide dysregulation in critical illness myopathy	Capasso, M.; Di Muzio, A.; Pandolfi, A.; Pace, M.; Di Tomo, P.; Ragno, M.; Uncini, A.	2008	Did not perform fiber type analysis
Comparative proteomic analyses of Duchenne muscular dystrophy and Becker muscular dystrophy muscles: changes contributing to preserve muscle function in Becker muscular dystrophy patients	Capitanio, D.; Moriggi, M.; Torretta, E.; Barbacini, P.; De Palma, S.; ViganÃ², A.; LochmÃ¼ller, H.; Muntoni, F.; Ferlini, A.; Mora, M.; Gelfi, C.	2020	Does not include males and females
Comparison of protein expression in human deltoideus and vastus lateralis muscles using two-dimensional gel electrophoresis	Capitanio, D.; ViganÃ², A.; Ricci, E.; Cerretelli, P.; Wait, R.; Gelfi, C.	2005	Does not include males and females
Aging-associated genes and let-7 microRNAs: a contribution to myogenic program dysregulation in oculopharyngeal muscular dystrophy	Cappelletti, C.; Galbardi, B.; Bruttini, M.; Salerno, F.; Canioni, E.; Pasanisi, M. B.; Rodolico, C.; Brizzi, T.; Mora, M.; Renieri, A.; Maggi, L.; Bernasconi, P.; Mantegazza, R.	2019	Did not perform fiber type analysis
Effects of energy deficit, dietary protein, and feeding on intracellular regulators of skeletal muscle proteolysis	Carbone, J. W.; Margolis, L. M.; McClung, J. P.; Cao, J. J.; Murphy, N. E.; Sauter, E. R.; Combs, G. F., Jr.; Young, A. J.; Pasiakos, S. M.	2013	Did not perform fiber type analysis
Effects of short-term energy deficit on muscle protein breakdown and intramuscular proteolysis in normal-weight young adults	Carbone, J. W.; Pasiakos, S. M.; Vislocky, L. M.; Anderson, J. M.; Rodriguez, N. R.	2014	Did not perform fiber type analysis

Discordant gene expression in skeletal muscle and adipose tissue of patients with type 2 diabetes: effect of interleukin-6 infusion	Carey, A. L.; Petersen, E. W.; Bruce, C. R.; Southgate, R. J.; Pilegaard, H.; Hawley, J. A.; Pedersen, B. K.; Febbraio, M. A.	2006	Does not include males and females
Insulin receptor kinase in human skeletal muscle from obese subjects with and without noninsulin dependent diabetes	Caro, J. F.; Sinha, M. K.; Raju, S. M.; Ittoop, O.; Pories, W. J.; Flickinger, E. G.; Meelheim, D.; Dohm, G. L.	1987	No healthy subjects or controls
Necrosis of capillaries in denervation atrophy of human skeletal muscle	Carpenter, S.; Karpati, G.	1982	Does not report sex of subjects
Patients with obstructive sleep apnea exhibit genioglossus dysfunction that is normalized after treatment with continuous positive airway pressure	Carrera, M.; BarbÃ©, F.; Sauleda, J.; TomÃ¡s, M.; GÃ³mez, C.; AgustÃ, A. G.	1999	Does not include males and females
Effects of obesity upon genioglossus structure and function in obstructive sleep apnoea	Carrera, M.; BarbÃ©, F.; Sauleda, J.; TomÃ¡s, M.; GÃ³mez, C.; Santos, C.; AgustÃ, A. G.	2004	Does not include male and female data
Human soleus and vastus lateralis muscle protein metabolism with an amino acid infusion	Carroll, C. C.; Fluckey, J. D.; Williams, R. H.; Sullivan, D. H.; Trappe, T. A.	2005	Does not include male and female data
Effect of acute hypohydration on glycemic regulation in healthy adults: a randomized crossover trial	Carroll, H. A.; Templeman, I.; Chen, Y. C.; Edinburgh, R. M.; Burch, E. K.; Jewitt, J. T.; Povey, G.; Robinson, T. D.; Dooley, W. L.; Jones, R.; Tsintzas, K.; Gallo, W.; Melander, O.; Thompson, D.; James, L. J.; Johnson, L.; Betts, J. A.	2019	Did not perform fiber type analysis
Distribution of capillaries in normal and diseased human skeletal muscle	Carry, M. R.; Ringel, S. P.; Starcevich, J. M.	1986	Does not include males and females
PYGM mRNA expression in McArdle disease: Demographic, clinical, morphological and genetic features	Carvalho, A. A. S.; Christofolini, D. M.; Perez, M. M.; Alves, B. C. A.; Rodart, I.; Figueiredo, F. W. S.; Turke, K. C.; Feder, D.; Junior, M. C. F.; Nucci, A. M.; Fonseca, F. L. A.	2020	Does not report sex of subjects
The effect of zidovudine on skeletal muscle mtDNA in HIV-1 infected patients with mild or no muscle dysfunction	Casademont, J.; Barrientos, A.; Grau, J. M.; Pedrol, E.; Estivill, X.; Urbano-MÃ¡rquez, A.; Nunes, V.	1996	No healthy subjects or controls
Glycogen resynthesis in human muscle fibre types following exercise-induced glycogen depletion	Casey, A.; Short, A. H.; Hultman, E.; Greenhaff, P. L.	1995	Does not include males and females
Fasting inhibits insulin-mediated glycolysis and anaplerosis in human skeletal muscle	Castillo, C. E.; Katz, A.; Spencer, M. K.; Yan, Z.; Nyomba, B. L.	1991	Does not include males and females
Reorganization of the nuclear compartments involved in transcription and RNA processing in myonuclei of type I spinal muscular atrophy	Castillo-Iglesias, M. S.; Berciano, M. T.; Narcis, J. O.; Val-Bernal, J. F.; Rodriguez-Rey, J. C.; Tapia, O.; Lafarga, M.	2019	Case studies
Influence of complete spinal cord injury on skeletal muscle within 6 mo of injury	Castro, M. J.; Apple, D. F., Jr.; Staron, R. S.; Campos, G. E.; Dudley, G. A.	1999	Does not include male and female data
High-intensity high-volume swimming induces more robust signaling through PGC-1Î± and AMPK activation than sprint interval swimming in m. triceps brachii	Casuso, R. A.; Plaza-DÃaz, J.; Ruiz-Ojeda, F. J.; AragÃ³n-Vela, J.; Robles-Sanchez, C.; Nordsborg, N. B.; Hebberecht, M.; Salmeron, L. M.; Huertas, J. R.	2017	Does not include males and females

Response to Electrostimulation Is Impaired in Muscle Cells from Patients with Chronic Obstructive Pulmonary Disease	Catteau, M.; Passerieux, E.; Blervaque, L.; Gouzi, F.; Ayoub, B.; Hayot, M.; PomiÃ¨s, P.	2021	Did not perform fiber type analysis
Effects of chronic iron deficiency anaemia on myoglobin content, enzyme activity, and capillary density in the human skeletal muscle	Celsing, F.; Ekblom, B.; SylvÃ©n, C.; Everett, J.; Astrand, P. O.	1988	Did not perform fiber type analysis
The metastasis promoting protein S100A4 is increased in idiopathic inflammatory myopathies	Cerezo, L. A.; KuncovÃ¡, K.; Mann, H.; TomcÃk, M.; ZÃ¡mecnÃk, J.; Lukanidin, E.; Neidhart, M.; Gay, S.; Grigorian, M.; Vencovsky, J.; Senolt, L.	2011	Did not perform fiber type analysis
Random mtDNA deletions and functional consequence in aged human skeletal muscle	Chabi, B.; Mousson de Camaret, B.; Chevrollier, A.; Boisgard, S.; Stepien, G.	2005	Does not report sex of subjects
Impaired redox status and cytochrome c oxidase deficiency in patients with polymyalgia rheumatica	Chariot, P.; Chevalier, X.; Yerroum, M.; Drogou, I.; Authier, F. J.; Gherardi, R.	2001	No cross-sectional area data
Cytochrome c oxidase deficiencies in the muscle of patients with inflammatory myopathies	Chariot, P.; Ruet, E.; Authier, F. J.; Labes, D.; Poron, F.; Gherardi, R.	1996	Does not report sex of subjects
Can obesity-induced inflammation in skeletal muscle and intramuscular adipose tissue accurately detect liver fibrosis?	Chasapi, A.; Balampanis, K.; Kourea, E.; Kalfarentzos, F.; Lambadiari, V.; Lambrou, G. I.; Melachrinou, M.; Sotiropoulou-Bonikou, G.	2018	No healthy subjects or controls
SRC-3/AIB-1 may Enhance Hepatic NFATC1 Transcription and Mediate Inflammation in a Tissue-Specific Manner in Morbid Obesity	Chasapi, A.; Balampanis, K.; Tanoglidi, A.; Kourea, E.; Lambrou, G. I.; Lambadiari, V.; Kalfarentzos, F.; Hatziagelaki, E.; Melachrinou, M.; Sotiropoulou-Bonikou, G.	2020	Did not perform fiber type analysis
The regulation of glycogen phosphorylase and glycogen breakdown in human skeletal muscle	Chasiotis, D.	1983	Did not perform fiber type analysis
Retinol-binding protein 4 is associated with impaired glucose tolerance but not with whole body or hepatic insulin resistance in Mexican Americans	Chavez, A. O.; Coletta, D. K.; Kamath, S.; Cromack, D. T.; Monroy, A.; Folli, F.; DeFronzo, R. A.; Tripathy, D.	2009	Did not perform fiber type analysis
Effect of short-term free Fatty acids elevation on mitochondrial function in skeletal muscle of healthy individuals	Chavez, A. O.; Kamath, S.; Jani, R.; Sharma, L. K.; Monroy, A.; Abdul-Ghani, M. A.; Centonze, V. E.; Sathyanarayana, P.; Coletta, D. K.; Jenkinson, C. P.; Bai, Y.; Folli, F.; Defronzo, R. A.; Tripathy, D.	2010	Did not perform fiber type analysis
Bariatric surgery in morbidly obese insulin resistant humans normalises insulin signalling but not insulin-stimulated glucose disposal	Chen, M. Z.; Hudson, C. A.; Vincent, E. E.; de Berker, D. A.; May, M. T.; Hers, I.; Dayan, C. M.; Andrews, R. C.; TavarÃ©, J. M.	2015	Did not perform fiber type analysis
Transcriptional pathways associated with skeletal muscle disuse atrophy in humans	Chen, Y. W.; Gregory, C. M.; Scarborough, M. T.; Shi, R.; Walter, G. A.; Vandenborne, K.	2007	Did not perform fiber type analysis
Effects of detraining on enzymes of energy metabolism in individual human muscle fibers	Chi, M. M.; Hintz, C. S.; Coyle, E. F.; Martin, W. H., 3rd; Ivy, J. L.; Nemeth, P. M.; Holloszy, J. O.; Lowry, O. H.	1983	Does not include males and females
Changes in indices of antioxidant status, lipid peroxidation and inflammation in human skeletal muscle after eccentric muscle actions	Child, R.; Brown, S.; Day, S.; Donnelly, A.; Roper, H.; Saxton, J.	1999	Did not perform fiber type analysis
Evaluation of muscle oxidative potential by 31P-MRS during incremental exercise in old and young humans	Chilibeck, P. D.; McCreary, C. R.; Marsh, G. D.; Paterson, D. H.; Noble, E. G.; Taylor, A. W.; Thompson, R. T.	1998	Does not include male and female data

The effect of strength training on estimates of mitochondrial density and distribution throughout muscle fibres	Chilibeck, P. D.; Syrotuik, D. G.; Bell, G. J.	1999	Does not include male and female data
Low-dose benzo(a)pyrene and its epoxide metabolite inhibit myogenic differentiation in human skeletal muscle-derived progenitor cells	Chiu, C. Y.; Yen, Y. P.; Tsai, K. S.; Yang, R. S.; Liu, S. H.	2014	Does not include male and female data
Morin Stain Detects Aluminum-Containing Macrophages in Macrophagic Myofasciitis and Vaccination Granuloma With High Sensitivity and Specificity	Chkheidze, R.; Burns, D. K.; White, C. L.; Castro, D.; Fuller, J.; Cai, C.	2017	No healthy subjects or controls
Calcium-activated force of human muscle fibers following a standardized eccentric contraction	Choi, S. J.; Widrick, J. J.	2010	Does not include male and female data
Changes in dysferlin, proteins from dystrophin glycoprotein complex, costameres, and cytoskeleton in human soleus and vastus lateralis muscles after a long-term bedrest with or without exercise	Chopard, A.; Arrighi, N.; Carnino, A.; Marini, J. F.	2005	Does not include males and females
Anastomoses of transverse tubules with terminal cisternae in polymyositis	Chou, S. M.; Nonaka, I.; Voice, G. F.	1980	No healthy subjects or controls
Training status diverges muscle diacylglycerol accumulation during free fatty acid elevation	Chow, L. S.; Mashek, D. G.; Austin, E.; Eberly, L. E.; Persson, X. M.; Mashek, M. T.; Seaquist, E. R.; Jensen, M. D.	2014	Did not perform fiber type analysis
Effect of acute physiological free fatty acid elevation in the context of hyperinsulinemia on fiber type-specific IMCL accumulation	Chow, L. S.; Mashek, D. G.; Wang, Q.; Shepherd, S. O.; Goodpaster, B. H.; DubÃ©, J. J.	2017	Does not include male and female data
Exercise training increases glycogen synthase activity and GLUT4 expression but not insulin signaling in overweight nondiabetic and type 2 diabetic subjects	Christ-Roberts, C. Y.; Pratipanawatr, T.; Pratipanawatr, W.; Berria, R.; Belfort, R.; Kashyap, S.; Mandarino, L. J.	2004	Did not perform fiber type analysis
Increased insulin receptor signaling and glycogen synthase activity contribute to the synergistic effect of exercise on insulin action	Christ-Roberts, C. Y.; Pratipanawatr, T.; Pratipanawatr, W.; Berria, R.; Belfort, R.; Mandarino, L. J.	2003	Does not report sex of subjects
Evaluation of functional erythropoietin receptor status in skeletal muscle in vivo: acute and prolonged studies in healthy human subjects	Christensen, B.; Lundby, C.; Jessen, N.; Nielsen, T. S.; Vestergaard, P. F.; MÃ¸ller, N.; Pilegaard, H.; Pedersen, S. B.; Kopchick, J. J.; JÃ¸rgensen, J. O.	2012	Does not include males and females
VO2 kinetics and performance in soccer players after intense training and inactivity	Christensen, P. M.; Krustrup, P.; Gunnarsson, T. P.; Kiilerich, K.; Nybo, L.; Bangsbo, J.	2011	Does not include males and females
Skeletal muscle fiber composition, nutritional status and subjective fatigue during surgical convalescence	Christensen, T.; Nygaard, E.; Kehlet, H.	1988	No healthy subjects or controls
A fast, reliable and sample-sparing method to identify fibre types of single muscle fibres	Christiansen, D.; MacInnis, M. J.; Zacharewicz, E.; Xu, H.; Frankish, B. P.; Murphy, R. M.	2019	Does not include males and females
Acute exercise increases circulating inflammatory markers in overweight and obese compared with lean subjects	Christiansen, T.; Bruun, J. M.; Paulsen, S. K.; Olholm, J.; Overgaard, K.; Pedersen, S. B.; Richelsen, B.	2013	Did not perform fiber type analysis

Exercise training versus diet-induced weight-loss on metabolic risk factors and inflammatory markers in obese subjects: a 12-week randomized intervention study	Christiansen, T.; Paulsen, S. K.; Bruun, J. M.; Pedersen, S. B.; Richelsen, B.	2010	Did not perform fiber type analysis
In vivo activation of ROCK1 by insulin is impaired in skeletal muscle of humans with type 2 diabetes	Chun, K. H.; Choi, K. D.; Lee, D. H.; Jung, Y.; Henry, R. R.; Ciaraldi, T. P.; Kim, Y. B.	2011	Did not perform fiber type analysis
A morphometric study of muscle mitochondria in cytochrome c oxidase deficiency	Chung, S.; Nonaka, I.	1988	Only in children (0-17 years)
Abnormal muscle fructose bisphosphatase activity in malnourished cancer patients	Church, J. M.; Choong, B. Y.; Hill, G. L.	1986	Does not include male and female data
Abnormalities of muscle metabolism and histology in malnourished patients awaiting surgery: effects of a course of intravenous nutrition	Church, J. M.; Choong, S. Y.; Hill, G. L.	1984	Does not include male and female data
EGF receptor (EGFR) inhibition promotes a slow-twitch oxidative, over a fast-twitch, muscle phenotype	Ciano, M.; Mantellato, G.; Connolly, M.; Paul-Clark, M.; Willis-Owen, S.; Moffatt, M. F.; Cookson, Wocm; Mitchell, J. A.; Polkey, M. I.; Hughes, S. M.; Kemp, P. R.; Natanek, S. A.	2019	Does not include male and female data
Insulin and insulin-like growth factor-1 action on human skeletal muscle: preferential effects of insulin-like growth factor-1 in type 2 diabetic subjects	Ciaraldi, T. P.; Carter, L.; Rehman, N.; Mohideen, P.; Mudaliar, S.; Henry, R. R.	2002	Does not include male and female data
Skeletal muscle GLUT1 transporter protein expression and basal leg glucose uptake are reduced in type 2 diabetes	Ciaraldi, T. P.; Mudaliar, S.; Barzin, A.; Macievic, J. A.; Edelman, S. V.; Park, K. S.; Henry, R. R.	2005	Does not include males and females
Tissue-specific expression and regulation of GSK-3 in human skeletal muscle and adipose tissue	Ciaraldi, T. P.; Oh, D. K.; Christiansen, L.; Nikoulina, S. E.; Kong, A. P.; Baxi, S.; Mudaliar, S.; Henry, R. R.	2006	Did not perform fiber type analysis
Effects of the rapid-acting insulin analog glulisine on cultured human skeletal muscle cells: comparisons with insulin and insulin-like growth factor I	Ciaraldi, T. P.; Phillips, S. A.; Carter, L.; Aroda, V.; Mudaliar, S.; Henry, R. R.	2005	Did not perform fiber type analysis
Expression of the beta chemokines CCL3, CCL4, CCL5 and their receptors in idiopathic inflammatory myopathies	Civatte, M.; Bartoli, C.; Schleinitz, N.; Chetaille, B.; Pellissier, J. F.; Figarella-Branger, D.	2005	Did not perform fiber type analysis
DNAJB2 expression in normal and diseased human and mouse skeletal muscle	Claeys, K. G.; Sozanska, M.; Martin, J. J.; Lacene, E.; Vignaud, L.; Stockholm, D.; LaforÃªt, P.; Eymard, B.; Kichler, A.; Scherman, D.; Voit, T.; Israeli, D.	2010	Does not include male and female data
Adaptations in human neuromuscular function following prolonged unweighting: I. Skeletal muscle contractile properties and applied ischemia efficacy	Clark, B. C.; Fernhall, B.; Ploutz-Snyder, L. L.	2006	Does not include male and female data
Changes in the power-duration relationship following prolonged exercise: estimation using conventional and all-out protocols and relationship with muscle glycogen	Clark, I. E.; Vanhatalo, A.; Thompson, C.; Wylie, L. J.; Bailey, S. J.; Kirby, B. S.; Wilkins, B. W.; Jones, A. M.	2019	Does not include males and females

Effects of live high, train low hypoxic exposure on lactate metabolism in trained humans	Clark, S. A.; Aughey, R. J.; Gore, C. J.; Hahn, A. G.; Townsend, N. E.; Kinsman, T. A.; Chow, C. M.; McKenna, M. J.; Hawley, J. A.	2004	Does not include males and females
Maximal isometric strength and fiber type composition in power and endurance athletes	Clarkson, P. M.; Kroll, W.; McBride, T. C.	1980	Does not include males and females
Age, isometric strength, rate of tension development and fiber type composition	Clarkson, P. M.; Kroll, W.; Melchionda, A. M.	1981	Does not include males and females
Isokinetic strength, endurance, and fiber type composition in elite American paddlers	Clarkson, P. M.; Kroll, W.; Melchionda, A. M.	1982	Does not include male and female data
Changes in phosphatidylcholine fatty acid composition are associated with altered skeletal muscle insulin responsiveness in normal man	Clore, J. N.; Harris, P. A.; Li, J.; Azzam, A.; Gill, R.; Zuelzer, W.; Rizzo, W. B.; Blackard, W. G.	2000	Does not include male and female data
Morphometric analysis of gastrocnemius muscle biopsies from patients with peripheral arterial disease: objective grading of muscle degeneration	Cluff, K.; Miserlis, D.; Naganathan, G. K.; Pipinos,, II; Koutakis, P.; Samal, A.; McComb, R. D.; Subbiah, J.; Casale, G. P.	2013	Does not include male and female data
Calf muscle adaptation to peripheral vascular disease	Clyne, C. A.; Mears, H.; Weller, R. O.; O'Donnell, T. F.	1985	Does not include male and female data
Exercise and Weight Loss Improve Muscle Mitochondrial Respiration, Lipid Partitioning, and Insulin Sensitivity After Gastric Bypass Surgery	Coen, P. M.; Menshikova, E. V.; Distefano, G.; Zheng, D.; Tanner, C. J.; Standley, R. A.; Helbling, N. L.; Dubis, G. S.; Ritov, V. B.; Xie, H.; Desimone, M. E.; Smith, S. R.; Stefanovic-Racic, M.; Toledo, F. G.; Houmard, J. A.; Goodpaster, B. H.	2015	Does not include male and female data
A quantitative PCR measurement of messenger RNA expression of IGF-I, IGF-II and IGFBP-5 in human skeletal muscle	Coenen Schimke, J. M.; Ljungqvist, O. H.; Sarkar, G.; Conover, C. A.; Nair, K. S.	1999	Did not perform fiber type analysis
Histochemical and enzymatic characteristics of skeletal muscle in master athletes	Coggan, A. R.; Spina, R. J.; Rogers, M. A.; King, D. S.; Brown, M.; Nemeth, P. M.; Holloszy, J. O.	1990	Does not include males and females
Immunofluorescence studies of skeletal muscle extracellular membranes in diabetes mellitus	Cohn, R. A.; Mauer, S. M.; Barbosa, J.; Michael, A. F.	1978	Did not perform fiber type analysis
Changes of laminin beta 2 chain expression in congenital muscular dystrophy	Cohn, R. D.; Herrmann, R.; Wewer, U. M.; Voit, T.	1997	Only in children (0-17 years)
Effect of acute physiological hyperinsulinemia on gene expression in human skeletal muscle in vivo	Coletta, D. K.; Balas, B.; Chavez, A. O.; Baig, M.; Abdul-Ghani, M.; Kashyap, S. R.; Folli, F.; Tripathy, D.; Mandarino, L. J.; Cornell, J. E.; Defronzo, R. A.; Jenkinson, C. P.	2008	Did not perform fiber type analysis
Autocrine and immune cell-derived BDNF in human skeletal muscle: implications for myogenesis and tissue regeneration	Colombo, E.; Bedogni, F.; Lorenzetti, I.; Landsberger, N.; Previtali, S. C.; Farina, C.	2013	Did not perform fiber type analysis
The neurotrophin receptor p75NTR is induced on mature myofibres in inflammatory myopathies and promotes myotube survival to inflammatory stress	Colombo, E.; Romaggi, S.; Blasevich, F.; Mora, M.; Falcone, C.; LochmÃ¼ller, H.; Morandi, L.; Farina, C.	2012	Does not report sex of subjects

Deficient function of the sarcoplasmic reticulum in patients susceptible to malignant hyperthermia	Condrescu, M.; LÃ³pez, J. R.; Medina, P.; Alamo, L.	1987	Did not perform fiber type analysis
Effects of chronic renal failure on enzymes of energy metabolism in individual human muscle fibers	Conjard, A.; Ferrier, B.; Martin, M.; Caillette, A.; Carrier, H.; Baverel, G.	1995	Does not include male and female data
Congenital muscular dystrophy syndromes distinguished by alkaline and acid phosphatase, merosin, and dystrophin staining	Connolly, A. M.; Pestronk, A.; Planer, G. J.; Yue, J.; Mehta, S.; Choksi, R.	1996	Only in children (0-17 years)
Novel events in the molecular regulation of muscle mass in critically ill patients	Constantin, D.; McCullough, J.; Mahajan, R. P.; Greenhaff, P. L.	2011	Does not include males and females
Skeletal muscle molecular responses to resistance training and dietary supplementation in COPD	Constantin, D.; Menon, M. K.; Houchen-Wolloff, L.; Morgan, M. D.; Singh, S. J.; Greenhaff, P.; Steiner, M. C.	2013	Did not perform fiber type analysis
PDC activity and acetyl group accumulation in skeletal muscle during prolonged exercise	Constantin-Teodosiu, D.; Cederblad, G.; Hultman, E.	1992	Does not include males and females
PDC activity and acetyl group accumulation in skeletal muscle during isometric contraction	Constantin-Teodosiu, D.; Cederblad, G.; Hultman, E.	1993	Does not include male and female data
Anaerobic energy production in human skeletal muscle in intense contraction: a comparison of 31P magnetic resonance spectroscopy and biochemical techniques	Constantin-Teodosiu, D.; Greenhaff, P. L.; McIntyre, D. B.; Round, J. M.; Jones, D. A.	1997	Did not perform fiber type analysis
Carnitine metabolism in human muscle fiber types during submaximal dynamic exercise	Constantin-Teodosiu, D.; Howell, S.; Greenhaff, P. L.	1996	Does not report sex of subjects
Human skeletal muscle: sodium MR imaging and quantification-potential applications in exercise and disease	Constantinides, C. D.; Gillen, J. S.; Boada, F. E.; Pomper, M. G.; Bottomley, P. A.	2000	Did not perform muscle biopsy
Muscle-specific Perilipin2 down-regulation affects lipid metabolism and induces myofiber hypertrophy	Conte, M.; Armani, A.; Conte, G.; Serra, A.; Franceschi, C.; Mele, M.; Sandri, M.; Salvioli, S.	2019	Did not perform fiber type analysis
Differential expression of perilipin 2 and 5 in human skeletal muscle during aging and their association with atrophy-related genes	Conte, M.; Vasuri, F.; Bertaggia, E.; Armani, A.; Santoro, A.; Bellavista, E.; Degiovanni, A.; D'Errico-Grigioni, A.; Trisolino, G.; Capri, M.; Franchi, M. V.; Narici, M. V.; Sandri, M.; Franceschi, C.; Salvioli, S.	2015	Did not perform fiber type analysis
Increased expression of Myosin binding protein H in the skeletal muscle of amyotrophic lateral sclerosis patients	Conti, A.; Riva, N.; Pesca, M.; Iannaccone, S.; Cannistraci, C. V.; Corbo, M.; Previtali, S. C.; Quattrini, A.; Alessio, M.	2014	Did not perform fiber type analysis
Satellite cell characterization from aging human muscle	Corbu, A.; Scaramozza, A.; Badiali-DeGiorgi, L.; Tarantino, L.; Papa, V.; Rinaldi, R.; D'Alessandro, R.; Zavatta, M.; Laus, M.; Lattanzi, G.; Cenacchi, G.	2010	Only in children (0-17 years)
Antioxidant property of Propofol in the ischemic and reperfused human skeletal muscle	Corbucci, G. G.; Marchi, A.; Velluti, C.; Chelo, C.; Grella, E.; Lettieri, B.	2002	Does not report sex of subjects
Metabolic aspects of cardiac and skeletal muscle tissues in the condition of hypoxia, ischaemia and reperfusion induced by extracorporeal circulation	Corbucci, G. G.; Menichetti, A.; Cogliati, A.; Ruvolo, C.	1995	Does not include males and females

Protein kinetics in stable heart failure patients	Cortes, C. W.; Thompson, P. D.; Moyna, N. M.; Schluter, M. D.; Leskiw, M. J.; Donaldson, M. R.; Duncan, B. H.; Stein, T. P.	2003	Did not perform fiber type analysis
Influence of training on NIRS muscle oxygen saturation during submaximal exercise	Costes, F.; Prieur, F.; FÃ©asson, L.; Geyssant, A.; BarthÃ©lÃ©my, J. C.; Denis, C.	2001	Does not include male and female data
Increased susceptibility to oxidative damage in post-diabetic human myotubes	Costford, S. R.; Crawford, S. A.; Dent, R.; McPherson, R.; Harper, M. E.	2009	Does not include males and females
Glycogen depletion pattern in human muscle fibres during distance running	Costill, D. L.; Gollnick, P. D.; Jansson, E. D.; Saltin, B.; Stein, E. M.	1973	Does not include males and females
Determination of human muscle pH in needle-biopsy specimens	Costill, D. L.; Sharp, R. L.; Fink, W. J.; Katz, A.	1982	Did not perform fiber type analysis
Isokinetic strength training protocols: do they induce skeletal muscle fiber hypertrophy?	CÃ´tÃ©, C.; Simoneau, J. A.; LagassÃ©, P.; Boulay, M.; Thibault, M. C.; Marcotte, M.; Bouchard, C.	1988	Does not include male and female data
Suction-modified needle biopsy technique for the human soleus muscle	Cotter, J. A.; Yu, A.; Kreitenberg, A.; Haddad, F. H.; Baker, M. J.; Fox, J. C.; Adams, G. R.	2013	No cross-sectional area data
Exercise-induced quadriceps oxidative stress and peripheral muscle dysfunction in patients with chronic obstructive pulmonary disease	Couillard, A.; Maltais, F.; Saey, D.; DebigarÃ©, R.; Michaud, A.; Koechlin, C.; LeBlanc, P.; PrÃ©faut, C.	2003	Does not include males and females
Daily training with high carbohydrate availability increases exogenous carbohydrate oxidation during endurance cycling	Cox, G. R.; Clark, S. A.; Cox, A. J.; Halson, S. L.; Hargreaves, M.; Hawley, J. A.; Jeacocke, N.; Snow, R. J.; Yeo, W. K.; Burke, L. M.	2010	Does not include males and females
Activation of liver X receptors promotes lipid accumulation but does not alter insulin action in human skeletal muscle cells	Cozzone, D.; Debard, C.; Dif, N.; Ricard, N.; Disse, E.; Vouillarmet, J.; Rabasa-Lhoret, R.; Laville, M.; Pruneau, D.; Rieusset, J.; Lefai, E.; Vidal, H.	2006	Did not perform fiber type analysis
Isoform-specific defects of insulin stimulation of Akt/protein kinase B (PKB) in skeletal muscle cells from type 2 diabetic patients	Cozzone, D.; FrÃ¶jdÃ¶, S.; Disse, E.; Debard, C.; Laville, M.; Pirola, L.; Vidal, H.	2008	Did not perform fiber type analysis
Changes in satellite cells in human skeletal muscle after a single bout of high intensity exercise	Crameri, R. M.; Langberg, H.; Magnusson, P.; Jensen, C. H.; SchrÃ¸der, H. D.; Olesen, J. L.; Suetta, C.; Teisner, B.; Kjaer, M.	2004	Does not include males and females
Degeneration and regeneration of motor neurons in psychotic patients	Crayton, J. W.; Meltzer, H. Y.	1979	Does not include male and female data
Increased technetium uptake is not equivalent to muscle necrosis: scintigraphic, morphological and intramuscular pressure analyses of sore muscles after exercise	Crenshaw, A. G.; FridÃ©n, J.; Hargens, A. R.; Lang, G. H.; Thornell, L. E.	1993	Does not include males and females
Extreme endurance training: evidence of capillary and mitochondria compartmentalization in human skeletal muscle	Crenshaw, A. G.; FridÃ©n, J.; Thornell, L. E.; Hargens, A. R.	1991	Does not include males and females
Skeletal muscle gene expression profiling in mitochondrial disorders	Crimi, M.; Bordoni, A.; Menozzi, G.; Riva, L.; Fortunato, F.; Galbiati, S.; Del Bo, R.; Pozzoli, U.; Bresolin, N.; Comi, G. P.	2005	Did not perform fiber type analysis

Markers of oxidative stress in the skeletal muscle of patients on haemodialysis	Crowe, A. V.; McArdle, A.; McArdle, F.; Pattwell, D. M.; Bell, G. M.; Kemp, G. J.; Bone, J. M.; Griffiths, R. D.; Jackson, M. J.	2007	Does not include male and female data
Markers of inflammation and disuse in vastus lateralis of chronic obstructive pulmonary disease patients	Crul, T.; Spruit, M. A.; Gayan-Ramirez, G.; Quarck, R.; Gosselink, R.; Troosters, T.; Pitta, F.; Decramer, M.	2007	Does not include males and females
The pathology of a cricopharyngeal dysphagia	Cruse, J. P.; Edwards, D. A.; Smith, J. F.; Wyllie, J. H.	1979	No healthy subjects or controls
The ultrastruct of normal human muscle in relation to fibre type	Cullen, M. J.; Weightman, D.	1975	Does not include males and females
Junctional membrane Ca(2+) dynamics in human muscle fibers are altered by malignant hyperthermia causative RyR mutation	Cully, T. R.; Choi, R. H.; Bjorksten, A. R.; Stephenson, D. G.; Murphy, R. M.; Launikonis, B. S.	2018	Did not perform fiber type analysis
Acute response and subcellular movement of HSP27, Î±B-crystallin and HSP70 in human skeletal muscle after blood-flow-restricted low-load resistance exercise	Cumming, K. T.; Paulsen, G.; Wernbom, M.; Ugelstad, I.; Raastad, T.	2014	Does not include male and female data
Skeletal muscle wastage in Crohn's disease: a pathway shared with heart failure?	Cuoco, L.; Vescovo, G.; Castaman, R.; Ravara, B.; Cammarota, G.; Angelini, A.; Salvagnini, M.; Dalla Libera, L.	2008	Does not include male and female data
Insulin resistance differentially affects the PI 3-kinase- and MAP kinase-mediated signaling in human muscle	Cusi, K.; Maezono, K.; Osman, A.; Pendergrass, M.; Patti, M. E.; Pratipanawatr, T.; DeFronzo, R. A.; Kahn, C. R.; Mandarino, L. J.	2000	Did not perform fiber type analysis
Exercise increases hexokinase II mRNA, but not activity in obesity and type 2 diabetes	Cusi, K. J.; Pratipanawatr, T.; Koval, J.; Printz, R.; Ardehali, H.; Granner, D. K.; Defronzo, R. A.; Mandarino, L. J.	2001	No healthy subjects or controls
Human masseter muscle fibers from the elderly express less neonatal Myosin than those of young adults	Cvetko, E.; Karen, P.; JanÃ¡Ä�ek, J.; KubÃ-novÃ¡, L.; Plasencia, A. L.; ErÅ¾en, I.	2012	Does not include male and female data
Exercise capacity and cytochrome oxidase activity in muscle mitochondria of COPD patients	D'Agostino, B.; Polverino, M.; Cirino, G.; Lombardi, A.; Grassi, B.; Sullo, N.; Santoriello, C.; Polverino, F.; Orlotti, D.; Matteis, M.; Rossi, F.	2010	Did not perform fiber type analysis
Effect of acute environmental hypoxia on protein metabolism in human skeletal muscle	D'Hulst, G.; Jamart, C.; Van Thienen, R.; Hespel, P.; Francaux, M.; Deldicque, L.	2013	Does not include males and females
Acute systemic insulin intolerance does not alter the response of the Akt/GSK-3 pathway to environmental hypoxia in human skeletal muscle	D'Hulst, G.; Sylow, L.; Hespel, P.; Deldicque, L.	2015	Does not include males and females
Decreased Satellite Cell Number and Function in Humans and Mice With Type 1 Diabetes Is the Result of Altered Notch Signaling	D'Souza, D. M.; Zhou, S.; Rebalka, I. A.; MacDonald, B.; Moradi, J.; Krause, M. P.; Al-Sajee, D.; Punthakee, Z.; Tarnopolsky, M. A.; Hawke, T. J.	2016	Did not perform fiber type analysis
Muscle metabolic remodelling patterns in Duchenne muscular dystrophy revealed by ultra-high-resolution mass spectrometry imaging	Dabaj, I.; Ferey, J.; Marguet, F.; Gilard, V.; Basset, C.; Bahri, Y.; Brehin, A. C.; Vanhulle, C.; Leturcq, F.; Marret, S.; LaquerriÃ¨re, A.; Schmitz-Afonso, I.; Afonso, C.; Bekri, S.; Tebani, A.	2021	Does not include males and females

Asymptomatic or minimally symptomatic hyperCKemia: histopathologic correlates	Dabby, R.; Sadeh, M.; Herman, O.; Berger, E.; Watemberg, N.; Hayek, S.; Jossiphov, J.; Nevo, Y.	2006	No healthy subjects or controls
Distinct lipid droplet characteristics and distribution unmask the apparent contradiction of the athlete's paradox	Daemen, S.; Gemmink, A.; Brouwers, B.; Meex, R. C. R.; Huntjens, P. R.; Schaart, G.; Moonen-Kornips, E.; JÃ¶rgensen, J.; Hoeks, J.; Schrauwen, P.; Hesselink, M. K. C.	2018	Does not report sex of subjects
Three-dimensional reconstruction of the human skeletal muscle mitochondrial network as a tool to assess mitochondrial content and structural organization	Dahl, R.; Larsen, S.; Dohlmann, T. L.; Qvortrup, K.; Helge, J. W.; Dela, F.; Prats, C.	2015	Does not include males and females
A transient antioxidant stress response accompanies the onset of disuse atrophy in human skeletal muscle	Dalla Libera, L.; Ravara, B.; Gobbo, V.; Tarricone, E.; Vitadello, M.; Biolo, G.; Vescovo, G.; Gorza, L.	2009	Does not include males and females
Skeletal muscle sarcoplasmic reticulum phenotype in myotonic dystrophy	Damiani, E.; Angelini, C.; Pelosi, M.; Sacchetto, R.; Bortoloso, E.; Margreth, A.	1996	Case studies
Reduced glycogen synthase activity in skeletal muscle from obese patients with and without type 2 (non-insulin-dependent) diabetes mellitus	Damsbo, P.; Vaag, A.; Hother-Nielsen, O.; Beck-Nielsen, H.	1991	Did not perform fiber type analysis
Apoptosis in idiopathic inflammatory myopathies with partial invasion; a role for CD8+ cytotoxic T cells?	Danielsson, O.; HÃ¤ggqvist, B.; GrÃ¶ntoft, L.; Ã–llinger, K.; Ernerudh, J.	2020	Does not report sex of subjects
Expression of apoptosis related proteins in normal and diseased muscle: a possible role for Bcl-2 in protection of striated muscle	Danielsson, O.; Nilsson, C.; Lindvall, B.; Ernerudh, J.	2009	Does not include male and female data
Clinical and ultrastructural observations in a kindred with normo-hyperkalaemic periodic paralysis	Danowski, T. S.; Fisher, E. R.; Vidalon, C.; Vester, J. W.; Thompson, R.; Nolan, S.; Stephan, T.; Sunder, J. H.	1975	Did not perform fiber type analysis
The Human Skeletal Muscle Transcriptome in Response to Oral Shilajit Supplementation	Das, A.; Datta, S.; Rhea, B.; Sinha, M.; Veeraragavan, M.; Gordillo, G.; Roy, S.	2016	Did not perform fiber type analysis
The response to stretch of human intercostal muscle spindles studied in vitro	Davis, J. N.	1975	No healthy subjects or controls
Muscle phosphocreatine repletion following single and repeated short sprint efforts	Dawson, B.; Goodman, C.; Lawrence, S.; Preen, D.; Polglaze, T.; Fitzsimons, M.; Fournier, P.	1997	Does not include males and females
Clinical and histological features of immune-mediated necrotising myopathy: A multi-centre South Australian cohort study	Day, J.; Otto, S.; Cash, K.; Limaye, V.	2020	No healthy subjects or controls
Differential DNA methylation with age displays both common and dynamic features across human tissues that are influenced by CpG landscape	Day, K.; Waite, L. L.; Thalacker-Mercer, A.; West, A.; Bamman, M. M.; Brooks, J. D.; Myers, R. M.; Absher, D.	2013	Did not perform fiber type analysis
Next-generation sequencing methylation profiling of subjects with obesity identifies novel gene changes	Day, S. E.; Coletta, R. L.; Kim, J. Y.; Campbell, L. E.; Benjamin, T. R.; Roust, L. R.; De Filippis, E. A.; Dinu, V.; Shaibi, G. Q.; Mandarino, L. J.; Coletta, D. K.	2016	Did not perform fiber type analysis
BDNF Val66Met Polymorphism, the Allele-Specific Analysis by qRT-PCR - a Novel Protocol	de Assis, G. G.; Hoffman, J. R.; Gasanov, E. V.	2020	Did not perform fiber type analysis

Fiber type-specific muscle glycogen sparing due to carbohydrate intake before and during exercise	De Bock, K.; Derave, W.; Ramaekers, M.; Richter, E. A.; Hespel, P.	2007	Does not include males and females
Exercise in the fasted state facilitates fibre type-specific intramyocellular lipid breakdown and stimulates glycogen resynthesis in humans	De Bock, K.; Richter, E. A.; Russell, A. P.; Eijnde, B. O.; Derave, W.; Ramaekers, M.; Koninckx, E.; LÃ©ger, B.; Verhaeghe, J.; Hespel, P.	2005	Does not include males and females
Histological organization is similar in human vocal muscle and tongue--a study of muscles and nerves	de Campos, D.; do Nascimento, P. S.; Ellwanger, J. H.; Gehlen, G.; Rodrigues, M. F.; Jotz, G. P.; Xavier, L. L.	2012	Autopsies
Rosiglitazone modifies the adipogenic potential of human muscle satellite cells	De Coppi, P.; Milan, G.; Scarda, A.; Boldrin, L.; Centobene, C.; Piccoli, M.; Pozzobon, M.; Pilon, C.; Pagano, C.; Gamba, P.; Vettor, R.	2006	Did not perform fiber type analysis
Insulin-resistant muscle is exercise resistant: evidence for reduced response of nuclear-encoded mitochondrial genes to exercise	De Filippis, E.; Alvarez, G.; Berria, R.; Cusi, K.; Everman, S.; Meyer, C.; Mandarino, L. J.	2008	Did not perform fiber type analysis
Weight increase and overweight are associated with DNA oxidative damage in skeletal muscle	de la Maza, M. P.; Olivares, D.; Hirsch, S.; Sierralta, W.; GattÃ¡s, V.; Barrera, G.; Bunout, D.; Leiva, L.; FernÃ¡ndez, M.	2006	Does not include males and females
In vivo and in vitro dysferlin expression in human muscle satellite cells	De Luna, N.; Gallardo, E.; Illa, I.	2004	Did not perform fiber type analysis
Role of thrombospondin 1 in macrophage inflammation in dysferlin myopathy	De Luna, N.; Gallardo, E.; Sonnet, C.; Chazaud, B.; Dominguez-Perles, R.; Suarez-Calvet, X.; Gherardi, R. K.; Illa, I.	2010	Did not perform fiber type analysis
Exercise reduces cellular stress related to skeletal muscle insulin resistance	de Matos, M. A.; Ottone Vde, O.; Duarte, T. C.; Sampaio, P. F.; Costa, K. B.; Fonseca, C. A.; Neves, M. P.; Schneider, S. M.; Moseley, P.; Coimbra, C. C.; MagalhÃ£es Fde, C.; Rocha-Vieira, E.; Amorim, F. T.	2014	Did not perform fiber type analysis
Morphological and physiological differences in the upper trapezius muscle in patients with work-related trapezius myalgia compared to healthy controls: A systematic review	De Meulemeester, K.; Calders, P.; De Pauw, R.; Grymonpon, I.; Govaerts, A.; Cagnie, B.	2017	Review (ex: systematic review/meta-analysis)
Muscle involvement in rheumatoid arthritis: an ultrastructural study	de Palma, L.; Chillemi, C.; Albanelli, S.; Rapali, S.; Bertoni-Freddari, C.	2000	No healthy subjects or controls
Free and total carnitine and acylcarnitine content of plasma, urine, liver and muscle of alcoholics	de Sousa, C.; Leung, N. W.; Chalmers, R. A.; Peters, T. J.	1988	Did not perform fiber type analysis
Short-term activation of peroxysome proliferator-activated receptor beta/delta increases fatty acid oxidation but does not restore insulin action in muscle cells from type 2 diabetic patients	Debard, C.; Cozzone, D.; Ricard, N.; Vouillarmet, J.; Disse, E.; Husson, B.; Laville, M.; Vidal, H.	2006	Did not perform fiber type analysis
Expression of key genes of fatty acid oxidation, including adiponectin receptors, in skeletal muscle of Type 2 diabetic patients	Debard, C.; Laville, M.; Berbe, V.; Loizon, E.; Guillet, C.; Morio-Liondore, B.; Boirie, Y.; Vidal, H.	2004	Did not perform fiber type analysis
Mitochondrial dysfunction in human skeletal muscle biopsies of lipid storage disorder	Debashree, B.; Kumar, M.; Keshava Prasad, T. S.; Natarajan, A.; Christopher, R.; Nalini,	2018	No healthy subjects or controls

	A.; Bindu, P. S.; Gayathri, N.; Srinivas Bharath, M. M.		
Correlation between plasma carnitine, muscle carnitine and glycogen levels in maintenance hemodialysis patients	Debska, SlizieÅ„; Kawecka, A.; Wojnarowski, K.; Prajs, J.; Malgorzewicz, S.; Kunicka, D.; Zdrojewski, Z.; Walysiak, SzydÅ‚owska; LipiÅ„ski, J.; Rutkowski, B.	2000	Did not perform fiber type analysis
Insulin resistance is a significant determinant of sarcopenia in advanced kidney disease	Deger, S. M.; Hewlett, J. R.; Gamboa, J.; Ellis, C. D.; Hung, A. M.; Siew, E. D.; Mamnungu, C.; Sha, F.; Bian, A.; Stewart, T. G.; Abumrad, N. N.; Ikizler, T. A.	2018	Did not perform fiber type analysis
The cell-specific expression of metalloproteinase-disintegrins (ADAMs) in inflammatory myopathies	Dehmel, T.; Janke, A.; Hartung, H. P.; Goebel, H. H.; Wiendl, H.; Kieseier, B. C.	2007	No healthy subjects or controls
Systemic inflammation correlates with increased expression of skeletal muscle ubiquitin but not uncoupling proteins in cancer cachexia	DeJong, C. H.; Busquets, S.; Moses, A. G.; Schrauwen, P.; Ross, J. A.; Argiles, J. M.; Fearon, K. C.	2005	No healthy subjects or controls
Uremic acidosis and intracellular buffering	Del Canale, S.; Fiaccadori, E.; Coffrini, E.; Vitali, P.; Ronda, N.; Antonucci, C.; Arduini, U.; Guariglia, A.	1986	Does not report sex of subjects
Skeletal muscle changes in chronic alcoholic patients. A conventional, histochemical, ultrastructural and morphometric study	Del Villar Negro, A.; Merino Angulo, J.; Rivera-Pomar, J. M.	1984	No healthy subjects or controls
IgG from amyotrophic lateral sclerosis affects tubular calcium channels of skeletal muscle	Delbono, O.; GarcÃa, J.; Appel, S. H.; Stefani, E.	1991	No healthy subjects or controls
Effects of resistance exercise with and without creatine supplementation on gene expression and cell signaling in human skeletal muscle	Deldicque, L.; Atherton, P.; Patel, R.; Theisen, D.; Nielens, H.; Rennie, M. J.; Francaux, M.	2008	Does not include male and female data
Increased IGF mRNA in human skeletal muscle after creatine supplementation	Deldicque, L.; Louis, M.; Theisen, D.; Nielens, H.; Dehoux, M.; Thissen, J. P.; Rennie, M. J.; Francaux, M.	2005	Does not include males and females
Early structural and functional signature of 3-day human skeletal muscle disuse using the dry immersion model	Demangel, R.; Treffel, L.; Py, G.; Brioche, T.; Pagano, A. F.; Bareille, M. P.; Beck, A.; Pessemesse, L.; Candau, R.; Gharib, C.; Chopard, A.; Millet, C.	2017	Does not include males and females
Human and Rodent Skeletal Muscles Express Angiotensin II Type 1 Receptors	Deminice, R.; Hyatt, H.; Yoshihara, T.; Ozdemir, M.; Nguyen, B.; Levine, S.; Powers, S.	2020	No healthy subjects or controls
Combined creatine and protein supplementation in conjunction with resistance training promotes muscle GLUT-4 content and glucose tolerance in humans	Derave, W.; Eijnde, B. O.; Verbessem, P.; Ramaekers, M.; Van Leemputte, M.; Richter, E. A.; Hespel, P.	2003	Does not include male and female data
Muscle atrophy and preferential loss of myosin in prolonged critically ill patients	Derde, S.; Hermans, G.; Derese, I.; GÃ¼iza, F.; HedstrÃ¶m, Y.; Wouters, P. J.; Bruyninckx, F.; D'Hoore, A.; Larsson, L.; Van den Berghe, G.; Vanhorebeek, I.	2012	Does not include male and female data
Neural factors account for strength decrements observed after short-term muscle unloading	Deschenes, M. R.; Giles, J. A.; McCoy, R. W.; Volek, J. S.; Gomez, A. L.; Kraemer, W. J.	2002	Does not include male and female data
Molecular motor MYO1C, acetyltransferase KAT6B and osteogenetic transcription factor	Desh, H.; Gray, S. L.; Horton, M. J.; Raoul, G.; Rowlerson, A. M.; Ferri, J.; Vieira, A. R.; Sciote, J. J.	2014	No cross-sectional area data

RUNX2 expression in human masseter muscle contributes to development of malocclusion			
Sodium channel and sodium pump in normal and pathological muscles from patients with myotonic muscular dystrophy and lower motor neuron impairment	Desnuelle, C.; Lombet, A.; Serratrice, G.; Lazdunski, M.	1982	No healthy subjects or controls
Muscle tissue adaptations of high-altitude natives to training in chronic hypoxia or acute normoxia	Desplanches, D.; Hoppeler, H.; TÃ¼scher, L.; Mayet, M. H.; Spielvogel, H.; Ferretti, G.; Kayser, B.; Leuenberger, M.; GrÃ¼nenfelder, A.; Favier, R.	1996	Does not include males and females
Training state and skeletal muscle autophagy in response to 36 h of fasting	Dethlefsen, M. M.; Bertholdt, L.; Gudiksen, A.; Stankiewicz, T.; Bangsbo, J.; van Hall, G.; Plomgaard, P.; Pilegaard, H.	2018	Does not include males and females
Evaluation of the mechanisms of sarcopenia in chronic inflammatory disease: protocol for a prospective cohort study	Dhaliwal, A.; Williams, F. R.; Quinlan, J. I.; Allen, S. L.; Greig, C.; Filer, A.; Raza, K.; Ghosh, S.; Lavery, G. G.; Newsome, P. N.; Choudhary, S.; Breen, L.; Armstrong, M. J.; Elsharkawy, A. M.; Lord, J. M.	2021	Did not perform fiber type analysis
Potential therapeutic targets for ALS: MIR206, MIR208b and MIR499 are modulated during disease progression in the skeletal muscle of patients	Di Pietro, L.; Baranzini, M.; Berardinelli, M. G.; Lattanzi, W.; Monforte, M.; Tasca, G.; Conte, A.; Logroscino, G.; Michetti, F.; Ricci, E.; Sabatelli, M.; Bernardini, C.	2017	No healthy subjects or controls
Lowered tumor necrosis factor receptors, but not increased insulin sensitivity, with infliximab	Di Rocco, P.; Manco, M.; Rosa, G.; Greco, A. V.; Mingrone, G.	2004	Did not perform fiber type analysis
Alterations in skeletal muscle repair in young adults with type 1 diabetes mellitus	Dial, A. G.; Grafham, G. K.; Monaco, C. M. F.; Voth, J.; Brandt, L.; Tarnopolsky, M. A.; Hawke, T. J.	2021	Did not perform fiber type analysis
Intra- and inter-individual metabolic profiling highlights carnitine and lysophosphatidylcholine pathways as key molecular defects in type 2 diabetes	Diamanti, K.; Cavalli, M.; Pan, G.; Pereira, M. J.; Kumar, C.; Skrtic, S.; Grabherr, M.; RisÃ©rus, U.; Eriksson, J. W.; Komorowski, J.; Wadelius, C.	2019	Did not perform fiber type analysis
The increase of pericyte population in human neuromuscular disorders supports their role in muscle regeneration in vivo	DÃaz-Manera, J.; Gallardo, E.; de Luna, N.; Navas, M.; Soria, L.; Garibaldi, M.; Rojas-GarcÃa, R.; Tonlorenzi, R.; Cossu, G.; Illa, I.	2012	Did not perform fiber type analysis
Rapamycin does not affect post-absorptive protein metabolism in human skeletal muscle	Dickinson, J. M.; Drummond, M. J.; Fry, C. S.; Gundermann, D. M.; Walker, D. K.; Timmerman, K. L.; Volpi, E.; Rasmussen, B. B.	2013	Did not perform fiber type analysis
Altered Energetics of Exercise Explain Risk of Rhabdomyolysis in Very Long-Chain Acyl-CoA Dehydrogenase Deficiency	Diekman, E. F.; Visser, G.; Schmitz, J. P.; Nievelstein, R. A.; de Sain-van der Velden, M.; Wardrop, M.; Van der Pol, W. L.; Houten, S. M.; van Riel, N. A.; Takken, T.; Jeneson, J. A.	2016	Does not include male and female data
Morphologic features of the myopathy associated with chronic renal failure	Diesel, W.; Emms, M.; Knight, B. K.; Noakes, T. D.; Swanepoel, C. R.; van Zyl Smit, R.; Kaschula, R. O.; Sinclair-Smith, C. C.	1993	Does not report sex of subjects
Conchotome and needle percutaneous biopsy of skeletal muscle	Dietrichson, P.; Coakley, J.; Smith, P. E.; Griffiths, R. D.; Helliwell, T. R.; Edwards, R. H.	1987	No healthy subjects or controls

Action potential-evoked calcium release is impaired in single skeletal muscle fibers from heart failure patients	DiFranco, M.; QuiÃ±onez, M.; Shieh, P.; Fonarow, G. C.; Cruz, D.; Deng, M. C.; Vergara, J. L.; Middlekauff, H. R.	2014	Does not include males and females
Muscle protein metabolism responds similarly to exogenous amino acids in healthy younger and older adults during NO-induced hyperemia	Dillon, E. L.; Casperson, S. L.; Durham, W. J.; Randolph, K. M.; Urban, R. J.; Volpi, E.; Ahmad, M.; Kinsky, M. P.; Sheffield-Moore, M.	2011	Did not perform fiber type analysis
Nuclear respiratory factor 1 and endurance exercise promote human telomere transcription	Diman, A.; Boros, J.; Poulain, F.; Rodriguez, J.; Purnelle, M.; Episkopou, H.; Bertrand, L.; Francaux, M.; Deldicque, L.; Decottignies, A.	2016	Does not include males and females
Impaired signaling for neuromuscular synaptic maintenance is a feature of Motor Neuron Disease	Ding, Q.; Kesavan, K.; Lee, K. M.; Wimberger, E.; Robertson, T.; Gill, M.; Power, D.; Chang, J.; Fard, A. T.; Mar, J. C.; Henderson, R. D.; Heggie, S.; McCombe, P. A.; Jeffree, R. L.; Colditz, M. J.; Hilliard, M. A.; Ng, D. C. H.; Steyn, F. J.; Phillips, W. D.; Wolvetang, E. J.; Ngo, S. T.; Noakes, P. G.	2022	Does not include male and female data
Chronological Age Does not Influence Ex-vivo Mitochondrial Respiration and Quality Control in Skeletal Muscle	Distefano, G.; Standley, R. A.; DubÃ©, J. J.; Carnero, E. A.; Ritov, V. B.; Stefanovic-Racic, M.; Toledo, F. G.; Piva, S. R.; Goodpaster, B. H.; Coen, P. M.	2017	Does not include male and female data
Measured muscle sodium content in biopsy specimens is a reflection of true intracellular content	Djurhuus, M. S.; Klitgaard, N. A.; Hyltoft Petersen, P.	2002	No healthy subjects or controls
Methodological aspects of measuring human skeletal muscle electrolyte content and ouabain binding capacity	Djurhuus, M. S.; Klitgaard, N. A.; Tveskov, C.; Madsen, K.; Guldager, B.; Jelnes, R.; Petersen, P. H.; Beck-Nielsen, H.	1998	Did not perform fiber type analysis
Reduced glucose transporter GLUT4 in skeletal muscle predicts insulin resistance in non-diabetic chronic heart failure patients independently of body composition	Doehner, W.; Gathercole, D.; Cicoira, M.; Krack, A.; Coats, A. J.; Camici, P. G.; Anker, S. D.	2010	Does not include males and females
The influence of aging and sex on skeletal muscle mass and strength	Doherty, T. J.	2001	Review (ex: systematic review/meta-analysis)
Statin Treatment Decreases Mitochondrial Respiration But Muscle Coenzyme Q10 Levels Are Unaltered: The LIFESTAT Study	Dohlmann, T. L.; Morville, T.; Kuhlman, A. B.; ChrÃ¸is, K. M.; Helge, J. W.; Dela, F.; Larsen, S.	2019	Did not perform fiber type analysis
Decreased expression of glucose transporter in muscle from insulin-resistant patients	Dohm, G. L.; Elton, C. W.; Friedman, J. E.; Pilch, P. F.; Pories, W. J.; Atkinson, S. M., Jr.; Caro, J. F.	1991	Did not perform fiber type analysis
IGF-I--stimulated glucose transport in human skeletal muscle and IGF-I resistance in obesity and NIDDM	Dohm, G. L.; Elton, C. W.; Raju, M. S.; Mooney, N. D.; DiMarchi, R.; Pories, W. J.; Flickinger, E. G.; Atkinson, S. M., Jr.; Caro, J. F.	1990	Did not perform fiber type analysis
Seasonal variation in lipoprotein lipase and plasma lipids in physically active, normal weight humans	Donahoo, W. T.; Jensen, D. R.; Shepard, T. Y.; Eckel, R. H.	2000	Did not perform fiber type analysis
Injury and Apoptosis in the Palatopharyngeal Muscle in Patients with Obstructive Sleep Apnea-Hypopnea Syndrome	Dong, J.; Niu, X.; Chen, X.	2020	Does not include males and females

Functional modulation of satellite cells in long-term denervated human laryngeal muscle	Donghui, C.; Shicai, C.; Wei, W.; Fei, L.; Jianjun, J.; Gang, C.; Hongliang, Z.	2010	No healthy subjects or controls
Gain and loss of extracellular molecules in sporadic inclusion body myositis and polymyositis--a proteomics-based study	Doppler, K.; Lindner, A.; SchÃ¼tz, W.; SchÃ¼tz, M.; Bornemann, A.	2012	Did not perform fiber type analysis
Role of xanthine oxidase in reperfusion injury of ischemic skeletal muscles in the pig and human	Dorion, D.; Zhong, A.; Chiu, C.; Forrest, C. R.; Boyd, B.; Pang, C. Y.	1993	No healthy subjects or controls
Signs of inflammation in both symptomatic and asymptomatic muscles from patients with polymyositis and dermatomyositis	Dorph, C.; Englund, P.; Nennesmo, I.; Lundberg, I. E.	2006	Did not perform fiber type analysis
A simple and rapid method for the determination of the concentrations of magnesium, sodium, potassium and sodium, potassium pumps in human skeletal muscle	DÃ¸rup, I.; Skajaa, K.; Clausen, T.	1988	Does not include male and female data
Reduced concentrations of potassium, magnesium, and sodium-potassium pumps in human skeletal muscle during treatment with diuretics	DÃ¸rup, I.; Skajaa, K.; Clausen, T.; Kjeldsen, K.	1988	No healthy subjects or controls
Atrophy and hypertrophy signalling of the quadriceps and diaphragm in COPD	Doucet, M.; DubÃ©, A.; Joanisse, D. R.; DebigarÃ©, R.; Michaud, A.; ParÃ©, MÃˆ; Vaillancourt, R.; FrÃ©chette, E.; Maltais, F.	2010	Did not perform fiber type analysis
Detection of enterovirus in human skeletal muscle from patients with chronic inflammatory muscle disease or fibromyalgia and healthy subjects	Douche-Aourik, F.; Berlier, W.; FÃ©asson, L.; Bourlet, T.; Harrath, R.; Omar, S.; Grattard, F.; Denis, C.; Pozzetto, B.	2003	Did not perform fiber type analysis
Statin therapy induces ultrastructural damage in skeletal muscle in patients without myalgia	Draeger, A.; Monastyrskaya, K.; Mohaupt, M.; Hoppeler, H.; Savolainen, H.; Allemann, C.; Babiychuk, E. B.	2006	No healthy subjects or controls
Low-Grade Systemic Inflammation Interferes with Anabolic and Catabolic Characteristics of the Aged Human Skeletal Muscle	Draganidis, D.; Jamurtas, A. Z.; Chondrogianni, N.; Mastorakos, G.; Jung, T.; Grune, T.; Papadopoulos, C.; Papanikolaou, K.; Papassotiriou, I.; Papaevgeniou, N.; Poulios, A.; Batrakoulis, A.; Deli, C. K.; Georgakouli, K.; Chatzinikolaou, A.; Karagounis, L. G.; Fatouros, I. G.	2021	Does not include males and females
Apelin and APJ regulation in adipose tissue and skeletal muscle of type 2 diabetic mice and humans	Dray, C.; Debard, C.; Jager, J.; Disse, E.; Daviaud, D.; Martin, P.; AttanÃ©, C.; Wanecq, E.; GuignÃ©, C.; Bost, F.; Tanti, J. F.; Laville, M.; Vidal, H.; Valet, P.; Castan-Laurell, I.	2010	Did not perform fiber type analysis
Effect of dietary macronutrient composition on AMPK and SIRT1 expression and activity in human skeletal muscle	Draznin, B.; Wang, C.; Adochio, R.; Leitner, J. W.; Cornier, M. A.	2012	Did not perform fiber type analysis
TGF-Î² Induction of miR-143/145 Is Associated to Exercise Response by Influencing Differentiation and Insulin Signaling Molecules in Human Skeletal Muscle	Dreher, S. I.; HÃ¶ckele, S.; Huypens, P.; Irmler, M.; Hoffmann, C.; Jeske, T.; Hastreiter, M.; Moller, A.; Birkenfeld, A. L.; HÃ¤ring, H. U.; Peter, A.; Beckers, J.; HrabÄ› de Angelis, M.; Weigert, C.	2021	Did not perform fiber type analysis

Effects of free fatty acids on glucose transport and IRS-1-associated phosphatidylinositol 3-kinase activity	Dresner, A.; Laurent, D.; Marcucci, M.; Griffin, M. E.; Dufour, S.; Cline, G. W.; Slezak, L. A.; Andersen, D. K.; Hundal, R. S.; Rothman, D. L.; Petersen, K. F.; Shulman, G. I.	1999	Does not include males and females
Pathology of skeletal muscle in fibromyalgia: a histo-immuno-chemical and ultrastructural study	Drewes, A. M.; Andreasen, A.; SchrÃ¸der, H. D.; HÃ¸gsaa, B.; Jennum, P.	1993	Does not include males and females
Alterations of skeletal muscle in chronic heart failure	Drexler, H.; Riede, U.; MÃ¼nzel, T.; KÃ¶nig, H.; Funke, E.; Just, H.	1992	Does not include male and female data
Skeletal muscle amino acid transporter expression is increased in young and older adults following resistance exercise	Drummond, M. J.; Fry, C. S.; Glynn, E. L.; Timmerman, K. L.; Dickinson, J. M.; Walker, D. K.; Gundermann, D. M.; Volpi, E.; Rasmussen, B. B.	2011	Did not perform fiber type analysis
Human muscle gene expression following resistance exercise and blood flow restriction	Drummond, M. J.; Fujita, S.; Abe, T.; Dreyer, H. C.; Volpi, E.; Rasmussen, B. B.	2008	Does not include males and females
Short-term bed rest increases TLR4 and IL-6 expression in skeletal muscle of older adults	Drummond, M. J.; Timmerman, K. L.; Markofski, M. M.; Walker, D. K.; Dickinson, J. M.; Jamaluddin, M.; Brasier, A. R.; Rasmussen, B. B.; Volpi, E.	2013	Did not perform fiber type analysis
Nutritional status in alcoholics with and without chronic skeletal muscle myopathy	Duane, P.; Peters, T. J.	1988	No healthy subjects or controls
Serum carnosinase activities in patients with alcoholic chronic skeletal muscle myopathy	Duane, P.; Peters, T. J.	1988	Does not include male and female data
Effects of acute lipid overload on skeletal muscle insulin resistance, metabolic flexibility, and mitochondrial performance	DubÃ©, J. J.; Coen, P. M.; DiStefano, G.; Chacon, A. C.; Helbling, N. L.; Desimone, M. E.; Stafanovic-Racic, M.; Hames, K. C.; Despines, A. A.; Toledo, F. G.; Goodpaster, B. H.	2014	Did not perform fiber type analysis
Endurance training, expression, and physiology of LDH, MCT1, and MCT4 in human skeletal muscle	Dubouchaud, H.; Butterfield, G. E.; Wolfel, E. E.; Bergman, B. C.; Brooks, G. A.	2000	Does not include males and females
Diagnostic value of markers of muscle degeneration in sporadic inclusion body myositis	Dubourg, O.; Wanschitz, J.; Maisonobe, T.; BÃ©hin, A.; Allenbach, Y.; Herson, S.; Benveniste, O.	2011	No healthy subjects or controls
Edematous myositis: a clinical presentation first suggesting dermatomyositis diagnosis	Duchesne, M.; Leonard-Louis, S.; Landon-Cardinal, O.; Anquetil, C.; Mariampillai, K.; Monzani, Q.; Benveniste, O.; Allenbach, Y.	2020	No healthy subjects or controls
Muscle fiber composition and blood ammonia levels after intense exercise in humans	Dudley, G. A.; Staron, R. S.; Murray, T. F.; Hagerman, F. C.; Luginbuhl, A.	1983	Does not include males and females
Activation of the Keap1/Nrf2 stress response pathway in autophagic vacuolar myopathies	Duleh, S.; Wang, X.; Komirenko, A.; Margeta, M.	2016	Did not perform fiber type analysis
Quercetin's effect on cycling efficiency and substrate utilization	Dumke, C. L.; Nieman, D. C.; Utter, A. C.; Rigby, M. D.; Quindry, J. C.; Triplett, N. T.; McAnulty, S. R.; McAnulty, L. S.	2009	Does not include males and females

In vivo analysis of Î³H2AX+ cells in skeletal muscle from aged and obese humans	Dungan, C. M.; Peck, B. D.; Walton, R. G.; Huang, Z.; Bamman, M. M.; Kern, P. A.; Peterson, C. A.	2020	Does not include male and female data
Cytochemical studies of lectin binding by diseased human muscle	Dunn, M. J.; Sewry, C. A.; Dubowitz, V.	1982	Did not perform fiber type analysis
Nogo provides a molecular marker for diagnosis of amyotrophic lateral sclerosis	Dupuis, L.; Gonzalez de Aguilar, J. L.; di Scala, F.; Rene, F.; de Tapia, M.; Pradat, P. F.; Lacomblez, L.; Seihlan, D.; Prinjha, R.; Walsh, F. S.; Meininger, V.; Loeffler, J. P.	2002	No healthy subjects or controls
Identification of novel Kirrel3 gene splice variants in adult human skeletal muscle	Durcan, P. J.; Conradie, J. D.; Van deVyver, M.; Myburgh, K. H.	2014	Does not include males and females
Fatiguing exercise reduces DNA binding activity of NF-kappaB in skeletal muscle nuclei	Durham, W. J.; Li, Y. P.; Gerken, E.; Farid, M.; Arbogast, S.; Wolfe, R. R.; Reid, M. B.	2004	Does not include males and females
Deconditioning fails to explain peripheral skeletal muscle alterations in men with chronic heart failure	Duscha, B. D.; Annex, B. H.; Green, H. J.; Pippen, A. M.; Kraus, W. E.	2002	Does not include male and female data
Skeletal muscle capillary density is related to anaerobic threshold and claudication in peripheral artery disease	Duscha, B. D.; Kraus, W. E.; Jones, W. S.; Robbins, J. L.; Piner, L. W.; Huffman, K. M.; Allen, J. D.; Annex, B. H.	2020	Did not perform fiber type analysis
Effects of carnosine on contractile apparatus CaÂ²â�º sensitivity and sarcoplasmic reticulum CaÂ²â�º release in human skeletal muscle fibers	Dutka, T. L.; Lamboley, C. R.; McKenna, M. J.; Murphy, R. M.; Lamb, G. D.	2012	Does not include males and females
Acute effects of taurine on sarcoplasmic reticulum Ca2+ accumulation and contractility in human type I and type II skeletal muscle fibers	Dutka, T. L.; Lamboley, C. R.; Murphy, R. M.; Lamb, G. D.	2014	Does not include male and female data
Mass cytometry reveals an impairment of B cell homeostasis in anti-synthetase syndrome	DzanguÃ©-Tchoupou, G.; Allenbach, Y.; PreuÃŸe, C.; Stenzel, W.; Benveniste, O.	2019	Did not perform fiber type analysis
The use of biopsies in the study of human skeletal muscle	Eberstein, A.; Goodgold, J.	1967	No healthy subjects or controls
Comprehensive proteome analysis of human skeletal muscle in cachexia and sarcopenia: a pilot study	Ebhardt, H. A.; Degen, S.; Tadini, V.; Schilb, A.; Johns, N.; Greig, C. A.; Fearon, K. C. H.; Aebersold, R.; Jacobi, C.	2017	Does not report sex of subjects
Muscular mitochondrial function in amyotrophic lateral sclerosis is progressively altered as the disease develops: a temporal study in man	Echaniz-Laguna, A.; Zoll, J.; Ponsot, E.; N'Guessan, B.; Tranchant, C.; Loeffler, J. P.; Lampert, E.	2006	Did not perform fiber type analysis
Mitochondrial respiratory chain function in skeletal muscle of ALS patients	Echaniz-Laguna, A.; Zoll, J.; Ribera, F.; Tranchant, C.; Warter, J. M.; Lonsdorfer, J.; Lampert, E.	2002	Did not perform fiber type analysis
Tissue-specific regulation of lipoprotein lipase by isoproterenol in normal-weight humans	Eckel, R. H.; Jensen, D. R.; Schlaepfer, I. R.; Yost, T. J.	1996	Did not perform fiber type analysis
Morphological differentiation of human muscle cocultured with mouse spinal cord	Ecob-Prince, M. S.; Brown, A. E.	1988	Did not perform fiber type analysis
Atypical persisting fibres in explants of human muscle cocultured with embryonic nerve cells	Ecob-Prince, M. S.; Cullen, M. J.	1988	No healthy subjects or controls

Human fiber size and enzymatic properties after 5 and 11 days of spaceflight	Edgerton, V. R.; Zhou, M. Y.; Ohira, Y.; Klitgaard, H.; Jiang, B.; Bell, G.; Harris, B.; Saltin, B.; Gollnick, P. D.; Roy, R. R.; et al.,	1995	Does not include male and female data
Effect of salbutamol on digoxin concentration in serum and skeletal muscle	Edner, M.; Jogestrand, T.	1989	Does not include males and females
Oral salbutamol decreases serum digoxin concentration	Edner, M.; Jogestrand, T.	1990	Does not include males and females
Effect of salbutamol on digoxin pharmacokinetics	Edner, M.; Jogestrand, T.; Dahlqvist, R.	1992	Does not include males and females
Selective atrophy of red muscle fibres in the quadriceps in long-standing knee-joint dysfunction. Injuries to the anterior cruciate ligament	EdstrÃ¶m, L.	1970	Does not include males and females
Differences in sizes of red and white muscle fibres in vastus lateralis of musculus quadriceps femoris of normal individuals and athletes. Relation to physical performance	EdstrÃ¶m, L.; Ekblom, B.	1972	Does not include males and females
Sulphur and phosphorus content in relation to fibre composition and atrophy of skeletal muscle in patients with Parkinson's disease	EdstrÃ¶m, L.; Gremski, W.; WrÃ³blewski, R.	1979	Does not include male and female data
Neurogenic effects on the palatopharyngeal muscle in patients with obstructive sleep apnoea: a muscle biopsy study	EdstrÃ¶m, L.; Larsson, H.; Larsson, L.	1992	Does not include males and females
Intracellular elemental composition of single muscle fibres in muscular dystrophy and dystrophia myotonica	EdstrÃ¶m, L.; Wroblewski, R.	1989	Did not perform fiber type analysis
AMP kinase expression and activity in human skeletal muscle: effects of immobilization, retraining, and creatine supplementation	Eijnde, B. O.; Derave, W.; Wojtaszewski, J. F.; Richter, E. A.; Hespel, P.	2005	Did not perform fiber type analysis
No effects of oral ribose supplementation on repeated maximal exercise and de novo ATP resynthesis	Eijnde, B. O.; Van Leemputte, M.; Brouns, F.; Van Der Vusse, G. J.; Labarque, V.; Ramaekers, M.; Van Schuylenberg, R.; Verbessem, P.; Wijnen, H.; Hespel, P.	2001	Does not include males and females
Human skeletal muscle function and metabolism during intense exercise at high O2 and N2 pressures	Eiken, O.; Hesser, C. M.; Lind, F.; Thorsson, A.; Tesch, P. A.	1987	Does not include males and females
Altered mitochondrial apparent affinity for ADP and impaired function of mitochondrial creatine kinase in gluteus medius of patients with hip osteoarthritis	Eimre, M.; Puhke, R.; Alev, K.; Seppet, E.; Sikkut, A.; Peet, N.; Kadaja, L.; Lenzner, A.; Haviko, T.; Seene, T.; Saks, V. A.; Seppet, E. K.	2006	Does not include male and female data
Monitoring molecular changes induced by ischemia/reperfusion in human free muscle flap tissue samples	Eisenhardt, S. U.; Schmidt, Y.; Karaxha, G.; Iblher, N.; Penna, V.; Torio-Padron, N.; Stark, G. B.; Bannasch, H.	2012	No healthy subjects or controls
The effect of (steroid) immunosuppression on skeletal muscle glycogen metabolism in patients after kidney transplantation	Ekstrand, A.; Schalin-JÃ¤ntti, C.; LÃ¶fman, M.; Parkkonen, M.; WidÃ©n, E.; Franssila-Kallunki, A.; Saloranta, C.; Koivisto, V.; Groop, L.	1996	Did not perform fiber type analysis

Patients experiencing statin-induced myalgia exhibit a unique program of skeletal muscle gene expression following statin re-challenge	Elam, M. B.; Majumdar, G.; Mozhui, K.; Gerling, I. C.; Vera, S. R.; Fish-Trotter, H.; Williams, R. W.; Childress, R. D.; Raghow, R.	2017	Did not perform fiber type analysis
Biochemical and morphometric properties of mitochondrial populations in human muscle fibres	Elander, A.; SjÃ¶strÃ¶m, M.; Lundgren, F.; ScherstÃ©n, T.; Bylund-Fellenius, A. C.	1985	Does not include males and females
Increased lipid availability for three days reduces whole body glucose uptake, impairs muscle mitochondrial function and initiates opposing effects on PGC-1Î± promoter methylation in healthy subjects	Eldor, R.; Norton, L.; Fourcaudot, M.; Galindo, C.; DeFronzo, R. A.; Abdul-Ghani, M.	2017	Did not perform fiber type analysis
Alterations in muscle proteome of patients diagnosed with amyotrophic lateral sclerosis	Elf, K.; Shevchenko, G.; Nygren, I.; Larsson, L.; Bergquist, J.; Askmark, H.; Artemenko, K.	2014	Did not perform fiber type analysis
Beta-adrenoceptors and human skeletal muscle characterisation of receptor subtype and effect of age	Elfellah, M. S.; Dalling, R.; Kantola, I. M.; Reid, J. L.	1989	Did not perform fiber type analysis
Differential isoform expression of SERCA and myosin heavy chain in hypopharyngeal muscles	Elrabie Ahmed, M.; Bando, H.; Fuse, S.; Mostafa Abdelfattah, H.; Elrabie Ahmed, M.; Abdel-Kader Ahmed, M.; Tsujikawa, T.; Hisa, Y.	2019	Does not include males and females
The determination of lactate dehydrogenase isoenzymes in normal human muscle and other tissues	Emery, A. E.	1967	Did not perform fiber type analysis
Microvascular changes in early and advanced dermatomyositis: a quantitative study	Emslie-Smith, A. M.; Engel, A. G.	1990	Did not perform fiber type analysis
Autophagic glycogenosis of late onset with mitochondrial abnormalities: light and electron microscopic observations	Engel, A. G.; Dale, A. J.	1968	Case studies
Nemaline (Z-disc) myopathy	Engel, A. G.; Gomez, M. R.	1967	Only in children (0-17 years)
Selective and nonselective susceptibility of muscle fiber types. A new approach to human neuromuscular diseases	Engel, W. K.	1970	Does not report sex of subjects
Altered glutamate metabolism is associated with reduced muscle glutathione levels in patients with emphysema	Engelen, M. P.; Schols, A. M.; Does, J. D.; Deutz, N. E.; Wouters, E. F.	2000	Did not perform fiber type analysis
Exercise-induced lactate increase in relation to muscle substrates in patients with chronic obstructive pulmonary disease	Engelen, M. P.; Schols, A. M.; Does, J. D.; Gosker, H. R.; Deutz, N. E.; Wouters, E. F.	2000	Does not include male and female data
Effects of exercise on amino acid metabolism in patients with chronic obstructive pulmonary disease	Engelen, M. P.; Wouters, E. F.; Deutz, N. E.; Does, J. D.; Schols, A. M.	2001	Did not perform fiber type analysis
Interleukin-1alpha expression in capillaries and major histocompatibility complex class I expression in type II muscle fibers from polymyositis and dermatomyositis patients: important pathogenic features independent of inflammatory cell clusters in muscle tissue	Englund, P.; Nennesmo, I.; Klareskog, L.; Lundberg, I. E.	2002	Does not include male and female data

Charcot-Marie-Tooth disease--muscle biopsy findings in relation to neurophysiology	Ericson, U.; Ansved, T.; Borg, K.	1998	Does not include male and female data
Potassium in skeletal muscle in untreated primary hypertension and in chronic renal failure, studied by X-ray fluorescence technique	Ericsson, F.	1984	Did not perform fiber type analysis
Effect of nifedipine on cellular electrolytes in fourteen patients with untreated primary hypertension	Ericsson, F.	1985	Did not perform fiber type analysis
Effect of digoxin upon intracellular potassium in man	Ericsson, F.; Carlmark, B.; Jogestrand, T.; Sundqvist, K.	1981	Did not perform fiber type analysis
Hypertrophic muscle fibers with fissures in power-lifters; fiber splitting or defect regeneration?	Eriksson, A.; LindstrÃ¶m, M.; Carlsson, L.; Thornell, L. E.	2006	Does not report sex of subjects
Simultaneous analysis of expression of the three myotonic dystrophy locus genes in adult skeletal muscle samples: the CTG expansion correlates inversely with DMPK and 59 expression levels, but not DMAHP levels	Eriksson, M.; Ansved, T.; EdstrÃ¶m, L.; Anvret, M.; Carey, N.	1999	Did not perform fiber type analysis
Decreased DMPK transcript levels in myotonic dystrophy 1 type IIA muscle fibers	Eriksson, M.; Hedberg, B.; Carey, N.; Ansved, T.	2001	Does not report sex of subjects
The skeletal muscle VEGF mRNA response to acute exercise in patients with chronic heart failure	Esposito, F.; Mathieu-Costello, O.; Entin, P. L.; Wagner, P. D.; Richardson, R. S.	2010	Does not include males and females
Limited maximal exercise capacity in patients with chronic heart failure: partitioning the contributors	Esposito, F.; Mathieu-Costello, O.; Shabetai, R.; Wagner, P. D.; Richardson, R. S.	2010	Does not include males and females
Acute and chronic exercise in patients with heart failure with reduced ejection fraction: evidence of structural and functional plasticity and intact angiogenic signalling in skeletal muscle	Esposito, F.; Mathieu-Costello, O.; Wagner, P. D.; Richardson, R. S.	2018	Does not report sex of subjects
Metabolic characteristics of fibre types in human skeletal muscle	EssÃ©n, B.; Jansson, E.; Henriksson, J.; Taylor, A. W.; Saltin, B.	1975	Does not include male and female data
Short-term starvation decreases skeletal muscle protein synthesis rate in man	EssÃ©n, P.; McNurlan, M. A.; Wernerman, J.; Milne, E.; Vinnars, E.; Garlick, P. J.	1992	Does not include males and females
Effect of exercise on concentrations of free amino acids in pools of type I and type II fibres in human muscle with reduced glycogen stores	EssÃ©n-Gustavsson, B.; Blomstrand, E.	2002	Does not include males and females
Enzyme levels in pools of microdissected human muscle fibres of identified type. Adaptive response to exercise	EssÃ©n-Gustavsson, B.; Henriksson, J.	1984	Does not include males and females
Effects of acetate infusion and hyperoxia on muscle substrate phosphorylation after onset of moderate exercise	Evans, M. K.; Savasi, I.; Heigenhauser, G. J.; Spriet, L. L.	2001	Does not include males and females
Investigation of MGF mRNA expression in patients with amyotrophic lateral sclerosis using parallel in vivo and in vitro approaches	Evans, R. M.; Harridge, S. D.; Velloso, C. P.; Yang, S. Y.; Goldspink, G.; Orrell, R. W.	2010	Did not perform fiber type analysis

The gene expression and activity of calpains and the muscle wasting-associated ubiquitin ligases, atrogin-1 and MuRF1, are not altered in patients with primary hyperparathyroidism	Evenson, A.; Mitchell, J.; Wei, W.; Poylin, V.; Parangi, S.; Hasselgren, P. O.	2006	No healthy subjects or controls
Association between specific adipose tissue CD4+ T-cell populations and insulin resistance in obese individuals	Fabbrini, E.; Cella, M.; McCartney, S. A.; Fuchs, A.; Abumrad, N. A.; Pietka, T. A.; Chen, Z.; Finck, B. N.; Han, D. H.; Magkos, F.; Conte, C.; Bradley, D.; Fraterrigo, G.; Eagon, J. C.; Patterson, B. W.; Colonna, M.; Klein, S.	2013	Did not perform fiber type analysis
Increase in decorin and biglycan in Duchenne Muscular Dystrophy: role of fibroblasts as cell source of these proteoglycans in the disease	Fadic, R.; Mezzano, V.; Alvarez, K.; Cabrera, D.; Holmgren, J.; Brandan, E.	2006	Does not include males and females
A microcalorimetric study of the sodium-potassium-pump and thermogenesis in human skeletal muscle	Fagher, B.; SjÃ¶gren, A.; Monti, M.	1987	Did not perform fiber type analysis
Physiological abnormalities of skeletal muscle in dialysis patients	Fahal, I. H.; Bell, G. M.; Bone, J. M.; Edwards, R. H.	1997	Does not include males and females
Significant molecular and systemic adaptations after repeated sprint training in hypoxia	Faiss, R.; LÃ©ger, B.; Vesin, J. M.; Fournier, P. E.; Eggel, Y.; DÃ©riaz, O.; Millet, G. P.	2013	Does not include males and females
Co-expression of SERCA isoforms, phospholamban and sarcolipin in human skeletal muscle fibers	Fajardo, V. A.; Bombardier, E.; Vigna, C.; Devji, T.; Bloemberg, D.; Gamu, D.; Gramolini, A. O.; Quadrilatero, J.; Tupling, A. R.	2013	Does not include males and females
Carbohydrate and lipid metabolism of skeletal muscle in type 2 diabetic patients	Falholt, K.; Jensen, I.; Lindkaer Jensen, S.; Mortensen, H.; VÃ¸lund, A.; Heding, L. G.; Noerskov Petersen, P.; Falholt, W.	1988	Did not perform fiber type analysis
Missense mutations in the beta-myosin heavy-chain gene cause central core disease in hypertrophic cardiomyopathy	Fananapazir, L.; Dalakas, M. C.; Cyran, F.; Cohn, G.; Epstein, N. D.	1993	Did not perform fiber type analysis
Loss of calpain-3 autocatalytic activity in LGMD2A patients with normal protein expression	Fanin, M.; Nascimbeni, A. C.; Fulizio, L.; Trevisan, C. P.; Meznaric-Petrusa, M.; Angelini, C.	2003	Did not perform fiber type analysis
Tissue-specific regulation of lipoprotein lipase activity by insulin/glucose in normal-weight humans	Farese, R. V., Jr.; Yost, T. J.; Eckel, R. H.	1991	Did not perform fiber type analysis
Morphological study on the origin of the semitendinosus muscle in the long head of biceps femoris	FarfÃ¡n, E.; Rojas, S.; OlivÃ©-VilÃ¡s, R.; RodrÃguez-Baeza, A.	2021	Autopsies
Whey protein supplementation accelerates satellite cell proliferation during recovery from eccentric exercise	Farup, J.; Rahbek, S. K.; Knudsen, I. S.; de Paoli, F.; Mackey, A. L.; Vissing, K.	2014	Does not include males and females
Influence of exercise contraction mode and protein supplementation on human skeletal muscle satellite cell content and muscle fiber growth	Farup, J.; Rahbek, S. K.; Riis, S.; Vendelbo, M. H.; Paoli, Fd; Vissing, K.	2014	Does not include males and females
Muscle pathology in ankylosing spondylitis: clinical, enzymatic, electromyographic and histologic correlation	Faus-Riera, S.; MartÃnez-Pardo, S.; Blanch-RubiÃ³, J.; Benito-Ruiz, P.; DurÃ³-Pujol, J. C.; Corominas-Torres, J. M.	1991	Does not report sex of subjects

The Muscle Metabolome Differs between Healthy and Frail Older Adults	Fazelzadeh, P.; Hangelbroek, R. W.; Tieland, M.; de Groot, L. C.; Verdijk, L. B.; van Loon, L. J.; Smilde, A. K.; Alves, R. D.; Vervoort, J.; MÃ¼ller, M.; van Duynhoven, J. P.; Boekschoten, M. V.	2016	Did not perform fiber type analysis
Exercise training decreases activation of the mitochondrial fission protein dynamin-related protein-1 in insulin-resistant human skeletal muscle	Fealy, C. E.; Mulya, A.; Lai, N.; Kirwan, J. P.	2014	Did not perform fiber type analysis
Overexpression of IFN-induced protein 10 and its receptor CXCR3 in myasthenia gravis	Feferman, T.; Maiti, P. K.; Berrih-Aknin, S.; Bismuth, J.; Bidault, J.; Fuchs, S.; Souroujon, M. C.	2005	Did not perform fiber type analysis
Crura ultrastructural alterations in patients with hiatal hernia: a pilot study	Fei, L.; del Genio, G.; Brusciano, L.; Esposito, V.; Cuttitta, D.; Pizza, F.; Rossetti, G.; Trapani, V.; Filippone, G.; Francesco, M.; del Genio, A.	2007	Did not perform fiber type analysis
Hiatal hernia recurrence: surgical complication or disease? Electron microscope findings of the diaphragmatic pillars	Fei, L.; del Genio, G.; Rossetti, G.; Sampaolo, S.; Moccia, F.; Trapani, V.; Cimmino, M.; del Genio, A.	2009	Did not perform fiber type analysis
Cyclooxygenase 2 contributes to bradykinin-induced microvascular responses inÂ peripheral arterioles after cardiopulmonary bypass	Feng, J.; Anderson, K.; Liu, Y.; Singh, A. K.; Ehsan, A.; Sellke, F. W.	2017	No healthy subjects or controls
Muscle oxygen uptake and energy turnover during dynamic exercise at different contraction frequencies in humans	Ferguson, R. A.; Ball, D.; Krustrup, P.; Aagaard, P.; Kjaer, M.; Sargeant, A. J.; Hellsten, Y.; Bangsbo, J.	2001	Does not include males and females
Postexercise carbohydrate-protein supplementation improves subsequent exercise performance and intracellular signaling for protein synthesis	Ferguson-Stegall, L.; McCleave, E. L.; Ding, Z.; Doerner, P. G., 3rd; Wang, B.; Liao, Y. H.; Kammer, L.; Liu, Y.; Hwang, J.; Dessard, B. M.; Ivy, J. L.	2011	Did not perform fiber type analysis
Muscle fibre type and obstructive sleep apnea	Ferini-Strambi, L. J.; Smirne, S.; Moz, U.; Sferrazza, B.; Iannaccone, S.	1998	Full text not available
Does oxidative stress modulate limb muscle atrophy in severe COPD patients?	Fermoselle, C.; Rabinovich, R.; AusÃn, P.; Puig-Vilanova, E.; Coronell, C.; Sanchez, F.; Roca, J.; Gea, J.; Barreiro, E.	2012	Does not include males and females
Skeletal muscle acetylcholinesterase molecular forms in amyotrophic lateral sclerosis	Fernandez, H. L.; Stiles, J. R.; Donoso, J. A.	1986	No healthy subjects or controls
Relationship between muscle water and glycogen recovery after prolonged exercise in the heat in humans	FernÃ¡ndez-ElÃas, V. E.; Ortega, J. F.; Nelson, R. K.; Mora-Rodriguez, R.	2015	Does not include males and females
Reduced efficiency, but increased fat oxidation, in mitochondria from human skeletal muscle after 24-h ultraendurance exercise	FernstrÃ¶m, M.; Bakkman, L.; Tonkonogi, M.; Shabalina, I. G.; Rozhdestvenskaya, Z.; Mattsson, C. M.; Enqvist, J. K.; Ekblom, B.; Sahlin, K.	2007	Does not include males and females
Effects of acute and chronic endurance exercise on mitochondrial uncoupling in human skeletal muscle	FernstrÃ¶m, M.; Tonkonogi, M.; Sahlin, K.	2004	Did not perform fiber type analysis
Apoptosis and insulin resistance in liver and peripheral tissues of morbidly obese patients is associated with different stages of non-alcoholic fatty liver disease	Ferreira, D. M.; Castro, R. E.; Machado, M. V.; Evangelista, T.; Silvestre, A.; Costa, A.; Coutinho, J.; Carepa, F.; Cortez-Pinto, H.; Rodrigues, C. M.	2011	No healthy subjects or controls

The interplay of central and peripheral factors in limiting maximal O2 consumption in man after prolonged bed rest	Ferretti, G.; Antonutto, G.; Denis, C.; Hoppeler, H.; Minetti, A. E.; Narici, M. V.; Desplanches, D.	1997	Does not include males and females
Association of soluble guanylate cyclase with the sarcolemma of mammalian skeletal muscle fibers	Feussner, M.; Richter, H.; Baum, O.; Gossrau, R.	2001	Does not report sex of subjects
Hypophosphatemia in course of chronic obstructive pulmonary disease. Prevalence, mechanisms, and relationships with skeletal muscle phosphorus content	Fiaccadori, E.; Coffrini, E.; Ronda, N.; Vezzani, A.; Cacciani, G.; Fracchia, C.; Rampulla, C.; Borghetti, A.	1990	No healthy subjects or controls
Intracellular acid-base and electrolyte metabolism in skeletal muscle of patients with chronic obstructive lung disease and acute respiratory failure	Fiaccadori, E.; Del Canale, S.; Arduini, U.; Antonucci, C.; Coffrini, E.; Vitali, P.; Meley, R.; Guariglia, A.	1986	Did not perform fiber type analysis
Skeletal muscle energetics, acid-base equilibrium and lactate metabolism in patients with severe hypercapnia and hypoxemia	Fiaccadori, E.; Del Canale, S.; Vitali, P.; Coffrini, E.; Ronda, N.; Guariglia, A.	1987	Did not perform fiber type analysis
CD24, a signal-transducing molecule expressed on human B lymphocytes, is a marker for human regenerating muscle	Figarella-Branger, D.; Moreau, H.; Pellissier, J. F.; Bianco, N.; Rougon, G.	1993	Did not perform fiber type analysis
Studies on apoptosis and fibrosis in skeletal musculature: a comparison of heart failure patients with and without cardiac cachexia	Filippatos, G. S.; Kanatselos, C.; Manolatos, D. D.; Vougas, B.; Sideris, A.; Kardara, D.; Anker, S. D.; Kardaras, F.; Uhal, B.	2003	Did not perform fiber type analysis
Expression of anti-inflammatory macrophage genes within skeletal muscle correlates with insulin sensitivity in human obesity and type 2 diabetes	Fink, L. N.; Oberbach, A.; Costford, S. R.; Chan, K. L.; Sams, A.; BlÃ¼her, M.; Klip, A.	2013	Did not perform fiber type analysis
Physiological properties of skinned fibres from normal and dystrophic (Duchenne) human muscle activated by Ca2+ and Sr2+	Fink, R. H.; Stephenson, D. G.; Williams, D. A.	1990	Does not report sex of subjects
Pioglitazone corrects dysregulation of skeletal muscle mitochondrial proteins involved in ATP synthesis in type 2 diabetes	Fiorentino, T. V.; Monroy, A.; Kamath, S.; Sotero, R.; Cas, M. D.; Daniele, G.; Chavez, A. O.; Abdul-Ghani, M.; Hribal, M. L.; Sesti, G.; Tripathy, D.; DeFronzo, R. A.; Folli, F.	2021	Did not perform fiber type analysis
Sequestosome-1 (p62) expression reveals chaperone-assisted selective autophagy in immune-mediated necrotizing myopathies	Fischer, N.; PreuÃŸe, C.; Radke, J.; Pehl, D.; Allenbach, Y.; Schneider, U.; Feist, E.; von Casteleyn, V.; Hahn, K.; Ruck, T.; Meuth, S. G.; Goebel, H. H.; Graf, R.; Mammen, A.; Benveniste, O.; Stenzel, W.	2020	No healthy subjects or controls
Disruption of myofibrils in the skeletal muscle of psychotic patients	Fischman, D. A.; Meltzer, H. Y.; Poppei, R. W.	1970	Did not perform fiber type analysis
Levels of adenylate deaminase, adenylate kinase, and creatine kinase in frozen human muscle biopsy specimens relative to type 1/type 2 fiber distribution: evidence for a carrier state of myoadenylate deaminase deficiency	Fishbein, W. N.; Armbrustmacher, V. W.; Griffin, J. L.; Davis, J. I.; Foster, W. D.	1984	No healthy subjects or controls
The deleterious effects of bed rest on human skeletal muscle fibers are exacerbated by hypercortisolemia and ameliorated by dietary supplementation	Fitts, R. H.; Romatowski, J. G.; Peters, J. R.; Paddon-Jones, D.; Wolfe, R. R.; Ferrando, A. A.	2007	Does not include males and females

Effects of a nucleoside reverse transcriptase inhibitor, stavudine, on glucose disposal and mitochondrial function in muscle of healthy adults	Fleischman, A.; Johnsen, S.; Systrom, D. M.; Hrovat, M.; Farrar, C. T.; Frontera, W.; Fitch, K.; Thomas, B. J.; Torriani, M.; CÃ´tÃ©, H. C.; Grinspoon, S. K.	2007	Did not perform fiber type analysis
Dysferlin interacts with calsequestrin-1, myomesin-2 and dynein in human skeletal muscle	Flix, B.; de la Torre, C.; Castillo, J.; Casal, C.; Illa, I.; Gallardo, E.	2013	Does not report sex of subjects
Muscle biopsy, macro EMG, and clinical characteristics in patients with schizophrenia	Flyckt, L.; Borg, J.; Borg, K.; Ansved, T.; Edman, G.; Bjerkenstedt, L.; Wiesel, F. A.	2000	No cross-sectional area data
Neuromuscular and psychomotor abnormalities in patients with schizophrenia and their first-degree relatives	Flyckt, L.; Wiesel, F. A.; Borg, J.; Edman, G.; Ansved, T.; Sydow, O.; Borg, K.	2000	No cross-sectional area data
Muscle fiber composition and respiratory capacity in triathletes	Flynn, M. G.; Costill, D. L.; Kirwan, J. P.; Fink, W. J.; Dengel, D. R.	1987	Does not include males and females
Immunohistochemical changes in the expression of HSP27 in exercised human vastus lateralis muscle	Folkesson, M.; Mackey, A. L.; Holm, L.; Kjaer, M.; Paulsen, G.; Raastad, T.; Henriksson, J.; Kadi, F.	2008	Does not include males and females
The expression of heat shock protein in human skeletal muscle: effects of muscle fibre phenotype and training background	Folkesson, M.; Mackey, A. L.; Langberg, H.; Oskarsson, E.; Piehl-Aulin, K.; Henriksson, J.; Kadi, F.	2013	Does not include male and female data
Mitochondrial H(+)-ATP synthase in human skeletal muscle: contribution to dyslipidaemia and insulin resistance	Formentini, L.; Ryan, A. J.; GÃ¡lvez-Santisteban, M.; Carter, L.; Taub, P.; Lapek, J. D., Jr.; Gonzalez, D. J.; Villarreal, F.; Ciaraldi, T. P.; Cuezva, J. M.; Henry, R. R.	2017	Did not perform fiber type analysis
Evidence of apoptosis via TUNEL staining in muscle biopsy from patients with mitochondrial encephaloneuromyopathies	Formichi, P.; Battisti, C.; Bianchi, S.; Cardaioli, E.; Federico, A.	2003	Did not perform fiber type analysis
Vitamin E prevents neutrophil accumulation and attenuates tissue damage in ischemic-reperfused human skeletal muscle	Formigli, L.; Ibba Manneschi, L.; Tani, A.; Gandini, E.; Adembri, C.; Pratesi, C.; Novelli, G. P.; Zecchi Orlandini, S.	1997	Does not include males and females
Neutrophils as mediators of human skeletal muscle ischemia-reperfusion syndrome	Formigli, L.; Lombardo, L. D.; Adembri, C.; Brunelleschi, S.; Ferrari, E.; Novelli, G. P.	1992	Does not include males and females
Skeletal muscle enzyme activity, fiber composition and VO2 max in relation to distance running performance	Foster, C.; Costill, D. L.; Daniels, J. T.; Fink, W. J.	1978	Does not include males and females
Differential signalling mechanisms predisposing primary human skeletal muscle cells to altered proliferation and differentiation: roles of IGF-I and TNFalpha	Foulstone, E. J.; Huser, C.; Crown, A. L.; Holly, J. M.; Stewart, C. E.	2004	No healthy subjects or controls
Adaptations of the IGF system during malignancy: human skeletal muscle versus the systemic environment	Foulstone, E. J.; Savage, P. B.; Crown, A. L.; Holly, J. M.; Stewart, C. E.	2003	Did not perform fiber type analysis
Protein homeostasis in LGMDR9 (LGMD2I) - The role of ubiquitin-proteasome and autophagy-lysosomal system	Franekova, V.; Storjord, H. I.; Leivseth, G.; Nilssen, Ã˜	2021	Did not perform fiber type analysis
Isolation and Expansion of Muscle Precursor Cells from Human Skeletal Muscle Biopsies	Franzin, C.; Piccoli, M.; Urbani, L.; Biz, C.; Gamba, P.; De Coppi, P.; Pozzobon, M.	2016	Does not report sex of subjects

Fatigue depresses maximal in vitro skeletal muscle Na(+)-K(+)-ATPase activity in untrained and trained individuals	Fraser, S. F.; Li, J. L.; Carey, M. F.; Wang, X. N.; Sangkabutra, T.; Sostaric, S.; Selig, S. E.; Kjeldsen, K.; McKenna, M. J.	2002	Does not include males and females
Measurement of Na+, K+-ATPase activity in human skeletal muscle	Fraser, S. F.; McKenna, M. J.	1998	Does not include males and females
Cardiac troponin T and creatine kinase MB are not increased in exterior oblique muscle of patients with renal failure	Fredericks, S.; Murray, J. F.; Bewick, M.; Chang, R.; Collinson, P. O.; Carter, N. D.; Holt, D. W.	2001	Does not report sex of subjects
Derangements in mitochondrial metabolism in intercostal and leg muscle of critically ill patients with sepsis-induced multiple organ failure	Fredriksson, K.; Hammarqvist, F.; StrigÃ¥rd, K.; Hultenby, K.; Ljungqvist, O.; Wernerman, J.; Rooyackers, O.	2006	Did not perform fiber type analysis
Dysregulation of mitochondrial dynamics and the muscle transcriptome in ICU patients suffering from sepsis induced multiple organ failure	Fredriksson, K.; TjÃ¤der, I.; Keller, P.; Petrovic, N.; Ahlman, B.; SchÃ©ele, C.; Wernerman, J.; Timmons, J. A.; Rooyackers, O.	2008	Did not perform fiber type analysis
Long-Term Endurance Exercise in Humans Stimulates Cell Fusion of Myoblasts along with Fusogenic Endogenous Retroviral Genes In Vivo	Frese, S.; Ruebner, M.; Suhr, F.; Konou, T. M.; Tappe, K. A.; Toigo, M.; Jung, H. H.; Henke, C.; Steigleder, R.; Strissel, P. L.; Huebner, H.; Beckmann, M. W.; van der Keylen, P.; Schoser, B.; Schiffer, T.; Frese, L.; Bloch, W.; Strick, R.	2015	Does not include males and females
Microarray analysis for delineating the gene expression in biopsies of gastrocnemius muscle of patients with chronic critical limb ischaemia compared with non-ischaemic controls	Freund, D.; Brilloff, S.; Ghazy, T.; Kirschner, S.; GÃ¤bel, G.; Hinterseher, I.; Weiss, N.; Mahlmann, A.	2018	Did not perform fiber type analysis
Impaired insulin-stimulated muscle glycogen synthase activation in vivo in man is related to low fasting glycogen synthase phosphatase activity	Freymond, D.; Bogardus, C.; Okubo, M.; Stone, K.; Mott, D.	1988	Did not perform fiber type analysis
Histological indications of a progressive snorers disease in an upper airway muscle	Friberg, D.; Ansved, T.; Borg, K.; Carlsson-Nordlander, B.; Larsson, H.; Svanborg, E.	1998	Does not include males and females
Restoration of insulin responsiveness in skeletal muscle of morbidly obese patients after weight loss. Effect on muscle glucose transport and glucose transporter GLUT4	Friedman, J. E.; Dohm, G. L.; Leggett-Frazier, N.; Elton, C. W.; Tapscott, E. B.; Pories, W. P.; Caro, J. F.	1992	Did not perform fiber type analysis
Gene expression changes in vastus lateralis muscle after different strength training regimes during rehabilitation following anterior cruciate ligament reconstruction	Friedmann-Bette, B.; Lornsen, H.; Parstorfer, M.; Gwechenberger, T.; Profit, F.; Weber, M. A.; BariÃ©, A.	2021	Did not perform fiber type analysis
Strength Training Effects on Muscular Regeneration after ACL Reconstruction	Friedmann-Bette, B.; Profit, F.; Gwechenberger, T.; Weiberg, N.; Parstorfer, M.; Weber, M. A.; Streich, N.; BariÃ©, A.	2018	Does not include male and female data
Akt2 influences glycogen synthase activity in human skeletal muscle through regulation of NHâ‚‚-terminal (sites 2 + 2a) phosphorylation	Friedrichsen, M.; Birk, J. B.; Richter, E. A.; Ribel-Madsen, R.; PehmÃ¸ller, C.; Hansen, B. F.; Beck-Nielsen, H.; Hirshman, M. F.; Goodyear, L. J.; Vaag, A.; Poulsen, P.; Wojtaszewski, J. F.	2013	Did not perform fiber type analysis

Skeletal muscle in paramyotonia congenita: biochemistry, histochemistry and morphology	Friis, M. L.; Johnsen, T.; Saltin, B.; Paulson, O. B.	1985	Does not include male and female data
Muscle fibre composition in relation to blood pressure response to isometric exercise in normotensive and hypertensive subjects	Frisk-Holmberg, M.; EssÃ©n, B.; Fredrikson, M.; StrÃ¶m, G.; Wibell, L.	1983	Does not include male and female data
Effects of small-sided recreational team handball training on mechanical muscle function, body composition and bone mineralization in untrained young adults-A randomized controlled trial	Fristrup, B.; Krustrup, P.; Andersen, J. L.; Hornstrup, T.; LÃ¸wenstein, F. T.; Larsen, M. A.; Helge, J. W.; PÃ³voas, S. C. A.; Aagaard, P.	2020	Does not include males and females
PC-1 content in skeletal muscle of non-obese, non-diabetic subjects: relationship to insulin receptor tyrosine kinase and whole body insulin sensitivity	Frittitta, L.; Youngren, J.; Vigneri, R.; Maddux, B. A.; Trischitta, V.; Goldfine, I. D.	1996	No healthy subjects or controls
Metabolic profile and nitric oxide synthase expression of skeletal muscle fibers are altered in patients with type 1 diabetes	Fritzsche, K.; BlÃ¼her, M.; Schering, S.; Buchwalow, I. B.; Kern, M.; Linke, A.; Oberbach, A.; Adams, V.; Punkt, K.	2008	Does not include male and female data
Skeletal muscle fiber quality in older men and women	Frontera, W. R.; Suh, D.; Krivickas, L. S.; Hughes, V. A.; Goldstein, R.; Roubenoff, R.	2000	Single Fiber Analysis
Aging of human muscle: understanding sarcopenia at the single muscle cell level	Frontera, W. R.; Zayas, A. R.; Rodriguez, N.	2012	Review (ex: systematic review/meta-analysis)
ACL injury reduces satellite cell abundance and promotes fibrogenic cell expansion within skeletal muscle	Fry, C. S.; Johnson, D. L.; Ireland, M. L.; Noehren, B.	2017	Does not include male and female data
Exercise induces interleukin-8 receptor (CXCR2) expression in human skeletal muscle	Frydelund-Larsen, L.; Penkowa, M.; Akerstrom, T.; Zankari, A.; Nielsen, S.; Pedersen, B. K.	2007	Does not include males and females
Exercise induces isoform-specific increase in 5'AMP-activated protein kinase activity in human skeletal muscle	Fujii, N.; Hayashi, T.; Hirshman, M. F.; Smith, J. T.; Habinowski, S. A.; Kaijser, L.; Mu, J.; Ljungqvist, O.; Birnbaum, M. J.; Witters, L. A.; Thorell, A.; Goodyear, L. J.	2000	Did not perform fiber type analysis
Essential amino acid and carbohydrate ingestion before resistance exercise does not enhance postexercise muscle protein synthesis	Fujita, S.; Dreyer, H. C.; Drummond, M. J.; Glynn, E. L.; Volpi, E.; Rasmussen, B. B.	2009	Did not perform fiber type analysis
Supraphysiological hyperinsulinaemia is necessary to stimulate skeletal muscle protein anabolism in older adults: evidence of a true age-related insulin resistance of muscle protein metabolism	Fujita, S.; Glynn, E. L.; Timmerman, K. L.; Rasmussen, B. B.; Volpi, E.	2009	Did not perform fiber type analysis
Effect of insulin on human skeletal muscle protein synthesis is modulated by insulin-induced changes in muscle blood flow and amino acid availability	Fujita, S.; Rasmussen, B. B.; Cadenas, J. G.; Grady, J. J.; Volpi, E.	2006	Did not perform fiber type analysis
Elevated expression of interleukin-18 in the granulomatous lesions of muscular sarcoidosis	Fukami, T.; Miyazaki, E.; Matsumoto, T.; Kumamoto, T.; Tsuda, T.	2001	No healthy subjects or controls
Morphological spectrum and clinical features of myopathies with tubular aggregates	Funk, F.; Ceuterick-de Groote, C.; Martin, J. J.; Meinhardt, A.; Taratuto, A. L.; De Bleecker, J.; Van Coster, R.; De Paepe, B.;	2013	Does not report sex of subjects

	Schara, U.; Vorgerd, M.; HÃ¤usler, M.; Koppi, S.; Maschke, M.; De Jonghe, P.; Van Maldergem, L.; Noel, S.; Zimmermann, C. W.; Wirth, S.; Isenmann, S.; Stadler, R.; SchrÃ¶der, J. M.; Schulz, J. B.; Weis, J.; Claeys, K. G.		
Adaptation to peripheral muscle training	Gaffney, F. A.; Grimby, G.; Danneskiold-SamsÃ¸e, B.; Halskov, O.	1981	Does not include male and female data
Analysis of the 4q35 chromatin organization reveals distinct long-range interactions in patients affected with Facio-Scapulo-Humeral Dystrophy	Gaillard, M. C.; Broucqsault, N.; Morere, J.; LaberthonniÃ¨re, C.; Dion, C.; Badja, C.; Roche, S.; Nguyen, K.; Magdinier, F.; Robin, J. D.	2019	Did not perform fiber type analysis
Enhanced skeletal muscle lipid oxidative efficiency in insulin-resistant vs insulin-sensitive nondiabetic, nonobese humans	Galgani, J. E.; Vasquez, K.; Watkins, G.; Dupuy, A.; Bertrand-Michel, J.; Levade, T.; Moro, C.	2013	Did not perform fiber type analysis
Suppression of skeletal muscle turnover in cancer cachexia: evidence from the transcriptome in sequential human muscle biopsies	Gallagher, I. J.; Stephens, N. A.; MacDonald, A. J.; Skipworth, R. J.; Husi, H.; Greig, C. A.; Ross, J. A.; Timmons, J. A.; Fearon, K. C.	2012	Did not perform fiber type analysis
Effects of 84-days of bedrest and resistance training on single muscle fibre myosin heavy chain distribution in human vastus lateralis and soleus muscles	Gallagher, P.; Trappe, S.; Harber, M.; Creer, A.; Mazzetti, S.; Trappe, T.; Alkner, B.; Tesch, P.	2005	Does not include males and females
Resting extracellular signal-regulated protein kinase 1/2 expression following a continuum of chronic resistance exercise training paradigms	Galpin, A. J.; Fry, A. C.; Nicoll, J. X.; Moore, C. A.; Schilling, B. K.; Thomason, D. B.	2016	Does not include males and females
Effects of non-esterified fatty acids on insulin-stimulated glucose transport in isolated skeletal muscle from patients with type 2 (non-insulin-dependent) diabetes mellitus	Galuska, D.; Nolte, L.; WahlstrÃ¶m, E.; Smedegaard Kristensen, J. S.; Wallberg-Henriksson, H.; Zierath, J. R.	1994	Does not include males and females
Metformin increases insulin-stimulated glucose transport in insulin-resistant human skeletal muscle	Galuska, D.; Zierath, J.; ThÃ¶rne, A.; Sonnenfeld, T.; Wallberg-Henriksson, H.	1991	Did not perform fiber type analysis
Overexpression of microRNA-206 in the skeletal muscle from myotonic dystrophy type 1 patients	Gambardella, S.; Rinaldi, F.; Lepore, S. M.; Viola, A.; Loro, E.; Angelini, C.; Vergani, L.; Novelli, G.; Botta, A.	2010	Did not perform fiber type analysis
Mitochondrial dysfunction and oxidative stress in patients with chronic kidney disease	Gamboa, J. L.; Billings, F. T. th; Bojanowski, M. T.; Gilliam, L. A.; Yu, C.; Roshanravan, B.; Roberts, L. J., 2nd; Himmelfarb, J.; Ikizler, T. A.; Brown, N. J.	2016	Did not perform fiber type analysis
Skeletal Muscle Mitochondrial Dysfunction Is Present in Patients with CKD before Initiation of Maintenance Hemodialysis	Gamboa, J. L.; Roshanravan, B.; Towse, T.; Keller, C. A.; Falck, A. M.; Yu, C.; Frontera, W. R.; Brown, N. J.; Ikizler, T. A.	2020	Did not perform fiber type analysis
The effect of unloading on protein synthesis in human skeletal muscle	Gamrin, L.; Berg, H. E.; EssÃ©n, P.; Tesch, P. A.; Hultman, E.; Garlick, P. J.; McNurlan, M. A.; Wernerman, J.	1998	Does not include males and females
A descriptive study of skeletal muscle metabolism in critically ill patients: free amino acids, energy-rich phosphates, protein, nucleic acids, fat, water, and electrolytes	Gamrin, L.; EssÃ©n, P.; Forsberg, A. M.; Hultman, E.; Wernerman, J.	1996	No healthy subjects or controls

Anterior shoulder instability: histomorphometric study of the subscapularis and deltoid muscles	Gamulin, A.; Pizzolato, G.; Stern, R.; Hoffmeyer, P.	2002	No cross-sectional area data
Novel ACTA1 mutation causes late-presenting nemaline myopathy with unusual dark cores	Garibaldi, M.; Fattori, F.; Pennisi, E. M.; Merlonghi, G.; Fionda, L.; Vanoli, F.; Leonardi, L.; Bucci, E.; Morino, S.; Micaloni, A.; Tartaglione, T.; Uijterwijk, B.; Zierikzee, M.; Ottenheijm, C.; Bertini, E. S.; Stoppacciaro, A.; Raffa, S.; Salvetti, M.; Antonini, G.	2021	No healthy subjects or controls
Cross-bridge mechanisms of muscle weakness in multiple sclerosis	Garner, D. J.; Widrick, J. J.	2003	Does not include male and female data
Motor unit organization of human medial gastrocnemius	Garnett, R. A.; O'Donovan, M. J.; Stephens, J. A.; Taylor, A.	1979	Does not include males and females
Chronic heart failure with diabetes mellitus is characterized by a severe skeletal muscle pathology	Garnham, J. O.; Roberts, L. D.; Espino-Gonzalez, E.; Whitehead, A.; Swoboda, P. P.; Koshy, A.; Gierula, J.; Paton, M. F.; Cubbon, R. M.; Kearney, M. T.; Egginton, S.; Bowen, T. S.; Witte, K. K.	2020	Does not include male and female data
Coordinated changes in mitochondrial function and biogenesis in healthy and diseased human skeletal muscle	Garnier, A.; Fortin, D.; Zoll, J.; N'Guessan, B.; Mettauer, B.; Lampert, E.; Veksler, V.; Ventura-Clapier, R.	2005	Did not perform fiber type analysis
Gene expression of GLUT4 in skeletal muscle from insulin-resistant patients with obesity, IGT, GDM, and NIDDM	Garvey, W. T.; Maianu, L.; Hancock, J. A.; Golichowski, A. M.; Baron, A.	1992	Does not include male and female data
Evidence for defects in the trafficking and translocation of GLUT4 glucose transporters in skeletal muscle as a cause of human insulin resistance	Garvey, W. T.; Maianu, L.; Zhu, J. H.; Brechtel-Hook, G.; Wallace, P.; Baron, A. D.	1998	Did not perform fiber type analysis
Investigation of glycosylation processes in mitochondria and microsomal membranes from human skeletal muscle	Gasnier, F.; Lerme, F.; Rousson, R.; Roussouly, P.; Vaganay, E.; Louisot, P.; Gateau-Roesch, O.	1991	No healthy subjects or controls
Induction of GLUT-1 protein in adult human skeletal muscle fibers	Gaster, M.; Franch, J.; Staehr, P.; Beck-Nielsen, H.; Smith, T.; SchrÃ¸der, H. D.	2000	Did not perform fiber type analysis
GLUT11, but not GLUT8 or GLUT12, is expressed in human skeletal muscle in a fibre type-specific pattern	Gaster, M.; Handberg, A.; SchÃ¼rmann, A.; Joost, H. G.; Beck-Nielsen, H.; SchrÃ¸der, H. D.	2004	Did not perform fiber type analysis
GLUT4 expression at the plasma membrane is related to fibre volume in human skeletal muscle fibres	Gaster, M.; Vach, W.; Beck-Nielsen, H.; SchrÃ¸der, H. D.	2002	Does not include males and females
Culturing of diagnostic muscle biopsies as spheroid-like structures: a pilot study of morphology and viability	GÃ¡ti, I.; Danielsson, O.; Betmark, T.; Ernerudh, J.; Ollinger, K.; Dizdar, N.	2010	Does not include males and females
Acute resistance exercise increases skeletal muscle angiogenic growth factor expression	Gavin, T. P.; Drew, J. L.; Kubik, C. J.; Pofahl, W. E.; Hickner, R. C.	2007	Did not perform fiber type analysis
Angiogenic growth factor response to acute systemic exercise in human skeletal muscle	Gavin, T. P.; Robinson, C. B.; Yeager, R. C.; England, J. A.; Nifong, L. W.; Hickner, R. C.	2004	Does not include males and females

Metabolic characteristics of the deltoid muscle in patients with chronic obstructive pulmonary disease	Gea, J. G.; Pasto, M.; Carmona, M. A.; Orozco-Levi, M.; Palomeque, J.; Broquetas, J.	2001	Does not include males and females
Local depletion of glycogen with supramaximal exercise in human skeletal muscle fibres	Gejl, K. D.; Ã˜rtenblad, N.; Andersson, E.; Plomgaard, P.; Holmberg, H. C.; Nielsen, J.	2017	Does not include males and females
Dissociation of intramyocellular lipid storage and insulin resistance in trained athletes and type 2 diabetes patients; involvement of perilipin 5?	Gemmink, A.; Daemen, S.; Brouwers, B.; Huntjens, P. R.; Schaart, G.; Moonen-Kornips, E.; JÃ¶rgensen, J.; Hoeks, J.; Schrauwen, P.; Hesselink, M. K. C.	2018	Does not include males and females
Inhibition of myogenic microRNAs 1, 133, and 206 by inflammatory cytokines links inflammation and muscle degeneration in adult inflammatory myopathies	Georgantas, R. W.; Streicher, K.; Greenberg, S. A.; Greenlees, L. M.; Zhu, W.; Brohawn, P. Z.; Higgs, B. W.; Czapiga, M.; Morehouse, C. A.; Amato, A.; Richman, L.; Jallal, B.; Yao, Y.; Ranade, K.	2014	Did not perform fiber type analysis
AG490 improves the survival of human myoblasts in vitro and in vivo	GÃ©rard, C.; Dufour, C.; Goudenege, S.; Skuk, D.; Tremblay, J. P.	2012	Autopsies
Skeletal muscle profiles among elite long, middle, and short distance swimmers	Gerard, E. S.; Caiozzo, V. J.; Rubin, B. D.; Prietto, C. A.; Davidson, D. M.	1986	Only in children (0-17 years)
The relationships between EMG and muscle morphology throughout sustained static knee extension at two submaximal force levels	Gerdle, B.; Karlsson, S.; Crenshaw, A. G.; FridÃ©n, J.	1997	Same subjects as another study
Elevated muscle TLR4 expression and metabolic endotoxemia in human aging	Ghosh, S.; Lertwattanarak, R.; GarduÃ±o Jde, J.; Galeana, J. J.; Li, J.; Zamarripa, F.; Lancaster, J. L.; Mohan, S.; Hussey, S.; Musi, N.	2015	Did not perform fiber type analysis
Atrophy, fibrosis, and increased PAX7-positive cells in pharyngeal muscles of oculopharyngeal muscular dystrophy patients	Gidaro, T.; Negroni, E.; PeriÃ©, S.; Mirabella, M.; LainÃ©, J.; Lacau St Guily, J.; Butler-Browne, G.; Mouly, V.; Trollet, C.	2013	Does not include male and female data
Fine structure of the human skeletal muscle capillary. A morphometric analysis	GidlÃ¶f, A.; Lewis, D. H.; Hammersen, F.	1988	Did not perform fiber type analysis
The effect of prolonged total ischemia on the ultrastructure of human skeletal muscle capillaries. A morphometric analysis	GidlÃ¶f, A.; Lewis, D. H.; Hammersen, F.	1988	Does not include males and females
Rapidly elevated levels of PGC-1Î±-b protein in human skeletal muscle after exercise: exploring regulatory factors in a randomized controlled trial	Gidlund, E. K.; Ydfors, M.; Appel, S.; Rundqvist, H.; Sundberg, C. J.; Norrbom, J.	2015	Did not perform fiber type analysis
The proteomic signature of insulin-resistant human skeletal muscle reveals increased glycolytic and decreased mitochondrial enzymes	Giebelstein, J.; Poschmann, G.; HÃ¸jlund, K.; Schechinger, W.; Dietrich, J. W.; Levin, K.; Beck-Nielsen, H.; Podwojski, K.; StÃ¼hler, K.; Meyer, H. E.; Klein, H. H.	2012	Does not include male and female data
Protein and amino acid metabolism during early starvation as reflected by excretion of urea and methylhistidines	Giesecke, K.; Magnusson, I.; Ahlberg, M.; Hagenfeldt, L.; Wahren, J.	1989	Does not include male and female data
Altered skeletal muscle mitochondrial phenotype in COPD: disease vs. disuse	Gifford, J. R.; Trinity, J. D.; Kwon, O. S.; Layec, G.; Garten, R. S.; Park, S. Y.; Nelson, A. D.; Richardson, R. S.	2018	Did not perform fiber type analysis
Elevated IL-4 and IFN-Î³ Levels in Muscle Tissue of Patients with Dermatomyositis	GiriÅŸ, M.; DurmuÅŸ, H.; Yetimler, B.; TaÅŸli, H.; Parman, Y.; TÃ¼zÃ¼n, E.	2017	Did not perform fiber type analysis

Overexpression of autophagic proteins in the skeletal muscle of sporadic inclusion body myositis	Girolamo, F.; Lia, A.; Amati, A.; Strippoli, M.; Coppola, C.; Virgintino, D.; Roncali, L.; Toscano, A.; Serlenga, L.; Trojano, M.	2013	No healthy subjects or controls
Myogenic Progenitor Cells Exhibit Type I Interferon-Driven Proangiogenic Properties and Molecular Signature During Juvenile Dermatomyositis	Gitiaux, C.; Latroche, C.; Weiss-Gayet, M.; Rodero, M. P.; Duffy, D.; Bader-Meunier, B.; Glorion, C.; Nusbaum, P.; Bodemer, C.; Mouchiroud, G.; Chelly, J.; Germain, S.; Desguerre, I.; Chazaud, B.	2018	Did not perform fiber type analysis
Expression of perilipins in human skeletal muscle in vitro and in vivo in relation to diet, exercise and energy balance	Gjelstad, I. M.; Haugen, F.; Gulseth, H. L.; Norheim, F.; Jans, A.; Bakke, S. S.; Raastad, T.; TjÃ¸nna, A. E.; WislÃ¸ff, U.; Blaak, E. E.; RisÃ©rus, U.; Gaster, M.; Roche, H. M.; Birkeland, K. I.; Drevon, C. A.	2012	Does not include male and female data
Skeletal muscle fibre types, physical performance, physical activity and attitude to physical activity in women and men. A follow-up from age 16 to 27	Glenmark, B.	1994	Same subjects as another study
Prediction of physical activity level in adulthood by physical characteristics, physical performance and physical activity in adolescence: an 11-year follow-up study	Glenmark, B.; Hedberg, G.; Jansson, E.	1994	No cross-sectional area data
Muscle strength from adolescence to adulthood--relationship to muscle fibre types	Glenmark, B.; Hedberg, G.; Kaijser, L.; Jansson, E.	1994	Same subjects as another study
Little change in markers of protein breakdown and oxidative stress in humans in immobilization-induced skeletal muscle atrophy	Glover, E. I.; Yasuda, N.; Tarnopolsky, M. A.; Abadi, A.; Phillips, S. M.	2010	Does not include male and female data
Environmental hypoxia favors myoblast differentiation and fast phenotype but blunts activation of protein synthesis after resistance exercise in human skeletal muscle	Gnimassou, O.; FernÃ¡ndez-Verdejo, R.; Brook, M.; Naslain, D.; Balan, E.; Sayda, M.; Cegielski, J.; Nielens, H.; Decottignies, A.; Demoulin, J. B.; Smith, K.; Atherton, P. J.; Francaux, M.; Deldicque, L.	2018	Does not include males and females
Skeletal muscle molecular alterations precede whole-muscle dysfunction in NYHA Class II heart failure patients	Godard, M. P.; Whitman, S. A.; Song, Y. H.; Delafontaine, P.	2012	Does not report sex of subjects
Metabolic adaptations to a high-fat diet in endurance cyclists	Goedecke, J. H.; Christie, C.; Wilson, G.; Dennis, S. C.; Noakes, T. D.; Hopkins, W. G.; Lambert, E. V.	1999	Does not include males and females
Insulin sensitivity measured by the minimal model: no associations with fasting respiratory exchange ratio in trained athletes	Goedecke, J. H.; Levitt, N. S.; St Clair Gibson, A.; Grobler, L.; Noakes, T. D.; Lambert, E. V.	2001	Does not include male and female data
Progressive defect of insulin action on glycogen synthase in obesity and diabetes	Golay, A.; Munger, R.; Assimacopoulos-Jeannet, F.; Bobbioni-Harsch, E.; Habicht, F.; Felber, J. P.	2002	Did not perform fiber type analysis
Non-invasive evaluation of buccal respiratory chain enzyme dysfunction in mitochondrial disease: comparison with studies in muscle biopsy	Goldenthal, M. J.; Kuruvilla, T.; Damle, S.; Salganicoff, L.; Sheth, S.; Shah, N.; Marks, H.; Khurana, D.; Valencia, I.; Legido, A.	2012	Did not perform muscle biopsy
Effects of experimental weight perturbation on skeletal muscle work efficiency, fuel utilization, and biochemistry in human subjects	Goldsmith, R.; Joanisse, D. R.; Gallagher, D.; Pavlovich, K.; Shamoon, E.; Leibel, R. L.; Rosenbaum, M.	2010	Does not include male and female data

Glycogen depletion patterns in human skeletal muscle fibers during prolonged work	Gollnick, P. D.; Armstrong, R. B.; Saubert, C. W. th; Sembrowich, W. L.; Shepherd, R. E.; Saltin, B.	1973	Does not include males and females
Phosphorylase a in human skeletal muscle during exercise and electrical stimulation	Gollnick, P. D.; Karlsson, J.; Piehl, K.; Saltin, B.	1978	Does not include males and females
The muscle fiber composition of skeletal muscle as a predictor of athletic success. An overview	Gollnick, P. D.; Matoba, H.	1984	Review (ex: systematic review/meta-analysis)
Selective glycogen depletion pattern in human muscle fibres after exercise of varying intensity and at varying pedalling rates	Gollnick, P. D.; Piehl, K.; Saltin, B.	1974	Does not include males and females
Human soleus muscle: a comparison of fiber composition and enzyme activities with other leg muscles	Gollnick, P. D.; SjÃ¶din, B.; Karlsson, J.; Jansson, E.; Saltin, B.	1974	Does not include male and female data
Cell interactome in sarcopenia during aging	GonzÃ¡lez-Blanco, L.; BermÃºdez, M.; Bermejo-Millo, J. C.; GutiÃ©rrez-RodrÃ-guez, J.; Solano, J. J.; AntuÃ±a, E.; MenÃ©ndez-Valle, I.; Caballero, B.; Vega-Naredo, I.; Potes, Y.; Coto-Montes, A.	2022	Did not perform fiber type analysis
Associations of Peripheral Artery Disease With Calf Skeletal Muscle Mitochondrial DNA Heteroplasmy	Gonzalez-Freire, M.; Moore, A. Z.; Peterson, C. A.; Kosmac, K.; McDermott, M. M.; Sufit, R. L.; Guralnik, J. M.; Polonsky, T.; Tian, L.; Kibbe, M. R.; Criqui, M. H.; Li, L.; Leeuwenburgh, C.; Ferrucci, L.	2020	Did not perform fiber type analysis
Unaccustomed eccentric contractions impair plasma K+ regulation in the absence of changes in muscle Na+,K+-ATPase content	Goodman, C. A.; Bennie, J. A.; Leikis, M. J.; McKenna, M. J.	2014	Did not perform fiber type analysis
Skeletal muscle lipid content and insulin resistance: evidence for a paradox in endurance-trained athletes	Goodpaster, B. H.; He, J.; Watkins, S.; Kelley, D. E.	2001	Does not include male and female data
Altered antioxidant status in peripheral skeletal muscle of patients with COPD	Gosker, H. R.; Bast, A.; Haenen, G. R.; Fischer, M. A.; van der Vusse, G. J.; Wouters, E. F.; Schols, A. M.	2005	Did not perform fiber type analysis
Reduced mitochondrial density in the vastus lateralis muscle of patients with COPD	Gosker, H. R.; Hesselink, M. K.; Duimel, H.; Ward, K. A.; Schols, A. M.	2007	Did not perform fiber type analysis
Myopathological features in skeletal muscle of patients with chronic obstructive pulmonary disease	Gosker, H. R.; Kubat, B.; Schaart, G.; van der Vusse, G. J.; Wouters, E. F.; Schols, A. M.	2003	Does not include male and female data
Exercise training restores uncoupling protein-3 content in limb muscles of patients with chronic obstructive pulmonary disease	Gosker, H. R.; Schrauwen, P.; Broekhuizen, R.; Hesselink, M. K.; Moonen-Kornips, E.; Ward, K. A.; Franssen, F. M.; Wouters, E. F.; Schols, A. M.	2006	Does not include male and female data
Uncoupling protein-3 content is decreased in peripheral skeletal muscle of patients with COPD	Gosker, H. R.; Schrauwen, P.; Hesselink, M. K.; Schaart, G.; van der Vusse, G. J.; Wouters, E. F.; Schols, A. M.	2003	Does not include male and female data
Skeletal muscle fibre-type shifting and metabolic profile in patients with chronic obstructive pulmonary disease	Gosker, H. R.; van Mameren, H.; van Dijk, P. J.; Engelen, M. P.; van der Vusse, G. J.; Wouters, E. F.; Schols, A. M.	2002	Does not include male and female data

A comparative study of the human external sphincter and periurethral levator ani muscles	Gosling, J. A.; Dixon, J. S.; Critchley, H. O.; Thompson, S. A.	1981	No healthy subjects or controls
Effects of transcutaneous electrical nerve stimulation on the H-reflex of muscles of different fibre type composition	Goulet, C. G.; Arsenault, A. B.; Bourbonnais, D.; Levin, M. F.	1997	Did not perform muscle biopsy
The relationship between muscle fiber type-specific PGC-1Î± content and mitochondrial content varies between rodent models and humans	Gouspillou, G.; Sgarioto, N.; Norris, B.; Barbat-Artigas, S.; Aubertin-Leheudre, M.; Morais, J. A.; Burelle, Y.; Taivassalo, T.; Hepple, R. T.	2014	Does not include males and females
Fiber atrophy, oxidative stress, and oxidative fiber reduction are the attributes of different phenotypes in chronic obstructive pulmonary disease patients	Gouzi, F.; Abdellaoui, A.; Molinari, N.; Pinot, E.; Ayoub, B.; Laoudj-Chenivesse, D.; Cristol, J. P.; Mercier, J.; Hayot, M.; PrÃ©faut, C.	2013	Does not include male and female data
Oxidative stress regulates autophagy in cultured muscle cells of patients with chronic obstructive pulmonary disease	Gouzi, F.; BlaquiÃ¨re, M.; Catteau, M.; Bughin, F.; Maury, J.; Passerieux, E.; Ayoub, B.; Mercier, J.; Hayot, M.; PomiÃ¨s, P.	2018	Did not perform fiber type analysis
Impaired training-induced adaptation of blood pressure in COPD patients: implication of the muscle capillary bed	Gouzi, F.; Maury, J.; Bughin, F.; BlaquiÃ¨re, M.; Ayoub, B.; Mercier, J.; Perez-Martin, A.; PomiÃ¨s, P.; Hayot, M.	2016	Did not perform fiber type analysis
Reference values for vastus lateralis fiber type proportion and fiber size	Gouzi, F.; Maury, J.; Molinari, N.; PomiÃ¨s, P.; Mercier, J.; PrÃ©faut, C.; Hayot, M.	2014	Not peer reviewed (ex: thesis, conference poster)
Blunted muscle angiogenic training-response in COPD patients versus sedentary controls	Gouzi, F.; PrÃ©faut, C.; Abdellaoui, A.; Roudier, E.; de Rigal, P.; Molinari, N.; Laoudj-Chenivesse, D.; Mercier, J.; Birot, O.; Hayot, M.	2013	Does not include male and female data
Glutamate ingestion: the plasma and muscle free amino acid pools of resting humans	Graham, T. E.; Sgro, V.; Friars, D.; Gibala, M. J.	2000	Did not perform fiber type analysis
Forty high-intensity interval training sessions blunt exercise-induced changes in the nuclear protein content of PGC-1Î± and p53 in human skeletal muscle	Granata, C.; Oliveira, R. S. F.; Little, J. P.; Bishop, D. J.	2020	Does not include males and females
Capillary supply in relation to myosin heavy chain fibre composition of human intrinsic tongue muscles	Granberg, I.; Lindell, B.; Eriksson, P. O.; Pedrosa-DomellÃ¶f, F.; StÃ¥l, P.	2010	Autopsies
Regeneration of Mitochondrial Function in Gastrocnemius Muscle in Peripheral Arterial Disease After Successful Revascularisation	Gratl, A.; Frese, J.; Speichinger, F.; Pesta, D.; Frech, A.; Omran, S.; Greiner, A.	2020	Did not perform fiber type analysis
Effect of weight loss on muscle lipid content in morbidly obese subjects	Gray, R. E.; Tanner, C. J.; Pories, W. J.; MacDonald, K. G.; Houmard, J. A.	2003	Does not include male and female data
Increased skeletal muscle expression of PKC-theta but not PKC-alpha mRNA in type 2 diabetes: inverse relationship with in-vivo insulin sensitivity	Gray, S.; Idris, I.; Davis, K. R.; Donnelly, R.	2003	Does not include males and females
Deregulated microRNAs in myotonic dystrophy type 2	Greco, S.; Perfetti, A.; Fasanaro, P.; Cardani, R.; Capogrossi, M. C.; Meola, G.; Martelli, F.	2012	No healthy subjects or controls

Adaptations in human muscle sarcoplasmic reticulum to prolonged submaximal training	Green, H. J.; Ballantyne, C. S.; MacDougall, J. D.; Tarnopolsky, M. A.; Schertzer, J. D.	2003	Does not include males and females
Vastus lateralis Na(+)-K(+)-ATPase activity, protein, and isoform distribution in chronic obstructive pulmonary disease	Green, H. J.; Burnett, M. E.; D'Arsigny, C. L.; Webb, K. A.; McBride, I.; Ouyang, J.; O'Donnell, D. E.	2009	Did not perform fiber type analysis
Increases in human skeletal muscle Na(+)-K(+)-ATPase concentration with short-term training	Green, H. J.; Chin, E. R.; Ball-Burnett, M.; Ranney, D.	1993	Does not include males and females
Male and female differences in enzyme activities of energy metabolism in vastus lateralis muscle	Green, H. J.; Fraser, I. G.; Ranney, D. A.	1984	Did not perform fiber type analysis
Fiber type distribution and maximal activities of enzymes involved in energy metabolism following short-term supramaximal exercise	Green, H. J.; Houston, M. E.; Thomson, J. A.; Fraser, I. G.	1984	Does not include males and females
Biochemical and histochemical alterations in skeletal muscle in man during a period of reduced activity	Green, H. J.; Thomson, J. A.; Daub, B. D.; Ranney, D. A.	1980	Does not report sex of subjects
Fiber composition, fiber size and enzyme activities in vastus lateralis of elite athletes involved in high intensity exercise	Green, H. J.; Thomson, J. A.; Daub, W. D.; Houston, M. E.; Ranney, D. A.	1979	Does not include males and females
Plasma cells in muscle in inclusion body myositis and polymyositis	Greenberg, S. A.; Bradshaw, E. M.; Pinkus, J. L.; Pinkus, G. S.; Burleson, T.; Due, B.; Bregoli, L.; O'Connor, K. C.; Amato, A. A.	2005	No healthy subjects or controls
Association of inclusion body myositis with T cell large granular lymphocytic leukaemia	Greenberg, S. A.; Pinkus, J. L.; Amato, A. A.; Kristensen, T.; Dorfman, D. M.	2016	Did not perform fiber type analysis
Highly differentiated cytotoxic T cells in inclusion body myositis	Greenberg, S. A.; Pinkus, J. L.; Kong, S. W.; Baecher-Allan, C.; Amato, A. A.; Dorfman, D. M.	2019	Does not report sex of subjects
Nuclear localization of valosin-containing protein in normal muscle and muscle affected by inclusion-body myositis	Greenberg, S. A.; Watts, G. D.; Kimonis, V. E.; Amato, A. A.; Pinkus, J. L.	2007	Did not perform fiber type analysis
Regulators of blood lipids and lipoproteins? PPARÎ´ and AMPK, induced by exercise, are correlated with lipids and lipoproteins in overweight/obese men and women	Greene, N. P.; Fluckey, J. D.; Lambert, B. S.; Greene, E. S.; Riechman, S. E.; Crouse, S. F.	2012	Did not perform fiber type analysis
Effect of oral creatine supplementation on skeletal muscle phosphocreatine resynthesis	Greenhaff, P. L.; Bodin, K.; Soderlund, K.; Hultman, E.	1994	Does not include males and females
The metabolic responses of human type I and II muscle fibres during maximal treadmill sprinting	Greenhaff, P. L.; Nevill, M. E.; Soderlund, K.; Bodin, K.; Boobis, L. H.; Williams, C.; Hultman, E.	1994	Does not include male and female data
Energy metabolism in single human muscle fibers during contraction without and with epinephrine infusion	Greenhaff, P. L.; Ren, J. M.; SÃ¶derlund, K.; Hultman, E.	1991	Does not include male and female data
Energy metabolism in single human muscle fibres during intermittent contraction with occluded circulation	Greenhaff, P. L.; SÃ¶derlund, K.; Ren, J. M.; Hultman, E.	1993	Does not include male and female data
Acute responsiveness to single leg cycling in adults with obesity	Gries, K. J.; Hart, C. R.; Kunz, H. E.; Ryan, Z.; Zhang, X.; Parvizi, M.; Liu, Y.; Dasari, S.; Lanza, I. R.	2022	Did not perform fiber type analysis

Mechanism of muscle wasting in myotonic dystrophy	Griggs, R. C.; Jozefowicz, R.; Kingston, W.; Nair, K. S.; Herr, B. E.; Halliday, D.	1990	Does not include males and females
Physical activity and effects of muscle training in the elderly	Grimby, G.	1988	Does not report sex of subjects
Is there a change in relative muscle fibre composition with age?	Grimby, G.; Aniansson, A.; Zetterberg, C.; Saltin, B.	1984	No healthy subjects or controls
Muscle fiber composition in patients with traumatic cord lesion	Grimby, G.; Broberg, C.; Krotkiewska, I.; Krotkiewski, M.	1976	No healthy subjects or controls
Morphology and enzymatic capacity in arm and leg muscles in 78-81 year old men and women	Grimby, G.; Danneskiold-SamsÃ¸e, B.; Hvid, K.; Saltin, B.	1982	No healthy subjects or controls
Fatigue of chronically overused motor units in prior polio patients	Grimby, L.; TollbÃ¤ck, A.; MÃ¼ller, U.; Larsson, L.	1996	No healthy subjects or controls
Delays in insulin signaling towards glucose disposal in human skeletal muscle	Grimmsmann, T.; Levin, K.; Meyer, M. M.; Beck-Nielsen, H.; Klein, H. H.	2002	Does not include males and females
Improved single muscle fiber quality in the oldest-old	Grosicki, G. J.; Standley, R. A.; Murach, K. A.; Raue, U.; Minchev, K.; Coen, P. M.; Newman, A. B.; Cummings, S.; Harris, T.; Kritchevsky, S.; Goodpaster, B. H.; Trappe, S.	2016	Does not include male and female data
Increased expression of glial cell line-derived neurotrophic factor mRNA in muscle biopsies from patients with amyotrophic lateral sclerosis	GrundstrÃ¶m, E.; Askmark, H.; Lindeberg, J.; Nygren, I.; Ebendal, T.; Aquilonius, S. M.	1999	Did not perform fiber type analysis
Vascular endothelial growth factor is highly expressed in muscle tissue of patients with polymyositis and patients with dermatomyositis	Grundtman, C.; Tham, E.; Ulfgren, A. K.; Lundberg, I. E.	2008	Did not perform fiber type analysis
The triglyceride content in skeletal muscle is associated with hepatic but not peripheral insulin resistance in elderly twins	Grunnet, L. G.; Laurila, E.; Hansson, O.; Almgren, P.; Groop, L.; BrÃ¸ns, C.; Poulsen, P.; Vaag, A.	2012	No cross-sectional area data
Intracellular bicarbonate and pH of skeletal muscle in chronic renal failure	Guariglia, A.; Antonucci, C.; Coffrini, E.; Del Canale, S.; Fiaccadori, E.; Reni, F.; Vitali, P.; Arduini, U.; Borghetti, A.	1985	Did not perform fiber type analysis
The serum nitric oxide levels in patients with Duchenne muscular dystrophy	GÃ¼cÃ¼yener, K.; Ergenekon, E.; Erbas, D.; Pinarli, G.; SerdaroÄŸlu, A.	2000	Does not include males and females
Fine needle aspiration coupled with real-time PCR: a painless methodology to study adaptive functional changes in skeletal muscle	Guescini, M.; Fatone, C.; Stocchi, L.; Guidi, C.; Potenza, L.; Ditroilo, M.; Ranchelli, A.; Di Loreto, C.; Sisti, D.; De Feo, P.; Stocchi, V.	2007	Does not include males and females
Characterization of sarcoplasmic reticulum Ca(2+) ATPase pumps in muscle of patients with myotonic dystrophy and with hypothyroid myopathy	Guglielmi, V.; Oosterhof, A.; Voermans, N. C.; Cardani, R.; Molenaar, J. P.; van Kuppevelt, T. H.; Meola, G.; van Engelen, B. G.; Tomelleri, G.; Vattemi, G.	2016	Did not perform fiber type analysis
Effects of conditioned media from murine lung cancer cells and human tumor cells on cultured myotubes	Guigni, B. A.; van der Velden, J.; Kinsey, C. M.; Carson, J. A.; Toth, M. J.	2020	Did not perform fiber type analysis
miR-708-5p and miR-34c-5p are involved in nNOS regulation in dystrophic context	Guilbaud, M.; Gentil, C.; Peccate, C.; Gargaun, E.; Holtzmann, I.; Gruszczynski, C.;	2018	Does not report sex of subjects

	Falcone, S.; Mamchaoui, K.; Ben Yaou, R.; Leturcq, F.; Jeanson-Leh, L.; PiÃ©tri-Rouxel, F.		
The A-allele of the FTO Gene rs9939609 Polymorphism Is Associated With Decreased Proportion of Slow Oxidative Muscle Fibers and Over-represented in Heavier Athletes	Guilherme, Jplf; Egorova, E. S.; Semenova, E. A.; Kostryukova, E. S.; Kulemin, N. A.; Borisov, O. V.; Khabibova, S. A.; Larin, A. K.; Ospanova, E. A.; Pavlenko, A. V.; Lyubaeva, E. V.; Popov, D. V.; Lysenko, E. A.; Vepkhvadze, T. F.; Lednev, E. M.; Govorun, V. M.; Generozov, E. V.; Ahmetov,, II; Lancha Junior, A. H.	2019	Did not perform fiber type analysis
Investigation of fluoroquinolone-induced myalgia using (31)P magnetic resonance spectroscopy and in vitro contracture tests	Guis, S.; Bendahan, D.; Kozak-Ribbens, G.; Mattei, J. P.; Le Fur, Y.; Confort-Gouny, S.; Figarella-Branger, D.; Jouglard, J.; Cozzone, P. J.	2002	No healthy subjects or controls
Differential effects of acute hypertriglyceridemia on insulin action and insulin receptor autophosphorylation	Gumbiner, B.; Mucha, J. F.; Lindstrom, J. E.; Rekhi, I.; Livingston, J. N.	1996	Did not perform fiber type analysis
Altered mitochondrial network morphology and regulatory proteins in mitochondrial quality control in myotubes from severely obese humans with or without type 2 diabetes	Gundersen, A. E.; Kugler, B. A.; McDonald, P. M.; Veraksa, A.; Houmard, J. A.; Zou, K.	2020	Does not include males and females
Effect of whey protein- and carbohydrate-enriched diet on glycogen resynthesis during the first 48 h after a soccer game	Gunnarsson, T. P.; Bendiksen, M.; Bischoff, R.; Christensen, P. M.; Lesivig, B.; Madsen, K.; Stephens, F.; Greenhaff, P.; Krustrup, P.; Bangsbo, J.	2013	Does not include males and females
Effect of additional speed endurance training on performance and muscle adaptations	Gunnarsson, T. P.; Christensen, P. M.; Holse, K.; Christiansen, D.; Bangsbo, J.	2012	Does not include males and females
Differential patterns of transcript accumulation during human myogenesis	Gunning, P.; Hardeman, E.; Wade, R.; Ponte, P.; Bains, W.; Blau, H. M.; Kedes, L.	1987	No healthy subjects or controls
Increased nucleobindin-2 (NUCB2) transcriptional activity links the regulation of insulin sensitivity in Type 2 diabetes mellitus	Guo, Y.; Liao, Y.; Fang, G.; Dong, J.; Li, Z.	2013	Did not perform fiber type analysis
Kinetics of intramuscular triglyceride fatty acids in exercising humans	Guo, Z.; Burguera, B.; Jensen, M. D.	2000	Did not perform fiber type analysis
Prior heavy exercise elevates pyruvate dehydrogenase activity and speeds O2 uptake kinetics during subsequent moderate-intensity exercise in healthy young adults	Gurd, B. J.; Peters, S. J.; Heigenhauser, G. J.; LeBlanc, P. J.; Doherty, T. J.; Paterson, D. H.; Kowalchuk, J. M.	2006	Does not include males and females
Skeletal muscle magnesium and potassium in asthmatics treated with oral beta 2-agonists	Gustafson, T.; Boman, K.; Rosenhall, L.; SandstrÃ¶m, T.; Wester, P. O.	1996	Did not perform fiber type analysis
Exercise-induced expression of angiogenesis-related transcription and growth factors in human skeletal muscle	Gustafsson, T.; Puntschart, A.; Kaijser, L.; Jansson, E.; Sundberg, C. J.	1999	Does not include males and females
The influence of physical training on the angiopoietin and VEGF-A systems in human skeletal muscle	Gustafsson, T.; Rundqvist, H.; Norrbom, J.; Rullman, E.; Jansson, E.; Sundberg, C. J.	2007	Does not include males and females

ATOH8: a novel marker in human muscle fiber regeneration	GÃ¼ttsches, A. K.; Balakrishnan-Renuka, A.; Kley, R. A.; Tegenthoff, M.; Brand-Saberi, B.; Vorgerd, M.	2015	Did not perform fiber type analysis
Differing physiological effects of epinephrine in type 1 diabetes and nondiabetic humans	Guy, D. A.; Sandoval, D.; Richardson, M. A.; Tate, D.; Flakoll, P. J.; Davis, S. N.	2005	Did not perform fiber type analysis
Pretarsal and marginal orbicularis oculi muscle fiber changes in trachomatous cicatricial entropion: histopathological evaluation	Guzey, M.; Basar, E.; Ermis, S. S.; Bitiren, M.; Ozardali, I.; Mirzatas, C.	1999	No healthy subjects or controls
Transforming growth factor-beta 1 produced by vascular smooth muscle cells predicts fibrosis in the gastrocnemius of patients with peripheral artery disease	Ha, D. M.; Carpenter, L. C.; Koutakis, P.; Swanson, S. A.; Zhu, Z.; Hanna, M.; DeSpiegelaere, H. K.; Pipinos,, II; Casale, G. P.	2016	Does not include male and female data
Gene expression of group II phospholipase A2 in intestine in ulcerative colitis	HaapamÃ¤ki, M. M.; GrÃ¶nroos, J. M.; Nurmi, H.; Alanen, K.; Kallajoki, M.; Nevalainen, T. J.	1997	No healthy subjects or controls
Fat-induced membrane cholesterol accrual provokes cortical filamentous actin destabilisation and glucose transport dysfunction in skeletal muscle	Habegger, K. M.; Penque, B. A.; Sealls, W.; Tackett, L.; Bell, L. N.; Blue, E. K.; Gallagher, P. J.; Sturek, M.; Alloosh, M. A.; Steinberg, H. O.; Considine, R. V.; Elmendorf, J. S.	2012	Did not perform fiber type analysis
Comparative metabolomics of muscle interstitium fluid in human trapezius myalgia: an in vivo microdialysis study	HadrÃ©vi, J.; Ghafouri, B.; SjÃ¶rs, A.; Antti, H.; Larsson, B.; Crenshaw, A. G.; Gerdle, B.; HellstrÃ¶m, F.	2013	Does not include males and female
The blood pressure response to antihypertensive treatment with lisinopril or bendrofluazide is related to the calcium and magnesium contents in skeletal muscle	Haenni, A.; Lind, L.; Lithell, H.	1996	Did not perform fiber type analysis
Skeletal muscle magnesium content is correlated with plasma glucose concentration in patients with essential hypertension treated with lisinopril or bendrofluazide	Haenni, A.; Reneland, R.; Andersson, P. E.; Lind, L.; Lithell, H.	2002	Did not perform fiber type analysis
Cylinder or mobile cast brace after knee ligament surgery. A clinical analysis and morphologic and enzymatic studies of changes in the quadriceps muscle	HÃ¤ggmark, T.; Eriksson, E.	1979	Does not include male and female data
Cross-sectional area of the thigh muscle in man measured by computed tomography	HÃ¤ggmark, T.; Jansson, E.; Svane, B.	1978	Does not include males and females
Fibre types in human abdominal muscles	HÃ¤ggmark, T.; Thorstensson, A.	1979	Does not include male and female data
Differential distribution of immunoreactive S100-alpha and S100-beta proteins in normal nonnervous human tissues	Haimoto, H.; Hosoda, S.; Kato, K.	1987	Does not report sex of subjects
Mitochondrial dysfunction in airways and quadriceps muscle of patients with chronic obstructive pulmonary disease	Haji, G.; Wiegman, C. H.; Michaeloudes, C.; Patel, M. S.; Curtis, K.; Bhavsar, P.; Polkey, M. I.; Adcock, I. M.; Chung, K. F.	2020	Did not perform fiber type analysis
Electromyographic and force production characteristics of leg extensor muscles of elite weight lifters during isometric, concentric, and various stretch-shortening cycle exercises	HÃ¤kkinen, K.; Komi, P. V.; Kauhanen, H.	1986	Does not include males and females

Rate of protein synthesis in skeletal muscle of normal man and patients with muscular dystrophy: a reassessment	Halliday, D.; Pacy, P. J.; Cheng, K. N.; Dworzak, F.; Gibson, J. N.; Rennie, M. J.	1988	Did not perform fiber type analysis
Exercise intolerance in patients with chronic heart failure and increased expression of inducible nitric oxide synthase in the skeletal muscle	Hambrecht, R.; Adams, V.; Gielen, S.; Linke, A.; MÃ¶bius-Winkler, S.; Yu, J.; Niebauer, J.; Jiang, H.; Fiehn, E.; Schuler, G.	1999	Does not include males and females
Physical training in patients with stable chronic heart failure: effects on cardiorespiratory fitness and ultrastructural abnormalities of leg muscles	Hambrecht, R.; Niebauer, J.; Fiehn, E.; KÃ¤lberer, B.; Offner, B.; Hauer, K.; Riede, U.; Schlierf, G.; KÃ¼bler, W.; Schuler, G.	1995	Does not include males and females
Reduction of insulin-like growth factor-I expression in the skeletal muscle of noncachectic patients with chronic heart failure	Hambrecht, R.; Schulze, P. C.; Gielen, S.; Linke, A.; MÃ¶bius-Winkler, S.; Yu, J.; Kratzsch, J. JÃ¼; Baldauf, G.; Busse, M. W.; Schubert, A.; Adams, V.; Schuler, G.	2002	Does not include males and females
Expression of IGF-I splice variants in young and old human skeletal muscle after high resistance exercise	Hameed, M.; Orrell, R. W.; Cobbold, M.; Goldspink, G.; Harridge, S. D.	2003	Does not include males and females
Stress hormones initiate prolonged changes in the muscle amino acid pattern	Hammarqvist, F.; Ejesson, B.; Wernerman, J.	2001	Does not include males and females
Alpha-ketoglutarate preserves protein synthesis and free glutamine in skeletal muscle after surgery	Hammarqvist, F.; Wernerman, J.; von der Decken, A.; Vinnars, E.	1991	Did not perform fiber type analysis
The occurrence of paracrystalline mitochondrial inclusions in normal human skeletal muscle	Hammersen, F.; GidlÃ¶f, A.; Larsson, J.; Lewis, D. H.	1980	No healthy subjects or controls
Slow tonic muscle fibers in the thyroarytenoid muscles of human vocal folds; a possible specialization for speech	Han, Y.; Wang, J.; Fischman, D. A.; Biller, H. F.; Sanders, I.	1999	Does not include male and female data
Peripheral glucose uptake and skeletal muscle GLUT4 content in man: effect of insulin and free fatty acids	Handberg, A.; Vaag, A.; Beck-Nielsen, H.; Vinten, J.	1992	Did not perform fiber type analysis
Decreased tyrosine kinase activity in partially purified insulin receptors from muscle of young, non-obese first degree relatives of patients with type 2 (non-insulin-dependent) diabetes mellitus	Handberg, A.; Vaag, A.; Vinten, J.; Beck-Nielsen, H.	1993	Did not perform fiber type analysis
Expression of protocadherin gamma in skeletal muscle tissue is associated with age and muscle weakness	Hangelbroek, R. W.; Fazelzadeh, P.; Tieland, M.; Boekschoten, M. V.; Hooiveld, G. J.; van Duynhoven, J. P.; Timmons, J. A.; Verdijk, L. B.; de Groot, L. C.; van Loon, L. J.; MÃ¼ller, M.	2016	Did not perform fiber type analysis
Ecto- and cytosolic 5'-nucleotidases in normal and AMP deaminase-deficient human skeletal muscle	Hanisch, F.; Hellsten, Y.; Zierz, S.	2006	Does not include male and female data
Supplement with whey protein hydrolysate in contrast to carbohydrate supports mitochondrial adaptations in trained runners	Hansen, M.; Oxfeldt, M.; Larsen, A. E.; Thomsen, L. S.; Rokkedal-Lausch, T.; Christensen, B.; Rittig, N.; De Paoli, F. V.; Bangsbo, J.; Ã˜rtenblad, N.; Madsen, K.	2020	Did not perform fiber type analysis
Expression of insulin receptor spliced variants and their functional correlates in muscle from patients with non-insulin-dependent diabetes mellitus	Hansen, T.; BjÃ¸rbaek, C.; Vestergaard, H.; GrÃ¸nskov, K.; Bak, J. F.; Pedersen, O.	1993	Did not perform fiber type analysis

Myonuclear content regulates cell size with similar scaling properties in mice and humans	Hansson, K. A.; EftestÃ¸l, E.; Bruusgaard, J. C.; Juvkam, I.; Cramer, A. W.; Malthe-SÃ¸renssen, A.; Millay, D. P.; Gundersen, K.	2020	Does not include males and females
Fumarase activity in skeletal muscle of man	Haralambie, G.	1977	Does not include males and females
Further studies on creatine kinase activity in human skeletal muscle	Haralambie, G.	1978	Did not perform fiber type analysis
Single muscle fiber contractile properties of young competitive distance runners	Harber, M.; Trappe, S.	2008	Does not include males and females
Mechanisms of enhanced insulin sensitivity in endurance-trained athletes: effects on blood flow and differential expression of GLUT 4 in skeletal muscles	Hardin, D. S.; Azzarelli, B.; Edwards, J.; Wigglesworth, J.; Maianu, L.; Brechtel, G.; Johnson, A.; Baron, A.; Garvey, W. T.	1995	Does not include males and females
Mechanisms of insulin resistance in cystic fibrosis	Hardin, D. S.; Leblanc, A.; Marshall, G.; Seilheimer, D. K.	2001	Did not perform fiber type analysis
Effects of sprint training on extrarenal potassium regulation with intense exercise in Type 1 diabetes	Harmer, A. R.; Ruell, P. A.; McKenna, M. J.; Chisholm, D. J.; Hunter, S. K.; Thom, J. M.; Morris, N. R.; Flack, J. R.	2006	Did not perform fiber type analysis
Carnitine measurements in liver, muscle tissue, and blood in normal subjects	Harper, P.; WadstrÃ¶m, C.; Cederblad, G.	1993	Did not perform fiber type analysis
The histochemistry of reactive masticatory muscle hypertrophy	Harriman, D. G.	1996	Autopsies
Increased skeletal muscle mitochondrial free radical production in peripheral arterial disease despite preserved mitochondrial respiratory capacity	Hart, C. R.; Layec, G.; Trinity, J. D.; Kwon, O. S.; Zhao, J.; Reese, V. R.; Gifford, J. R.; Richardson, R. S.	2018	Does not include male and female data
Body composition, cardiovascular risk factors and liver function in long-term androgenic-anabolic steroids using bodybuilders three months after drug withdrawal	Hartgens, F.; Kuipers, H.; Wijnen, J. A.; Keizer, H. A.	1996	Does not include males and females
Gene expression comparison of biopsies from Duchenne muscular dystrophy (DMD) and normal skeletal muscle	Haslett, J. N.; Sanoudou, D.; Kho, A. T.; Bennett, R. R.; Greenberg, S. A.; Kohane, I. S.; Beggs, A. H.; Kunkel, L. M.	2002	Did not perform fiber type analysis
Gene expression profiling of Duchenne muscular dystrophy skeletal muscle	Haslett, J. N.; Sanoudou, D.; Kho, A. T.; Han, M.; Bennett, R. R.; Kohane, I. S.; Beggs, A. H.; Kunkel, L. M.	2003	Does not include males and females
Isolation of human skeletal muscle myosin heavy chain and actin for measurement of fractional synthesis rates	Hasten, D. L.; Morris, G. S.; Ramanadham, S.; Yarasheski, K. E.	1998	Did not perform fiber type analysis
Skeletal muscle responses to lower limb suspension in humans	Hather, B. M.; Adams, G. R.; Tesch, P. A.; Dudley, G. A.	1992	Does not include male and female data
Dietary intervention increases n-3 long-chain polyunsaturated fatty acids in skeletal muscle membrane phospholipids of obese subjects. Implications for insulin sensitivity	Haugaard, S. B.; Madsbad, S.; HÃ¸y, C. E.; Vaag, A.	2006	Did not perform fiber type analysis

Skeletal muscle structural lipids improve during weight-maintenance after a very low calorie dietary intervention	Haugaard, S. B.; Vaag, A.; Mu, H.; Madsbad, S.	2009	Did not perform fiber type analysis
Contractile and connective tissue protein content of human skeletal muscle: effects of 35 and 90 days of simulated microgravity and exercise countermeasures	Haus, J. M.; Carrithers, J. A.; Carroll, C. C.; Tesch, P. A.; Trappe, T. A.	2007	Did not perform fiber type analysis
Skeletal muscle microbiopsy: a validation study of a minimally invasive technique	Hayot, M.; Michaud, A.; Koechlin, C.; Caron, M. A.; Leblanc, P.; PrÃ©faut, C.; Maltais, F.	2005	Does not include males and females
Structural changes to airway smooth muscle in cystic fibrosis	Hays, S. R.; Ferrando, R. E.; Carter, R.; Wong, H. H.; Woodruff, P. G.	2005	Did not perform fiber type analysis
Muscle glycogen content in type 2 diabetes mellitus	He, J.; Kelley, D. E.	2004	Does not include male and female data
ATP consumption and efficiency of human single muscle fibers with different myosin isoform composition	He, Z. H.; Bottinelli, R.; Pellegrino, M. A.; Ferenczi, M. A.; Reggiani, C.	2000	Does not include males and females
Fibre loss and distribution in skeletal muscle from patients with severe peripheral arterial insufficiency	Hedberg, B.; Angquist, K. A.; Henriksson-Larsen, K.; SjÃ¶strÃ¶m, M.	1989	No healthy subjects or controls
Mitochondrial DNA variants in inclusion body myositis characterized by deep sequencing	Hedberg-Oldfors, C.; Lindgren, U.; Basu, S.; Visuttijai, K.; Lindberg, C.; Falkenberg, M.; Larsson Lekholm, E.; Oldfors, A.	2021	Did not perform fiber type analysis
Mitochondrial DNA expression in mitochondrial myopathies and coordinated expression of nuclear genes involved in ATP production	Heddi, A.; Lestienne, P.; Wallace, D. C.; Stepien, G.	1993	Did not perform fiber type analysis
Type 2 iodothyronine deiodinase in skeletal muscle: effects of hypothyroidism and fasting	Heemstra, K. A.; Soeters, M. R.; Fliers, E.; Serlie, M. J.; Burggraaf, J.; van Doorn, M. B.; van der Klaauw, A. A.; Romijn, J. A.; Smit, J. W.; Corssmit, E. P.; Visser, T. J.	2009	Does not include males and females
Reproducibility and absolute quantification of muscle glycogen in patients with glycogen storage disease by 13C NMR spectroscopy at 7 Tesla	Heinicke, K.; Dimitrov, I. E.; Romain, N.; Cheshkov, S.; Ren, J.; Malloy, C. R.; Haller, R. G.	2014	Did not perform fiber type analysis
Response of skeletal muscle UCP2-expression during metabolic adaptation to caloric restriction	Heinitz, S.; Piaggi, P.; Yang, S.; Bonfiglio, S.; Steel, J.; Krakoff, J.; Votruba, S. B.	2018	Did not perform fiber type analysis
The pathology of the lower leg muscles in pure forefoot pes cavus	Helliwell, T. R.; Tynan, M.; Hayward, M.; Klenerman, L.; Whitehouse, G.; Edwards, R. H.	1995	Autopsies
Effect of sprint cycle training on activities of antioxidant enzymes in human skeletal muscle	Hellsten, Y.; Apple, F. S.; SjÃ¶din, B.	1996	Does not include males and females
Expression of interleukin-18 in muscle tissue of patients with polymyositis or dermatomyositis and effects of conventional immunosuppressive treatment	Helmers, S. B.; Bruton, M.; Loell, I.; Ulfgren, A. K.; Gracie, A. J.; McInnes, I. B.; Lundberg, I. E.	2018	Did not perform fiber type analysis
Glucose processing during the intravenous glucose tolerance test	Henriksen, J. E.; Alford, F.; Handberg, A.; Vaag, A.; Beck-Nielsen, H.	1996	Does not include males and females

Intracellular skeletal muscle glucose metabolism is differentially altered by dexamethasone treatment of normoglycemic relatives of type 2 diabetic patients	Henriksen, J. E.; Alford, F.; Vaag, A.; Handberg, A.; Beck-Nielsen, H.	1999	Did not perform fiber type analysis
Effects of preoperative oral carbohydrates and peptides on postoperative endocrine response, mobilization, nutrition and muscle function in abdominal surgery	Henriksen, M. G.; Hessov, I.; Dela, F.; Hansen, H. V.; Haraldsted, V.; Rodt, S. A.	2003	Did not perform fiber type analysis
Redox state changes in human skeletal muscle after isometric contraction	Henriksson, J.; Katz, A.; Sahlin, K.	1986	Did not perform fiber type analysis
Distribution, number and size of different types of fibres in whole cross-sections of female m tibialis anterior. An enzyme histochemical study	Henriksson-LarsÃ©n, K.	1985	Does not include males and females
Distribution of fibre sizes in human skeletal muscle. An enzyme histochemical study in m tibialis anterior	Henriksson-LarsÃ©n, K.; FridÃ©n, J.; Wretling, M. L.	1985	Autopsies
Distribution of different fibre types in human skeletal muscles. I. Method for the preparation and analysis of cross-sections of whole tibialis anterior	Henriksson-LarsÃ©n, K. B.; Lexell, J.; SjÃ¶strÃ¶m, M.	1983	Autopsies
A noninvasive, log-transform method for fiber type discrimination using mechanomyography	Herda, T. J.; Housh, T. J.; Fry, A. C.; Weir, J. P.; Schilling, B. K.; Ryan, E. D.; Cramer, J. T.	2010	Does not include males and females
The change in motor unit firing rates at de-recruitment relative to recruitment is correlated with type I myosin heavy chain isoform content of the vastus lateralis in vivo	Herda, T. J.; Miller, J. D.; Trevino, M. A.; Mosier, E. M.; Gallagher, P. M.; Fry, A. C.; Vardiman, J. P.	2016	Does not include male and female data
Effect of tolerating macronutrient deficit on the development of intensive-care unit acquired weakness: a subanalysis of the EPaNIC trial	Hermans, G.; Casaer, M. P.; Clerckx, B.; GÃ¼iza, F.; Vanhullebusch, T.; Derde, S.; Meersseman, P.; Derese, I.; Mesotten, D.; Wouters, P. J.; Van Cromphaut, S.; Debaveye, Y.; Gosselink, R.; Gunst, J.; Wilmer, A.; Van den Berghe, G.; Vanhorebeek, I.	2013	Does not include male and female data
Oral creatine supplementation facilitates the rehabilitation of disuse atrophy and alters the expression of muscle myogenic factors in humans	Hespel, P.; Op't Eijnde, B.; Van Leemputte, M.; UrsÃ¸, B.; Greenhaff, P. L.; Labarque, V.; Dymarkowski, S.; Van Hecke, P.; Richter, E. A.	2001	Does not include male and female data
Light-microscopic study of the beta 1 integrin subunit in human skeletal muscle	Heub, D.; NeundÃ¶rfer, B.	1997	No healthy subjects or controls
Light-microscopic study of phosphoprotein B-50 in myopathies	Heuss, D.; Engelhardt, A.; GÃ¶bel, H.; NeundÃ¶rfer, B.	1995	No healthy subjects or controls
Potential for strength and endurance training to amplify endurance performance	Hickson, R. C.; Dvorak, B. A.; Gorostiaga, E. M.; Kurowski, T. T.; Foster, C.	1988	Does not include male and female data
Structural and metabolic characteristics of human skeletal muscle following 30 days of simulated microgravity	Hikida, R. S.; Gollnick, P. D.; Dudley, G. A.; Convertino, V. A.; Buchanan, P.	1989	Does not report sex of subjects

Muscle fiber necrosis associated with human marathon runners	Hikida, R. S.; Staron, R. S.; Hagerman, F. C.; Sherman, W. M.; Costill, D. L.	1983	Does not include males and females
Kinetic properties of myosin heavy chain isoforms in single fibers from human skeletal muscle	Hilber, K.; Galler, S.; Gohlsch, B.; Pette, D.	1999	Does not include male and female data
Sarcoplasmic reticulum function and muscle contractile character following fatiguing exercise in humans	Hill, C. A.; Thompson, M. W.; Ruell, P. A.; Thom, J. M.; White, M. J.	2001	Did not perform fiber type analysis
Physiological hyperinsulinemia stimulates p70(S6k) phosphorylation in human skeletal muscle	Hillier, T.; Long, W.; Jahn, L.; Wei, L.; Barrett, E. J.	2000	Did not perform fiber type analysis
Older adults with sarcopenia have distinct skeletal muscle phosphodiester, phosphocreatine, and phospholipid profiles	Hinkley, J. M.; Cornnell, H. H.; Standley, R. A.; Chen, E. Y.; Narain, N. R.; Greenwood, B. P.; Bussberg, V.; Tolstikov, V. V.; Kiebish, M. A.; Yi, F.; Vega, R. B.; Goodpaster, B. H.; Coen, P. M.	2020	Did not perform fiber type analysis
Roux-en-Y gastric bypass surgery enhances contraction-mediated glucose metabolism in primary human myotubes	Hinkley, J. M.; Zou, K.; Park, S.; Turner, K.; Zheng, D.; Houmard, J. A.	2017	No healthy subjects or controls
Human histocompatibility leukocyte antigen-DR and heat shock protein-70 expression in eye muscle tissue in thyroid-associated ophthalmopathy	Hiromatsu, Y.; Tanaka, K.; Ishisaka, N.; Kamachi, J.; Kuroki, T.; Hoshino, T.; Inoue, Y.; Wall, J. R.; Nonaka, K.	1995	Did not perform fiber type analysis
Effect of moderate acute exercise on expression of mRNA involved in the calcineurin signaling pathway in human skeletal muscle	Hitomi, Y.; Kizaki, T.; Katsumura, T.; Mizuno, M.; Itoh, C. E.; Esaki, K.; Fujioka, Y.; Takemasa, T.; Haga, S.; Ohno, H.	2003	Does not include males and females
Increased angiogenic response but deficient arteriolization and abnormal microvessel ultrastructure in critical leg ischaemia	Ho, T. K.; Rajkumar, V.; Black, C. M.; Abraham, D. J.; Baker, D. M.	2006	Does not include males and females
Increased endogenous angiogenic response and hypoxia-inducible factor-1alpha in human critical limb ischemia	Ho, T. K.; Rajkumar, V.; Ponticos, M.; Leoni, P.; Black, D. C.; Abraham, D. J.; Baker, D. M.	2006	Did not perform fiber type analysis
Angiogenic effects of stromal cell-derived factor-1 (SDF-1/CXCL12) variants in vitro and the in vivo expressions of CXCL12 variants and CXCR4 in human critical leg ischemia	Ho, T. K.; Tsui, J.; Xu, S.; Leoni, P.; Abraham, D. J.; Baker, D. M.	2010	Did not perform fiber type analysis
Quantitative analysis of dystrophin in fast- and slow-twitch mammalian skeletal muscle	Ho-Kim, M. A.; Rogers, P. A.	1992	Does not report sex of subjects
ABCA1 expression in humans is associated with physical activity and alcohol consumption	Hoang, A.; Tefft, C.; Duffy, S. J.; Formosa, M.; Henstridge, D. C.; Kingwell, B. A.; Sviridov, D.	2008	Does not include males and females
Loss of muscle oxidative capacity after an extreme endurance run: the Paris-Dakar foot-race	HÃ¶chli, D.; Schneiter, T.; Ferretti, G.; Howald, H.; Claassen, H.; Moia, C.; Atchou, G.; Belleri, M.; Veicsteinas, A.; Hoppeler, H.	1995	Does not include males and females
Characterisation of L-Type Amino Acid Transporter 1 (LAT1) Expression in Human Skeletal Muscle by Immunofluorescent Microscopy	Hodson, N.; Brown, T.; Joanisse, S.; Aguirre, N.; West, D. W. D.; Moore, D. R.; Baar, K.; Breen, L.; Philp, A.	2017	Does not include males and females
Human muscle proteome modifications after acute or repeated eccentric exercises	Hody, S.; Leprince, P.; Sergeant, K.; Renaut, J.; Croisier, J. L.; Wang, F.; Rogister, B.	2011	Does not include males and females

Early time course of change in angiogenic proteins in human skeletal muscle and vascular cells with endurance training	Hoier, B.; Olsen, K.; Hanskov, D. J. A.; Jorgensen, M.; Norup, L. R.; Hellsten, Y.	2020	Did not perform fiber type analysis
Subcellular localization and mechanism of secretion of vascular endothelial growth factor in human skeletal muscle	Hoier, B.; Prats, C.; Qvortrup, K.; Pilegaard, H.; Bangsbo, J.; Hellsten, Y.	2013	Did not perform fiber type analysis
Dysregulation of glycogen synthase COOH- and NH2-terminal phosphorylation by insulin in obesity and type 2 diabetes mellitus	HÃ¸jlund, K.; Birk, J. B.; Klein, D. K.; Levin, K.; Rose, A. J.; Hansen, B. F.; Nielsen, J. N.; Beck-Nielsen, H.; Wojtaszewski, J. F.	2009	Did not perform fiber type analysis
Reduced plasma adiponectin concentrations may contribute to impaired insulin activation of glycogen synthase in skeletal muscle of patients with type 2 diabetes	HÃ¸jlund, K.; Frystyk, J.; Levin, K.; Flyvbjerg, A.; Wojtaszewski, J. F.; Beck-Nielsen, H.	2006	Did not perform fiber type analysis
Human ATP synthase beta is phosphorylated at multiple sites and shows abnormal phosphorylation at specific sites in insulin-resistant muscle	HÃ¸jlund, K.; Yi, Z.; Lefort, N.; Langlais, P.; Bowen, B.; Levin, K.; Beck-Nielsen, H.; Mandarino, L. J.	2010	Did not perform fiber type analysis
Effect of needle biopsy from the vastus lateralis muscle on insulin-stimulated glucose metabolism in humans	Holck, P.; PÃ¸rksen, N.; Nielsen, M. F.; Nyholm, B.; Bak, J. F.; Andreasen, F.; MÃ¸ller, N.; Schmitz, O.	1994	Does not include males and females
Muscle GLUT4 in cirrhosis	Holland-Fischer, P.; Andersen, P. H.; Lund, S.; Pedersen, S. B.; Vinter-Jensen, L.; Nielsen, M. F.; Kaal, A.; Dall, R.; Schmitz, O.; Vilstrup, H.	2007	Did not perform fiber type analysis
Endothelial and myogenic differentiation of hematopoietic progenitor cells in inflammatory myopathies	Hollemann, D.; Budka, H.; LÃ¶scher, W. N.; Yanagida, G.; Fischer, M. B.; Wanschitz, J. V.	2008	Does not report sex of subjects
Effect of induced metabolic acidosis on human skeletal muscle metabolism during exercise	Hollidge-Horvat, M. G.; Parolin, M. L.; Wong, D.; Jones, N. L.; Heigenhauser, G. J.	1999	Does not include males and females
Effect of induced metabolic alkalosis on human skeletal muscle metabolism during exercise	Hollidge-Horvat, M. G.; Parolin, M. L.; Wong, D.; Jones, N. L.; Heigenhauser, G. J.	2000	Does not include males and females
Cardiomyopathy is common in patients with the mitochondrial DNA m.3243A>G mutation and correlates with mutation load	Hollingsworth, K. G.; Gorman, G. S.; Trenell, M. I.; McFarland, R.; Taylor, R. W.; Turnbull, D. M.; MacGowan, G. A.; Blamire, A. M.; Chinnery, P. F.	2012	Did not perform fiber type analysis
Linkage between postabsorptive amino acid release and glutamate uptake in skeletal muscle tissue of healthy young subjects, cancer patients, and the elderly	Holm, E.; Hack, V.; Tokus, M.; Breitkreutz, R.; Babylon, A.; DrÃ¶ge, W.	1997	Does not include males and females
In vitro metabolism of glucose by human skeletal muscle. Method and normal values	Holm, J.; SchérstÃ©n, T.	1972	No healthy subjects or controls
The single-biopsy approach in determining protein synthesis in human slow-turning-over tissue: use of flood-primed, continuous infusion of amino acid tracers	Holm, L.; Reitelseder, S.; Dideriksen, K.; Nielsen, R. H.; BÃ¼low, J.; Kjaer, M.	2014	Does not include males and females
Increased abundance of the adaptor protein containing pleckstrin homology domain, phosphotyrosine binding domain and leucine zipper motif (APPL1) in patients with obesity and	Holmes, R. M.; Yi, Z.; De Filippis, E.; Berria, R.; Shahani, S.; Sathyanarayana, P.; Sherman, V.; Fujiwara, K.; Meyer, C.; Christ-	2011	Did not perform fiber type analysis

type 2 diabetes: evidence for altered adiponectin signalling	Roberts, C.; Hwang, H.; Finlayson, J.; Dong, L. Q.; Mandarino, L. J.; Bajaj, M.		
A unique library of myogenic cells from facioscapulohumeral muscular dystrophy subjects and unaffected relatives: family, disease and cell function	Homma, S.; Chen, J. C.; Rahimov, F.; Beermann, M. L.; Hanger, K.; Bibat, G. M.; Wagner, K. R.; Kunkel, L. M.; Emerson, C. P., Jr.; Miller, J. B.	2012	Did not perform fiber type analysis
Activities of respiratory chain complexes and pyruvate dehydrogenase in isolated muscle mitochondria in premature neonates	Honzik, T.; Wenchich, L.; BÃ¶hm, M.; Hansikova, H.; Pejznochova, M.; Zapadlo, M.; Plavka, R.; Zeman, J.	2008	Only in children (0-17 years)
The inferior oblique as muscle of choice for biopsies of extraocular muscles	Hoogenraad, T. U.; Tan, K. E.; Eelderink, H. J.; Veldman, H.; Jennekens, F. G.	1979	No healthy subjects or controls
Endurance training in humans: aerobic capacity and structure of skeletal muscle	Hoppeler, H.; Howald, H.; Conley, K.; Lindstedt, S. L.; Claassen, H.; Vock, P.; Weibel, E. R.	1985	Does not include male and female data
Morphological adaptations of human skeletal muscle to chronic hypoxia	Hoppeler, H.; Kleinert, E.; Schlegel, C.; Claassen, H.; Howald, H.; Kayar, S. R.; Cerretelli, P.	1990	Does not include males and females
The ultrastructure of the normal human skeletal muscle. A morphometric analysis on untrained men, women and well-trained orienteers	Hoppeler, H.; LÃ¼thi, P.; Claassen, H.; Weibel, E. R.; Howald, H.	1973	Did not perform fiber type analysis
High efficiency of type I muscle fibers improves performance	Horowitz, J. F.; Sidossis, L. S.; Coyle, E. F.	1994	Does not include males and females
Metabolic basis to Sherpa altitude adaptation	Horscroft, J. A.; Kotwica, A. O.; Laner, V.; West, J. A.; Hennis, P. J.; Levett, D. Z. H.; Howard, D. J.; Fernandez, B. O.; Burgess, S. L.; Ament, Z.; Gilbert-Kawai, E. T.; Vercueil, A.; Landis, B. D.; Mitchell, K.; Mythen, M. G.; Branco, C.; Johnson, R. S.; Feelisch, M.; Montgomery, H. E.; Griffin, J. L.; Grocott, M. P. W.; Gnaiger, E.; Martin, D. S.; Murray, A. J.	2017	Did not perform fiber type analysis
Adaptive responses to muscle lengthening and shortening in humans	HortobÃ¡gyi, T.; Hill, J. P.; Houmard, J. A.; Fraser, D. D.; Lambert, N. J.; Israel, R. G.	1996	Does not include males and females
The effects of detraining on power athletes	HortobÃ¡gyi, T.; Houmard, J. A.; Stevenson, J. R.; Fraser, D. D.; Johns, R. A.; Israel, R. G.	1993	Does not include males and females
Abundant expression of myosin heavy-chain IIB RNA in a subset of human masseter muscle fibres	Horton, M. J.; Brandon, C. A.; Morris, T. J.; Braun, T. W.; Yaw, K. M.; Sciote, J. J.	2001	Does not include male and female data
Immunohistochemical staining of dystrophin on formalin-fixed paraffin-embedded sections in Duchenne/Becker muscular dystrophy and manifesting carriers of Duchenne muscular dystrophy	Hoshino, S.; Ohkoshi, N.; Watanabe, M.; Shoji, S.	2000	No healthy subjects or controls
Interrelationships between skeletal muscle adaptations and performance as studied by detraining and retraining	Houston, M. E.; Bentzen, H.; Larsen, H.	1979	Does not include males and females

Metabolic effects of two frequencies of short-term surface electrical stimulation on human muscle	Houston, M. E.; Farrance, B. W.; Wight, R. I.	1982	No cross-sectional area data
Myosin phosphorylation, twitch potentiation, and fatigue in human skeletal muscle	Houston, M. E.; Grange, R. W.	1990	Does not include male and female data
Myosin light chain phosphorylation in intact human muscle	Houston, M. E.; Lingley, M. D.; Stuart, D. S.; Grange, R. W.	1987	Did not perform fiber type analysis
Glycogen depletion and lactate responses in freestyle wrestling	Houston, M. E.; Sharratt, M. T.; Bruce, R. W.	1983	Does not include males and females
Human skeletal muscle mitochondrial dynamics in relation to oxidative capacity and insulin sensitivity	Houzelle, A.; JÃ¶rgensen, J. A.; Schaart, G.; Daemen, S.; van Polanen, N.; Fealy, C. E.; Hesselink, M. K. C.; Schrauwen, P.; Hoeks, J.	2021	Does not include males and females
Ultrastructure and biochemical function of skeletal muscle in twins	Howald, H.	1976	Did not perform fiber type analysis
Content of intramyocellular lipids derived by electron microscopy, biochemical assays, and (1)H-MR spectroscopy	Howald, H.; Boesch, C.; Kreis, R.; Matter, S.; Billeter, R.; Essen-Gustavsson, B.; Hoppeler, H.	2002	Did not perform fiber type analysis
Performing at extreme altitude: muscle cellular and subcellular adaptations	Howald, H.; Hoppeler, H.	2003	Review (ex: systematic review/meta-analysis)
Exercise training increases branched-chain oxoacid dehydrogenase kinase content in human skeletal muscle	Howarth, K. R.; Burgomaster, K. A.; Phillips, S. M.; Gibala, M. J.	2007	Did not perform fiber type analysis
Effects of dichloroacetate infusion on human skeletal muscle metabolism at the onset of exercise	Howlett, R. A.; Heigenhauser, G. J.; Hultman, E.; Hollidge-Horvat, M. G.; Spriet, L. L.	1999	Did not perform fiber type analysis
Regulation of skeletal muscle glycogen phosphorylase and PDH at varying exercise power outputs	Howlett, R. A.; Parolin, M. L.; Dyck, D. J.; Hultman, E.; Jones, N. L.; Heigenhauser, G. J.; Spriet, L. L.	1998	Did not perform fiber type analysis
The HSP expression of passive repetitive plyometric trained skeletal muscle	Hsu, C. C.; Hsu, M. C.; Huang, M. S.; Chen, C. S.; Shiang, T. Y.; Wang, C. H.; Chen, T.; Su, B.	2005	Does not report sex of subjects
Blood lactate threshold and type II fibre predominance in patients with exertional heatstroke	Hsu, Y. D.; Lee, W. H.; Chang, M. K.; Shieh, S. D.; Tsao, W. L.	1997	Does not include males and females
Electrical stimulation influences chronic intermittent hypoxia-hypercapnia induction of muscle fibre transformation by regulating the microRNA/Sox6 pathway	Huang, S.; Jin, L.; Shen, J.; Shang, P.; Jiang, X.; Wang, X.	2016	Animal study
Peroxisomal gene and protein expression increase in response to a high-lipid challenge in human skeletal muscle	Huang, T. Y.; Zheng, D.; Hickner, R. C.; Brault, J. J.; Cortright, R. N.	2019	Does not include males and females
Overexpression of PGC-1Î± increases peroxisomal activity and mitochondrial fatty acid oxidation in human primary myotubes	Huang, T. Y.; Zheng, D.; Houmard, J. A.; Brault, J. J.; Hickner, R. C.; Cortright, R. N.	2017	Does not include males and females

Down-regulation of insulin receptor substrates (IRS)-1 and IRS-2 and Src homologous and collagen-like protein Shc gene expression by insulin in skeletal muscle is not associated with insulin resistance or type 2 diabetes	Huang, X.; Vaag, A.; Hansson, M.; Groop, L.	2002	Did not perform fiber type analysis
Impaired insulin-stimulated expression of the glycogen synthase gene in skeletal muscle of type 2 diabetic patients is acquired rather than inherited	Huang, X.; Vaag, A.; Hansson, M.; Weng, J.; Laurila, E.; Groop, L.	2000	Did not perform fiber type analysis
The value of electron microscopy in muscle biopsies	Hudgson, P.	1970	Review (ex: systematic review/meta-analysis)
Human muscular mitochondrial fusion in athletes during exercise	Huertas, J. R.; Ruiz-Ojeda, F. J.; Plaza-DÃaz, J.; Nordsborg, N. B.; MartÃn-Albo, J.; Rueda-Robles, A.; Casuso, R. A.	2019	Does not include males and females
Molecular alterations in skeletal muscle in rheumatoid arthritis are related to disease activity, physical inactivity, and disability	Huffman, K. M.; Jessee, R.; Andonian, B.; Davis, B. N.; Narowski, R.; Huebner, J. L.; Kraus, V. B.; McCracken, J.; Gilmore, B. F.; Tune, K. N.; Campbell, M.; Koves, T. R.; Muoio, D. M.; Hubal, M. J.; Kraus, W. E.	2017	Did not perform fiber type analysis
Normal rates of whole-body fat oxidation and gluconeogenesis after overnight fasting and moderate-intensity exercise in patients with medium-chain acyl-CoA dehydrogenase deficiency	Huidekoper, H. H.; Ackermans, M. T.; Koopman, R.; van Loon, L. J.; Sauerwein, H. P.; Wijburg, F. A.	2013	Did not perform fiber type analysis
Training with low muscle glycogen enhances fat metabolism in well-trained cyclists	Hulston, C. J.; Venables, M. C.; Mann, C. H.; Martin, C.; Philp, A.; Baar, K.; Jeukendrup, A. E.	2010	Does not include males and females
Relationship between isometric endurance and fibre types in human leg muscles	HultÃ©n, B.; Thorstensson, A.; SjÃ¶din, B.; Karlsson, J.	1975	Does not report sex of subjects
Effect of induced metabolic acidosis on intracellular pH, buffer capacity and contraction force of human skeletal muscle	Hultman, E.; Del Canale, S.; SjÃ¶holm, H.	1985	Did not perform fiber type analysis
Energy metabolism and contraction force of human skeletal muscle in situ during electrical stimulation	Hultman, E.; SjÃ¶holm, H.	1983	Did not perform fiber type analysis
Skeletal muscle metabolism, contraction force and glycogen utilization during prolonged electrical stimulation in humans	Hultman, E.; Spriet, L. L.	1986	Did not perform fiber type analysis
Mechanical ventilation-induced diaphragm disuse in humans triggers autophagy	Hussain, S. N.; Mofarrahi, M.; Sigala, I.; Kim, H. C.; Vassilakopoulos, T.; Maltais, F.; Bellenis, I.; Chaturvedi, R.; Gottfried, S. B.; Metrakos, P.; Danialou, G.; Matecki, S.; Jaber, S.; Petrof, B. J.; Goldberg, P.	2010	Does not include males and females
A sustained increase in plasma NEFA upregulates the Toll-like receptor network in human muscle	Hussey, S. E.; Lum, H.; Alvarez, A.; Cipriani, Y.; GarduÃ±o-Garcia, J.; Anaya, L.; Dube, J.; Musi, N.	2014	Did not perform fiber type analysis
Exercise increases skeletal muscle GLUT4 gene expression in patients with type 2 diabetes	Hussey, S. E.; McGee, S. L.; Garnham, A.; McConell, G. K.; Hargreaves, M.	2012	Did not perform fiber type analysis

Effect of exercise on the skeletal muscle proteome in patients with type 2 diabetes	Hussey, S. E.; Sharoff, C. G.; Garnham, A.; Yi, Z.; Bowen, B. P.; Mandarino, L. J.; Hargreaves, M.	2013	Did not perform fiber type analysis
Oxidative stress and mitochondrial impairment can be separated from lipofuscin accumulation in aged human skeletal muscle	HÃ¼tter, E.; Skovbro, M.; Lener, B.; Prats, C.; RabÃ¸l, R.; Dela, F.; Jansen-DÃ¼rr, P.	2007	Did not perform fiber type analysis
Extracellular matrix remodeling and its contribution to protective adaptation following lengthening contractions in human muscle	Hyldahl, R. D.; Nelson, B.; Xin, L.; Welling, T.; Groscost, L.; Hubal, M. J.; Chipkin, S.; Clarkson, P. M.; Parcell, A. C.	2015	Did not perform fiber type analysis
Activation of nuclear factor-ÎºB following muscle eccentric contractions in humans is localized primarily to skeletal muscle-residing pericytes	Hyldahl, R. D.; Xin, L.; Hubal, M. J.; Moeckel-Cole, S.; Chipkin, S.; Clarkson, P. M.	2011	Does not include males and females
Tibialis anterior muscle needle biopsy and sensitive biomolecular methods: a useful tool in myotonic dystrophy type 1	Iachettini, S.; Valaperta, R.; Marchesi, A.; Perfetti, A.; Cuomo, G.; Fossati, B.; Vaienti, L.; Costa, E.; Meola, G.; Cardani, R.	2015	Does not include male and female data
Four weeks of speed endurance training reduces energy expenditure during exercise and maintains muscle oxidative capacity despite a reduction in training volume	Iaia, F. M.; Hellsten, Y.; Nielsen, J. J.; FernstrÃ¶m, M.; Sahlin, K.; Bangsbo, J.	2009	Does not include males and females
Relationship between performance at different exercise intensities and skeletal muscle characteristics	Iaia, F. M.; Perez-Gomez, J.; Thomassen, M.; Nordsborg, N. B.; Hellsten, Y.; Bangsbo, J.	2011	Does not include males and females
Partial biochemical maturation of aneurally cultured human skeletal muscle	Iannaccone, S. T.; Nagy, B.; Samaha, F. J.	1982	Only in children (0-17 years)
Twenty-four hour energy expenditure and skeletal muscle gene expression changes after bariatric surgery	Iesari, S.; le Roux, C. W.; De Gaetano, A.; Manco, M.; Nanni, G.; Mingrone, G.	2013	No healthy subjects or controls
Muscle Transcriptomics Shows Overexpression of Cadherin 1 in Inclusion Body Myositis	Ikenaga, C.; Date, H.; Kanagawa, M.; Mitsui, J.; Ishiura, H.; Yoshimura, J.; Pinal-Fernandez, I.; Mammen, A. L.; Lloyd, T. E.; Tsuji, S.; Shimizu, J.; Toda, T.; Goto, J.	2022	Did not perform fiber type analysis
Dysferlin expression in tubular aggregates: their possible relationship to endoplasmic reticulum stress	Ikezoe, K.; Furuya, H.; Ohyagi, Y.; Osoegawa, M.; Nishino, I.; Nonaka, I.; Kira, J.	2003	Does not report sex of subjects
Ultrastructural detection of DNA fragmentation in myonuclei of fatal reducing body myopathy	Ikezoe, K.; Nakagawa, M.; Osoegawa, M.; Kira, J.; Nonaka, I.	2004	Did not perform fiber type analysis
Effect of eccentric contraction on satellite cell activation in human vastus lateralis muscle	Imaoka, Y.; Kawai, M.; Mori, F.; Miyata, H.	2015	Does not include male and female data
Skeletal muscle 11Î²HSD1 activity of nondiabetic subjects is unaltered in central obesity-associated insulin resistance	Inder, W. J.; Obeyesekere, V. R.; Alford, F. P.; Jang, C.	2011	Did not perform fiber type analysis
Mini invasive skeletal muscle biopsy technique with a tri-axial end cut needle	Invernizzi, M.; Rizzi, M.; Carda, S.; Cisari, C.; Molinari, C.; RenÃ², F.	2015	Does not include males and females
Physiological hyperinsulinemia impairs insulin-stimulated glycogen synthase activity and glycogen synthesis	Iozzo, P.; Pratipanawatr, T.; Pijl, H.; Vogt, C.; Kumar, V.; Pipek, R.; Matsuda, M.; Mandarino, L. J.; Cusi, K. J.; DeFronzo, R. A.	2001	Did not perform fiber type analysis
Muscle biopsy investigations on neuromuscular insufficiency of the rotator cuff: a contribution	Irlenbusch, U.; Gansen, H. K.	2003	Does not include male and female data

to the functional impingement of the shoulder joint			
Immunoglobulin deposition in skeletal muscle in primary muscle diseases	Isenberg, D. A.	1983	Did not perform fiber type analysis
Results of in vitro contracture tests for the diagnosis of malignant hyperthermia susceptibility in monozygote twins	Islander, G.; Ranklev Twetman, E.	1997	No healthy subjects or controls
Inclusion body myositis: laser microdissection reveals differential up-regulation of IFN-Î³ signaling cascade in attacked versus nonattacked myofibers	Ivanidze, J.; Hoffmann, R.; LochmÃ¼ller, H.; Engel, A. G.; Hohlfeld, R.; Dornmair, K.	2011	Does not include males and females
Progressive metabolite changes in individual human muscle fibers with increasing work rates	Ivy, J. L.; Chi, M. M.; Hintz, C. S.; Sherman, W. M.; Hellendall, R. P.; Lowry, O. H.	1987	Does not include males and females
Post exercise carbohydrate-protein supplementation: phosphorylation of muscle proteins involved in glycogen synthesis and protein translation	Ivy, J. L.; Ding, Z.; Hwang, H.; Cialdella-Kam, L. C.; Morrison, P. J.	2008	Does not include males and females
Isokinetic contractile properties of the quadriceps with relation to fiber type	Ivy, J. L.; Withers, R. T.; Brose, G.; Maxwell, B. D.; Costill, D. L.	1981	Does not include males and females
Muscle respiratory capacity and fiber type as determinants of the lactate threshold	Ivy, J. L.; Withers, R. T.; Van Handel, P. J.; Elger, D. H.; Costill, D. L.	1980	Does not include males and females
Alpha B-crystallin in oxidative muscle fibers and its accumulation in ragged-red fibers: a comparative immunohistochemical and histochemical study in human skeletal muscle	Iwaki, T.; Iwaki, A.; Goldman, J. E.	1993	Does not report sex of subjects
Caveolin-3 is aberrantly expressed in skeletal muscle cells in myasthenia gravis	Iwasa, K.; Furukawa, Y.; Yoshikawa, H.; Yamada, M.	2016	Did not perform fiber type analysis
Nutritional status affects branched-chain oxoacid dehydrogenase activity during exercise in humans	Jackman, M. L.; Gibala, M. J.; Hultman, E.; Graham, T. E.	1997	Did not perform fiber type analysis
Coordinated alpha-crystallin B phosphorylation and desmin expression indicate adaptation and deadaptation to resistance exercise-induced loading in human skeletal muscle	Jacko, D.; Bersiner, K.; Schulz, O.; Przyklenk, A.; Spahiu, F.; HÃ¶hfeld, J.; Bloch, W.; Gehlert, S.	2020	Does not include males and females
Glutathione depletion during experimental damage to rat skeletal muscle and its relevance to Duchenne muscular dystrophy	Jackson, M. J.; Brooke, M. H.; Kaiser, K.; Edwards, R. H.	1991	Did not perform fiber type analysis
Lactate concentrations after short, maximal exercise at various glycogen levels	Jacobs, I.	1981	Does not include males and females
Muscle glycogen in soldiers on different diets during military field manoeuvres	Jacobs, I.; Anderberg, A.; SchÃ©le, R.; Lithell, H.	1983	Did not perform fiber type analysis
Relationship of ergometer-specific VO2 max and muscle enzymes to blood lactate during submaximal exercise	Jacobs, I.; SjÃ¶din, B.	1985	Does not include males and females

Lactate in human skeletal muscle after 10 and 30 s of supramaximal exercise	Jacobs, I.; Tesch, P. A.; Bar-Or, O.; Karlsson, J.; Dotan, R.	1983	Did not perform fiber type analysis
Mitochondrial function in human skeletal muscle following high-altitude exposure	Jacobs, R. A.; Boushel, R.; Wright-Paradis, C.; Calbet, J. A.; Robach, P.; Gnaiger, E.; Lundby, C.	2013	Does not include males and females
Twenty-eight days of exposure to 3454 m increases mitochondrial volume density in human skeletal muscle	Jacobs, R. A.; Lundby, A. K.; Fenk, S.; Gehrig, S.; Siebenmann, C.; FlÃ¼ck, D.; Kirk, N.; Hilty, M. P.; Lundby, C.	2016	Did not perform fiber type analysis
Mitochondria express enhanced quality as well as quantity in association with aerobic fitness across recreationally active individuals up to elite athletes	Jacobs, R. A.; Lundby, C.	2013	Does not include males and females
Lactate oxidation in human skeletal muscle mitochondria	Jacobs, R. A.; Meinild, A. K.; Nordsborg, N. B.; Lundby, C.	2013	Did not perform fiber type analysis
Twenty-eight days at 3454-m altitude diminishes respiratory capacity but enhances efficiency in human skeletal muscle mitochondria	Jacobs, R. A.; Siebenmann, C.; Hug, M.; Toigo, M.; Meinild, A. K.; Lundby, C.	2012	Does not include males and females
Skeletal muscle mRNA levels for cathepsin B, but not components of the ubiquitin-proteasome pathway, are increased in patients with lung cancer referred for thoracotomy	Jagoe, R. T.; Redfern, C. P.; Roberts, R. G.; Gibson, G. J.; Goodship, T. H.	2002	No healthy subjects or controls
Use of motor units in relation to muscle fiber type and size in man	Jakobsson, F.; Borg, K.; EdstrÃ¶m, L.; Grimby, L.	1988	Does not include male and female data
Motoneuron activity and muscle fibre type composition in hemiparesis	Jakobsson, F.; Grimby, L.; EdstrÃ¶m, L.	1992	Does not include male and female data
Long-term oxygen therapy may improve skeletal muscle metabolism in advanced chronic obstructive pulmonary disease patients with chronic hypoxaemia	Jakobsson, P.; Jorfeldt, L.	1995	Did not perform fiber type analysis
Skeletal muscle metabolites and fibre types in patients with advanced chronic obstructive pulmonary disease (COPD), with and without chronic respiratory failure	Jakobsson, P.; Jorfeldt, L.; Brundin, A.	1990	No healthy subjects or controls
Metabolic enzyme activity in the quadriceps femoris muscle in patients with severe chronic obstructive pulmonary disease	Jakobsson, P.; Jorfeldt, L.; Henriksson, J.	1995	Did not perform fiber type analysis
Reducing NF-ÎºB Signaling Nutritionally is Associated with Expedited Recovery of Skeletal Muscle Function After Damage	Jameson, T. S. O.; Pavis, G. F.; Dirks, M. L.; Lee, B. P.; Abdelrahman, D. R.; Murton, A. J.; Porter, C.; Alamdari, N.; Mikus, C. R.; Wall, B. T.; Stephens, F. B.	2021	Did not perform fiber type analysis
Intermediate filaments in the human extraocular muscles	Janbaz, A. H.; LindstrÃ¶m, M.; Liu, J. X.; Pedrosa DomellÃ¶f, F.	2014	Does not include males and females
Adiponectin, skeletal muscle adiponectin receptor expression and insulin resistance following dexamethasone	Jang, C.; Inder, W. J.; Obeyesekere, V. R.; Alford, F. P.	2008	Did not perform fiber type analysis

11Beta hydroxysteroid dehydrogenase type 1 is expressed and is biologically active in human skeletal muscle	Jang, C.; Obeyesekere, V. R.; Dilley, R. J.; Alford, F. P.; Inder, W. J.	2006	Does not include male and female data
Altered activity of 11beta-hydroxysteroid dehydrogenase types 1 and 2 in skeletal muscle confers metabolic protection in subjects with type 2 diabetes	Jang, C.; Obeyesekere, V. R.; Dilley, R. J.; Krozowski, Z.; Inder, W. J.; Alford, F. P.	2007	Did not perform fiber type analysis
Correlation of Utrophin Levels with the Dystrophin Protein Complex and Muscle Fibre Regeneration in Duchenne and Becker Muscular Dystrophy Muscle Biopsies	Janghra, N.; Morgan, J. E.; Sewry, C. A.; Wilson, F. X.; Davies, K. E.; Muntoni, F.; Tinsley, J.	2016	Only in children (0-17 years
Muscle 3243A-->G mutation load and capacity of the mitochondrial energy-generating system	Janssen, A. J.; Schuelke, M.; Smeitink, J. A.; Trijbels, F. J.; Sengers, R. C.; Lucke, B.; Wintjes, L. T.; Morava, E.; van Engelen, B. G.; Smits, B. W.; Hol, F. A.; Siers, M. H.; Ter Laak, H.; van der Knaap, M. S.; Van Spronsen, F. J.; Rodenburg, R. J.; van den Heuvel, L. P.	2008	Did not perform fiber type analysis
Measurement of the energy-generating capacity of human muscle mitochondria: diagnostic procedure and application to human pathology	Janssen, A. J.; Trijbels, F. J.; Sengers, R. C.; Wintjes, L. T.; Ruitenbeek, W.; Smeitink, J. A.; Morava, E.; van Engelen, B. G.; van den Heuvel, L. P.; Rodenburg, R. J.	2006	Did not perform fiber type analysis
Acid soluble and insoluble glycogen in human skeletal muscle	Jansson, E.	1981	Did not perform fiber type analysis
Calf muscle adaptation in intermittent claudication. Side-differences in muscle metabolic characteristics in patients with unilateral arterial disease	Jansson, E.; Johansson, J.; SylvÃ©n, C.; Kaijser, L.	1988	No healthy subjects or controls
Creatine kinase MB and citrate synthase in type I and type II muscle fibres in trained and untrained men	Jansson, E.; SylvÃ©n, C.	1985	Does not include males and females
Impaired muscle differentiation in explant cultures of Duchenne muscular dystrophy	Jasmin, G.; Tautu, C.; Vanasse, M.; Brochu, P.; Simoneau, R.	1984	Does not include males and females
Overexpression of von Hippel-Lindau protein in skeletal muscles of patients with chronic obstructive pulmonary disease	Jatta, K.; Eliason, G.; Portela-Gomes, G. M.; Grimelius, L.; Caro, O.; Nilholm, L.; SirjsÃ¶, A.; Piehl-Aulin, K.; Abdel-Halim, S. M.	2009	Does not include male and female data
Discrepancies between Skinned Single Muscle Fibres and Whole Thigh Muscle Function Characteristics in Young and Elderly Human Subjects	Jee, H.; Lim, J. Y.	2016	Single Fiber Analysis
Effect of acute exercise on glycogen synthase in muscle from obese and diabetic subjects	Jensen, J.; Tantiwong, P.; StuenÃ¦s, J. T.; Molina-Carrion, M.; DeFronzo, R. A.; Sakamoto, K.; Musi, N.	2012	Did not perform fiber type analysis
Histology and Function of the Rectus Abdominis Muscle in Patients With Incisional Hernia	Jensen, K. K.; Oma, E.; Kjaer, M.; JÃ¸rgensen, L. N.; Andersen, J. L.	2020	No healthy subjects or controls
Effect of high intensity training on capillarization and presence of angiogenic factors in human skeletal muscle	Jensen, L.; Bangsbo, J.; Hellsten, Y.	2004	Does not include males and females

Association between insulin resistance and impairment of FGF21 signal transduction in skeletal muscles	Jeon, J. Y.; Choi, S. E.; Ha, E. S.; Kim, T. H.; Jung, J. G.; Han, S. J.; Kim, H. J.; Kim, D. J.; Kang, Y.; Lee, K. W.	2016	Did not perform fiber type analysis
Cycle ergometry is not a sensitive diagnostic test for mitochondrial myopathy	Jeppesen, T. D.; Olsen, D.; Vissing, J.	2003	Did not perform fiber type analysis
Muscle phenotype and mutation load in 51 persons with the 3243A>G mitochondrial DNA mutation	Jeppesen, T. D.; Schwartz, M.; Frederiksen, A. L.; Wibrand, F.; Olsen, D. B.; Vissing, J.	2006	Did not perform fiber type analysis
Aerobic training is safe and improves exercise capacity in patients with mitochondrial myopathy	Jeppesen, T. D.; Schwartz, M.; Olsen, D. B.; Wibrand, F.; Krag, T.; DunÃ¸, M.; Hauerslev, S.; Vissing, J.	2006	Does not include male and female data
Human muscle fiber fine structure: morphometric data on controls	Jerusalem, F.; Engel, A. G.; Peterson, H. A.	1975	Did not perform fiber type analysis
Exercise increases TBC1D1 phosphorylation in human skeletal muscle	Jessen, N.; An, D.; Lihn, A. S.; Nygren, J.; Hirshman, M. F.; Thorell, A.; Goodyear, L. J.	2011	Did not perform fiber type analysi
Effects of carbohydrate (CHO) and fat supplementation on CHO metabolism during prolonged exercise	Jeukendrup, A. E.; Saris, W. H.; Brouns, F.; Halliday, D.; Wagenmakers, J. M.	1996	Does not include males and females
Growth Differentiation Factor 15 Is a Novel Diagnostic Biomarker of Mitochondrial Diseases	Ji, X.; Zhao, L.; Ji, K.; Zhao, Y.; Li, W.; Zhang, R.; Hou, Y.; Lu, J.; Yan, C.	2017	Does not include male and female data
Localized expression of specific P2X receptors in dystrophin-deficient DMD and mdx muscle	Jiang, T.; Yeung, D.; Lien, C. F.; GÃ³recki, D. C.	2005	No healthy subjects or controls
Mitochondrial morphology and MAVS-IFN1 signaling pathway in muscles of anti-MDA5 dermatomyositis	Jiang, Y.; Liu, Y.; Zhao, Y.; Zheng, Y.; Yu, M.; Deng, J.; Hao, H.; Zhang, W.; Wang, Z.; Yuan, Y.	2021	Did not perform fiber type analysis
Altered glycogen synthase and phosphorylase activities in skeletal muscle of tetraplegic patients	Jiao, Y.; Shashkin, P.; Hjeltnes, N.; Wallberg-Henriksson, H.; Katz, A.	2001	Does not include males and females
A new glycogen synthase activity ratio in skeletal muscle: effects of exercise and insulin	Jiao, Y.; Shashkin, P.; Katz, A.	2001	Did not perform fiber type analysis
Strong immunoreactivity of cathepsin L at the site of rimmed vacuoles in diseased muscles	Jimi, T.; Satoh, Y.; Takeda, A.; Shibuya, S.; Wakayama, Y.; Sugita, K.	1992	Case studies
Peripheral EphrinB1/EphB1 signalling attenuates muscle hyperalgesia in MPS patients and a rat model of taut band-associated persistent muscle pain	Jin, F.; Zhao, L.; Hu, Q.; Qi, F.	2020	Does not report sex of subjects
Increased SRF transcriptional activity in human and mouse skeletal muscle is a signature of insulin resistance	Jin, W.; Goldfine, A. B.; Boes, T.; Henry, R. R.; Ciaraldi, T. P.; Kim, E. Y.; Emecan, M.; Fitzpatrick, C.; Sen, A.; Shah, A.; Mun, E.; Vokes, V.; Schroeder, J.; Tatro, E.; Jimenez-Chillaron, J.; Patti, M. E.	2011	Did not perform fiber type analysis
Mitochondrial function in skeletal muscle of patients with protracted critical illness and ICU-acquired weakness	JiroutkovÃ¡, K.; KrajÄovÃ¡, A.; Ziak, J.; Fric, M.; Waldauf, P.; DÅ¾upa, V.; Gojda, J.; NÄ›mcova-FÃ¼rstovÃ¡, V.; KovÃ¡Å™, J.; Elkalaf, M.; Trnka, J.; DuÅ¡ka, F.	2015	No healthy subjects or controls

Skeletal muscle digoxin concentration and its relation to serum digoxin concentration and cardiac effect in healthy man	Jogestrand, T.; Sundqvist, K.	1981	Did not perform fiber type analysis
Insulin action on glucose transport in isolated skeletal muscle from patients with liver cirrhosis	Johansson, U.; Eriksson, L. S.; Galuska, D.; Zierath, J. R.; Wallberg-Henriksson, H.	1994	Did not perform fiber type analysis
Increases in Na+,K+-ATPase activity of erythrocytes and skeletal muscle after chronic ethanol consumption: evidence for reduced efficiency of the enzyme	Johnson, J. H.; Crider, B. P.	1989	Does not report sex of subjects
Immunocytochemical studies of cytochrome oxidase subunits in skeletal muscle of patients with partial cytochrome oxidase deficiencies	Johnson, M. A.; Kadenbach, B.; Droste, M.; Old, S. L.; Turnbull, D. M.	1988	Does not include male and female data
Deficiency of the syntrophins and alpha-dystrobrevin in patients with inherited myopathy	Jones, K. J.; Compton, A. G.; Yang, N.; Mills, M. A.; Peters, M. F.; Mowat, D.; Kunkel, L. M.; Froehner, S. C.; North, K. N.	2003	Does not report sex of subjects
Long- and medium-chain fatty acid oxidation is increased in exercise-trained human skeletal muscle	Jong-Yeon, K.; Hickner, R. C.; Dohm, G. L.; Houmard, J. A.	2002	Did not perform fiber type analysis
Mitochondrial respiratory chain dysfunction in various neuromuscular diseases	Jongpiputvanich, S.; Sueblinvong, T.; Norapucsunton, T.	2005	Did not perform fiber type analysis
Changes in plasma concentration of kynurenine following intake of branched-chain amino acids are not caused by alterations in muscle kynurenine metabolism	Jonsson, W. O.; Ponette, J.; Horwath, O.; Rydenstam, T.; SÃ¶derlund, K.; Ekblom, B.; Azzolini, M.; Ruas, J. L.; Blomstrand, E.	2022	Did not perform fiber type analysis
Binding of digoxin to slow- and fast-twitch skeletal muscle fibres	Joreteg, T.	1986	Does not include males and females
The impact of aging on mitochondrial function and biogenesis pathways in skeletal muscle of sedentary high- and low-functioning elderly individuals	Joseph, A. M.; Adhihetty, P. J.; Buford, T. W.; Wohlgemuth, S. E.; Lees, H. A.; Nguyen, L. M.; Aranda, J. M.; Sandesara, B. D.; Pahor, M.; Manini, T. M.; Marzetti, E.; Leeuwenburgh, C.	2012	Does not include male and female data
Mitochondrial abnormalities in myofibrillar myopathies	Joshi, P. R.; Hauburger, A.; Kley, R.; Claeys, K. G.; Schneider, I.; Kress, W.; Stoltenburg, G.; Weis, J.; Vorgerd, M.; Deschauer, M.; Hanisch, F.	2014	Did not perform fiber type analysis
Fiber-type-specific expression of essential (alkali) myosin light chains in human skeletal muscles	Jostarndt, K.; Puntschart, A.; Hoppeler, H.; Billeter, R.	1996	Review (ex: systematic review/meta-analysis)
Fibre-type specific expression of fast and slow essential myosin light chain mRNAs in trained human skeletal muscles	Jostarndt-FÃ¶gen, K.; Puntschart, A.; Hoppeler, H.; Billeter, R.	1998	Does not report sex of subjects
Histochemical changes in the multifidus in mechanical derangements of the spine	Jowett, R. L.; Fidler, M. W.; Troup, J. D.	1975	No healthy subjects or controls
Changes in the contractile material of human skeletal muscle following tendinous injury	JÃ³zsa, L.; BÃ¡lint, B. J.; VÃ¡ndor, E.	1979	No healthy subjects or controls

Effect of high-intensity intermittent training on lactate and H+ release from human skeletal muscle	Juel, C.; Klarskov, C.; Nielsen, J. J.; Krustrup, P.; Mohr, M.; Bangsbo, J.	2004	Does not include males and females
Kinetics of lactate transport in sarcolemmal giant vesicles obtained from human skeletal muscle	Juel, C.; Kristiansen, S.; Pilegaard, H.; Wojtaszewski, J.; Richter, E. A.	1994	Does not include males and females
Human skeletal muscle and erythrocyte proteins involved in acid-base homeostasis: adaptations to chronic hypoxia	Juel, C.; Lundby, C.; Sander, M.; Calbet, J. A.; Hall, Gv	2003	Does not include male and female data
Exercise-induced translocation of Na(+)-K(+) pump subunits to the plasma membrane in human skeletal muscle	Juel, C.; Nielsen, J. J.; Bangsbo, J.	2000	Does not include males and female
Exercise-induced increase in maximal in vitro Na-K-ATPase activity in human skeletal muscle	Juel, C.; Nordsborg, N. B.; Bangsbo, J.	2013	Does not include males and females
Effects of prolonged recombinant human erythropoietin administration on muscle membrane transport systems and metabolic marker enzymes	Juel, C.; Thomsen, J. J.; Rentsch, R. L.; Lundby, C.	2007	Does not include males and females
Central and peripheral circulation in relation to muscle-fibre composition in normo- and hyper-tensive man	Juhlin-Dannfelt, A.; Frisk-Holmberg, M.; Karlsson, J.; Tesch, P.	1979	Does not include males and females
Functional and molecular adaptations of quadriceps and hamstring muscles to blood flow restricted training in patients with ACL rupture	Kacin, A.; DrobniÄ, M.; MarÅ¡, T.; MiÅ¡, K.; PetriÄ, M.; Weber, D.; Tomc Å½argi, T.; MartinÄiÄ, D.; Pirkmajer, S.	2021	Did not perform fiber type analysis
Adaptation of human skeletal muscle to training and anabolic steroids	Kadi, F.	2000	Does not include male and female data
Cellular adaptation of the trapezius muscle in strength-trained athletes	Kadi, F.; Eriksson, A.; Holmner, S.; Butler-Browne, G. S.; Thornell, L. E.	1999	Does not report sex of subjects
Effects of one bout of endurance exercise on the expression of myogenin in human quadriceps muscle	Kadi, F.; Johansson, F.; Johansson, R.; SjÃ¶strÃ¶m, M.; Henriksson, J.	2004	Does not include males and females
Subcellular localisation and composition of intramuscular triacylglycerol influence insulin sensitivity in humans	Kahn, D.; Perreault, L.; Macias, E.; Zarini, S.; Newsom, S. A.; Strauss, A.; Kerege, A.; Harrison, K.; Snell-Bergeon, J.; Bergman, B. C.	2021	Did not perform fiber type analysis
Effects of muscle electrostimulation during simulated weightlessness	Kakurin, L. I.; Yegorov, B. B.; Il'ina, Y. I.; Cherepakhin, M. A.	1975	Does not include males and females
GLUT4 and UBC9 protein expression is reduced in muscle from type 2 diabetic patients with severe insulin resistance	Kampmann, U.; Christensen, B.; Nielsen, T. S.; Pedersen, S. B.; Ã˜rskov, L.; Lund, S.; MÃ¸ller, N.; Jessen, N.	2011	Did not perform fiber type analysis
Mechano Growth Factor E peptide (MGF-E), derived from an isoform of IGF-1, activates human muscle progenitor cells and induces an increase in their fusion potential at different ages	Kandalla, P. K.; Goldspink, G.; Butler-Browne, G.; Mouly, V.	2011	Did not perform fiber type analysis
Relationship between serum amyloid A level and Tanis/SelS mRNA expression in skeletal muscle and adipose tissue from healthy and type 2 diabetic subjects	Karlsson, H. K.; Tsuchida, H.; Lake, S.; Koistinen, H. A.; Krook, A.	2004	Does not include males and females

Muscle fibre types, ubiquinone content and exercise capacity in hypertension and effort angina	Karlsson, J.; Diamant, B.; Folkers, K.; Lund, B.	1991	Does not include males and females
Distribution of LDH isozymes in human skeletal muscle	Karlsson, J.; Frith, K.; SjÃ¶din, B.; Gollnick, P. D.; Saltin, B.	1974	Does not report sex of subject
Relevance of muscle fibre type to fatigue in short intense and prolonged exercise in man	Karlsson, J.; SjÃ¶din, B.; Jacobs, I.; Kaiser, P.	1981	No cross-sectional area data
The effects of partial chronic denervation on forearm metabolism	Karpati, G.; Klassen, G.; Tanser, P.	1979	Does not report sex of subjects
Tauroursodeoxycholic Acid may improve liver and muscle but not adipose tissue insulin sensitivity in obese men and women	Kars, M.; Yang, L.; Gregor, M. F.; Mohammed, B. S.; Pietka, T. A.; Finck, B. N.; Patterson, B. W.; Horton, J. D.; Mittendorfer, B.; Hotamisligil, G. S.; Klein, S.	2010	Did not perform fiber type analysis
Red and white muscle fibres in meniscectomy patients. Effects of postoperative physiotherapy	Karumo, I.; Rehunen, S.; NÃ¤veri, H.; Alho, A.	1977	Does not include male and female data
Insulin reduces plasma arginase activity in type 2 diabetic patients	Kashyap, S. R.; Lara, A.; Zhang, R.; Park, Y. M.; DeFronzo, R. A.	2008	Did not perform fiber type analysis
Insulin resistance is associated with impaired nitric oxide synthase activity in skeletal muscle of type 2 diabetic subjects	Kashyap, S. R.; Roman, L. J.; Lamont, J.; Masters, B. S.; Bajaj, M.; Suraamornkul, S.; Belfort, R.; Berria, R.; Kellogg, D. L., Jr.; Liu, Y.; DeFronzo, R. A.	2005	Did not perform fiber type analysis
Changes in serum protein characteristics of type I and type II muscle fibers after 400-m sprint	Kato, K.; Miyamura, M.; Yabe, K.; Kaimoto, H.; Katsumata, K.	1988	Does not include males and females
Smooth-to-striated muscle transition in human esophagus: an immunohistochemical study using fetal and adult materials	Katori, Y.; Cho, B. H.; Song, C. H.; Fujimiya, M.; Murakami, G.; Kawase, T.	2010	Autopsies
Method for the determination of the arteriovenous muscle protein balance during non-steady-state blood and muscle amino acid concentrations	Katsanos, C. S.; Chinkes, D. L.; Sheffield-Moore, M.; Aarsland, A.; Kobayashi, H.; Wolfe, R. R.	2005	Did not perform fiber type analysis
A high proportion of leucine is required for optimal stimulation of the rate of muscle protein synthesis by essential amino acids in the elderly	Katsanos, C. S.; Kobayashi, H.; Sheffield-Moore, M.; Aarsland, A.; Wolfe, R. R.	2006	Did not perform fiber type analysis
Differential responses of glycogen synthase to ischaemia and ischaemic contraction in human skeletal muscle	Katz, A.	1997	Does not include males and females
No change in insulin mediators in human skeletal muscle during isometric contraction or recovery	Katz, A.; Hultman, E.; Huang, L.; Villar-Palasi, C.; Larner, J.	1996	Does not include males and females
G-1,6-P2 in human skeletal muscle after isometric contraction	Katz, A.; Lee, A. D.	1988	Does not include males and females
Hexokinase kinetics in human skeletal muscle after hyperinsulinaemia, hyperglycaemia and hyperepinephrinaemia	Katz, A.; Raz, I.	1994	Does not include males and females

Rapid activation of glycogen synthase and protein phosphatase in human skeletal muscle after isometric contraction requires an intact circulation	Katz, A.; Raz, I.	1995	Did not perform fiber type analysis
Muscle ATP turnover rate during isometric contraction in humans	Katz, A.; Sahlin, K.; Henriksson, J.	1986	Does not include male and female data
Muscle ammonia metabolism during isometric contraction in humans	Katz, A.; Sahlin, K.; Henriksson, J.	1986	Does not include male and female data
Phosphofructokinase activity in human skeletal muscle: effects of euglycaemic hyperinsulinaemia and fasting	Katz, A.; Yan, Z.	1993	Does not include males and females
Long-term morphometric and immunohistochemical findings in human free microvascular muscle flaps	Kauhanen, M. S.; Lorenzetti, F.; Leivo, I. V.; Tukiainen, E.; Asko-Seljavaara, S. L.	2004	Does not include male and female data
Muscle fiber diameter and muscle type distribution following free microvascular muscle transfers: a prospective study	Kauhanen, M. S.; Salmi, A. M.; von Boguslawsky, E. K.; Leivo, I. V.; Asko-Seljavaara, S. L.	1998	No healthy subjects or controls
The influence of low versus high carbohydrate diet on a 45-min strenuous cycling exercise	Kavouras, S. A.; Troup, J. P.; Berning, J. R.	2004	Does not include males and females
Muscle ultrastructure and biochemistry of lowland Tibetans	Kayser, B.; Hoppeler, H.; Desplanches, D.; Marconi, C.; Broers, B.; Cerretelli, P.	1996	Does not report sex of subjects
Characterization of lobulated fibers in limb girdle muscular dystrophy type 2A by gene expression profiling	Keira, Y.; Noguchi, S.; Kurokawa, R.; Fujita, M.; Minami, N.; Hayashi, Y. K.; Kato, T.; Nishino, I.	2007	Did not perform fiber type analysis
Interleukin-6 production by contracting human skeletal muscle: autocrine regulation by IL-6	Keller, P.; Keller, C.; Carey, A. L.; Jauffred, S.; Fischer, C. P.; Steensberg, A.; Pedersen, B. K.	2003	Does not include males and females
Interleukin-6 receptor expression in contracting human skeletal muscle: regulating role of IL-6	Keller, P.; Penkowa, M.; Keller, C.; Steensberg, A.; Fischer, C. P.; Giralt, M.; Hidalgo, J.; Pedersen, B. K.	2005	Does not include males and females
Skeletal muscle fatty acid metabolism in association with insulin resistance, obesity, and weight loss	Kelley, D. E.; Goodpaster, B.; Wing, R. R.; Simoneau, J. A.	1999	Did not perform fiber type analysis
Dysfunction of mitochondria in human skeletal muscle in type 2 diabetes	Kelley, D. E.; He, J.; Menshikova, E. V.; Ritov, V. B.	2002	Did not perform fiber type analysis
Intracellular defects in glucose metabolism in obese patients with NIDDM	Kelley, D. E.; Mokan, M.; Mandarino, L. J.	1992	Does not include males and females
Metabolic pathways of glucose in skeletal muscle of lean NIDDM patients	Kelley, D. E.; Mokan, M.; Mandarino, L. J.	1993	Does not include males and females
Impaired free fatty acid utilization by skeletal muscle in non-insulin-dependent diabetes mellitus	Kelley, D. E.; Simoneau, J. A.	1994	Does not include male and female data
Strength, skeletal muscle composition, and enzyme activity in multiple sclerosis	Kent-Braun, J. A.; Ng, A. V.; Castro, M.; Weiner, M. W.; Gelinas, D.; Dudley, G. A.; Miller, R. G.	1997	Does not include male and female data

A single bout of whole-leg, peristaltic pulse external pneumatic compression upregulates PGC-1Î± mRNA and endothelial nitric oxide sythase protein in human skeletal muscle tissue	Kephart, W. C.; Mobley, C. B.; Fox, C. D.; Pascoe, D. D.; Sefton, J. M.; Wilson, T. J.; Goodlett, M. D.; Kavazis, A. N.; Roberts, M. D.; Martin, J. S.	2015	Did not perform fiber type analysis
Insulin-like and fibroblast growth factors in spinal cords, nerve roots and skeletal muscle of human controls and patients with amyotrophic lateral sclerosis	Kerkhoff, H.; Hassan, S. M.; Troost, D.; Van Etten, R. W.; Veldman, H.; Jennekens, F. G.	1994	Autopsies
Atrophy/hypertrophy cell signaling in muscles of young athletes trained with vibrational-proprioceptive stimulation	Kern, H.; Pelosi, L.; Coletto, L.; MusarÃ², A.; Sandri, M.; Vogelauer, M.; Trimmel, L.; Cvecka, J.; Hamar, D.; Kovarik, J.; LÃ¶fler, S.; Sarabon, N.; Protasi, F.; Adami, N.; Biral, D.; Zampieri, S.; Carraro, U.	2011	Does not include males and females
Age-related changes in human thyroarytenoid muscles: a histological and histochemical study	Kersing, W.; Jennekens, F. G.	2004	Autopsies
Dysferlin deficiency shows compensatory induction of Rab27A/Slp2a that may contribute to inflammatory onset	Kesari, A.; Fukuda, M.; Knoblach, S.; Bashir, R.; Nader, G. A.; Rao, D.; Nagaraju, K.; Hoffman, E. P.	2008	Does not include males and females
Increased biological relevance of transcriptome analyses in human skeletal muscle using a model-specific pipeline	Khan, Y.; HammarstrÃ¶m, D.; RÃ¸nnestad, B. R.; Ellefsen, S.; Ahmad, R.	2020	Does not include male and female data
Time course of responses of human skeletal muscle to oxidative stress induced by nondamaging exercise	Khassaf, M.; Child, R. B.; McArdle, A.; Brodie, D. A.; Esanu, C.; Jackson, M. J.	2001	Does not include males and females
An ultrastructural study of multifidus muscle in progressive idiopathic scoliosis. Changes resulting from a sarcolemmal defect at the myotendinous junction	Khosla, S.; Tredwell, S. J.; Day, B.; Shinn, S. L.; Ovalle, W. K., Jr.	1980	Does not include males and females
Insulin resistance is associated with reduced fasting and insulin-stimulated glycogen synthase phosphatase activity in human skeletal muscle	Kida, Y.; Esposito-Del Puente, A.; Bogardus, C.; Mott, D. M.	1990	Did not perform fiber type analysis
Contraction-mediated inactivation of glycogen synthase is accompanied by inactivation of glycogen synthase phosphatase in human skeletal muscle	Kida, Y.; Katz, A.; Lee, A. D.; Mott, D. M.	1989	Does not include males and females
Defective insulin response of phosphorylase phosphatase in insulin-resistant humans	Kida, Y.; Raz, I.; Maeda, R.; Nyomba, B. L.; Stone, K.; Bogardus, C.; Sommercorn, J.; Mott, D. M.	1992	Did not perform fiber type analysis
Muscular long-chain fatty acid content during graded exercise in humans	Kiens, B.; Roemen, T. H.; van der Vusse, G. J.	1999	Does not include males and females
Sporadic inclusion body myositis: clinical, pathological, and genetic analysis of eight Polish patients	Kierdaszuk, B.; Berdynski, M.; Palczewski, P.; Golebiowski, M.; Zekanowski, C.; Kaminska, A. M.	2015	Does not include males and females
Regulation of PDH in human arm and leg muscles at rest and during intense exercise	Kiilerich, K.; Birk, J. B.; Damsgaard, R.; Wojtaszewski, J. F.; Pilegaard, H.	2008	Does not include males and females
Impact of low-volume concurrent strength training distribution on muscular adaptation	Kilen, A.; Bay, J.; Bejder, J.; Breenfeldt Andersen, A.; Bonne, T. C.; Larsen, P. D.; Carlsen, A.; Egelund, J.; Nybo, L.; Mackey, A. L.; Olsen, N. V.; Aachmann-Andersen, N. J.; Andersen, J. L.; Nordsborg, N. B.	2020	Does not include male and female data

Amino acids stimulate leg muscle protein synthesis in peripheral arterial disease	Killewich, L. A.; Tuvdendorj, D.; Bahadorani, J.; Hunter, G. C.; Wolfe, R. R.	2007	Did not perform fiber type analysis
Intramuscular nerve distribution of the masseter muscle as a basis for botulinum toxin injection	Kim, D. H.; Hong, H. S.; Won, S. Y.; Kim, H. J.; Hu, K. S.; Choi, J. H.; Kim, H. J.	2010	Autopsies
Impact of heat therapy on recovery after eccentric exercise in humans	Kim, K.; Kuang, S.; Song, Q.; Gavin, T. P.; Roseguini, B. T.	2019	Does not include male and female data
IL-6 induction of TLR-4 gene expression via STAT3 has an effect on insulin resistance in human skeletal muscle	Kim, T. H.; Choi, S. E.; Ha, E. S.; Jung, J. G.; Han, S. J.; Kim, H. J.; Kim, D. J.; Kang, Y.; Lee, K. W.	2013	Did not perform fiber type analysis
Anti-TIF1Î³ antibody and the expression of TIF1Î³ in idiopathic inflammatory myopathies	Kim, Y.; Song, K. S.; Sohn, E. H.; Kang, S. W.; Yoo, I. S.; Shim, S. C.; Yoo, S. J.; Kim, J.	2019	Did not perform fiber type analysis
Insulin-stimulated protein kinase C lambda/zeta activity is reduced in skeletal muscle of humans with obesity and type 2 diabetes: reversal with weight reduction	Kim, Y. B.; Kotani, K.; Ciaraldi, T. P.; Henry, R. R.; Kahn, B. B.	2003	Does not include males and females
Normal insulin-dependent activation of Akt/protein kinase B, with diminished activation of phosphoinositide 3-kinase, in muscle in type 2 diabetes	Kim, Y. B.; Nikoulina, S. E.; Ciaraldi, T. P.; Henry, R. R.; Kahn, B. B.	1999	Does not include males and females
Local restoration of dystrophin expression with the morpholino oligomer AVI-4658 in Duchenne muscular dystrophy: a single-blind, placebo-controlled, dose-escalation, proof-of-concept study	Kinali, M.; Arechavala-Gomeza, V.; Feng, L.; Cirak, S.; Hunt, D.; Adkin, C.; Guglieri, M.; Ashton, E.; Abbs, S.; Nihoyannopoulos, P.; Garralda, M. E.; Rutherford, M.; McCulley, C.; Popplewell, L.; Graham, I. R.; Dickson, G.; Wood, M. J.; Wells, D. J.; Wilton, S. D.; Kole, R.; Straub, V.; Bushby, K.; Sewry, C.; Morgan, J. E.; Muntoni, F.	2009	Only in children (0-17 years)
Dietary fat and carbohydrates differentially alter insulin sensitivity during caloric restriction	Kirk, E.; Reeds, D. N.; Finck, B. N.; Mayurranjan, S. M.; Patterson, B. W.; Klein, S.	2009	Did not perform fiber type analysis
Galalpha1-->4Gal-glycans are expressed on myofibrillar associated proteins	Kirkeby, S.; Moe, D.; ClÃ¤esson, M. H.	1998	Autopsies
Localized agglutinin staining in muscle capillaries from normal and very old atrophic human muscle using winged bean (Psophocarpus tetragonolobus) lectin	Kirkeby, S.; Singha, N. C.; Surolia, A.	1997	Did not perform fiber type analysis
Hypertrophic cardiomyopathy-related beta-myosin mutations cause highly variable calcium sensitivity with functional imbalances among individual muscle cells	Kirschner, S. E.; Becker, E.; Antognozzi, M.; Kubis, H. P.; Francino, A.; Navarro-LÃ³pez, F.; Bit-Avragim, N.; Perrot, A.; Mirrakhimov, M. M.; Osterziel, K. J.; McKenna, W. J.; Brenner, B.; Kraft, T.	2005	Did not perform fiber type analysis
The transcription factor Prox1 is essential for satellite cell differentiation and muscle fibre-type regulation	KivelÃ¤, R.; Salmela, I.; Nguyen, Y. H.; Petrova, T. V.; Koistinen, H. A.; Wiener, Z.; Alitalo, K.	2016	Does not report sex of subjects
Adrenaline and glycogenolysis in skeletal muscle during exercise: a study in adrenalectomised humans	Kjaer, M.; Howlett, K.; Langfort, J.; Zimmerman-Belsing, T.; Lorentsen, J.; Bulow, J.; Ihlemann, J.; Feldt-Rasmussen, U.; Galbo, H.	2000	Did not perform fiber type analysis

Oxygen conserving mitochondrial adaptations in the skeletal muscles of breath hold divers	Kjeld, T.; Stride, N.; Gudiksen, A.; Hansen, E. G.; Arendrup, H. C.; Horstmann, P. F.; Zerahn, B.; Jensen, L. T.; Nordsborg, N.; Bejder, J.; Halling, J. F.	2018	Does not include males and females
Effect of thyroid function on number of Na-K pumps in human skeletal muscle	Kjeldsen, K.; NÃ¸rgaard, A.; GÃ¸tzsche, C. O.; Thomassen, A.; Clausen, T.	1984	Did not perform fiber type analysis
Human skeletal muscle Na, K-ATPase concentration quantified by 3H-ouabain binding to intact biopsies before and after moderate physical conditioning	Kjeldsen, K.; NÃ¸rgaard, A.; Hau, C.	1990	Does not include males and females
Sarco(endo)plasmic reticulum Ca2+ ATPases (SERCA1 and -2) in human extraocular muscles	Kjellgren, D.; Ryan, M.; Ohlendieck, K.; Thornell, L. E.; Pedrosa-DomellÃ¶f, F.	2003	Autopsies
Uncoordinated expression of myosin heavy chains and myosin-binding protein C isoforms in human extraocular muscles	Kjellgren, D.; StÃ¥l, P.; Larsson, L.; FÃ¼rst, D.; Pedrosa-DomellÃ¶f, F.	2006	Autopsies
Myosin heavy chain isoforms in human extraocular muscles	Kjellgren, D.; Thornell, L. E.; Andersen, J.; Pedrosa-DomellÃ¶f, F.	2003	Autopsies
Proteasome proteolytic activity in skeletal muscle is increased in patients with sepsis	Klaude, M.; Fredriksson, K.; TjÃ¤der, I.; Hammarqvist, F.; Ahlman, B.; Rooyackers, O.; Wernerman, J.	2007	Did not perform fiber type analysis
Acute metabolic acidosis decreases muscle protein synthesis but not albumin synthesis in humans	Kleger, G. R.; Turgay, M.; Imoberdorf, R.; McNurlan, M. A.; Garlick, P. J.; Ballmer, P. E.	2001	Does not include males and females
Sarcoplasmic reticulum of human skeletal muscle: age-related changes and effect of training	Klitgaard, H.; Ausoni, S.; Damiani, E.	1989	Does not include males and females
Comprehensive expression analysis of FSHD candidate genes at the mRNA and protein level	Klooster, R.; Straasheijm, K.; Shah, B.; Sowden, J.; Frants, R.; Thornton, C.; Tawil, R.; van der Maarel, S.	2009	Did not perform fiber type analysis
Fibroregulation of mesenchymal progenitor cells by BMP-4 after traumatic muscle injury	Kluk, M. W.; Ji, Y.; Shin, E. H.; Amrani, O.; Onodera, J.; Jackson, W. M.; Nesti, L. J.	2012	No healthy subjects or controls
PD1 pathway in immune-mediated myopathies: Pathogenesis of dysfunctional T cells revisited	Knauss, S.; Preusse, C.; Allenbach, Y.; Leonard-Louis, S.; Touat, M.; Fischer, N.; Radbruch, H.; Mothes, R.; Matyash, V.; BÃ¶hmerle, W.; Endres, M.; Goebel, H. H.; Benveniste, O.; Stenzel, W.	2019	Review (ex: systematic review/meta-analysis)
Increased Myogenic and Protein Turnover Signaling in Skeletal Muscle of Chronic Obstructive Pulmonary Disease Patients With Sarcopenia	Kneppers, A. E. M.; Langen, R. C. J.; Gosker, H. R.; Verdijk, L. B.; Cebron Lipovec, N.; Leermakers, P. A.; Kelders, Mcjm; de Theije, C. C.; Omersa, D.; Lainscak, M.; Schols, Amwj	2017	Did not perform fiber type analysis
Exercise increases phosphorylation of the putative mTORC2 activity readout NDRG1 in human skeletal muscle	Knudsen, J. R.; Persson, K. W.; Meister, J.; Carl, C. S.; Raun, S. H.; Andersen, N. R.; Sylow, L.; Kiens, B.; Jensen, T. E.; Richter, E. A.; Kleinert, M.	2022	Does not include males and females
Possible involvement of Fas-mediated apoptosis in eye muscle tissue from patients with thyroid-associated ophthalmopathy	Koga, M.; Hiromatsu, Y.; Jimi, A.; Inoue, Y.; Nonaka, K.	1998	No healthy subjects or controls

Pronounced limb and fibre type differences in subcellular lipid droplet content and distribution in elite skiers before and after exhaustive exercise	Koh, H. E.; Nielsen, J.; Saltin, B.; Holmberg, H. C.; Ã˜rtenblad, N.	2017	Does not include males and females
Heterogeneity in insulin-stimulated glucose uptake among different muscle groups in healthy lean people and people with obesity	Koh, H. E.; van Vliet, S.; Meyer, G. A.; Laforest, R.; Gropler, R. J.; Klein, S.; Mittendorfer, B.	2021	Does not include male and female data
Mutation of the myosin converter domain alters cross-bridge elasticity	KÃ¶hler, J.; Winkler, G.; Schulte, I.; Scholz, T.; McKenna, W.; Brenner, B.; Kraft, T.	2002	No healthy subjects or controls
Fiber type and metabolic characteristics of lion (Panthera leo), caracal (Caracal caracal) and human skeletal muscle	Kohn, T. A.; Burroughs, R.; Hartman, M. J.; Noakes, T. D.	2011	Does not include males and females
Elevated levels of amyloid precursor protein in muscle of patients with amyotrophic lateral sclerosis and a mouse model of the disease	Koistinen, H.; Prinjha, R.; Soden, P.; Harper, A.; Banner, S. J.; Pradat, P. F.; Loeffler, J. P.; Dingwall, C.	2006	No healthy subjects or controls
Low frequency of mtDNA point mutations in patients with PEO associated with POLG1 mutations	Kollberg, G.; Jansson, M.; PÃ©rez-Bercoff, A.; Melberg, A.; Lindberg, C.; Holme, E.; Moslemi, A. R.; Oldfors, A.	2005	Does not include males and females
Muscle metabolism, lactate breaking point, and biomechanical features of endurance running	Komi, P. V.; Ito, A.; SjÃ¶din, B.; Wallenstein, R.; Karlsson, J.	1981	Does not include males and females
Skeletal muscle fibre types, enzyme activities and physical performance in young males and females	Komi, P. V.; Karlsson, J.	1978	Only in children (0-17 years)
Skeletal muscle fibres and muscle enzyme activities in monozygous and dizygous twins of both sexes	Komi, P. V.; Viitasalo, J. H.; Havu, M.; Thorstensson, A.; SjÃ¶din, B.; Karlsson, J.	1977	No cross-sectional area data
MicroRNA-1 and microRNA-206 improve differentiation potential of human satellite cells: a novel approach for tissue engineering of skeletal muscle	Koning, M.; Werker, P. M.; van der Schaft, D. W.; Bank, R. A.; Harmsen, M. C.	2012	No healthy subjects or controls
Failed upregulation of TFAM protein and mitochondrial DNA in oxidatively deficient fibers of chronic obstructive pulmonary disease locomotor muscle	Konokhova, Y.; Spendiff, S.; Jagoe, R. T.; Aare, S.; Kapchinsky, S.; MacMillan, N. J.; Rozakis, P.; Picard, M.; Aubertin-Leheudre, M.; Pion, C. H.; Bourbeau, J.; Hepple, R. T.; Taivassalo, T.	2016	Does not include males and females
Transcriptional levels of growth factors in skeletal muscle of maintenance hemodialysis patients	Kopple, J. D.; Wang, H.; Fournier, M.; Storer, T.; Zhang, S. M.; Song, H. Y.; Lewis, M.	2006	Did not perform fiber type analysis
Level of skeletal muscle glucose transporter protein correlates with insulin-stimulated whole body glucose disposal in man	Koranyi, L. I.; Bourey, R. E.; Vuorinen-Markkola, H.; Koivisto, V. A.; Mueckler, M.; Permutt, M. A.; Yki-JÃ¤rvinen, H.	1991	Does not include males and females
Myosin heavy-chain isoform composition of human single jaw-muscle fibers	Korfage, J. A.; Van Eijden, T. M.	2003	Autopsies
Myosin heavy chain composition in human masticatory muscles by immunohistochemistry and gel electrophoresis	Korfage, J. A.; Van Eijden, T. M.	2003	Autopsies
Effects of immunosuppressive treatment on microsomal prostaglandin E synthase 1 and cyclooxygenases expression in muscle tissue of patients with polymyositis or dermatomyositis	Korotkova, M.; Helmers, S. B.; Loell, I.; Alexanderson, H.; Grundtman, C.; Dorph, C.; Lundberg, I. E.; Jakobsson, P. J.	2008	No healthy subjects or controls

Increased fat accumulation in liver may link insulin resistance with subcutaneous abdominal adipocyte enlargement, visceral adiposity, and hypoadiponectinemia in obese individuals	Koska, J.; Stefan, N.; Permana, P. A.; Weyer, C.; Sonoda, M.; Bogardus, C.; Smith, S. R.; Joanisse, D. R.; Funahashi, T.; Krakoff, J.; Bunt, J. C.	2008	Did not perform fiber type analysis
Type IV collagen and its degradation in paralyzed human muscle: effect of functional electrical stimulation	Koskinen, S. O.; Kjaer, M.; Mohr, T.; SÃ¸rensen, F. B.; Suuronen, T.; Takala, T. E.	2000	Did not perform fiber type analysis
Correlations of Calf Muscle Macrophage Content With Muscle Properties and Walking Performance in Peripheral Artery Disease	Kosmac, K.; Gonzalez-Freire, M.; McDermott, M. M.; White, S. H.; Walton, R. G.; Sufit, R. L.; Tian, L.; Li, L.; Kibbe, M. R.; Criqui, M. H.; Guralnik, J. M.; S. Polonsky T; Leeuwenburgh, C.; Ferrucci, L.; Peterson, C. A.	2020	Does not include male and female data
Abnormal myofiber morphology and limb dysfunction in claudication	Koutakis, P.; Myers, S. A.; Cluff, K.; Ha, D. M.; Haynatzki, G.; McComb, R. D.; Uchida, K.; Miserlis, D.; Papoutsi, E.; Johanning, J. M.; Casale, G. P.; Pipinos,, II	2015	Does not include male and female data
Oxidative damage in the gastrocnemius of patients with peripheral artery disease is myofiber type selective	Koutakis, P.; Weiss, D. J.; Miserlis, D.; Shostrom, V. K.; Papoutsi, E.; Ha, D. M.; Carpenter, L. A.; McComb, R. D.; Casale, G. P.; Pipinos,, II	2014	Does not include male and female data
Regulation of hexokinase II activity and expression in human muscle by moderate exercise	Koval, J. A.; DeFronzo, R. A.; O'Doherty, R. M.; Printz, R.; Ardehali, H.; Granner, D. K.; Mandarino, L. J.	1998	Did not perform fiber type analysis
Differential expression of microRNAs and other small RNAs in muscle tissue of patients with ALS and healthy age-matched controls	Kovanda, A.; Leonardis, L.; Zidar, J.; Koritnik, B.; Dolenc-Groselj, L.; Ristic Kovacic, S.; Curk, T.; Rogelj, B.	2018	Did not perform fiber type analysis
Hypothalamic-pituitary-adrenal responses to short-duration high-intensity cycle exercise	Kraemer, W. J.; Patton, J. F.; Knuttgen, H. G.; Marchitelli, L. J.; Cruthirds, C.; Damokosh, A.; Harman, E.; Frykman, P.; Dziados, J. E.	1989	Does not include males and females
Familial hypertrophic cardiomyopathy: functional effects of myosin mutation R723G in cardiomyocytes	Kraft, T.; Witjas-Paalberends, E. R.; Boontje, N. M.; Tripathi, S.; Brandis, A.; Montag, J.; Hodgkinson, J. L.; Francino, A.; Navarro-Lopez, F.; Brenner, B.; Stienen, G. J.; van der Velden, J.	2013	Did not perform muscle biopsy
Autophagy is affected in patients with hypokalemic periodic paralysis: an involvement in vacuolar myopathy?	Krag, T. O.; Holm-Yildiz, S.; Witting, N.; Vissing, J.	2021	Did not perform fiber type analysis
Human skeletal muscle fibre type variations correlate with PPAR alpha, PPAR delta and PGC-1 alpha mRNA	KrÃ¤mer, D. K.; AhlsÃ©n, M.; Norrbom, J.; Jansson, E.; Hjeltnes, N.; Gustafsson, T.; Krook, A.	2006	Does not include males and females
Adenosine Triphosphate Production of Muscle Mitochondria after Acute Exercise in Lean and Obese Humans	Kras, K. A.; Hoffman, N.; Roust, L. R.; Benjamin, T. R.; D. E. Filippis EA; Katsanos, C. S.	2019	Did not perform fiber type analysis
Obesity modifies the stoichiometry of mitochondrial proteins in a way that is distinct to the subcellular localization of the mitochondria in skeletal muscle	Kras, K. A.; Langlais, P. R.; Hoffman, N.; Roust, L. R.; Benjamin, T. R.; De Filippis, E. A.; Dinu, V.; Katsanos, C. S.	2018	Did not perform fiber type analysis
Mitochondrial changes in skeletal muscle in amyotrophic lateral sclerosis and other neurogenic atrophies	Krasnianski, A.; Deschauer, M.; Neudecker, S.; Gellerich, F. N.; MÃ¼ller, T.; Schoser, B. G.; Krasnianski, M.; Zierz, S.	2005	Did not perform fiber type analysis

Muscle fibre type composition in infant and adult populations and relationships with obesity	Kriketos, A. D.; Baur, L. A.; O'Connor, J.; Carey, D.; King, S.; Caterson, I. D.; Storlien, L. H.	1997	Does not include male and female data
Ceramide content is higher in type I compared to type II fibers in obesity and type 2 diabetes mellitus	Kristensen, D.; Prats, C.; Larsen, S.; Ara, I.; Dela, F.; Helge, J. W.	2013	Does not include males and females
A PGC-1Î±- and muscle fibre type-related decrease in markers of mitochondrial oxidative metabolism in skeletal muscle of humans with inherited insulin resistance	Kristensen, J. M.; Skov, V.; Petersson, S. J.; Ã˜rtenblad, N.; Wojtaszewski, J. F.; Beck-Nielsen, H.; HÃ¸jlund, K.	2014	Does not include male and female data
Alpine Skiing With total knee ArthroPlasty (ASWAP): metabolism, inflammation, and skeletal muscle fiber characteristics	Kristensen, M.; PÃ¶tzelsberger, B.; Scheiber, P.; Bergdahl, A.; Hansen, C. N.; Andersen, J. L.; Narici, M.; Salvioli, S.; Conte, M.; MÃ¼ller, E.; Dela, F.	2015	Does not include male and female data
Obesity leads to impairments in the morphology and organization of human skeletal muscle lipid droplets and mitochondrial networks, which are resolved with gastric bypass surgery-induced improvements in insulin sensitivity	Kristensen, M. D.; Petersen, S. M.; MÃ¸ller, K. E.; Lund, M. T.; Hansen, M.; Hansen, C. N.; Courraud, J.; Helge, J. W.; Dela, F.; Prats, C.	2018	Does not include males and females
Fructose transport and GLUT-5 protein in human sarcolemmal vesicles	Kristiansen, S.; Darakhshan, F.; Richter, E. A.; Hundal, H. S.	1997	Does not include males and females
Preservation of in vitro muscle fiber function in dermatomyositis and inclusion body myositis: a single fiber study	Krivickas, L. S.; Amato, A. A.; Krishnan, G.; Murray, A. V.; Frontera, W. R.	2005	Does not include male and female data
Contractile properties of single muscle fibers in myotonic dystrophy	Krivickas, L. S.; Ansved, T.; Suh, D.; Frontera, W. R.	2000	Does not include male and female data
Relationship between force and size in human single muscle fibres	Krivickas, L. S.; Dorer, D. J.; Ochala, J.; Frontera, W. R.	2011	Does not include male and female data
Insulin stimulates interleukin-6 and tumor necrosis factor-alpha gene expression in human subcutaneous adipose tissue	Krogh-Madsen, R.; Plomgaard, P.; Keller, P.; Keller, C.; Pedersen, B. K.	2004	Does not include males and females
Influence of TNF-alpha and IL-6 infusions on insulin sensitivity and expression of IL-18 in humans	Krogh-Madsen, R.; Plomgaard, P.; MÃ¸ller, K.; Mittendorfer, B.; Pedersen, B. K.	2006	Does not include males and females
Effects of exercise on mitogen- and stress-activated kinase signal transduction in human skeletal muscle	Krook, A.; Widegren, U.; Jiang, X. J.; Henriksson, J.; Wallberg-Henriksson, H.; Alessi, D.; Zierath, J. R.	2000	Did not perform fiber type analysis
Intact initiation of autophagy and mitochondrial fission by acute exercise in skeletal muscle of patients with TypeÂ 2 diabetes	Kruse, R.; Pedersen, A. J.; Kristensen, J. M.; Petersson, S. J.; Wojtaszewski, J. F.; HÃ¸jlund, K.	2017	Does not include males and females
Effects of insulin and exercise training on FGF21, its receptors and target genes in obesity and type 2 diabetes	Kruse, R.; Vienberg, S. G.; Vind, B. F.; Andersen, B.; HÃ¸jlund, K.	2017	Did not perform fiber type analysis
Markers of autophagy are adapted to hyperglycaemia in skeletal muscle in type 2 diabetes	Kruse, R.; Vind, B. F.; Petersson, S. J.; Kristensen, J. M.; HÃ¸jlund, K.	2015	Did not perform fiber type analysis
Autosomal recessive rippling muscle disease with homozygous CAV3 mutations	Kubisch, C.; Ketelsen, U. P.; Goebel, I.; Omran, H.	2005	Not peer reviewed (ex: thesis,

			conference poster)
Homozygous mutation in TXNRD1 is associated with genetic generalized epilepsy	Kudin, A. P.; Baron, G.; Zsurka, G.; Hampel, K. G.; Elger, C. E.; Grote, A.; Weber, Y.; Lerche, H.; Thiele, H.; NÃ¼rnberg, P.; Schulz, H.; Ruppert, A. K.; Sander, T.; Cheng, Q.; ArnÃ©r, E. S.; Schomburg, L.; Seeher, S.; Fradejas-Villar, N.; Schweizer, U.; Kunz, W. S.	2017	Only in children (0-17 years)
Diagnostic usefulness of intramuscular nerve bundles	Kudo, M.; Griggs, R. C.	1982	No healthy subjects or controls
Roux-en-Y gastric bypass surgery restores insulin-mediated glucose partitioning and mitochondrial dynamics in primary myotubes from severely obese humans	Kugler, B. A.; Gundersen, A. E.; Li, J.; Deng, W.; Eugene, N.; Gona, P. N.; Houmard, J. A.; Zou, K.	2020	Does not include males and females
Metformin regulates metabolic and nonmetabolic pathways in skeletal muscle and subcutaneous adipose tissues of older adults	Kulkarni, A. S.; Brutsaert, E. F.; Anghel, V.; Zhang, K.; Bloomgarden, N.; Pollak, M.; Mar, J. C.; Hawkins, M.; Crandall, J. P.; Barzilai, N.	2018	Did not perform fiber type analysis
Immunohistochemical study of clathrin in distal myopathy with rimmed vacuoles	Kumamoto, T.; Abe, T.; Nagao, S.; Ueyama, H.; Tsuda, T.	1998	Did not perform fiber type analysis
Proteasome expression in the skeletal muscles of patients with muscular dystrophy	Kumamoto, T.; Fujimoto, S.; Ito, T.; Horinouchi, H.; Ueyama, H.; Tsuda, T.	2000	No healthy subjects or controls
Increased lysosome-related proteins in the skeletal muscles of distal myopathy with rimmed vacuoles	Kumamoto, T.; Ito, T.; Horinouchi, H.; Ueyama, H.; Toyoshima, I.; Tsuda, T.	2000	Did not perform fiber type analysis
Calpain and cathepsins in the skeletal muscle of inflammatory myopathies	Kumamoto, T.; Ueyama, H.; Sugihara, R.; Kominami, E.; Goll, D. E.; Tsuda, T.	1997	No healthy subjects or controls
Expression of lysosome-related proteins and genes in the skeletal muscles of inclusion body myositis	Kumamoto, T.; Ueyama, H.; Tsumura, H.; Toyoshima, I.; Tsuda, T.	2004	Did not perform fiber type analysis
Adipose tissue macrophage populations and inflammation are associated with systemic inflammation and insulin resistance in obesity	Kunz, H. E.; Hart, C. R.; Gries, K. J.; Parvizi, M.; Laurenti, M.; Dalla Man, C.; Moore, N.; Zhang, X.; Ryan, Z.; Polley, E. C.; Jensen, M. D.; Vella, A.; Lanza, I. R.	2021	Did not perform fiber type analysis
Detection of mitochondrial defects by laser fluorimetry	Kunz, W. S.; Winkler, K.; Kuznetsov, A. V.; Lins, H.; Kirches, E.; Wallesch, C. W.	1997	Does not include males and females
Expression of tumor necrosis factor-alpha in regenerating muscle fibers in inflammatory and non-inflammatory myopathies	Kuru, S.; Inukai, A.; Kato, T.; Liang, Y.; Kimura, S.; Sobue, G.	2003	Did not perform fiber type analysis
Cryopreservation of mitochondria and mitochondrial function in cardiac and skeletal muscle fibers	Kuznetsov, A. V.; Kunz, W. S.; Saks, V.; Usson, Y.; Mazat, J. P.; Letellier, T.; Gellerich, F. N.; Margreiter, R.	2003	Did not perform fiber type analysis
Application of inhibitor titrations for the detection of oxidative phosphorylation defects in saponin-skinned muscle fibers of patients with mitochondrial diseases	Kuznetsov, A. V.; Winkler, K.; Kirches, E.; Lins, H.; Feistner, H.; Kunz, W. S.	1997	Does not report sex of subjects

The effect of simvastatin treatment on natural antioxidants in low-density lipoproteins and high-energy phosphates and ubiquinone in skeletal muscle	Laaksonen, R.; Jokelainen, K.; Laakso, J.; Sahi, T.; Harkonen, M.; Tikkanen, M. J.; Himberg, J. J.	1996	Does not include males and females
Thyroid hormone receptors are down-regulated in skeletal muscle of patients with non-thyroidal illness syndrome secondary to non-septic shock	Lado-Abeal, J.; Romero, A.; Castro-Piedras, I.; Rodriguez-Perez, A.; Alvarez-Escudero, J.	2010	No healthy subjects or controls
Deep morphological analysis of muscle biopsies from type III glycogenesis (GSDIII), debranching enzyme deficiency, revealed stereotyped vacuolar myopathy and autophagy impairment	LaforÃªt, P.; Inoue, M.; Goillot, E.; Lefeuvre, C.; Cagin, U.; Streichenberger, N.; Leonard-Louis, S.; Brochier, G.; Madelaine, A.; Labasse, C.; Hedberg-Oldfors, C.; Krag, T.; Jauze, L.; Fabregue, J.; Labrune, P.; Milisenda, J.; Nadaj-Pakleza, A.; Sacconi, S.; Mingozzi, F.; Ronzitti, G.; Petit, F.; Schoser, B.; Oldfors, A.; Vissing, J.; Romero, N. B.; Nishino, I.; Malfatti, E.	2019	Does not include male and female data
Role of accelerated aging in limb muscle wasting of patients with COPD	Lakhdar, R.; McGuinness, D.; Drost, E. M.; Shiels, P. G.; Bastos, R.; MacNee, W.; Rabinovich, R. A.	2018	Does not include male and female data
Skeletal muscle pathology in chronic heart block	Lambert, C. D.; Fairfax, A. J.	1977	Autopsies
Influence of creatine monohydrate ingestion on muscle metabolites and intense exercise capacity in individuals with multiple sclerosis	Lambert, C. P.; Archer, R. L.; Carrithers, J. A.; Fink, W. J.; Evans, W. J.; Trappe, T. A.	2003	Did not perform fiber type analysis
Endogenous and maximal sarcoplasmic reticulum calcium content and calsequestrin expression in type I and type II human skeletal muscle fibres	Lamboley, C. R.; Murphy, R. M.; McKenna, M. J.; Lamb, G. D.	2013	Does not include male and female data
Sarcoplasmic reticulum Ca2+ uptake and leak properties, and SERCA isoform expression, in type I and type II fibres of human skeletal muscle	Lamboley, C. R.; Murphy, R. M.; McKenna, M. J.; Lamb, G. D.	2014	Does not include male and female data
Contractile properties and sarcoplasmic reticulum calcium content in typeÂ I and typeÂ II skeletal muscle fibres in active aged humans	Lamboley, C. R.; Wyckelsma, V. L.; Dutka, T. L.; McKenna, M. J.; Murphy, R. M.; Lamb, G. D.	2015	Does not include male and female data
Regulation of STARS and its downstream targets suggest a novel pathway involved in human skeletal muscle hypertrophy and atrophy	Lamon, S.; Wallace, M. A.; LÃ©ger, B.; Russell, A. P.	2009	Does not include males and females
Structure of skeletal muscle in heart transplant recipients	Lampert, E.; Mettauer, B.; Hoppeler, H.; Charloux, A.; Charpentier, A.; Lonsdorfer, J.	1996	Did not perform fiber type analysis
Skeletal muscle response to short endurance training in heart transplant recipients	Lampert, E.; Mettauer, B.; Hoppeler, H.; Charloux, A.; Charpentier, A.; Lonsdorfer, J.	1998	Did not perform fiber type analysis
Muscle coenzyme Q10 level in statin-related myopathy	Lamperti, C.; Naini, A. B.; Lucchini, V.; Prelle, A.; Bresolin, N.; Moggio, M.; Sciacco, M.; Kaufmann, P.; DiMauro, S.	2005	No healthy subjects or controls
Sarcoplasmic reticulum Ca2+-ATPase and acylphosphatase activities in muscle biopsies from patients with Duchenne muscular dystrophy	Landi, N.; Nassi, P.; Liguri, G.; Bobbi, S.; Sbrilli, C.; Marconi, G.	1986	No healthy subjects or controls
Skeletal muscle sodium and potassium changes after successful surgery in acromegaly: relation	Landin, K.; Petruson, B.; Jakobsson, K. E.; Bengtsson, B. A.	1993	Does not include males and females

to body composition, blood glucose, plasma insulin and blood pressure			
Effects of sleeping with reduced carbohydrate availability on acute training responses	Lane, S. C.; Camera, D. M.; Lassiter, D. G.; Areta, J. L.; Bird, S. R.; Yeo, W. K.; Jeacocke, N. A.; Krook, A.; Zierath, J. R.; Burke, L. M.; Hawley, J. A.	2015	Does not include males and females
Inflammation-induced acute phase response in skeletal muscle and critical illness myopathy	Langhans, C.; Weber-Carstens, S.; Schmidt, F.; Hamati, J.; Kny, M.; Zhu, X.; Wollersheim, T.; Koch, S.; Krebs, M.; Schulz, H.; Lodka, D.; Saar, K.; Labeit, S.; Spies, C.; Hubner, N.; Spranger, J.; Spuler, S.; Boschmann, M.; Dittmar, G.; Butler-Browne, G.; Mouly, V.; Fielitz, J.	2014	Did not perform fiber type analysis
Muscular contractile failure in septic patients: role of the inducible nitric oxide synthase pathway	Lanone, S.; Mebazaa, A.; Heymes, C.; Henin, D.; Poderoso, J. J.; Panis, Y.; Zedda, C.; Billiar, T.; Payen, D.; Aubier, M.; Boczkowski, J.	2000	No healthy subjects or controls
High-intensity sprint training inhibits mitochondrial respiration through aconitase inactivation	Larsen, F. J.; Schiffer, T. A.; Ã˜rtenblad, N.; Zinner, C.; Morales-Alamo, D.; Willis, S. J.; Calbet, J. A.; Holmberg, H. C.; Boushel, R.	2016	Does not include males and females
Are substrate use during exercise and mitochondrial respiratory capacity decreased in arm and leg muscle in type 2 diabetes?	Larsen, S.; Ara, I.; RabÃ¸l, R.; Andersen, J. L.; Boushel, R.; Dela, F.; Helge, J. W.	2009	Does not include males and females
Influence of exercise amount and intensity on long-term weight loss maintenance and skeletal muscle mitochondrial ROS production in humans	Larsen, S.; Dandanell, S.; Kristensen, K. B.; JÃ¸rgensen, S. D.; Dela, F.; Helge, J. W.	2019	Does not include male and female data
Increased mitochondrial substrate sensitivity in skeletal muscle of patients with type 2 diabetes	Larsen, S.; Stride, N.; Hey-Mogensen, M.; Hansen, C. N.; Andersen, J. L.; Madsbad, S.; Worm, D.; Helge, J. W.; Dela, F.	2011	Does not include males and females
Skeletal muscle morphology in patients with restless legs syndrome	Larsson, B. W.; Kadi, F.; Ulfberg, J.; Aulin, K. P.	2007	Does not include male and female data
The effect of long-term arterial occlusion on energy metabolism of the human quadriceps muscle	Larsson, J.; Hultman, E.	1979	Did not perform fiber type analysis
Effects of long-term physical training and detraining on enzyme histochemical and functional skeletal muscle characteristic in man	Larsson, L.; Ansved, T.	1985	Does not include males and females
Age-related changes in contractile properties and expression of myosin isoforms in single skeletal muscle cells	Larsson, L.; Li, X.; Yu, F.; Degens, H.	1997	Does not include males and females
Adaptation by alternative RNA splicing of slow troponin T isoforms in type 1 but not type 2 Charcot-Marie-Tooth disease	Larsson, L.; Wang, X.; Yu, F.; HÃ¶Ã¶k, P.; Borg, K.; Chong, S. M.; Jin, J. P.	2008	Does not include male and female
Muscle changes in work-related chronic myalgia	Larsson, S. E.; Bengtsson, A.; BodegÃ¥rd, L.; Henriksson, K. G.; Larsson, J.	1988	Does not include males and females
Correlation Between Quantitative MRI and Muscle Histopathology in Muscle Biopsies from Healthy Controls and Patients with IBM, FSHD and OPMD	Lassche, S.; KÃ¼sters, B.; Heerschap, A.; Schyns, M. V. P.; Ottenheijm, C. A. C.; Voermans, N. C.; van Engelen, B. G. M.	2020	Does not include male and female data

Muscle fiber dysfunction contributes to weakness in inclusion body myositis	Lassche, S.; Rietveld, A.; Heerschap, A.; van Hees, H. W.; Hopman, M. T.; Voermans, N. C.; Saris, C. G.; van Engelen, B. G.; Ottenheijm, C. A.	2019	Does not include male and female data
FAK tyrosine phosphorylation is regulated by AMPK and controls metabolism in human skeletal muscle	Lassiter, D. G.; NylÃ©n, C.; SjÃ¶gren, R. J. O.; Chibalin, A. V.; Wallberg-Henriksson, H.; NÃ¤slund, E.; Krook, A.; Zierath, J. R.	2018	Does not include males and females
Fatty involution of the gluteus medius muscles: a late-onset girdle myopathy?	Lassoued, S.; Laroche, M.	2011	No healthy subjects or controls
Effects of breaking up prolonged sitting on skeletal muscle gene expression	Latouche, C.; Jowett, J. B.; Carey, A. L.; Bertovic, D. A.; Owen, N.; Dunstan, D. W.; Kingwell, B. A.	2013	Does not include males and females
MicroRNA-194 Modulates Glucose Metabolism and Its Skeletal Muscle Expression Is Reduced in Diabetes	Latouche, C.; Natoli, A.; Reddy-Luthmoodoo, M.; Heywood, S. E.; Armitage, J. A.; Kingwell, B. A.	2016	Does not include males and females
Adipogenic progenitors from obese human skeletal muscle give rise to functional white adipocytes that contribute to insulin resistance	Laurens, C.; Louche, K.; Sengenes, C.; CouÃ©, M.; Langin, D.; Moro, C.; Bourlier, V.	2016	Does not include males and females
Growth and differentiation factor 15 is secreted by skeletal muscle during exercise and promotes lipolysis in humans	Laurens, C.; Parmar, A.; Murphy, E.; Carper, D.; Lair, B.; Maes, P.; Vion, J.; Boulet, N.; Fontaine, C.; MarquÃ¨s, M.; Larrouy, D.; Harant, I.; Thalamas, C.; Montastier, E.; Caspar-Bauguil, S.; Bourlier, V.; Tavernier, G.; Grolleau, J. L.; BouloumiÃ©, A.; Langin, D.; Viguerie, N.; Bertile, F.; Blanc, S.; de Glisezinski, I.; O'Gorman, D.; Moro, C.	2020	Does not include males and females
Gross ultrastructural changes and necrotic fiber segments in elbow flexor muscles after maximal voluntary eccentric action in humans	Lauritzen, F.; Paulsen, G.; Raastad, T.; Bergersen, L. H.; Owe, S. G.	2009	Did not perform fiber type analysis
Acute regulation by insulin of phosphatidylinositol-3-kinase, Rad, Glut 4, and lipoprotein lipase mRNA levels in human muscle	Laville, M.; Auboeuf, D.; Khalfallah, Y.; Vega, N.; Riou, J. P.; Vidal, H.	1996	Did not perform fiber type analysis
Skeletal muscle transcriptional networks linked to type I myofiber grouping in Parkinson's disease	Lavin, K. M.; Sealfon, S. C.; McDonald, M. N.; Roberts, B. M.; Wilk, K.; Nair, V. D.; Ge, Y.; Lakshman Kumar, P.; Windham, S. T.; Bamman, M. M.	2020	Does not include male and female data
Increased shelterin mRNA expression in peripheral blood mononuclear cells and skeletal muscle following an ultra-long-distance running event	Laye, M. J.; Solomon, T. P.; Karstoft, K.; Pedersen, K. K.; Nielsen, S. D.; Pedersen, B. K.	2012	Did not perform fiber type analysis
Acute High-Intensity Exercise Impairs Skeletal Muscle Respiratory Capacity	Layec, G.; Blain, G. M.; Rossman, M. J.; Park, S. Y.; Hart, C. R.; Trinity, J. D.; Gifford, J. R.; Sidhu, S. K.; Weavil, J. C.; Hureau, T. J.; Amann, M.; Richardson, R. S.	2018	Does not include males and females
Immunochemical quantification of sarcoplasmic reticulum Ca(2+)-ATPase and calsequestrin in muscle biopsies from patients with myotonia congenita and paramyotonia congenita Eulenburg	Leberer, E.; Reichmann, H.	1994	Does not include male and female data

Effects of respiratory alkalosis on human skeletal muscle metabolism at the onset of submaximal exercise	LeBlanc, P. J.; Parolin, M. L.; Jones, N. L.; Heigenhauser, G. J.	2002	Does not include males and females
Effects of aerobic training on pyruvate dehydrogenase and pyruvate dehydrogenase kinase in human skeletal muscle	LeBlanc, P. J.; Peters, S. J.; Tunstall, R. J.; Cameron-Smith, D.; Heigenhauser, G. J.	2004	Does not include males and females
Sarcolemmal loss of active nNOS (Nos1) is an oxidative stress-dependent, early event driving disuse atrophy	Lechado, I. Terradas A.; Vitadello, M.; Traini, L.; Namuduri, A. V.; Gastaldello, S.; Gorza, L.	2018	Does not include males and females
Skeletal muscle secretome in Duchenne muscular dystrophy: a pivotal anti-inflammatory role of adiponectin	Lecompte, S.; Abou-Samra, M.; Boursereau, R.; Noel, L.; Brichard, S. M.	2017	Does not include male and female data
Transient increase in glucose 1,6-bisphosphate in human skeletal muscle during isometric contraction	Lee, A. D.; Katz, A.	1989	Does not include males and females
Identification of differentially expressed genes in skeletal muscle of non-diabetic insulin-resistant and insulin-sensitive Pima Indians by differential display PCR	Lee, Y. H.; Tokraks, S.; Pratley, R. E.; Bogardus, C.; Permana, P. A.	2003	Did not perform fiber type analysis
Influence of Smoking on the Expression of Genes and Proteins Related to Fat Infiltration, Inflammation, and Fibrosis in the Rotator Cuff Muscles of Patients With Chronic Rotator Cuff Tears: A Pilot Study	Lee, Y. S.; Kim, J. Y.; Ki, S. Y.; Chung, S. W.	2019	Does not include males and females
AMPK activation is fiber type specific in human skeletal muscle: effects of exercise and short-term exercise training	Lee-Young, R. S.; Canny, B. J.; Myers, D. E.; McConell, G. K.	2009	Does not include males and females
Effect of acute exercise on citrate synthase activity in untrained and trained human skeletal muscle	Leek, B. T.; Mudaliar, S. R.; Henry, R.; Mathieu-Costello, O.; Richardson, R. S.	2001	Does not include males and females
Molecular signalling towards mitochondrial breakdown is enhanced in skeletal muscle of patients with chronic obstructive pulmonary disease (COPD)	Leermakers, P. A.; Schols, Amwj; Kneppers, A. E. M.; Kelders, Mcjm; de Theije, C. C.; Lainscak, M.; Gosker, H. R.	2018	Did not perform fiber type analysis
Novel Essential Amino Acid Supplements Following Resistance Exercise Induce Aminoacidemia and Enhance Anabolic Signaling Irrespective of Age: A Proof-of-Concept Trial	Lees, M. J.; Wilson, O. J.; Webb, E. K.; Traylor, D. A.; Prior, T.; Elia, A.; Harlow, P. S.; Black, A. D.; Parker, P. J.; Harris, N.; Cooke, M.; Balchin, C.; Butterworth, M.; Phillips, S. M.; Ispoglou, T.	2020	Did not perform fiber type analysis
Expression of the splice variants of the p85alpha regulatory subunit of phosphoinositide 3-kinase in muscle and adipose tissue of healthy subjects and type 2 diabetic patients	Lefai, E.; Roques, M.; Vega, N.; Laville, M.; Vidal, H.	2001	Did not perform fiber type analysis
The effect of starvation and total parenteral nutrition on skeletal muscle amino acid content and membrane potential difference in normal man	Legaspi, A.; Roberts, J. P.; Albert, J. D.; Tracey, K. J.; Shires, G. T.; Lowry, S. F.	1988	Does not include males and females
Effect of starvation and total parenteral nutrition on electrolyte homeostasis in normal man	Legaspi, A.; Roberts, J. P.; Horowitz, G. D.; Albert, J. D.; Tracey, K. J.; Shires, G. T.; Lowry, S. F.	1988	Did not perform fiber type analysis

Akt signalling through GSK-3beta, mTOR and Foxo1 is involved in human skeletal muscle hypertrophy and atrophy	LÃ©ger, B.; Cartoni, R.; Praz, M.; Lamon, S.; DÃ©riaz, O.; Crettenand, A.; Gobelet, C.; Rohmer, P.; Konzelmann, M.; Luthi, F.; Russell, A. P.	2006	Does not include males and females
Human skeletal muscle atrophy in amyotrophic lateral sclerosis reveals a reduction in Akt and an increase in atrogin-1	LÃ©ger, B.; Vergani, L.; SorarÃ¹, G.; Hespel, P.; Derave, W.; Gobelet, C.; D'Ascenzio, C.; Angelini, C.; Russell, A. P.	2006	Does not include male and female data
Normal protein content but abnormally inhibited enzyme activity in muscle carnitine palmitoyltransferase II deficiency	Lehmann, D.; Zierz, S.	2014	Did not perform fiber type analysis
Human hexokinase II gene: exon-intron organization, mutation screening in NIDDM, and its relationship to muscle hexokinase activity	Lehto, M.; Huang, X.; Davis, E. M.; Le Beau, M. M.; Laurila, E.; Eriksson, K. F.; Bell, G. I.; Groop, L.	1995	Did not perform fiber type analysis
Dramatic reversal of derangements in muscle metabolism and left ventricular function after bariatric surgery	Leichman, J. G.; Wilson, E. B.; Scarborough, T.; Aguilar, D.; Miller, C. C., 3rd; Yu, S.; Algahim, M. F.; Reyes, M.; Moody, F. G.; Taegtmeyer, H.	2008	Did not perform fiber type analysis
Permeability and morphology of skeletal muscle capillaries in type 1 (insulin-dependent) diabetes mellitus	Leinonen, H.; Matikainen, E.; Juntunen, J.	1982	Does not include male and female data
MAPK signaling in the quadriceps of patients with chronic obstructive pulmonary disease	Lemire, B. B.; DebigarÃ©, R.; DubÃ©, A.; ThÃ©riault, M. E.; CÃ´tÃ©, C. H.; Maltais, F.	2012	Does not include males and females
Immunohistology of eye muscle in idiopathic orbital inflammatory disease (pseudotumor), Graves' ophthalmopathy and healthy controls	Lenderink, T.; Jager, M. J.; Bruijn, J. A.; de Keizer, R. J.	1993	No healthy subjects or controls
Dietary carnitine intake related to skeletal muscle and plasma carnitine concentrations in adult men and women	Lennon, D. L.; Shrago, E. R.; Madden, M.; Nagle, F. J.; Hanson, P.	1986	Did not perform fiber type analysis
Partial neuromuscular blockade and cardiovascular responses to static exercise in man	Leonard, B.; Mitchell, J. H.; Mizuno, M.; Rube, N.; Saltin, B.; Secher, N. H.	1985	Does not include males and females
Prolonged exercise to fatigue in humans impairs skeletal muscle Na+-K+-ATPase activity, sarcoplasmic reticulum Ca2+ release, and Ca2+ uptake	Leppik, J. A.; Aughey, R. J.; Medved, I.; Fairweather, I.; Carey, M. F.; McKenna, M. J.	2004	Did not perform fiber type analysis
JNK regulates muscle remodeling via myostatin/SMAD inhibition	Lessard, S. J.; MacDonald, T. L.; Pathak, P.; Han, M. S.; Coffey, V. G.; Edge, J.; Rivas, D. A.; Hirshman, M. F.; Davis, R. J.; Goodyear, L. J.	2018	Does not include males and females
Discrimination and consistency of five myosin ATPase stains in human normal and Duchenne dystrophic muscle	Lester, J. M.; Silber, D. I.; Bradley, W. G.; Cohen, M. H.; Hirsch, R. P.; Brenner, J. F.	1982	Case studies Elizabeth Bueckers
The Effect of ACL Reconstruction on Involved and Contralateral Limb Vastus Lateralis Morphology and Histology: A Pilot Study	Leszczynski, E. C.; Kuenze, C.; Brazier, B.; Visker, J.; Ferguson, D. P.	2021	Does not include males and females
Acclimatization of skeletal muscle mitochondria to high-altitude hypoxia during an ascent of Everest	Levett, D. Z.; Radford, E. J.; Menassa, D. A.; Graber, E. F.; Morash, A. J.; Hoppeler, H.; Clarke, K.; Martin, D. S.; Ferguson-Smith, A. C.; Montgomery, H. E.; Grocott, M. P.; Murray, A. J.	2012	Does not include male and female data

Tissue magnesium status in diabetes mellitus	Levin, G. E.; Mather, H. M.; Pilkington, T. R.	1981	Did not perform fiber type analysis
Increased inflammatory cytokine expression in the vastus lateralis of patients with knee osteoarthritis	Levinger, I.; Levinger, P.; Trenerry, M. K.; Feller, J. A.; Bartlett, J. R.; Bergman, N.; McKenna, M. J.; Cameron-Smith, D.	2011	Did not perform fiber type analysis
Downregulation of the serum response factor/miR-1 axis in the quadriceps of patients with COPD	Lewis, A.; Riddoch-Contreras, J.; Natanek, S. A.; Donaldson, A.; Man, W. D.; Moxham, J.; Hopkinson, N. S.; Polkey, M. I.; Kemp, P. R.	2012	Does not include male and female data
Metabolic and morphometric profile of muscle fibers in chronic hemodialysis patients	Lewis, M. I.; Fournier, M.; Wang, H.; Storer, T. W.; Casaburi, R.; Cohen, A. H.; Kopple, J. D.	2012	Does not include male and female data
Human aging, muscle mass, and fiber type composition	Lexell, J.	1995	Review (ex: systematic review/meta-analysis)
What is the effect of ageing on type 2 muscle fibres?	Lexell, J.; Downham, D.	1992	Does not include males and females
Mitochondrial DNA deletions in oculopharyngeal muscular dystrophy	Lezza, A. M.; Cormio, A.; Gerardi, P.; Silvestri, G.; Servidei, S.; Serlenga, L.; Cantatore, P.; Gadaleta, M. N.	1997	Case studies
Growth hormone exerts acute vascular effects independent of systemic or muscle insulin-like growth factor I	Li, G.; Del Rincon, J. P.; Jahn, L. A.; Wu, Y.; Gaylinn, B.; Thorner, M. O.; Liu, Z.	2008	Did not perform fiber type analysis
Muscle Injury Associated Elevated Oxidative Stress and Abnormal Myogenesis in Patients with Idiopathic Scoliosis	Li, J.; Tang, M.; Yang, G.; Wang, L.; Gao, Q.; Zhang, H.	2019	Does not include male and female data
Force-generating capacity of human myosin isoforms extracted from single muscle fibre segments	Li, M.; Larsson, L.	2010	Does not include males and females
There is no slowing of motility speed with increased body size in rat, human, horse and rhinoceros independent on temperature and skeletal muscle myosin isoform	Li, M.; Li, M.; Marx, J. O.; Larsson, L.	2011	Does not include males and females
Aberrant post-translational modifications compromise human myosin motor function in old age	Li, M.; Ogilvie, H.; Ochala, J.; Artemenko, K.; Iwamoto, H.; Yagi, N.; Bergquist, J.; Larsson, L.	2015	Does not include male and female data
The role of lymphotoxin in pathogenesis of polymyositis	Liang, Y.; Inukai, A.; Kuru, S.; Kato, T.; Doyu, M.; Sobue, G.	2000	Does not include males and females
Effect of postoperative nutrition on muscle high energy phosphates	Liaw, K. Y.; Askanazi, J.; Michelsen, C. B.; Furst, P. F.; Elwyn, D. H.; Kinney, J. M.	1982	Does not include males and females
In vivo measurement of human wrist extensor muscle sarcomere length changes	Lieber, R. L.; Loren, G. J.; FridÃ©n, J.	1994	Did not perform fiber type analysis
Both Traditional and Stair Climbing-based HIIT Cardiac Rehabilitation Induce Beneficial Muscle Adaptations	Lim, C.; Dunford, E. C.; Valentino, S. E.; Oikawa, S. Y.; McGlory, C.; Baker, S. K.; Macdonald, M. J.; Phillips, S. M.	2021	Does not include males and females

Myosin heavy chain isoform composition of human masseter muscle from subjects with different mandibular plane angles	Lim, D.; Beitzel, F.; Lynch, G.; Woods, M. G.	2006	No healthy subjects or controls
Large-scale mitochondrial DNA deletions in skeletal muscle of patients with end-stage renal disease	Lim, P. S.; Cheng, Y. M.; Wei, Y. H.	2000	Did not perform fiber type analysis
Mitochondrial DNA mutations and oxidative damage in skeletal muscle of patients with chronic uremia	Lim, P. S.; Ma, Y. S.; Cheng, Y. M.; Chai, H.; Lee, C. F.; Chen, T. L.; Wei, Y. H.	2002	No healthy subjects or controls
Disuse atrophy of human skeletal muscle. An enzyme histochemical study	Lindboe, C. F.; Platou, C. S.	1982	No healthy subjects or controls
Effect of immobilization of short duration on the muscle fibre size	Lindboe, C. F.; Platou, C. S.	1984	Does not include males and females
Low expression of IL-18 and IL-18 receptor in human skeletal muscle is associated with systemic and intramuscular lipid metabolism-Role of HIV lipodystrophy	Lindegaard, B.; Hvid, T.; Wolsk Mygind, H.; Mortensen, O. H.; GrÃ¸ndal, T.; Abildgaard, J.; Gerstoft, J.; Pedersen, B. K.; Baranowski, M.	2018	Does not include males and females
Mitochondrial dysfunction in calf muscles of patients with combined peripheral arterial disease and diabetes type 2	Lindegaard Pedersen, B.; BÃ¦kgaard, N.; Quistorff, B.	2017	Did not perform fiber type analysis
Negative regulation of HIF in skeletal muscle of elite endurance athletes: a tentative mechanism promoting oxidative metabolism	Lindholm, M. E.; Fischer, H.; Poellinger, L.; Johnson, R. S.; Gustafsson, T.; Sundberg, C. J.; Rundqvist, H.	2014	Does not include males and females
Ultrastructural analysis of skeletal muscle. Microvascular dimensions and basement membrane thickness in chronic heart failure	Lindsay, D. C.; Anand, I. S.; Bennett, J. G.; Pepper, J. R.; Yacoub, M. H.; Rothery, S. M.; Severs, N. J.; Poole-Wilson, P. A.	1994	No healthy subjects or controls
Histological abnormalities of muscle from limb, thorax and diaphragm in chronic heart failure	Lindsay, D. C.; Lovegrove, C. A.; Dunn, M. J.; Bennett, J. G.; Pepper, J. R.; Yacoub, M. H.; Poole-Wilson, P. A.	1996	No healthy subjects or controls
Antioxidative effects of exercise training in patients with chronic heart failure: increase in radical scavenger enzyme activity in skeletal muscle	Linke, A.; Adams, V.; Schulze, P. C.; Erbs, S.; Gielen, S.; Fiehn, E.; MÃ¶bius-Winkler, S.; Schubert, A.; Schuler, G.; Hambrecht, R.	2005	Does not include males and females
Cellular redox activity of coenzyme Q10: effect of CoQ10 supplementation on human skeletal muscle	Linnane, A. W.; Kopsidas, G.; Zhang, C.; Yarovaya, N.; Kovalenko, S.; Papakostopoulos, P.; Eastwood, H.; Graves, S.; Richardson, M.	2002	Does not include males and females
Effect of hyperoxia on aerobic and anaerobic performances and muscle metabolism during maximal cycling exercise	Linossier, M. T.; Dormois, D.; Arsac, L.; Denis, C.; Gay, J. P.; Geyssant, A.; Lacour, J. R.	2000	Does not include males and females
Enzyme adaptations of human skeletal muscle during bicycle short-sprint training and detraining	Linossier, M. T.; Dormois, D.; Perier, C.; Frey, J.; Geyssant, A.; Denis, C.	1997	Does not include males and females
Fatigue in type I fiber predominance: a muscle force and surface EMG study on the relative role of type I and type II muscle fibers	Linssen, W. H.; Stegeman, D. F.; Joosten, E. M.; Binkhorst, R. A.; Merks, M. J.; ter Laak, H. J.; Notermans, S. L.	1991	No healthy subjects or controls
Force and fatigue in human type I muscle fibres. A surface EMG study in patients with congenital myopathy and type I fibre predominance	Linssen, W. H.; Stegeman, D. F.; Joosten, E. M.; Merks, H. J.; ter Laak, H. J.; Binkhorst, R. A.; Notermans, S. L.	1991	Did not perform muscle biopsy

Human skeletal muscle myosin function at physiological and non-physiological temperatures	Lionikas, A.; Li, M.; Larsson, L.	2006	Does not include males and females
Cable parameters, sodium, potassium, chloride, and water content, and potassium efflux in isolated external intercostal muscle of normal volunteers and patients with myotonia congenita	Lipicky, R. J.; Bryant, S. H.; Salmon, J. H.	1971	Does not include males and females
Changes in lipoprotein-lipase activity and lipid stores in human skeletal muscle with prolonged heavy exercise	Lithell, H.; Orlander, J.; SchÃ©le, R.; SjÃ¶din, B.; Karlsson, J.	1979	Does not include males and females
The intracellular mechanism of insulin resistance in pancreatic cancer patients	Liu, J.; Knezetic, J. A.; StrÃ¶mmer, L.; Permert, J.; Larsson, J.; Adrian, T. E.	2000	Did not perform fiber type analysis
A Novel Type of Multiterminal Motor Endplate in Human Extraocular Muscles	Liu, J. X.; DomellÃ¶f, F. P.	2018	Autopsies
Myosin heavy chain composition of muscle spindles in human biceps brachii	Liu, J. X.; Eriksson, P. O.; Thornell, L. E.; Pedrosa-DomellÃ¶f, F.	2002	Autopsies
Complex Correlations Between Desmin Content, Myofiber Types, and Innervation Patterns in the Human Extraocular Muscles	Liu, J. X.; Pedrosa DomellÃ¶f, F.	2020	Does not include male and female data
Cytoskeletal Proteins in Myotendinous Junctions of Human Extraocular Muscles	Liu, J. X.; Pedrosa DomellÃ¶f, F.	2021	Does not include males and females
Muscle spindles in the deep muscles of the human neck: a morphological and immunocytochemical study	Liu, J. X.; Thornell, L. E.; Pedrosa-DomellÃ¶f, F.	2003	Does not include male and female data
Distribution of SERCA isoforms in human intrafusal fibers	Liu, J. X.; Thornell, L. E.; Pedrosa-DomellÃ¶f, F.	2003	Does not include male and female data
Immunolocalization of GQ1b and related gangliosides in human extraocular neuromuscular junctions and muscle spindles	Liu, J. X.; Willison, H. J.; Pedrosa-DomellÃ¶f, F.	2009	Did not perform fiber type analysis
Quantitative evaluation of skeletal muscle defects in second harmonic generation images	Liu, W.; Raben, N.; Ralston, E.	2013	Case studies
Response of growth and myogenic factors in human skeletal muscle to strength training	Liu, Y.; Heinichen, M.; Wirth, K.; Schmidtbleicher, D.; Steinacker, J. M.	2008	Does not include males and females
HSP70 expression in skeletal muscle of patients with peripheral arterial occlusive disease	Liu, Y.; Lehmann, M.; Baur, C.; Storck, M.; Sunder-Plassmann, L.; Steinacker, J. M.	2002	Did not perform fiber type analysis
Different effects on human skeletal myosin heavy chain isoform expression: strength vs. combination training	Liu, Y.; Schlumberger, A.; Wirth, K.; Schmidtbleicher, D.; Steinacker, J. M.	2003	Does not include males and females
Perimysial microarteriopathy in dermatomyositis with anti-nuclear matrix protein-2 antibodies	Liu, Y.; Zheng, Y.; Gang, Q.; Xie, Z.; Jin, Y.; Zhang, X.; Deng, X.; Hao, H.; Gao, F.; Zhang, Z.; Xiong, H.; Zhang, W.; Wang, Z.; Yuan, Y.	2020	Does not report sex of subjects
Unlike insulin, amino acids stimulate p70S6K but not GSK-3 or glycogen synthase in human skeletal muscle	Liu, Z.; Wu, Y.; Nicklas, E. W.; Jahn, L. A.; Price, W. J.; Barrett, E. J.	2004	Did not perform fiber type analysis
Muscle Fiber Type Changes in Lumbrical Muscles at Early Stages of Chronic Nerve Compression	Liu, Z. Y.; Chen, J. H.; Chen, Z. B.	2019	Animal study

Characterisation of insulin-like growth factor I receptor in skeletal muscles of normal and insulin resistant subjects	Livingston, N.; Pollare, T.; Lithell, H.; Arner, P.	1988	Does not include males and females
Wrist extensor muscle pathology in lateral epicondylitis	Ljung, B. O.; Lieber, R. L.; FridÃ©n, J.	1999	Does not report sex of subjects
RNA-sequencing reveals altered skeletal muscle contraction, E3 ligases, autophagy, apoptosis, and chaperone expression in patients with critical illness myopathy	Llano-Diez, M.; Fury, W.; Okamoto, H.; Bai, Y.; Gromada, J.; Larsson, L.	2019	Does not include males and females
Effect of hemodialysis on protein synthesis	LÃ¶fberg, E.; EssÃ©n, P.; McNurlan, M.; Wernerman, J.; Garlick, P.; Anderstam, B.; BergstrÃ¶m, J.; Alvestrand, A.	2000	Did not perform fiber type analysis
Increased concentrations of glycogen synthase protein in skeletal muscle of patients with NIDDM	LÃ¶fman, M.; Yki-JÃ¤rvinen, H.; Parkkonen, M.; LindstrÃ¶m, J.; Koranyi, L.; Schalin-JÃ¤ntti, C.; Groop, L.	1995	Did not perform fiber type analysis
Human satellite progenitor cells for use in myofascial repair: isolation and characterization	Logan, M. S.; Propst, J. T.; Nottingham, J. M.; Goodwin, R. L.; Pabon, D. F.; Terracio, L.; Yost, M. J.; Fann, S. A.	2010	Did not perform fiber type analysis
Intracellular free [Ca2+] in human skeletal muscle with myopathic carnitine deficiency	LÃ³pez, J. R.; BriceÃ±o, L. E.; Cordovez, G.; SÃ¡nchez, V.; Linares, N.	1989	Did not perform fiber type analysis
Neurogenic Muscle Biopsy Findings Are Common in Mitochondrial Myopathy	Lu, J. Q.; Mubaraki, A.; Yan, C.; Provias, J.; Tarnopolsky, M. A.	2019	Does not include male and female data
Polyglucosan bodies in intramuscular nerves: Association with muscle fiber denervation atrophy	Lu, J. Q.; Phan, C.; Zochodne, D.; Yan, C.	2016	Case studies
Myocellular basis for tapering in competitive distance runners	Luden, N.; Hayes, E.; Galpin, A.; Minchev, K.; Jemiolo, B.; Raue, U.; Trappe, T. A.; Harber, M. P.; Bowers, T.; Trappe, S.	2010	Does not include males and females
Differential metabolic effects of casein and soy protein meals on skeletal muscle in healthy volunteers	Luiking, Y. C.; Engelen, M. P.; Soeters, P. B.; Boirie, Y.; Deutz, N. E.	2011	Did not perform fiber type analysis
Erythropoietin receptor in human skeletal muscle and the effects of acute and long-term injections with recombinant human erythropoietin on the skeletal muscle	Lundby, C.; Hellsten, Y.; Jensen, M. B.; Munch, A. S.; Pilegaard, H.	2008	Does not include males and females
Acclimatization to 4100 m does not change capillary density or mRNA expression of potential angiogenesis regulatory factors in human skeletal muscle	Lundby, C.; Pilegaard, H.; Andersen, J. L.; van Hall, G.; Sander, M.; Calbet, J. A.	2004	Does not include male and female data
Oxidative DNA damage and repair in skeletal muscle of humans exposed to high-altitude hypoxia	Lundby, C.; Pilegaard, H.; van Hall, G.; Sander, M.; Calbet, J.; Loft, S.; MÃ¸ller, P.	2003	Did not perform fiber type analysis
Metabolic studies in human skeletal muscle tissue. Evaluation of an in vitro preparation intended for clinical metabolic studies	Lundholm, K.; Bylund, A. C.; Holm, J.; Smeds, S.; ScherstÃ©n, T.	1975	No healthy subjects or controls
Skeletal muscle glutathione after surgical trauma	Luo, J. L.; Hammarqvist, F.; Andersson, K.; Wernerman, J.	1996	No healthy subjects or controls

Surgical trauma decreases glutathione synthetic capacity in human skeletal muscle tissue	Luo, J. L.; Hammarqvist, F.; Andersson, K.; Wernerman, J.	1998	No healthy subjects or controls
Determination of intracellular glutathione in human skeletal muscle by reversed-phase high-performance liquid chromatography	Luo, J. L.; Hammarqvist, F.; Cotgreave, I. A.; Lind, C.; Andersson, K.; Wernerman, J.	1995	Does not include males and females
Investigation of splicing changes and post-translational processing of LMNA in sporadic inclusion body myositis	Luo, Y. B.; Mitrpant, C.; Johnsen, R.; Fabian, V.; Needham, M.; Fletcher, S.; Wilton, S. D.; Mastaglia, F. L.	2013	Did not perform fiber type analysis
Investigation of age-related changes in LMNA splicing and expression of progerin in human skeletal muscles	Luo, Y. B.; Mitrpant, C.; Johnsen, R. D.; Fabian, V. A.; Fletcher, S.; Mastaglia, F. L.; Wilton, S. D.	2013	Did not perform fiber type analysis
Role of the chemokine receptors CXCR3, CXCR4 and CCR7 in the intramuscular recruitment of plasmacytoid dendritic cells in dermatomyositis	Lv, J.; Li, L.; Li, W.; Ji, K.; Hou, Y.; Yan, C.; Dai, T.	2018	Did not perform fiber type analysis
Distribution of adenosine A1, A2A and A2B receptors in human skeletal muscle	Lynge, J.; Hellsten, Y.	2000	Does not include males and females
Study of the correlation between the noncanonical pathway of pyroptosis and idiopathic inflammatory myopathy	Ma, M.; Chai, K.; Deng, R.	2021	Animal study
Mitochondrial (mt)DNA changes in tissue may not be reflected by depletion of mtDNA in peripheral blood mononuclear cells in HIV-infected patients	Maagaard, A.; Holberg-Petersen, M.; Kollberg, G.; Oldfors, A.; Sandvik, L.; Bruun, J. N.	2006	Did not perform fiber type analysis
Acute change of titin at mid-sarcomere remains despite 8 wk of plyometric training	Macaluso, F.; Isaacs, A. W.; Di Felice, V.; Myburgh, K. H.	2014	Does not report sex of subjects
Habitual Myofibrillar Protein Synthesis Is Normal in Patients with Upper GI Cancer Cachexia	MacDonald, A. J.; Johns, N.; Stephens, N.; Greig, C.; Ross, J. A.; Small, A. C.; Husi, H.; Fearon, K. C.; Preston, T.	2015	Did not perform fiber type analysis
Muscle substrate utilization and lactate production	MacDougall, J. D.; Ray, S.; Sale, D. G.; McCartney, N.; Lee, P.; Garner, S.	1999	Does not include males and females
Muscle ultrastructural characteristics of elite powerlifters and bodybuilders	MacDougall, J. D.; Sale, D. G.; Elder, G. C.; Sutton, J. R.	1982	Does not report sex of subjects
Biochemical adaptation of human skeletal muscle to heavy resistance training and immobilization	MacDougall, J. D.; Ward, G. R.; Sale, D. G.; Sutton, J. R.	1977	Does not include males and females
Mechanisms of sarcopenia: motor unit remodelling and muscle fibre type shifts with ageing	Machek, S. B.	2018	Review (ex: systematic review/meta-analysis)
The anterior scalene muscle in thoracic outlet compression syndrome. Histochemical and morphometric studies	Machleder, H. I.; Moll, F.; Verity, M. A.	1986	Does not include males and females
Human skeletal muscle fibre types and force: velocity properties	MacIntosh, B. R.; Herzog, W.; Suter, E.; Wiley, J. P.; Sokolosky, J.	1993	Does not include male and female data
Sequenced response of extracellular matrix deadhesion and fibrotic regulators after muscle damage is involved in protection against future injury in human skeletal muscle	Mackey, A. L.; Brandstetter, S.; Schjerling, P.; Bojsen-Moller, J.; Qvortrup, K.; Pedersen, M. M.; Doessing, S.; Kjaer, M.; Magnusson, S. P.; Langberg, H.	2011	Does not include males and females

Skeletal muscle adiposity is associated with physical activity, exercise capacity and fibre shift in COPD	Maddocks, M.; Shrikrishna, D.; Vitoriano, S.; Natanek, S. A.; Tanner, R. J.; Hart, N.; Kemp, P. R.; Moxham, J.; Polkey, M. I.; Hopkinson, N. S.	2014	Does not include male and female data
Acute and regular exercise distinctly modulate serum, plasma and skeletal muscle BDNF in the elderly	MÃ¡derovÃ¡, D.; Krumpolec, P.; SlobodovÃ¡, L.; SchÃ¶n, M.; TirpÃ¡kovÃ¡, V.; KovaniÄovÃ¡, Z.; KlepochovÃ¡, R.; Vajda, M.; Å utovskÃ½, S.; CveÄka, J.; ValkoviÄ, L.; TurÄÃ¡ni, P.; KrÅ¡Å¡Ã¡k, M.; Sedliak, M.; Tsai, C. L.; UkropcovÃ¡, B.; Ukropec, J.	2019	Does not include male and female data
Calcium content and respiratory control index of isolated skeletal muscle mitochondria: effects of different isolation media	Madsen, K.; Ertbjerg, P.; Pedersen, P. K.	1996	Does not include males and females
Capillary density measurements in skeletal muscle using immunohistochemical staining with anti-collagen type IV antibodies	Madsen, K.; Holmskov, U.	1995	Does not include males and females
New insights into the protein aggregation pathology in myotilinopathy by combined proteomic and immunolocalization analyses	Maerkens, A.; OlivÃ©, M.; Schreiner, A.; Feldkirchner, S.; Schessl, J.; Uszkoreit, J.; Barkovits, K.; GÃ¼ttsches, A. K.; Theis, V.; Eisenacher, M.; Tegenthoff, M.; Goldfarb, L. G.; SchrÃ¶der, R.; Schoser, B.; van der Ven, P. F.; FÃ¼rst, D. O.; Vorgerd, M.; Marcus, K.; Kley, R. A.	2016	No healthy subjects or controls
Counterregulation in peripheral tissues: effect of systemic hypoglycemia on levels of substrates and catecholamines in human skeletal muscle and adipose tissue	Maggs, D. G.; Jacob, R.; Rife, F.; Caprio, S.; Tamborlane, W. V.; Sherwin, R. S.	1997	Did not perform fiber type analysis
Exercise capacity in heart failure patients: relative importance of heart and skeletal muscle	Magnusson, G.; Kaijser, L.; Rong, H.; Isberg, B.; SylvÃ©n, C.; Saltin, B.	1996	Does not include male and female data
Oncofetal expression of Wilms' tumor 1 (WT1) protein in human fetal, adult and neoplastic skeletal muscle tissues	Magro, G.; Salvatorelli, L.; Puzzo, L.; Musumeci, G.; Bisceglia, M.; Parenti, R.	2015	No healthy subjects or controls
Disuse-induced insulin resistance susceptibility coincides with a dysregulated skeletal muscle metabolic transcriptome	Mahmassani, Z. S.; Reidy, P. T.; McKenzie, A. I.; Stubben, C.; Howard, M. T.; Drummond, M. J.	2019	Did not perform fiber type analysis
Age-dependent skeletal muscle transcriptome response to bed rest-induced atrophy	Mahmassani, Z. S.; Reidy, P. T.; McKenzie, A. I.; Stubben, C.; Howard, M. T.; Drummond, M. J.	2019	Did not perform fiber type analysis
Hyperinsulinemia augments endothelin-1 protein expression and impairs vasodilation of human skeletal muscle arterioles	Mahmoud, A. M.; Szczurek, M. R.; Blackburn, B. K.; Mey, J. T.; Chen, Z.; Robinson, A. T.; Bian, J. T.; Unterman, T. G.; Minshall, R. D.; Brown, M. D.; Kirwan, J. P.; Phillips, S. A.; Haus, J. M.	2016	Did not perform fiber type analysis
Real-time RT-PCR analysis of housekeeping genes in human skeletal muscle following acute exercise	Mahoney, D. J.; Carey, K.; Fu, M. H.; Snow, R.; Cameron-Smith, D.; Parise, G.; Tarnopolsky, M. A.	2004	Did not perform fiber type analysis
An Integrated Understanding of the Molecular Mechanisms of How Adipose Tissue Metabolism Affects Long-term Body Weight Maintenance	Mai, K.; Li, L.; Wiegand, S.; Brachs, M.; Leupelt, V.; Ernert, A.; KÃ¼hnen, P.; HÃ¼bner, N.; Robinson, P.; Chen, W.; Krude, H.; Spranger, J.	2019	Did not perform fiber type analysis

Peripheral muscle dysfunction in idiopathic pulmonary arterial hypertension	Mainguy, V.; Maltais, F.; Saey, D.; Gagnon, P.; Martel, S.; Simon, M.; Provencher, S.	2010	Does not include male and female data
Insulin downregulates pyruvate dehydrogenase kinase (PDK) mRNA: potential mechanism contributing to increased lipid oxidation in insulin-resistant subjects	Majer, M.; Popov, K. M.; Harris, R. A.; Bogardus, C.; Prochazka, M.	1998	Did not perform fiber type analysis
Skeletal muscle capillaries in intermittent claudication	MÃ¤kitie, J.	1977	No healthy subjects or controls
Androgen-dependent impairment of myogenesis in spinal and bulbar muscular atrophy	Malena, A.; Pennuto, M.; Tezze, C.; Querin, G.; D'Ascenzo, C.; Silani, V.; Cenacchi, G.; Scaramozza, A.; Romito, S.; Morandi, L.; Pegoraro, E.; Russell, A. P.; SorarÃ¹, G.; Vergani, L.	2013	Does not include males and females
Skeletal muscle proteomic signature and metabolic impairment in pulmonary hypertension	Malenfant, S.; Potus, F.; Fournier, F.; Breuils-Bonnet, S.; Pflieger, A.; Bourassa, S.; Tremblay, Ãˆ; NehmÃ©, B.; Droit, A.; Bonnet, S.; Provencher, S.	2015	Does not include males and females
Effect of long-term muscle paralysis on human single fiber mechanics	Malisoux, L.; Jamart, C.; Delplace, K.; Nielens, H.; Francaux, M.; Theisen, D.	2007	Does not include males and females
Immunological changes in human skeletal muscle and blood after eccentric exercise and multiple biopsies	Malm, C.; Nyberg, P.; Engstrom, M.; Sjodin, B.; Lenkei, R.; Ekblom, B.; Lundberg, I.	2000	Does not include males and females
Leukocytes, cytokines, growth factors and hormones in human skeletal muscle and blood after uphill or downhill running	Malm, C.; SjÃ¶din, T. L.; SjÃ¶berg, B.; Lenkei, R.; RenstrÃ¶m, P.; Lundberg, I. E.; Ekblom, B.	2004	Does not include male and female data
Exercise-induced muscle damage and inflammation: re-evaluation by proteomics	Malm, C.; Yu, J. G.	2012	Does not include male and female data
Age-related changes in muscle fiber types in the human thyroarytenoid muscle: an immunohistochemical and stereological study using confocal laser scanning microscopy	Malmgren, L. T.; Fisher, P. J.; Bookman, L. M.; Uno, T.	1999	Autopsies
Numerical densities of myonuclei and satellite cells in muscle fiber types in the aging human thyroarytenoid muscle: an immunohistochemical and stereological study using confocal laser scanning microscopy	Malmgren, L. T.; Fisher, P. J.; Jones, C. E.; Bookman, L. M.; Uno, T.	2000	Autopsies
Muscle fiber and satellite cell apoptosis in the aging human thyroarytenoid muscle: a stereological study with confocal laser scanning microscopy	Malmgren, L. T.; Jones, C. E.; Bookman, L. M.	2001	Autopsies
Oxidative enzyme activities of the vastus lateralis muscle and the functional status in patients with COPD	Maltais, F.; LeBlanc, P.; Whittom, F.; Simard, C.; Marquis, K.; BÃ©langer, M.; Breton, M. J.; Jobin, J.	2000	Did not perform fiber type analysis
Oxidative capacity of the skeletal muscle and lactic acid kinetics during exercise in normal subjects and in patients with COPD	Maltais, F.; Simard, A. A.; Simard, C.; Jobin, J.; DesgagnÃ©s, P.; LeBlanc, P.	1996	Does not include males and females

Quadriceps myostatin expression in COPD	Man, W. D.; Natanek, S. A.; Riddoch-Contreras, J.; Lewis, A.; Marsh, G. S.; Kemp, P. R.; Polkey, M. I.	2010	Did not perform fiber type analysis
Cellular and molecular responses of human skeletal muscle exposed to hypoxic environment	Mancinelli, R.; Pietrangelo, T.; La Rovere, R.; Toniolo, L.; FanÃ², G.; Reggiani, C.; Fulle, S.	2011	Does not include males and females
Regular football training down-regulates miR-1303 muscle expression in veterans	Mancini, A.; Vitucci, D.; Orlandella, F. M.; Terracciano, A.; Mariniello, R. M.; Imperlini, E.; Grazioli, E.; OrrÃ¹, S.; Krustrup, P.; Salvatore, G.; Buono, P.	2021	Does not include males and females
Contribution of intrinsic skeletal muscle changes to 31P NMR skeletal muscle metabolic abnormalities in patients with chronic heart failure	Mancini, D. M.; Coyle, E.; Coggan, A.; Beltz, J.; Ferraro, N.; Montain, S.; Wilson, J. R.	1989	Does not include males and females
Differential regulation of intracellular glucose metabolism by glucose and insulin in human muscle	Mandarino, L. J.; Consoli, A.; Jain, A.; Kelley, D. E.	1993	Did not perform fiber type analysis
Interaction of carbohydrate and fat fuels in human skeletal muscle: impact of obesity and NIDDM	Mandarino, L. J.; Consoli, A.; Jain, A.; Kelley, D. E.	1996	Did not perform fiber type analysis
Fasting hyperglycemia normalizes oxidative and nonoxidative pathways of insulin-stimulated glucose metabolism in noninsulin-dependent diabetes mellitus	Mandarino, L. J.; Consoli, A.; Kelley, D. E.; Reilly, J. J.; Nurjhan, N.	1990	Did not perform fiber type analysis
Regulation of hexokinase II and glycogen synthase mRNA, protein, and activity in human muscle	Mandarino, L. J.; Printz, R. L.; Cusi, K. A.; Kinchington, P.; O'Doherty, R. M.; Osawa, H.; Sewell, C.; Consoli, A.; Granner, D. K.; DeFronzo, R. A.	1995	Did not perform fiber type analysis
Reduced force of diaphragm muscle fibers in patients with chronic thromboembolic pulmonary hypertension	Manders, E.; Bonta, P. I.; Kloek, J. J.; Symersky, P.; Bogaard, H. J.; Hooijman, P. E.; Jasper, J. R.; Malik, F. I.; Stienen, G. J.; Vonk-Noordegraaf, A.; de Man, F. S.; Ottenheijm, C. A.	2016	No healthy subjects or controls
Molecular Mechanisms of Diaphragm Myopathy in Humans With Severe Heart Failure	Mangner, N.; Garbade, J.; Heyne, E.; van den Berg, M.; Winzer, E. B.; Hommel, J.; Sandri, M.; Jozwiak-Nozdrzykowska, J.; Meyer, A. L.; Lehmann, S.; Schmitz, C.; Malfatti, E.; Schwarzer, M.; Ottenheijm, C. A. C.; Bowen, T. S.; Linke, A.; Adams, V.	2021	Does not include male and female data
Myogenic and proteolytic mRNA expression following blood flow restricted exercise	Manini, T. M.; Vincent, K. R.; Leeuwenburgh, C. L.; Lees, H. A.; Kavazis, A. N.; Borst, S. E.; Clark, B. C.	2011	Did not perform fiber type analysis
Determination of human skeletal muscle buffer value by homogenate technique: methods of measurement	Mannion, A. F.; Jakeman, P. M.; Willan, P. L.	1993	Did not perform fiber type analysis
Effects of isokinetic training of the knee extensors on high-intensity exercise performance and skeletal muscle buffering	Mannion, A. F.; Jakeman, P. M.; Willan, P. L.	1994	Did not perform fiber type analysis
Skeletal muscle buffer value, fibre type distribution and high intensity exercise performance in man	Mannion, A. F.; Jakeman, P. M.; Willan, P. L.	1995	Does not include male and female data

Insulin resistance and tissue glycogen content in the tumor-bearing state	Marat, D.; Noguchi, Y.; Yoshikawa, T.; Tsuburaya, A.; Ito, T.; Kondo, J.	1999	Did not perform fiber type analysis
Differential unfolded protein response in skeletal muscle from non-diabetic glucose tolerant or intolerant patients with obesity before and after bariatric surgery	Marciniak, C.; Duhem, C.; Boulinguiez, A.; Raverdy, V.; Baud, G.; Verkindt, H.; Caiazzo, R.; Staels, B.; Duez, H.; Pattou, F.; Lancel, S.	2020	Did not perform fiber type analysis
Oxidative Stress and Upregulation of Antioxidant Proteins, Including Adiponectin, in Extraocular Muscular Cells, Orbital Adipocytes, and Thyrocytes in Graves' Disease Associated with Orbitopathy	Marique, L.; Senou, M.; Craps, J.; Delaigle, A.; Van Regemorter, E.; WÃ©rion, A.; Van Regemorter, V.; Mourad, M.; Nyssen-Behets, C.; LengelÃ©, B.; Baldeschi, L.; Boschi, A.; Brichard, S.; Daumerie, C.; Many, M. C.	2015	No healthy subjects or controls
Extensive inflammatory cell infiltration in human skeletal muscle in response to an ultraendurance exercise bout in experienced athletes	Marklund, P.; Mattsson, C. M.; WÃ¥hlin-Larsson, B.; Ponsot, E.; Lindvall, B.; Lindvall, L.; Ekblom, B.; Kadi, F.	2013	Does not include males and females
Ibuprofen treatment blunts early translational signaling responses in human skeletal muscle following resistance exercise	Markworth, J. F.; Vella, L. D.; Figueiredo, V. C.; Cameron-Smith, D.	2014	Does not include males and females
Ultrastructural fiber typing of human abdominal muscles obliquus internus and obliquus externus	MÃ¡rquez, A.; Finol, H. J.	1990	No healthy subjects or controls
Superoxide Anion Production and Bioenergetic Profile in Young and Elderly Human Primary Myoblasts	Marrone, M.; La Rovere, R. M. L.; Guarnieri, S.; Di Filippo, E. S.; Monaco, G.; Pietrangelo, T.; Bultynck, G.; Fulle, S.; Mancinelli, R.	2018	Does not include males and females
Kearns-Sayre syndrome: biochemical studies of mitochondrial metabolism	Martens, M. E.; Peterson, P. L.; Lee, C. P.; Nigro, M. A.; Hart, Z.; Glasberg, M.; Hatfield, J. S.; Chang, C. H.	1988	Did not perform fiber type analysis
Variation among normal persons in short-term ventilatory capacity	Martin, B. J.; Thomas, C. M.	1982	Did not perform muscle biopsy
Analytical subcellular fractionation of normal human skeletal muscle by sucrose density gradient centrifugation	Martin, F. C.; Levi, A. J.; Slavin, G.; Peters, T. J.	1983	Did not perform fiber type analysis
Investigation of the organelle pathology of skeletal muscle in chronic alcoholism	Martin, F. C.; Slavin, G.; Levi, A. J.; Peters, T. J.	1984	Does not include male and female data
Nemaline myopathy. I. Histochemical study	Martin, L.; Reniers, J.	1968	Case studies
The influence of functional electrical stimulation on the properties of vastus lateralis fibres following total knee arthroplasty	Martin, T. P.; Gundersen, L. A.; Blevins, F. T.; Coutts, R. D.	1991	Does not include male and female data
Effects of fiber type and training on beta-adrenoceptor density in human skeletal muscle	Martin, W. H., 3rd; Coggan, A. R.; Spina, R. J.; Saffitz, J. E.	1989	Does not include males and females
Skeletal muscle beta-adrenoceptor distribution and responses to isoproterenol in hyperthyroidism	Martin, W. H., 3rd; Korte, E.; Tolley, T. K.; Saffitz, J. E.	1992	Does not include male and female data
Muscle glycogen utilization during shivering thermogenesis in humans	Martineau, L.; Jacobs, I.	1988	Does not include males and females

Muscle glycogen availability and temperature regulation in humans	Martineau, L.; Jacobs, I.	1989	Does not include males and females
Effects of muscle glycogen and plasma FFA availability on human metabolic responses in cold water	Martineau, L.; Jacobs, I.	1991	Does not include males and females
Uremia induces adipose tissue inflammation and muscle mitochondrial dysfunction	Martinez Cantarin, M. P.; Whitaker-Menezes, D.; Lin, Z.; Falkner, B.	2017	No healthy subjects or controls
Aging voice and the laryngeal muscle atrophy	Martins, R. H.; Benito Pessin, A. B.; Nassib, D. J.; Branco, A.; Rodrigues, S. A.; Matheus, S. M.	2015	Autopsies
Expression of muscle-type phosphorylase in innervated and aneural cultured muscle of patients with myophosphorylase deficiency	Martinuzzi, A.; Vergani, L.; Carrozzo, R.; Fanin, M.; Bartoloni, L.; Angelini, C.; Askanas, V.; Engel, W. K.	1993	Did not perform fiber type analysis
Skeletal muscle apoptotic signaling predicts thigh muscle volume and gait speed in community-dwelling older persons: an exploratory study	Marzetti, E.; Lees, H. A.; Manini, T. M.; Buford, T. W.; Aranda, J. M., Jr.; Calvani, R.; Capuani, G.; Marsiske, M.; Lott, D. J.; Vandenborne, K.; Bernabei, R.; Pahor, M.; Leeuwenburgh, C.; Wohlgemuth, S. E.	2012	Did not perform fiber type analysis
Altered mitochondrial quality control signaling in muscle of old gastric cancer patients with cachexia	Marzetti, E.; Lorenzi, M.; Landi, F.; Picca, A.; Rosa, F.; Tanganelli, F.; Galli, M.; Doglietto, G. B.; Pacelli, F.; Cesari, M.; Bernabei, R.; Calvani, R.; Bossola, M.	2017	Did not perform fiber type analysis
Insulin resistance in non-obese subjects is associated with activation of the JNK pathway and impaired insulin signaling in skeletal muscle	Masharani, U. B.; Maddux, B. A.; Li, X.; Sakkas, G. K.; Mulligan, K.; Schambelan, M.; Goldfine, I. D.; Youngren, J. F.	2011	Did not perform fiber type analysis
Heparanase activity in alveolar and embryonal rhabdomyosarcoma: implications for tumor invasion	Masola, V.; Maran, C.; Tassone, E.; Zin, A.; Rosolen, A.; Onisto, M.	2009	Did not perform fiber type analysis
Regulation of skeletal muscle morphology in type 2 diabetic subjects by troglitazone and metformin: relationship to glucose disposal	Mathieu-Costello, O.; Kong, A.; Ciaraldi, T. P.; Cui, L.; Ju, Y.; Chu, N.; Kim, D.; Mudaliar, S.; Henry, R. R.	2003	Does not include males and females
Protein expression of VEGF, IGF-1 and FGF in retroocular connective tissues and clinical correlation in Graves' ophthalmopathy	Matos, K.; Manso, P. G.; Marback, E.; Furlanetto, R.; Alberti, G. N.; NosÃ©, V.	2008	No healthy subjects or controls
Skeletal muscle fibre plasticity in response to selected environmental and physiological stimuli	Matsakas, A.; Patel, K.	2009	Review (ex: systematic review/meta-analysis)
Subcellular localization of fukutin and fukutin-related protein in muscle cells	Matsumoto, H.; Noguchi, S.; Sugie, K.; Ogawa, M.; Murayama, K.; Hayashi, Y. K.; Nishino, I.	2004	No healthy subjects or controls
Analysis of muscle proteins in acute quadriplegic myopathy	Matsumoto, N.; Nakamura, T.; Yasui, Y.; Torii, J.	2000	No healthy subjects or controls
Morphological adaptation of muscle collagen and receptor of advanced glycation end product (RAGE) in osteoarthritis patients with 12 weeks of resistance training: influence of anti-inflammatory or glucosamine treatment	Mattiello-Sverzut, A. C.; Petersen, S. G.; Kjaer, M.; Mackey, A. L.	2013	No healthy subjects or controls

Ultrastructural Localization of Histidine-rich Glycoprotein in Skeletal Muscle Fibers: Colocalization With AMP Deaminase	Mattii, L.; Bianchi, F.; Falleni, A.; Frascarelli, S.; Masini, M.; AlÃ¬, G.; Chiellini, G.; Sabbatini, A. R. M.	2020	Did not perform fiber type analysis
Skeletal muscle basement membrane in maturity-onset diabetes in the young	Mauer, M.; Steffes, M.; Johnson, E.; Rich, S.; Hoogwerf, B.; Chavers, B.; Barbosa, J.	1985	Did not perform fiber type analysis
IL-6 and Akt are involved in muscular pathogenesis in myasthenia gravis	Maurer, M.; Bougoin, S.; Feferman, T.; Frenkian, M.; Bismuth, J.; Mouly, V.; Clairac, G.; Tzartos, S.; Fadel, E.; Eymard, B.; Fuchs, S.; Souroujon, M. C.; Berrih-Aknin, S.	2015	Did not perform fiber type analysis
Muscle ultrasound in children: normal values and application to neuromuscular disorders	Maurits, N. M.; Beenakker, E. A.; van Schaik, D. E.; Fock, J. M.; van der Hoeven, J. H.	2004	Only in children (0-17 years)
Translational signaling responses preceding resistance training-mediated myofiber hypertrophy in young and old humans	Mayhew, D. L.; Kim, J. S.; Cross, J. M.; Ferrando, A. A.; Bamman, M. M.	2009	Does not include male and female data
Adiponectin decreases pyruvate dehydrogenase kinase 4 gene expression in obese- and diabetic-derived myotubes	McAinch, A. J.; Cameron-Smith, D.	2009	Did not perform fiber type analysis
Differential regulation of adiponectin receptor gene expression by adiponectin and leptin in myotubes derived from obese and diabetic individuals	McAinch, A. J.; Steinberg, G. R.; Mollica, J.; O'Brien, P. E.; Dixon, J. B.; Macaulay, S. L.; Kemp, B. E.; Cameron-Smith, D.	2006	Did not perform fiber type analysis
Effect of resistance exercise and carbohydrate ingestion on oxidative stress	McAnulty, S. R.; McAnulty, L. S.; Nieman, D. C.; Morrow, J. D.; Utter, A. C.; Dumke, C. L.	2005	Does not include males and females
Characteristics of titin in strength and power athletes	McBride, J. M.; Triplett-McBride, T.; Davie, A. J.; Abernethy, P. J.; Newton, R. U.	2003	Does not include males and females
Skeletal muscle nNOS mu protein content is increased by exercise training in humans	McConell, G. K.; Bradley, S. J.; Stephens, T. J.; Canny, B. J.; Kingwell, B. A.; Lee-Young, R. S.	2007	Does not include males and females
Circulating and tissue specific transcription of angiopoietin-like protein 4 in human Type 2 diabetes	McCulloch, L. J.; Bramwell, L. R.; Knight, B.; Kos, K.	2020	Did not perform fiber type analysis
Peripheral artery disease, calf skeletal muscle mitochondrial DNA copy number, and functional performance	McDermott, M. M.; Peterson, C. A.; Sufit, R.; Ferrucci, L.; Guralnik, J. M.; Kibbe, M. R.; Polonsky, T. S.; Tian, L.; Criqui, M. H.; Zhao, L.; Stein, J. H.; Li, L.; Leeuwenburgh, C.	2018	Did not perform fiber type analysis
Expression of class I and class II MHC antigens in neuromuscular diseases	McDouall, R. M.; Dunn, M. J.; Dubowitz, V.	1989	Does not include male and female data
RNA sequencing on muscle biopsy from a 5-week bed rest study reveals the effect of exercise and potential interactions with dorsal root ganglion neurons	McFarland, A. J.; Ray, P. R.; Bhai, S.; Levine, B. D.; Price, T. J.	2022	Did not perform fiber type analysis
Association of interleukin-6 signalling with the muscle stem cell response following muscle-lengthening contractions in humans	McKay, B. R.; De Lisio, M.; Johnston, A. P.; O'Reilly, C. E.; Phillips, S. M.; Tarnopolsky, M. A.; Parise, G.	2009	Does not include males and females
Co-expression of IGF-1 family members with myogenic regulatory factors following acute	McKay, B. R.; O'Reilly, C. E.; Phillips, S. M.; Tarnopolsky, M. A.; Parise, G.	2008	Does not include males and females

damaging muscle-lengthening contractions in humans			
Satellite cell number and cell cycle kinetics in response to acute myotrauma in humans: immunohistochemistry versus flow cytometry	McKay, B. R.; Toth, K. G.; Tarnopolsky, M. A.; Parise, G.	2010	Does not include males and females
Impaired muscle Ca2+ and K+ regulation contribute to poor exercise performance post-lung transplantation	McKenna, M. J.; Fraser, S. F.; Li, J. L.; Wang, X. N.; Carey, M. F.; Side, E. A.; Morton, J.; Snell, G. I.; Kjeldsen, K.; Williams, T. J.	2003	Does not include male and female data
N-acetylcysteine attenuates the decline in muscle Na+,K+-pump activity and delays fatigue during prolonged exercise in humans	McKenna, M. J.; Medved, I.; Goodman, C. A.; Brown, M. J.; Bjorksten, A. R.; Murphy, K. T.; Petersen, A. C.; Sostaric, S.; Gong, X.	2006	Does not include males and females
Creatine supplementation increases muscle total creatine but not maximal intermittent exercise performance	McKenna, M. J.; Morton, J.; Selig, S. E.; Snow, R. J.	1999	Did not perform fiber type analysis
Unchanged [3H]ouabain binding site content but reduced Na+-K+ pump Î±2-protein abundance in skeletal muscle in older adults	McKenna, M. J.; Perry, B. D.; Serpiello, F. R.; Caldow, M. K.; Levinger, P.; Cameron-Smith, D.; Levinger, I.	2012	Did not perform fiber type analysis
A pilot study examining the impact of exercise training on skeletal muscle genes related to the TLR signaling pathway in older adults following hip fracture recovery	McKenzie, A. I.; Briggs, R. A.; Barrows, K. M.; Nelson, D. S.; Kwon, O. S.; Hopkins, P. N.; Higgins, T. F.; Marcus, R. L.; Drummond, M. J.	2017	Did not perform fiber type analysis
Gene and MicroRNA Expression Responses to Exercise; Relationship with Insulin Sensitivity	McLean, C. S.; Mielke, C.; Cordova, J. M.; Langlais, P. R.; Bowen, B.; Miranda, D.; Coletta, D. K.; Mandarino, L. J.	2015	Did not perform fiber type analysis
Wnt and extraocular muscle sparing in amyotrophic lateral sclerosis	McLoon, L. K.; Harandi, V. M.; BrÃ¤nnstrÃ¶m, T.; Andersen, P. M.; Liu, J. X.	2014	Autopsies
Protein synthesis in cancer patients with inflammatory response: investigations with [15N]glycine	McMillan, D. C.; Preston, T.; Fearon, K. C.; Burns, H. J.; Slater, C.; Shenkin, A.	1994	No healthy subjects or controls
Plectin defects in epidermolysis bullosa simplex with muscular dystrophy	McMillan, J. R.; Akiyama, M.; Rouan, F.; Mellerio, J. E.; Lane, E. B.; Leigh, I. M.; Owaribe, K.; Wiche, G.; Fujii, N.; Uitto, J.; Eady, R. A.; Shimizu, H.	2007	Does not report sex of subjects
Response of protein synthesis in human skeletal muscle to insulin: an investigation with L-[2H5]phenylalanine	McNurlan, M. A.; EssÃ©n, P.; Thorell, A.; Calder, A. G.; Anderson, S. E.; Ljungqvist, O.; Sandgren, A.; Grant, I.; TjÃ¤der, I.; Ballmer, P. E.; et al.,	1994	Did not perform fiber type analysis
Age-dependent increases in oxidative damage to DNA, lipids, and proteins in human skeletal muscle	Mecocci, P.; FanÃ³, G.; Fulle, S.; MacGarvey, U.; Shinobu, L.; Polidori, M. C.; Cherubini, A.; Vecchiet, J.; Senin, U.; Beal, M. F.	1999	Did not perform fiber type analysis
Pharmacological targeting of the ephrin receptor kinase signalling by GLPG1790 in vitro and in vivo reverts oncophenotype, induces myogenic differentiation and radiosensitizes embryonal rhabdomyosarcoma cells	Megiorni, F.; Gravina, G. L.; Camero, S.; Ceccarelli, S.; Del Fattore, A.; Desiderio, V.; Papaccio, F.; McDowell, H. P.; Shukla, R.; Pizzuti, A.; Beirinckx, F.; Pujuguet, P.; Saniere, L.; der Aar, E. V.; Maggio, R.; De Felice, F.; Marchese, C.; Dominici, C.; Tombolini, V.; Festuccia, C.; Marampon, F.	2017	Only in children (0-17 years)
Single muscle fibre contractile properties differ between body-builders, power athletes and control subjects	Meijer, J. P.; Jaspers, R. T.; Rittweger, J.; Seynnes, O. R.; Kamandulis, S.; Brazaitis,	2015	Does not report sex of subjects

	M.; Skurvydas, A.; PiÅ¡ot, R.; Å imuniÄ, B.; Narici, M. V.; Degens, H.		
Neuromuscular abnormalities in the major mental illnesses. II. Muscle fiber and subterminal motor nerve abnormalities	Meltzer, H. Y.; Crayton, J. W.	1975	Does not include male and female data
Incidence of Z band streaming and myofibrillar disruptions in skeletal muscle from healthy young people	Meltzer, H. Y.; Kuncl, R. W.; Yang, V.	1976	Did not perform fiber type analysis
Quantitative histochemical evaluation of normal human skeletal muscle	Meltzer, H. Y.; Rastogi, S.; Ellison, J.	1976	No cross-sectional area data
Reduced muscle fiber force production and disrupted myofibril architecture in patients with chronic rotator cuff tears	Mendias, C. L.; Roche, S. M.; Harning, J. A.; Davis, M. E.; Lynch, E. B.; Sibilsky Enselman, E. R.; Jacobson, J. A.; Claflin, D. R.; Calve, S.; Bedi, A.	2015	Does not include male and female data
Inflammatory and satellite cells in the quadriceps of patients with COPD and response to resistance training	Menon, M. K.; Houchen, L.; Singh, S. J.; Morgan, M. D.; Bradding, P.; Steiner, M. C.	2012	Does not include male and female data
Calorie Restriction-induced Weight Loss and Exercise Have Differential Effects on Skeletal Muscle Mitochondria Despite Similar Effects on Insulin Sensitivity	Menshikova, E. V.; Ritov, V. B.; Dube, J. J.; Amati, F.; Stefanovic-Racic, M.; Toledo, F. G. S.; Coen, P. M.; Goodpaster, B. H.	2017	Did not perform fiber type analysis
Improved skeletal muscle oxidative enzyme activity and restoration of PGC-1 alpha and PPAR beta/delta gene expression upon rosiglitazone treatment in obese patients with type 2 diabetes mellitus	Mensink, M.; Hesselink, M. K.; Russell, A. P.; Schaart, G.; Sels, J. P.; Schrauwen, P.	2007	Does not include males and females
The beta enolase subunit displays three different patterns of microheterogeneity in human striated muscle	Merkulova, T.; Thornell, L. E.; Butler-Browne, G.; Oberlin, C.; Lucas, M.; LamandÃ©, N.; Lazar, M.; Keller, A.	1999	No healthy subjects or controls
Inflammatory and protein metabolism signaling responses in human skeletal muscle after burn injury	Merritt, E. K.; Cross, J. M.; Bamman, M. M.	2012	Did not perform fiber type analysis
Heightened muscle inflammation susceptibility may impair regenerative capacity in aging humans	Merritt, E. K.; Stec, M. J.; Thalacker-Mercer, A.; Windham, S. T.; Cross, J. M.; Shelley, D. P.; Craig Tuggle, S.; Kosek, D. J.; Kim, J. S.; Bamman, M. M.	2013	Does not include male and female data
Importance of pH regulation and lactate/H+ transport capacity for work production during supramaximal exercise in humans	Messonnier, L.; Kristensen, M.; Juel, C.; Denis, C.	2007	Does not include male and female data
Correlation of Fiber-Type Composition and Sprint Performance in Youth Soccer Players	Metaxas, T.; Mandroukas, A.; Michailidis, Y.; Koutlianos, N.; Christoulas, K.; Ekblom, B.	2019	Only in children (0-17 years)
Oxidative capacity of skeletal muscle in heart failure patients versus sedentary or active control subjects	Mettauer, B.; Zoll, J.; Sanchez, H.; Lampert, E.; Ribera, F.; Veksler, V.; Bigard, X.; Mateo, P.; Epailly, E.; Lonsdorfer, J.; Ventura-Clapier, R.	2001	Did not perform fiber type analysis
Effect of caffeine on intrinsic mechanical properties of normal and malignant hyperthermia-susceptible muscle	Metterlein, T.; Hartung, E.; Roewer, N.; Anetseder, M.	2015	Does not report sex of subjects
Neuromuscular Electrical Stimulation and Anabolic Signaling in Patients with Stroke	Mettler, J. A.; Bennett, S. M.; Doucet, B. M.; Magee, D. M.	2017	Does not report sex of subjects

Effect of weight loss on lactate transporter expression in skeletal muscle of obese subjects	Metz, L.; Mercier, J.; Tremblay, A.; AlmÃ©ras, N.; Joanisse, D. R.	2008	Does not include male and female data
Dicarbonyl stress and glyoxalase enzyme system regulation in human skeletal muscle	Mey, J. T.; Blackburn, B. K.; Miranda, E. R.; Chaves, A. B.; Briller, J.; Bonini, M. G.; Haus, J. M.	2018	Did not perform fiber type analysis
Skeletal muscle Nur77 and NOR1 insulin responsiveness is blunted in obesity and type 2 diabetes but improved after exercise training	Mey, J. T.; Solomon, T. P. J.; Kirwan, J. P.; Haus, J. M.	2019	Did not perform fiber type analysis
Insulin signalling in skeletal muscle of subjects with or without Type II-diabetes and first degree relatives of patients with the disease	Meyer, M. M.; Levin, K.; Grimmsmann, T.; Beck-Nielsen, H.; Klein, H. H.	2002	Did not perform fiber type analysis
Intact skeletal muscle mitochondrial enzyme activity but diminished exercise capacity in advanced heart failure patients on optimal medical and device therapy	Middlekauff, H. R.; Verity, M. A.; Horwich, T. B.; Fonarow, G. C.; Hamilton, M. A.; Shieh, P.	2013	Does not report sex of subjects
Abnormalities of calcium handling proteins in skeletal muscle mirror those of the heart in humans with heart failure: a shared mechanism?	Middlekauff, H. R.; Vigna, C.; Verity, M. A.; Fonarow, G. C.; Horwich, T. B.; Hamilton, M. A.; Shieh, P.; Tupling, A. R.	2012	No healthy subjects or controls
Adenine nucleotide translocase is acetylated in vivo in human muscle: Modeling predicts a decreased ADP affinity and altered control of oxidative phosphorylation	Mielke, C.; Lefort, N.; McLean, C. G.; Cordova, J. M.; Langlais, P. R.; Bordner, A. J.; Te, J. A.; Ozkan, S. B.; Willis, W. T.; Mandarino, L. J.	2014	Did not perform fiber type analysis
Calcium channel blockers are inadequate for malignant hyperthermia crisis	Migita, T.; Mukaida, K.; Yasuda, T.; Hamada, H.; Kawamoto, M.	2012	No healthy subjects or controls
Muscle coenzyme Q: a potential test for mitochondrial activity and redox status	Miles, L.; Miles, M. V.; Tang, P. H.; Horn, P. S.; Wong, B. L.; DeGrauw, T. J.; Morehart, P. J.; Bove, K. E.	2005	Only in children (0-17 years)
Muscle, adipose, and connective tissue variations in intrinsic musculature of the adult human tongue	Miller, J. L.; Watkin, K. L.; Chen, M. F.	2002	Autopsies
Mechanisms underlying skeletal muscle weakness in human heart failure: alterations in single fiber myosin protein content and function	Miller, M. S.; Vanburen, P.; Lewinter, M. M.; Lecker, S. H.; Selby, D. E.; Palmer, B. M.; Maughan, D. W.; Ades, P. A.; Toth, M. J.	2009	Does not include male and female data
A ketogenic diet combined with exercise alters mitochondrial function in human skeletal muscle while improving metabolic health	Miller, V. J.; LaFountain, R. A.; Barnhart, E.; Sapper, T. S.; Short, J.; Arnold, W. D.; Hyde, P. N.; Crabtree, C. D.; Kackley, M. L.; Kraemer, W. J.; Villamena, F. A.; Volek, J. S.	2020	Did not perform fiber type analysis
Effects of intermittent training on anaerobic performance and MCT transporters in athletes	Millet, G.; Bentley, D. J.; Roels, B.; Mc Naughton, L. R.; Mercier, J.; Cameron-Smith, D.	2014	Does not include males and females
mRNA expression of the long and short forms of uncoupling protein-3 in obese and lean humans	Millet, L.; Vidal, H.; Larrouy, D.; Andreelli, F.; Laville, M.; Langin, D.	1998	Did not perform fiber type analysis
Unreliable use of standard muscle hydration value in obesity	Mingrone, G.; Bertuzzi, A.; Capristo, E.; Greco, A. V.; Manco, M.; Pietrobelli, A.; Salinari, S.; Heymsfield, S. B.	2001	No healthy subjects or controls
Decreased uncoupling protein expression and intramyocytic triglyceride depletion in formerly obese subjects	Mingrone, G.; Rosa, G.; Greco, A. V.; Manco, M.; Vega, N.; Hesselink, M. K.; Castagneto, M.; Schrauwen, P.; Vidal, H.	2003	Did not perform fiber type analysis

Effects of general anaesthetic procedures on mitochondrial function of human skeletal muscle	MirÃ³, O.; Barrientos, A.; Alonso, J. R.; Casademont, J.; Jarreta, D.; Urbano-MÃ¡rquez, A.; Cardellach, F.	1999	Did not perform fiber type analysis
Histological and biochemical assessment of mitochondrial function in dermatomyositis	MirÃ³, O.; Casademont, J.; Grau, J. M.; Jarreta, D.; Urbano-MÃ¡rquez, A.; Cardellach, F.	1998	Does not include male and female data
Skeletal muscle mitochondrial function in polymyalgia rheumatica and in giant cell arteritis	MirÃ³, O.; Casademont, J.; Jarreta, D.; Grau, J. M.; Urbano-MÃ¡rquez, A.; Cardellach, F.	1999	Did not perform fiber type analysis
Absence of mitochondrial dysfunction in polymyalgia rheumatica. Evidence based on a simultaneous molecular and biochemical approach	MirÃ³, O.; Jarreta, D.; Casademont, J.; Barrientos, A.; RodrÃguez, B.; GÃ³mez, M.; Nunes, V.; Urbano-MÃ¡rquez, A.; Cardellach, F.	1999	Did not perform fiber type analysis
Skeletal muscle mitochondrial function is preserved in young patients with chronic renal failure	MirÃ³, O.; Marrades, R. M.; Roca, J.; Sala, E.; MasanÃ©s, F.; Campistol, J. M.; Torregrosa, J. V.; Casademont, J.; Wagner, P. D.; Cardellach, F.	2002	Does not report sex of subjects
Chronic corticosteroid administration causes mitochondrial dysfunction in skeletal muscle	Mitsui, T.; Azuma, H.; Nagasawa, M.; Iuchi, T.; Akaike, M.; Odomi, M.; Matsumoto, T.	2002	Does not include male and female data
In situ hybridization of myoglobin mRNA: results on the skeletal muscles of normal subjects and patients with neuromuscular diseases	Mitsui, T.; Kawai, H.; Naruo, T.; Nishino, H.; Saito, S.	1993	No healthy subjects or controls
Fiber-type specific caffeine sensitivities in normal human skinned muscle fibers	Mitsumoto, H.; DeBoer, G. E.; Bunge, G.; Andrish, J. T.; Tetzlaff, J. E.; Cruse, R. P.	1990	Does not include male and female data
Human respiratory muscles: fibre morphology and capillary supply	Mizuno, M.	1991	Does not include male and female data
Limb skeletal muscle adaptation in athletes after training at altitude	Mizuno, M.; Juel, C.; Bro-Rasmussen, T.; Mygind, E.; Schibye, B.; Rasmussen, B.; Saltin, B.	1990	Does not include males and females
Fibre types, capillary supply and enzyme activities in human intercostal muscles	Mizuno, M.; Secher, N. H.; Saltin, B.	1985	Does not include male and female data
Grouping patients for masseter muscle genotype-phenotype studies	Moawad, H. A.; Sinanan, A. C.; Lewis, M. P.; Hunt, N. P.	2012	Did not perform fiber type analysis
Activation of mTORC1 by leucine is potentiated by branched-chain amino acids and even more so by essential amino acids following resistance exercise	Moberg, M.; AprÃ³, W.; Ekblom, B.; van Hall, G.; Holmberg, H. C.; Blomstrand, E.	2016	Does not include males and females
Increased autophagy signaling but not proteasome activity in human skeletal muscle after prolonged low-intensity exercise with negative energy balance	Moberg, M.; Hendo, G.; Jakobsson, M.; Mattsson, C. M.; Ekblom-Bak, E.; Flockhart, M.; PontÃ©n, M.; SÃ¶derlund, K.; Ekblom, B.	2017	Did not perform fiber type analysis
Identification of neuromuscular junctions by correlative confocal and transmission electron microscopy	Modla, S.; Mendonca, J.; Czymmek, K. J.; Akins, R. E.	2010	Animal study
Angiogenesis-related factors in skeletal muscles of COPD patients: roles of angiopoietin-2	Mofarrahi, M.; Sigala, I.; Vassilokopoulos, T.; Harel, S.; Guo, Y.; Debigare, R.; Maltais, F.; Hussain, S. N.	2013	Does not include males and females

Maximal lipid oxidation in patients with type 2 diabetes is normal and shows an adequate increase in response to aerobic training	Mogensen, M.; Vind, B. F.; HÃ¸jlund, K.; Beck-Nielsen, H.; Sahlin, K.	2009	Does not include males and females
Lack of anionic phospholipid calcium binding sites in Duchenne muscular dystrophy	Moggio, M.; Fagiolari, G.; Prelle, A.; Gallanti, A.; Sciacco, M.; Scarlato, G.	1992	No healthy subjects or controls
Skeletal Muscle Mitochondrial Content, Oxidative Capacity, and Mfn2 Expression Are Reduced in Older Patients With Heart Failure and Preserved Ejection Fraction and Are Related to Exercise Intolerance	Molina, A. J.; Bharadwaj, M. S.; Van Horn, C.; Nicklas, B. J.; Lyles, M. F.; Eggebeen, J.; Haykowsky, M. J.; Brubaker, P. H.; Kitzman, D. W.	2016	Did not perform fiber type analysis
Molecular and cellular adaptations to exercise training in skeletal muscle from cancer patients treated with chemotherapy	MÃ¸ller, A. B.; LÃ¸nbro, S.; Farup, J.; Voss, T. S.; Rittig, N.; Wang, J.; HÃ¸jris, I.; Mikkelsen, U. R.; Jessen, N.	2019	Does not include males and females
Physical exercise increases autophagic signaling through ULK1 in human skeletal muscle	MÃ¸ller, A. B.; Vendelbo, M. H.; Christensen, B.; Clasen, B. F.; Bak, A. M.; JÃ¸rgensen, J. O.; MÃ¸ller, N.; Jessen, N.	2015	Does not include males and females
Resistance exercise, but not endurance exercise, induces IKKÎ² phosphorylation in human skeletal muscle of training-accustomed individuals	MÃ¸ller, A. B.; Vendelbo, M. H.; Rahbek, S. K.; Clasen, B. F.; Schjerling, P.; Vissing, K.; Jessen, N.	2013	Does not include males and females
Muscle biopsy studies in patients with moderate liver cirrhosis with special reference to energy-rich phosphagens and electrolytes	MÃ¶ller, P.; BergstrÃ¶m, J.; FÃ¼rst, P.; HellstrÃ¶m, K.	1984	Did not perform fiber type analysis
Myoglobin in human skeletal muscle	MÃ¶ller, P.; SylvÃ©n, C.	1981	Did not perform fiber type analysis
S-glutathionylation of troponin I (fast) increases contractile apparatus Ca2+ sensitivity in fast-twitch muscle fibres of rats and humans	Mollica, J. P.; Dutka, T. L.; Merry, T. L.; Lamboley, C. R.; McConell, G. K.; McKenna, M. J.; Murphy, R. M.; Lamb, G. D.	2012	Does not include male and female data
Chronic obstructive pulmonary disease does not impair responses to resistance training	MÃ¸lmen, K. S.; HammarstrÃ¶m, D.; Falch, G. S.; Grundtvig, M.; Koll, L.; Hanestadhaugen, M.; Khan, Y.; Ahmad, R.; Malerbakken, B.; RÃ¸dÃ¸len, T. J.; Lien, R.; RÃ¸nnestad, B. R.; Raastad, T.; Ellefsen, S.	2021	Does not include male and female data
Sexual dimorphism in human skeletal muscle mitochondrial bioenergetics in response to type 1 diabetes	Monaco, C. M. F.; Bellissimo, C. A.; Hughes, M. C.; Ramos, S. V.; Laham, R.; Perry, C. G. R.; Hawke, T. J.	2020	Did not perform fiber type analysis
Altered mitochondrial bioenergetics and ultrastructure in the skeletal muscle of young adults with type 1 diabetes	Monaco, C. M. F.; Hughes, M. C.; Ramos, S. V.; Varah, N. E.; Lamberz, C.; Rahman, F. A.; McGlory, C.; Tarnopolsky, M. A.; Krause, M. P.; Laham, R.; Hawke, T. J.; Perry, C. G. R.	2018	Did not perform fiber type analysis
Myosin heavy chain composition of the human lateral pterygoid and digastric muscles in young adults and elderly	Monemi, M.; Liu, J. X.; Thornell, L. E.; Eriksson, P. O.	2000	Autopsies
Diverse changes in fibre type composition of the human lateral pterygoid and digastric muscles during aging	Monemi, M.; Thornell, L.; Eriksson, P.	1999	Autopsies
Muscle Expression of Type I and Type II Interferons Is Increased in Juvenile Dermatomyositis and Related to Clinical and Histologic Features	Moneta, G. M.; Pires Marafon, D.; Marasco, E.; Rosina, S.; Verardo, M.; Fiorillo, C.; Minetti, C.; Bracci-Laudiero, L.; Ravelli, A.; De Benedetti, F.; Nicolai, R.	2019	No healthy subjects or controls

Quantitative and qualitative alterations of dystrophin are expressed in muscle cell cultures of Xp21 muscular dystrophy patients (Duchenne and Becker type)	Mongini, T.; Doriguzzi, C.; Palmucci, L.; ChiadÃ²-Piat, L.	1996	Does not include males and females
Activity patterns of human skeletal muscles: relation to muscle fiber type composition	Monster, A. W.; Chan, H.; O'Connor, D.	1978	Does not include males and females
Free amino acids in plasma and skeletal muscle of patients with liver cirrhosis	Montanari, A.; Simoni, I.; Vallisa, D.; TrifirÃ², A.; Colla, R.; Abbiati, R.; Borghi, L.; Novarini, A.	1988	Did not perform fiber type analysis
Refuting the myth of non-response to exercise training: 'non-responders' do respond to higher dose of training	Montero, D.; Lundby, C.	2017	Does not include males and females
Skeletal muscle inflammation and nitric oxide in patients with COPD	Montes de Oca, M.; Torres, S. H.; De Sanctis, J.; Mata, A.; HernÃ¡ndez, N.; TÃ¡lamo, C.	2005	Did not perform fiber type analysis
Caspase-12 ablation preserves muscle function in the mdx mouse	Moorwood, C.; Barton, E. R.	2014	Animal study
Skeletal muscle water and electrolytes following prolonged dehydrating exercise	Mora-RodrÃguez, R.; FernÃ¡ndez-ElÃas, V. E.; Hamouti, N.; Ortega, J. F.	2015	Does not include males and females
The mitochondrial tRNA(Leu(UUR)) mutation in mitochondrial encephalomyopathy, lactic acidosis, and strokelike episodes (MELAS): genetic, biochemical, and morphological correlations in skeletal muscle	Moraes, C. T.; Ricci, E.; Bonilla, E.; DiMauro, S.; Schon, E. A.	1992	Does not report sex of subjects
Skeletal muscle signaling, metabolism, and performance during sprint exercise in severe acute hypoxia after the ingestion of antioxidants	Morales-Alamo, D.; Guerra, B.; Ponce-GonzÃ¡lez, J. G.; Guadalupe-Grau, A.; Santana, A.; Martin-Rincon, M.; Gelabert-Rebato, M.; Cadefau, J. A.; Cusso, R.; Dorado, C.; Calbet, J. A. L.	2017	Does not include males and females
Increased oxidative stress and anaerobic energy release, but blunted Thr172-AMPKÎ± phosphorylation, in response to sprint exercise in severe acute hypoxia in humans	Morales-Alamo, D.; Ponce-GonzÃ¡lez, J. G.; Guadalupe-Grau, A.; RodrÃguez-GarcÃa, L.; Santana, A.; Cusso, M. R.; Guerrero, M.; Guerra, B.; Dorado, C.; Calbet, J. A.	2012	Does not include males and females
Critical role for free radicals on sprint exercise-induced CaMKII and AMPKÎ± phosphorylation in human skeletal muscle	Morales-Alamo, D.; Ponce-GonzÃ¡lez, J. G.; Guadalupe-Grau, A.; RodrÃguez-GarcÃa, L.; Santana, A.; Cusso, R.; Guerrero, M.; Dorado, C.; Guerra, B.; Calbet, J. A.	2013	Does not include males and females
Control of Muscle Fibro-Adipogenic Progenitors by Myogenic Lineage is Altered in Aging and Duchenne Muscular Dystrophy	Moratal, C.; Arrighi, N.; Dechesne, C. A.; Dani, C.	2019	Did not perform fiber type analysis
MRF4 negatively regulates adult skeletal muscle growth by repressing MEF2 activity	Moretti, I.; Ciciliot, S.; Dyar, K. A.; Abraham, R.; Murgia, M.; Agatea, L.; Akimoto, T.; Bicciato, S.; Forcato, M.; Pierre, P.; Uhlenhaut, N. H.; Rigby, P. W.; Carvajal, J. J.; Blaauw, B.; Calabria, E.; Schiaffino, S.	2016	Does not report sex of subjects
Use of papain in the preparation of adult mammalian skeletal muscle for tissue culture	Morgan, J.; Cohen, L.	1974	Does not report sex of subjects
Age-Associated ALU Element Instability in White Blood Cells Is Linked to Lower Survival in Elderly Adults: A Preliminary Cohort Study	Morgan, R. G.; Venturelli, M.; Gross, C.; Tarperi, C.; Schena, F.; Reggiani, C.; Naro,	2017	Did not perform fiber type analysis

	F.; Pedrinolla, A.; Monaco, L.; Richardson, R. S.; Donato, A. J.		
Phosphorylated TDP-43 aggregates in skeletal and cardiac muscle are a marker of myogenic degeneration in amyotrophic lateral sclerosis and various conditions	Mori, F.; Tada, M.; Kon, T.; Miki, Y.; Tanji, K.; Kurotaki, H.; Tomiyama, M.; Ishihara, T.; Onodera, O.; Kakita, A.; Wakabayashi, K.	2019	No healthy subjects or controls
Long term bed rest with and without vibration exercise countermeasures: effects on human muscle protein dysregulation	Moriggi, M.; Vasso, M.; Fania, C.; Capitanio, D.; Bonifacio, G.; Salanova, M.; Blottner, D.; Rittweger, J.; Felsenberg, D.; Cerretelli, P.; Gelfi, C.	2010	Does not include males and females
Electromechanical changes during electrically induced and maximal voluntary contractions: surface and intramuscular EMG responses during sustained maximal voluntary contraction	Moritani, T.; Muro, M.; Kijima, A.; Gaffney, F. A.; Parsons, D.	1985	Does not include males and females
Low skeletal muscle capillarization limits muscle adaptation to resistance exercise training in older adults	Moro, T.; Brightwell, C. R.; Phalen, D. E.; McKenna, C. F.; Lane, S. J.; Porter, C.; Volpi, E.; Rasmussen, B. B.; Fry, C. S.	2019	Does not include male and female data
Isolation and characterization of mesoangioblasts from facioscapulohumeral muscular dystrophy muscle biopsies	Morosetti, R.; Mirabella, M.; Gliubizzi, C.; Broccolini, A.; Sancricca, C.; Pescatori, M.; Gidaro, T.; Tasca, G.; Frusciante, R.; Tonali, P. A.; Cossu, G.; Ricci, E.	2007	Did not perform fiber type analysis
Maximum shortening velocity and myosin heavy-chain isoform expression in human masseter muscle fibers	Morris, T. J.; Brandon, C. A.; Horton, M. J.; Carlson, D. S.; Sciote, J. J.	2001	No healthy subjects or controls
Genetic and metabolic effects on skeletal muscle AMPK in young and older twins	Mortensen, B.; Poulsen, P.; Wegner, L.; Stender-Petersen, K. L.; Ribel-Madsen, R.; Friedrichsen, M.; Birk, J. B.; Vaag, A.; Wojtaszewski, J. F.	2009	Did not perform fiber type analysis
Postabsorptive respiratory quotient and insulin-stimulated glucose storage rate in nondiabetic pima indians are related To glycogen synthase fractional activity in cultured myoblasts	Mott, D. M.; Pratley, R. E.; Bogardus, C.	1998	Did not perform fiber type analysis
A study of the contractility, biochemistry and morphology of an isolated preparation of human skeletal muscle	Moulds, R. F.; Young, A.; Jones, D. A.; Edwards, R. H.	1977	No cross-sectional area data
Muscle-specific expression of hypoxia-inducible factor in human skeletal muscle	Mounier, R.; Pedersen, B. K.; Plomgaard, P.	2010	Does not include males and females
Hemodynamics and O2 uptake during maximal knee extensor exercise in untrained and trained human quadriceps muscle: effects of hyperoxia	Mourtzakis, M.; GonzÃ¡lez-Alonso, J.; Graham, T. E.; Saltin, B.	2004	Does not include males and females
Altered pharyngeal muscles in Parkinson disease	Mu, L.; Sobotka, S.; Chen, J.; Su, H.; Sanders, I.; Adler, C. H.; Shill, H. A.; Caviness, J. N.; Samanta, J. E.; Beach, T. G.	2012	Autopsies
Adult human mylohyoid muscle fibers express slow-tonic, alpha-cardiac, and developmental myosin heavy-chain isoforms	Mu, L.; Su, H.; Wang, J.; Han, Y.; Sanders, I.	2004	Autopsies
TWIST1 and TWIST2 regulate glycogen storage and inflammatory genes in skeletal muscle	Mudry, J. M.; Massart, J.; Szekeres, F. L.; Krook, A.	2015	Did not perform fiber type analysis

Single muscle fiber gene expression with run taper	Murach, K.; Raue, U.; Wilkerson, B.; Minchev, K.; Jemiolo, B.; Bagley, J.; Luden, N.; Trappe, S.	2014	Does not include males and females
Eosinophilic major basic protein and interleukin-5 in eosinophilic myositis	Murata, K.; Sugie, K.; Takamure, M.; Fujimoto, T.; Ueno, S.	2003	Did not perform fiber type analysis
Antioxidant treatment with N-acetylcysteine regulates mammalian skeletal muscle Na+-K+-ATPase alpha gene expression during repeated contractions	Murphy, K. T.; Medved, I.; Brown, M. J.; Cameron-Smith, D.; McKenna, M. J.	2008	Does not include males and females
Calpain-3 is autolyzed and hence activated in human skeletal muscle 24 h following a single bout of eccentric exercise	Murphy, R. M.; Goodman, C. A.; McKenna, M. J.; Bennie, J.; Leikis, M.; Lamb, G. D.	2007	Did not perform fiber type analysis
Activation of skeletal muscle calpain-3 by eccentric exercise in humans does not result in its translocation to the nucleus or cytosol	Murphy, R. M.; Vissing, K.; Latchman, H.; Lamboley, C.; McKenna, M. J.; Overgaard, K.; Lamb, G. D.	2011	Does not include males and females
A carnitine/acylcarnitine translocase assay applicable to biopsied muscle specimens without requiring mitochondrial isolation	Murthy, M. S.; Kamanna, V. S.; Pande, S. V.	1986	No healthy subjects or controls
Bradykinin stimulates prostaglandin E(2) release in human skeletal muscular fibroblasts	Muscella, A.; Cossa, L. G.; Vetrugno, C.; Marsigliante, S.	2020	Does not include males and females
Content of methylhistidines in normal and pathological human skeletal muscles	Mussini, E.; Cornelio, F.; Dworzak, F.; Cotellessa, L.; Morandi, L.; Colombo, L.; De Ponte, G.; Marcucci, F.	1983	No healthy subjects or controls
HMGB1 and RAGE in skeletal muscle inflammation: Implications for protein accumulation in inclusion body myositis	Muth, I. E.; ZschÃ¼ntzsch, J.; Kleinschnitz, K.; Wrede, A.; Gerhardt, E.; Balcarek, P.; Schreiber-Katz, O.; Zierz, S.; Dalakas, M. C.; Voll, R. E.; Schmidt, J.	2015	No healthy subjects or controls
Cellular and morphological changes with EAA supplementation before and after total knee arthroplasty	Muyskens, J. B.; Foote, D. M.; Bigot, N. J.; Strycker, L. A.; Smolkowski, K.; Kirkpatrick, T. K.; Lantz, B. A.; Shah, S. N.; Mohler, C. G.; Jewett, B. A.; Owen, E. C.; Dreyer, H. C.	2019	Does not include male and female data
Fibre characteristics and enzyme levels of arm and leg muscles in elite cross-country skiers	Mygind, E.	1995	Does not include males and females
Skeletal muscle involvement in friedreich ataxia and potential effects of recombinant human erythropoietin administration on muscle regeneration and neovascularization	Nachbauer, W.; Boesch, S.; Reindl, M.; Eigentler, A.; Hufler, K.; Poewe, W.; LÃ¶scher, W.; Wanschitz, J.	2012	Does not include male and female data
Calcium uptake in frozen muscle biopsy sections compared with other predictors of malignant hyperthermia susceptibility	Nagarajan, K.; Fishbein, W. N.; Muldoon, S. M.; Pezeshkpour, G.	1987	Does not report sex of subjects
Costimulatory markers in muscle of patients with idiopathic inflammatory myopathies and in cultured muscle cells	Nagaraju, K.; Raben, N.; Villalba, M. L.; Danning, C.; Loeffler, L. A.; Lee, E.; Tresser, N.; Abati, A.; Fetsch, P.; Plotz, P. H.	1999	Did not perform fiber type analysis
Altered mitochondrial regulation in quadriceps muscles of patients with COPD	Naimi, A. I.; Bourbeau, J.; Perrault, H.; Baril, J.; Wright-Paradis, C.; Rossi, A.; Taivassalo, T.; Sheel, A. W.; RabÃ¸l, R.; Dela, F.; Boushel, R.	2011	Does not include males and females

Asian Indians have enhanced skeletal muscle mitochondrial capacity to produce ATP in association with severe insulin resistance	Nair, K. S.; Bigelow, M. L.; Asmann, Y. W.; Chow, L. S.; Coenen-Schimke, J. M.; Klaus, K. A.; Guo, Z. K.; Sreekumar, R.; Irving, B. A.	2008	Did not perform fiber type analysis
Heat stress produces an early phase of protection against oxidative damage in human muscle	Naito, J.; Hartung, E.; Schramm, E.; Inselmann, G.	1999	Did not perform fiber type analysis
Cross reactive identification of types 1 and 2C fibers in human skeletal muscles with monoclonal anti-neurofilament (200 kd) antibody	Nakamura, T.; Kawahara, H.; Miyashita, H.; Watarai, K.; Takagi, M.; Tachibana, S.	1987	Does not include male and female data
Muscle fiber atrophy in the quadriceps in knee-joint disorders. Histochemical studies on 112 cases	Nakamura, T.; Kurosawa, H.; Kawahara, H.; Watarai, K.; Miyashita, H.	1986	No healthy subjects or controls
Messenger RNA degradation may be inhibited in sporadic inclusion body myositis	Nakano, S.; Shinde, A.; Ito, H.; Ito, H.; Kusaka, H.	2005	Does not report sex of subjects
Efficacy and mechanism of hypoxic postconditioning in salvage of ex vivo human rectus abdominis muscle from hypoxia/reoxygenation injury	Naparus, A.; Ashrafpour, H.; Hofer, S. O.; Zhong, T.; Huang, N.; Cahoon, N. J.; McAllister, S. E.; Neligan, P. C.; Roy, T.; Lipa, J. E.; Pang, C. Y.	2012	Does not include males and females
Combination of hypoxic preconditioning and postconditioning does not induce additive protection of ex vivo human skeletal muscle from hypoxia/reoxygenation injury	Naparus, A.; Ashrafpour, H.; Huang, N.; Hofer, S. O.; Zhong, T.; Forrest, C. R.; Pang, C. Y.	2012	Does not include males and females
Insulin-mediated phosphorylation of the proline-rich Akt substrate PRAS40 is impaired in insulin target tissues of high-fat diet-fed rats	Nascimento, E. B.; Fodor, M.; van der Zon, G. C.; Jazet, I. M.; Meinders, A. E.; Voshol, P. J.; Vlasblom, R.; Baan, B.; Eckel, J.; Maassen, J. A.; Diamant, M.; Ouwens, D. M.	2006	Did not perform fiber type analysis
Sestrin 3 regulation in type 2 diabetic patients and its influence on metabolism and differentiation in skeletal muscle	Nascimento, E. B.; Osler, M. E.; Zierath, J. R.	2013	Did not perform fiber type analysis
Enhanced glucose metabolism in cultured human skeletal muscle after Roux-en-Y gastric bypass surgery	Nascimento, E. B.; Riedl, I.; Jiang, L. Q.; Kulkarni, S. S.; NÃ¤slund, E.; Krook, A.	2015	No healthy subjects or controls
Pathways associated with reduced quadriceps oxidative fibres and endurance in COPD	Natanek, S. A.; Gosker, H. R.; Slot, I. G.; Marsh, G. S.; Hopkinson, N. S.; Moxham, J.; Kemp, P. R.; Schols, A. M.; Polkey, M. I.	2013	Does not include male and female data
Yin Yang 1 expression and localisation in quadriceps muscle in COPD	Natanek, S. A.; Riddoch-Contreras, J.; Marsh, G. S.; Hopkinson, N. S.; Man, W. D.; Moxham, J.; Polkey, M. I.; Kemp, P. R.	2011	Not in english
MuRF-1 and atrogin-1 protein expression and quadriceps fiber size and muscle mass in stable patients with COPD	Natanek, S. A.; Riddoch-Contreras, J.; Marsh, G. S.; Hopkinson, N. S.; Moxham, J.; Man, W. D.; Kemp, P. R.; Polkey, M. I.	2013	Does not include male and female data
Effects of 2 weeks lower limb immobilization and two separate rehabilitation regimens on gastrocnemius muscle protein turnover signaling and normalization genes	Nedergaard, A.; Jespersen, J. G.; Pingel, J.; Christensen, B.; Sroczynski, N.; Langberg, H.; Kjaer, M.; Schjerling, P.	2012	Does not include males and females
Glycosaminoglycan modifications in Duchenne muscular dystrophy: specific remodeling of chondroitin sulfate/dermatan sulfate	Negroni, E.; Henault, E.; Chevalier, F.; Gilbert-Sirieix, M.; Van Kuppevelt, T. H.; Papy-Garcia, D.; Uzan, G.; Albanese, P.	2014	Only in children (0-17 years)
Myoglobin levels in individual human skeletal muscle fibers of different types	Nemeth, P. M.; Lowry, O. H.	1984	Does not include males and females

Effect of training on muscle metabolism during treadmill sprinting	Nevill, M. E.; Boobis, L. H.; Brooks, S.; Williams, C.	1989	Did not perform fiber type analysis
Ultrastructural changes after concentric and eccentric contractions of human muscle	Newham, D. J.; McPhail, G.; Mills, K. R.; Edwards, R. H.	1983	Does not include males and females
Skeletal muscle phosphatidylcholine and phosphatidylethanolamine are related to insulin sensitivity and respond to acute exercise in humans	Newsom, S. A.; Brozinick, J. T.; Kiseljak-Vassiliades, K.; Strauss, A. N.; Bacon, S. D.; Kerege, A. A.; Bui, H. H.; Sanders, P.; Siddall, P.; Wei, T.; Thomas, M.; Kuo, M. S.; Nemkov, T.; D'Alessandro, A.; Hansen, K. C.; Perreault, L.; Bergman, B. C.	2016	Did not perform fiber type analysis
Mechanomyographic determination of post-activation potentiation in myopathies	Ng, A. R.; Arimura, K.; Akataki, K.; Mita, K.; Higuchi, I.; Osame, M.	2006	Did not perform fiber type analysis
Effect of chronic obstructive pulmonary disease on calcium pump ATPase expression in human diaphragm	Nguyen, T.; Rubinstein, N. A.; Vijayasarathy, C.; Rome, L. C.; Kaiser, L. R.; Shrager, J. B.; Levine, S.	2005	Does not include male and female data
Ethanol acutely decreases calcium transients in cultured human myotubes	NicolÃ¡s, J. M.; AntÃºnez, E.; Thomas, A. P.; FernÃ¡ndez-SolÃ , J.; TobÃas, E.; Estruch, R.; Urbano-MÃ¡rquez, A.	1998	Does not include males and females
On skeletal muscle pathology in trichinosis	Nicolesco, S.	1978	Case studies
Plasticity in mitochondrial cristae density allows metabolic capacity modulation in human skeletal muscle	Nielsen, J.; Gejl, K. D.; Hey-Mogensen, M.; Holmberg, H. C.; Suetta, C.; Krustrup, P.; Elemans, C. P. H.; Ã˜rtenblad, N.	2017	Does not include males and females
Human skeletal muscle glycogen utilization in exhaustive exercise: role of subcellular localization and fibre type	Nielsen, J.; Holmberg, H. C.; SchrÃ¸der, H. D.; Saltin, B.; Ortenblad, N.	2011	Does not include males and females
Increased subsarcolemmal lipids in type 2 diabetes: effect of training on localization of lipids, mitochondria, and glycogen in sedentary human skeletal muscle	Nielsen, J.; Mogensen, M.; Vind, B. F.; Sahlin, K.; HÃ¸jlund, K.; SchrÃ¸der, H. D.; Ortenblad, N.	2010	Does not include males and females
Blood flow restricted training leads to myocellular macrophage infiltration and upregulation of heat shock proteins, but no apparent muscle damage	Nielsen, J. L.; Aagaard, P.; Prokhorova, T. A.; Nygaard, T.; Bech, R. D.; Suetta, C.; Frandsen, U.	2017	Does not include males and females
Effects of lengthening contraction on calcium kinetics and skeletal muscle contractility in humans	Nielsen, J. S.; Madsen, K.; JÃ¸rgensen, L. V.; Sahlin, K.	2005	Does not include males and females
Influence of carbohydrate ingestion on immune changes after 2 h of intensive resistance training	Nieman, D. C.; Davis, J. M.; Brown, V. A.; Henson, D. A.; Dumke, C. L.; Utter, A. C.; Vinci, D. M.; Downs, M. F.; Smith, J. C.; Carson, J.; Brown, A.; McAnulty, S. R.; McAnulty, L. S.	2004	Does not include males and females
Carbohydrate ingestion influences skeletal muscle cytokine mRNA and plasma cytokine levels after a 3-h run	Nieman, D. C.; Davis, J. M.; Henson, D. A.; Walberg-Rankin, J.; Shute, M.; Dumke, C. L.; Utter, A. C.; Vinci, D. M.; Carson, J. A.; Brown, A.; Lee, W. J.; McAnulty, S. R.; McAnulty, L. S.	2003	Did not perform fiber type analysis
Bariatric surgery rapidly improves mitochondrial respiration in morbidly obese patients	Nijhawan, S.; Richards, W.; O'Hea, M. F.; Audia, J. P.; Alvarez, D. F.	2013	No healthy subjects or controls

Relation of plasma high-density lipoprotein cholesterol to lipoprotein-lipase activity in adipose tissue and skeletal muscle of man	NikkilÃ¤, E. A.; Taskinen, M. R.; Kekki, M.	1978	Did not perform fiber type analysis
Lipoprotein lipase activity in adipose tissue and skeletal muscle of runners: relation to serum lipoproteins	NikkilÃ¤, E. A.; Taskinen, M. R.; Rehunen, S.; HÃ¤rkÃ¶nen, M.	1978	Did not perform fiber type analysis
Muscle loss in elderly	NikoliÄ‡, M.; Vranid, T. S.; Arbanas, J.; CvijanoviÄ‡, O.; Bajek, S.	2010	Autopsies
Regulation of skeletal muscle PPAR delta mRNA expression in twins	Nilsson, E.; Poulsen, P.; SjÃ¶gren, M.; Ling, C.; RidderstrÃ¥le, M.; Groop, L.; Vaag, A.	2007	No cross-sectional area data
Redox state and mitochondrial respiratory chain function in skeletal muscle of LGMD2A patients	Nilsson, M. I.; Macneil, L. G.; Kitaoka, Y.; Alqarni, F.; Suri, R.; Akhtar, M.; Haikalis, M. E.; Dhaliwal, P.; Saeed, M.; Tarnopolsky, M. A.	2014	Does not include male and female data
Quantitative and morphometric studies of age-related changes in human ciliary muscle	Nishida, S.; Mizutani, S.	1992	No healthy subjects or controls
Myonuclear breakdown in sporadic inclusion body myositis is accompanied by DNA double strand breaks	Nishii, M.; Nakano, S.; Nakamura, S.; Wate, R.; Shinde, A.; Kaneko, S.; Kusaka, H.	2011	Does not report sex of subjects
ADAM-17 is expressed in the inflammatory myopathy and is involved with interstitial lung disease	Nishimi, A.; Isozaki, T.; Nishimi, S.; Ishii, S.; Tokunaga, T.; Furuya, H.; Wakabayashi, K.; Kasama, T.	2018	Did not perform fiber type analysis
Mitochondrial dysfunction and cerebral metabolic abnormalities in patients with mitochondrial encephalomyopathy subtypes: Evidence from proton MR spectroscopy and muscle biopsy	Niu, F. N.; Meng, H. L.; Chang, L. L.; Wu, H. Y.; Li, W. P.; Liu, R. Y.; Wang, H. T.; Zhang, B.; Xu, Y.	2017	Did not perform fiber type analysis
Cellular and Morphological Alterations in the Vastus Lateralis Muscle as the Result of ACL Injury and Reconstruction	Noehren, B.; Andersen, A.; Hardy, P.; Johnson, D. L.; Ireland, M. L.; Thompson, K. L.; Damon, B.	2016	Does not include male and female data
Novel demonstration of conformationally modified tau in sporadic inclusion-body myositis muscle fibers	Nogalska, A.; D'Agostino, C.; Engel, W. K.; Askanas, V.	2011	Does not report sex of subjects
Role of human skeletal muscle insulin receptor kinase in the in vivo insulin resistance of noninsulin-dependent diabetes mellitus and obesity	Nolan, J. J.; Freidenberg, G.; Henry, R.; Reichart, D.; Olefsky, J. M.	1994	Does not include males and females
Acute endurance exercise increases skeletal muscle uncoupling protein-3 gene expression in untrained but not trained humans	Noland, R. C.; Hickner, R. C.; Jimenez-Linan, M.; Vidal-Puig, A.; Zheng, D.; Dohm, G. L.; Cortright, R. N.	2003	Did not perform fiber type analysis
Glycogen and lactate metabolism during low-intensity exercise in man	Nordheim, K.; VÃ¸llestad, N. K.	1990	Does not include male and female data
Effect of high-intensity training on exercise-induced gene expression specific to ion homeostasis and metabolism	Nordsborg, N.; Bangsbo, J.; Pilegaard, H.	2003	Does not include males and females
The lactate receptor GPR81 is predominantly expressed in type II human skeletal muscle fibers: potential for lactate autocrine signaling	NordstrÃ¶m, F.; Liegnell, R.; AprÃ³, W.; Blackwood, S. J.; Katz, A.; Moberg, M.	2023	Does not include male and female data

Effect of amiodarone on 3H-ouabain binding sites in human skeletal muscle	NÃ¸rgaard, A.; Jensen, J. H.; Andreasen, F.	1990	Did not perform fiber type analysis
A method for the determination of the total number of 3H-ouabain binding sites in biopsies of human skeletal muscle	NÃ¸rgaard, A.; Kjeldsen, K.; Clausen, T.	1984	Did not perform fiber type analysis
Proteomic identification of secreted proteins from human skeletal muscle cells and expression in response to strength training	Norheim, F.; Raastad, T.; Thiede, B.; Rustan, A. C.; Drevon, C. A.; Haugen, F.	2011	Does not include males and females
Inosine monophosphate accumulation in energy-deficient human skeletal muscle with reference to substrate availability, fibre types and AMP deaminase activity	Norman, B.	1995	Does not include male and female data
Muscle AMP deaminase deficiency in 2% of a healthy population	Norman, B.; Glenmark, B.; Jansson, E.	1995	Did not perform fiber type analysis
Genetic and other determinants of AMP deaminase activity in healthy adult skeletal muscle	Norman, B.; Mahnke-Zizelman, D. K.; Vallis, A.; Sabina, R. L.	1998	Does not include male and female data
Regulation of skeletal muscle ATP catabolism by AMPD1 genotype during sprint exercise in asymptomatic subjects	Norman, B.; Sabina, R. L.; Jansson, E.	2001	Does not include male and female data
Increased IMP content in glycogen-depleted muscle fibres during submaximal exercise in man	Norman, B.; Sollevi, A.; Jansson, E.	1988	Does not include males and females
Alternative splice variant PGC-1Î±-b is strongly induced by exercise in human skeletal muscle	Norrbom, J.; SÃ¤llstedt, E. K.; Fischer, H.; Sundberg, C. J.; Rundqvist, H.; Gustafsson, T.	2011	Does not include males and females
Calpain-10 gene and protein expression in human skeletal muscle: effect of acute lipid-induced insulin resistance and type 2 diabetes	Norton, L.; Parr, T.; Chokkalingam, K.; Bardsley, R. G.; Ye, H.; Bell, G. I.; Pelsers, M. M.; van Loon, L. J.; Tsintzas, K.	2008	Does not include males and females
Molecular and immunohistochemical analysis of HER2/neu oncogene in synovial sarcoma	Nuciforo, P. G.; Pellegrini, C.; Fasani, R.; Maggioni, M.; Coggi, G.; Parafioriti, A.; Bosari, S.	2003	No healthy subjects or controls
Skeletal muscle morphology, protein synthesis, and gene expression in Ehlers-Danlos syndrome	Nygaard, R. H.; Jensen, J. K.; Voermans, N. C.; Heinemeier, K. M.; Schjerling, P.; Holm, L.; Agergaard, J.; Mackey, A. L.; Andersen, J. L.; Remvig, L.; Kjaer, M.	2017	Does not include male and female data
Short-term low-calorie diet remodels skeletal muscle lipid profile and metabolic gene expression in obese adults	NylÃ©n, C.; Lundell, L. S.; Massart, J.; Zierath, J. R.; NÃ¤slund, E.	2019	Did not perform fiber type analysis
Tyrosine kinase activity of skeletal muscle during insulin infusion in humans	Nyomba, B. L.; Ossowski, V. M.; Saad, M. F.; Bogardus, C.; Mott, D. M.	1992	Does not include males and females
Exercise training increases insulin-stimulated glucose disposal and GLUT4 (SLC2A4) protein content in patients with type 2 diabetes	O'Gorman, D. J.; Karlsson, H. K.; McQuaid, S.; Yousif, O.; Rahman, Y.; Gasparro, D.; Glund, S.; Chibalin, A. V.; Zierath, J. R.; Nolan, J. J.	2006	Did not perform fiber type analysis
Comparative effectiveness of accommodating and weight resistance training modes	O'Hagan, F. T.; Sale, D. G.; MacDougall, J. D.; Garner, S. H.	1995	Does not include male and female data

Obese subcutaneous adipose tissue impairs human myogenesis, particularly in old skeletal muscle, via resistin-mediated activation of NFÎºB	O'Leary, M. F.; Wallace, G. R.; Davis, E. T.; Murphy, D. P.; Nicholson, T.; Bennett, A. J.; Tsintzas, K.; Jones, S. W.	2018	Did not perform fiber type analysis
Chimpanzee super strength and human skeletal muscle evolution	O'Neill, M. C.; Umberger, B. R.; Holowka, N. B.; Larson, S. G.; Reiser, P. J.	2017	Does not report sex of subjects
Sonographically guided percutaneous muscle biopsy in diagnosis of neuromuscular disease: a useful alternative to open surgical biopsy	O'Sullivan, P. J.; Gorman, G. M.; Hardiman, O. M.; Farrell, M. J.; Logan, P. M.	2006	No healthy subjects or controls
Low-volume resistance exercise attenuates the decline in strength and muscle mass associated with immobilization	Oates, B. R.; Glover, E. I.; West, D. W.; Fry, J. L.; Tarnopolsky, M. A.; Phillips, S. M.	2010	Does not include male and female data
Effects of a preferential myosin loss on Ca2+ activation of force generation in single human skeletal muscle fibres	Ochala, J.; Larsson, L.	2008	Does not include males and females
Defective regulation of contractile function in muscle fibres carrying an E41K beta-tropomyosin mutation	Ochala, J.; Li, M.; Ohlsson, M.; Oldfors, A.; Larsson, L.	2008	Does not include male and female data
Changes in human skeletal muscles due to ageing. Histological and histochemical observations on autopsy material	Oertel, G.	1986	Autopsies
Ultra-high-resolution scanning electron microscopy of mitochondria and sarcoplasmic reticulum arrangement in human red, white, and intermediate muscle fibers	Ogata, T.; Yamasaki, Y.	1997	Does not include male and female data
An electron-microscopic study of the T-system in progressive muscular dystrophy (Duchenne) using lanthanum	Oguchi, K.; Tsukagoshi, H.	1980	Did not perform fiber type analysis
Histochemical responses of human soleus muscle fibers to long-term bedrest with or without countermeasures	Ohira, Y.; Yoshinaga, T.; Nonaka, I.; Ohara, M.; Yoshioka, T.; Yamashita-Goto, K.; Izumi, R.; Yasukawa, K.; Sekiguchi, C.; Shenkman, B. S.; Kozzlovskaya, I. B.	2000	Does not include males and females
Myonuclear domain and myosin phenotype in human soleus after bed rest with or without loading	Ohira, Y.; Yoshinaga, T.; Ohara, M.; Nonaka, I.; Yoshioka, T.; Yamashita-Goto, K.; Shenkman, B. S.; Kozlovskaya, I. B.; Roy, R. R.; Edgerton, V. R.	1999	Does not include males and females
Skeletal muscle biopsy: indications and results in 200 patients	Ojeda, V. J.; Jacobsen, P. F.; Spagnolo, D. V.; Cole, K.	1982	Did not perform fiber type analysis
Skeletal muscle contractile protein function is preserved in human heart failure	Okada, Y.; Toth, M. J.; Vanburen, P.	2008	Does not include males and females
Mitochondrial DNA deletions in inclusion body myositis	Oldfors, A.; Larsson, N. G.; Lindberg, C.; Holme, E.	1993	No healthy subjects or controls
Bcl-2 and Bax protein expression in human myopathies	OlivÃ©, M.; Ferrer, I.	1999	Did not perform fiber type analysis
Expression of the intermediate filament protein synemin in myofibrillar myopathies and other muscle diseases	OlivÃ©, M.; Goldfarb, L.; Dagvadorj, A.; Sambuughin, N.; Paulin, D.; Li, Z.; Goudeau, B.; Vicart, P.; Ferrer, I.	2003	Did not perform fiber type analysis

Overexpression of semicarbazide-sensitive amine oxidase in human myopathies	OlivÃ©, M.; Unzeta, M.; Moreno, D.; Ferrer, I.	2004	Does not include males and females
Muscle structural changes in mitochondrial myopathy relate to genotype	Olsen, D. B.; Langkilde, A. R.; Ã˜rngreen, M. C.; Rostrup, E.; Schwartz, M.; Vissing, J.	2003	Does not include male and female data
Effects of Repeated Eyelid Injections with Botulinum Toxin A on Innervation of Treated Muscles in Patients with Blepharospasm	Olson, R. M.; Mokhtarzadeh, A.; McLoon, L. K.; Harrison, A. R.	2019	Did not perform fiber type analysis
Fibre type-specific increase in passive muscle tension in spinal cord-injured subjects with spasticity	Olsson, M. C.; KrÃ¼ger, M.; Meyer, L. H.; Ahnlund, L.; Gransberg, L.; Linke, W. A.; Larsson, L.	2006	Does not include males and females
Elevated levels of transcripts encoding a human retroviral envelope protein (syncytin) in muscles from patients with motor neuron disease	Oluwole, S. O.; Yao, Y.; Conradi, S.; Kristensson, K.; Karlsson, H.	2007	Did not perform fiber type analysis
Neurogenic muscle involvement in myasthenia gravis. A clinical and histopathological study	Oosterhuis, H.; Bethlem, J.	1973	No cross-sectional area data
Effect of oral creatine supplementation on human muscle GLUT4 protein content after immobilization	Op 't Eijnde, B.; UrsÃ¸, B.; Richter, E. A.; Greenhaff, P. L.; Hespel, P.	2001	Did not perform fiber type analysis
Pre-cachexia in patients with stages I-III non-small cell lung cancer: systemic inflammation and functional impairment without activation of skeletal muscle ubiquitin proteasome system	Op den Kamp, C. M.; Langen, R. C.; Minnaard, R.; Kelders, M. C.; Snepvangers, F. J.; Hesselink, M. K.; Dingemans, A. C.; Schols, A. M.	2012	Did not perform fiber type analysis
Subcellular adaptation of the human diaphragm in chronic obstructive pulmonary disease	Orozco-Levi, M.; Gea, J.; Lloreta, J. L.; FÃ©lez, M.; Minguella, J.; Serrano, S.; Broquetas, J. M.	1999	Does not include males and females
Role of glycogen availability in sarcoplasmic reticulum Ca2+ kinetics in human skeletal muscle	Ã˜rtenblad, N.; Nielsen, J.; Saltin, B.; Holmberg, H. C.	2011	Does not include males and females
Orbicularis Oculi Morphological Alterations in Affected and Nonaffected Sides in Hemifacial Spasm	Osaki, M. H.; Osaki, T. H.; Osaki, T.; Gameiro, G. R.; Belfort, R., Jr.; Marie, S. K. N.	2020	Does not include males and females
Freeze-fracture electronmicroscopic analysis of plasma membranes of cultured muscle cells in Duchenne dystrophy	Osame, M.; Engel, A. G.; Rebouche, C. J.; Scott, R. E.	1981	No healthy subjects or controls
Changes in gene expression in responders and nonresponders to a low-intensity walking intervention	Osler, M. E.; Fritz, T.; Caidahl, K.; Krook, A.; Zierath, J. R.; Wallberg-Henriksson, H.	2015	Did not perform fiber type analysis
Regulation of MAP kinase pathway activity in vivo in human skeletal muscle	Osman, A. A.; Pendergrass, M.; Koval, J.; Maezono, K.; Cusi, K.; Pratipanawatr, T.; Mandarino, L. J.	2000	Did not perform fiber type analysis
Remarkable heterogeneity in myosin heavy-chain composition of the human young masseter compared with young biceps brachii	Osterlund, C.; LindstrÃ¶m, M.; Thornell, L. E.; Eriksson, P. O.	2012	Only in children (0-17 years)
Intrafusal myosin heavy chain expression of human masseter and biceps muscles at young age shows fundamental similarities but also marked differences	Ã–sterlund, C.; Liu, J. X.; Thornell, L. E.; Eriksson, P. O.	2013	Autopsies
Differences in fibre type composition between human masseter and biceps muscles in young	Osterlund, C.; Thornell, L. E.; Eriksson, P. O.	2011	Does not report sex of subjects

and adults reveal unique masseter fibre type growth pattern			
Evidence that interleukin-6 is produced in human skeletal muscle during prolonged running	Ostrowski, K.; Rohde, T.; Zacho, M.; Asp, S.; Pedersen, B. K.	1998	Does not include males and females
Myotoxicity of local anesthetics is equivalent in individuals with and without predisposition to malignant hyperthermia	Otsuki, S.; Yasuda, T.; Mukaida, K.; Noda, Y.; Kanzaki, R.; Miyoshi, H.; Kondo, T.; Hamada, H.; Kawamoto, M.	2018	No healthy subjects or controls
Activation of the ubiquitin-proteasome pathway in the diaphragm in chronic obstructive pulmonary disease	Ottenheijm, C. A.; Heunks, L. M.; Li, Y. P.; Jin, B.; Minnaard, R.; van Hees, H. W.; Dekhuijzen, P. N.	2006	No healthy subjects or controls
Changes in cross-bridge cycling underlie muscle weakness in patients with tropomyosin 3-based myopathy	Ottenheijm, C. A.; Lawlor, M. W.; Stienen, G. J.; Granzier, H.; Beggs, A. H.	2011	Does not include male and female data
Muscle fiber atrophy and regeneration coexist in collagen VI-deficient human muscle: role of calpain-3 and nuclear factor-Î°B signaling	Paco, S.; Ferrer, I.; Jou, C.; CusÃ, V.; Corbera, J.; Torner, F.; Gualandi, F.; Sabatelli, P.; Orozco, A.; GÃ³mez-Foix, A. M.; Colomer, J.; Nascimento, A.; Jimenez-Mallebrera, C.	2012	Only in children (0-17 years)
Adaptation to chronic eccentric exercise in humans: the influence of contraction velocity	Paddon-Jones, D.; Leveritt, M.; Lonergan, A.; Abernethy, P.	2001	Does not include male and female data
Hypercortisolemia alters muscle protein anabolism following ingestion of essential amino acids	Paddon-Jones, D.; Sheffield-Moore, M.; Creson, D. L.; Sanford, A. P.; Wolf, S. E.; Wolfe, R. R.; Ferrando, A. A.	2003	Did not perform fiber type analysis
The catabolic effects of prolonged inactivity and acute hypercortisolemia are offset by dietary supplementation	Paddon-Jones, D.; Sheffield-Moore, M.; Urban, R. J.; Aarsland, A.; Wolfe, R. R.; Ferrando, A. A.	2005	Does not include males and females
Responses of intercostal muscle biopsies from normal subjects and patients with myasthenia gravis	Pagala, M. K.; Nandakumar, N. V.; Venkatachari, S. A.; Ravindran, K.; Namba, T.; Grob, D.	1990	Does not include male and female data
Isoforms of creatine kinase: MM in the study of skeletal muscle damage	Page, S.; Jackson, M. J.; Coakley, J.; Edwards, R. H.	1989	Does not report sex of subjects
Altered muscle saccharide pattern in X-linked muscular dystrophy	PaljÃ¤rvi, L.; Karjalainen, K.; Kalimo, H.	1984	Did not perform fiber type analysis
Histochemical method for simultaneous fiber typing and demonstration of capillaries in skeletal muscle	PaljÃ¤rvi, L.; Naukkarinen, A.	1990	Review (ex: systematic review/meta-analysis)
Morphometric approaches to perifascicular atrophy in muscle biopsy: do they help to diagnose polymyositis?	PaljÃ¤rvi, L.; SnÃ¤ll, E. V.	1984	Did not perform fiber type analysis
An electron microscopic study of muscle capillary wall thickening in systemic lupus erythematosus	Pallis, M.; Hopkinson, N.; Lowe, J.; Powell, R.	1994	Does not include males and females
Distribution of cell adhesion molecules in skeletal muscle from patients with systemic lupus erythematosus	Pallis, M.; Robson, D. K.; Haskard, D. O.; Powell, R. J.	1993	Did not perform fiber type analysis
Acetylcholine receptors from human muscle as pharmacological targets for ALS therapy	Palma, E.; Reyes-Ruiz, J. M.; Lopergolo, D.; Roseti, C.; Bertollini, C.; Ruffolo, G.; Cifelli,	2016	No healthy subjects or controls

	P.; Onesti, E.; Limatola, C.; Miledi, R.; Inghilleri, M.		
Metabolic and performance responses to constant-load vs. variable-intensity exercise in trained cyclists	Palmer, G. S.; Borghouts, L. B.; Noakes, T. D.; Hawley, J. A.	1999	Does not include males and females
Vascular endothelial cell growth factor and fibroblast growth factor 2 expression in patients with critical limb ischemia	Palmer-Kazen, U.; Wariaro, D.; Luo, F.; Wahlberg, E.	2004	Did not perform fiber type analysis
Heat shock factor activation in human muscles following a demanding intermittent exercise protocol is attenuated with hyperthermia	Palomero, J.; Broome, C. S.; Rasmussen, P.; Mohr, M.; Nielsen, B.; Nybo, L.; McArdle, A.; Drust, B.	2008	Does not include males and females
Regulation of myosin heavy chain antisense long noncoding RNA in human vastus lateralis in response to exercise training	Pandorf, C. E.; Haddad, F.; Owerkowicz, T.; Carroll, L. P.; Baldwin, K. M.; Adams, G. R.	2020	Did not perform fiber type analysis
Latissimus dorsi fine needle muscle biopsy: a novel and efficient approach to study proximal muscles of upper limbs	Paoli, A.; Pacelli, Q. F.; Toniolo, L.; Miotti, D.; Reggiani, C.	2010	Does not include male and female data
Adhesion molecule expression in vivo on extraocular muscles (EOM) in thyroid-associated ophthalmopathy (TAO)	Pappa, A.; Calder, V.; Fells, P.; Lightman, S.	1997	No healthy subjects or controls
Structural protein alterations to resistance and endurance cycling exercise training	Parcell, A. C.; Woolstenhulme, M. T.; Sawyer, R. D.	2009	Does not include males and females
Satellite cells: promoting adaptation over a lifetime	Parise, G.	2014	Not peer reviewed (ex: thesis, conference poster)
Myosin heavy chain isoform expression in human extraocular muscles: longitudinal variation and patterns of expression in global and orbital layers	Park, K. A.; Lim, J.; Sohn, S.; Oh, S. Y.	2012	No healthy subjects or controls
Innervated myotendinous cylinders alterations in human extraocular muscles in patients with strabismus	Park, S. E.; Sa, H. S.; Oh, S. Y.	2009	No healthy subjects or controls
The age-dependent induction of apoptosis-inducing factor (AIF) in the human semitendinosus skeletal muscle	Park, S. Y.; Kim, H. Y.; Lee, J. H.; Yoon, K. H.; Chang, M. S.; Park, S. K.	2010	Does not include males and females
Differential expression of apoptosis-related factors induces the age-related apoptosis of the gracilis muscle in humans	Park, S. Y.; Lee, J. H.; Kim, H. Y.; Yoon, K. H.; Park, S. K.; Chang, M. S.	2014	Does not report sex of subjects
Age and sex differences in human skeletal muscle fibrosis markers and transforming growth factor-Î² signaling	Parker, L.; Caldow, M. K.; Watts, R.; Levinger, P.; Cameron-Smith, D.; Levinger, I.	2017	Did not perform fiber type analysis
Regulation of skeletal muscle glycogen phosphorylase and PDH during maximal intermittent exercise	Parolin, M. L.; Chesley, A.; Matsos, M. P.; Spriet, L. L.; Jones, N. L.; Heigenhauser, G. J.	1999	Does not include males and females
Regulation of glycogen phosphorylase and PDH during exercise in human skeletal muscle during hypoxia	Parolin, M. L.; Spriet, L. L.; Hultman, E.; Hollidge-Horvat, M. G.; Jones, N. L.; Heigenhauser, G. J.	2000	Does not include males and females

Effects of PDH activation by dichloroacetate in human skeletal muscle during exercise in hypoxia	Parolin, M. L.; Spriet, L. L.; Hultman, E.; Matsos, M. P.; Hollidge-Horvat, M. G.; Jones, N. L.; Heigenhauser, G. J.	2000	Does not include males and females
Increased satellite cell apoptosis in vastus lateralis muscle after anterior cruciate ligament reconstruction	Parstorfer, M.; Profit, F.; Weiberg, N.; Wehrstein, M.; BariÃ©, A.; Friedmann-Bette, B.	2021	Does not include males and females
Altered muscle energy metabolism in post-absorptive patients with chronic renal failure	Pastoris, O.; Aquilani, R.; Foppa, P.; Bovio, G.; Segagni, S.; Baiardi, P.; Catapano, M.; Maccario, M.; Salvadeo, A.; Dossena, M.	1997	Did not perform fiber type analysis
Changes in inorganic phosphate and force production in human skeletal muscle after cast immobilization	Pathare, N.; Walter, G. A.; Stevens, J. E.; Yang, Z.; Okerke, E.; Gibbs, J. D.; Esterhai, J. L.; Scarborough, M. T.; Gibbs, C. P.; Sweeney, H. L.; Vandenborne, K.	2005	Does not include male and female data
The effect of exercise on skeletal muscle fibre type distribution in obesity: From cellular levels to clinical application	Pattanakuhar, S.; Pongchaidecha, A.; Chattipakorn, N.; Chattipakorn, S. C.	2017	Review (ex: systematic review/meta-analysis)
Inflammatory markers CD11b, CD16, CD66b, CD68, myeloperoxidase and neutrophil elastase in eccentric exercised human skeletal muscles	Paulsen, G.; Egner, I.; Raastad, T.; Reinholt, F.; Owe, S.; Lauritzen, F.; Brorson, S. H.; Koskinen, S.	2013	Did not perform fiber type analysis
Subcellular movement and expression of HSP27, alphaB-crystallin, and HSP70 after two bouts of eccentric exercise in humans	Paulsen, G.; Lauritzen, F.; Bayer, M. L.; Kalhovde, J. M.; Ugelstad, I.; Owe, S. G.; HallÃ©n, J.; Bergersen, L. H.; Raastad, T.	2009	No cross-sectional area data
Ultrastructural fiber typing in normal and diseased human muscle	Payne, C. M.; Stern, L. Z.; Curless, R. G.; Hannapel, L. K.	1975	No healthy subjects or controls
Anterior Cruciate Ligament Tear Promotes Skeletal Muscle Myostatin Expression, Fibrogenic Cell Expansion, and a Decline in Muscle Quality	Peck, B. D.; Brightwell, C. R.; Johnson, D. L.; Ireland, M. L.; Noehren, B.; Fry, C. S.	2019	Did not perform fiber type analysis
The HO-1/CO system regulates mitochondrial-capillary density relationships in human skeletal muscle	Pecorella, S. R.; Potter, J. V.; Cherry, A. D.; Peacher, D. F.; Welty-Wolf, K. E.; Moon, R. E.; Piantadosi, C. A.; Suliman, H. B.	2015	Did not perform fiber type analysis
High rates of muscle glycogen resynthesis after exhaustive exercise when carbohydrate is coingested with caffeine	Pedersen, D. J.; Lessard, S. J.; Coffey, V. G.; Churchley, E. G.; Wootton, A. M.; Ng, T.; Watt, M. J.; Hawley, J. A.	2008	Does not report sex of subjects
Evidence against altered expression of GLUT1 or GLUT4 in skeletal muscle of patients with obesity or NIDDM	Pedersen, O.; Bak, J. F.; Andersen, P. H.; Lund, S.; Moller, D. E.; Flier, J. S.; Kahn, B. B.	1990	Did not perform fiber type analysis
Epinephrine stimulates human muscle lipoprotein lipase activity in vivo	Pedersen, S. B.; Bak, J. F.; Holck, P.; Schmitz, O.; Richelsen, B.	1999	Does not include males and females
Altered surface myoelectric signals in peripheral vascular disease: correlations with muscle fiber composition	Pedrinelli, R.; Marino, L.; Dell'Omo, G.; Siciliano, G.; Rossi, B.	1998	Does not include males and females
Lack of muscle toxicity with didanosine (ddI). Clinical and experimental studies	Pedrol, E.; MasanÃ©s, F.; FernÃ¡ndez-SolÃ¡, J.; Cofan, M.; Casademont, J.; Grau, J. M.; Urbano-MÃ¡rquez, A.	1996	Does not report sex of subjects
Effect of Plyometrics on the Energy Cost of Running and MHC and Titin Isoforms	Pellegrino, J.; Ruby, B. C.; Dumke, C. L.	2016	Does not include male and female data

Distribution and morphometry of skeletal muscle fibers in patients with chronic obstructive pulmonary disease and chronic hypoxemia	Pereira, M. C.; Isayama, R. N.; Seabra, J. C.; Campos, G. E.; Paschoal, I. A.	2004	Does not include male and female data
Comparison of the molecular, antigenic and ATPase determinants of fast myosin heavy chains in rat and human: a single-fibre study	Pereira Sant'Ana, J. A.; Ennion, S.; Sargeant, A. J.; Moorman, A. F.; Goldspink, G.	1997	Does not report sex of subjects
Expression levels of RyR1 and RyR3 control resting free Ca2+ in skeletal muscle	Perez, C. F.; LÃ³pez, J. R.; Allen, P. D.	2005	Did not perform fiber type analysis
Antioxidants Facilitate High-intensity Exercise IL-15 Expression in Skeletal Muscle	PÃ©rez-LÃ³pez, A.; Martin-Rincon, M.; Santana, A.; Perez-Suarez, I.; Dorado, C.; Calbet, J. A. L.; Morales-Alamo, D.	2019	Does not include males and females
Experimental Hyperglycemia Alters Circulating Concentrations and Renal Clearance of Oxidative and Advanced Glycation End Products in Healthy Obese Humans	Perkins, R. K.; Miranda, E. R.; Karstoft, K.; Beisswenger, P. J.; Solomon, T. P. J.; Haus, J. M.	2019	Did not perform fiber type analysis
The effects of osteoarthritis and age on skeletal muscle strength, Na+-K+-ATPase content, gene and isoform expression	Perry, B. D.; Levinger, P.; Serpiello, F. R.; Caldow, M. K.; Cameron-Smith, D.; Bartlett, J. R.; Feller, J. A.; Bergman, N. R.; Levinger, I.; McKenna, M. J.	2013	Did not perform fiber type analysis
Dissociation between short-term unloading and resistance training effects on skeletal muscle Na+,K+-ATPase, muscle function, and fatigue in humans	Perry, B. D.; Wyckelsma, V. L.; Murphy, R. M.; Steward, C. H.; Anderson, M.; Levinger, I.; Petersen, A. C.; McKenna, M. J.	2016	Does not include male and female data
Repeated transient mRNA bursts precede increases in transcriptional and mitochondrial proteins during training in human skeletal muscle	Perry, C. G.; Lally, J.; Holloway, G. P.; Heigenhauser, G. J.; Bonen, A.; Spriet, L. L.	2010	Does not include males and females
The effects of training in hyperoxia vs. normoxia on skeletal muscle enzyme activities and exercise performance	Perry, C. G.; Talanian, J. L.; Heigenhauser, G. J.; Spriet, L. L.	2007	Did not perform fiber type analysis
Skeletal muscle of gastric cancer patients expresses genes involved in muscle regeneration	Pessina, P.; Conti, V.; Pacelli, F.; Rosa, F.; Doglietto, G. B.; Brunelli, S.; Bossola, M.	2010	No healthy subjects or controls
Immunochemical demonstration of a novel beta-subunit isoform of X, K-ATPase in human skeletal muscle	Pestov, N. B.; Korneenko, T. V.; Zhao, H.; Adams, G.; Shakhparonov, M. I.; Modyanov, N. N.	2000	Does not include males and females
Sarcopenia, age, atrophy, and myopathy: Mitochondrial oxidative enzyme activities	Pestronk, A.; Keeling, R.; Choksi, R.	2017	Does not report sex of subjects
Human skeletal muscle pyruvate dehydrogenase kinase activity increases after a low-carbohydrate diet	Peters, S. J.; St Amand, T. A.; Howlett, R. A.; Heigenhauser, G. J.; Spriet, L. L.	1998	Did not perform fiber type analysis
Infusion with the antioxidant N-acetylcysteine attenuates early adaptive responses to exercise in human skeletal muscle	Petersen, A. C.; McKenna, M. J.; Medved, I.; Murphy, K. T.; Brown, M. J.; Della Gatta, P.; Cameron-Smith, D.	2012	Does not include males and females
Depressed Na+-K+-ATPase activity in skeletal muscle at fatigue is correlated with increased Na+-K+-ATPase mRNA expression following intense exercise	Petersen, A. C.; Murphy, K. T.; Snow, R. J.; Leppik, J. A.; Aughey, R. J.; Garnham, A. P.; Cameron-Smith, D.; McKenna, M. J.	2005	Did not perform fiber type analysis

Human skeletal muscle protein: effect of malnutrition, elective surgery and total parenteral nutrition	Petersson, B.; Hultman, E.; Andersson, K.; Wernerman, J.	1995	No healthy subjects or controls
Long-term changes in muscle free amino acid levels after elective abdominal surgery	Petersson, B.; Vinnars, E.; Waller, S. O.; Wernerman, J.	1992	No healthy subjects or controls
Distinct Skeletal Muscle Gene Regulation from Active Contraction, Passive Vibration, and Whole Body Heat Stress in Humans	Petrie, M. A.; Kimball, A. L.; McHenry, C. L.; Suneja, M.; Yen, C. L.; Sharma, A.; Shields, R. K.	2016	Does not include males and females
Alpha-cardiac-like myosin heavy chain as an intermediate between MHCIIa and MHCI beta in transforming rabbit muscle	Peuker, H.; Conjard, A.; Pette, D.	1998	Animal study
Aldosterone increases Na+ -K+ -ATPase activity in skeletal muscle of patients with Conn's syndrome	Phakdeekitcharoen, B.; Kittikanokrat, W.; Kijkunasathian, C.; Chatsudthipong, V.	2011	Did not perform fiber type analysis
Thyroid hormone increases mRNA and protein expression of Na+-K+-ATPase alpha2 and beta1 subunits in human skeletal muscles	Phakdeekitcharoen, B.; Phudhichareonrat, S.; Pookarnjanamorakot, C.; Kijkunasathian, C.; Tubtong, N.; Kittikanokrat, W.; Radinahamed, P.	2007	No healthy subjects or controls
Effects of Roux-en-Y gastric bypass on circulating follistatin, activin A, and peripheral ActRIIB signaling in humans with obesity and type 2 diabetes	Pham, T. C. P.; Bojsen-MÃ¸ller, K. N.; Madsbad, S.; Wojtaszewski, J. F. P.; Richter, E. A.; Sylow, L.	2021	Did not perform fiber type analysis
Myotoxic reactions to lipid-lowering therapy are associated with altered oxidation of fatty acids	Phillips, P. S.; Ciaraldi, T. P.; Kim, D. L.; Verity, M. A.; Wolfson, T.; Henry, R. R.	2009	Does not report sex of subjects
Adipocyte differentiation-related protein in human skeletal muscle: relationship to insulin sensitivity	Phillips, S. A.; Choe, C. C.; Ciaraldi, T. P.; Greenberg, A. S.; Kong, A. P.; Baxi, S. C.; Christiansen, L.; Mudaliar, S. R.; Henry, R. R.	2005	Does not include male and female data
Respiratory and skeletal muscle function in steroid-dependent bronchial asthma	Picado, C.; Fiz, J. A.; Montserrat, J. M.; Grau, J. M.; Fernandez-Sola, J.; Luengo, M. T.; Casademont, J.; Agusti-Vidal, A.	1990	Did not perform muscle biopsy
Altered Expression of Mitoferrin and Frataxin, Larger Labile Iron Pool and Greater Mitochondrial DNA Damage in the Skeletal Muscle of Older Adults	Picca, A.; Saini, S. K.; Mankowski, R. T.; Kamenov, G.; Anton, S. D.; Manini, T. M.; Buford, T. W.; Wohlgemuth, S. E.; Xiao, R.; Calvani, R.; Coelho-JÃºnior, H. J.; Landi, F.; Bernabei, R.; Hood, D. A.; Marzetti, E.; Leeuwenburgh, C.	2020	Did not perform fiber type analysis
Time course for refilling of glycogen stores in human muscle fibres following exercise-induced glycogen depletion	Piehl, K.	1974	Does not include males and females
Hyaluronan in human skeletal muscle of lower extremity: concentration, distribution, and effect of exercise	Piehl-Aulin, K.; Laurent, C.; EngstrÃ¶m-Laurent, A.; HellstrÃ¶m, S.; Henriksson, J.	1991	Does not include male and female data
Desmin immunolocalisation in autosomal dominant Emery-Dreifuss muscular dystrophy	Piercy, R. J.; Zhou, H.; Feng, L.; Pombo, A.; Muntoni, F.; Brown, S. C.	2007	No healthy subjects or controls
Effects of local vibrations on skeletal muscle trophism in elderly people: mechanical, cellular, and molecular events	Pietrangelo, T.; Mancinelli, R.; Toniolo, L.; Cancellara, L.; Paoli, A.; Puglielli, C.; Iodice, P.; Doria, C.; Bosco, G.; D'Amelio, L.; di Tano, G.; Fulle, S.; Saggini, R.; FanÃ², G.; Reggiani, C.	2009	No healthy subjects or controls

A method for the ultrastructural preservation of tiny percutaneous needle biopsy material from skeletal muscle	Pietrangelo, T.; Perni, S.; Di Tano, G.; FanÃ²-Illic, G.; Franzini-Armstrong, C.	2013	Does not include males and females
Acute effects of dietary fat on inflammatory markers and gene expression in first-degree relatives of type 2 diabetes patients	Pietraszek, A.; Gregersen, S.; Hermansen, K.	2011	Did not perform fiber type analysis
Effects of a meal rich in medium-chain saturated fat on postprandial lipemia in relatives of type 2 diabetics	Pietraszek, A.; Hermansen, K.; Pedersen, S. B.; Langdahl, B. L.; Holst, J. J.; Gregersen, S.	2013	Did not perform fiber type analysis
Low-load resistance training to task failure with and without blood flow restriction: muscular functional and structural adaptations	Pignanelli, C.; Petrick, H. L.; Keyvani, F.; Heigenhauser, G. J. F.; Quadrilatero, J.; Holloway, G. P.; Burr, J. F.	2020	Does not include males and females
Lactate transport studied in sarcolemmal giant vesicles from human muscle biopsies: relation to training status	Pilegaard, H.; Bangsbo, J.; Richter, E. A.; Juel, C.	1994	Does not include males and females
Effect of high-intensity exercise training on lactate/H+ transport capacity in human skeletal muscle	Pilegaard, H.; Domino, K.; Noland, T.; Juel, C.; Hellsten, Y.; Halestrap, A. P.; Bangsbo, J.	1999	Does not include males and females
Lactate/H+ transport in skeletal muscle from spinal-cord-injured patients	Pilegaard, H.; Mohr, T.; Kjaer, M.; Juel, C.	1998	Does not include males and females
Residual force enhancement and force depression in human single muscle fibres	Pinnell, R. A. M.; Mashouri, P.; Mazara, N.; Weersink, E.; Brown, S. H. M.; Power, G. A.	2019	Does not include males and females
Abnormal mitochondrial respiration in skeletal muscle in patients with peripheral arterial disease	Pipinos,, II; Sharov, V. G.; Shepard, A. D.; Anagnostopoulos, P. V.; Katsamouris, A.; Todor, A.; Filis, K. A.; Sabbah, H. N.	2003	No healthy subjects or controls
Ryanodine receptor fragmentation and sarcoplasmic reticulum Ca2+ leak after one session of high-intensity interval exercise	Place, N.; Ivarsson, N.; Venckunas, T.; Neyroud, D.; Brazaitis, M.; Cheng, A. J.; Ochala, J.; Kamandulis, S.; Girard, S.; VolungeviÄius, G.; PauÅ¾as, H.; Mekideche, A.; Kayser, B.; Martinez-Redondo, V.; Ruas, J. L.; Bruton, J.; Truffert, A.; Lanner, J. T.; Skurvydas, A.; Westerblad, H.	2015	Does not include males and females
Cellular markers of muscle atrophy in chronic obstructive pulmonary disease	Plant, P. J.; Brooks, D.; Faughnan, M.; Bayley, T.; Bain, J.; Singer, L.; Correa, J.; Pearce, D.; Binnie, M.; Batt, J.	2010	Did not perform fiber type analysis
Muscle contractile properties as an explanation of the higher mean power output in marmosets than humans during jumping	Plas, R. L.; Degens, H.; Meijer, J. P.; de Wit, G. M.; Philippens, I. H.; Bobbert, M. F.; Jaspers, R. T.	2015	Does not include male and female data
Electron-microscopic investigation of muscle mitochondria in chronic fatigue syndrome	Plioplys, A. V.; Plioplys, S.	1995	Did not perform fiber type analysis
Associations between insulin resistance and TNF-alpha in plasma, skeletal muscle and adipose tissue in humans with and without type 2 diabetes	Plomgaard, P.; Nielsen, A. R.; Fischer, C. P.; Mortensen, O. H.; Broholm, C.; Penkowa, M.; Krogh-Madsen, R.; Erikstrup, C.; Lindegaard, B.; Petersen, A. M.; Taudorf, S.; Pedersen, B. K.	2007	Does not report sex of subjects
Autoantibodies to cytosolic 5'-nucleotidase 1A in inclusion body myositis	Pluk, H.; van Hoeve, B. J.; van Dooren, S. H.; Stammen-Vogelzangs, J.; van der Heijden, A.; Schelhaas, H. J.; Verbeek, M. M.;	2013	Did not perform fiber type analysis

	Badrising, U. A.; Arnardottir, S.; Gheorghe, K.; Lundberg, I. E.; Boelens, W. C.; van Engelen, B. G.; Pruijn, G. J.		
Resveratrol Improves Vascular Function and Mitochondrial Number but Not Glucose Metabolism in Older Adults	Pollack, R. M.; Barzilai, N.; Anghel, V.; Kulkarni, A. S.; Golden, A.; O'Broin, P.; Sinclair, D. A.; Bonkowski, M. S.; Coleville, A. J.; Powell, D.; Kim, S.; Moaddel, R.; Stein, D.; Zhang, K.; Hawkins, M.; Crandall, J. P.	2017	Did not perform fiber type analysis
Iron metabolism gene expression in human skeletal muscle	Polonifi, A.; Politou, M.; Kalotychou, V.; Xiromeritis, K.; Tsironi, M.; Berdoukas, V.; Vaiopoulos, G.; Aessopos, A.	2010	No healthy subjects or controls
Reduced myotube diameter, atrophic signalling and elevated oxidative stress in cultured satellite cells from COPD patients	PomiÃ¨s, P.; Rodriguez, J.; BlaquiÃ¨re, M.; Sedraoui, S.; Gouzi, F.; Carnac, G.; Laoudj-Chenivesse, D.; Mercier, J.; PrÃ©faut, C.; Hayot, M.	2015	Does not include male and female data
Impairment of maximal aerobic power with moderate hypoxia in endurance athletes: do skeletal muscle mitochondria play a role?	Ponsot, E.; Dufour, S. P.; Doutreleau, S.; Lonsdorfer-Wolf, E.; Lampert, E.; Piquard, F.; Geny, B.; Mettauer, B.; Ventura-Clapier, R.; Richard, R.	2010	Does not include males and females
Exercise training in normobaric hypoxia in endurance runners. II. Improvement of mitochondrial properties in skeletal muscle	Ponsot, E.; Dufour, S. P.; Zoll, J.; Doutrelau, S.; N'Guessan, B.; Geny, B.; Hoppeler, H.; Lampert, E.; Mettauer, B.; Ventura-Clapier, R.; Richard, R.	2006	Does not include males and females
Telomere length and regulatory proteins in human skeletal muscle with and without ongoing regenerative cycles	Ponsot, E.; Echaniz-Laguna, A.; Delis, A. M.; Kadi, F.	2012	Did not perform fiber type analysis
Decreased capillarization and a shift to fast myosin heavy chain IIx in the biceps brachii muscle from young adults with spastic paresis	PontÃ©n, E. M.; StÃ¥l, P. S.	2007	Does not include males and females
Needle biopsy of skeletal muscle: a phase and electron microscopic evaluation of its usefulness in the study of muscle dsease	Porro, R. S.; Webster, H. F.; Tobin, W.	1969	No healthy subjects or controls
Uncoupled skeletal muscle mitochondria contribute to hypermetabolism in severely burned adults	Porter, C.; Herndon, D. N.; BÃ¸rsheim, E.; Chao, T.; Reidy, P. T.; Borack, M. S.; Rasmussen, B. B.; Chondronikola, M.; Saraf, M. K.; Sidossis, L. S.	2014	Does not include males and females
Biopsy sampling requirements for the estimation of muscle capillarization	Porter, M. M.; Koolage, C. W.; Lexell, J.	2002	Does not include male and female data
Structural and molecular study of the supraspinatus muscle of modern humans (Homo sapiens) and common chimpanzees (Pan troglodytes)	Potau, J. M.; Casado, A.; de Diego, M.; Ciurana, N.; Arias-Martorell, J.; Bello-Hellegouarch, G.; Barbosa, M.; de Paz, F. J.; Pastor, J. F.; PÃ©rez-PÃ©rez, A.	2018	Autopsies
Overweight in elderly people induces impaired autophagy in skeletal muscle	Potes, Y.; de LuxÃ¡n-Delgado, B.; Rodriguez-GonzÃ¡lez, S.; GuimarÃ£es, M. R. M.; Solano, J. J.; FernÃ¡ndez-FernÃ¡ndez, M.; BermÃºdez, M.; Boga, J. A.; Vega-Naredo, I.; Coto-Montes, A.	2017	Did not perform fiber type analysis
Elevated inosine monophosphate levels in resting muscle of patients with stable chronic obstructive pulmonary disease	Pouw, E. M.; Schols, A. M.; van der Vusse, G. J.; Wouters, E. F.	1998	Does not include males and females

Role of hexosamines in insulin resistance and nutrient sensing in human adipose and muscle tissue	Pouwels, M. J.; Tack, C. J.; Span, P. N.; Olthaar, A. J.; Sweep, C. G.; Huvers, F. C.; Lutterman, J. A.; Hermus, A. R.	2004	Did not perform fiber type analysis
Tissue-engineered human bioartificial muscles expressing a foreign recombinant protein for gene therapy	Powell, C.; Shansky, J.; Del Tatto, M.; Forman, D. E.; Hennessey, J.; Sullivan, K.; Zielinski, B. A.; Vandenburgh, H. H.	1999	Does not include males and females
31P MR spectroscopy and in vitro markers of oxidative capacity in type 2 diabetes patients	Praet, S. F.; De Feyter, H. M.; Jonkers, R. A.; Nicolay, K.; van Pul, C.; Kuipers, H.; van Loon, L. J.; Prompers, J. J.	2006	Does not include males and females
In Vivo Visualization of Tissue Damage Induced by Percutaneous Muscle Biopsy via Novel High-Resolution MR Imaging	Preisner, F.; Friedmann-Bette, B.; Wehrstein, M.; Vollherbst, D. F.; Heiland, S.; Bendszus, M.; Hilgenfeld, T.	2021	Does not include males and females
NanoString technology distinguishes anti-TIF-1Î³(+) from anti-Mi-2(+) dermatomyositis patients	Preusse, C.; Eede, P.; Heinzeling, L.; Freitag, K.; Koll, R.; Froehlich, W.; Schneider, U.; Allenbach, Y.; Benveniste, O.; SchÃ¤nzer, A.; Goebel, H. H.; Stenzel, W.; Radke, J.	2021	No cross-sectional area data
Th2-M2 immunity in lesions of muscular sarcoidosis and macrophagic myofasciitis	Preusse, C.; Goebel, H. H.; Pehl, D.; Rinnenthal, J. L.; Kley, R. A.; Allenbach, Y.; Heppner, F. L.; Vorgerd, M.; Authier, F. J.; Gherardi, R.; Stenzel, W.	2015	Does not report sex of subjects
Myopathy with atypical mitochondria in type I skeletal muscle fibers. A histochemical and ultrastructural study	Price, H. M.; Gordon, G. R.; Munsat, T. L.; Pearson, C. M.	1967	No healthy subjects or controls
Low clonogenic potential of circulating angiogenic cells is associated with lower density of capillaries in skeletal muscle in patients with impaired glucose tolerance	Prior, S. J.; Ryan, A. S.	2013	Did not perform fiber type analysis
Sarcopenia Is Associated With Lower Skeletal Muscle Capillarization and Exercise Capacity in Older Adults	Prior, S. J.; Ryan, A. S.; Blumenthal, J. B.; Watson, J. M.; Katzel, L. I.; Goldberg, A. P.	2016	Does not include male and female data
Annexin expression in inflammatory myopathies	Probst-Cousin, S.; Berghoff, C.; NeundÃ¶rfer, B.; Heuss, D.	2004	Does not report sex of subjects
Skeletal muscle IL-4, IL-4Ralpha, IL-13 and IL-13Ralpha1 expression and response to strength training	Prokopchuk, O.; Liu, Y.; Wang, L.; Wirth, K.; Schmidtbleicher, D.; Steinacker, J. M.	2007	Does not include males and females
Mitochondrial changes in platelets are not related to those in skeletal muscle during human septic shock	Protti, A.; Fortunato, F.; Caspani, M. L.; Pluderi, M.; Lucchini, V.; Grimoldi, N.; Solimeno, L. P.; Fagiolari, G.; Ciscato, P.; Zella, S. M.; Moggio, M.; Comi, G. P.; Gattinoni, L.	2014	Did not perform fiber type analysis
Adding strength to endurance training does not enhance aerobic capacity in cyclists	Psilander, N.; Frank, P.; Flockhart, M.; Sahlin, K.	2015	Does not include males and females
Abnormal mitochondrial function in locomotor and respiratory muscles of COPD patients	Puente-Maestu, L.; PÃ©rez-Parra, J.; Godoy, R.; Moreno, N.; Tejedor, A.; GonzÃ¡lez-Aragoneses, F.; Bravo, J. L.; Alvarez, F. V.; CamaÃ±o, S.; AgustÃ, A.	2009	Does not report sex of subjects
Epigenetic mechanisms in respiratory muscle dysfunction of patients with chronic obstructive pulmonary disease	Puig-Vilanova, E.; AguilÃ³, R.; RodrÃguez-Fuster, A.; MartÃnez-Llorens, J.; Gea, J.; Barreiro, E.	2014	Does not include male and female data

Quadriceps muscle weakness and atrophy are associated with a differential epigenetic profile in advanced COPD	Puig-Vilanova, E.; MartÃnez-Llorens, J.; Ausin, P.; Roca, J.; Gea, J.; Barreiro, E.	2015	Does not include males and females
Microstructural Changes of Tensor Fasciae Latae and Gluteus Medius Muscles Following Total Hip Arthroplasty: A Prospective Trial	Pumberger, M.; V. O. N. Roth P; Preininger, B.; Mueller, M.; Perka, C.; Winkler, T.	2017	No healthy subjects or controls
Succinate Dehydrogenase B (SDHB) Immunohistochemistry for the Evaluation of Muscle Biopsies	Punsoni, M.; Mangray, S.; Lombardo, K. A.; Heath, N.; Stopa, E. G.; Yakirevich, E.	2017	Did not perform fiber type analysis
An efficient polymerase chain reaction approach for the quantitation of multiple RNAs in human tissue samples	Puntschart, A.; Jostarndt, K.; Hoppeler, H.; Billeter, R.	1994	Does not report sex of subjects
The effects of exercise and adipose tissue lipolysis on plasma adiponectin concentration and adiponectin receptor expression in human skeletal muscle	Punyadeera, C.; Zorenc, A. H.; Koopman, R.; McAinch, A. J.; Smit, E.; Manders, R.; Keizer, H. A.; Cameron-Smith, D.; van Loon, L. J.	2005	Does not include males and females
Skeletal muscle pyruvate dehydrogenase activity during maximal exercise in humans	Putman, C. T.; Jones, N. L.; Lands, L. C.; Bragg, T. M.; Hollidge-Horvat, M. G.; Heigenhauser, G. J.	1995	Does not include males and females
INa and IKir are reduced in Type 1 hypokalemic and thyrotoxic periodic paralysis	Puwanant, A.; Ruff, R. L.	2010	Did not perform fiber type analysis
The impact of gastrocnemius muscle cell changes in chronic venous insufficiency	Qiao, T.; Liu, C.; Ran, F.	2005	Does not report sex of subjects
Prolonged moderate-intensity aerobic exercise does not alter apoptotic signaling and DNA fragmentation in human skeletal muscle	Quadrilatero, J.; Bombardier, E.; Norris, S. M.; Talanian, J. L.; Palmer, M. S.; Logan, H. M.; Tupling, A. R.; Heigenhauser, G. J.; Spriet, L. L.	2010	Does not include male and female data
Nutritional status, muscle composition and plasma and muscle free amino acids in renal transplant patients	Qureshi, A. R.; Lindholm, B.; Alvestrand, A.; BergstrÃ¶m, J.; Tollemar, J.; Hultman, E.; Groth, C. G.	1994	Did not perform fiber type analysis
Mitochondrial dysfunction in COPD patients with low body mass index	Rabinovich, R. A.; Bastos, R.; Ardite, E.; LlinÃ s, L.; Orozco-Levi, M.; Gea, J.; VilarÃ³, J.; BarberÃ , J. A.; RodrÃguez-Roisin, R.; FernÃ¡ndez-Checa, J. C.; Roca, J.	2007	Does not include males and females
Genome-wide mRNA expression profiling in vastus lateralis of COPD patients with low and normal fat free mass index and healthy controls	Rabinovich, R. A.; Drost, E.; Manning, J. R.; Dunbar, D. R.; DÃaz-Ramos, M.; Lakhdar, R.; Bastos, R.; MacNee, W.	2015	Does not include male and female data
Opposite effects of pioglitazone and rosiglitazone on mitochondrial respiration in skeletal muscle of patients with type 2 diabetes	RabÃ¸l, R.; Boushel, R.; Almdal, T.; Hansen, C. N.; Ploug, T.; Haugaard, S. B.; Prats, C.; Madsbad, S.; Dela, F.	2010	Did not perform fiber type analysis
Regional anatomic differences in skeletal muscle mitochondrial respiration in type 2 diabetes and obesity	RabÃ¸l, R.; Larsen, S.; HÃ¸jberg, P. M.; Almdal, T.; Boushel, R.; Haugaard, S. B.; Andersen, J. L.; Madsbad, S.; Dela, F.	2010	Does not include male and female data
Human septic myopathy: induction of cyclooxygenase, heme oxygenase and activation of the ubiquitin proteolytic pathway	Rabuel, C.; Renaud, E.; Brealey, D.; Ratajczak, P.; Damy, T.; Alves, A.; Habib, A.; Singer, M.; Payen, D.; Mebazaa, A.	2004	Did not perform fiber type analysis
Skeletal muscle telomere length in healthy, experienced, endurance runners	Rae, D. E.; Vignaud, A.; Butler-Browne, G. S.; Thornell, L. E.; Sinclair-Smith, C.; Derman, E. W.; Lambert, M. I.; Collins, M.	2010	Did not perform fiber type analysis
Sarcomeric myosin expression in the tongue body of humans, macaques and rats	Rahnert, J. A.; Sokoloff, A. J.; Burkholder, T. J.	2010	Autopsies

Markers of inflammation, proteolysis, and apoptosis in ESRD	Raj, D. S.; Shah, H.; Shah, V. O.; Ferrando, A.; Bankhurst, A.; Wolfe, R.; Zager, P. G.	2003	Does not include males and females
Depressed aerobic enzyme activity of skeletal muscle in severe chronic heart failure	Ralston, M. A.; Merola, A. J.; Leier, C. V.	1991	Did not perform fiber type analysis
Ultrastructural changes in paravertebral muscles associated with degenerative spondylolisthesis	Ramsbacher, J.; Theallier-Janko, A.; Stoltenburg-Didinger, G.; Brock, M.	2001	No healthy subjects or controls
Effects of AMPD1 common mutation on the metabolic-chronotropic relationship: Insights from patients with myoadenylate deaminase deficiency	Rannou, F.; Scotet, V.; Marcorelles, P.; Monnoyer, R.; Le MarÃ©chal, C.	2017	Did not perform fiber type analysis
Mild hypothermia affects the morphology and impairs glutamine-induced anabolic response in human primary myotubes	Rantala, R.; Chaillou, T.	2019	Does not include male and female data
Lumbar muscle fiber size and type distribution in normal subjects	Rantanen, J.; Rissanen, A.; Kalimo, H.	1994	Autopsies
Masseter myosin heavy chain composition varies with mandibular asymmetry	Raoul, G.; Rowlerson, A.; Sciote, J.; Codaccioni, E.; Stevens, L.; Maurage, C. A.; Duhamel, A.; Ferri, J.	2011	No healthy subjects or controls
Malonyl coenzyme A and the regulation of functional carnitine palmitoyltransferase-1 activity and fat oxidation in human skeletal muscle	Rasmussen, B. B.; HolmbÃ¤ck, U. C.; Volpi, E.; Morio-Liondore, B.; Paddon-Jones, D.; Wolfe, R. R.	2002	Did not perform fiber type analysis
Oral and intravenously administered amino acids produce similar effects on muscle protein synthesis in the elderly	Rasmussen, B. B.; Wolfe, R. R.; Volpi, E.	2002	Did not perform fiber type analysis
TWEAK-Fn14 pathway activation after exercise in human skeletal muscle: insights from two exercise modes and a time course investigation	Raue, U.; Jemiolo, B.; Yang, Y.; Trappe, S.	2015	Does not include male and female data
The concentration of sodium, potassium pumps in chronic obstructive lung disease (COLD) patients: the impact of magnesium depletion and steroid treatment	Ravn, H. B.; DÃ¸rup, I.	1997	Did not perform fiber type analysis
Atx regulates skeletal muscle regeneration via LPAR1 and promotes hypertrophy	Ray, R.; Sinha, S.; Aidinis, V.; Rai, V.	2021	Does not include male and female data
Comparative gene expression profiling between human cultured myotubes and skeletal muscle tissue	Raymond, F.; MÃ©tairon, S.; Kussmann, M.; Colomer, J.; Nascimento, A.; Mormeneo, E.; GarcÃa-MartÃnez, C.; GÃ³mez-Foix, A. M.	2010	Does not include males and females
Increased levels of leukemia inhibitory factor mRNA in muscular dystrophy and human muscle trauma	Reardon, K. A.; Kapsa, R. M.; Davis, J.; Kornberg, A. J.; Austin, L.; Choong, P.; Byrne, E.	2000	Did not perform fiber type analysis
Creatine supplementation does not enhance submaximal aerobic training adaptations in healthy young men and women	Reardon, T. F.; Ruell, P. A.; Fiatarone Singh, M. A.; Thompson, C. H.; Rooney, K. B.	2006	Did not perform fiber type analysis
Chronic changes in skeletal muscle histology and function in peripheral arterial disease	Regensteiner, J. G.; Wolfel, E. E.; Brass, E. P.; Carry, M. R.; Ringel, S. P.; Hargarten, M. E.; Stamm, E. R.; Hiatt, W. R.	1993	Does not include males and females

Calcium handling in muscle fibres of mice and men: evolutionary adaptation in different species to optimize performance and save energy	Reggiani, C.	2014	Review (ex: systematic review/meta-analysis)
A novel ultrastructural finding in statin-exposed patients with inflammatory myositis	Rehman, Z. U.; Blumbergs, P.; Swift, J.; Otto, S.; Smith, C.; Koszyca, B.; Manavis, J.; Limaye, V.	2016	Not peer reviewed (ex: thesis, conference poster)
High-energy phosphate compounds in human slow-twitch and fast-twitch muscle fibres	Rehunen, S.; HÃ¤rkÃ¶nen, M.	1980	No cross-sectional area data
High-energy phosphate compounds in slow-twitch and fast-twitch muscle fibres. Changes during exercise in some neuromuscular diseases	Rehunen, S.; Karli, P.; HÃ¤rkÃ¶nen, M.	1985	Does not include males and females
High-energy phosphate compounds during exercise in human slow-twitch and fast-twitch muscle fibres	Rehunen, S.; NÃ¤veri, H.; Kuoppasalmi, K.; HÃ¤rkÃ¶nen, M.	1982	Does not include male and female data
Unaltered respiratory chain enzyme activity and mitochondrial DNA in skeletal muscle from patients with idiopathic Parkinson's syndrome	Reichmann, H.; Janetzky, B.; Bischof, F.; Seibel, P.; SchÃ¶ls, L.; Kuhn, W.; Przuntek, H.	1994	No healthy subjects or controls
Aging-related effects of bed rest followed by eccentric exercise rehabilitation on skeletal muscle macrophages and insulin sensitivity	Reidy, P. T.; Lindsay, C. C.; McKenzie, A. I.; Fry, C. S.; Supiano, M. A.; Marcus, R. L.; LaStayo, P. C.; Drummond, M. J.	2018	Does not include male and female data
An accumulation of muscle macrophages is accompanied by altered insulin sensitivity after reduced activity and recovery	Reidy, P. T.; Yonemura, N. M.; Madsen, J. H.; McKenzie, A. I.; Mahmassani, Z. S.; Rondina, M. T.; Lin, Y. K.; Kaput, K.; Drummond, M. J.	2019	Does not include male and female data
Macrophage migration inhibitory factor in normal human skeletal muscle and inflammatory myopathies	Reimann, J.; Schnell, S.; Schwartz, S.; Kappes-Horn, K.; Dodel, R.; Bacher, M.	2010	Did not perform fiber type analysis
Skeletal muscle sonography: a correlative study of echogenicity and morphology	Reimers, K.; Reimers, C. D.; Wagner, S.; Paetzke, I.; Pongratz, D. E.	1993	No healthy subjects or controls
Classical NF-ÎºB activation impairs skeletal muscle oxidative phenotype by reducing IKK-Î± expression	Remels, A. H.; Gosker, H. R.; Langen, R. C.; Polkey, M.; Sliwinski, P.; Galdiz, J.; van den Borst, B.; Pansters, N. A.; Schols, A. M.	2014	Does not include male and female data
NADH content in type I and type II human muscle fibres after dynamic exercise	Ren, J. M.; Henriksson, J.; Katz, A.; Sahlin, K.	1988	Does not include males and females
Expression and subcellular localization of P9-ZFD protein in patients with myasthenia gravis	Ren, M. S.; Lu, C. Z.; Qiao, J.; Ren, H. M.; Xu, R.; Gan, R. B.	2004	No healthy subjects or controls
Regenerative potential of human skeletal muscle during aging	Renault, V.; Thornell, L. E.; Eriksson, P. O.; Butler-Browne, G.; Mouly, V.	2002	Autopsies
Histochemical and quantitative analysis of muscle biopsies	Reniers, J.; Martin, L.; Joris, C.	1970	Does not include male and female data
Receptor and post-receptor abnormalities contribute to insulin resistance in myotonic dystrophy type 1 and type 2 skeletal muscle	Renna, L. V.; BosÃ¨, F.; Iachettini, S.; Fossati, B.; Saraceno, L.; Milani, V.; Colombo, R.; Meola, G.; Cardani, R.	2017	No cross-sectional area data

Premature senescence in primary muscle cultures of myotonic dystrophy type 2 is not associated with p16 induction	Renna, L. V.; Cardani, R.; Botta, A.; Rossi, G.; Fossati, B.; Costa, E.; Meola, G.	2014	Does not report sex of subjects
Thigh muscle atrophy in below-knee amputees	RenstrÃ¶m, P.; Grimby, G.; Morelli, B.; Palmertz, B.	1983	Does not include male and female data
Malignant hyperthermia in a family. The ultrastructure of muscle biopsies of healthy members	Reske-Nielsen, E.; Haase, J.; Kelstrup, J.	1975	No healthy subjects or controls
Ultrastructure of muscle biopsies in recent, short-term and long-term juvenile diabetes	Reske-Nielsen, E.; Harmsen, A.; Vorre, P.	1977	No healthy subjects or controls
The ryanodine contracture test may help diagnose susceptibility to malignant hyperthermia	Reuter, D. A.; Anetseder, M.; MÃ¼ller, R.; Roewer, N.; Hartung, E. J.	2003	No healthy subjects or controls
Elevated toll-like receptor 4 expression and signaling in muscle from insulin-resistant subjects	Reyna, S. M.; Ghosh, S.; Tantiwong, P.; Meka, C. S.; Eagan, P.; Jenkinson, C. P.; Cersosimo, E.; Defronzo, R. A.; Coletta, D. K.; Sriwijitkamol, A.; Musi, N.	2008	Did not perform fiber type analysis
Obesity, type 2 diabetes, and impaired insulin-stimulated blood flow: role of skeletal muscle NO synthase and endothelin-1	Reynolds, L. J.; Credeur, D. P.; Manrique, C.; Padilla, J.; Fadel, P. J.; Thyfault, J. P.	2017	Did not perform fiber type analysis
Effects of intense and prolonged exercise on insulin sensitivity and glycogen metabolism in hypertensive subjects	RhÃ©aume, C.; Waib, P. H.; KouamÃ©, N.; Nadeau, A.; LacourciÃ¨re, Y.; Joanisse, D. R.; Simoneau, J. A.; ClÃ©roux, J.	2003	Does not include males and females
Carbon monoxide, skeletal muscle oxidative stress, and mitochondrial biogenesis in humans	Rhodes, M. A.; Carraway, M. S.; Piantadosi, C. A.; Reynolds, C. M.; Cherry, A. D.; Wester, T. E.; Natoli, M. J.; Massey, E. W.; Moon, R. E.; Suliman, H. B.	2009	Does not include male and female data
Timing of Creatine Supplementation around Exercise: A Real Concern?	Ribeiro, F.; Longobardi, I.; Perim, P.; Duarte, B.; Ferreira, P.; Gualano, B.; Roschel, H.; Saunders, B.	2021	Review (ex: systematic review/meta-analysis)
Lipid infusion decreases the expression of nuclear encoded mitochondrial genes and increases the expression of extracellular matrix genes in human skeletal muscle	Richardson, D. K.; Kashyap, S.; Bajaj, M.; Cusi, K.; Mandarino, S. J.; Finlayson, J.; DeFronzo, R. A.; Jenkinson, C. P.; Mandarino, L. J.	2005	Did not perform fiber type analysis
Reduced mechanical efficiency in chronic obstructive pulmonary disease but normal peak VO2 with small muscle mass exercise	Richardson, R. S.; Leek, B. T.; Gavin, T. P.; Haseler, L. J.; Mudaliar, S. R.; Henry, R.; Mathieu-Costello, O.; Wagner, P. D.	2004	Does not report sex of subjects
Human VEGF gene expression in skeletal muscle: effect of acute normoxic and hypoxic exercise	Richardson, R. S.; Wagner, H.; Mudaliar, S. R.; Henry, R.; Noyszewski, E. A.; Wagner, P. D.	1999	Does not include males and females
Exercise adaptation attenuates VEGF gene expression in human skeletal muscle	Richardson, R. S.; Wagner, H.; Mudaliar, S. R.; Saucedo, E.; Henry, R.; Wagner, P. D.	2000	Does not include males and females
p38 mitogen-activated protein kinase is not activated in the quadriceps of patients with stable chronic obstructive pulmonary disease	Riddoch-Contreras, J.; George, T.; Natanek, S. A.; Marsh, G. S.; Hopkinson, N. S.; Tal-Singer, R.; Kemp, P.; Polkey, M. I.	2012	Did not perform fiber type analysis
Influence of exercise on the fiber composition of skeletal muscle	Riedy, M.; Matoba, H.; VÃ¸llestad, N. K.; Oakley, C. R.; Blank, S.; Hermansen, L.; Gollnick, P. D.	1984	Does not include males and females

Insulin acutely regulates the expression of the peroxisome proliferator-activated receptor-gamma in human adipocytes	Rieusset, J.; Andreelli, F.; Auboeuf, D.; Roques, M.; Vallier, P.; Riou, J. P.; Auwerx, J.; Laville, M.; Vidal, H.	1999	Did not perform fiber type analysis
Ragged red fibers in normal aging and inflammatory myopathy	Rifai, Z.; Welle, S.; Kamp, C.; Thornton, C. A.	1995	Did not perform fiber type analysis
Distinct interferon signatures stratify inflammatory and dysimmune myopathies	Rigolet, M.; Hou, C.; Baba Amer, Y.; Aouizerate, J.; Periou, B.; Gherardi, R. K.; Lafuste, P.; Authier, F. J.	2019	Does not include male and female data
Hyperthyroidism and cation pumps in human skeletal muscle	Riis, A. L.; JÃ¸rgensen, J. O.; MÃ¸ller, N.; Weeke, J.; Clausen, T.	2005	Did not perform fiber type analysis
Disproportionate loss of thin filaments in human soleus muscle after 17-day bed rest	Riley, D. A.; Bain, J. L.; Thompson, J. L.; Fitts, R. H.; Widrick, J. J.; Trappe, S. W.; Trappe, T. A.; Costill, D. L.	1998	Does not include males and females
Decreased thin filament density and length in human atrophic soleus muscle fibers after spaceflight	Riley, D. A.; Bain, J. L.; Thompson, J. L.; Fitts, R. H.; Widrick, J. J.; Trappe, S. W.; Trappe, T. A.; Costill, D. L.	2000	Does not include males and females
Thin filament diversity and physiological properties of fast and slow fiber types in astronaut leg muscles	Riley, D. A.; Bain, J. L.; Thompson, J. L.; Fitts, R. H.; Widrick, J. J.; Trappe, S. W.; Trappe, T. A.; Costill, D. L.	2002	Does not include males and females
Analysis of Single Nucleotide Polymorphisms (SNPs) of the small-conductance calcium activated potassium channel (SK3) gene as genetic modifier of the cardiac phenotype in myotonic dystrophy type 1 patients	Rinaldi, F.; Botta, A.; Vallo, L.; Contino, G.; Morgante, A.; Iraci, R.; Catalli, C.; Silvestri, G.; Ventriglia, V. M.; Politano, L.; Novelli, G.	2008	No healthy subjects or controls
Altered skeletal muscle glucose transport and blood lipid levels in habitual cigarette smokers	RincÃ³n, J.; Krook, A.; Galuska, D.; Wallberg-Henriksson, H.; Zierath, J. R.	1999	Does not include males and females
Hodgkin's disease treated with neck radiation is associated with increased antibody-dependent cellular cytotoxicity against human extraocular muscle cells	Ringel, M. D.; Taylor, T.; Barsouk, A.; Wall, J. R.; Freter, C. E.; Howard, R. S.; Diehl, L.; Burman, K. D.	1997	Did not perform fiber type analysis
Histochemical fiber types and fiber sizes in human masticatory muscles	Ringqvist, M.	1971	No cross-sectional area data
Fibre sizes of human masseter muscle in relation to bite force	Ringqvist, M.	1973	Does not include male and female data
Histochemical enzyme profiles of fibres in human masseter muscles with special regard to fibres with intermediate myofibrillar ATPase reaction	Ringqvist, M.	1973	Does not include male and female data
Size and distribution of histochemical fibre types in masseter muscle of adults with different states of occlusion	Ringqvist, M.	1974	Does not include male and female data
Impaired Muscle Mitochondrial Biogenesis and Myogenesis in Spinal Muscular Atrophy	Ripolone, M.; Ronchi, D.; Violano, R.; Vallejo, D.; Fagiolari, G.; Barca, E.; Lucchini, V.; Colombo, I.; Villa, L.; Berardinelli, A.; Balottin, U.; Morandi, L.; Mora, M.; Bordoni, A.; Fortunato, F.; Corti, S.; Parisi,	2015	Did not perform fiber type analysis

	D.; Toscano, A.; Sciacco, M.; DiMauro, S.; Comi, G. P.; Moggio, M.		
Calcium currents in normal and dystrophic human skeletal muscle cells in culture	Rivet, M.; Cognard, C.; Rideau, Y.; Duport, G.; Raymond, G.	1990	Did not perform fiber type analysis
Strong iron demand during hypoxia-induced erythropoiesis is associated with down-regulation of iron-related proteins and myoglobin in human skeletal muscle	Robach, P.; Cairo, G.; Gelfi, C.; Bernuzzi, F.; Pilegaard, H.; ViganÃ², A.; Santambrogio, P.; Cerretelli, P.; Calbet, J. A.; Moutereau, S.; Lundby, C.	2007	Does not include males and females
Alterations of systemic and muscle iron metabolism in human subjects treated with low-dose recombinant erythropoietin	Robach, P.; Recalcati, S.; Girelli, D.; Gelfi, C.; Aachmann-Andersen, N. J.; Thomsen, J. J.; Norgaard, A. M.; Alberghini, A.; Campostrini, N.; Castagna, A.; ViganÃ², A.; Santambrogio, P.; Kempf, T.; Wollert, K. C.; Moutereau, S.; Lundby, C.; Cairo, G.	2009	Does not include males and females
Skeletal muscle LINE-1 ORF1 mRNA is higher in older humans but decreases with endurance exercise and is negatively associated with higher physical activity	Roberson, P. A.; Romero, M. A.; Osburn, S. C.; Mumford, P. W.; Vann, C. G.; Fox, C. D.; McCullough, D. J.; Brown, M. D.; Roberts, M. D.	2019	Did not perform fiber type analysis
Electrophoretic separation of myosin heavy chain isoforms using a modified mini gel system	Roberts, M. D.; Dalbo, V. J.; Sunderland, K. L.; Kerksick, C. M.	2012	Does not include males and females
Enhanced metabolic cycling in subjects after colonic resection for ulcerative colitis	Robertson, M. D.; Bickerton, A. S.; Dennis, A. L.; Vidal, H.; Jewell, D. P.; Frayn, K. N.	2005	Did not perform fiber type analysis
Lipoprotein lipase activity in surgical patients: influence of trauma and infection	Robin, A. P.; Askanazi, J.; Greenwood, M. R.; Carpentier, Y. A.; Gump, F. E.; Kinney, J. M.	1981	Did not perform fiber type analysis
High insulin combined with essential amino acids stimulates skeletal muscle mitochondrial protein synthesis while decreasing insulin sensitivity in healthy humans	Robinson, M. M.; Soop, M.; Sohn, T. S.; Morse, D. M.; Schimke, J. M.; Klaus, K. A.; Nair, K. S.	2014	Did not perform fiber type analysis
Role of submaximal exercise in promoting creatine and glycogen accumulation in human skeletal muscle	Robinson, T. M.; Sewell, D. A.; Hultman, E.; Greenhaff, P. L.	1999	Does not include males and females
Unmasking potential intracellular roles for dysferlin through improved immunolabeling methods	Roche, J. A.; Ru, L. W.; O'Neill, A. M.; Resneck, W. G.; Lovering, R. M.; Bloch, R. J.	2011	Autopsies
Effects of muscle glycogen on performance of repeated sprints and mechanisms of fatigue	Rockwell, M. S.; Rankin, J. W.; Dixon, H.	2003	Does not include males and females
Histochemical and morphometrical ageing changes in human vocal cord muscles	RodeÃ±o, M. T.; SÃ¡nchez-FernÃ¡ndez, J. M.; Rivera-Pomar, J. M.	1993	No healthy subjects or controls
Effects of intermittent hypoxic training on amino and fatty acid oxidative combustion in human permeabilized muscle fibers	Roels, B.; Thomas, C.; Bentley, D. J.; Mercier, J.; Hayot, M.; Millet, G.	2007	Does not include males and females
Decreased Aerobic Exercise Capacity After Long-Term Remission From Cushing Syndrome: Exploration of Mechanisms	Roerink, Shpp; Cocks, M. S.; Wagenmakers, Maem; Rodighiero, R. P.; Strauss, J. A.; Shepherd, S. O.; Plantinga, T. S.; Thijssen, D. H. J.; Hopman, M. T. E.; Pereira, A. M.; Smit,	2020	Does not include male and female data

	J. W.; Wagenmakers, A. J. M.; Netea-Maier, R. T.; Hermus, Armm		
Skeletal muscle mitochondrial function and exercise capacity in HIV-infected patients with lipodystrophy and elevated p-lactate levels	RÃ¸ge, B. T.; Calbet, J. A.; MÃ¸ller, K.; Ullum, H.; Hendel, H. W.; Gerstoft, J.; Pedersen, B. K.	2002	Did not perform fiber type analysis
Changes in skeletal muscle with aging: effects of exercise training	Rogers, M. A.; Evans, W. J.	1993	Review (ex: systematic review/meta-analysis)
Altered gene and protein expressions of vitamin D receptor in skeletal muscle in sarcopenic patients who sustained distal radius fractures	Roh, Y. H.; Hong, S. W.; Chung, S. W.; Lee, Y. S.	2019	Did not perform fiber type analysis
Amyloid beta peptides in human plasma and tissues and their significance for Alzheimer's disease	Roher, A. E.; Esh, C. L.; Kokjohn, T. A.; CastaÃ±o, E. M.; Van Vickle, G. D.; Kalback, W. M.; Patton, R. L.; Luehrs, D. C.; Daugs, I. D.; Kuo, Y. M.; Emmerling, M. R.; Soares, H.; Quinn, J. F.; Kaye, J.; Connor, D. J.; Silverberg, N. B.; Adler, C. H.; Seward, J. D.; Beach, T. G.; Sabbagh, M. N.	2009	Did not perform fiber type analysis
Progressive exercise training improves maximal aerobic capacity in individuals with well-healed burn injuries	Romero, S. A.; Moralez, G.; Jaffery, M. F.; Huang, M.; Cramer, M. N.; Romain, N.; Kouda, K.; Haller, R. G.; Crandall, C. G.	2019	Did not perform fiber type analysis
Distinct inflammatory gene expression in extraocular muscle and fat from patients with Graves' orbitopathy	Romero-Kusabara, I. L.; Filho, J. V.; Scalissi, N. M.; Melo, K. C.; Demartino, G.; Longui, C. A.; Melo, M. R.; Cury, A. N.	2017	Did not perform fiber type analysis
Age influences DNA methylation and gene expression of COX7A1 in human skeletal muscle	RÃ¶nn, T.; Poulsen, P.; Hansson, O.; Holmkvist, J.; Almgren, P.; Nilsson, P.; Tuomi, T.; Isomaa, B.; Groop, L.; Vaag, A.; Ling, C.	2008	Did not perform fiber type analysis
Disturbed energy balance in skeletal muscle of patients with untreated primary hypertension	Ronquist, G.; Soussi, B.; Frithz, G.; ScherstÃ©n, T.; WaldenstrÃ¶m, A.	1995	Does not include males and females
Proteomic Profiling Unravels a Key Role of Specific Macrophage Subtypes in Sporadic Inclusion Body Myositis	Roos, A.; Preusse, C.; Hathazi, D.; Goebel, H. H.; Stenzel, W.	2019	No healthy subjects or controls
Histopathological changes in skeletal muscle associated with chronic ischaemia	Roos, S.; Fyhr, I. M.; Sunnerhagen, K. S.; Moslemi, A. R.; Oldfors, A.; Ullman, M.	2016	Did not perform fiber type analysis
Muscle metabolism and atrophy: let's talk about sex	Rosa-Caldwell, M. E.; Greene, N. P.	2019	Review (ex: systematic review/meta-analysis)
Effect of eccentric exercise velocity on akt/mtor/p70(s6k) signaling in human skeletal muscle	Roschel, H.; Ugrinowistch, C.; Barroso, R.; Batista, M. A.; Souza, E. O.; Aoki, M. S.; Siqueira-Filho, M. A.; Zanuto, R.; Carvalho, C. R.; Neves, M.; Mello, M. T.; Tricoli, V.	2011	Does not include males and females
Triiodothyronine and leptin repletion in humans similarly reverse weight-loss-induced changes in skeletal muscle	Rosenbaum, M.; Goldsmith, R. L.; Haddad, F.; Baldwin, K. M.; Smiley, R.; Gallagher, D.; Leibel, R. L.	2018	Did not perform fiber type analysis
Skeletal Muscle mRNA Response to Hypobaric and Normobaric Hypoxia After Normoxic Endurance Exercise	Ross, C. I.; Shute, R. J.; Ruby, B. C.; Slivka, D. R.	2019	Did not perform fiber type analysis

Motor neuron branching patterns in psychotic patients	Ross-Stanton, J.; Meltzer, H. Y.	1981	Did not perform fiber type analysis
Quantitation of glycolysis and skeletal muscle glycogen synthesis in humans	Rossetti, L.; Lee, Y. T.; Ruiz, J.; Aldridge, S. C.; Shamoon, H.; Boden, G.	1993	Did not perform fiber type analysis
A test to evaluate the physical impact on technical performance in soccer	Rostgaard, T.; Iaia, F. M.; Simonsen, D. S.; Bangsbo, J.	2008	Does not include males and females
Cellular infiltrates in human skeletal muscle: exercise induced damage as a model for inflammatory muscle disease?	Round, J. M.; Jones, D. A.; Cambridge, G.	1987	Did not perform fiber type analysis
A flexible microprocessor system for the measurement of cell size	Round, J. M.; Jones, D. A.; Edwards, R. H.	1982	No healthy subjects or controls
Characterization of polymyositis infiltrates using monoclonal antibodies to human leucocyte antigens	Rowe, D. J.; Isenberg, D. A.; McDougall, J.; Beverley, P. C.	1981	Does not report sex of subjects
Transcriptome and translational signaling following endurance exercise in trained skeletal muscle: impact of dietary protein	Rowlands, D. S.; Thomson, J. S.; Timmons, B. W.; Raymond, F.; Fuerholz, A.; Mansourian, R.; Zwahlen, M. C.; MÃ©tairon, S.; Glover, E.; Stellingwerff, T.; Kussmann, M.; Tarnopolsky, M. A.	2011	Does not include males and females
Single-cell transcriptional profiles in human skeletal muscle	Rubenstein, A. B.; Smith, G. R.; Raue, U.; Begue, G.; Minchev, K.; Ruf-Zamojski, F.; Nair, V. D.; Wang, X.; Zhou, L.; Zaslavsky, E.; Trappe, T. A.; Trappe, S.; Sealfon, S. C.	2020	No cross-sectional area data
Morphological Differences in the Inferior Oblique Muscles from Subjects with Over-elevation in Adduction	Rudell, J. C.; Stager, D., Jr.; Felius, J.; McLoon, L. K.	2020	Does not report sex of subjects
Total body potassium, skeletal muscle potassium and magnesium in patients with Bartter's syndrome	Rudin, A.; Bosaeus, I.; Hessov, I.	1990	No healthy subjects or controls
Differential expression of nitric oxide synthases (NOS 1-3) in human skeletal muscle following exercise countermeasure during 12 weeks of bed rest	Rudnick, J.; PÃ¼ttmann, B.; Tesch, P. A.; Alkner, B.; Schoser, B. G.; Salanova, M.; Kirsch, K.; Gunga, H. C.; Schiffl, G.; LÃ¼ck, G.; Blottner, D.	2004	Does not include males and females
Sodium channel slow inactivation and the distribution of sodium channels on skeletal muscle fibres enable the performance properties of different skeletal muscle fibre types	Ruff, R. L.	1996	Does not report sex of subjects
Sodium channel regulation of skeletal muscle membrane excitability	Ruff, R. L.	1997	Does not report sex of subjects
Na+ current densities and voltage dependence in human intercostal muscle fibres	Ruff, R. L.; Whittlesey, D.	1992	Does not include males and females
Tissue-specific regulation of lipoprotein lipase in humans: effects of fasting	Ruge, T.; Svensson, M.; Eriksson, J. W.; Olivecrona, G.	2005	Did not perform fiber type analysis
Modifications of skeletal muscle ryanodine receptor type 1 and exercise intolerance in heart failure	Rullman, E.; Andersson, D. C.; Melin, M.; Reiken, S.; Mancini, D. M.; Marks, A. R.; Lund, L. H.; Gustafsson, T.	2013	Did not perform fiber type analysis

Endurance exercise activates matrix metalloproteinases in human skeletal muscle	Rullman, E.; Norrbom, J.; StrÃ¶mberg, A.; WÃ¥gsÃ¤ter, D.; Rundqvist, H.; Haas, T.; Gustafsson, T.	2009	Does not include males and females
Is skeletal muscle oxidative capacity decreased in old age?	Russ, D. W.; Kent-Braun, J. A.	2004	Review (ex: systematic review/meta-analysis)
COPD results in a reduction in UCP3 long mRNA and UCP3 protein content in types I and IIa skeletal muscle fibers	Russell, A. P.; Somm, E.; DebigarÃ©, R.; Hartley, O.; Richard, D.; Gastaldi, G.; Melotti, A.; Michaud, A.; Giacobino, J. P.; Muzzin, P.; LeBlanc, P.; Maltais, F.	2004	No healthy subjects or controls
UCP3 protein regulation in human skeletal muscle fibre types I, IIa and IIx is dependent on exercise intensity	Russell, A. P.; Somm, E.; Praz, M.; Crettenand, A.; Hartley, O.; Melotti, A.; Giacobino, J. P.; Muzzin, P.; Gobelet, C.; DÃ©riaz, O.	2003	Does not include males and females
UCP3 protein expression is lower in type I, IIa and IIx muscle fiber types of endurance-trained compared to untrained subjects	Russell, A. P.; Wadley, G.; Hesselink, M. K.; Schaart, G.; Lo, S.; LÃ©ger, B.; Garnham, A.; Kornips, E.; Cameron-Smith, D.; Giacobino, J. P.; Muzzin, P.; Snow, R.; Schrauwen, P.	2003	Does not include males and females
Striated muscle activator of Rho signalling (STARS) is reduced in ageing human skeletal muscle and targeted by miR-628-5p	Russell, A. P.; Wallace, M. A.; Kalanon, M.; Zacharewicz, E.; Della Gatta, P. A.; Garnham, A.; Lamon, S.	2017	Does not include males and females
Changes in skeletal muscle after discontinuation of growth hormone treatment in young adults with hypopituitarism	Rutherford, O. M.; Jones, D. A.; Round, J. M.; Preece, M. A.	1989	Only in children (0-17 years)
Assessment of in vivo skeletal muscle mitochondrial respiratory capacity in humans by near-infrared spectroscopy: a comparison with in situ measurements	Ryan, T. E.; Brophy, P.; Lin, C. T.; Hickner, R. C.; Neufer, P. D.	2014	Did not perform fiber type analysis
Interventional- and amputation-stage muscle proteomes in the chronically threatened ischemic limb	Ryan, T. E.; Kim, K.; Scali, S. T.; Berceli, S. A.; Thome, T.; Salyers, Z. R.; O'Malley, K. A.; Green, T. D.; Karnekar, R.; Fisher-Wellman, K. H.; Yamaguchi, D. J.; McClung, J. M.	2022	Did not perform fiber type analysis
Extensive skeletal muscle cell mitochondriopathy distinguishes critical limb ischemia patients from claudicants	Ryan, T. E.; Yamaguchi, D. J.; Schmidt, C. A.; Zeczycki, T. N.; Shaikh, S. R.; Brophy, P.; Green, T. D.; Tarpey, M. D.; Karnekar, R.; Goldberg, E. J.; Sparagna, G. C.; Torres, M. J.; Annex, B. H.; Neufer, P. D.; Spangenburg, E. E.; McClung, J. M.	2018	Does not include male and female data
Effect of endurance versus resistance training on local muscle and systemic inflammation and oxidative stress in COPD	RyrsÃ¸, C. K.; Thaning, P.; Siebenmann, C.; Lundby, C.; Lange, P.; Pedersen, B. K.; Hellsten, Y.; Iepsen, U. W.	2018	Did not perform fiber type analysis
Immunohistochemical analysis of human skeletal muscle AMP deaminase deficiency. Evidence of a correlation between the muscle HPRG content and the level of the residual AMP deaminase activity	Sabbatini, A. R.; Toscano, A.; Aguennouz, M.; Martini, D.; Polizzi, E.; Ranieri-Raggi, M.; Moir, A. J.; Migliorato, A.; Musumeci, O.; Vita, G.; Raggi, A.	2006	No healthy subjects or controls
Myoadenylate deaminase deficiency. Functional and metabolic abnormalities associated with disruption of the purine nucleotide cycle	Sabina, R. L.; Swain, J. L.; Olanow, C. W.; Bradley, W. G.; Fishbein, W. N.; DiMauro, S.; Holmes, E. W.	1984	Did not perform muscle biopsy

Mechanisms underlying Andersen's syndrome pathology in skeletal muscle are revealed in human myotubes	Sacconi, S.; Simkin, D.; Arrighi, N.; Chapon, F.; Larroque, M. M.; Vicart, S.; Sternberg, D.; Fontaine, B.; Barhanin, J.; Desnuelle, C.; Bendahhou, S.	2009	Did not perform fiber type analysis
Gene expression profiling in limb-girdle muscular dystrophy 2A	SÃ¡enz, A.; Azpitarte, M.; ArmaÃ±anzas, R.; Leturcq, F.; Alzualde, A.; Inza, I.; GarcÃa-Bragado, F.; De la Herran, G.; Corcuera, J.; Cabello, A.; Navarro, C.; De la Torre, C.; Gallardo, E.; Illa, I.; LÃ³pez de Munain, A.	2008	Did not perform fiber type analysis
NADH and NADPH in human skeletal muscle at rest and during ischaemia	Sahlin, K.	1983	Does not include males and females
The potential for mitochondrial fat oxidation in human skeletal muscle influences whole body fat oxidation during low-intensity exercise	Sahlin, K.; Mogensen, M.; Bagger, M.; FernstrÃ¶m, M.; Pedersen, P. K.	2007	Does not include males and females
Phosphocreatine content in single fibers of human muscle after sustained submaximal exercise	Sahlin, K.; SÃ¶derlund, K.; Tonkonogi, M.; Hirakoba, K.	1997	Does not include males and females
Prior heavy exercise eliminates VO2 slow component and reduces efficiency during submaximal exercise in humans	Sahlin, K.; SÃ¸rensen, J. B.; Gladden, L. B.; Rossiter, H. B.; Pedersen, P. K.	2005	Does not include males and females
Dysregulated Genes, MicroRNAs, Biological Pathways, and Gastrocnemius Muscle Fiber Types Associated With Progression of Peripheral Artery Disease: A Preliminary Analysis	Saini, S. K.; PÃ©rez-Cremades, D.; Cheng, H. S.; Kosmac, K.; Peterson, C. A.; Li, L.; Tian, L.; Dong, G.; Wu, K. K.; Bouverat, B.; Wohlgemuth, S. E.; Ryan, T.; Sufit, R. L.; Ferrucci, L.; McDermott, M. M.; Leeuwenburgh, C.; Feinberg, M. W.	2022	Does not include male and female data
Exercise regulates Akt and glycogen synthase kinase-3 activities in human skeletal muscle	Sakamoto, K.; Arnolds, D. E.; Ekberg, I.; Thorell, A.; Goodyear, L. J.	2004	Did not perform fiber type analysis
Interferon-stimulated gene 15 (ISG15) conjugates proteins in dermatomyositis muscle with perifascicular atrophy	Salajegheh, M.; Kong, S. W.; Pinkus, J. L.; Walsh, R. J.; Liao, A.; Nazareno, R.; Amato, A. A.; Krastins, B.; Morehouse, C.; Higgs, B. W.; Jallal, B.; Yao, Y.; Sarracino, D. A.; Parker, K. C.; Greenberg, S. A.	2010	Does not report sex of subjects
Nature of "Tau" immunoreactivity in normal myonuclei and inclusion body myositis	Salajegheh, M.; Pinkus, J. L.; Nazareno, R.; Amato, A. A.; Parker, K. C.; Greenberg, S. A.	2009	Does not report sex of subjects
Upregulation of thrombospondin-1(TSP-1) and its binding partners, CD36 and CD47, in sporadic inclusion body myositis	Salajegheh, M.; Raju, R.; Schmidt, J.; Dalakas, M. C.	2007	No healthy subjects or controls
Ryanodine receptor type-1 (RyR1) expression and protein S-nitrosylation pattern in human soleus myofibres following bed rest and exercise countermeasure	Salanova, M.; Schiffl, G.; Rittweger, J.; Felsenberg, D.; Blottner, D.	2008	Does not include males and females
Phosphomannosyl receptors of lysosomal enzymes of skeletal muscle in neuromuscular diseases	Salminen, A.; MarjomÃ¤ki, V.; Tolonen, U.; MyllylÃ¤, V. V.	1988	Did not perform fiber type analysis
Contribution of muscle and liver to glucose-fatty acid cycle in humans	Saloranta, C.; Koivisto, V.; WidÃ©n, E.; Falholt, K.; DeFronzo, R. A.; HÃ¤rkÃ¶nen, M.; Groop, L.	1993	Does not include males and females

Morphology, enzyme activities and buffer capacity in leg muscles of Kenyan and Scandinavian runners	Saltin, B.; Kim, C. K.; Terrados, N.; Larsen, H.; Svedenhag, J.; Rolf, C. J.	1995	Does not include male and female data
Skeletal muscle oxidative function in vivo and ex vivo in athletes with marked hypertrophy from resistance training	Salvadego, D.; Domenis, R.; Lazzer, S.; Porcelli, S.; Rittweger, J.; Rizzo, G.; Mavelli, I.; Simunic, B.; Pisot, R.; Grassi, B.	2013	Does not include males and females
Resistance Exercise-induced Regulation of Muscle Protein Synthesis to Intraset Rest	Salvador, A. F.; Askow, A. T.; McKenna, C. F.; Fang, H. Y.; Burke, S. K.; Li, Z.; Ulanov, A. V.; Paluska, S. A.; Petruzzello, S. J.; Boppart, M. D.; Oliver, J. M.; Burd, N. A.	2020	Did not perform fiber type analysis
Myofibrillar-protein isoforms and sarcoplasmic-reticulum Ca2+-transport activity of single human muscle fibres	Salviati, G.; Betto, R.; Danieli Betto, D.; Zeviani, M.	1984	Does not include male and female data
Adiposity and immune-muscle crosstalk in South Asians &Europeans: A cross-sectional study	Samaan, M. C.; Anand, S. S.; Sharma, A. M.; Bonner, A.; Beyene, J.; Samjoo, I.; Tarnopolsky, M. A.	2015	Does not include male and female data
Deficient muscle regeneration potential in sarcopenic COPD patients: Role of satellite cells	Sancho-MuÃ±oz, A.; Guitart, M.; RodrÃ-guez, D. A.; Gea, J.; MartÃnez-Llorens, J.; Barreiro, E.	2021	Does not include male and female data
The human tongue slows down to speak: muscle fibers of the human tongue	Sanders, I.; Mu, L.; Amirali, A.; Su, H.; Sobotka, S.	2013	Autopsies
Scalene muscle abnormalities in traumatic thoracic outlet syndrome	Sanders, R. J.; Jackson, C. G.; Banchero, N.; Pearce, W. H.	1990	No healthy subjects or controls
Muscle Na-K-pump and fatigue responses to progressive exercise in normoxia and hypoxia	Sandiford, S. D.; Green, H. J.; Duhamel, T. A.; Schertzer, J. D.; Perco, J. D.; Ouyang, J.	2005	Does not include males and females
Representativeness of a muscle biopsy specimen for the whole muscle	Sandstedt, P. E.	1981	No cross-sectional area data
Myosin heavy chain isoform expression and high energy phosphate content in human muscle fibres at rest and post-exercise	Sant'Ana Pereira, J. A.; Sargeant, A. J.; Rademaker, A. C.; de Haan, A.; van Mechelen, W.	1996	Does not include males and females
Analysis of MTMR1 expression and correlation with muscle pathological features in juvenile/adult onset myotonic dystrophy type 1 (DM1) and in myotonic dystrophy type 2 (DM2)	Santoro, M.; Modoni, A.; Masciullo, M.; Gidaro, T.; Broccolini, A.; Ricci, E.; Tonali, P. A.; Silvestri, G.	2010	Does not include male and female data
Greater performance impairment of black runners than white runners when running in hypoxia	Santos-Concejero, J.; Tucker, R.; Myburgh, K. H.; Essen-Gustavsson, B.; Kohn, T. A.	2014	Does not include males and females
Dietary palmitate and oleate differently modulate insulin sensitivity in human skeletal muscle	Sarabhai, T.; Koliaki, C.; Mastrototaro, L.; Kahl, S.; Pesta, D.; Apostolopoulou, M.; Wolkersdorfer, M.; BÃ¶nner, A. C.; Bobrov, P.; Markgraf, D. F.; Herder, C.; Roden, M.	2022	Did not perform fiber type analysis
Functional and structural changes after disuse of human muscle	Sargeant, A. J.; Davies, C. T.; Edwards, R. H.; Maunder, C.; Young, A.	1977	Does not include males and females
Effect of cocaine on the contracture response to 1% halothane in patients undergoing diagnostic muscle biopsy for malignant hyperthermia	Sato, N.; Brum, J. M.; Mitsumoto, H.; DeBoer, G. E.	1995	No healthy subjects or controls

Fibre types in skeletal muscles of chronic obstructive pulmonary disease patients related to respiratory function and exercise tolerance	Satta, A.; Migliori, G. B.; Spanevello, A.; Neri, M.; Bottinelli, R.; Canepari, M.; Pellegrino, M. A.; Reggiani, C.	1997	Does not include males and females
Cytochrome oxidase activity and mitochondrial gene expression in skeletal muscle of patients with chronic obstructive pulmonary disease	Sauleda, J.; GarcÃa-Palmer, F.; Wiesner, R. J.; Tarraga, S.; Harting, I.; TomÃ¡s, P.; GÃ³mez, C.; Saus, C.; Palou, A.; AgustÃ, A. G.	1998	Does not include males and females
Observational study on the occurrence of muscle spindles in human digastric and mylohyoideus muscles	Saverino, D.; De Santanna, A.; Simone, R.; Cervioni, S.; Cattrysse, E.; Testa, M.	2014	Autopsies David Wrucke
Altered oxidative stress and antioxidant defence in skeletal muscle during the first year following spinal cord injury	Savikj, M.; Kostovski, E.; Lundell, L. S.; Iversen, P. O.; Massart, J.; Widegren, U.	2019	Does not include males and females
Retained differentiation capacity of human skeletal muscle satellite cells from spinal cord-injured individuals	Savikj, M.; Ruby, M. A.; Kostovski, E.; Iversen, P. O.; Zierath, J. R.; Krook, A.; Widegren, U.	2018	Does not include males and females
Chronic primary hyperinsulinaemia is associated with altered insulin receptor mRNA splicing in muscle of patients with insulinoma	Sbraccia, P.; D'Adamo, M.; Leonetti, F.; Caiola, S.; Iozzo, P.; Giaccari, A.; Buongiorno, A.; Tamburrano, G.	1996	Did not perform fiber type analysis
CAV3 mutations causing exercise intolerance, myalgia and rhabdomyolysis: Expanding the phenotypic spectrum of caveolinopathies	Scalco, R. S.; Gardiner, A. R.; Pitceathly, R. D.; Hilton-Jones, D.; Schapira, A. H.; Turner, C.; Parton, M.; Desikan, M.; Barresi, R.; Marsh, J.; Manzur, A. Y.; Childs, A. M.; Feng, L.; Murphy, E.; Lamont, P. J.; Ravenscroft, G.; Wallefeld, W.; Davis, M. R.; Laing, N. G.; Holton, J. L.; Fialho, D.; Bushby, K.; Hanna, M. G.; Phadke, R.; Jungbluth, H.; Houlden, H.; Quinlivan, R.	2016	No healthy subjects or controls
Skeletal muscle satellite cells in amyotrophic lateral sclerosis	Scaramozza, A.; Marchese, V.; Papa, V.; Salaroli, R.; SorarÃ¹, G.; Angelini, C.; Cenacchi, G.	2014	Did not perform fiber type analysis
Studies of fragmented sarcoplasmic reticulum from human skeletal muscle	Scarpa, A.; DiMauro, S.; Bonilla, E.; Schotland, D. L.	1978	Does not include males and females
Morphometric analysis of skeletal muscle fibres and capillaries in mitochondrial myopathies	Scelsi, R.	1992	Does not report sex of subjects
Histochemical and ultrastructural aspects of m. vastus lateralis in sedentary old people (age 65--89 years)	Scelsi, R.; Marchetti, C.; Poggi, P.	1980	Does not include male and female data
Lack of robust satellite cell activation and muscle regeneration during the progression of Pompe disease	Schaaf, G. J.; van Gestel, T. J.; Brusse, E.; Verdijk, R. M.; de Coo, I. F.; van Doorn, P. A.; van der Ploeg, A. T.; Pijnappel, W. W.	2015	Does not report sex of subjects
Effect of insulin on GLUT-4 mRNA and protein concentrations in skeletal muscle of patients with NIDDM and their first-degree relatives	Schalin-JÃ¤ntti, C.; Yki-JÃ¤rvinen, H.; Koranyi, L.; Bourey, R.; LindstrÃ¶m, J.; Nikula-IjÃ¤s, P.; Franssila-Kallunki, A.; Groop, L. C.	1994	Did not perform fiber type analysis
The relationship between the mean muscle fibre area and the muscle cross-sectional area of the thigh in subjects with large differences in thigh girth	Schantz, P.; Fox, E. R.; Norgren, P.; TydÃ©n, A.	1981	No cross-sectional area data

Increases in myofibrillar ATPase intermediate human skeletal muscle fibers in response to endurance training	Schantz, P.; Henriksson, J.	1983	Does not include male and female data
Skeletal muscle of trained and untrained paraplegics and tetraplegics	Schantz, P.; SjÃ¶berg, B.; Widebeck, A. M.; Ekblom, B.	1997	Does not include males and females
Coexistence of slow and fast isoforms of contractile and regulatory proteins in human skeletal muscle fibres induced by endurance training	Schantz, P. G.; Dhoot, G. K.	1987	Does not include male and female data
Enzyme levels of the NADH shuttle systems: measurements in isolated muscle fibres from humans of differing physical activity	Schantz, P. G.; Henriksson, J.	1987	Does not include male and female data
Malate-aspartate and alpha-glycerophosphate shuttle enzyme levels in human skeletal muscle: methodological considerations and effect of endurance training	Schantz, P. G.; SjÃ¶berg, B.; Svedenhag, J.	1986	Does not include male and female data
Morphological Characteristics of Idiopathic Inflammatory Myopathies in Juvenile Patients	SchÃ¤nzer, A.; Rager, L.; Dahlhaus, I.; Dittmayer, C.; Preusse, C.; Della Marina, A.; Goebel, H. H.; Hahn, A.; Stenzel, W.	2021	Only in children (0-17 years)
Skeletal muscle alterations in patients with chronic heart failure	Schaufelberger, M.; Eriksson, B. O.; Grimby, G.; Held, P.; Swedberg, K.	1997	Does not include male and female data
Skeletal muscle metabolism during exercise in patients with chronic heart failure	Schaufelberger, M.; Eriksson, B. O.; Held, P.; Swedberg, K.	1996	Did not perform fiber type analysis
Skeletal muscle characteristics, muscle strength and thigh muscle area in patients before and after cardiac transplantation	Schaufelberger, M.; Eriksson, B. O.; LÃ¶nn, L.; Rundqvist, B.; Sunnerhagen, K. S.; Swedberg, K.	2001	Does not include male and female data
Metallothionein-mediated antioxidant defense system and its response to exercise training are impaired in human type 2 diabetes	Scheede-Bergdahl, C.; Penkowa, M.; Hidalgo, J.; Olsen, D. B.; Schjerling, P.; Prats, C.; Boushel, R.; Dela, F.	2005	Full text not available
Fiber type characterization in skeletal muscle by diffusion tensor imaging	Scheel, M.; von Roth, P.; Winkler, T.; Arampatzis, A.; Prokscha, T.; Hamm, B.; Diederichs, G.	2013	Does not include males and females
Satellite cells derived from obese humans with type 2 diabetes and differentiated into myocytes in vitro exhibit abnormal response to IL-6	Scheele, C.; Nielsen, S.; Kelly, M.; Broholm, C.; Nielsen, A. R.; Taudorf, S.; Pedersen, M.; Fischer, C. P.; Pedersen, B. K.	2012	Did not perform fiber type analysis
Acute exercise increases triglyceride synthesis in skeletal muscle and prevents fatty acid-induced insulin resistance	Schenk, S.; Horowitz, J. F.	2007	Does not include males and females
Decreased skeletal muscle mitochondrial DNA in patients treated with high-dose simvastatin	Schick, B. A.; Laaksonen, R.; Frohlich, J. J.; PÃ¤ivÃ¤, H.; LehtimÃ¤ki, T.; Humphries, K. H.; CÃ´tÃ©, H. C.	2007	Did not perform fiber type analysis
Basal and exercise induced label-free quantitative protein profiling of m. vastus lateralis in trained and untrained individuals	Schild, M.; Ruhs, A.; Beiter, T.; ZÃ¼gel, M.; Hudemann, J.; Reimer, A.; Krumholz-Wagner, I.; Wagner, C.; Keller, J.; Eder, K.; KrÃ¼ger, K.; KrÃ¼ger, M.; Braun, T.; NieÃŸ, A.; Steinacker, J.; Mooren, F. C.	2015	Does not include males and females
Microanalysis of cardiolipin in small biopsies including skeletal muscle from patients with mitochondrial disease	Schlame, M.; Shanske, S.; Doty, S.; KÃ¶nig, T.; Sculco, T.; DiMauro, S.; Blanck, T. J.	1999	Did not perform fiber type analysis

Disorganization of glycolytic and gluconeogenic pathways in skeletal muscle of aged persons studied by histometric and enzymatic methods	Schlenska, G. K.; Kleine, T. O.	1980	Does not include male and female data
Contracture knots in normal and diseased muscle fibres	Schmalbruch, H.	1973	Autopsies
Endurance training modulates the muscular transcriptome response to acute exercise	Schmutz, S.; DÃ¤pp, C.; Wittwer, M.; Vogt, M.; Hoppeler, H.; FlÃ¼ck, M.	2006	Does not include males and females
Soluble guanylyl cyclase is localized at the neuromuscular junction in human skeletal muscle	Schoser, B. G.; Behrends, S.	2001	Does not report sex of subjects
Tenascin in denervated human muscle	Schoser, B. G.; Goebel, H. H.	1996	No healthy subjects or controls
Cell death and apoptosis-related proteins in muscle biopsies of sporadic amyotrophic lateral sclerosis and polyneuropathy	Schoser, B. G.; Wehling, S.; Blottner, D.	2001	Did not perform fiber type analysis
Intramyocellular lipid content and molecular adaptations in response to a 1-week high-fat diet	Schrauwen-Hinderling, V. B.; Kooi, M. E.; Hesselink, M. K.; Moonen-Kornips, E.; Schaart, G.; Mustard, K. J.; Hardie, D. G.; Saris, W. H.; Nicolay, K.; Schrauwen, P.	2005	Does not include males and females
Eosinophils in hereditary and inflammatory myopathies	SchrÃ¶der, T.; Fuchss, J.; Schneider, I.; Stoltenburg-Didinger, G.; Hanisch, F.	2013	Did not perform fiber type analysis
Triple immunofluorescence confocal laser scanning microscopy: spatial correlation of novel cellular differentiation markers in human muscle biopsies	Schubert, W.	1991	No healthy subjects or controls
Effects of resistance training on the rate of muscle protein synthesis in frail elderly people	Schulte, J. N.; Yarasheski, K. E.	2001	Did not perform muscle biopsy
Enzyme histochemical and histographic data on normal human facial muscles	Schwarting, S.; SchrÃ¶der, M.; Stennert, E.; Goebel, H. H.	1982	No healthy subjects or controls
Morphology of denervated human facial muscles	Schwarting, S.; SchrÃ¶der, M.; Stennert, E.; Goebel, H. H.	1984	No healthy subjects or controls
Unloaded shortening velocity and myosin heavy chain variations in human laryngeal muscle fibers	Sciote, J. J.; Morris, T. J.; Brandon, C. A.; Horton, M. J.; Rosen, C.	2002	Does not include male and female data
Fibre type classification and myosin isoforms in the human masseter muscle	Sciote, J. J.; Rowlerson, A. M.; Hopper, C.; Hunt, N. P.	1994	Does not include male and female data
The cross sectional area of diaphragmatic muscle fibres in emphysema, measured by an automated image analysis system	Scott, K. W.; Hoy, J.	1976	Autopsies
The genetic regulatory signature of type 2 diabetes in human skeletal muscle	Scott, L. J.; Erdos, M. R.; Huyghe, J. R.; Welch, R. P.; Beck, A. T.; Wolford, B. N.; Chines, P. S.; Didion, J. P.; Narisu, N.; Stringham, H. M.; Taylor, D. L.; Jackson, A. U.; Vadlamudi, S.; Bonnycastle, L. L.; Kinnunen, L.; Saramies, J.; Sundvall, J.; Albanus, R. D.; Kiseleva, A.; Hensley, J.;	2016	Did not perform fiber type analysis

	Crawford, G. E.; Jiang, H.; Wen, X.; Watanabe, R. M.; Lakka, T. A.; Mohlke, K. L.; Laakso, M.; Tuomilehto, J.; Koistinen, H. A.; Boehnke, M.; Collins, F. S.; Parker, S. C.		
Home-hit improves muscle capillarisation and eNOS/NAD(P)Hoxidase protein ratio in obese individuals with elevated cardiovascular disease risk	Scott, S. N.; Shepherd, S. O.; Hopkins, N.; Dawson, E. A.; Strauss, J. A.; Wright, D. J.; Cooper, R. G.; Kumar, P.; Wagenmakers, A. J. M.; Cocks, M.	2019	Does not include male and female data
Gene expression profiling in tibial muscular dystrophy reveals unfolded protein response and altered autophagy	Screen, M.; Raheem, O.; Holmlund-Hampf, J.; Jonson, P. H.; Huovinen, S.; Hackman, P.; Udd, B.	2014	No healthy subjects or controls
Estimating relative motoneurone size in human masseter muscle	Scutter, S. D.; TÃ¼rker, K. S.	2000	Did not perform fiber type analysis
Contralateral influence on recruitment of curarized muscle fibres during maximal voluntary extension of the legs	Secher, N. H.; RÃ¸rsgaard, S.; Secher, O.	1978	Does not include males and females
In situ detection of polymerase chain reaction-amplified HIV-1 nucleic acids in skeletal muscle in patients with myopathy	Seidman, R.; Peress, N. S.; Nuovo, G. J.	1994	Did not perform fiber type analysis
Regulation of the ubiquitin proteasome system in mechanically injured human skeletal muscle	Seiffert, M.; Gosenca, D.; Ponelies, N.; Ising, N.; Patel, M. B.; Obertacke, U.; Majetschak, M.	2007	No healthy subjects or controls
Mitochondrial activity in Pompe's disease	Selak, M. A.; de Chadarevian, J. P.; Melvin, J. J.; Grover, W. D.; Salganicoff, L.; Kaye, E. M.	2000	Only in children (0-17 years)
Are MuSK antibodies the primary cause of myasthenic symptoms?	Selcen, D.; Fukuda, T.; Shen, X. M.; Engel, A. G.	2004	Did not perform fiber type analysis
Tissue-differential expression of two distinct genes for human IMP dehydrogenase (E.C.1.1.1.205)	Senda, M.; Natsumeda, Y.	1994	Does not report sex of subjects
Immunohistochemical localization of carbonic anhydrase IV in capillaries of rat and human skeletal muscle	Sender, S.; Gros, G.; Waheed, A.; Hageman, G. S.; Sly, W. S.	1994	Did not perform fiber type analysis
Tumor necrosis factor-Î± impairs adiponectin signalling, mitochondrial biogenesis, and myogenesis in primary human myotubes cultures	Sente, T.; Van Berendoncks, A. M.; Fransen, E.; Vrints, C. J.; Hoymans, V. Y.	2016	Did not perform fiber type analysis
Primary skeletal muscle myoblasts from chronic heart failure patients exhibit loss of anti-inflammatory and proliferative activity	Sente, T.; Van Berendoncks, A. M.; Jonckheere, A. I.; Rodenburg, R. J.; Lauwers, P.; Van Hoof, V.; Wouters, A.; Lardon, F.; Hoymans, V. Y.; Vrints, C. J.	2016	Did not perform fiber type analysis
Muscle fiber area distribution of musculus uvulae in obstructive sleep apnea and non-apneic snorers	SÃ©riÃ¨s, F.; Simoneau, J. A.; St Pierre, S.	2000	Does not include male and female data
Adipose tissue Î¼-crystallin is a thyroid hormone-binding protein associated with systemic insulin sensitivity	Serrano, M.; Moreno, M.; Ortega, F. J.; Xifra, G.; Ricart, W.; Moreno-Navarrete, J. M.; FernÃ¡ndez-Real, J. M.	2014	Did not perform fiber type analysis
Early effect of Roux-en-Y gastric bypass on insulin sensitivity and signaling	Severino, A.; Castagneto-Gissey, L.; Raffaelli, M.; Gastaldelli, A.; Capristo, E.;	2016	Did not perform fiber type analysis

	Iaconelli, A.; Guidone, C.; Callari, C.; Bellantone, R.; Mingrone, G.		
Skeletal muscle pathology in autosomal dominant Emery-Dreifuss muscular dystrophy with lamin A/C mutations	Sewry, C. A.; Brown, S. C.; Mercuri, E.; Bonne, G.; Feng, L.; Camici, G.; Morris, G. E.; Muntoni, F.	2001	No healthy subjects or controls
Human obesity and type 2 diabetes are associated with alterations in SREBP1 isoform expression that are reproduced ex vivo by tumor necrosis factor-alpha	Sewter, C.; Berger, D.; Considine, R. V.; Medina, G.; Rochford, J.; Ciaraldi, T.; Henry, R.; Dohm, L.; Flier, J. S.; O'Rahilly, S.; Vidal-Puig, A. J.	2002	Did not perform fiber type analysis
Effect of prehabilitation on the outcome of anterior cruciate ligament reconstruction	Shaarani, S. R.; O'Hare, C.; Quinn, A.; Moyna, N.; Moran, R.; O'Byrne, J. M.	2013	Does not include males and females
Ultrasound-guided percutaneous biopsies of the semitendinosus muscle following ACL reconstruction--a methodological description	Shalabi, A.; Eriksson, K.; Jansson, E.; Wredmark, T.	2002	Does not report sex of subjects
Human skeletal muscle biopsy procedures using the modified BergstrÃ¶m technique	Shanely, R. A.; Zwetsloot, K. A.; Triplett, N. T.; Meaney, M. P.; Farris, G. E.; Nieman, D. C.	2014	Not peer reviewed (ex: thesis, conference poster)
Effect of Chronic Hyperglycemia on Glucose Metabolism in Subjects With Normal Glucose Tolerance	Shannon, C.; Merovci, A.; Xiong, J.; Tripathy, D.; Lorenzo, F.; McClain, D.; Abdul-Ghani, M.; Norton, L.; DeFronzo, R. A.	2018	Did not perform fiber type analysis
Fiber Type-Specific Morphological and Cellular Changes of Paraspinal Muscles in Patients with Severe Adolescent Idiopathic Scoliosis	Shao, X.; Chen, J.; Yang, J.; Sui, W.; Deng, Y.; Huang, Z.; Hu, P.; Yang, J.	2020	Only in children (0-17 years)
Fasting decreases the content of D-chiroinositol in human skeletal muscle	Shashkin, P. N.; Huang, L. C.; Larner, J.; Vandenhoff, G. E.; Katz, A.	2002	Did not perform fiber type analysis
Reduction expression of thrombomodulin and endothelial cell nitric oxide synthase in dermatomyositis	Shen, G. L.; Lv, H.; Bi, H. Y.; Zhang, W.; Yao, S.; Yuan, Y.	2007	Did not perform fiber type analysis
Time-course of human muscle fibre size reduction during head-down tilt bedrest	Shenkman, B.; Belozerova, I.; Nemirovskaya, T.; Cheglova, I.; Yudaitcheva, A.; Kiseleva, E.; Mazin, M.	1998	No cross-sectional area data
Involvement of phosphoinositide 3-kinase in insulin stimulation of MAP-kinase and phosphorylation of protein kinase-B in human skeletal muscle: implications for glucose metabolism	Shepherd, P. R.; Nave, B. T.; Rincon, J.; Haigh, R. J.; Foulstone, E.; Proud, C.; Zierath, J. R.; Siddle, K.; Wallberg-Henriksson, H.	1997	Does not include males and females
Training alters the distribution of perilipin proteins in muscle following acute free fatty acid exposure	Shepherd, S. O.; Strauss, J. A.; Wang, Q.; Dube, J. J.; Goodpaster, B.; Mashek, D. G.; Chow, L. S.	2017	Does not include male and female data
Identification of human T cell leukemia/lymphoma virus type I antibodies, DNA, and protein in patients with polymyositis	Sherman, M. P.; Amin, R. M.; Rodgers-Johnson, P. E.; Morgan, O. S.; Char, G.; Mora, C. A.; Iannone, R.; Collins, G. H.; Papsidero, L.; Gibbs, C. J., Jr.; et al.,	1995	Did not perform fiber type analysis
FGF23, a novel muscle biomarker detected in the early stages of ALS	Si, Y.; Kazamel, M.; Benatar, M.; Wuu, J.; Kwon, Y.; Kwan, T.; Jiang, N.; Kentrup, D.; Faul, C.; Alesce, L.; King, P. H.	2021	Did not perform fiber type analysis

The vitamin D activator CYP27B1 is upregulated in muscle fibers in denervating disease and can track progression in amyotrophic lateral sclerosis	Si, Y.; Kazamel, M.; Kwon, Y.; Lee, I.; Anderson, T.; Zhou, S.; Bamman, M.; Wiggins, D.; Kwan, T.; King, P. H.	2020	No healthy subjects or controls
Transforming Growth Factor Beta (TGF-Î²) Is a Muscle Biomarker of Disease Progression in ALS and Correlates with Smad Expression	Si, Y.; Kim, S.; Cui, X.; Zheng, L.; Oh, S. J.; Anderson, T.; AlSharabati, M.; Kazamel, M.; Volpicelli-Daley, L.; Bamman, M. M.; Yu, S.; King, P. H.	2015	Did not perform fiber type analysis
Residual muscle cytochrome c oxidase activity accounts for submaximal exercise lactate threshold in chronic progressive external ophthalmoplegia	Siciliano, G.; Rossi, B.; Manca, L.; Angelini, C.; Tessa, A.; Vergani, L.; Martinuzzi, A.; Muratorio, A.	1996	Did not perform fiber type analysis
Segmental myofiber necrosis in myotonic dystrophy - An immunoperoxidase study of immunoglobulins in skeletal muscle	Silver, M. M.; Banerjee, D.; Hudson, A. J.	1983	No healthy subjects or controls
Establishment of long-term myogenic cultures from patients with Duchenne muscular dystrophy by retroviral transduction of a temperature-sensitive SV40 large T antigen	Simon, L. V.; Beauchamp, J. R.; O'Hare, M.; Olsen, I.	1996	Does not include males and females
Overexpression of muscle uncoupling protein 2 content in human obesity associates with reduced skeletal muscle lipid utilization	Simoneau, J. A.; Kelley, D. E.; Neverova, M.; Warden, C. H.	1998	Did not perform fiber type analysis
Human skeletal muscle fiber type alteration with high-intensity intermittent training	Simoneau, J. A.; Lortie, G.; Boulay, M. R.; Marcotte, M.; Thibault, M. C.; Bouchard, C.	1985	Does not include male and female data
Inheritance of human skeletal muscle and anaerobic capacity adaptation to high-intensity intermittent training	Simoneau, J. A.; Lortie, G.; Boulay, M. R.; Marcotte, M.; Thibault, M. C.; Bouchard, C.	1986	Does not include male and female data
Effects of two high-intensity intermittent training programs interspaced by detraining on human skeletal muscle and performance	Simoneau, J. A.; Lortie, G.; Boulay, M. R.; Marcotte, M.; Thibault, M. C.; Bouchard, C.	1987	Did not perform fiber type analysis
Repeatability of fibre type and enzyme activity measurements in human skeletal muscle	Simoneau, J. A.; Lortie, G.; Boulay, M. R.; Thibault, M. C.; Bouchard, C.	1986	Does not include male and female data
Specific force of the vastus lateralis in adults with achondroplasia	Sims, D. T.; OnambÃ©lÃ©-Pearson, G. L.; Burden, A.; Payton, C.; Morse, C. I.	2018	Did not perform muscle biopsy
Muscle carnitine in hypo- and hyperthyroidism	Sinclair, C.; Gilchrist, J. M.; Hennessey, J. V.; Kandula, M.	2005	Did not perform fiber type analysis
Ultrastructural relationship of quadriceps muscle degeneration with a distant peroneal nerve conduction in human myotonia dystrophica	Sinha, A. A.; Olson, N. D.; Nuttall, F. Q.	1987	Did not perform fiber type analysis
AMP deaminase deficiency: study of the human skeletal muscle purine metabolism during ischaemic isometric exercise	Sinkeler, S. P.; Binkhorst, R. A.; Joosten, E. M.; Wevers, R. A.; Coerwinkei, M. M.; Oei, T. L.	1987	Did not perform fiber type analysis
Simvastatin induces impairment in skeletal muscle while heart is protected	Sirvent, P.; Bordenave, S.; Vermaelen, M.; Roels, B.; Vassort, G.; Mercier, J.; Raynaud, E.; Lacampagne, A.	2005	Does not include males and females
Muscle mitochondrial metabolism and calcium signaling impairment in patients treated with statins	Sirvent, P.; Fabre, O.; Bordenave, S.; Hillaire-Buys, D.; Raynaud De Mauverger, E.; Lacampagne, A.; Mercier, J.	2012	Does not include males and females

Myofibers from Duchenne/Becker muscular dystrophy and myositis express the intermediate filament nestin	SjÃ¶berg, G.; EdstrÃ¶m, L.; Lendahl, U.; Sejersen, T.	1994	Did not perform fiber type analysis
Water and ion shifts in skeletal muscle of humans with intense dynamic knee extension	SjÃ¸gaard, G.; Adams, R. P.; Saltin, B.	1985	Does not include males and females
Magnesium and potassium status in healthy subjects as assessed by analysis of magnesium and potassium in skeletal muscle biopsies and magnesium in mononuclear cells	SjÃ¶gren, A.; FlorÃ©n, C. H.; Nilsson, A.	1987	Did not perform fiber type analysis
Magnesium, potassium and zinc deficiency in subjects with type II diabetes mellitus	SjÃ¶gren, A.; FlorÃ©n, C. H.; Nilsson, A.	1988	Did not perform fiber type analysis
Quantitative estimation of anaerobic and oxidative energy metabolism and contraction characteristics in intact human skeletal muscle in response to electrical stimulation	SjÃ¶holm, H.; Sahlin, K.; EdstrÃ¶m, L.; Hultman, E.	1983	Does not include male and female data
Intermittent claudication and muscle fiber fine structure: correlation between clinical and morphological data	SjÃ¶strÃ¶m, M.; Angquist, K. A.; Rais, O.	1980	Does not include males and females
Fine structural details of human muscle fibres after fibre type specific glycogen depletion	SjÃ¶strÃ¶m, M.; FridÃ©n, J.; Ekblom, B.	1982	Does not include males and females
Z- and M-band appearance in different histochemically defined types of human skeletal muscle fibers	SjÃ¶strÃ¶m, M.; Kidman, S.; LarsÃ©n, K. H.; Angquist, K. A.	1982	Does not include males and females
Effect of conditioned media from mature human adipocytes on insulin-stimulated Akt/PKB phosphorylation in human skeletal muscle cells: role of BMI and fat cell size	Skurk, T.; Alberti-Huber, C.; Hauner, H.	2009	Did not perform fiber type analysis
Pre- and post-synaptic abnormalities associated with impaired neuromuscular transmission in a group of patients with 'limb-girdle myasthenia'	Slater, C. R.; Fawcett, P. R.; Walls, T. J.; Lyons, P. R.; Bailey, S. J.; Beeson, D.; Young, C.; Gardner-Medwin, D.	2006	Did not perform fiber type analysis
Neuromuscular organization of the superior longitudinalis muscle in the human tongue. 1. Motor endplate morphology and muscle fiber architecture	Slaughter, K.; Li, H.; Sokoloff, A. J.	2005	Autopsies
Neuromuscular differences between volleyball players, middle distance runners and untrained controls	Sleivert, G. G.; Backus, R. D.; Wenger, H. A.	1995	Does not include males and females
The muscle oxidative regulatory response to acute exercise is not impaired in less advanced COPD despite a decreased oxidative phenotype	Slot, I. G.; van den Borst, B.; Hellwig, V. A.; Barreiro, E.; Schols, A. M.; Gosker, H. R.	2014	Does not include male and female data
Myosin heavy chain-2b transcripts and isoform are expressed in human laryngeal muscles	Smerdu, V.; Cvetko, E.	2013	Autopsies
Type IIx myosin heavy chain transcripts are expressed in type IIb fibers of human skeletal muscle	Smerdu, V.; Karsch-Mizrachi, I.; Campione, M.; Leinwand, L.; Schiaffino, S.	1994	No healthy subjects or controls
Demonstration of myosin heavy chain isoforms in rat and humans: the specificity of seven available monoclonal antibodies used in	Smerdu, V.; Soukup, T.	2008	Autopsies

immunohistochemical and immunoblotting methods			
Treatment of combined hyperlipidemia with fluvastatin and gemfibrozil, alone or in combination, does not induce muscle damage	Smit, J. W.; Jansen, G. H.; de Bruin, T. W.; Erkelens, D. W.	1995	Did not perform fiber type analysis
Metabo- and mechanoreceptor expression in human heart failure: Relationships with the locomotor muscle afferent influence on exercise responses	Smith, J. R.; Hart, C. R.; Ramos, P. A.; Akinsanya, J. G.; Lanza, I. R.; Joyner, M. J.; Curry, T. B.; Olson, T. P.	2020	Did not perform fiber type analysis
Energy metabolism in type I and type II human muscle fibres during short term electrical stimulation at different frequencies	SÃ¶derlund, K.; Greenhaff, P. L.; Hultman, E.	1992	Does not include male and female data
Effects of delayed freezing on content of phosphagens in human skeletal muscle biopsy samples	SÃ¶derlund, K.; Hultman, E.	1986	Did not perform fiber type analysis
ATP content in single fibres from human skeletal muscle after electrical stimulation and during recovery	SÃ¶derlund, K.; Hultman, E.	1990	Does not include male and female data
ATP and phosphocreatine changes in single human muscle fibers after intense electrical stimulation	SÃ¶derlund, K.; Hultman, E.	1991	Does not include male and female data
Increased antiangiogenic protein expression in the skeletal muscle of diabetic swine and patients	Sodha, N. R.; Boodhwani, M.; Clements, R. T.; Xu, S. H.; Khabbaz, K. R.; Sellke, F. W.	2008	Did not perform fiber type analysis
Muscle-Saturated Bioactive Lipids Are Increased with Aging and Influenced by High-Intensity Interval Training	SÃ¸gaard, D.; Baranowski, M.; Larsen, S.; Taulo Lund, M.; Munk Scheuer, C.; Vestergaard Abildskov, C.; Greve Dideriksen, S.; Dela, F.; Wulff Helge, J.	2019	Did not perform fiber type analysis
Training Does Not Alter Muscle Ceramide and Diacylglycerol in Offsprings of Type 2 Diabetic Patients Despite Improved Insulin Sensitivity	SÃ¸gaard, D.; Ã˜stergÃ¥rd, T.; Blachnio-Zabielska, A. U.; Baranowski, M.; VigelsÃ¸, A. H.; Andersen, J. L.; Dela, F.; Helge, J. W.	2016	Does not include male and female data
Limited expression of slow tonic myosin heavy chain in human cranial muscles	Sokoloff, A. J.; Li, H.; Burkholder, T. J.	2007	Case studies
Immunohistochemical characterization of slow and fast myosin heavy chain composition of muscle fibres in the styloglossus muscle of the human and macaque (Macaca rhesus)	Sokoloff, A. J.; Yang, B.; Li, H.; Burkholder, T. J.	2007	No cross-sectional area data
Exercise-dependent increases in protein synthesis are accompanied by chromatin modifications and increased MRTF-SRF signalling	Solagna, F.; Nogara, L.; Dyar, K. A.; Greulich, F.; Mir, A. A.; TÃ¼rk, C.; Bock, T.; Geremia, A.; Baraldo, M.; Sartori, R.; Farup, J.; Uhlenhaut, H.; Vissing, K.; KrÃ¼ger, M.; Blaauw, B.	2020	Did not perform fiber type analysis
Ethanol produces muscle damage in human volunteers	Song, S. K.; Rubin, E.	1972	Does not include males and females
TDP-43 in skeletal muscle of patients affected with amyotrophic lateral sclerosis	SorarÃº, G.; Orsetti, V.; Buratti, E.; Baralle, F.; Cima, V.; Volpe, M.; D'Ascenzo, C.; Palmieri, A.; Koutsikos, K.; Pegoraro, E.; Angelini, C.	2010	Did not perform fiber type analysis
Activities of mitochondrial complexes correlate with nNOS amount in muscle from ALS patients	SorarÃ¹, G.; Vergani, L.; Fedrizzi, L.; D'Ascenzo, C.; Polo, A.; Bernazzi, B.; Angelini, C.	2007	Did not perform fiber type analysis

An altered response in macrophage phenotype following damage in aged human skeletal muscle: implications for skeletal muscle repair	Sorensen, J. R.; Kaluhiokalani, J. P.; Hafen, P. S.; Deyhle, M. R.; Parcell, A. C.; Hyldahl, R. D.	2019	Did not perform fiber type analysis
Preparation and Culture of Myogenic Precursor Cells/Primary Myoblasts from Skeletal Muscle of Adult and Aged Humans	Soriano-Arroquia, A.; Clegg, P. D.; Molloy, A. P.; Goljanek-Whysall, K.	2017	Does not include males and females
Oral digoxin effects on exercise performance, K(+) regulation and skeletal muscle Na(+) ,K(+) -ATPase in healthy humans	Sostaric, S.; Petersen, A. C.; Goodman, C. A.; Gong, X.; Aw, T. J.; Brown, M. J.; Garnham, A.; Steward, C. H.; Murphy, K. T.; Carey, K. A.; Leppik, J.; Fraser, S. F.; Cameron-Smith, D.; Krum, H.; Snow, R. J.; McKenna, M. J.	2022	Does not include males and females
Unusual intrafusal fibres in human muscle spindles	Soukup, T.; Thornell, L. E.	1999	Case studies
Skeletal Muscle Phenotype in Patients Undergoing Long-Term Hemodialysis Awaiting Kidney Transplantation	Souweine, J. S.; Gouzi, F.; Badia, Ã‰; Pomies, P.; Garrigue, V.; Morena, M.; Hayot, M.; Mercier, J.; Ayoub, B.; Quintrec, M. L.; Raynaud, F.; Cristol, J. P.	2021	No cross-sectional area data
Spinal muscle in scoliosis. Part 2. The proportion and size of type 1 and type 2 skeletal muscle fibres measured using a computer-controlled microscope	Spencer, G. S.; Eccles, M. J.	1976	Does not report sex of subjects
Role of glycogen in control of glycolysis and IMP formation in human muscle during exercise	Spencer, M. K.; Katz, A.	1991	Did not perform fiber type analysis
Measurement of totally activated pyruvate dehydrogenase complex activity in human muscle: evaluation of a useful assay	Sperl, W.; Trijbels, J. M.; Ruitenbeek, W.; van Laack, H. L.; Janssen, A. J.; Kerkhof, C. M.; Sengers, R. C.	1993	Did not perform fiber type analysis
Impaired copper and iron metabolism in blood cells and muscles of patients affected by copper deficiency myeloneuropathy	Spinazzi, M.; Sghirlanzoni, A.; Salviati, L.; Angelini, C.	2014	Did not perform fiber type analysis
pH measurement in human skeletal muscle samples: effect of phosphagen hydrolysis	Spriet, L. L.; SÃ¶derlund, K.; Thomson, J. A.; Hultman, E.	1986	Did not perform fiber type analysis
Pyruvate dehydrogenase activation and kinase expression in human skeletal muscle during fasting	Spriet, L. L.; Tunstall, R. J.; Watt, M. J.; Mehan, K. A.; Hargreaves, M.; Cameron-Smith, D.	2004	Did not perform fiber type analysis
Increased DNA fragmentation and ultrastructural changes in fibromyalgic muscle fibres	Sprott, H.; Salemi, S.; Gay, R. E.; Bradley, L. A.; AlarcÃ³n, G. S.; Oh, S. J.; Michel, B. A.; Gay, S.	2004	Does not include male and female data
Muscle and nerve pathology in Dunnigan familial partial lipodystrophy	Spuler, S.; Kalbhenn, T.; Zabojszcza, J.; van Landeghem, F. K.; Ludtke, A.; Wenzel, K.; Koehnlein, M.; Schuelke, M.; LÃ¼demann, L.; Schmidt, H. H.	2007	Does not report sex of subjects
Improved medium with EGF and BSA for differentiated human skeletal muscle cells	St Clair, J. A.; Meyer-Demarest, S. D.; Ham, R. G.	1992	No healthy subjects or controls
Induction of adiponectin gene expression in human myotubes by an adiponectin-containing HEK293 cell culture supernatant	Staiger, H.; Kausch, C.; Guirguis, A.; Weisser, M.; Maerker, E.; Stumvoll, M.; Lammers, R.; Machicao, F.; HÃ¤ring, H. U.	2003	Did not perform fiber type analysis
Characterization of human oro-facial and masticatory muscles with respect to fibre types,	StÃ¥l, P.	1994	Not peer reviewed (ex:

myosins and capillaries. Morphological, enzyme-histochemical, immuno-histochemical and biochemical investigations			thesis, conference poster)
Muscle-specific enzyme activity patterns of the capillary bed of human oro-facial, masticatory and limb muscles	StÃ¥l, P.; Eriksson, P. O.; Thornell, L. E.	1995	Autopsies
Characterisation of human soft palate muscles with respect to fibre types, myosins and capillary supply	StÃ¥l, P. S.; Lindman, R.	2000	Does not include male and female data
Capillary supply of the soft palate muscles is reduced in long-term habitual snorers	StÃ¥l, P. S.; Lindman, R.; Johansson, B.	2009	Does not include male and female data
Dynamic electromyography and muscle biopsy changes in a 4-year follow-up: study of patients with a history of polio	StÃ¥lberg, E.; Grimby, G.	1995	Does not include male and female data
Effects of Î²-hydroxy-Î²-methylbutyrate on skeletal muscle mitochondrial content and dynamics, and lipids after 10 days of bed rest in older adults	Standley, R. A.; Distefano, G.; Pereira, S. L.; Tian, M.; Kelly, O. J.; Coen, P. M.; Deutz, N. E. P.; Wolfe, R. R.; Goodpaster, B. H.	2017	Does not include male and female data
Prostaglandin E2 induces transcription of skeletal muscle mass regulators interleukin-6 and muscle RING finger-1 in humans	Standley, R. A.; Liu, S. Z.; Jemiolo, B.; Trappe, S. W.; Trappe, T. A.	2013	Does not include males and females
Proteolytic and autolytic activity of skeletal muscle in Turner's syndrome	StÄƒnescu, V.; Biener, J.; Grigorescu, A.; Diaconescu, C.; Maximilian, C.	1967	Did not perform fiber type analysis
The effect of parenteral nutritional repletion on muscle water and electrolytes. Implications for body composition	Starker, P. M.; Askanazi, J.; Lasala, P. A.; Elwyn, D. H.; Gump, F. E.; Kinney, J. M.	1983	Does not include males and females
Correlation between myofibrillar ATPase activity and myosin heavy chain composition in single human muscle fibers	Staron, R. S.	1991	Case studies
Lipid depletion and repletion in skeletal muscle following a marathon	Staron, R. S.; Hikida, R. S.; Murray, T. F.; Hagerman, F. C.; Hagerman, M. T.	1989	Does not include males and females
Sensitivity of CPT I to malonyl-CoA in trained and untrained human skeletal muscle	Starritt, E. C.; Howlett, R. A.; Heigenhauser, G. J.; Spriet, L. L.	2000	Did not perform fiber type analysis
Extracellular matrix disruption and pain after eccentric muscle action	Stauber, W. T.; Clarkson, P. M.; Fritz, V. K.; Evans, W. J.	1990	Did not perform fiber type analysis
The effects of age and resistance loading on skeletal muscle ribosome biogenesis	Stec, M. J.; Mayhew, D. L.; Bamman, M. M.	2015	Did not perform fiber type analysis
Adipose tissue, but not skeletal muscle, sirtuin 1 expression is decreased in obesity and related to insulin sensitivity	Stefanowicz, M.; NikoÅ‚ajuk, A.; Matulewicz, N.; Karczewska-Kupczewska, M.	2018	Does not include males and females
Expression of myosin heavy chain isoforms in skeletal muscle of patients with peripheral arterial occlusive disease	Steinacker, J. M.; Opitz-Gress, A.; Baur, S.; Lormes, W.; Bolkart, K.; Sunder-Plassmann, L.; Liewald, F.; Lehmann, M.; Liu, Y.	2000	Does not include male and female data
Effects of rotator cuff ruptures on the cellular and intracellular composition of the human supraspinatus muscle	Steinbacher, P.; Tauber, M.; Kogler, S.; Stoiber, W.; Resch, H.; SÃ¤nger, A. M.	2010	No healthy subjects or controls

Leptin increases FA oxidation in lean but not obese human skeletal muscle: evidence of peripheral leptin resistance	Steinberg, G. R.; Parolin, M. L.; Heigenhauser, G. J.; Dyck, D. J.	2002	Does not include males and females
Adenine nucleotide loss in the skeletal muscles during exercise in chronic obstructive pulmonary disease	Steiner, M. C.; Evans, R.; Deacon, S. J.; Singh, S. J.; Patel, P.; Fox, J.; Greenhaff, P. L.; Morgan, M. D.	2005	Did not perform fiber type analysis
Progressive increase in human skeletal muscle AMPKalpha2 activity and ACC phosphorylation during exercise	Stephens, T. J.; Chen, Z. P.; Canny, B. J.; Michell, B. J.; Kemp, B. E.; McConell, G. K.	2002	Did not perform fiber type analysis
Short-term intensified cycle training alters acute and chronic responses of PGC1Î± and Cytochrome C oxidase IV to exercise in human skeletal muscle	Stepto, N. K.; Benziane, B.; Wadley, G. D.; Chibalin, A. V.; Canny, B. J.; Eynon, N.; McConell, G. K.	2012	Does not include males and females
Electrophoretic determination of the myosin/actin ratio in the diagnosis of critical illness myopathy	Stibler, H.; EdstrÃ¶m, L.; Ahlbeck, K.; Remahl, S.; Ansved, T.	2003	Does not include male and female data
Myofibrillar ATPase activity in skinned human skeletal muscle fibres: fibre type and temperature dependence	Stienen, G. J.; Kiers, J. L.; Bottinelli, R.; Reggiani, C.	1996	Does not include males and females
Histochemical and immunohistochemical profile of human and rat ocular medial rectus muscles	Stirn Kranjc, B.; Smerdu, V.; Erzen, I.	2009	Autopsies
Fibre type related changes in the metabolic profile and fibre diameter of human vastus medialis muscle after anterior cruciate ligament rupture	Stockmar, C.; Lill, H.; Trapp, A.; Josten, C.; Punkt, K.	2006	No healthy subjects or controls
Divergence exists in the subcellular distribution of intramuscular triglyceride in human skeletal muscle dependent on the choice of lipid dye	Strauss, J. A.; Shepherd, D. A.; Macey, M.; Jevons, E. F. P.; Shepherd, S. O.	2020	Does not include males and females
CX3CL1--a macrophage chemoattractant induced by a single bout of exercise in human skeletal muscle	StrÃ¶mberg, A.; Olsson, K.; Dijksterhuis, J. P.; Rullman, E.; Schulte, G.; Gustafsson, T.	2016	Did not perform fiber type analysis
Cycle training increased GLUT4 and activation of mammalian target of rapamycin in fast twitch muscle fibers	Stuart, C. A.; Howell, M. E.; Baker, J. D.; Dykes, R. J.; Duffourc, M. M.; Ramsey, M. W.; Stone, M. H.	2010	Does not include male and female data
Insulin-stimulated translocation of glucose transporter (GLUT) 12 parallels that of GLUT4 in normal muscle	Stuart, C. A.; Howell, M. E.; Zhang, Y.; Yin, D.	2009	Did not perform fiber type analysis
Myosin content of individual human muscle fibers isolated by laser capture microdissection	Stuart, C. A.; Stone, W. L.; Howell, M. E.; Brannon, M. F.; Hall, H. K.; Gibson, A. L.; Stone, M. H.	2016	Does not include male and female data
Comparison of GLUT1, GLUT3, and GLUT4 mRNA and the subcellular distribution of their proteins in normal human muscle	Stuart, C. A.; Wen, G.; Gustafson, W. C.; Thompson, E. A.	2000	Did not perform fiber type analysis
Altered GLUT1 and GLUT3 gene expression and subcellular redistribution of GLUT4: protein in muscle from patients with acanthosis nigricans and severe insulin resistance	Stuart, C. A.; Wen, G.; Williamson, M. E.; Jiang, J.; Gilkison, C. R.; Blackwell, S. J.; Nagamani, M.; Ferrando, A. A.	2001	Did not perform fiber type analysis
Myosin light chain phosphorylation and contractile performance of human skeletal muscle	Stuart, D. S.; Lingley, M. D.; Grange, R. W.; Houston, M. E.	1988	Did not perform fiber type analysis

Physiological properties of human diaphragm muscle fibres and the effect of chronic obstructive pulmonary disease	Stubbings, A. K.; Moore, A. J.; Dusmet, M.; Goldstraw, P.; West, T. G.; Polkey, M. I.; Ferenczi, M. A.	2008	Does not include male and female data
Rigid spine syndrome (vacuolar variant). A quantitative electromyograhic study	StÃ¼bgen, J. P.	2007	No healthy subjects or controls
Glucocorticoid receptor concentrations in muscle biopsies from patients with neuromuscular diseases	Stuerenburg, H. J.; Kunze, K.	1999	Did not perform fiber type analysis
Age related profiles of free amino acids in human skeletal muscle	Stuerenburg, H. J.; Stangneth, B.; Schoser, B. G.	2006	Did not perform fiber type analysis
Relationships between urinary inositol excretions and whole-body glucose tolerance and skeletal muscle insulin receptor phosphorylation	Stull, A. J.; Thyfault, J. P.; Haub, M. D.; Ostlund, R. E., Jr.; Campbell, W. W.	2008	Did not perform fiber type analysis
Skeletal muscle protein tyrosine phosphatase 1B regulates insulin sensitivity in African Americans	Stull, A. J.; Wang, Z. Q.; Zhang, X. H.; Yu, Y.; Johnson, W. D.; Cefalu, W. T.	2012	Did not perform fiber type analysis
Effect of insulin on human skeletal muscle mitochondrial ATP production, protein synthesis, and mRNA transcripts	Stump, C. S.; Short, K. R.; Bigelow, M. L.; Schimke, J. M.; Nair, K. S.	2003	Did not perform fiber type analysis
Thrombospondin-1 mediates muscle damage in brachio-cervical inflammatory myopathy and systemic sclerosis	SuÃ¡rez-Calvet, X.; Alonso-PÃ©rez, J.; CastellvÃ, I.; Carrasco-Rozas, A.; FernÃ¡ndez-SimÃ³n, E.; Zamora, C.; MartÃ-nez-MartÃnez, L.; Alonso-JimÃ©nez, A.; Rojas-GarcÃa, R.; TurÃ³n, J.; Querol, L.; de Luna, N.; Milena-Millan, A.; Corominas, H.; Castillo, D.; CortÃ©s-Vicente, E.; Illa, I.; Gallardo, E.; DÃaz-Manera, J.	2020	No healthy subjects or controls
Isolation of human fibroadipogenic progenitors and satellite cells from frozen muscle biopsies	SuÃ¡rez-Calvet, X.; FernÃ¡ndez-SimÃ³n, E.; PiÃ±ol-Jurado, P.; Alonso-PÃ©rez, J.; Carrasco-Rozas, A.; LleixÃ , C.; LÃ³pez-FernÃ¡ndez, S.; Pons, G.; Soria, L.; Bigot, A.; Mouly, V.; Illa, I.; Gallardo, E.; Jaiswal, J. K.; DÃaz-Manera, J.	2021	Did not perform fiber type analysis
Altered RIG-I/DDX58-mediated innate immunity in dermatomyositis	SuÃ¡rez-Calvet, X.; Gallardo, E.; Nogales-Gadea, G.; Querol, L.; Navas, M.; DÃaz-Manera, J.; Rojas-Garcia, R.; Illa, I.	2014	Did not perform fiber type analysis
Cardiorespiratory responses, nitric oxide production and inflammatory factors in patients with myocardial infarction after rehabilitation	Subiela, J. V.; Torres, S. H.; De Sanctis, J. B.; HernÃ¡ndez, N.	2018	Does not include males and females
Aging affects the transcriptional regulation of human skeletal muscle disuse atrophy	Suetta, C.; Frandsen, U.; Jensen, L.; Jensen, M. M.; Jespersen, J. G.; Hvid, L. G.; Bayer, M.; Petersson, S. J.; SchrÃ¸der, H. D.; Andersen, J. L.; Heinemeier, K. M.; Aagaard, P.; Schjerling, P.; Kjaer, M.	2012	Does not include males and females
Fas and Fas ligand interaction induces apoptosis in inflammatory myopathies: CD4+ T cells cause muscle cell injury directly in polymyositis	Sugiura, T.; Murakawa, Y.; Nagai, A.; Kondo, M.; Kobayashi, S.	1999	Did not perform fiber type analysis

Skeletal Muscle and Peripheral Nerve Histopathology in COVID-19	Suh, J.; Mukerji, S. S.; Collens, S. I.; Padera, R. F., Jr.; Pinkus, G. S.; Amato, A. A.; Solomon, I. H.	2021	Does not include male and female data
Skeletal muscle biochemistry and histology in ambulatory patients with long-term heart failure	Sullivan, M. J.; Green, H. J.; Cobb, F. R.	1990	Does not include males and females
Altered skeletal muscle metabolic response to exercise in chronic heart failure. Relation to skeletal muscle aerobic enzyme activity	Sullivan, M. J.; Green, H. J.; Cobb, F. R.	1991	Does not include males and females
Ultracytochemical localization of adenyl cyclase activity in the human pathologic skeletal muscle fibers	Sumi, T.; Hizawa, K.	1978	No healthy subjects or controls
Ultracytochemical localization of adenyl cyclase in the human pathologic skeletal muscle fibers	Sumi, T.; Hizawa, K.	1979	No healthy subjects or controls
Intramuscular renin-angiotensin system is activated in human muscular dystrophy	Sun, G.; Haginoya, K.; Dai, H.; Chiba, Y.; Uematsu, M.; Hino-Fukuyo, N.; Onuma, A.; Iinuma, K.; Tsuchiya, S.	2009	Only in children (0-17 years)
Exercise and training during graded leg ischaemia in healthy man with special reference to effects on skeletal muscle	Sundberg, C. J.	1994	Does not include males and females
Chronic alcoholic skeletal muscle myopathy: a clinical, histological and biochemical assessment of muscle lipid	Sunnasy, D.; Cairns, S. R.; Martin, F.; Slavin, G.; Peters, T. J.	1983	Autopsies
Muscular performance in heart failure	Sunnerhagen, K. S.; Cider, A.; Schaufelberger, M.; Hedberg, M.; Grimby, G.	1998	Does not include male and female data
Enzyme activities and glycogen concentration in skeletal muscle in alcoholism. The effect of abstinence and physical conditioning	Suominen, H.; Forsberg, S.; Heikkinen, E.; Osterback, L.	1974	Does not include males and females
Identification of a program of contractile protein gene expression initiated upon skeletal muscle differentiation	Sutherland, C. J.; Esser, K. A.; Elsom, V. L.; Gordon, M. L.; Hardeman, E. C.	1993	Animal study
Quadriceps muscle strength in scoliosis	Swallow, E. B.; Barreiro, E.; Gosker, H.; Sathyapala, S. A.; Sanchez, F.; Hopkinson, N. S.; Moxham, J.; Schols, A.; Gea, J.; Polkey, M. I.	2009	Does not include males and females
A novel technique for nonvolitional assessment of quadriceps muscle endurance in humans	Swallow, E. B.; Gosker, H. R.; Ward, K. A.; Moore, A. J.; Dayer, M. J.; Hopkinson, N. S.; Schols, A. M.; Moxham, J.; Polkey, M. I.	2007	Does not include males and females
Depression of force production and ATPase activity in different types of human skeletal muscle fibers from patients with chronic heart failure	Szentesi, P.; Bekedam, M. A.; van Beek-Harmsen, B. J.; van der Laarse, W. J.; Zaremba, R.; Boonstra, A.; Visser, F. C.; Stienen, G. J.	2005	Does not include male and female data
In focus in HCB	Taatjes, D. J.; Roth, J.	2019	Review (ex: systematic review/meta-analysis)
Distal myopathy with rimmed vacuoles: impaired O-glycan formation in muscular glycoproteins	Tajima, Y.; Uyama, E.; Go, S.; Sato, C.; Tao, N.; Kotani, M.; Hino, H.; Suzuki, A.; Sanai, Y.; Kitajima, K.; Sakuraba, H.	2005	Does not include males and females

Myosin light chain components in single muscle fibers of Duchenne muscular dystrophy	Takagi, A.; Ishiura, S.; Nonaka, I.; Sugita, H.	1982	No healthy subjects or controls
Relationships between fiber composition and NMR measurements in human skeletal muscle	Takahashi, H.; Kuno, S. Y.; Katsuta, S.; Shimojo, H.; Masuda, K.; Yoshioka, H.; Anno, I.; Itai, Y.	1996	Does not include males and females
Lysosomal and nonlysosomal hydrolases of skeletal muscle in neuromuscular diseases	Takala, T. E.; MyllylÃ¤, V. V.; Salminen, A.; Tolonen, U.; Hassinen, I. E.; Vihko, V.	1983	Does not report sex of subjects
The carnosine content of vastus lateralis is elevated in resistance-trained bodybuilders	Tallon, M. J.; Harris, R. C.; Boobis, L. H.; Fallowfield, J. L.; Wise, J. A.	2005	Does not include males and females
Immunohistochemical staining of normal and Graves' extraocular muscle	Tallstedt, L.; Norberg, R.	1988	No healthy subjects or controls
Phenotypic adaptations in human muscle fibers 6 and 24 wk after spinal cord injury	Talmadge, R. J.; Castro, M. J.; Apple, D. F., Jr.; Dudley, G. A.	2002	Does not include males and females
Skeletal muscle extracellular matrix remodeling after short-term overfeeding in healthy humans	Tam, C. S.; Chaudhuri, R.; Hutchison, A. T.; Samocha-Bonet, D.; Heilbronn, L. K.	2017	Did not perform fiber type analysis
Low macrophage accumulation in skeletal muscle of obese type 2 diabetics and elderly subjects	Tam, C. S.; Sparks, L. M.; Johannsen, D. L.; Covington, J. D.; Church, T. S.; Ravussin, E.	2012	Did not perform fiber type analysis
Age-related differences in lean mass, protein synthesis and skeletal muscle markers of proteolysis after bed rest and exercise rehabilitation	Tanner, R. E.; Brunker, L. B.; Agergaard, J.; Barrows, K. M.; Briggs, R. A.; Kwon, O. S.; Young, L. M.; Hopkins, P. N.; Volpi, E.; Marcus, R. L.; LaStayo, P. C.; Drummond, M. J.	2015	Did not perform fiber type analysis
NF-Î°B activity in muscle from obese and type 2 diabetic subjects under basal and exercise-stimulated conditions	Tantiwong, P.; Shanmugasundaram, K.; Monroy, A.; Ghosh, S.; Li, M.; DeFronzo, R. A.; Cersosimo, E.; Sriwijitkamol, A.; Mohan, S.; Musi, N.	2010	Did not perform fiber type analysis
Diagnostic utility of skin biopsy in dystrophinopathies	Tanveer, N.; Sharma, M. C.; Sarkar, C.; Gulati, S.; Kalra, V.; Singh, S.; Bhatia, R.	2009	No healthy subjects or controls
Osteoporosis and sarcopenia: the connections	Tarantino, U.; Baldi, J.; Celi, M.; Rao, C.; Liuni, F. M.; Iundusi, R.; Gasbarra, E.	2013	Review (ex: systematic review/meta-analysis)
Carbohydrate loading and metabolism during exercise in men and women	Tarnopolsky, M. A.; Atkinson, S. A.; Phillips, S. M.; MacDougall, J. D.	1995	Did not perform fiber type analysis
Electrical pulse stimulation: an in vitro exercise model for the induction of human skeletal muscle cell hypertrophy. A proof-of-concept study	Tarum, J.; Folkesson, M.; Atherton, P. J.; Kadi, F.	2017	Did not perform fiber type analysis
Motor protein function in skeletal abdominal muscle of cachectic cancer patients	Taskin, S.; Stumpf, V. I.; Bachmann, J.; Weber, C.; Martignoni, M. E.; Friedrich, O.	2014	No healthy subjects or controls
High density lipoprotein subfractions in relation to lipoprotein lipase activity of tissues in man--	Taskinen, M. R.; NikkilÃ¤, E. A.	1981	Did not perform fiber type analysis

evidence for reciprocal regulation of HDL2 and HDL3 levels by lipoprotein lipase			
The effect of branched chain amino acids on skeletal muscle mitochondrial function in young and elderly adults	Tatpati, L. L.; Irving, B. A.; Tom, A.; Bigelow, M. L.; Klaus, K.; Short, K. R.; Nair, K. S.	2010	Did not perform fiber type analysis
Skeletal muscle analysis of wheelchair athletes	Taylor, A. W.; McDonnell, E.; Royer, D.; Loiselle, R.; Lush, N.; Steadward, R.	1979	No healthy subjects or controls
Acute and chronic effect of sprint interval training combined with postexercise blood-flow restriction in trained individuals	Taylor, C. W.; Ingham, S. A.; Ferguson, R. A.	2016	Does not include males and females
Validation of 13C NMR measurement of human skeletal muscle glycogen by direct biochemical assay of needle biopsy samples	Taylor, R.; Price, T. B.; Rothman, D. L.; Shulman, R. G.; Shulman, G. I.	1992	Did not perform fiber type analysis
Actomyosin ATPase activity of human laryngeal muscles	Teig, E.; Dahl, H. A.; Thorkelsen, H.	1978	Does not include males and females
Ca(2+) dependency of limb muscle fiber contractile mechanics in young and older adults	Teigen, L. E.; Sundberg, C. W.; Kelly, L. J.; Hunter, S. K.; Fitts, R. H.	2020	Single Fiber Analysis
Type 2 fiber predominance in muscle cramp and exertional myalgia	Telerman-Toppet, N.; Bacq, M.; Khoubesserian, P.; CoÃ«rs, C.	1985	Does not report sex of subjects
Cytochrome c oxidase deficiency in human posterior cricoarytenoid muscle	Tellis, C. M.; Rosen, C.; Close, J. M.; Horton, M.; Yaruss, J. S.; Verdolini-Abbott, K.; Sciote, J. J.	2011	No healthy subjects or controls
IRF2BP2 is a skeletal and cardiac muscle-enriched ischemia-inducible activator of VEGFA expression	Teng, A. C.; Kuraitis, D.; Deeke, S. A.; Ahmadi, A.; Dugan, S. G.; Cheng, B. L.; Crowson, M. G.; Burgon, P. G.; Suuronen, E. J.; Chen, H. H.; Stewart, A. F.	2010	Does not report sex of subjects
Endurance training-induced changes in insulin sensitivity and gene expression	Teran-Garcia, M.; Rankinen, T.; Koza, R. A.; Rao, D. C.; Bouchard, C.	2005	Did not perform fiber type analysis
Muscle regeneration and satellite cells in Fukuyama type congenital muscular dystrophy	Terasawa, K.	1986	Only in children (0-17 years)
Throwing performance after resistance training and detraining	Terzis, G.; Stratakos, G.; Manta, P.; Georgiadis, G.	2008	Does not include males and females
Relationship between lactate accumulation, LDH activity, LDH isozyme and fibre type distribution in human skeletal muscle	Tesch, P.; SjÃ¶din, B.; Karlsson, J.	1978	Does not include males and females
Muscle fatigue and its relation to lactate accumulation and LDH activity in man	Tesch, P.; SjÃ¶din, B.; Thorstensson, A.; Karlsson, J.	1978	Does not include males and females
Muscle fiber type composition and G-tolerance	Tesch, P. A.; Balldin, U. I.	1984	Does not include males and females
Influence of lactate accumulation of EMG frequency spectrum during repeated concentric contractions	Tesch, P. A.; Komi, P. V.; Jacobs, I.; Karlsson, J.; Viitasalo, J. T.	1983	Does not report sex of subjects
Atrophy and hypertrophy signalling in the diaphragm of patients with COPD	Testelmans, D.; Crul, T.; Maes, K.; Agten, A.; Crombach, M.; Decramer, M.; Gayan-Ramirez, G.	2010	Does not include male and female data

Postoperative inflammation and insulin resistance in relation to body composition, adiposity and carbohydrate treatment: A randomised controlled study	Tewari, N.; Awad, S.; DuÅ¡ka, F.; Williams, J. P.; Bennett, A.; Macdonald, I. A.; Lobo, D. N.	2019	No healthy subjects or controls
Tumour necrosis factor-mediated cell death pathways do not contribute to muscle fibre death in dystrophinopathies	Tews, D. S.	2005	No healthy subjects or controls
Does habitual dietary intake influence myofiber hypertrophy in response to resistance training? A cluster analysis	Thalacker-Mercer, A. E.; Petrella, J. K.; Bamman, M. M.	2009	Did not perform fiber type analysis
Alcohol-induced autophagy contributes to loss in skeletal muscle mass	Thapaliya, S.; Runkana, A.; McMullen, M. R.; Nagy, L. E.; McDonald, C.; Naga Prasad, S. V.; Dasarathy, S.	2014	Did not perform fiber type analysis
Electrical stimulation-induced changes in performance and fiber type proportion of human knee extensor muscles	ThÃ©riault, R.; Boulay, M. R.; ThÃ©riault, G.; Simoneau, J. A.	1996	Does not report sex of subjects
Human skeletal muscle adaptation in response to chronic low-frequency electrical stimulation	ThÃ©riault, R.; ThÃ©riault, G.; Simoneau, J. A.	1994	Did not perform fiber type analysis
Inheritance of human muscle enzyme adaptation to isokinetic strength training	Thibault, M. C.; Simoneau, J. A.; CÃ´tÃ©, C.; Boulay, M. R.; LagassÃ©, P.; Marcotte, M.; Bouchard, C.	1986	Did not perform fiber type analysis
Monocarboxylate transporters, blood lactate removal after supramaximal exercise, and fatigue indexes in humans	Thomas, C.; Perrey, S.; Lambert, K.; Hugon, G.; Mornet, D.; Mercier, J.	2005	Does not include males and females
Relationships between maximal muscle oxidative capacity and blood lactate removal after supramaximal exercise and fatigue indexes in humans	Thomas, C.; Sirvent, P.; Perrey, S.; Raynaud, E.; Mercier, J.	2004	Does not include males and females
Rotator cuff tear state modulates self-renewal and differentiation capacity of human skeletal muscle progenitor cells	Thomas, K. A.; Gibbons, M. C.; Lane, J. G.; Singh, A.; Ward, S. R.; Engler, A. J.	2017	No healthy subjects or controls
Intensive training and reduced volume increases muscle FXYD1 expression and phosphorylation at rest and during exercise in athletes	Thomassen, M.; Gunnarsson, T. P.; Christensen, P. M.; Pavlovic, D.; Shattock, M. J.; Bangsbo, J.	2016	Did not perform fiber type analysis
Abundance of ClC-1 chloride channel in human skeletal muscle: fiber type specific differences and effect of training	Thomassen, M.; Hostrup, M.; Murphy, R. M.; Cromer, B. A.; Skovgaard, C.; Gunnarsson, T. P.; Christensen, P. M.; Bangsbo, J.	2018	Does not include male and female data
Fibre type-specific change in FXYD1 phosphorylation during acute intense exercise in humans	Thomassen, M.; Murphy, R. M.; Bangsbo, J.	2013	Does not include males and females
Protein kinase CÎ± activity is important for contraction-induced FXYD1 phosphorylation in skeletal muscle	Thomassen, M.; Rose, A. J.; Jensen, T. E.; Maarbjerg, S. J.; Bune, L.; Leitges, M.; Richter, E. A.; Bangsbo, J.; Nordsborg, N. B.	2011	Does not include males and females
Influence of dietary nitrate supplementation on physiological and muscle metabolic adaptations to sprint interval training	Thompson, C.; Wylie, L. J.; Blackwell, J. R.; Fulford, J.; Black, M. I.; Kelly, J.; McDonagh, S. T.; Carter, J.; Bailey, S. J.; Vanhatalo, A.; Jones, A. M.	2017	Does not include male and female data
The repeated bout effect and heat shock proteins: intramuscular HSP27 and HSP70	Thompson, H. S.; Clarkson, P. M.; Scordilis, S. P.	2002	Did not perform fiber type analysis

expression following two bouts of eccentric exercise in humans			
Myoglobin content of granular cell tumor of the tongue. An immunoperoxidase study	Thompson, S. H.	1984	Did not perform fiber type analysis
Exercise and insulin cause GLUT-4 translocation in human skeletal muscle	Thorell, A.; Hirshman, M. F.; Nygren, J.; Jorfeldt, L.; Wojtaszewski, J. F.; Dufresne, S. D.; Horton, E. S.; Ljungqvist, O.; Goodyear, L. J.	1999	Did not perform fiber type analysis
Surgery-induced insulin resistance in human patients: relation to glucose transport and utilization	Thorell, A.; Nygren, J.; Hirshman, M. F.; Hayashi, T.; Nair, K. S.; Horton, E. S.; Goodyear, L. J.; Ljungqvist, O.	1999	Did not perform fiber type analysis
Satellite cell dysfunction contributes to the progressive muscle atrophy in myotonic dystrophy type 1	Thornell, L. E.; LindstÃ¶m, M.; Renault, V.; Klein, A.; Mouly, V.; Ansved, T.; Butler-Browne, G.; Furling, D.	2009	Does not report sex of subjects
Store-operated Ca(2+) entry (SOCE) contributes to normal skeletal muscle contractility in young but not in aged skeletal muscle	Thornton, A. M.; Zhao, X.; Weisleder, N.; Brotto, L. S.; Bougoin, S.; Nosek, T. M.; Reid, M.; Hardin, B.; Pan, Z.; Ma, J.; Parness, J.; Brotto, M.	2011	Animal study
Actomyosin ATPase, myokinase, CPK and LDH in human fast and slow twitch muscle fibres	Thorstensson, A.; SjÃ¶din, B.; Tesch, P.; Karlsson, J.	1977	Does not include males and females
Protein supplementation improves physical performance in frail elderly people: a randomized, double-blind, placebo-controlled trial	Tieland, M.; van de Rest, O.; Dirks, M. L.; van der Zwaluw, N.; Mensink, M.; van Loon, L. J.; de Groot, L. C.	2012	Does not include male and female data
Skeletal muscle fiber distribution influences serum high-density lipoprotein cholesterol level	Tikkanen, H. O.; NÃ¤veri, H.; HÃ¤rkÃ¶nen, M.	1996	Review (ex: systematic review/meta-analysis)
Changes in myofibrillar protein composition of human diaphragm elicited by congestive heart failure	Tikunov, B. A.; Mancini, D.; Levine, S.	1996	Does not include male and female data
Human muscle gene expression responses to endurance training provide a novel perspective on Duchenne muscular dystrophy	Timmons, J. A.; Larsson, O.; Jansson, E.; Fischer, H.; Gustafsson, T.; Greenhaff, P. L.; Ridden, J.; Rachman, J.; Peyrard-Janvid, M.; Wahlestedt, C.; Sundberg, C. J.	2005	Does not include males and females
Timing of amino acid-carbohydrate ingestion alters anabolic response of muscle to resistance exercise	Tipton, K. D.; Rasmussen, B. B.; Miller, S. L.; Wolf, S. E.; Owens-Stovall, S. K.; Petrini, B. E.; Wolfe, R. R.	2001	Did not perform fiber type analysis
Impact of Amyotrophic Lateral Sclerosis on Slow Tonic Myofiber Composition in Human Extraocular Muscles	Tjust, A. E.; Danielsson, A.; Andersen, P. M.; BrÃ¤nnstrÃ¶m, T.; Pedrosa DomellÃ¶f, F.	2017	Autopsies
Fiber type-specific analysis of AMPK isoforms in human skeletal muscle: advancement in methods via capillary nanoimmunoassay	Tobias, I. S.; Lazauskas, K. K.; Arevalo, J. A.; Bagley, J. R.; Brown, L. E.; Galpin, A. J.	2018	Case studies
The utilization of a biopsy needle to obtain small muscle tissue specimens to analyze the gene and protein expression	Tobina, T.; Nakashima, H.; Mori, S.; Abe, M.; Kumahara, H.; Yoshimura, E.; Nishida, Y.; Kiyonaga, A.; Shono, N.; Tanaka, H.	2009	Did not perform fiber type analysis
Muscle high energy phosphates in chronic peripheral vascular disease	Todd, G. J.; Van de Wiele, B.; Askanazi, J.; Yoshikawa, K.; Elwyn, D. H.; Kinney, J. M.; Reemtsma, K.	1988	No healthy subjects or controls

Population-based study of the relationship among muscle morphology, insulin action, and hypertension	Toft, I.; BÃ¸naa, K. H.; Lindal, S.; Berg, T. J.; Jenssen, T.	1999	Does not include male and female data
Isokinetic strength, macro EMG and muscle biopsy of paretic foot dorsiflexors in chronic neurogenic paresis	TollbÃ¤ck, A.; Borg, J.; Borg, K.; Knutsson, E.	1993	No healthy subjects or controls
Torque-velocity relation and muscle fibre characteristics of foot dorsiflexors after long-term overuse of residual muscle fibres due to prior polio or L5 root lesion	TollbÃ¤ck, A.; Knutsson, E.; Borg, J.; Borg, K.; Jakobsson, F.	1992	Does not include male and female data
Substrate utilisation of cultured skeletal muscle cells in patients with CFS	Tomas, C.; Elson, J. L.; Newton, J. L.; Walker, M.	2020	Did not perform fiber type analysis
The effects of ageing and of cachexia upon skeletal muscle. A histopathological study	Tomlinson, B. E.; Walton, J. N.; Rebeiz, J. J.	1969	Autopsies
Endurance training increases stimulation of uncoupling of skeletal muscle mitochondria in humans by non-esterified fatty acids: an uncoupling-protein-mediated effect?	Tonkonogi, M.; Krook, A.; Walsh, B.; Sahlin, K.	2000	Did not perform fiber type analysis
Rate of oxidative phosphorylation in isolated mitochondria from human skeletal muscle: effect of training status	Tonkonogi, M.; Sahlin, K.	1997	Does not include males and females
Mitochondrial function and antioxidative defence in human muscle: effects of endurance training and oxidative stress	Tonkonogi, M.; Walsh, B.; Svensson, M.; Sahlin, K.	2000	Did not perform fiber type analysis
Calpain 3 deficiency affects SERCA expression and function in the skeletal muscle	Toral-Ojeda, I.; Aldanondo, G.; Lasa-Elgarresta, J.; Lasa-FernÃ¡ndez, H.; FernÃ¡ndez-TorrÃ³n, R.; LÃ³pez de Munain, A.; Vallejo-Illarramendi, A.	2016	Did not perform fiber type analysis
Inflammation and nitric oxide production in skeletal muscle of type 2 diabetic patients	Torres, S. H.; De Sanctis, J. B.; de, L. BriceÃ±o M.; HernÃ¡ndez, N.; Finol, H. J.	2004	Does not include male and female data
Capillary damage in skeletal muscle in advanced Chagas' disease patients	Torres, S. H.; Finol, H. J.; Montes de Oca, M.; VÃ¡squez, F.; PuigbÃ³, J. J.; Loyo, J. G.	2004	Does not include male and female data
Skeletal muscle myofibrillar mRNA expression in heart failure: relationship to local and circulating hormones	Toth, M. J.; Ades, P. A.; Lewinter, M. M.; Tracy, R. P.; Tchernof, A.	2006	Does not include males and females
Skeletal muscle fiber size and fiber type distribution in human cancer: Effects of weight loss and relationship to physical function	Toth, M. J.; Callahan, D. M.; Miller, M. S.; Tourville, T. W.; Hackett, S. B.; Couch, M. E.; Dittus, K.	2016	Does not include male and female data
Skeletal muscle myofibrillar protein metabolism in heart failure: relationship to immune activation and functional capacity	Toth, M. J.; Matthews, D. E.; Ades, P. A.; Tischler, M. D.; Van Buren, P.; Previs, M.; LeWinter, M. M.	2005	Does not include males and females
Molecular mechanisms underlying skeletal muscle weakness in human cancer: reduced myosin-actin cross-bridge formation and kinetics	Toth, M. J.; Miller, M. S.; Callahan, D. M.; Sweeny, A. P.; Nunez, I.; Grunberg, S. M.; Der-Torossian, H.; Couch, M. E.; Dittus, K.	2013	Does not include male and female data
Skeletal muscle mitochondrial density, gene expression, and enzyme activities in human heart failure: minimal effects of the disease and resistance training	Toth, M. J.; Miller, M. S.; Ward, K. A.; Ades, P. A.	2012	Does not include male and female data

Effect of age on skeletal muscle myofibrillar mRNA abundance: relationship to myosin heavy chain protein synthesis rate	Toth, M. J.; Tchernof, A.	2006	Does not include male and female data
Exercise in space: human skeletal muscle after 6 months aboard the International Space Station	Trappe, S.; Costill, D.; Gallagher, P.; Creer, A.; Peters, J. R.; Evans, H.; Riley, D. A.; Fitts, R. H.	2009	Does not report sex of subjects
Prostaglandin and myokine involvement in the cyclooxygenase-inhibiting drug enhancement of skeletal muscle adaptations to resistance exercise in older adults	Trappe, T. A.; Standley, R. A.; Jemiolo, B.; Carroll, C. C.; Trappe, S. W.	2013	Did not perform fiber type analysis
AS160 phosphorylation is associated with activation of alpha2beta2gamma1- but not alpha2beta2gamma3-AMPK trimeric complex in skeletal muscle during exercise in humans	Treebak, J. T.; Birk, J. B.; Rose, A. J.; Kiens, B.; Richter, E. A.; Wojtaszewski, J. F.	2007	Does not include males and females
The filtrum remodelling with combined Er:YAG CO2 laser resurfacing	Trelles, M. A.; GarcÃa-Solana, L.; Rigau, J.	1999	Does not include males and females
Impact of exercise intensity on body fatness and skeletal muscle metabolism	Tremblay, A.; Simoneau, J. A.; Bouchard, C.	1994	Did not perform fiber type analysis
Effect of GH on human skeletal muscle lipid metabolism in GH deficiency	Trepp, R.; FlÃ¼ck, M.; Stettler, C.; Boesch, C.; Ith, M.; Kreis, R.; Hoppeler, H.; Howald, H.; Schmid, J. P.; Diem, P.; Christ, E. R.	2008	Does not include male and female data
The influence of myosin heavy chain isoform content on mechanical behavior of the vastus lateralis in vivo	Trevino, M. A.; Herda, T. J.; Fry, A. C.; Gallagher, P. M.; Vardiman, J. P.; Mosier, E. M.; Miller, J. D.	2016	No cross-sectional area data
Influence of the contractile properties of muscle on motor unit firing rates during a moderate-intensity contraction in vivo	Trevino, M. A.; Herda, T. J.; Fry, A. C.; Gallagher, P. M.; Vardiman, J. P.; Mosier, E. M.; Miller, J. D.	2016	Does not include male and female data
Loss of mitochondrial energetics is associated with poor recovery of muscle function but not mass following disuse atrophy	Trevino, M. B.; Zhang, X.; Standley, R. A.; Wang, M.; Han, X.; Reis, F. C. G.; Periasamy, M.; Yu, G.; Kelly, D. P.; Goodpaster, B. H.; Vega, R. B.; Coen, P. M.	2019	Does not report sex of subjects
Effect of N-acetylcysteine infusion on exercise-induced modulation of insulin sensitivity and signaling pathways in human skeletal muscle	Trewin, A. J.; Lundell, L. S.; Perry, B. D.; Patil, K. V.; Chibalin, A. V.; Levinger, I.; McQuade, L. R.; Stepto, N. K.	2015	Does not include males and females
Acute HIIE elicits similar changes in human skeletal muscle mitochondrial H(2)O(2) release, respiration, and cell signaling as endurance exercise even with less work	Trewin, A. J.; Parker, L.; Shaw, C. S.; Hiam, D. S.; Garnham, A.; Levinger, I.; McConell, G. K.; Stepto, N. K.	2018	Did not perform fiber type analysis
Resistance training prevents deterioration in quadriceps muscle function during acute exacerbations of chronic obstructive pulmonary disease	Troosters, T.; Probst, V. S.; Crul, T.; Pitta, F.; Gayan-Ramirez, G.; Decramer, M.; Gosselink, R.	2010	No healthy subjects or controls
Chronic alcoholic proximal wasting: physiological, morphological and biochemical studies in skeletal muscle	Trounce, I.; Byrne, E.; Dennett, X.; Santamaria, J.; Doery, J.; Peppard, R.	1987	Does not report sex of subjects
Metabolic and molecular responses to leucine-enriched branched chain amino acid supplementation in the skeletal muscle of alcoholic cirrhosis	Tsien, C.; Davuluri, G.; Singh, D.; Allawy, A.; Ten Have, G. A.; Thapaliya, S.; Schulze, J. M.; Barnes, D.; McCullough, A. J.; Engelen, M. P.; Deutz, N. E.; Dasarathy, S.	2015	Did not perform fiber type analysis

Independent and combined effects of acute physiological hyperglycaemia and hyperinsulinaemia on metabolic gene expression in human skeletal muscle	Tsintzas, K.; Norton, L.; Chokkalingam, K.; Nizamani, N.; Cooper, S.; Stephens, F.; Billeter, R.; Bennett, A.	2013	Does not include males and females
Potential role of endothelin 1 in ischaemia-induced angiogenesis in critical leg ischaemia	Tsui, J. C.; Baker, D. M.; Biecker, E.; Shaw, S.; Dashwood, M. R.	2002	No healthy subjects or controls
Semiquantitative measurement of acetylcholine receptor at the motor end-plate in myasthenia gravis	Tsujihata, M.; Ito, H.; Satoh, A.; Yoshimura, T.; Motomura, M.; Nakamura, T.	2001	Did not perform fiber type analysis
Early de novo DNA methylation and prolonged demethylation in the muscle lineage	Tsumagari, K.; Baribault, C.; Terragni, J.; Varley, K. E.; Gertz, J.; Pradhan, S.; Badoo, M.; Crain, C. M.; Song, L.; Crawford, G. E.; Myers, R. M.; Lacey, M.; Ehrlich, M.	2013	Did not perform fiber type analysis
Quantitation of argyrophilic nucleolar organizer regions in regenerating muscle fibers in Duchenne and Becker muscular dystrophies and polymyositis	Tuccari, G.; GiuffrÃ¨, G.; Crisafulli, C.; Monici, M. C.; Toscano, A.; Vita, G.	1999	No healthy subjects or controls
Skeletal muscle transcriptome in healthy aging	Tumasian, R. A., 3rd; Harish, A.; Kundu, G.; Yang, J. H.; Ubaida-Mohien, C.; Gonzalez-Freire, M.; Kaileh, M.; Zukley, L. M.; Chia, C. W.; Lyashkov, A.; Wood, W. H., 3rd; Piao, Y.; Coletta, C.; Ding, J.; Gorospe, M.; Sen, R.; De, S.; Ferrucci, L.	2021	Did not perform fiber type analysis
HIF-VEGF-VEGFR-2, TNF-alpha and IGF pathways are upregulated in critical human skeletal muscle ischemia as studied with DNA array	Tuomisto, T. T.; Rissanen, T. T.; Vajanto, I.; Korkeela, A.; Rutanen, J.; YlÃ¤-Herttuala, S.	2004	No healthy subjects or controls
Functional muscle impairment in facioscapulohumeral muscular dystrophy is correlated with oxidative stress and mitochondrial dysfunction	Turki, A.; Hayot, M.; Carnac, G.; Pillard, F.; Passerieux, E.; Bommart, S.; Raynaud de Mauverger, E.; Hugon, G.; Pincemail, J.; Pietri, S.; Lambert, K.; Belayew, A.; Vassetzky, Y.; Juntas Morales, R.; Mercier, J.; Laoudj-Chenivesse, D.	2012	Does not include male and female data
Clinical correlates of mitochondrial function in Huntington's disease muscle	Turner, C.; Cooper, J. M.; Schapira, A. H.	2007	Did not perform fiber type analysis
Discovery proteomics in aging human skeletal muscle finds change in spliceosome, immunity, proteostasis and mitochondria	Ubaida-Mohien, C.; Lyashkov, A.; Gonzalez-Freire, M.; Tharakan, R.; Shardell, M.; Moaddel, R.; Semba, R. D.; Chia, C. W.; Gorospe, M.; Sen, R.; Ferrucci, L.	2019	Does not include male and female data
Localization and characterization of dystrophin in muscle biopsy specimens from Duchenne muscular dystrophy and various neuromuscular disorders	Uchino, M.; Araki, S.; Miike, T.; Teramoto, H.; Nakamura, T.; Yasutake, T.	1989	Does not report sex of subjects
Expression of three calpain isoform genes in human skeletal muscles	Ueyama, H.; Kumamoto, T.; Fujimoto, S.; Murakami, T.; Tsuda, T.	1998	Did not perform fiber type analysis
Down-regulation of the aberrant expression of the inflammation mediator high mobility group box chromosomal protein 1 in muscle tissue of patients with polymyositis and dermatomyositis treated with corticosteroids	Ulfgren, A. K.; Grundtman, C.; Borg, K.; Alexanderson, H.; Andersson, U.; Harris, H. E.; Lundberg, I. E.	2004	Did not perform fiber type analysis

The greater risk of alcoholic cardiomyopathy and myopathy in women compared with men	Urbano-MÃ¡rquez, A.; Estruch, R.; FernÃ¡ndez-SolÃ¡, J.; NicolÃ¡s, J. M.; ParÃ©, J. C.; Rubin, E.	1995	Does not include males and females
Cytoplasmic creatine kinase isoenzymes quantitated in tissue specimens obtained at surgery	Urdal, P.; Urdal, K.; StrÃ¸mme, J. H.	1983	Did not perform fiber type analysis
Immunogold labeling of insulin growth factor-I receptors in elderly human skeletal muscle	Urso, M.; Cosmas, A.; Singh, M. F.; Manfredi, T.	2005	Did not perform fiber type analysis
Carbohydrate supplementation and perceived exertion during prolonged running	Utter, A. C.; Kang, J.; Nieman, D. C.; Dumke, C. L.; McAnulty, S. R.; Vinci, D. M.; McAnulty, L. S.	2004	Did not perform fiber type analysis
Hyperglycaemia compensates for the defects in insulin-mediated glucose metabolism and in the activation of glycogen synthase in the skeletal muscle of patients with type 2 (non-insulin-dependent) diabetes mellitus	Vaag, A.; Damsbo, P.; Hother-Nielsen, O.; Beck-Nielsen, H.	1992	Did not perform fiber type analysis
Skeletal muscle of patients with gyrate atrophy of the choroid and retina and hyperornithinaemia in ultralow-field magnetic resonance imaging and computed tomography	Valtonen, M.; NÃ¤ntÃ¶-Salonen, K.; HeinÃ¤nen, K.; Alanen, A.; Kalimo, H.; Simell, O.	1996	No healthy subjects or controls
Validation of an adiponectin immunoassay in human skeletal muscle biopsies	Van Berendoncks, A. M.; Conraads, V. M.; Van Leuven, W.; Van Hoof, V.; De Wilde, S.; Vrints, C. J.; Hoymans, V. Y.	2010	Does not include males and females
Functional adiponectin resistance at the level of the skeletal muscle in mild to moderate chronic heart failure	Van Berendoncks, A. M.; Garnier, A.; Beckers, P.; Hoymans, V. Y.; Possemiers, N.; Fortin, D.; Martinet, W.; Van Hoof, V.; Vrints, C. J.; Ventura-Clapier, R.; Conraads, V. M.	2010	Did not perform fiber type analysis
Identification of microRNAs in skeletal muscle associated with lung cancer cachexia	van de Worp, Wrph; Schols, Amwj; Dingemans, A. C.; Op den Kamp, C. M. H.; Degens, Jhrj; Kelders, Mcjm; Coort, S.; Woodruff, H. C.; Kratassiouk, G.; Harel-Bellan, A.; Theys, J.; van Helvoort, A.; Langen, R. C. J.	2020	Did not perform fiber type analysis
Loss of quadriceps muscle oxidative phenotype and decreased endurance in patients with mild-to-moderate COPD	van den Borst, B.; Slot, I. G.; Hellwig, V. A.; Vosse, B. A.; Kelders, M. C.; Barreiro, E.; Schols, A. M.; Gosker, H. R.	2013	Does not include male and female data
The functional, metabolic, and anabolic responses to exercise training in renal transplant and hemodialysis patients	van den Ham, E. C.; Kooman, J. P.; Schols, A. M.; Nieman, F. H.; Does, J. D.; Akkermans, M. A.; Janssen, P. P.; Gosker, H. R.; Ward, K. A.; MacDonald, J. H.; Christiaans, M. H.; Leunissen, K. M.; van Hooff, J. P.	2007	Does not include male and female data
Involvement of pelvic girdle and proximal leg muscles in early oculopharyngeal muscular dystrophy	van der Sluijs, B. M.; Lassche, S.; Knuiman, G. J.; Kusters, B.; Heerschap, A.; Hopman, M.; Schreuder, T. H.; van Engelen, B. G. M.; Voermans, N. C.	2017	Did not perform fiber type analysis
Adaptations in muscle oxidative capacity, fiber size, and oxygen supply capacity after repeated-sprint training in hypoxia combined with chronic hypoxic exposure	van der Zwaard, S.; Brocherie, F.; Kom, B. L. G.; Millet, G. P.; Deldicque, L.; van der Laarse, W. J.; Girard, O.; Jaspers, R. T.	2018	Does not include males and females

Maximal oxygen uptake is proportional to muscle fiber oxidative capacity, from chronic heart failure patients to professional cyclists	van der Zwaard, S.; de Ruiter, C. J.; Noordhof, D. A.; Sterrenburg, R.; Bloemers, F. W.; de Koning, J. J.; Jaspers, R. T.; van der Laarse, W. J.	2016	Does not include male and female data
Critical determinants of combined sprint and endurance performance: an integrative analysis from muscle fiber to the human body	van der Zwaard, S.; van der Laarse, W. J.; Weide, G.; Bloemers, F. W.; Hofmijster, M. J.; Levels, K.; Noordhof, D. A.; de Koning, J. J.; de Ruiter, C. J.; Jaspers, R. T.	2018	Does not report sex of subjects
Termination of damaged protein repair defines the occurrence of symptoms in carriers of the m.3243A > G tRNA(Leu) mutation	van Eijsden, R. G.; Eijssen, L. M.; Lindsey, P. J.; van den Burg, C. M.; de Wit, L. E.; Rubio-Gozalbo, M. E.; de Die, C. E.; Ayoubi, T.; Sluiter, W.; de Coo, I. F.; Smeets, H. J.	2008	Did not perform fiber type analysis
Adjustments of muscle capillarity but not mitochondrial protein with skiing in the elderly	van Ginkel, S.; Amami, M.; Dela, F.; Niederseer, D.; Narici, M. V.; Niebauer, J.; Scheiber, P.; MÃ¼ller, E.; FlÃ¼ck, M.	2015	Does not include male and female data
Cytochrome c oxidase activity and fatty acid oxidation in various types of human muscle	Van Hinsbergh, V. W.; Veerkamp, J. H.; Van Moerkerk, H. T.	1980	No healthy subjects or controls
Delving into disability in Crohn's disease: dysregulation of molecular pathways may explain skeletal muscle loss in Crohn's disease	van Langenberg, D. R.; Della Gatta, P.; Hill, B.; Zacharewicz, E.; Gibson, P. R.; Russell, A. P.	2014	Did not perform fiber type analysis
The effects of increasing exercise intensity on muscle fuel utilisation in humans	van Loon, L. J.; Greenhaff, P. L.; Constantin-Teodosiu, D.; Saris, W. H.; Wagenmakers, A. J.	2001	Does not include males and females
Creatine supplementation increases glycogen storage but not GLUT-4 expression in human skeletal muscle	van Loon, L. J.; Murphy, R.; Oosterlaar, A. M.; Cameron-Smith, D.; Hargreaves, M.; Wagenmakers, A. J.; Snow, R.	2004	Does not include males and females
Inhibition of adipose tissue lipolysis increases intramuscular lipid and glycogen use in vivo in humans	van Loon, L. J.; Thomason-Hughes, M.; Constantin-Teodosiu, D.; Koopman, R.; Greenhaff, P. L.; Hardie, D. G.; Keizer, H. A.; Saris, W. H.; Wagenmakers, A. J.	2005	Does not include males and females
Mitochondrial Respiration after One Session of Calf Raise Exercise in Patients with Peripheral Vascular Disease and Healthy Older Adults	van Schaardenburgh, M.; Wohlwend, M.; Rognmo, Ã˜; Mattsson, E. J.	2016	Did not perform fiber type analysis
Calcinosis in juvenile dermatomyositis: a possible role for the vitamin K-dependent protein matrix Gla protein	van Summeren, M. J.; Spliet, W. G.; van Royen-Kerkhof, A.; Vermeer, C.; Lilien, M.; Kuis, W.; Schurgers, L. J.	2008	Does not report sex of subjects
No independent or combined effects of vitamin D and conjugated linoleic acids on muscle protein synthesis in older adults: a randomized, double-blind, placebo-controlled clinical trial	van Vliet, S.; Fappi, A.; Reeds, D. N.; Mittendorfer, B.	2020	Did not perform fiber type analysis
Rare muscle diseases	van Wijngaarden, G. K.	1972	Case studies
Assessing dystrophies and other muscle diseases at the nanometer scale by atomic force microscopy	van Zwieten, R. W.; Puttini, S.; Lekka, M.; Witz, G.; Gicquel-Zouida, E.; Richard, I.; Lobrinus, J. A.; Chevalley, F.; Brune, H.; Dietler, G.; Kulik, A.; Kuntzer, T.; Mermod, N.	2014	Did not perform fiber type analysis
Identification of the IGF-1 processing product human Ec/rodent Eb peptide in various tissues: Evidence for its differential regulation after exercise-induced muscle damage in humans	Vassilakos, G.; Philippou, A.; Koutsilieris, M.	2017	Does not include males and females

Overexpression of TNF-Î± in mitochondrial diseases caused by mutations in mtDNA: evidence for signaling through its receptors on mitochondria	Vattemi, G.; Marini, M.; Ferreri, N. R.; Hao, S.; Malatesta, M.; Meneguzzi, A.; Guglielmi, V.; Fava, C.; Minuz, P.; Tomelleri, G.	2013	Does not report sex of subjects
Increased protein nitration in mitochondrial diseases: evidence for vessel wall involvement	Vattemi, G.; Mechref, Y.; Marini, M.; Tonin, P.; Minuz, P.; Grigoli, L.; Guglielmi, V.; Klouckova, I.; Chiamulera, C.; Meneguzzi, A.; Di Chio, M.; Tedesco, V.; Lovato, L.; Degan, M.; Arcaro, G.; Lechi, A.; Novotny, M. V.; Tomelleri, G.	2011	Did not perform fiber type analysis
Resistance exercise increases NF-Î°B activity in human skeletal muscle	Vella, L.; Caldow, M. K.; Larsen, A. E.; Tassoni, D.; Della Gatta, P. A.; Gran, P.; Russell, A. P.; Cameron-Smith, D.	2012	Did not perform fiber type analysis
PKM2 Determines Myofiber Hypertrophy In Vitro and Increases in Response to Resistance Exercise in Human Skeletal Muscle	Verbrugge, S. A. J.; Gehlert, S.; Stadhouders, L. E. M.; Jacko, D.; Aussieker, T.; M. J. de Wit G; Vogel, I. S. P.; Offringa, C.; SchÃ¶nfelder, M.; Jaspers, R. T.; Wackerhage, H.	2020	Does not include males and females
Alterations in activin A-myostatin-follistatin system associate with disease activity in inflammatory myopathies	VernerovÃ¡, L.; HorvÃ¡thovÃ¡, V.; KropÃ¡ÄkovÃ¡, T.; VokurkovÃ¡, M.; Klein, M.; TomÄÃk, M.; OreskÃ¡, S.; Å piritoviÄ‡, M.; Å torkÃ¡novÃ¡, H.; HeÅ™mÃ¡nkovÃ¡, B.; KubÃnovÃ¡, K.; KryÅ¡tÅ¯fkovÃ¡, O.; Mann, H.; Ukropec, J.; UkropcovÃ¡, B.; VencovskÃ½, J.	2020	Did not perform fiber type analysis
Skeletal muscle myofibrillar protein oxidation and exercise capacity in heart failure	Vescovo, G.; Ravara, B.; Dalla Libera, L.	2008	Does not include males and females
Apoptosis in the skeletal muscle of patients with heart failure: investigation of clinical and biochemical changes	Vescovo, G.; Volterrani, M.; Zennaro, R.; Sandri, M.; Ceconi, C.; Lorusso, R.; Ferrari, R.; Ambrosio, G. B.; Dalla Libera, L.	2000	Does not include males and females
Impaired expression of glycogen synthase mRNA in skeletal muscle of NIDDM patients	Vestergaard, H.; BjÃ¸rbaek, C.; Andersen, P. H.; Bak, J. F.; Pedersen, O.	1991	Did not perform fiber type analysis
Impaired activity and gene expression of hexokinase II in muscle from non-insulin-dependent diabetes mellitus patients	Vestergaard, H.; BjÃ¸rbaek, C.; Hansen, T.; Larsen, F. S.; Granner, D. K.; Pedersen, O.	1995	Did not perform fiber type analysis
Glycogen synthase and phosphofructokinase protein and mRNA levels in skeletal muscle from insulin-resistant patients with non-insulin-dependent diabetes mellitus	Vestergaard, H.; Lund, S.; Larsen, F. S.; Bjerrum, O. J.; Pedersen, O.	1993	Did not perform fiber type analysis
Insulin-resistant glucose metabolism in patients with microvascular angina--syndrome X	Vestergaard, H.; SkÃ¸tt, P.; Steffensen, R.; Wroblewski, H.; Pedersen, O.; Kastrup, J.	1995	Did not perform fiber type analysis
Visualization of defective mitochondrial function in skeletal muscle fibers of patients with sporadic amyotrophic lateral sclerosis	Vielhaber, S.; Winkler, K.; Kirches, E.; Kunz, D.; BÃ¼chner, M.; Feistner, H.; Elger, C. E.; Ludolph, A. C.; Riepe, M. W.; Kunz, W. S.	1999	Did not perform fiber type analysis
Proteins modulation in human skeletal muscle in the early phase of adaptation to hypobaric hypoxia	ViganÃ², A.; Ripamonti, M.; De Palma, S.; Capitanio, D.; Vasso, M.; Wait, R.; Lundby, C.; Cerretelli, P.; Gelfi, C.	2008	Does not include males and females

Diagnostic anoctamin-5 protein defect in patients with ANO5-mutated muscular dystrophy	Vihola, A.; Luque, H.; Savarese, M.; PenttilÃ¤, S.; Lindfors, M.; Leturcq, F.; Eymard, B.; Tasca, G.; Brais, B.; Conte, T.; Charton, K.; Richard, I.; Udd, B.	2018	Does not report sex of subjects
Interrelationships between electromyographic, mechanical, muscle structure and reflex time measurements in man	Viitasalo, J. T.; Komi, P. V.	1981	Does not include males and females
Impaired skeletal muscle mitochondrial function in morbidly obese patients is normalized one year after bariatric surgery	Vijgen, G. H.; Bouvy, N. D.; Hoeks, J.; Wijers, S.; Schrauwen, P.; van Marken Lichtenbelt, W. D.	2013	Does not include males and females
Myopathy in patients with Hashimoto's disease	Villar, J.; Finol, H. J.; Torres, S. H.; Roschman-GonzÃ¡lez, A.	2015	No cross-sectional area data
Mitochondrial dysfunction in myofibrillar myopathy	Vincent, A. E.; Grady, J. P.; Rocha, M. C.; Alston, C. L.; Rygiel, K. A.; Barresi, R.; Taylor, R. W.; Turnbull, D. M.	2016	No healthy subjects or controls
Dysferlin mutations and mitochondrial dysfunction	Vincent, A. E.; Rosa, H. S.; Alston, C. L.; Grady, J. P.; Rygiel, K. A.; Rocha, M. C.; Barresi, R.; Taylor, R. W.; Turnbull, D. M.	2016	Did not perform fiber type analysis
Remodeling of skeletal muscle microvasculature in sickle cell trait and alpha-thalassemia	Vincent, L.; FÃ©asson, L.; Oyono-EnguÃ©llÃ©, S.; Banimbek, V.; Denis, C.; Guarneri, C.; Aufradet, E.; Monchanin, G.; Martin, C.; Gozal, D.; Dohbobga, M.; Wouassi, D.; Garet, M.; Thiriet, P.; Messonnier, L.	2010	Does not include males and females
Skeletal muscle structural and energetic characteristics in subjects with sickle cell trait, alpha-thalassemia, or dual hemoglobinopathy	Vincent, L.; FÃ©asson, L.; Oyono-EnguÃ©llÃ©, S.; Banimbek, V.; Monchanin, G.; Dohbobga, M.; Wouassi, D.; Martin, C.; Gozal, D.; Geyssant, A.; Thiriet, P.; Denis, C.; Messonnier, L.	2010	Does not include males and females
Effects of regular physical activity on skeletal muscle structural, energetic, and microvascular properties in carriers of sickle cell trait	Vincent, L.; Oyono-EnguÃ©llÃ©, S.; FÃ©asson, L.; Banimbek, V.; Dohbobga, M.; Martin, C.; Thiriet, P.; Francina, A.; Dubouchaud, H.; Sanchez, H.; Chapot, R.; Denis, C.; Geyssant, A.; Messonnier, L.	2012	Does not include males and females
Hyperglycaemia normalises insulin action on glucose metabolism but not the impaired activation of AKT and glycogen synthase in the skeletal muscle of patients with type 2 diabetes	Vind, B. F.; Birk, J. B.; Vienberg, S. G.; Andersen, B.; Beck-Nielsen, H.; Wojtaszewski, J. F.; HÃ¸jlund, K.	2012	Did not perform fiber type analysis
Impaired insulin-induced site-specific phosphorylation of TBC1 domain family, member 4 (TBC1D4) in skeletal muscle of type 2 diabetes patients is restored by endurance exercise-training	Vind, B. F.; PehmÃ¸ller, C.; Treebak, J. T.; Birk, J. B.; Hey-Mogensen, M.; Beck-Nielsen, H.; Zierath, J. R.; Wojtaszewski, J. F.; HÃ¸jlund, K.	2011	Does not include males and females
Effect of transcutaneous electrical muscle stimulation on postoperative muscle mass and protein synthesis	Vinge, O.; Edvardsen, L.; Jensen, F.; Jensen, F. G.; Wernerman, J.; Kehlet, H.	1996	No healthy subjects or controls
Effect of resistance exercise on muscle steroid receptor protein content in strength-trained men and women	Vingren, J. L.; Kraemer, W. J.; Hatfield, D. L.; Volek, J. S.; Ratamess, N. A.; Anderson, J. M.; HÃ¤kkinen, K.; Ahtiainen, J.; Fragala, M. S.; Thomas, G. A.; Ho, J. Y.; Maresh, C. M.	2009	Did not perform fiber type analysis

Are exercise-induced genes induced by exercise?	Vissing, K.; Andersen, J. L.; Schjerling, P.	2005	Does not include males and females
Effects of concentric and repeated eccentric exercise on muscle damage and calpain-calpastatin gene expression in human skeletal muscle	Vissing, K.; Overgaard, K.; Nedergaard, A.; Fredsted, A.; Schjerling, P.	2008	Does not include males and females
Sarcolemmal FAT/CD36 in human skeletal muscle colocalizes with caveolin-3 and is more abundant in type 1 than in type 2 fibers	Vistisen, B.; Roepstorff, K.; Roepstorff, C.; Bonen, A.; van Deurs, B.; Kiens, B.	2004	Does not include males and females
Myofiber stress-response in myositis: parallel investigations on patients and experimental animal models of muscle regeneration and systemic inflammation	Vitadello, M.; Doria, A.; Tarricone, E.; Ghirardello, A.; Gorza, L.	2010	Does not include males and females
Differential diagnosis of vacuolar muscle biopsies: use of p62, LC3 and LAMP2 immunohistochemistry	Vittonatto, E.; Boschi, S.; C. HIADÃ²-Piat L; Ponzalino, V.; Bortolani, S.; Brusa, C.; Rainero, I.; Ricci, F.; Vercelli, L.; Mongini, T.	2017	Did not perform fiber type analysis
Evaluation of muscle capillary basement membrane in inflammatory myopathy. A morphometric ultrastructural study	Vlodavsky, E. A.; Ludatscher, R. M.; Sabo, E.; Kerner, H.	1999	Did not perform fiber type analysis
Dysregulation of Circular RNAs in Myotonic Dystrophy Type 1	Voellenkle, C.; Perfetti, A.; Carrara, M.; Fuschi, P.; Renna, L. V.; Longo, M.; Sain, S. B.; Cardani, R.; Valaperta, R.; Silvestri, G.; Legnini, I.; Bozzoni, I.; Furling, D.; Gaetano, C.; Falcone, G.; Meola, G.; Martelli, F.	2019	Did not perform fiber type analysis
Regulation of hexokinase II expression in human skeletal muscle in vivo	Vogt, C.; Ardehali, H.; Iozzo, P.; Yki-JÃ¤rvinen, H.; Koval, J.; Maezono, K.; Pendergrass, M.; Printz, R.; Granner, D.; DeFronzo, R.; Mandarino, L.	2000	Did not perform fiber type analysis
Effects of insulin on subcellular localization of hexokinase II in human skeletal muscle in vivo	Vogt, C.; Yki-Jarvinen, H.; Iozzo, P.; Pipek, R.; Pendergrass, M.; Koval, J.; Ardehali, H.; Printz, R.; Granner, D.; Defronzo, R.; Mandarino, L.	1998	Did not perform fiber type analysis
Molecular adaptations in human skeletal muscle to endurance training under simulated hypoxic conditions	Vogt, M.; Puntschart, A.; Geiser, J.; Zuleger, C.; Billeter, R.; Hoppeler, H.	2001	Does not include males and females
Resynthesis of glycogen in different muscle fibre types after prolonged exhaustive exercise in man	VÃ¸llestad, N. K.; Blom, P. C.; GrÃ¸nnerÃ¸d, O.	1989	Does not include males and females
Glycogen breakdown in different human muscle fibre types during exhaustive exercise of short duration	VÃ¸llestad, N. K.; Tabata, I.; MedbÃ¸, J. I.	1992	Does not include males and females
Muscle glycogen depletion patterns in type I and subgroups of type II fibres during prolonged severe exercise in man	VÃ¸llestad, N. K.; Vaage, O.; Hermansen, L.	1984	No cross-sectional area data
Sequential muscle biopsies during a 6-h tracer infusion do not affect human mixed muscle protein synthesis and muscle phenylalanine kinetics	Volpi, E.; Chinkes, D. L.; Rasmussen, B. B.	2008	Did not perform fiber type analysis
Antiangiogenic VEGF isoform in inflammatory myopathies	Volpi, N.; Pecorelli, A.; Lorenzoni, P.; Di Lazzaro, F.; Belmonte, G.; AglianÃ², M.;	2013	Did not perform fiber type analysis

	Cantarini, L.; Giannini, F.; Grasso, G.; Valacchi, G.		
Overview article: basal lamina of epidermis, muscle fibers, muscle capillaries, and renal tubules: changes with aging and in diabetes mellitus	Vracko, R.; Pecoraro, R. E.; Carter, W. B.	1980	Autopsies
Basal lamina of abdominal skeletal muscle capillaries in diabetics and nondiabetics	Vracko, R.; Strandness, D. E., Jr.	1967	Does not include males and females
Carnitine supplementation: effect on muscle carnitine and glycogen content during exercise	Vukovich, M. D.; Costill, D. L.; Fink, W. J.	1994	Does not include males and females
The effect of excessive weight loss on skeletal muscle in man. A study of obese patients following gastroplasty	WadstrÃ¶m, C.; Larsson, L.; Knutsson, E.; EdstrÃ¶m, L.	1991	No healthy subjects or controls
Electron microscopic study on the satellite cell in the muscle of Duchenne muscular dystrophy	Wakayama, Y.	1976	No healthy subjects or controls
Altered alpha1-syntrophin expression in myofibers with Duchenne and Fukuyama muscular dystrophies	Wakayama, Y.; Inoue, M.; Kojima, H.; Jimi, T.; Yamashita, S.; Kumagai, T.; Shibuya, S.; Hara, H.; Oniki, H.	2006	Does not include male and female data
Reduced expression of sarcospan in muscles of Fukuyama congenital muscular dystrophy	Wakayama, Y.; Inoue, M.; Kojima, H.; Yamashita, S.; Shibuya, S.; Jimi, T.; Hara, H.; Matsuzaki, Y.; Oniki, H.; Kanagawa, M.; Kobayashi, K.; Toda, T.	2008	Does not include male and female data
Dystrophin immunostaining and freeze-fracture studies of muscles of patients with early stage amyotrophic lateral sclerosis and Duchenne muscular dystrophy	Wakayama, Y.; Jimi, T.; Misugi, N.; Kumagai, T.; Miyake, S.; Shibuya, S.; Miike, T.	1989	Did not perform fiber type analysis
Dysbindin, syncoilin, and beta-synemin mRNA levels in dystrophic muscles	Wakayama, Y.; Matsuzaki, Y.; Yamashita, S.; Inoue, M.; Jimi, T.; Hara, H.; Unaki, A.; Iijima, S.; Masaki, H.	2010	Did not perform fiber type analysis
Angina pectoris patients with normal coronary angiograms but abnormal thallium perfusion scan exhibit low myocardial and skeletal muscle energy charge	WaldenstrÃ¶m, A.; Ronquist, G.; Lagerqvist, B.	1992	Did not perform fiber type analysis
Vasoactive enzymes and blood flow responses to passive and active exercise in peripheral arterial disease	Walker, M. A.; Hoier, B.; Walker, P. J.; Schulze, K.; Bangsbo, J.; Hellsten, Y.; Askew, C. D.	2016	Did not perform fiber type analysis
Histochemical and metabolic changes in lower leg muscles in exercise-induced pain	Wallensten, R.; Karlsson, J.	1984	Does not include male and female data
Alpha-actinin and myosin light chains in congenital nemaline myopathy	Wallgren-Pettersson, C.; Arjomaa, P.; Holmberg, C.	1990	Did not perform fiber type analysis
Effect of endurance training on oxidative and antioxidative function in human permeabilized muscle fibres	Walsh, B.; Tonkonogi, M.; Sahlin, K.	2001	Did not perform fiber type analysis
Expression of cell adhesion molecule, N-CAM, in diseases of adult human skeletal muscle	Walsh, F. S.; Moore, S. E.	1985	Does not include male and female data

Human skeletal muscle macrophages increase following cycle training and are associated with adaptations that may facilitate growth	Walton, R. G.; Kosmac, K.; Mula, J.; Fry, C. S.; Peck, B. D.; Groshong, J. S.; Finlin, B. S.; Zhu, B.; Kern, P. A.; Peterson, C. A.	2019	Did not perform fiber type analysis
The effect of an amino acid beverage on glucose response and glycogen replenishment after strenuous exercise	Wang, B.; Ding, Z.; Wang, W.; Hwang, J.; Liao, Y. H.; Ivy, J. L.	2015	Did not perform fiber type analysis
Acute effects of different diet compositions on skeletal muscle insulin signalling in obese individuals during caloric restriction	Wang, C. C.; Adochio, R. L.; Leitner, J. W.; Abeyta, I. M.; Draznin, B.; Cornier, M. A.	2013	Did not perform fiber type analysis
Transcriptome alterations in myotonic dystrophy skeletal muscle and heart	Wang, E. T.; Treacy, D.; Eichinger, K.; Struck, A.; Estabrook, J.; Olafson, H.; Wang, T. T.; Bhatt, K.; Westbrook, T.; Sedehizadeh, S.; Ward, A.; Day, J.; Brook, D.; Berglund, J. A.; Cooper, T.; Housman, D.; Thornton, C.; Burge, C.	2019	Did not perform fiber type analysis
Relationships between muscle mitochondrial DNA content, mitochondrial enzyme activity and oxidative capacity in man: alterations with disease	Wang, H.; Hiatt, W. R.; Barstow, T. J.; Brass, E. P.	1999	Does not include males and females
Muscle mass loss and intermuscular lipid accumulation were associated with insulin resistance in patients receiving hemodialysis	Wang, H. L.; Ding, T. T.; Lu, S.; Xu, Y.; Tian, J.; Hu, W. F.; Zhang, J. Y.	2013	Did not perform fiber type analysis
Resistance exercise enhances the molecular signaling of mitochondrial biogenesis induced by endurance exercise in human skeletal muscle	Wang, L.; Mascher, H.; Psilander, N.; Blomstrand, E.; Sahlin, K.	2011	Did not perform fiber type analysis
Similar expression of oxidative genes after interval and continuous exercise	Wang, L.; Psilander, N.; Tonkonogi, M.; Ding, S.; Sahlin, K.	2009	Did not perform fiber type analysis
The effect of continuous and interval exercise on PGC-1Î± and PDK4 mRNA in type I and type II fibres of human skeletal muscle	Wang, L.; Sahlin, K.	2012	Does not include males and females
MRI-informed muscle biopsies correlate MRI with pathology and DUX4 target gene expression in FSHD	Wang, L. H.; Friedman, S. D.; Shaw, D.; Snider, L.; Wong, C. J.; Budech, C. B.; Poliachik, S. L.; Gove, N. E.; Lewis, L. M.; Campbell, A. E.; Lemmers, Rjfl; Maarel, S. M.; Tapscott, S. J.; Tawil, R. N.	2019	Did not perform fiber type analysis
Nitric oxide in skeletal muscle: inhibition of nitric oxide synthase inhibits walking speed in rats	Wang, M. X.; Murrell, D. F.; Szabo, C.; Warren, R. F.; Sarris, M.; Murrell, G. A.	2001	No healthy subjects or controls
Endothelial interleukin-21 receptor up-regulation in peripheral artery disease	Wang, T.; Cunningham, A.; Houston, K.; Sharma, A. M.; Chen, L.; Dokun, A. O.; Lye, R. J.; Spolski, R.; Leonard, W. J.; Annex, B. H.	2016	Did not perform fiber type analysis
Skeletal muscle oxidative capacity, fiber type, and metabolites after lung transplantation	Wang, X. N.; Williams, T. J.; McKenna, M. J.; Li, J. L.; Fraser, S. F.; Side, E. A.; Snell, G. I.; Walters, E. H.; Carey, M. F.	1999	Does not include male and female data
Muscle-specific mutations accumulate with aging in critical human mtDNA control sites for replication	Wang, Y.; Michikawa, Y.; Mallidis, C.; Bai, Y.; Woodhouse, L.; Yarasheski, K. E.; Miller, C. A.; Askanas, V.; Engel, W. K.; Bhasin, S.; Attardi, G.	2001	Autopsies

Cisplatin inhibits the proliferation of Saos-2 osteosarcoma cells via the miR-376c/TGFA pathway	Wang, Y.; Wu, Y.; Cai, A.; Ma, C.; Cai, S.; Wang, H.; Que, Y.; Xu, S.; Xu, T.; Hu, Y.	2021	Did not perform fiber type analysis
Expression of myogenic regulatory factors and myo-endothelial remodeling in sporadic inclusion body myositis	Wanschitz, J. V.; Dubourg, O.; Lacene, E.; Fischer, M. B.; HÃ¶ftberger, R.; Budka, H.; Romero, N. B.; Eymard, B.; Herson, S.; Butler-Browne, G. S.; Voit, T.; Benveniste, O.	2013	Did not perform fiber type analysis
Evidence for susceptibility to malignant hyperthermia in patients with exercise-induced rhabdomyolysis	Wappler, F.; Fiege, M.; Steinfath, M.; Agarwal, K.; Scholz, J.; Singh, S.; Matschke, J.; Schulte Am Esch, J.	2001	No healthy subjects or controls
Ritanserin attenuates the in vitro effects of the 5-HT2 receptor agonist DOI on skeletal muscles from malignant hyperthermia-susceptible patients	Wappler, F.; Scholz, J.; Oppermann, S.; von Richthofen, V.; Steinfath, M.; Schulte am Esch, J.	1997	No healthy subjects or controls
Activation by exercise of human skeletal muscle pyruvate dehydrogenase in vivo	Ward, G. R.; Sutton, J. R.; Jones, N. L.; Toews, C. J.	1982	Does not include males and females
The antioxidant status of patients with either alcohol-induced liver damage or myopathy	Ward, R. J.; Peters, T. J.	1992	No healthy subjects or controls
Passive mechanical properties of the lumbar multifidus muscle support its role as a stabilizer	Ward, S. R.; Tomiya, A.; Regev, G. J.; Thacker, B. E.; Benzl, R. C.; Kim, C. W.; Lieber, R. L.	2009	No healthy subjects or controls
Changes in the human mitochondrial genome after treatment of malignant disease	Wardell, T. M.; Ferguson, E.; Chinnery, P. F.; Borthwick, G. M.; Taylor, R. W.; Jackson, G.; Craft, A.; Lightowlers, R. N.; Howell, N.; Turnbull, D. M.	2003	Did not perform fiber type analysis
Human muscle cells express the costimulatory molecule B7-H3, which modulates muscle-immune interactions	Waschbisch, A.; Wintterle, S.; LochmÃ¼ller, H.; Walter, M. C.; Wischhusen, J.; Kieseier, B. C.; Wiendl, H.	2008	No healthy subjects or controls
Muscle fiber types of human extraocular muscles: a histochemical and immunohistochemical study	Wasicky, R.; Ziya-Ghazvini, F.; Blumer, R.; Lukas, J. R.; Mayr, R.	2000	Autopsies
Skeletal muscle fibre swelling contributes to force depression in rats and humans: a mechanically-skinned fibre study	Watanabe, D.; Dutka, T. L.; Lamboley, C. R.; Lamb, G. D.	2019	Does not include males and females
A quantitative study of myonuclear and satellite cell nuclear size in Duchenne's muscular dystrophy, polymyositis and normal human skeletal muscle	Watkins, S. C.; Cullen, M. J.	1988	Does not include male and female data
Insulin-stimulated Glut 4 translocation in human skeletal muscle: a quantitative confocal microscopical assessment	Watkins, S. C.; Frederickson, A.; Theriault, R.; Korytkowski, M.; Turner, D. S.; Kelley, D. E.	1997	Did not perform fiber type analysis
Reductions in skeletal muscle mitochondrial mass are not restored following exercise training in patients with chronic kidney disease	Watson, E. L.; Baker, L. A.; Wilkinson, T. J.; Gould, D. W.; Graham-Brown, M. P. M.; Major, R. W.; Ashford, R. U.; Philp, A.; Smith, A. C.	2020	Did not perform fiber type analysis
Combined walking exercise and alkali therapy in patients with CKD4-5 regulates intramuscular free amino acid pools and ubiquitin E3 ligase expression	Watson, E. L.; Kosmadakis, G. C.; Smith, A. C.; Viana, J. L.; Brown, J. R.; Molyneux, K.; Pawluczyk, I. Z.; Mulheran, M.; Bishop, N. C.; Shirreffs, S.; Maughan, R. J.; Owen, P. J.;	2013	No healthy subjects or controls

	John, S. G.; McIntyre, C. W.; Feehally, J.; Bevington, A.		
Regulation of HSL serine phosphorylation in skeletal muscle and adipose tissue	Watt, M. J.; Holmes, A. G.; Pinnamaneni, S. K.; Garnham, A. P.; Steinberg, G. R.; Kemp, B. E.; Febbraio, M. A.	2006	Did not perform fiber type analysis
Idiopathic chronic fatigue in older adults is linked to impaired mitochondrial content and biogenesis signaling in skeletal muscle	Wawrzyniak, N. R.; Joseph, A. M.; Levin, D. G.; Gundermann, D. M.; Leeuwenburgh, C.; Sandesara, B.; Manini, T. M.; Adhihetty, P. J.	2016	Did not perform fiber type analysis
Muscle cell death in developing human skeletal muscle	Webb, J. N.	1971	Only in children (0-17 years)
A new mtDNA mutation showing accumulation with time and restriction to skeletal muscle	Weber, K.; Wilson, J. N.; Taylor, L.; Brierley, E.; Johnson, M. A.; Turnbull, D. M.; Bindoff, L. A.	1997	Does not report sex of subjects
Myoglobin plasma level related to muscle mass and fiber composition: a clinical marker of muscle wasting?	Weber, M. A.; Kinscherf, R.; Krakowski-Roosen, H.; Aulmann, M.; Renk, H.; KÃ¼nkele, A.; Edler, L.; Kauczor, H. U.; Hildebrandt, W.	2007	Does not include male and female data
Relationship of skeletal muscle perfusion measured by contrast-enhanced ultrasonography to histologic microvascular density	Weber, M. A.; Krakowski-Roosen, H.; Delorme, S.; Renk, H.; Krix, M.; Millies, J.; Kinscherf, R.; KÃ¼nkele, A.; Kauczor, H. U.; Hildebrandt, W.	2006	Does not include male and female data
Morphology, metabolism, microcirculation, and strength of skeletal muscles in cancer-related cachexia	Weber, M. A.; Krakowski-Roosen, H.; SchrÃ¶der, L.; Kinscherf, R.; Krix, M.; Kopp-Schneider, A.; Essig, M.; Bachert, P.; Kauczor, H. U.; Hildebrandt, W.	2009	Does not include male and female data
Critical illness myopathy and GLUT4: significance of insulin and muscle contraction	Weber-Carstens, S.; Schneider, J.; Wollersheim, T.; Assmann, A.; Bierbrauer, J.; Marg, A.; Al Hasani, H.; Chadt, A.; Wenzel, K.; Koch, S.; Fielitz, J.; Kleber, C.; Faust, K.; Mai, K.; Spies, C. D.; Luft, F. C.; Boschmann, M.; Spranger, J.; Spuler, S.	2013	Does not include male and female data
LMNA rs4641 and the muscle lamin A and C isoforms in twins--metabolic implications and transcriptional regulation	Wegner, L.; Anthonsen, S.; Bork-Jensen, J.; Dalgaard, L.; Hansen, T.; Pedersen, O.; Poulsen, P.; Vaag, A.	2010	No cross-sectional area data
Functional characterization of malignant hyperthermia-associated RyR1 mutations in exon 44, using the human myotube model	Wehner, M.; Rueffert, H.; Koenig, F.; Olthoff, D.	2004	Did not perform fiber type analysis
Resistance exercise and cyclooxygenase (COX) expression in human skeletal muscle: implications for COX-inhibiting drugs and protein synthesis	Weinheimer, E. M.; Jemiolo, B.; Carroll, C. C.; Harber, M. P.; Haus, J. M.; Burd, N. A.; LeMoine, J. K.; Trappe, S. W.; Trappe, T. A.	2007	No cross-sectional area data
Increased expression of CNTF receptor alpha in denervated human skeletal muscle	Weis, J.; Lie, D. C.; Ragoss, U.; ZÃ¼chner, S. L.; SchrÃ¶der, J. M.; Karpati, G.; Farruggella, T.; Stahl, N.; Yancopoulos, G. D.; DiStefano, P. S.	1998	Did not perform fiber type analysis
Oxidative damage and myofiber degeneration in the gastrocnemius of patients with peripheral arterial disease	Weiss, D. J.; Casale, G. P.; Koutakis, P.; Nella, A. A.; Swanson, S. A.; Zhu, Z.; Miserlis, D.; Johanning, J. M.; Pipinos,, II	2013	Did not perform fiber type analysis
Ryanodine contracture threshold times for diagnosis of malignant hyperthermia	Weisshorn, R.; Wappler, F.; Fiege, M.; Gerbershagen, M. U.; Kolodzie, K.; Alberts, P.; Horn, E. P.; Schulte Am Esch, J.	2004	No healthy subjects or controls

susceptibility: an experimental approach from a single laboratory			
High-abundance mRNAs in human muscle: comparison between young and old	Welle, S.; Bhatt, K.; Thornton, C. A.	2000	Does not include males and females
Expression of elongation factor-1 alpha and S1 in young and old human skeletal muscle	Welle, S.; Thornton, C.; Bhatt, K.; Krym, M.	1997	Did not perform fiber type analysis
Variability of triacylglycerol content in human skeletal muscle biopsy samples	Wendling, P. S.; Peters, S. J.; Heigenhauser, G. J.; Spriet, L. L.	1996	Did not perform fiber type analysis
Contractile function and sarcolemmal permeability after acute low-load resistance exercise with blood flow restriction	Wernbom, M.; Paulsen, G.; Nilsen, T. S.; Hisdal, J.; Raastad, T.	2012	Does not include male and female data
Stress hormones alter the pattern of free amino acids in human skeletal muscle	Wernerman, J.; Hammarqvist, F.; Botta, D.; Vinars, E.	1993	Does not include males and females
Enzymatic capacity and protein synthesis in human muscle postoperatively	Wernerman, J.; SylvÃ©n, C.; Jansson, E.; von der Decken, A.; Vinnars, E.	1988	No healthy subjects or controls
Size distribution of ribosomes in biopsy specimens of human skeletal muscle during starvation	Wernerman, J.; von der Decken, A.; Vinnars, E.	1985	Did not perform fiber type analysis
Extrasynaptic location of laminin beta 2 chain in developing and adult human skeletal muscle	Wewer, U. M.; Thornell, L. E.; Loechel, F.; Zhang, X.; Durkin, M. E.; Amano, S.; Burgeson, R. E.; Engvall, E.; Albrechtsen, R.; Virtanen, I.	1997	Does not report sex of subjects
Immunocytochemical studies of cathepsin D in human skeletal muscle	Whitaker, J. N.; Bertorini, T. E.; Mendell, J. R.	1983	Does not report sex of subjects
Mitochondrial dysfunction in adults after out-of-hospital cardiac arrest	Wiberg, S.; Stride, N.; Bro-Jeppesen, J.; Holmberg, M. J.; KjÃ¦rgaard, J.; Larsen, S.; Donnino, M. W.; Hassager, C.; Dela, F.	2020	Did not perform fiber type analysis
ATP production rate in mitochondria isolated from microsamples of human muscle	Wibom, R.; Hultman, E.	1990	Did not perform fiber type analysis
Histochemical and immunohistochemical study on muscle fibers in human extraocular muscle spindles	Wicke, W.; Wasicky, R.; Brugger, P. C.; Kaminski, S.; Lukas, J. R.	2007	Autopsies
Contractile properties of rat, rhesus monkey, and human type I muscle fibers	Widrick, J. J.; Romatowski, J. G.; Karhanek, M.; Fitts, R. H.	1997	Does not include males and females
Isometric force and maximal shortening velocity of single muscle fibers from elite master runners	Widrick, J. J.; Trappe, S. W.; Blaser, C. A.; Costill, D. L.; Fitts, R. H.	1996	Does not include males and females
Unilateral lower limb suspension does not mimic bed rest or spaceflight effects on human muscle fiber function	Widrick, J. J.; Trappe, S. W.; Romatowski, J. G.; Riley, D. A.; Costill, D. L.; Fitts, R. H.	2002	Does not include male and female data
Impairment of mitochondrial function in skeletal muscle of patients with amyotrophic lateral sclerosis	Wiedemann, F. R.; Winkler, K.; Kuznetsov, A. V.; Bartels, C.; Vielhaber, S.; Feistner, H.; Kunz, W. S.	1998	Does not include male and female data

Oestrogen receptor beta is present in both muscle fibres and endothelial cells within human skeletal muscle tissue	Wiik, A.; Ekman, M.; Morgan, G.; Johansson, O.; Jansson, E.; EsbjÃ¶rnsson, M.	2005	Did not perform fiber type analysis
Beta-adrenergic receptor blockade does not inhibit cold-induced thermogenesis in humans: possible involvement of brown adipose tissue	Wijers, S. L.; Schrauwen, P.; van Baak, M. A.; Saris, W. H.; van Marken Lichtenbelt, W. D.	2011	Does not include males and females
Acute Maltodextrin Supplementation During Resistance Exercise	Wilburn, D. T.; Machek, S. B.; Cardaci, T. D.; Hwang, P. S.; Willoughby, D. S.	2020	Does not include males and females
Relaxation rate of constituent muscle-fibre types in human quadriceps	Wiles, C. M.; Young, A.; Jones, D. A.; Edwards, R. H.	1979	Does not report sex of subjects
Unexpected dependence of RyR1 splice variant expression in human lower limb muscles on fiber-type composition	Willemse, H.; Theodoratos, A.; Smith, P. N.; Dulhunty, A. F.	2016	No healthy subjects or controls
Aberrant REDD1-mTORC1 responses to insulin in skeletal muscle from Type 2 diabetics	Williamson, D. L.; Dungan, C. M.; Mahmoud, A. M.; Mey, J. T.; Blackburn, B. K.; Haus, J. M.	2015	Did not perform fiber type analysis
Myomonitor rest position in the presence and absence of stress	Williamson, E. H.; Marshall, D. E., Jr.	1986	Did not perform muscle biopsy
Blood oxidative stress and post-exercise recovery are unaffected byhypobaric and hypoxic environments	Williamson-Reisdorph, C. M.; Quindry, T. S.; Tiemessen, K. G.; Cuddy, J.; Hailes, W.; Slivka, D.; Ruby, B. C.; Quindry, J. C.	2021	Did not perform fiber type analysis
The unified myofibrillar matrix for force generation in muscle	Willingham, T. B.; Kim, Y.; Lindberg, E.; Bleck, C. K. E.; Glancy, B.	2020	Animal study
p38 MAPK activation and H3K4 trimethylation is decreased by lactate in vitro and high intensity resistance training in human skeletal muscle	Willkomm, L.; Gehlert, S.; Jacko, D.; Schiffer, T.; Bloch, W.	2017	Does not include males and females
Incidence of acetylcholinesterase in the sarcoplasm of human and chicken muscles	Wilson, B. W.; Taylor, R. G.; Fowler, W. M., Jr.; Patterson, G. T.; Nieberg, P. A.; Linkhart, S. G.; Linkhart, T. A.; Fry, D. M.	1975	Does not include males and females
The challenges of muscle biopsy in a community based geriatric population	Wilson, D.; Breen, L.; Lord, J. M.; Sapey, E.	2018	Did not perform fiber type analysis
Specific labelling of myonuclei by an antibody against pericentriolar material 1 on skeletal muscle tissue sections	Winje, I. M.; Bengtsen, M.; EftestÃ¸l, E.; Juvkam, I.; Bruusgaard, J. C.; Gundersen, K.	2018	Does not report sex of subjects
Re-evaluation of the dysfunction of mitochondrial respiratory chain in skeletal muscle of patients with Parkinson's disease	Winkler-Stuck, K.; Kirches, E.; Mawrin, C.; Dietzmann, K.; Lins, H.; Wallesch, C. W.; Kunz, W. S.; Wiedemann, F. R.	2005	No healthy subjects or controls
Increased net muscle protein balance in response to simultaneous and separate ingestion of carbohydrate and essential amino acids following resistance exercise	Witard, O. C.; Cocke, T. L.; Ferrando, A. A.; Wolfe, R. R.; Tipton, K. D.	2014	Did not perform fiber type analysis
Regulatory gene expression in skeletal muscle of highly endurance-trained humans	Wittwer, M.; Billeter, R.; Hoppeler, H.; FlÃ¼ck, M.	2004	Does not include males and females
Isoform-specific and exercise intensity-dependent activation of 5'-AMP-activated protein kinase in human skeletal muscle	Wojtaszewski, J. F.; Nielsen, P.; Hansen, B. F.; Richter, E. A.; Kiens, B.	2000	Does not include males and females
Regulation of glycogen synthase kinase-3 in human skeletal muscle: effects of food intake and bicycle exercise	Wojtaszewski, J. F.; Nielsen, P.; Kiens, B.; Richter, E. A.	2001	Does not include males and females

Production and release of acylcarnitines by primary myotubes reflect the differences in fasting fat oxidation of the donors	Wolf, M.; Chen, S.; Zhao, X.; Scheler, M.; Irmler, M.; Staiger, H.; Beckers, J.; de Angelis, M. H.; Fritsche, A.; HÃ¤ring, H. U.; Schleicher, E. D.; Xu, G.; Lehmann, R.; Weigert, C.	2013	Did not perform fiber type analysis
Longitudinal measures of RNA expression and disease activity in FSHD muscle biopsies	Wong, C. J.; Wang, L. H.; Friedman, S. D.; Shaw, D.; Campbell, A. E.; Budech, C. B.; Lewis, L. M.; Lemmers, Rjfl; Statland, J. M.; van der Maarel, S. M.; Tawil, R. N.; Tapscott, S. J.	2020	Did not perform fiber type analysis
Perifascicular atrophic fibers in childhood dermatomyositis with particular reference to mitochondrial changes	Woo, M.; Chung, S. J.; Nonaka, I.	1988	No healthy subjects or controls
Development of a diagnostic method for detecting increased muscle protein degradation in patients with catabolic conditions	Workeneh, B. T.; Rondon-Berrios, H.; Zhang, L.; Hu, Z.; Ayehu, G.; Ferrando, A.; Kopple, J. D.; Wang, H.; Storer, T.; Fournier, M.; Lee, S. W.; Du, J.; Mitch, W. E.	2006	Did not perform fiber type analysis
Electron probe X-ray microanalysis of human skeletal muscle involved in rheumatoid arthritis	WrÃ³blewski, R.; Gremski, W.; Nordemar, R.; EdstrÃ¶m, L.	1978	Does not report sex of subjects
Ultrastructural and histochemical studies of muscle in rheumatoid arthritis	WrÃ³blewski, R.; Nordemar, R.	1975	Did not perform fiber type analysis
Integrative transcriptomics and proteomic analysis of extraocular muscles from patients with thyroid-associated ophthalmopathy	Wu, L.; Zhang, S.; Li, X.; Yao, J.; Ling, L.; Huang, X.; Hu, C.; Zhang, Y.; Sun, X.; Qin, B.; Liu, G.; Zhao, C.	2020	Autopsies
Genes and biochemical pathways in human skeletal muscle affecting resting energy expenditure and fuel partitioning	Wu, X.; Patki, A.; Lara-Castro, C.; Cui, X.; Zhang, K.; Walton, R. G.; Osier, M. V.; Gadbury, G. L.; Allison, D. B.; Martin, M.; Garvey, W. T.	2011	Did not perform fiber type analysis
The effect of insulin on expression of genes and biochemical pathways in human skeletal muscle	Wu, X.; Wang, J.; Cui, X.; Maianu, L.; Rhees, B.; Rosinski, J.; So, W. V.; Willi, S. M.; Osier, M. V.; Hill, H. S.; Page, G. P.; Allison, D. B.; Martin, M.; Garvey, W. T.	2007	Did not perform fiber type analysis
Fiber capillary supply related to fiber size and oxidative capacity in human and rat skeletal muscle	WÃ¼st, R. C.; Gibbings, S. L.; Degens, H.	2009	Does not include male and female data
Preservation of skeletal muscle mitochondrial content in older adults: relationship between mitochondria, fibre type and high-intensity exercise training	Wyckelsma, V. L.; Levinger, I.; McKenna, M. J.; Formosa, L. E.; Ryan, M. T.; Petersen, A. C.; Anderson, M. J.; Murphy, R. M.	2017	Does not report sex of subjects
Intense interval training in healthy older adults increases skeletal muscle [(3)H]ouabain-binding site content and elevates Na(+),K(+)-ATPase Î±(2) isoform abundance in Type II fibers	Wyckelsma, V. L.; Levinger, I.; Murphy, R. M.; Petersen, A. C.; Perry, B. D.; Hedges, C. P.; Anderson, M. J.; McKenna, M. J.	2017	Does not include male and female data
Cell specific differences in the protein abundances of GAPDH and Na(+),K(+)-ATPase in skeletal muscle from aged individuals	Wyckelsma, V. L.; McKenna, M. J.; Levinger, I.; Petersen, A. C.; Lamboley, C. R.; Murphy, R. M.	2016	Does not include male and female data
Single-fiber expression and fiber-specific adaptability to short-term intense exercise training of Na+-K+-ATPase Î±- and Î²-isoforms in human skeletal muscle	Wyckelsma, V. L.; McKenna, M. J.; Serpiello, F. R.; Lamboley, C. R.; Aughey, R. J.; Stepto, N. K.; Bishop, D. J.; Murphy, R. M.	2015	Does not include male and female data

Expression of B7-homolog 1 in Polymyositis	Xiaoyu, D.; Yunxia, W.; Qi, F.; Dapeng, W.; Xiuying, C.; Jianhua, J.; Hongxia, W.	2011	Did not perform fiber type analysis
Alternative splicing factor ASF/SF2 is down regulated in inflamed muscle	Xiong, Z.; Shaibani, A.; Li, Y. P.; Yan, Y.; Zhang, S.; Yang, Y.; Yang, F.; Wang, H.; Yang, X. F.	2006	No healthy subjects or controls
Reduction of mitochondria and up regulation of pyruvate dehydrogenase kinase 4 of skeletal muscle in patients with chronic kidney disease	Xu, C.; Kasimumali, A.; Guo, X.; Lu, R.; Xie, K.; Zhu, M.; Qian, Y.; Chen, X.; Pang, H.; Wang, Q.; Fan, Z.; Dai, H.; Mou, S.; Ni, Z.; Gu, L.	2020	Did not perform fiber type analysis
Cardiac troponin T and fast skeletal muscle denervation in ageing	Xu, Z.; Feng, X.; Dong, J.; Wang, Z. M.; Lee, J.; Furdui, C.; Files, D. C.; Beavers, K. M.; Kritchevsky, S.; Milligan, C.; Jin, J. P.; Delbono, O.; Zhang, T.	2017	Does not include males and females
Distinct and additive effects of sodium bicarbonate and continuous mild heat stress on fiber type shift via calcineurin/NFAT pathway in human skeletal myoblasts	Yamaguchi, T.; Omori, M.; Tanaka, N.; Fukui, N.	2013	Does not include males and females
Time course of myogenic and metabolic gene expression in response to acute exercise in human skeletal muscle	Yang, Y.; Creer, A.; Jemiolo, B.; Trappe, S.	2005	Does not include male and female data
DUX4-induced gene expression is the major molecular signature in FSHD skeletal muscle	Yao, Z.; Snider, L.; Balog, J.; Lemmers, R. J.; Van Der Maarel, S. M.; Tawil, R.; Tapscott, S. J.	2014	Did not perform fiber type analysis
Isoenzyme distribution of creatine kinase and lactate dehydrogenase in serum and skeletal muscle in Duchenne muscular dystrophy, collagen disease, and other muscular disorders	Yasmineh, W. G.; Ibrahim, G. A.; Abbasnezhad, M.; Awad, E. A.	1978	No healthy subjects or controls
Fat adaptation followed by carbohydrate restoration increases AMPK activity in skeletal muscle from trained humans	Yeo, W. K.; Lessard, S. J.; Chen, Z. P.; Garnham, A. P.; Burke, L. M.; Rivas, D. A.; Kemp, B. E.; Hawley, J. A.	2008	Does not include males and females
Acute signalling responses to intense endurance training commenced with low or normal muscle glycogen	Yeo, W. K.; McGee, S. L.; Carey, A. L.; Paton, C. D.; Garnham, A. P.; Hargreaves, M.; Hawley, J. A.	2010	Does not include males and females
Skeletal muscle adaptation and performance responses to once a day versus twice every second day endurance training regimens	Yeo, W. K.; Paton, C. D.; Garnham, A. P.; Burke, L. M.; Carey, A. L.; Hawley, J. A.	2008	Does not include males and females
Defect in insulin action on expression of the muscle/adipose tissue glucose transporter gene in skeletal muscle of type 1 diabetic patients	Yki-JÃ¤rvinen, H.; Vuorinen-Markkola, H.; Koranyi, L.; Bourey, R.; Tordjman, K.; Mueckler, M.; Permutt, A. M.; Koivisto, V. A.	1992	Does not include males and females
Histochemical changes in the multifidus muscle in patients with lumbar intervertebral disc herniation	Yoshihara, K.; Shirai, Y.; Nakayama, Y.; Uesaka, S.	2001	No healthy subjects or controls
Ultrastructural and histochemical study of the motor end plates of the intrinsic laryngeal muscles in amyotrophic lateral sclerosis	Yoshihara, T.; Ishii, T.; Iwata, M.; Nomoto, M.	1998	No healthy subjects or controls
Immunohistochemical analysis of clinically transplanted muscles	Yoshimura, K.; Harii, K.; Asato, H.; Ueda, K.; Yamada, A.	1998	Does not report sex of subjects
Effect of dietary n-3 PUFA supplementation on the muscle transcriptome in older adults	Yoshino, J.; Smith, G. I.; Kelly, S. C.; Julliand, S.; Reeds, D. N.; Mittendorfer, B.	2016	Did not perform fiber type analysis

Human skeletal muscle fibers in normal and pathological states; freeze-etch replica observations	Yoshioka, M.; Okuda, R.	1977	No healthy subjects or controls
Effect of dietary macronutrient composition on tissue-specific lipoprotein lipase activity and insulin action in normal-weight subjects	Yost, T. J.; Jensen, D. R.; Haugen, B. R.; Eckel, R. H.	1998	Did not perform fiber type analysis
The effect of knee injury on the number of muscle fibres in the human quadriceps femoris	Young, A.; Hughes, I.; Round, J. M.; Edwards, R. H.	1982	Does not include male and female data
Skeletal muscle metabolism of sea-level natives following short-term high-altitude residence	Young, A. J.; Evans, W. J.; Fisher, E. C.; Sharp, R. L.; Costill, D. L.; Maher, J. T.	1984	Does not include males and females
Insulin receptor autophosphorylation in cultured myoblasts correlates to glucose disposal in Pima Indians	Youngren, J. F.; Goldfine, I. D.; Pratley, R. E.	1999	Did not perform fiber type analysis
Human single masseter muscle fibers contain unique combinations of myosin and myosin binding protein C isoforms	Yu, F.; StÃ¥l, P.; Thornell, L. E.; Larsson, L.	2002	Does not include males and females
Evidence for myofibril remodeling as opposed to myofibril damage in human muscles with DOMS: an ultrastructural and immunoelectron microscopic study	Yu, J. G.; Carlsson, L.; Thornell, L. E.	2004	Does not include males and females
The mode of myofibril remodelling in human skeletal muscle affected by DOMS induced by eccentric contractions	Yu, J. G.; FÃ¼rst, D. O.; Thornell, L. E.	2003	Does not include males and females
Eccentric contractions leading to DOMS do not cause loss of desmin nor fibre necrosis in human muscle	Yu, J. G.; Malm, C.; Thornell, L. E.	2002	Does not include males and females
Desmin and actin alterations in human muscles affected by delayed onset muscle soreness: a high resolution immunocytochemical study	Yu, J. G.; Thornell, L. E.	2002	Does not include males and females
Muscle weakness in TPM3-myopathy is due to reduced Ca2+-sensitivity and impaired acto-myosin cross-bridge cycling in slow fibres	Yuen, M.; Cooper, S. T.; Marston, S. B.; Nowak, K. J.; McNamara, E.; Mokbel, N.; Ilkovski, B.; Ravenscroft, G.; Rendu, J.; de Winter, J. M.; Klinge, L.; Beggs, A. H.; North, K. N.; Ottenheijm, C. A.; Clarke, N. F.	2015	Does not report sex of subjects
Electron microscopic studies of muscle biopsy in primary fibromyalgia syndrome: a controlled and blinded study	Yunus, M. B.; Kalyan-Raman, U. P.; Masi, A. T.; Aldag, J. C.	1989	Does not include males and females
Plasma mitochondrial DNA is elevated in obese type 2 diabetes mellitus patients and correlates positively with insulin resistance	Yuzefovych, L. V.; Pastukh, V. M.; Ruchko, M. V.; Simmons, J. D.; Richards, W. O.; Rachek, L. I.	2019	Did not perform fiber type analysis
Comparison of different mass spectrometry techniques in the measurement of L-[ring-(13)C6]phenylalanine incorporation into mixed muscle proteins	Zabielski, P.; Ford, G. C.; Persson, X. M.; Jaleel, A.; Dewey, J. D.; Nair, K. S.	2013	Does not report sex of subjects
Intravenous glutamine does not stimulate mixed muscle protein synthesis in healthy young men and women	Zachwieja, J. J.; Witt, T. L.; Yarasheski, K. E.	2000	Did not perform fiber type analysis
STAC3 variants cause a congenital myopathy with distinctive dysmorphic features and malignant hyperthermia susceptibility	Zaharieva, I. T.; Sarkozy, A.; Munot, P.; Manzur, A.; O'Grady, G.; Rendu, J.; Malfatti, E.; Amthor, H.; Servais, L.; Urtizberea, J. A.;	2018	No healthy subjects or controls

	Neto, O. A.; Zanoteli, E.; Donkervoort, S.; Taylor, J.; Dixon, J.; Poke, G.; Foley, A. R.; Holmes, C.; Williams, G.; Holder, M.; Yum, S.; Medne, L.; Quijano-Roy, S.; Romero, N. B.; FaurÃ©, J.; Feng, L.; Bastaki, L.; Davis, M. R.; Phadke, R.; Sewry, C. A.; BÃ¶nnemann, C. G.; Jungbluth, H.; Bachmann, C.; Treves, S.; Muntoni, F.		
Etiopathogenesis of adolescent idiopathic scoliosis: Expression of melatonin receptors 1A/1B, calmodulin and estrogen receptorÂ 2 in deep paravertebral muscles revisited	Zamecnik, J.; Krskova, L.; Hacek, J.; Stetkarova, I.; Krbec, M.	2016	Did not perform fiber type analysis
Physical exercise in aging human skeletal muscle increases mitochondrial calcium uniporter expression levels and affects mitochondria dynamics	Zampieri, S.; Mammucari, C.; Romanello, V.; Barberi, L.; Pietrangelo, L.; Fusella, A.; Mosole, S.; Gherardi, G.; HÃ¶fer, C.; LÃ¶fler, S.; Sarabon, N.; Cvecka, J.; Krenn, M.; Carraro, U.; Kern, H.; Protasi, F.; MusarÃ², A.; Sandri, M.; Rizzuto, R.	2016	Does not include male and female data
Deficiency of muscle alpha-actinin-3 is compatible with high muscle performance	Zanoteli, E.; Lotuffo, R. M.; Oliveira, A. S.; Beggs, A. H.; Canovas, M.; Zatz, M.; Vainzof, M.	2003	Does not include males and females
Identification of hemostatic genes expressed in human and rat leg muscles and a novel gene (LPP1/PAP2A) suppressed during prolonged physical inactivity (sitting)	Zderic, T. W.; Hamilton, M. T.	2012	Does not include males and females
Relationship between PPARÎ± mRNA expression and mitochondrial respiratory function and ultrastructure of the skeletal muscle of patients with COPD	Zhang, J. Q.; Long, X. Y.; Xie, Y.; Zhao, Z. H.; Fang, L. Z.; Liu, L.; Fu, W. P.; Shu, J. K.; Wu, J. H.; Dai, L. M.	2017	Did not perform fiber type analysis
Fatty acid binding protein 3 is associated with skeletal muscle strength in polymyositis and dermatomyositis	Zhang, L.; Zhou, H.; Peng, Q.; Jiang, W.; Qiao, W.; Wang, G.	2017	Did not perform fiber type analysis
Hemojuvelin is a novel suppressor for Duchenne muscular dystrophy and age-related muscle wasting	Zhang, P.; He, J.; Wang, F.; Gong, J.; Wang, L.; Wu, Q.; Li, W.; Liu, H.; Wang, J.; Zhang, K.; Li, M.; Huang, X.; Pu, C.; Li, Y.; Jiang, F.; Wang, F.; Min, J.; Chen, X.	2019	Does not include male and female data
Activation of aconitase in mouse fast-twitch skeletal muscle during contraction-mediated oxidative stress	Zhang, S. J.; SandstrÃ¶m, M. E.; Lanner, J. T.; Thorell, A.; Westerblad, H.; Katz, A.	2007	Did not perform fiber type analysis
CKD autophagy activation and skeletal muscle atrophy-a preliminary study of mitophagy and inflammation	Zhang, Y. Y.; Gu, L. J.; Huang, J.; Cai, M. C.; Yu, H. L.; Zhang, W.; Bao, J. F.; Yuan, W. J.	2019	No healthy subjects or controls
Platelet-derived growth factor and its receptors are related to the progression of human muscular dystrophy: an immunohistochemical study	Zhao, Y.; Haginoya, K.; Sun, G.; Dai, H.; Onuma, A.; Iinuma, K.	2003	Does not report sex of subjects
Human myogenic endothelial cells exhibit chondrogenic and osteogenic potentials at the clonal level	Zheng, B.; Li, G.; Chen, W. C.; Deasy, B. M.; Pollett, J. B.; Sun, B.; Drowley, L.; Gharaibeh, B.; Usas, A.; PÃ©ault, B.; Huard, J.	2013	Did not perform fiber type analysis

Role of miRNAs in skeletal muscle aging	Zheng, Y.; Kong, J.; Li, Q.; Wang, Y.; Li, J.	2018	Did not perform fiber type analysis
Integrated analysis of long non-coding RNAs (lncRNAs) and mRNA expression profiles identifies lncRNA PRKG1-AS1 playing important roles in skeletal muscle aging	Zheng, Y.; Liu, T.; Li, Q.; Li, J.	2021	Did not perform fiber type analysis
Epigenetic allele silencing unveils recessive RYR1 mutations in core myopathies	Zhou, H.; Brockington, M.; Jungbluth, H.; Monk, D.; Stanier, P.; Sewry, C. A.; Moore, G. E.; Muntoni, F.	2006	Did not perform fiber type analysis
Myosin heavy chain isoforms of human muscle after short-term spaceflight	Zhou, M. Y.; Klitgaard, H.; Saltin, B.; Roy, R. R.; Edgerton, V. R.; Gollnick, P. D.	1995	Does not include male and female data
cDNA microarrays reveal distinct gene expression clusters in idiopathic inflammatory myopathies	Zhou, X.; Dimachkie, M. M.; Xiong, M.; Tan, F. K.; Arnett, F. C.	2004	Did not perform fiber type analysis
Human islet amyloid polypeptide at pharmacological levels inhibits insulin and phorbol ester-stimulated glucose transport in in vitro incubated human muscle strips	Zierath, J. R.; Galuska, D.; EngstrÃ¶m, A.; Johnson, K. H.; Betsholtz, C.; Westermark, P.; Wallberg-Henriksson, H.	1992	Did not perform fiber type analysis
Effect of human C-peptide on glucose transport in in vitro incubated human skeletal muscle	Zierath, J. R.; Galuska, D.; Johansson, B. L.; Wallberg-Henriksson, H.	1991	Does not include males and females
C-peptide stimulates glucose transport in isolated human skeletal muscle independent of insulin receptor and tyrosine kinase activation	Zierath, J. R.; Handberg, A.; Tally, M.; Wallberg-Henriksson, H.	1996	Does not include males and females
Insulin action on glucose transport and plasma membrane GLUT4 content in skeletal muscle from patients with NIDDM	Zierath, J. R.; He, L.; GumÃ , A.; Odegoard WahlstrÃ¶m, E.; Klip, A.; Wallberg-Henriksson, H.	1996	Does not include males and females
Coenzyme Q in serum and muscle of 5 patients with Kearns-Sayre syndrome and 12 patients with ophthalmoplegia plus	Zierz, S.; Jahns, G.; Jerusalem, F.	1989	Did not perform fiber type analysis
Additional biochemical criteria in the differential diagnosis of myositis	Zimmermann, C. W.; Langohr, H. D.; WiethÃ¶lter, H.; Peiffer, J.	1987	Does not include male and female data
Physical activity changes the regulation of mitochondrial respiration in human skeletal muscle	Zoll, J.; Sanchez, H.; N'Guessan, B.; Ribera, F.; Lampert, E.; Bigard, X.; Serrurier, B.; Fortin, D.; Geny, B.; Veksler, V.; Ventura-Clapier, R.; Mettauer, B.	2002	Does not include male and female data
Effects of immunosuppressive treatment on interleukin-15 and interleukin-15 receptor Î± expression in muscle tissue of patients with polymyositis or dermatomyositis	Zong, M.; Loell, I.; Lindroos, E.; Nader, G. A.; Alexanderson, H.; Hallengren, C. S.; Borg, K.; Arnardottir, S.; McInnes, I. B.; Lundberg, I. E.	2012	Did not perform fiber type analysis
The Duchenne muscular dystrophy gene product is localized in sarcolemma of human skeletal muscle	Zubrzycka-Gaarn, E. E.; Bulman, D. E.; Karpati, G.; Burghes, A. H.; Belfall, B.; Klamut, H. J.; Talbot, J.; Hodges, R. S.; Ray, P. N.; Worton, R. G.	1988	Did not perform fiber type analysis
Acetyl-CoA-driven respiration in frozen muscle contributes to the diagnosis of mitochondrial disease	Zuccolotto-Dos-Reis, F. H.; Escarso, S. H. A.; Araujo, J. S.; Espreafico, E. M.; Alberici, L. C.; Sobreira, Cfdr	2021	Did not perform fiber type analysis

Morphometric analysis of the capillary supply in skeletal muscles of trained and untrained subjects--its limitations in muscle biopsies	Zumstein, A.; Mathieu, O.; Howald, H.; Hoppeler, H.	1983	Did not perform fiber type analysis
Whole-body energy metabolism and skeletal muscle biochemical characteristics	Zurlo, F.; Nemeth, P. M.; Choksi, R. M.; Sesodia, S.; Ravussin, E.	1994	No cross-sectional area data
Mitochondrial creatine kinase activity alterations in skeletal muscle during long-distance running	Apple, F. S.; Rogers, M. A.	1986	Same subjects as another study
Skeletal muscle lactate dehydrogenase isozyme alterations in men and women marathon runners	Apple, F. S.; Rogers, M. A.	1986	Same subjects as another study
Relationship between lactate threshold during running and relative gastrocnemius area	Atomi, Y.; Fukunaga, T.; Hatta, H.; Yamamoto, Y.	1987	Did not perform fiber type analysis
Morphometric analyses of the muscles of weight-lifters	Cabric, M.	2002	Not in English
The effect of strength training on estimates of mitochondrial density and distribution throughout muscle fibres	Chilibeck, P. D.; Syrotuik, D. G.; Bell, G. J.	1999	Does not include male and female data
The effect of concurrent endurance and strength training on quantitative estimates of subsarcolemmal and intermyofibrillar mitochondria. / Effets d ' un entrainement combine d ' endurance et de musculation sur les estimations quantitatives des mitochondries du sous-sarcolemme et des myofibrilles	Chilibeck, P. D.; Syrotuik, D. G.; Bell, G. J.	2002	No cross-sectional area data
Cycling efficiency is related to the percentage of Type I muscle fibers. / L' efficacite lors de l' exercice est en rapport avec le pourcentage des fibres musculaires de type I	Coyle, E. F.; Sidossis, L. S.; Horowitz, J. F.; Beltz, J. D.	1992	Does not include males and females
Glycogen depletion in specific types of human skeletal muscle fibers in intermittent and continuous exercise	Edgerton, V. R.; Essen, B.; Saltin, B.; Simpson, D. R.	1973	Does not report sex of subjects
Rapid carbohydrate loading after a short bout of near maximal-intensity exercise. / Chargement rapide en glucide suite a une courte serie d ' exercices proche de l ' intensite maximale	Fairchild, T. J.; Fletcher, S.; Steele, P.; Goodman, C.; Dawson, B.; Fournier, P. A.	2002	Does not include males and females
Skeletal muscle fibre types in teenagers: relationship to physical performance and activity	Jansson, E.; Hedberg, G.	1991	Only in children (0-17 years)
Critical Power Concept: Males vs. Females and the Impact of Muscle Fiber Composition	Kantor, Michael A.; Albers, Jessica; Weed, Katelyn; Erickson, Zachary O.	2019	Did not perform muscle biopsy
Muscle and Blood Metabolites during a Soccer Game: Implications for Sprint Performance	Krustrup, Peter; Mohr, Magni; Steensberg, Adam; Bencke, Jesper; Kjaer, Michael; Bangsbo, Jens	2006	Does not report sex of subjects
Glycogen synthesis during exercise and rest with carbohydrate feeding in males and females. / Synthese du glycogene lors de l' exercice et au repos, avec une alimentation riche en hydrate de carbone, chez des hommes et des femmes	Kuipers, H.; Saris, W. H.; Brouns, F.; Keizer, H. A.; ten Bosch, C.	1989	Does not include male and female data
INDICES OF NEW MUSCLE FIBRES FORMATION IN YOUNG TRAINED WOMEN. / UKAZOVATELE	Laczo, Eugen; Truls, Raastad; Cumming, Kristoffer Toldnes; Sedlak, Peter; Zelko, Aurel	2010	Does not include males and females

NOVÃ�CH FORMÃ�CIÃ� SVALOVÃ�CH VLÃ�KIEN MLADÃ�CH Å PORTOVKÃ�Å‡			
Effects of acute moderate-intensity exercise on carnitine metabolism in men and women	Lennon, D. L. F.; Stratman, F. W.; Shrago, E.; Nagle, F. J.; Madden, M.; Hanson, P.; Carter, A. L.	1983	Did not perform fiber type analysis
Performance and fibre characteristics of human skeletal muscle during short sprint training and detraining on a cycle ergometer. / Performance et caracteristiques des fibres musculaires lors d ' un entrainement de sprint court et de desentrainement sur cycle ergometrique	Linossier, M. T.; Dormois, D.; Geyssant, A.; Denis, C.	1997	Does not include males and females
Influence of peak VO2 and muscle fiber type on the efficiency of moderate exercise. / Influence de la puissance maximale aerobie et du type de fibre musculaire sur l ' efficacite d ' un exercice physique d ' intensite moderee	Mallory, L. A.; Scheuermann, B. W.; Hoelting, B. D.; Weiss, M. L.; McAllister, R. M.; Barstow, T. J.	2002	Does not include male and female data
Relationship between the maximal running velocity, muscle fiber characteristics, force production and force relaxation of sprinters	Mero, A.; Luhtanen, P.; Viitasalo, J. T.; Komi, P. V.	1981	Does not include males and females
Resistance Exercise And The Transcriptional Co-activator Pgc-1a4 Enhance Skeletal Muscle Glycolytic Metabolism	Pataky, Mark William; Koh, Jin-Ho; Dasari, Surendra; Klaus, Katherine; Prabha Kumar, Arathi; Ruegsegger, Gregory; Nair, K. Sreekumaran	2021	Not peer reviewed
Perilipin family (PLIN) proteins in human skeletal muscle: the effect of sex, obesity, and endurance training	Peters, Sandra J.; Samjoo, Imtiaz A.; Devries, Michaela C.; Stevic, Ivan; Robertshaw, Holly A.; Tarnopolsky, Mark A.	2012	Did not perform fiber type analysis
Quantitative measures of enzyme activities in type I and type II muscle fibres of man after training	Reitman, J. S.; Henriksson, J.	1976	Does not report sex of subjects
Muscle fiber type, enzyme activities and maximal work of short duration in sedentary male and female subjects. / Type de fibre musculaire, activites enzymatiques et travail maximal de courte duree chez des sujets sedentaires, hommes et femmes	Simoneau, J. A.; Bouchard, C.	1990	Full text not available
Muscle morphology and metabolic potential in elite road cyclists during a season	Sjogaard, G.	1984	Does not report sex of subjects
Effect of training and 15-, 25-, and 42-km contests on the skeletal muscle content of adenine and guanine nucleotides, creatine phosphate, and glycogen. / Effet de l ' entrainement et des competitions de course de 15 km, 25 km et 42 km sur les concentrations musculaires d ' adenine, de guanine nucleotides, de creatine phosphate et de glycogene	Van der Vusse, G. J.; Janssen, G. M. E.; Coumans, W. A.; Kuipers, H.; Does, R. J. M. M.; ten Hoor, F.	1989	Did not perform fiber type analysis
Effects of dietary fat on muscle substrates, metabolism, and performance in athletes. / Effets d ' une alimentation riche en graisse sur les substrats musculaires, le metabolisme et la performance des athletes	Vogt, M.; Puntschart, A.; Howald, H.; Mueller, B.; Mannhart, C.; Gfeller-Tuescher, L.; Mullis, P.; Hoppeler, H.	2003	Does not include males and females
Intramuscular pressures and muscle metabolism after short-term and long-term exercise	Wallensten, R.; Eklund, B.	1983	Does not report sex of subjects

A randomized placebo-controlled trial of nicotinamide riboside+pterostilbene supplementation in experimental muscle injury in elderly subjects	Jensen, J. B.; Dollerup, O. L.; Moller, A. B.; Billeskov, T. B.; Dalbram, E.; Chubanava, S.; Damgaard, M. V.; Dellinger, R. W.; Trost, K.; Moritz, T.; et al.,	2022	Does not include male and female data
The Effect of Neuromuscular Electrical Stimulation (NMES)	Nct,	2008	Not peer reviewed (ex: thesis, conference poster)
Cholecalciferol or 25-Hydroxycholecalciferol Supplementation Does Not Affect Muscle Strength and Physical Performance in Prefrail and Frail Older Adults	Vaes, A. M. M.; Tieland, M.; Toussaint, N.; Nilwik, R.; Verdijk, L. B.; van Loon, L. J. C.; de Groot, Lcpgm	2018	Does not include male and female data
Comparing D3-Creatine Dilution and Dual-Energy X-ray Absorptiometry Muscle Mass Responses to Strength Training in Low-Functioning Older Adults	Balachandran, A. T.; Evans, W. J.; Cawthon, P. M.; Wang, Y.; Shankaran, M.; Hellerstein, M. K.; Qiu, P.; Manini, T.	2023	Did not perform muscle biopsy
Greater glycemic control following low-load, high-repetition resistance exercise compared with moderate-intensity continuous exercise in males and females: a randomized control trial	Beaudry, K. M.; Surdi, J. C.; Pancevski, K.; Tremblay, C.; Devries, M. C.	2024	Did not perform fiber type analysis
DNA methylation of insulin signaling pathways is associated with HOMA2-IR in primary myoblasts from older adults	Burton, M. A.; Garratt, E. S.; Hewitt, M. O.; Sharkh, H. Y.; Antoun, E.; Westbury, L. D.; Dennison, E. M.; Harvey, N. C.; Cooper, C.; MacIsaac, J. L.; Kobor, M. S.; Patel, H. P.; Godfrey, K. M.; Lillycrop, K. A.	2023	Did not perform fiber type analysis
Different roles of circulating and intramuscular GDF15 as markers of skeletal muscle health	Chiariello, A.; Conte, G.; Rossetti, L.; Trofarello, L.; Salvioli, S.; Conte, M.	2024	Does not report sex of subjects
Autophagy gene expression in skeletal muscle of older individuals is associated with physical performance, muscle volume and mitochondrial function in the study of muscle, mobility and aging (SOMMA)	Coen, P. M.; Huo, Z.; Tranah, G. J.; Barnes, H. N.; Zhang, X.; Wolff, C. A.; Wu, K.; Cawthon, P. M.; Hepple, R. T.; Toledo, F. G. S.; Evans, D. S.; Santiago-FernÃ¡ndez, O.; Cuervo, A. M.; Kritchevsky, S. B.; Newman, A. B.; Cummings, S. R.; Esser, K. A.	2024	Did not perform fiber type analysis
Alaska Backcountry Expeditionary Hunting Promotes Sustained Muscle Protein Synthesis	Coker, R. H.; Ruby, B. C.; Coker, M. S.; Bartlett, L.; Kowalski, B.; Goropashnaya, A. V.; Bateman, T.; Shankaran, M.; Hellerstein, M.; Evans, W. J.	2023	Did not perform fiber type analysis
Signatures of cysteine oxidation on muscle structural and contractile proteins are associated with physical performance and muscle function in older adults: Study of Muscle, Mobility and Aging (SOMMA)	Day, N. J.; Kelly, S. S.; Lui, L. Y.; Mansfield, T. A.; Gaffrey, M. J.; Trejo, J. B.; Sagendorf, T. J.; Attah, I. K.; Moore, R. J.; Douglas, C. M.; Newman, A. B.; Kritchevsky, S. B.; Kramer, P. A.; Marcinek, D. J.; Coen, P. M.; Goodpaster, B. H.; Hepple, R. T.; Cawthon, P. M.; Petyuk, V. A.; Esser, K. A.; Qian, W. J.; Cummings, S. R.	2024	Did not perform fiber type analysis
Histopathological correlations and fat replacement imaging patterns in recessive limb-girdle muscular dystrophy type 12	De Wel, B.; Huysmans, L.; Depuydt, C. E.; Goosens, V.; Peeters, R.; Santos, F. P.; Thal, D. R.; Dupont, P.; Maes, F.; Claeys, K. G.	2023	Did not perform fiber type analysis
Acute and long-term exercise adaptation of adipose tissue and skeletal muscle in humans: a	Dreher, S. I.; Irmler, M.; Pivovarova-Ramich, O.; Kessler, K.; JÃ¼rchott, K.; Sticht, C.; Fritsche, L.; Schneeweiss, P.; Machann, J.;	2023	Did not perform fiber type analysis

matched transcriptomics approach after 8-week training-intervention	Pfeiffer, A. F. H.; HrabÄ› de Angelis, M.; Beckers, J.; Birkenfeld, A. L.; Peter, A.; Niess, A. M.; Weigert, C.; Moller, A.		
Chronic aryl hydrocarbon receptor activity impairs muscle mitochondrial function with tobacco smoking	Fitzgerald, L. F.; Lackey, J.; Moussa, A.; Shah, S. V.; Castellanos, A. M.; Khan, S.; Schonk, M.; Thome, T.; Salyers, Z. R.; Jakkidi, N.; Kim, K.; Yang, Q.; Hepple, R. T.; Ryan, T. E.	2024	Animal study
Sex-specific alteration in human muscle transcriptome with age	Gharpure, M.; Chen, J.; Nerella, R.; Vyavahare, S.; Kumar, S.; Isales, C. M.; Hamrick, M.; Adusumilli, S.; Fulzele, S.	2023	Did not perform fiber type analysis
The Relevance of Muscle Fiber Type to Physical Characteristics and Performance in Team-Sport Athletes	Hopwood, H. J.; Bellinger, P. M.; Compton, H. R.; Bourne, M. N.; Minahan, C.	2023	Review (ex: systematic review/meta-analysis)
Plant Protein Blend Ingestion Stimulates Postexercise Myofibrillar Protein Synthesis Rates Equivalently to Whey in Resistance-Trained Adults	Van Der Heijden I; Monteyne, A. J.; West, S.; Morton, J. P.; Langan-Evans, C.; Hearris, M. A.; Abdelrahman, D. R.; Murton, A. J.; Stephens, F. B.; Wall, B. T.	2024	Did not perform fiber type analysis
Human skeletal muscle aging atlas	Kedlian, V. R.; Wang, Y.; Liu, T.; Chen, X.; Bolt, L.; Tudor, C.; Shen, Z.; Fasouli, E. S.; Prigmore, E.; Kleshchevnikov, V.; Pett, J. P.; Li, T.; Lawrence, J. E. G.; Perera, S.; Prete, M.; Huang, N.; Guo, Q.; Zeng, X.; Yang, L.; PolaÅ„ski, K.; Chipampe, N. J.; Dabrowska, M.; Li, X.; Bayraktar, O. A.; Patel, M.; Kumasaka, N.; Mahbubani, K. T.; Xiang, A. P.; Meyer, K. B.; Saeb-Parsy, K.; Teichmann, S. A.; Zhang, H.	2024	Review (ex: systematic review/meta-analysis)
Skeletal Muscle Energetics Explain the Sex Disparity in Mobility Impairment in the Study of Muscle, Mobility and Aging	Kramer, P. A.; Coen, P. M.; Cawthon, P. M.; Distefano, G.; Cummings, S. R.; Goodpaster, B. H.; Hepple, R. T.; Kritchevsky, S. B.; Shankland, E. G.; Marcinek, D. J.; Toledo, F. G. S.; Duchowny, K. A.; Ramos, S. V.; Harrison, S.; Newman, A. B.; Molina, A. J. A.	2024	Did not perform fiber type analysis
microRNA-mRNA expression profiles in the skeletal muscle of myotonic dystrophy type 1	Li, M.; Li, Y.; Wang, Z.; Cui, F.; Yang, F.; Wang, H.; Shi, Q.; Huang, X.	2024	No healthy subjects or controls
Higher expression of denervation-responsive genes is negatively associated with muscle volume and performance traits in the study of muscle, mobility, and aging (SOMMA)	Lukasiewicz, C. J.; Tranah, G. J.; Evans, D. S.; Coen, P. M.; Barnes, H. N.; Huo, Z.; Esser, K. A.; Zhang, X.; Wolff, C.; Wu, K.; Lane, N. E.; Kritchevsky, S. B.; Newman, A. B.; Cummings, S. R.; Cawthon, P. M.; Hepple, R. T.	2024	Did not perform fiber type analysis
Mitochondrial Energetics in Skeletal Muscle Are Associated With Leg Power and Cardiorespiratory Fitness in the Study of Muscle, Mobility and Aging	Mau, T.; Lui, L. Y.; Distefano, G.; Kramer, P. A.; Ramos, S. V.; Toledo, F. G. S.; Santanasto, A. J.; Shankland, E. G.; Marcinek, D. J.; Jurczak, M. J.; Sipula, I.; Bello, F. M.; Duchowny, K. A.; Molina, A. J. A.; Sparks, L. M.; Goodpaster, B. H.; Hepple, R. T.; Kritchevsky, S. B.; Newman, A. B.;	2023	Did not perform fiber type analysis

	Cawthon, P. M.; Cummings, S. R.; Coen, P. M.		
Small non-coding RNA profiling in patients with gastrointestinal cancer	Molfino, A.; BeltrÃ , M.; Amabile, M. I.; Belli, R.; Birolo, G.; Belloni, E.; De Lucia, S.; Garcia-Castillo, L.; Penna, F.; Imbimbo, G.; Nigri, G.; Pardini, B.; Costelli, P.; Muscaritoli, M.	2023	No healthy subjects or controls
Metabolomic response to acute resistance exercise in healthy older adults by 1H-NMR	Moosavi, D.; Vuckovic, I.; Kunz, H. E.; Lanza, I. R.	2024	Did not perform fiber type analysis
Human skeletal muscle-specific atrophy with aging: a comprehensive review	Naruse, M.; Trappe, S.; Trappe, T. A.	2023	Review (ex: systematic review/meta-analysis)
Effects of aging and lifelong aerobic exercise on expression of innate immune components in skeletal muscle of women	Perkins, R. K.; Lavin, K. M.; Raue, U.; Jemiolo, B.; Trappe, S. W.; Trappe, T. A.	2024	Does not include males and females
Acyl-CoA synthetase expression in human skeletal muscle is reduced in obesity and insulin resistance	Poppelreuther, M.; Lundsgaard, A. M.; Mensberg, P.; SjÃ¸berg, K.; VilsbÃ¸ll, T.; Kiens, B.; FÃ¼llekrug, J.	2023	Did not perform fiber type analysis
Associations between skeletal muscle energetics and accelerometry-based performance fatigability: Study of Muscle, Mobility and Aging	Qiao, Y. S.; Santanasto, A. J.; Coen, P. M.; Cawthon, P. M.; Cummings, S. R.; Forman, D. E.; Goodpaster, B. H.; Harezlak, J.; Hawkins, M.; Kritchevsky, S. B.; Nicklas, B. J.; Toledo, F. G. S.; Toto, P. E.; Newman, A. B.; Glynn, N. W.	2024	Did not perform fiber type analysis
Sex differences in insulin regulation of skeletal muscle glycogen synthase and changes during weight loss and exercise in adults	Ryan, A. S.; Li, G.; McMillin, S.; Ortmeyer, H. K.	2024	Did not perform fiber type analysis
High-dose atorvastatin therapy progressively decreases skeletal muscle mitochondrial respiratory capacity in humans	Ryan, T. E.; Torres, M. J.; Lin, C. T.; Clark, A. H.; Brophy, P. M.; Smith, C. A.; Smith, C. D.; Morris, E. M.; Thyfault, J. P.; Neufer, P. D.	2024	Did not perform fiber type analysis
Sex-specific increases in myostatin and SMAD3 contribute to obesity-related insulin resistance in human skeletal muscle and primary human myotubes	Saxena, G.; Gallagher, S.; Law, T. D.; Maschari, D.; Walsh, E.; Dudley, C.; Brault, J. J.; Consitt, L. A.	2024	Did not perform fiber type analysis
Skeletal Muscle Mitochondrial Respiration and Exercise Intolerance in Patients With Heart Failure With Preserved Ejection Fraction	Scandalis, L.; Kitzman, D. W.; Nicklas, B. J.; Lyles, M.; Brubaker, P.; Nelson, M. B.; Gordon, M.; Stone, J.; Bergstrom, J.; Neufer, P. D.; Gnaiger, E.; Molina, A. J. A.	2023	Did not perform fiber type analysis
Skeletal muscle mitochondria demonstrate similar respiration per cristae surface area independent of training status and sex in healthy humans	Schytz, C. T.; Ã˜rtenblad, N.; Lundby, A. M.; Jacobs, R. A.; Nielsen, J.; Lundby, C.	2024	Did not perform fiber type analysis
Skeletal Muscle Involvement in PatientsÂ With Truncations of Titin and Familial Dilated Cardiomyopathy	Skriver, S. V.; Krett, B.; Poulsen, N. S.; Krag, T.; Walas, H. R.; Christensen, A. H.; Bundgaard, H.; Vissing, J.; Vissing, C. R.	2024	No healthy subjects or controls
Proteomic characterization of human LMNA-related congenital muscular dystrophy muscle cells	Storey, E. C.; Holt, I.; Brown, S.; Synowsky, S.; Shirran, S.; Fuller, H. R.	2024	Does not include males and females

Expression of mitochondrial oxidative stress response genes in muscle is associated with mitochondrial respiration, physical performance, and muscle mass in the Study of Muscle, Mobility, and Aging	Tranah, G. J.; Barnes, H. N.; Cawthon, P. M.; Coen, P. M.; Esser, K. A.; Hepple, R. T.; Huo, Z.; Kramer, P. A.; Toledo, F. G. S.; Zhang, X.; Wu, K.; Wolff, C. A.; Evans, D. S.; Cummings, S. R.	2024	Did not perform fiber type analysis
NASA SPRINT exercise program efficacy for vastus lateralis and soleus skeletal muscle health during 70 days of simulated microgravity	Trappe, T. A.; Minchev, K.; Perkins, R. K.; Lavin, K. M.; Jemiolo, B.; Ratchford, S. M.; Claiborne, A.; Lee, G. A.; Finch, W. H.; Ryder, J. W.; Ploutz-Snyder, L.; Trappe, S. W.	2024	Single Fiber Analysis
Algae Ingestion Increases Resting and Exercised Myofibrillar Protein Synthesis Rates to a Similar Extent as Mycoprotein in Young Adults	van der Heijden, I.; West, S.; Monteyne, A. J.; Finnigan, T. J. A.; Abdelrahman, D. R.; Murton, A. J.; Stephens, F. B.; Wall, B. T.	2023	Did not perform fiber type analysis
Percutaneous biopsies of skeletal muscle and adipose tissue in individuals older than 70: methods and outcomes in the Study of Muscle, Mobility and Aging (SOMMA)	Zamora, Z.; Lui, L. Y.; Sparks, L. M.; Justice, J.; Lyles, M.; Gentle, L.; Gregory, H.; Yeo, R. X.; Kershaw, E. E.; Stefanovic-Racic, M.; Newman, A. B.; Kritchevsky, S.; Toledo, F. G. S.	2024	Did not perform fiber type analysis
Transcriptome profiling of fast/glycolytic and slow/oxidative muscle fibers in aging and obesity	Zhang, F. M.; Wu, H. F.; Wang, K. F.; Yu, D. Y.; Zhang, X. Z.; Ren, Q.; Chen, W. Z.; Lin, F.; Yu, Z.; Zhuang, C. L.	2024	Animal study
Skeletal muscle-specific DJ-1 ablation-induced atrogenes expression and mitochondrial dysfunction contributing to muscular atrophy	Zhang, S.; Yan, H.; Ding, J.; Wang, R.; Feng, Y.; Zhang, X.; Kong, X.; Gong, H.; Lu, X.; Ma, A.; Hua, Y.; Liu, H.; Guo, J.; Gao, H.; Zhou, Z.; Wang, R.; Chen, P.; Liu, T.; Kong, X.	2023	No healthy subjects or controls
Effects of hyperinsulinemia and hyperglycemia on insulin receptor function and glycogen synthase activation in skeletal muscle of normal man	Bak, J. F.; Moller, N.; Schmitz, O.; Richter, E. A.; Pedersen, O.	1991	Did not perform fiber type analysis
Comparison of two Î²alanine dosing protocols using a sustained release formulation on muscle carnosine elevations	Church, D. D.; Hoffman, J. R.; Varanoske, A. N.; Wang, R.; Baker, K.; La Monica, M. B.; Beyer, K. S.; Dodd, S. J.; Oliveira, L. P.; Fukuda, D. H.; et al.,	2017	Did not perform fiber type analysis
Autophagy response to acute high-intensity interval training and moderate-intensity continuous training is dissimilar in skeletal muscle and peripheral blood mononuclear cells and is influenced by sex	Escobar, K. A.; Welch, A. M.; Wells, A.; Fennel, Z.; Nava, R.; Li, Z.; Moriarty, T. A.; Nitta, C. H.; Zuhl, M. N.; VanDusseldorp, T. A.; et al.,	2021	Did not perform fiber type analysis
Responses of human skeletal muscle fibers to a 370-day antiorthostatic hypokinesis associated with physical exercise	Kuznetsov, S. L.; Stepantsov, V. V.	1990	Not in English
Combined polyphenols increase fat oxidation and mitochondrial oxidative capacity but do not improve peripheral and hepatic insulin sensitivity in healthy overweight men and women	Most, J.; Timmers, S.; Jocken, J. W.; Schrauwen, P.; Goossens, G. H.; Blaak, E. E.	2015	Not peer reviewed (ex: thesis, conference poster)
Human muscle protein synthetic responses during weight-bearing and non-weight-bearing exercise: a comparative study of exercise modes and recovery nutrition	Pasiakos, S. M.; McClung, H. L.; Margolis, L. M.; Murphy, N. E.; Lin, G. G.; Hydren, J. R.; Young, A. J.	2015	Did not perform fiber type analysis

Underpinning the Food Matrix Regulation of Postexercise Myofibrillar Protein Synthesis by Comparing Salmon Ingestion With the Sum of Its Isolated Nutrients in Healthy Young Adults	Paulussen, K. J.; Barnes, T. M.; Askow, A. T.; Salvador, A. F.; McKenna, C. F.; Scaroni, S. E.; Fliflet, A.; Ulanov, A. V.; Li, Z.; West, D. W.; et al.,	2023	Did not perform fiber type analysis
Effects of 12 Months of Caloric Restriction on Muscle Mitochondrial Function in Healthy Individuals	Sparks, L. M.; Redman, L. M.; Conley, K. E.; Harper, M. E.; Yi, F.; Hodges, A.; Eroshkin, A.; Costford, S. R.; Gabriel, M. E.; Shook, C.; et al.,	2017	Did not perform fiber type analysis
Skeletal muscle of young females under resistance exercise exhibits a unique innate immune cell infiltration profile compared to males and elderly individuals	Castrogiovanni, P.; Sanfilippo, C.; Imbesi, R.; Lazzarino, G.; Li Volti, G.; Tibullo, D.; Vicario, N.; Parenti, R.; Giuseppe, L.; Barbagallo, I.; Alanazi, A. M.; Vecchio, M.; Cappello, F.; Musumeci, G.; Di Rosa, M.	2024	Did not perform fiber type analysis
Peroxisome proliferator-activated receptors delta content is increased and associated with altered serum lipid profile with resistance exercise training	Chen, V. C. W.; Lee, C. W.; Bui, S.; Lee, T. V.; Fluckey, J. D.; Riechman, S. E.	2023	Did not perform fiber type analysis
A multimodal exercise countermeasure prevents the negative impact of head-down tilt bed rest on muscle volume and mitochondrial health in older adults	Dulac, M.; Hajj-Boutros, G.; Sonjak, V.; Faust, A.; Hussain, S. N. A.; Chevalier, S.; Dionne, I. J.; Morais, J. A.; Gouspillou, G.	2024	Did not perform fiber type analysis
Effects of Weight Loss and Aerobic Exercise Training on Adi-Pose Tissue Zinc Î±2-Glycoprotein and Associated Genes in Obesity	Ge, S. X.; Li, G. Y.; Ryan, A. S.	2023	Did not perform fiber type analysis
IL-6 and TNF are Potential Inflammatory Biomarkers in Facioscapulohumeral Muscular Dystrophy	Greco, A.; Mul, K.; Jaeger, M. H.; dos Santos, J. C.; Koenen, H.; de Jong, L.; Mann, R.; FÃ¼terer, J.; Netea, M. G.; Pruijn, G. J. M.; van Engelen, B. G. M.; Joosten, L. A. B.	2024	Did not perform fiber type analysis
HETEROPLASMY-ASSOCIATED MITOCHONDRIAL DNA VARIANTS IN HUMAN BLOOD AND SKELETAL MUSCLE SAMPLES	GÃ¼lec, Ã‡; Gedikbasi, A.; Sahin, G.; Toksoy, G.; Duramaz, A.; Uyguner, Z. O.	2024	Did not perform fiber type analysis
The Resistance Training Effects on Skeletal Muscle Stem Cells in Older Adult: A Systematic Review and Meta-Analysis	Heidari, D.; Shirvani, H.; Bazgir, B.; Shamsoddini, A.	2023	Review (ex: systematic review/meta-analysis)
High-Intensity Training Represses FXYD5 and Glycosylates Na,K-ATPase in Type II Muscle Fibres, Which Are Linked with Improved Muscle K⁺ Handling and Performance	Hostrup, M.; Lemminger, A. K.; Thomsen, L. B.; Schaufuss, A.; Alsoe, T. L.; Bergen, G. K.; Bell, A. B.; Bangsbo, J.; Thomassen, M.	2023	Single Fiber Analysis
Sex differences in muscle protein expression and DNA methylation in response to exercise training	Landen, S.; Jacques, M.; Hiam, D.; Alvarez-Romero, J.; Schittenhelm, R. B.; Shah, A. D.; Huang, C.; Steele, J. R.; Harvey, N. R.; Haupt, L. M.; Griffiths, L. R.; Ashton, K. J.; Lamon, S.; Voisin, S.; Eynon, N.	2023	Does not include male and female data
Influence of Frequent Corticosteroid Local Injections on the Expression of Genes and Proteins Related to Fatty Infiltration, Muscle Atrophy, Inflammation, and Fibrosis in Patients With Chronic Rotator Cuff Tears: A Pilot Study	Lee, J.; Lho, T.; Lee, J.; Lee, J.; Chung, S. W.	2024	Did not perform fiber type analysis
Glucose ingestion before and after resistance training sessions does not augment ribosome	Lian, K. S.; HammarstrÃ¶m, D.; Hamarsland, H.; Molmen, K. S.; Moen, S. C.; Ellefsen, S.	2024	Did not perform fiber type analysis

biogenesis in healthy moderately trained young adults			
Slow or fast: Implications of myofibre type and associated differences for manifestation of neuromuscular disorders	Lloyd, E. M.; Pinniger, G. J.; Murphy, R. M.; Grounds, M. D.	2023	Review (ex: systematic review/meta-analysis)
Mitochondrial Complex Abundance, Mitophagy Proteins, and Physical Performance in People With and Without Peripheral Artery Disease	Picca, A.; Wohlgemuth, S. E.; McDermott, M. M.; Saini, S. K.; Dayanidhi, S.; Zhang, D. X.; Xu, S. J.; Kosmac, K.; Tian, L.; Ferrucci, L.; Sufit, R. L.; Marzetti, E.; Leeuwenburgh, C.	2023	Did not perform fiber type analysis
Sex differences in muscle SIRT1 and SIRT3 and exercise plus weight loss effects on muscle sirtuins	Ryan, A. S.; Li, G. Y.	2023	Did not perform fiber type analysis
Ingestion of mycoprotein, pea protein, and their blend support comparable postexercise myofibrillar protein synthesis rates in resistance-trained individuals	West, S.; Monteyne, A. J.; Whelehan, G.; van der Heijden, I.; Abdelrahman, D. R.; Murton, A. J.; Finnigan, T. J. A.; Stephens, F. B.; Wall, B. T.	2023	Did not perform fiber type analysis
