# Supplementary material for: Sex differences in human skeletal muscle fiber types and the influence of age, physical activity, and muscle group: A systematic review and meta‐analysis
Source: Physiol Rep. 2025 Nov 2;13(21):e70616. doi: 10.14814/phy2.70616 (PMC12580412; doi:10.14814/phy2.70616)
Supplement: Supplementary file 4 — Data S4. Table of included studies and characteristics. [file PHY2-13-e70616-s001.pdf]

Characteristics of Included Studies Table

Ref #	First Author, Year, Description	Total Sample Size	Subset Sample Size	# of Males and Females (M:F)	Age Group	Physical Activity Level Group	Muscle Biopsied Group	Fiber Type Analysis Method	Risk of Bias Group
131	Messa_2020_Young_Non_Athletes	157	22	14:8	Young	-	Leg (VL)	mATPase	🟡
131	Messa_2020_Old_Non_Athletes	157	35	27:8	Older	-	Leg (VL)	mATPase	🟡
131	Messa_2020_Mid_Life_Track_And_Field_Athletes	157	51	44:7	Midlife	NS	Leg (VL)	mATPase	🟡
131	Messa_2020_Old_Track_And_Field_Athletes	157	35	32:3	Older	NS	Leg (VL)	mATPase	🟡
172	Terzis_2009	16	N/A	8:8	Young	NS	Leg (VL)	mATPase	🟡
163	Sorensen_2018	11	N/A	7:4	Young	NS	Leg (VL)	IHC	🟡
152	Rolf_1997_National_Orienteers	30	12	5:7	Young	NS	Leg (VL)	mATPase	🔴
152	Rolf_1997_Upcoming_Orienteers	30	18	8:10	Young	NS	Leg (RF)	mATPase	🔴
125	Martel_2006_Baseline_Young_Untrained_Leg	40	22	13:9	Young	S	Leg (VL)	mATPase	🟢
125	Martel_2006_Baseline_Young_Trained_Leg	40	22	13:9	Young	S	Leg (VL)	mATPase	🟢
125	Martel_2006_Baseline_Old_Untrained_Leg	40	18	11:7	Older	S	Leg (VL)	mATPase	🟢
125	Martel_2006_Baseline_Old_Trained_Leg	40	18	11:7	Older	S	Leg (VL)	mATPase	🟢
77	Fayet_2001_Age_50_To_59	51	16	8:8	Midlife	S	A/Sh (D)	mATPase	🟡
77	Fayet_2001_Age_60_To_69	51	15	7:8	Older	S	A/Sh (D)	mATPase	🟡
77	Fayet_2001_Age_70_To_79	51	20	11:9	Older	S	A/Sh (D)	mATPase	🟡
133	Miller_2013_Young	24	12	5:7	Young	NS	Leg (VL)	Homogenates	🟡
133	Miller_2013_Old	24	12	5:7	Older	NS	Leg (VL)	Homogenates	🟡
149	Ringqvist_1974	13	N/A	8:5	Midlife	-	Head (T)	mATPase	🔴
178	Varesco_2022	30	N/A	15:15	Older	NS	Leg (VL)	IHC	🟡
91	Green_2000	17	N/A	8:9	Midlife	S	Leg (VL)	Homogenates	🟡
162	Sjogaard_1982_Triceps_Brachii	12	12	6:6	Young	NS	A/Sh (TB)	mATPase	🔴
162	Sjogaard_1982_Vastus_Lateralis	12	12	6:6	Young	NS	Leg (VL)	mATPase	🔴

162	Sjogaard_1982_Soleus	12	12	6:6	Young	NS	Leg (S)	mATPase	
147	Porter_2002	30	N/A	15:15	Young	NS	Leg (TA)	mATPase	
92	Green_2015_Extensor_Carpi_Radialis_Brevis	36	19	10:9	Young	NS	A/Sh (ECRB)	mATPase	
92	Green_2015_Trapezius	36	17	10:7	Young	NS	B/Tr (Trap)	mATPase	
52	Cairns_2017	10	N/A	5:5	Young	NS	Leg (VL)	mATPase	
97	Hakkinen_2001_Mid_Life	42	21	10:11	Midlife	NS	Leg (VL)	mATPase	
97	Hakkinen_2001_Old	42	21	11:10	Older	NS	Leg (VL)	mATPase	
138	Norman_2014	138†	N/A	83:55	Young	NS	Leg (VL)	mATPase	
70	Esbjornsson_2012	16*	N/A	8:8	Young	NS	Leg (VL)	mATPase	
75	Esbjornsson-Liljedahl_1999	39*	N/A	20:19	Young	NS	Leg (VL)	mATPase	
88	Glenmark_1992	83†	N/A	55:28	Young	NS	Leg (VL)	mATPase	
87	Glenmark_1994_A	81†	N/A	55:26	Young	NS	Leg (VL)	mATPase	
89	Glenmark_1994_B	83†	N/A	55:28	Young	NS	Leg (VL)	mATPase	
158	Sharman_2001_Baseline_Control_Group	20	6	3:3	Midlife	NS	Leg (VL)	Homogenates	
158	Sharman_2001_Baseline_Experimental_Group	20	14	7:7	Older	NS	Leg (VL)	Homogenates	
54	Carter_2001	16	N/A	8:8	Young	S	Leg (VL)	mATPase	
51	Burke_1977	29	N/A	22:7	Young	NS	Leg (VL)	mATPase	
86	Gerdle_1998	20	N/A	11:9	Young	-	Leg (VL)	mATPase	
183	Whitman_2005_Young	42	21	10:11	Young	S	Leg (VL)	mATPase	
183	Whitman_2005_Old	42	21	11:10	Older	S	Leg (VL)	mATPase	
28	Fry_1994_Baseline_Control_Group	33	12	7:5	Young	NS	Leg (VL)	mATPase and Homogenates	
28	Fry_1994_Baseline_Experimental_Group	33	21	13:8	Young	NS	Leg (VL)	mATPase and Homogenates	
44	Barnouin_2017_Young	47	19	14:5	Young	NS	Leg (VL)	IHC	
44	Barnouin_2017_Old	47	28	22:6	Older	NS	Leg (VL)	IHC	
50	Brose_2003_Baseline_Experimental_Group	28	14	8:6	Older	-	Leg (VL)	mATPase	
50	Brose_2003_Baseline_Control_Group	28	14	7:7	Older	-	Leg (VL)	mATPase	
65	Duscha_2001	21	N/A	10:11	Midlife	S	Leg (VL)	Homogenates	
69	Lijedahl_1996	16	N/A	6:10	Young	NS	Leg (VL)	mATPase	

98	Hakkinen_2002_Baseline_Experimental_Group	31	21	10:11	Older	NS	Leg (VL)	mATPase	🔴
98	Hakkinen_2002_Baseline_Control_Group	31	10	5:5	Older	NS	Leg (VL)	mATPase	🔴
38	Alway_1992	4	N/A	2:2	Young	-	A/Sh (BB)	mATPase	🟡
111	Kosek_2006_Young	49	24	13:11	Young	-	Leg (VL)	IHC	🟢
111	Kosek_2006_Old	49	25	13:12	Older	-	Leg (VL)	IHC	🟢
108	Jaworowski_2002	30	N/A	15:15	Young	NS	Leg (TA)	mATPase	🟢
144	Owerkowicz_2016	17	N/A	8:9	Young	-	Leg (VL)	Homogenates	🟡
156	Serrano_2019	15	15	6:9	Young	NS	Leg (VL)	Homogenates	🟡
71	Esbjornsson_1993	34	N/A	18:16	Young	NS	Leg (VL)	mATPase	🟡
165	Staron_2000	150*	N/A	95:55	Young	NS	Leg (VL)	mATPase and Homogenates	🟡
68	Engelhardt_2022	150*	N/A	95:55	Young	NS	Leg (VL)	mATPase and Homogenates	🟡
123	Mannion_1997	21	N/A	12:9	Young	NS	B/Tr (LRES)	mATPase	🟡
173	Thorstensson_1987_Lumbar_Multifidus	16	16	9:7	Young	-	B/Tr (LM)	mATPase	🟡
173	Thorstensson_1987_Lumbar_Longissimus	16	16	9:7	Young	-	B/Tr (LL)	mATPase	🟡
175	Torres_2011	26	N/A	9:17	Midlife	-	Leg (VL)	mATPase	🟡
43	Bamman_2003	14	N/A	9:5	Older	-	Leg (VL)	mATPase and Homogenates	🟡
132	Miller_1993_Biceps_Brachii	16	16	8:8	Young	-	A/Sh (BB)	mATPase	🟡
132	Miller_1993_Vastus_Lateralis	16	16	8:8	Young	-	Leg (VL)	mATPase	🟡
49	Bouchard_1986_Dizygotic_Twins	122	52	32:20	Young	-	Leg (VL)	mATPase	🟡
49	Bouchard_1986_Monozygotic_Twins	122	70	38:32	Young	-	Leg (VL)	mATPase	🟡
113	Kumagai_2018	211†	N/A	102:109	Midlife	S	Leg (VL)	Homogenates	🟡
171	Takaragawa_2021	214†	N/A	107:107	Midlife	S	Leg (VL)	Homogenates	🟡
188	Yvert_2020	214†	N/A	107:107	Midlife	S	Leg (VL)	Homogenates	🟡
94	Guadalupe-Grau_2016_A	35	N/A	25:10	Young	-	Leg (VL)	mATPase and Homogenates	🟡
117	Lexell_1995_Biceps_Brachii	20	20	12:8	Older	NS	A/Sh (BB)	mATPase	🔴

117	Lexell_1995_Vastus_Lateralis	20	20	12:8	Older	NS	Leg (VL)	mATPase	🔴
151	Roepstorff_2006	17*	N/A	8:9	Young	NS	Leg (VL)	mATPase	🟢
103	Hoeg_2009	16*	N/A	8:8	Young	NS	Leg (VL)	mATPase	🟢
57	Coggan_1992_Young	40	20	10:10	Young	NS	Leg (G)	mATPase	🟡
57	Coggan_1992_Old	40	20	10:10	Older	NS	Leg (G)	mATPase	🟡
76	Essén-Gustavsson_1986_Age_20	64*	9	4:5	Young	-	Leg (VL)	mATPase	🟡
76	Essén-Gustavsson_1986_Age_30	64	10	6:4	Young	-	Leg (VL)	mATPase	🟡
76	Essén-Gustavsson_1986_Age_40	64	12	6:6	Midlife	-	Leg (VL)	mATPase	🟡
76	Essén-Gustavsson_1986_Age_50	64	11	5:6	Midlife	-	Leg (VL)	mATPase	🟡
76	Essén-Gustavsson_1986_Age_60	64	11	5:6	Older	-	Leg (VL)	mATPase	🟡
76	Essén-Gustavsson_1986_Age_70	64*	11	7:4	Older	-	Leg (VL)	mATPase	🟡
48	Borges_1989_Ages_20_to_40	22*	12	7:5	Young	-	Leg (VL)	mATPase	🟡
48	Borges_1989_Age_70	22*	10	7:3	Older	-	Leg (VL)	mATPase	🟡
66	Edstrom_1969	6	N/A	2:4	Young	-	Leg (VL)	mATPase	🔴
150	Roberts_2018_Age_20_To_29	221	47	21:26	Young	-	Leg (VL)	IHC	🟢
150	Roberts_2018_Age_30_To_50	221	79	45:34	Midlife	-	Leg (VL)	IHC	🟢
150	Roberts_2018_Age_55_To_64	221	51	19:32	Midlife	-	Leg (VL)	IHC	🟢
150	Roberts_2018_Age_65_To_81	221	44	17:27	Older	-	Leg (VL)	IHC	🟢
159	Simoneau_1989	418*	N/A	215:203	Young	NS	Leg (VL)	mATPase	🟡
160	Simoneau_1995	348*	N/A	199:149	Young	NS	Leg (VL)	mATPase	🟡
64	Dial_2021_Young	31	13	6:7	Young	S	Leg (VL)	IHC	🟢
64	Dial_2021_Mid_Life	31	18	7:11	Midlife	S	Leg (VL)	IHC	🟢
106	Horwath_2021	64	N/A	34:30	Young	NS	Leg (VL)	IHC	🟡
78	Flueck_2011	20	N/A	11:9	Older	NS	Leg (VL)	IHC	🟡
39	Apple_1986_A	15*	N/A	8:7	Young	NS	Leg (G)	mATPase	🟡
40	Apple_1986_B	14*	N/A	7:7	Young	NS	Leg (G)	mATPase	🔴
140	Nygaard_1983	8	N/A	4:4	Young	-	A/Sh (BB)	mATPase	🟡
182	Wens_2014	18	N/A	5:13	Midlife	-	Leg (VL)	mATPase	🟡
46	Bell_1990_Body_Builders	19	9	5:4	Young	NS	Leg (VL)	mATPase	🟡
46	Bell_1990_Controls	19	10	4:6	Young	-	Leg (VL)	mATPase	🟡
129	McGuigan_2001	8	N/A	4:4	Older	S	Leg (G)	mATPase and Homogenates	🟡
153	Ryushi_1988	19	N/A	9:10	Young	NS	Leg (VL)	mATPase	🟡
72	Esbjornsson_2021	16	N/A	9:10	Young	NS	Leg (VL)	mATPase	🟡

122	Mannion_1997_Thoracic_Region_Erector_Spinae	27	27	16:11	Young	NS	B/Tr (TRES)	mATPase	🟡
122	Mannion_1997_Lumbar_Region_Erector_Spinae	27	27	16:11	Young	NS	B/Tr (LRES)	mATPase	🟡
155	Schantz_1983_Vastus_Lateralis	18	18	11:7	Young	NS	Leg (VL)	mATPase	🔴
155	Schantz_1983_Triceps_Brachii	18	18	11:7	Young	NS	A/Sh (TB)	mATPase	🔴
184	Wiles_1979	32	N/A	24:8	Young	-	Leg (VL)	mATPase	🟡
102	Herda_2019	22	N/A	11:11	Young	S	Leg (VL)	Homogenates	🟡
166	Steffensen_2002_Untrained	42	14	7:7	Young	S	Leg (VL)	mATPase	🟡
166	Steffensen_2002_Moderately_Trained	42	14	7:7	Young	NS	Leg (VL)	mATPase	🟡
166	Steffensen_2002_Endurance_Trained	42	14	7:7	Young	NS	Leg (VL)	mATPase	🟡
134	Moesgaard_2022	24	N/A	12:12	Young	-	Leg (VL)	IHC	🟡
120	Machek_2020_Power_Lifters	22	12	6:6	Young	NS	Leg (VL)	Homogenates	🟡
120	Machek_2020_Controls	22	10	5:5	Young	S	Leg (VL)	Homogenates	🟡
135	Molsted_2007	12	N/A	9:3	Midlife	-	Leg (VL)	mATPase and Homogenates	🟡
145	Paoli_2013	18	N/A	9:9	Young	NS	B/Tr (LD)	Homogenates	🟡
181	Walker_2012_Young	22	10	5:5	Young	NS	Leg (VL)	IHC	🟡
181	Walker_2012_Old	22	11	6:5	Older	NS	Leg (VL)	IHC	🟡
100	Hall_2021	30	N/A	20:10	Young	NS	Leg (VL)	IHC	🟡
146	Pollock_2018	90	N/A	60:30	Older	NS	Leg (VL)	mATPase	🟡
116	Leenders_2013_Baseline_Control_Group	53	26	14:12	Older	S	Leg (VL)	IHC	🟡
116	Leenders_2013_Baseline_Experimental_Group	53	27	15:12	Older	S	Leg (VL)	IHC	🟡
174	Toft_2003_Midlife_Younger_Group	91	40	22:18	Midlife	-	Leg (VL)	mATPase	🟡
174	Toft_2003_Midlife_Older_Group	91	51	36:15	Midlife	-	Leg (VL)	mATPase	🟡
185	Williamson_2001	12	N/A	6:6	Young	-	Leg (VL)	Homogenates	🟡
137	Moro_2020	19	N/A	10:9	Older	-	Leg (VL)	IHC	🟡
141	O'Hagan_1995	12	N/A	6:6	Young	NS	A/Sh (BB)	mATPase	🟡
110	Kim_2005_Young	38	20	10:10	Young	-	Leg (VL)	IHC	🟢

110	Kim_2005_Old	38	18	9:9	Older	-	Leg (VL)	IHC	🟢
35	Abou_Sawan_2021	20	N/A	10:10	Young	NS	Leg (VL)	IHC	🟢
47	Binet_2023	28	N/A	13:15	Midlife	-	Leg (VL)	IHC	🟢
186	Yasuda_2005	27*	N/A	13:14	Young	NS	Leg (VL)	mATPase and Homogenates	🟡
121	Maher_2009	26*	N/A	10:16	Young	NS	Leg (VL)	mATPase	🟡
74	Esbjornsson-Liljedahl_1998	32	N/A	16:16	Young	NS	Leg (VL)	mATPase	🟡
41	Ausin_2017	15	N/A	7:8	Older	-	Leg (VL)	IHC	🟡
11	Staron_1994_Baseline_Experimental_Group	33	21	13:8	Young	-	Leg (VL)	mATPase	🟡
11	Staron_1994_Baseline_Control_Group	33	12	7:5	Young	S	Leg (VL)	mATPase	🟡
107	Hostler_2001	26	N/A	10:16	Young	S	Leg (VL)	mATPase and Homogenates	🟡
58	Coggan_1992	23	N/A	12:11	Older	S	Leg (G)	mATPase	🟡
59	Costill_1976_Sprinters	54	4	2:2	Young	NS	Leg (G)	mATPase	🟡
59	Costill_1976_Middle_Distance_Runners	54	14	7:7	Young	NS	Leg (G)	mATPase	🟡
59	Costill_1976_Jumpers	54	5	2:3	Young	NS	Leg (G)	mATPase	🟡
59	Costill_1976_Javelin_Throwers	54	6	3:3	Young	NS	Leg (G)	mATPase	🟡
59	Costill_1976_Discus_Shot_Put_Throwers	54	6	4:2	Young	NS	Leg (G)	mATPase	🟡
59	Costill_1976_Untrained	54	21	21:21	Young	S	Leg (G)	mATPase	🟡
161	Simoneau_1985	75	N/A	37:38	Young	S	Leg (VL)	mATPase	🟡
42	Bailly_2020	31	N/A	15:16	Young	-	Leg (VL)	IHC	🟡
83	Gerard_1987	8	N/A	4:4	Young	NS	Leg (VL)	mATPase	🟡
73	Esbjornsson-Liljedahl_2002	15	N/A	7:8	Young	NS	Leg (VL)	mATPase	🟡
112	Kuipers_1989	29	N/A	21:8	Young	NS	Leg (VL)	mATPase	🟡
105	Holmback_2003	30	N/A	15:15	Young	NS	Leg (TA)	mATPase and Homogenates	🟡
114	Larsson_2006	52	N/A	9:43	Young	NS	Leg (G)	mATPase and IHC	🟡
130	McPhee_2018_Young	71	31	16:15	Young	-	Leg (VL)	mATPase	🟡
130	McPhee_2018_Old	71	40	20:20	Older	-	Leg (VL)	mATPase	🟡
36	Ahmetov_2011	34	N/A	20:14	Young	NS	Leg (VL)	IHC	🟡
127	Mazis_2009	17	N/A	9:8	Midlife	NS	B/Tr (LM)	Histochemistry -H&E	🟡

84	Gerdle_2000	20*	20	11:9	Young	-	Leg (VL)	mATPase	
85	Gerdle_1997	20*	20	11:9	Young	-	Leg (VL)	mATPase	
187	Young_1984	13	N/A	9:4	Young	-	Leg (VL)	mATPase	
79	Froese_1985	30	N/A	12:18	Young	-	Leg (VL)	mATPase	
154	Sale_1987	21	N/A	13:8	Young	-	A/Sh (BB)	mATPase	
104	Hoeg_2013	38	N/A	23:15	Young	NS	Leg (VL)	mATPase	
100	Hall_2021_Endurance_Trained	164	51	37:14	Young	NS	Leg (VL)	IHC	
100	Hall_2021_Power_Trained	164	48	33:15	Young	NS	Leg (VL)	IHC	
100	Hall_2021_Controls	164	65	40:25	Young	-	Leg (VL)	IHC	
90	Goedecke_2000	61	N/A	45:16	Young	NS	Leg (VL)	mATPase	
61	Dastmalchi_2007	11	N/A	5:6	Midlife	NS	Leg (VL)	mATPase	
115	Leenders_2013	53	N/A	29:24	Older	-	Leg (VL)	IHC	
60	Dahlstrom_1997	13	N/A	6:7	Young	NS	Leg (VL)	mATPase	
148	Ricoy_1998	82	N/A	66:16	Young	NS	Leg (VL)	mATPase	
109	Karlsen_2019	29	N/A	18:11	Older	-	Leg (VL)	IHC	
53	Calsbeek_2002	24	N/A	13:11	Young	NS	Leg (VL)	mATPase	
124	Marin_1994_Females_Low_Waist_To_Hip_Ratio_And_Males	89*	70	48:22	Midlife	-	Leg (VL)	mATPase	
124	Marin_1994_Females_High_Waist_To_Hip_Ratio	89*	67	48:19	Midlife	-	Leg (VL)	mATPase	
45	Bell_1989_Trained	19	9	5:4	Young	NS	Leg (VL)	mATPase	
45	Bell_1989_Untrained	19	10	4:6	Young	-	Leg (VL)	mATPase	
170	Suter_1993	31	N/A	24:7	Young	-	Leg (VL)	mATPase	
136	Montero_2018	24	N/A	12:12	Young	NS	Leg (VL)	mATPase	
101	He_2001_Lean	42	22	11:11	Midlife	S	Leg (VL)	mATPase	
101	He_2001_Obese	42	20	14:6	Midlife	S	Leg (VL)	mATPase	
56	Churchward-Venne_2015	110	N/A	66:44	Older	-	Leg (VL)	IHC	
164	Stalberg_1989	65	N/A	34:31	Midlife	-	Leg (VL)	mATPase	
139	Norman_2009	120	N/A	62:59	Young	NS	Leg (VL)	mATPase	
63	Den-Hoed_2009	38	N/A	7:31	Young	-	Leg (VL)	IHC	
93	Guadalupe-Grau_2016_B_Baseline_Control_Group	48	26	15:11	Young	NS	Leg (VL)	Homogenates	
93	Guadalupe-Grau_2016_B_Baseline_Experimental_Group	48	22	16:16	Young	-	Leg (VL)	Homogenates	

142	Oh_2018_Young	53	28	17:11	Young	-	Leg (VL)	Homogenates	🟡
142	Oh_2018_Old	53	25	15:10	Older	-	Leg (VL)	Homogenates	🟡
176	Trevino_2019	20	N/A	10:10	Young	S	Leg (VL)	Homogenates	🟡
95	Guiherme_2020_Power_Traine d	164	56	36:20	Young	NS	Leg (VL)	IHC	🟡
95	Guiherme_2020_Endurance_Tr ained	164	108	77:31	Young	NS	Leg (VL)	IHC	🟡
126	Maunder-Sewry_1980	10	N/A	6:4	Young	-	Leg (VL)	mATPase	🔴
118	Liegnell_2020	16	N/A	8:8	Young	NS	Leg (VL)	Homogenates	🟡
96	Guiherme_2022_Power_Traine d	148	47	28:19	Young	NS	Leg (VL)	IHC	🟡
96	Guiherme_2022_Endurance_Tr ained	148	101	73:28	Young	NS	Leg (VL)	IHC	🟡
37	Ahmetov_2009_Sprint_Skaters	34	17	10:7	Young	NS	Leg (VL)	IHC	🟡
37	Ahmetov_2009_Endurance_Sk aters	34	17	10:7	Young	NS	Leg (VL)	IHC	🟡
179	Vescovo_1996	7	N/A	4:3	Midlife	-	Leg (G)	Homogenates	🟡
169	Suominen_1977	32	N/A	18:14	Older	S	Leg (VL)	mATPase	🔴
55	Caswell_2024	24	N/A	12:12	Young	NS	Leg (VL)	Homogenates	🟢
80	Galvan-Alvarez_2023_A	43†	N/A	30:13	Young	NS	Leg (VL)	Homogenates	🟡
81	Galvan-Alvarez_2024	61†	N/A	51:10	Young	NS	Leg (VL)	Homogenates	🟡
82	Galvan-Alvarez_2023_B	189†	N/A	120:49	Young	S	Leg (VL)	Homogenates	🟡
128	McDougall_2023	24	N/A	12:12	Young	NS	Leg (VL)	Homogenates	🟡
143	Olmos_2023	22	N/A	11:11	Young	S	Leg (VL)	Homogenates	🟡
62	DeJong_2023_Fittest	32	16	8:8	Older	-	Leg (VL)	IHC	🟡
62	DeJong_2023_Weakest	32	16	8:8	Older	-	Leg (VL)	IHC	🟡
177	Van-Vossel_2024	21	N/A	11:10	Young	NS	Leg (VL)	IHC	🟡
167	Sterczala_2024	33	N/A	19:14	Young	NS	Leg (VL)	IHC	🔴
157	Shadiow_2023	19	N/A	11:8	Young	NS	Leg (VL)	IHC	🔴
180	Vikmoen_2024	18	N/A	10:8	Young	NS	Leg (VL)	IHC	🟡
119	Lilja_2023	31	N/A	17:14	Young	NS	Leg (VL)	IHC	🟡
67	Engelbrecht_2024	24	N/A	12:12	Young	NS	Leg (VL)	mATPase	🟡
168	Sunnehagen_2000	69	69	40:29	Midlife	-	Leg (VL)	mATPase	🟡

If a first author published more than one article per year, then then first article published is marked as A and the second article published is marked as B; *: signifies when an article had duplicate data as another article and the participant sample size had to be split in half; †: signifies

when an article had duplicate data as two other articles and the participant sample size had to be split in thirds; S: sedentary; NS: not sedentary; -: physical activity level not described; A/Sh: Arm/Shoulder muscle biopsied group; B/Tr: back/trunk muscle biopsied group; VL: Vastus Lateralis; G: Gastrocnemius; D: Deltoid; BB: Biceps Brachii; TB: Triceps Brachii; TA: Tibialis Anterior; RF: Rectus Femoris; S: Soleus; T: Temporal; LM: Lumbar Multifidus; LL: Lumbar Longissimus; ECRB: Extensor Carpi Radialis Brevis; Trap: Trapezius; LRES: Lumbar Region Erector Spinae; TRES: Thoracic Region Erector Spinae; LD: Latissimus Dorsi; 🟢: risk of bias score = 6-7 points (good); 🟡; risk of bias score = 4-5 points (satisfactory); 🔴: risk of bias score = 0-3 points (unsatisfactory).
