# Supplementary figures and images for: Sex differences in human skeletal muscle fiber types and the influence of age, physical activity, and muscle group: A systematic review and meta‐analysis
Source: Physiol Rep. 2025 Nov 2;13(21):e70616. doi: 10.14814/phy2.70616 (PMC12580412; doi:10.14814/phy2.70616)

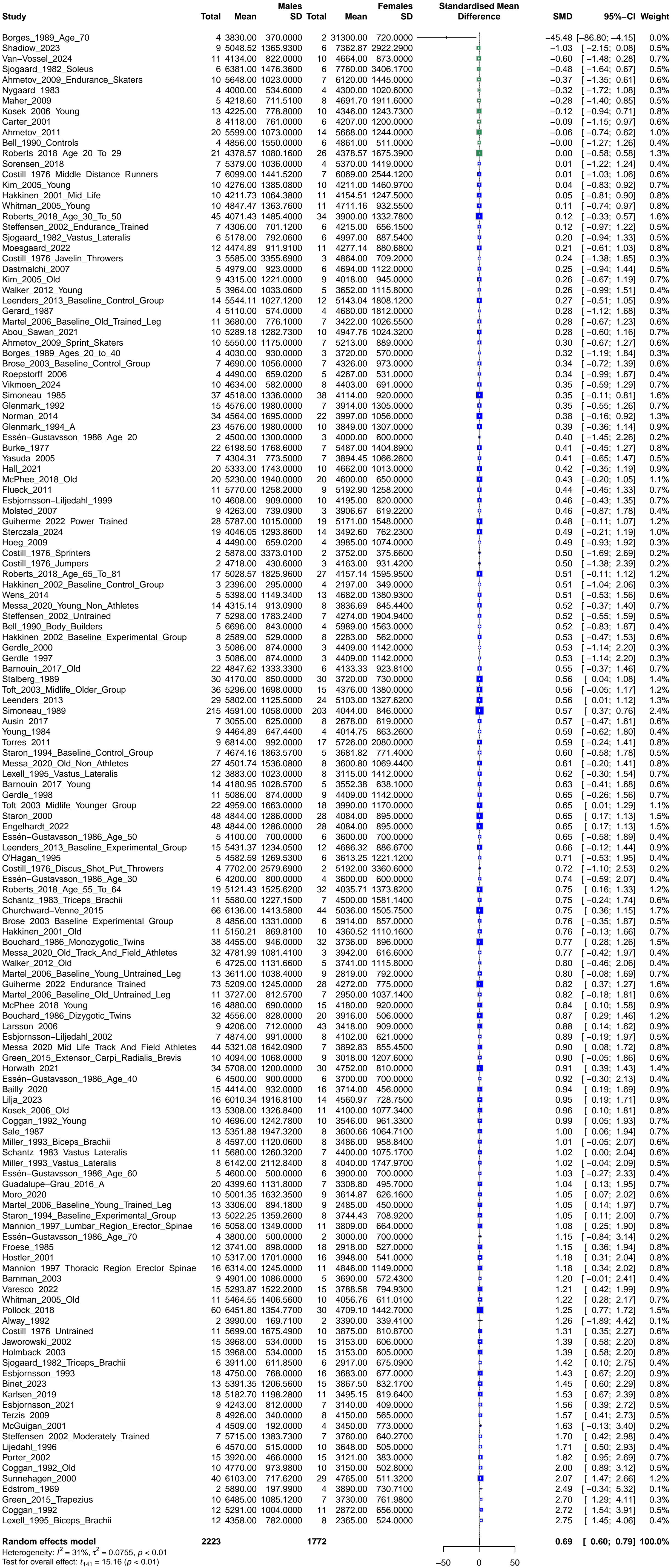



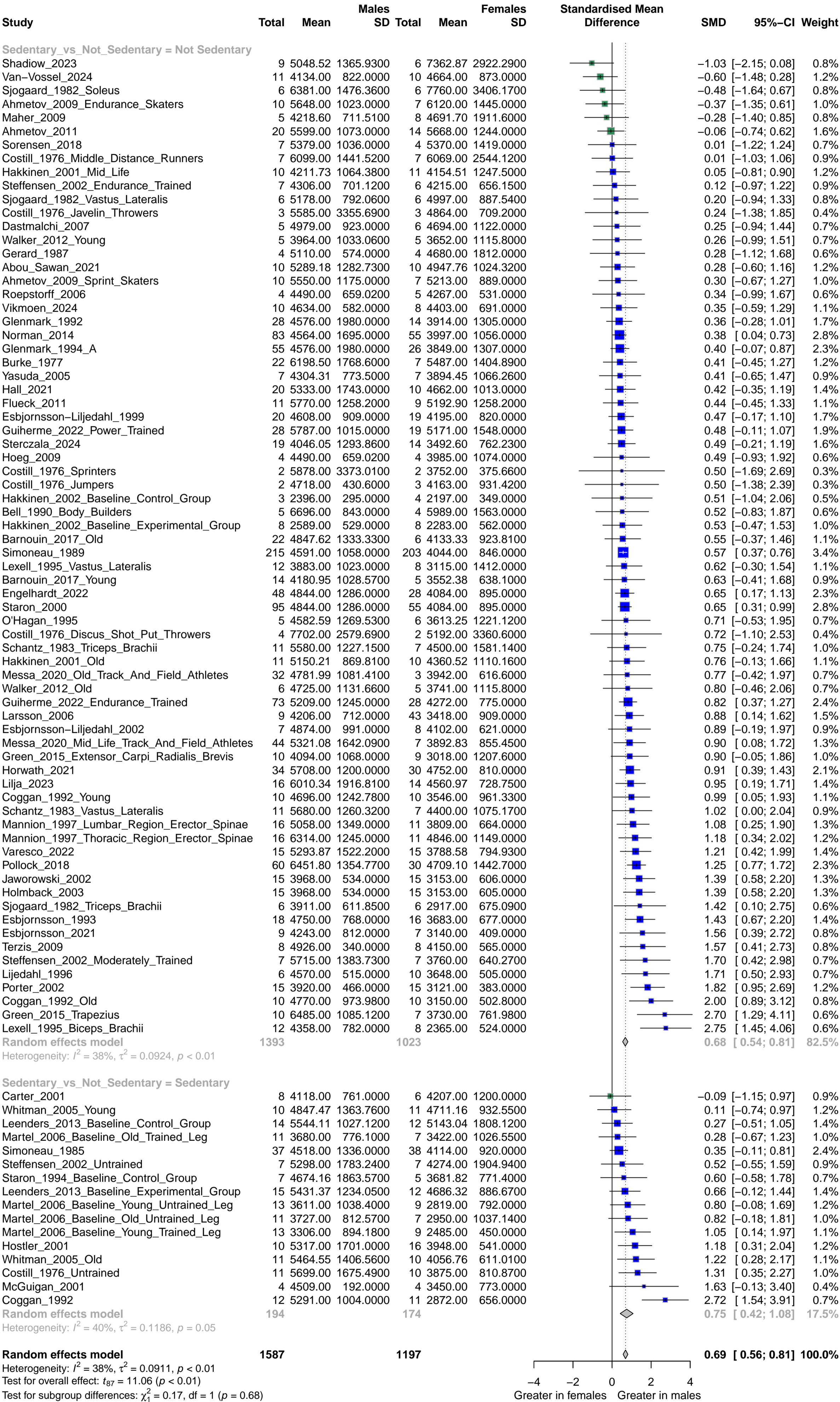

Supplement: Supplementary file 5 — Data S5. Individual forest plots of each main and subgroup outcome. [file PHY2-13-e70616-s005.pdf]
