# Supplementary material for: Sex differences in human skeletal muscle fiber types and the influence of age, physical activity, and muscle group: A systematic review and meta‐analysis
Source: Physiol Rep. 2025 Nov 2;13(21):e70616. doi: 10.14814/phy2.70616 (PMC12580412; doi:10.14814/phy2.70616)
Supplement: Supplementary file 6 — Data S6. Risk of bias tool. [file PHY2-13-e70616-s004.pdf]

<u>**Newcastle-Ottawa Scale Adapted for Cross-Sectional Studies**</u>

Selection:

1. Representativeness of the sample:
    a. Truly representative of the average in the target population. (all subjects or random sampling) *(Score: 1)*
    b. Somewhat representative of the average in the target group. (non-random sampling) *(Score: 1)*
    c. Selected group of users/convenience sample. *(Score: 0)*
    d. No description of the derivation of the included subjects (i.e., no description of the sampling strategy). *(Score: 0)*

2. Sample size:
    a. Justified and satisfactory (including sample size calculation). *(Score: 1)*
    b. Not justified. *(Score: 0)*
    c. No information provided. *(Score: 0)*

3. Non-respondents:
    a. The response rate is satisfactory (≥95%). *(Score: 1)*
    b. The response rate is unsatisfactory (<95%). *(Score: 0)*

4. Ascertainment of the screening/surveillance tool:
    a. Validated screening/surveillance (measurement) tool. *(Score: 2)*
    b. Non-validated screening/surveillance (measurement) tool, but the tool is available or described. *(Score: 1)*
    c. No description of the measurement tool. *(Score: 0)*

Comparability:

5. The potential confounders were investigated by subgroup analysis or multivariable analysis. (i.e., confounding factors controlled).
    a. The study investigates potential confounders. *(Score: 1)* Data/ results adjusted for relevant predictors/risk factors/confounders (sex and proportional area)
    b. The study does not investigate potential confounders. *(Score: 0)*

Outcome:

6. Assessment of the outcome:
    a. Independent blind assessment. *(Score: 2)*
    b. Record linkage. *(Score: 2)*
    c. Self report. *(Score: 1)*
    d. No description. *(Score: 0)*

7. Statistical test:
    a. The statistical test used to analyze the data is clearly described and appropriate. *(Score: 1)*
    b. The statistical test is not appropriate, not described or incomplete. *(Score: 0)*

Maximum of 9 points
Very Good Studies: 8-9 points
Good Studies: 6-7 points
Satisfactory Studies: 4-5 points
Unsatisfactory Studies: 0-3 points

This scale has been adapted from the Newcastle-Ottawa Quality Assessment Scale for cohort studies to provide quality assessment of cross-sectional studies[1].

---

[1] Herzog R, et al. Is Healthcare Workers' Intention to Vaccinate Related to their Knowledge, Beliefs and Attitudes? A Systematic Review. *BMC Public Health* 2013 **13**:154
