# Supplementary material for: Sex differences in human skeletal muscle fiber types and the influence of age, physical activity, and muscle group: A systematic review and meta‐analysis
Source: Physiol Rep. 2025 Nov 2;13(21):e70616. doi: 10.14814/phy2.70616 (PMC12580412; doi:10.14814/phy2.70616)
Supplement: Supplementary file 7 — Data S7. Funnel plots of each main outcome. [file PHY2-13-e70616-s002.pdf]

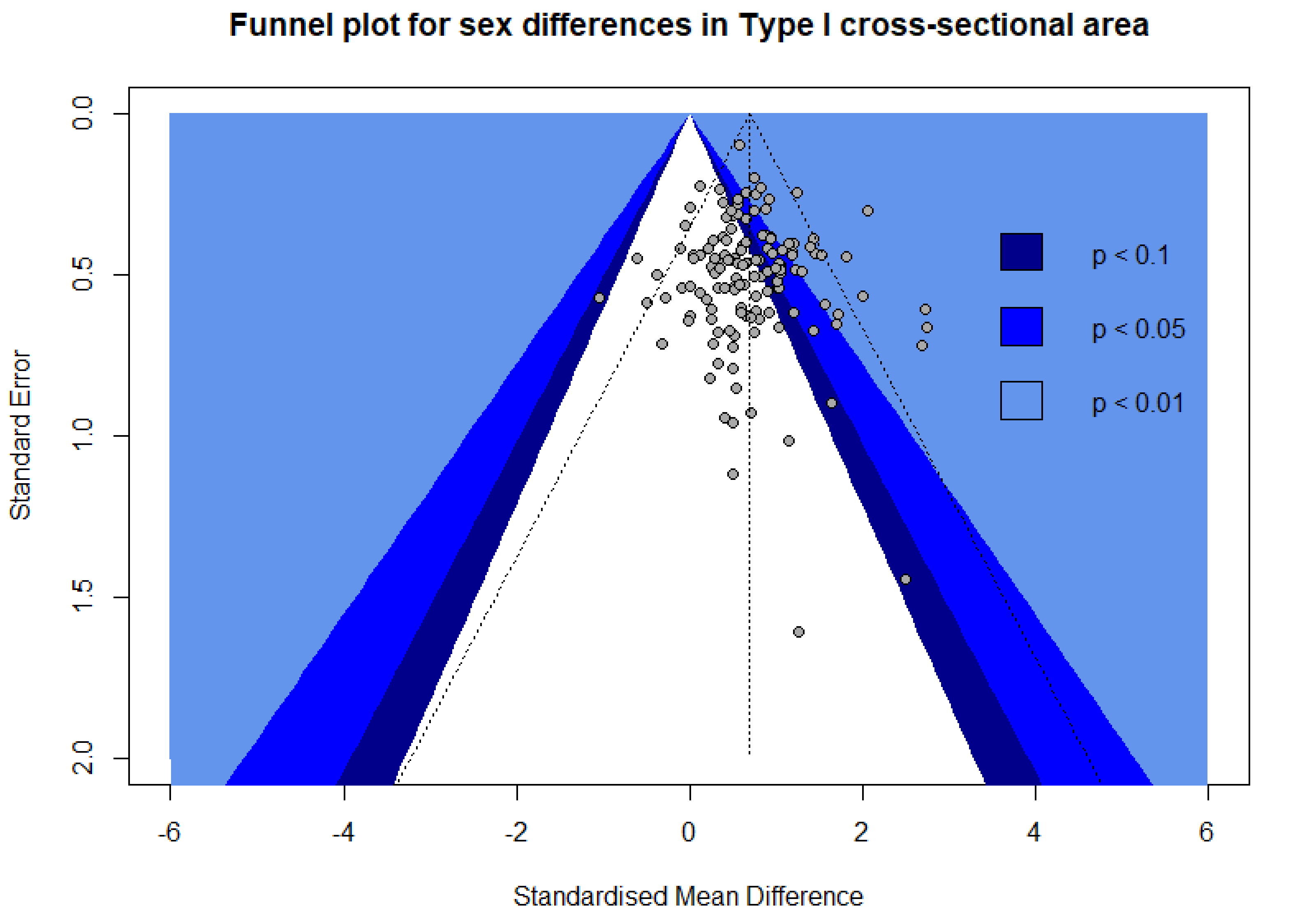

Funnel plot for sex differences in Type I cross-sectional area
Standard Error
0.0
0.5
1.0
1.5
2.0
-6
-4
-2
0
2
4
6
Standardised Mean Difference
p < 0.1
p < 0.05
p < 0.01

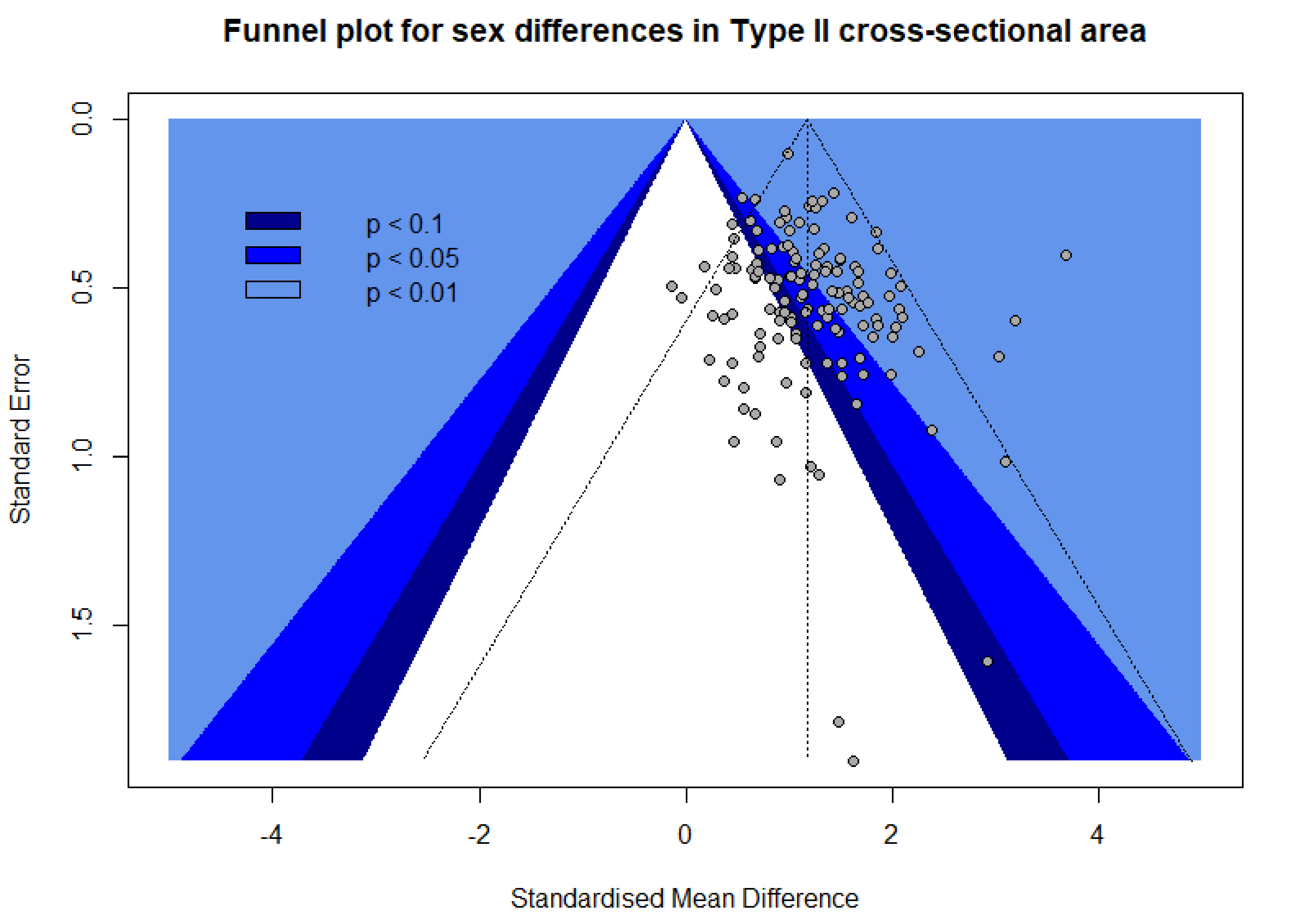

Funnel plot for sex differences in Type II cross-sectional area
p < 0.1
p < 0.05
p < 0.01
Standard Error
0.0
0.5
1.0
1.5
-4
-2
0
2
4
Standardised Mean Difference

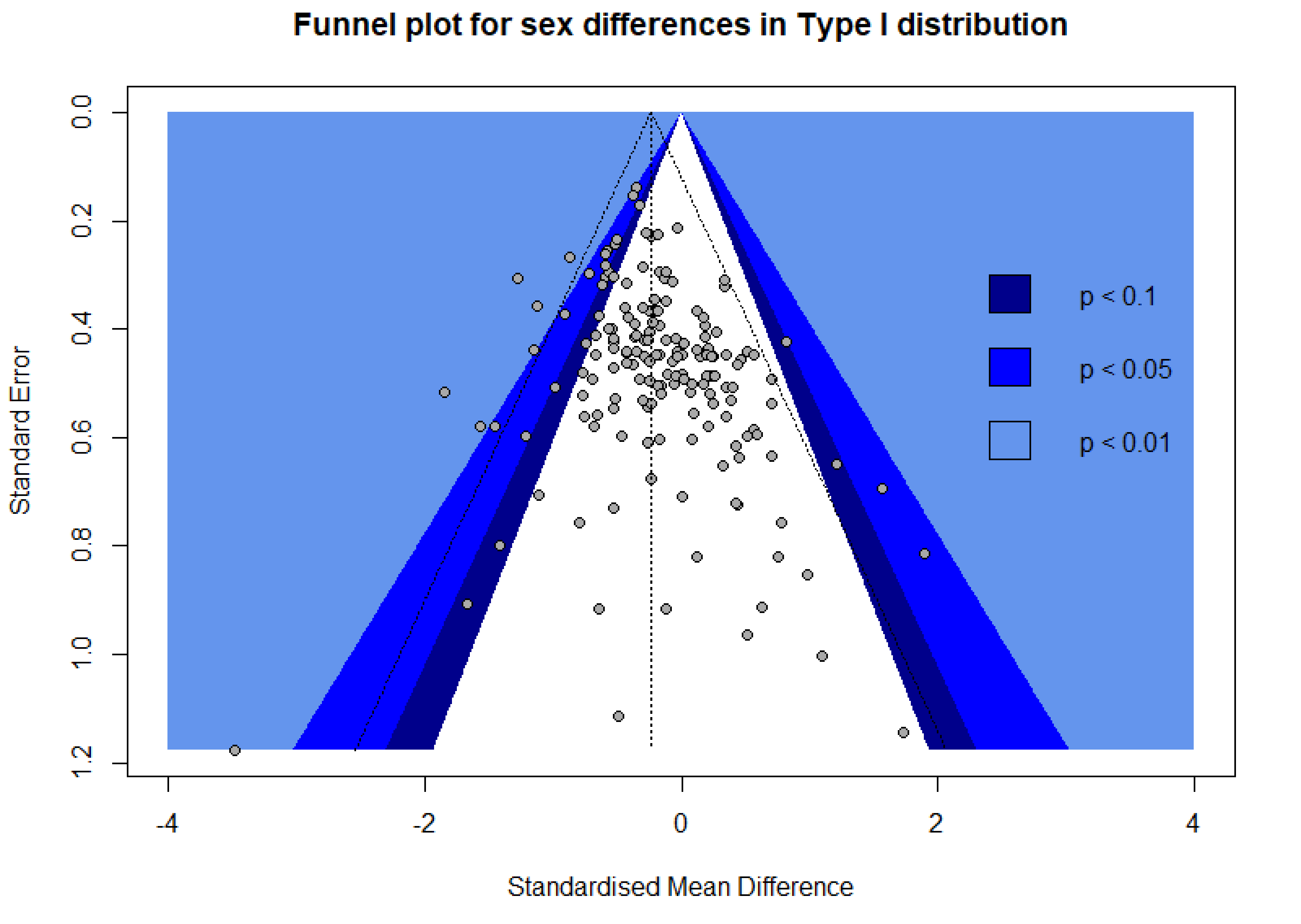

Funnel plot for sex differences in Type I distribution
Standard Error
0.0
0.2
0.4
0.6
0.8
1.0
1.2
-4
-2
0
2
4
Standardised Mean Difference
p < 0.1
p < 0.05
p < 0.01

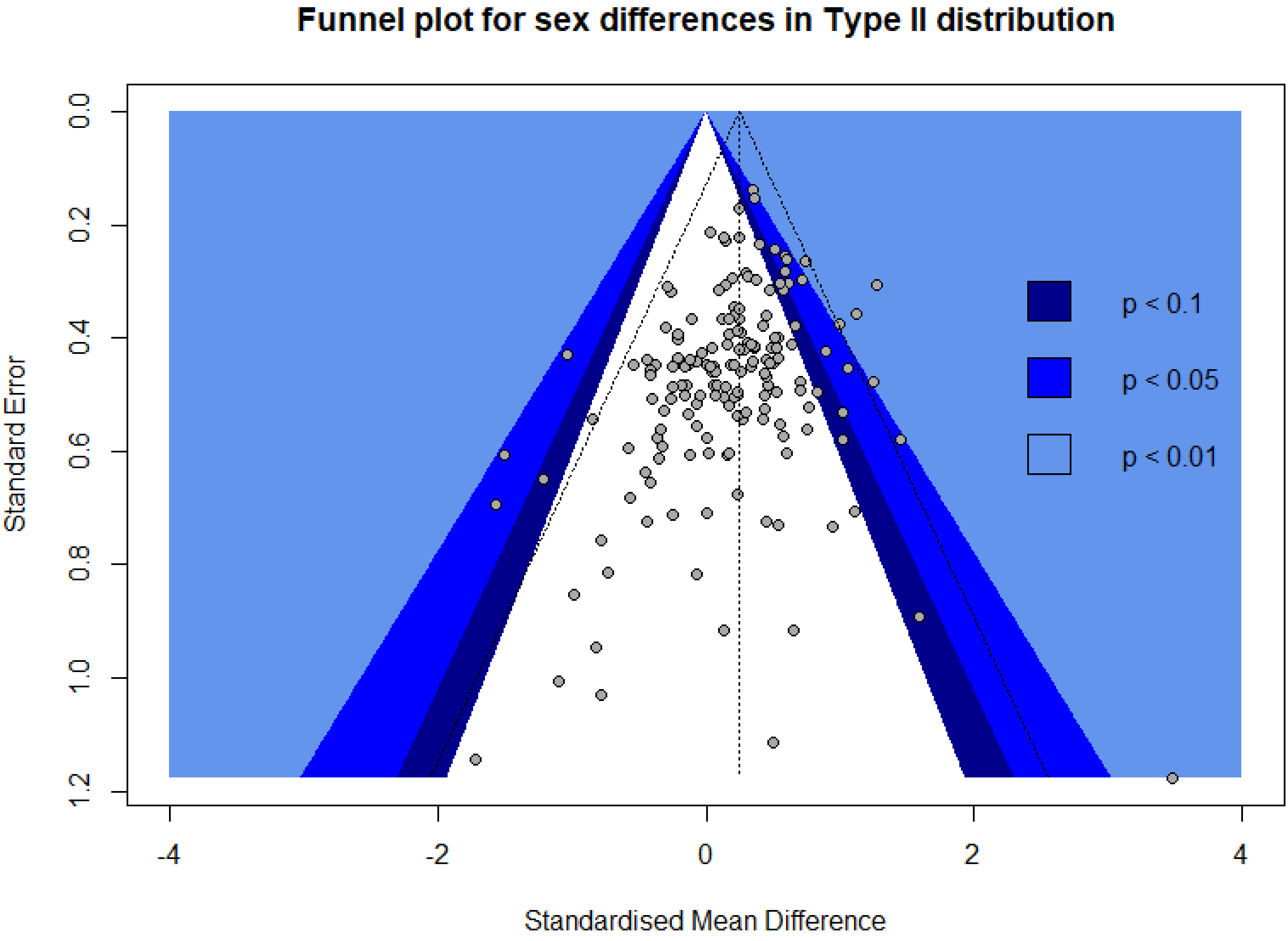
Funnel plot for sex differences in Type II distribution
Standard Error
0.0
0.2
0.4
0.6
0.8
1.0
1.2
-4
-2
0
2
4
Standardised Mean Difference
p < 0.1
p < 0.05
p < 0.01

Funnel plot for sex differences in Type I proportional area

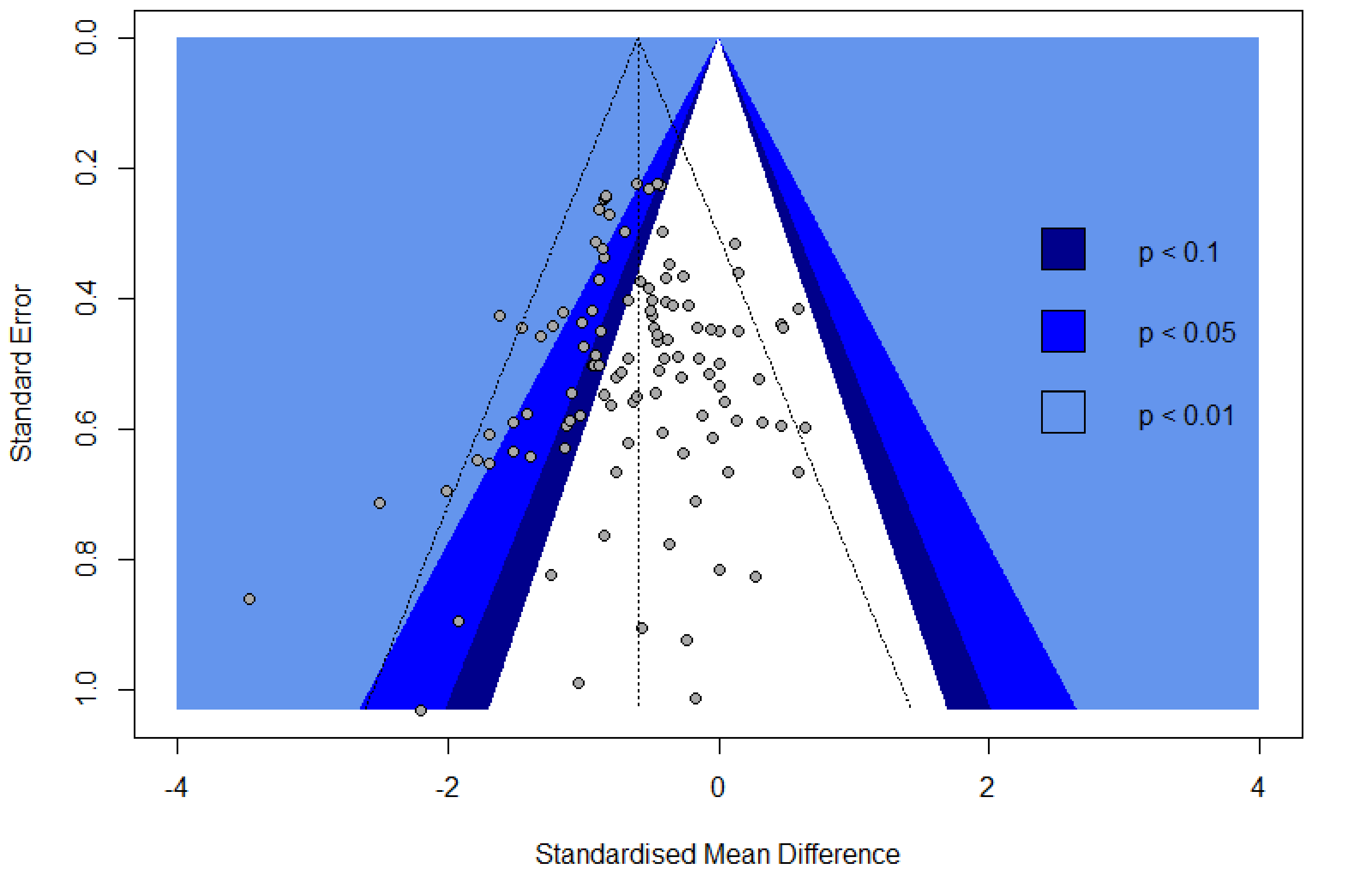

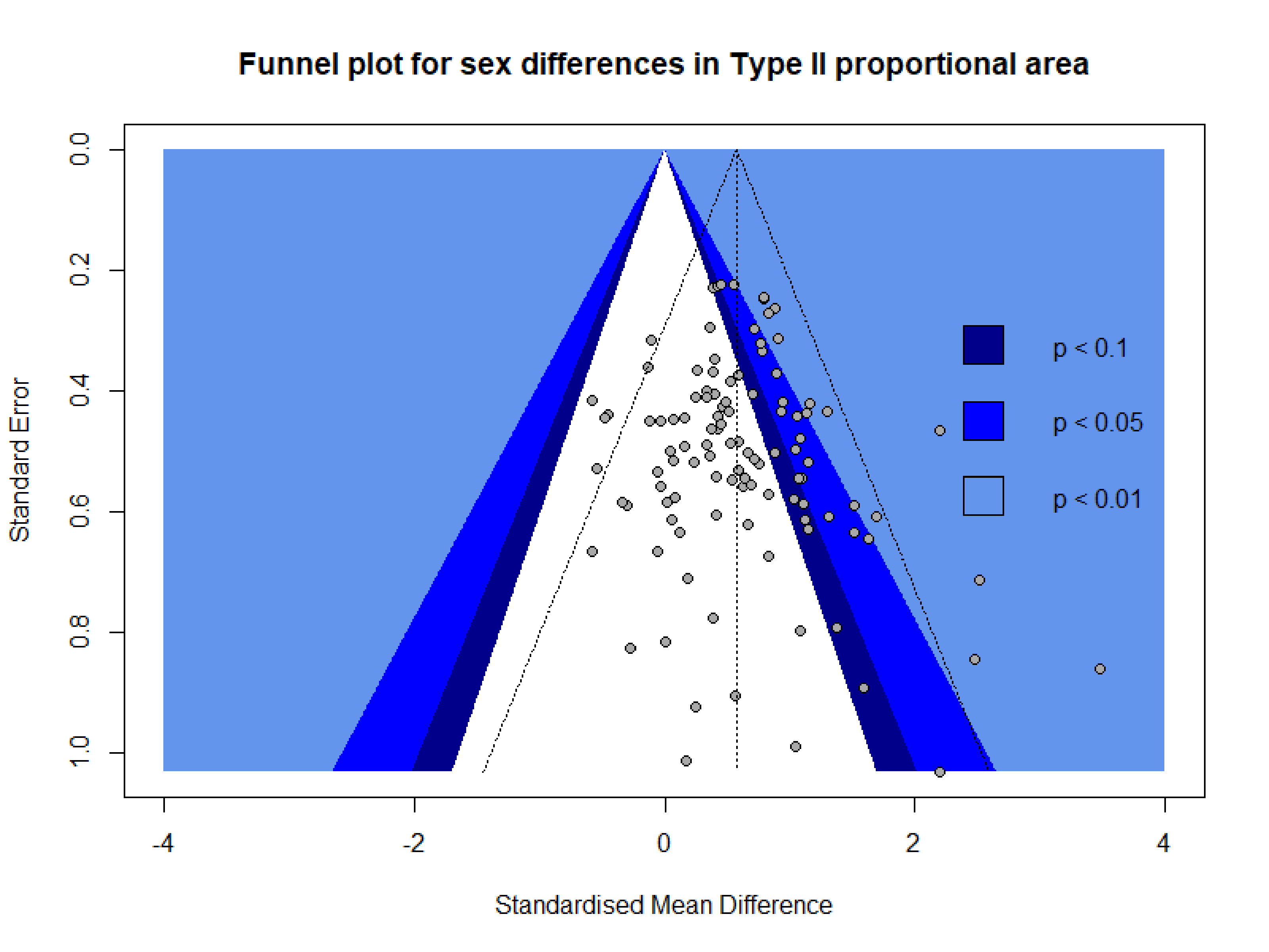

Funnel plot for sex differences in Type II proportional area
Standard Error
0.0
0.2
0.4
0.6
0.8
1.0
-4
-2
0
2
4
Standardised Mean Difference
p < 0.1
p < 0.05
p < 0.01
